# An organophotocatalytic redox-neutral strategy for late-stage drug functionalization with SO_2_ gas†

**DOI:** 10.1039/d4sc08380f

**Published:** 2025-02-13

**Authors:** Paramita Datta, Subir Maji, Prativa Biswas, Divya Jain, Partha Protim Dey, Swadhin K. Mandal

**Affiliations:** a Department of Chemical Sciences, Indian Institute of Science Education and Research (IISER) Kolkata Mohanpur Campus Nadia 741246 West Bengal India swadhin.mandal@iiserkol.ac.in

## Abstract

Sulfur dioxide (SO_2_), a widely considered air pollutant, imposes major effects on ecosystems, human health, and the climate. A promising solution combines synthetic chemistry through SO_2_ gas capture, transforming it into valuable pharmaceutical and agrochemical products such as sulfonamides, thiosulfonates, sulfonate esters, *etc.* Direct SO_2_ gas capture and its catalytic functionalization are currently limited and require redox-non-neutral transition metal-based catalysts, harsh conditions, and expensive SO_2_ substitutes/surrogates in stoichiometric amounts. This work presents a metal-free and redox-neutral organophotocatalytic strategy for direct SO_2_ gas capture and its catalytic functionalization with various fine chemicals such as amines, alcohols, and thiols. It utilizes phenalenyl-based molecule as a photocatalyst. In-depth studies (EPR, UV-Vis, and fluorescence quenching) shed light on the reaction mechanism and elucidate the complete SO_2_ activation cycle. A diverse array of bioactive motifs and clinically active molecules in the categories of antihistamine, antiplatelet, antipsychotic, antidementia, antidepressant, antioxidant, and antimalarial drugs, as well as terpenoids, natural products, amino acids, and peptides, were catalytically functionalized with gaseous SO_2_ under metal-free conditions highlighting the synthetic potential of this protocol.

## Abstract 2

We report here a metal-free, redox-neutral photocatalytic strategy of SO_2_ gas functionalization resulting in the one-pot synthesis of sulfonamides, sulfonate esters, and thiosulfonates through a photoexcited phenalenyl (PLY) ligand.

## Graphical abstract

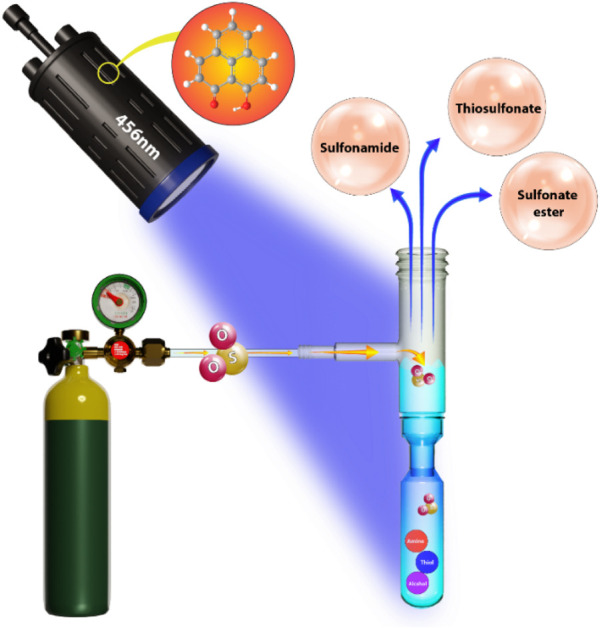

## Introduction

**Fig. 1:**
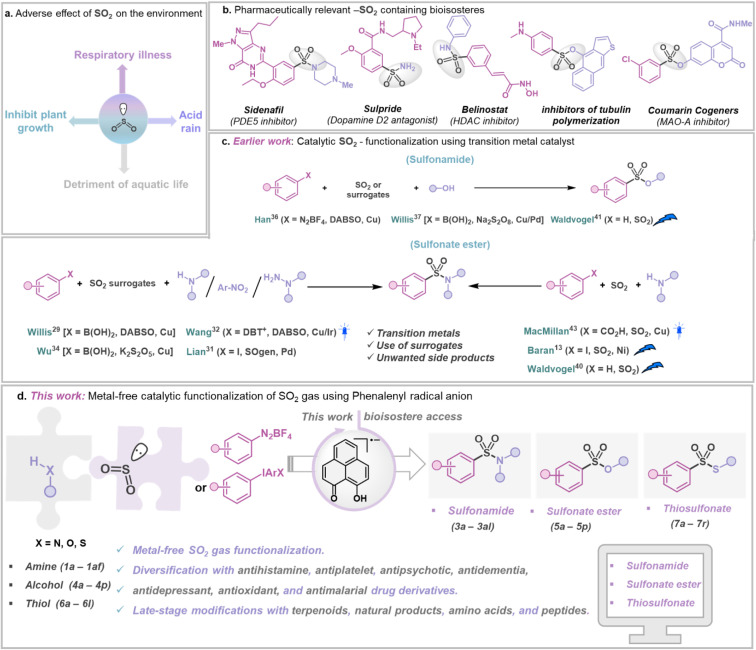
SO_2_ gas functionalization. (a) The harmful effect of sulfur dioxide gas as an air pollutant. (b) Biologically relevant SO_2_-containing molecules. (c) Earlier work: metal-catalyzed sulfinylation for the formation of various sulfonyl-containing compounds. (d) This work: organophotocatalytic SO_2_ gas functionalization using a phenalenyl-based molecule.

Accumulation of SO_2_ gas in the atmosphere poses a significant risk as it is considered a critical air pollutant and acknowledged as a pressing challenge due to its toxic nature and various environmental and health hazards (Fig. 1a).^1,2^ The substantial source of SO_2_ in the atmosphere is the burning of fossil fuels in power plants and other industrial facilities.^1^ In addition, coal burning has also been a major contributor to increased levels of SO_2_ in the atmosphere.^1^ During combustion, sulfur reacts with oxygen in the air to produce SO_2_, which further undergoes chemical reactions with other molecules to generate small acidic particulates, which, when inhaled, can penetrate human lungs, causing serious ailments such as asthma, bronchitis, *etc.* However, diverse oxidation states of S (S^IV^ and S^VI^) offer a surprising opportunity to chemists.^3,4^ Sulfur-containing compounds such as sulfonamides,^5,6^ thiosulfonates, and sulfonate esters^7^ are ubiquitous in pharmaceuticals,^8^ agrochemicals,^9^ and functional materials^10^ (Fig. 1b). Interestingly, sulfur is more common than fluorine or phosphorus in FDA-approved drugs.^11^ In this regard, utilizing gaseous SO_2_ directly in organic synthesis is hindered by its tendency to dimerize (forming dithionite) due to its moderate reduction potential (−0.78 V *vs.* Ag/AgCl).^12,13^ SO_2_, being amphoteric, can act as both Lewis acid^14^ and Lewis base,^15^ allowing it to react with electron donors and acceptors.^16^ For example, amine-SO_2_ adducts have been known for decades,^17–20^ but their synthetic applications remained limited. Moreover, the possibility of capturing SO_2_ with other electron donors, such as alcohols and thiols, has remained less explored.^21,22^ Such adducts, if integrated within a catalytic loop, can be visualized as potential reagents for direct SO_2_ capture from its source, followed by its functionalization to value-added sulfur(vi) containing organic compounds,^5–7^ but such a strategy has been rarely explored. Traditionally, sulfur(vi) containing compounds such as sulfonamides, sulfonate esters, and thiosulfonates are prepared non-catalytically by reacting nucleophiles with moisture-sensitive sulfonyl chlorides or a multicomponent approach with DABSO (1,4-diazabicyclo[2.2.2]octane-bis(sulfur dioxide) adduct) and other surrogates as reagents, limiting their storage and application.^23–28^ At present, the most common approaches involve metal-catalyzed synthesis of S(vi) containing structural motifs using DABSO, K_2_S_2_O_5_, SOgen, *etc.*, as a sulfur dioxide surrogate in stoichiometric amounts (Fig. 1c), generating an equivalent amount of chemical waste.^4,29–39^ Although SO_2_ gas offers the most atom-efficient way to introduce it in value-added organic products, direct SO_2_ capture and functionalization are still under-explored. In this context, the Baran group reported Ni-based electrocatalytic sulfinylation using SO_2_ gas.^13^ Additionally, electrochemical C–H sulfonylation was developed by the Waldvogel group to convert electron-rich arenes to valuable S^VI^ derivatives.^40–42^ Recently, the MacMillan group presented a strategy that allows aromatic acids to be decarboxylatively halosulfonylated, resulting in the synthesis of sulfonamides using a Cu-catalyst and SO_2_ gas.^43^ Notably, these methods often rely on net oxidation or reduction processes, thus representing non-redox-neutral pathways.^13,43^ Conversely, a redox-neutral strategy paves a straightforward, sustainable route to accomplish atom-economy without employing any species present solely to oxidize or reduce the catalytic system.^44,45^ In this work, we present a metal-free, redox-neutral photocatalytic strategy for direct SO_2_ gas capture resulting in the one-pot synthesis of sulfonamides or thiosulfonates, or sulfonate esters (Fig. 1d). This method offers operational simplicity as it does not need any addition of an external oxidant or reductant (*i.e.* redox-neutral).^44,45^ It uses a phenalenyl^46,47^ (PLY)-based molecule as a photo-oxidant. In this process, an *in situ* formed SO_2_-nucleophile adduct having photo-reductant properties is formed by transferring an electron to the photo-excited phenalenyl-based molecule, which in turn activates the other reaction partner by a single electron transfer (SET) process. The synthetic applicability of this strategy is showcased with broad substrate scope, making it highly attractive for catalytic functionalization of SO_2_ gas with various fine chemicals such as amines, alcohols, and thiols resulting in a library of SO_2_-containing organic compounds such as sulfonamides, sulfonate esters and thiosulfonates. Using this method, several commercially available drug molecules in the classes of antihistamine, antiplatelet, antipsychotic, antidementia, antidepressant, antioxidant, and antimalarial, as well as from various biologically active complex molecules such as terpenoids, natural products, amino acids, and peptides, were functionalized using SO_2_ gas directly.

## Results and discussion

At first, using morpholine (1a) and diphenyliodonium chloride (2a) as model substrates, we investigated the multicomponent SO_2_ gas incorporation reaction photochemically to ascertain the optimal reaction conditions (Table 1). After several trials (Table 1 and see the ESI, Tables S1–S4†), we observed the formation of the sulfonamide product (3a) with 84% NMR yield in MeCN (1.5 mL, 0.2 M) when blue light (*λ* = 456 nm, Kessil lamp, 40 W) was irradiated under SO_2_ gas (1 atm) for 12 h using PLY (O,O) (5 mol%) as the photocatalyst and Na_2_CO_3_ (2 equiv.) as the base. Several control experiments demonstrated that PLY (O,O) and a blue LED were indispensable for this reaction (Table 1 and see the ESI for further details, Table S4†). For example, the absence of the PLY (O,O) catalyst did not produce an appreciable yield of the corresponding products such as 3a (6% conversion, Table 1, entry 4). Screening of other organic photocatalysts such as 4CzIPN and 10-phenylphenothiazine (PTH), as well as riboflavin, Eosin Y, rose bengal, and dibromo substituted perylene bisimide, led to inferior yields as compared to PLY (O,O) under the optimized reaction conditions (up to 19%, see the ESI, Table S2†). This protocol appeared to be selective regarding the choice of solvents; for example, replacing MeCN with THF, DMF, DMSO, toluene, or 1,4-dioxane provided 3a in lower yields (12–37%), see the ESI, Table S3†. Notably, such a strategy does not require any external oxidant or reductant, thus enabling a redox-neutral chemical process. We next checked if such activation of SO_2_ gas can be coupled with alcohols or thiols instead of amines, converting them into diverse SO_2_-containing structural motifs. As a result, we tested the feasibility of this reaction by replacing amines with alcohols or thiols under optimized reaction conditions which led to sulfonate esters (5a–5p, 39–69%) or thiosulfonates (7a–7r, 58–82%). These results immediately indicate the power of this protocol for the incorporation of gaseous SO_2_ molecules into a range of fine organic chemicals (amines, alcohols, and thiols), opening a possibility to synthesize a diverse library of sulfonamides, sulfonate esters, and thiosulfonates adopting the organophotocatalytic and redox-neutral strategy using SO_2_ gas.

**Table 1:** Organophotocatalytic SO_2_ activation. An optimized reaction scheme for PLY (O,O) catalyzed SO_2_ gas functionalization with morpholine (1a) and diphenyliodonium chloride (2a)^a^

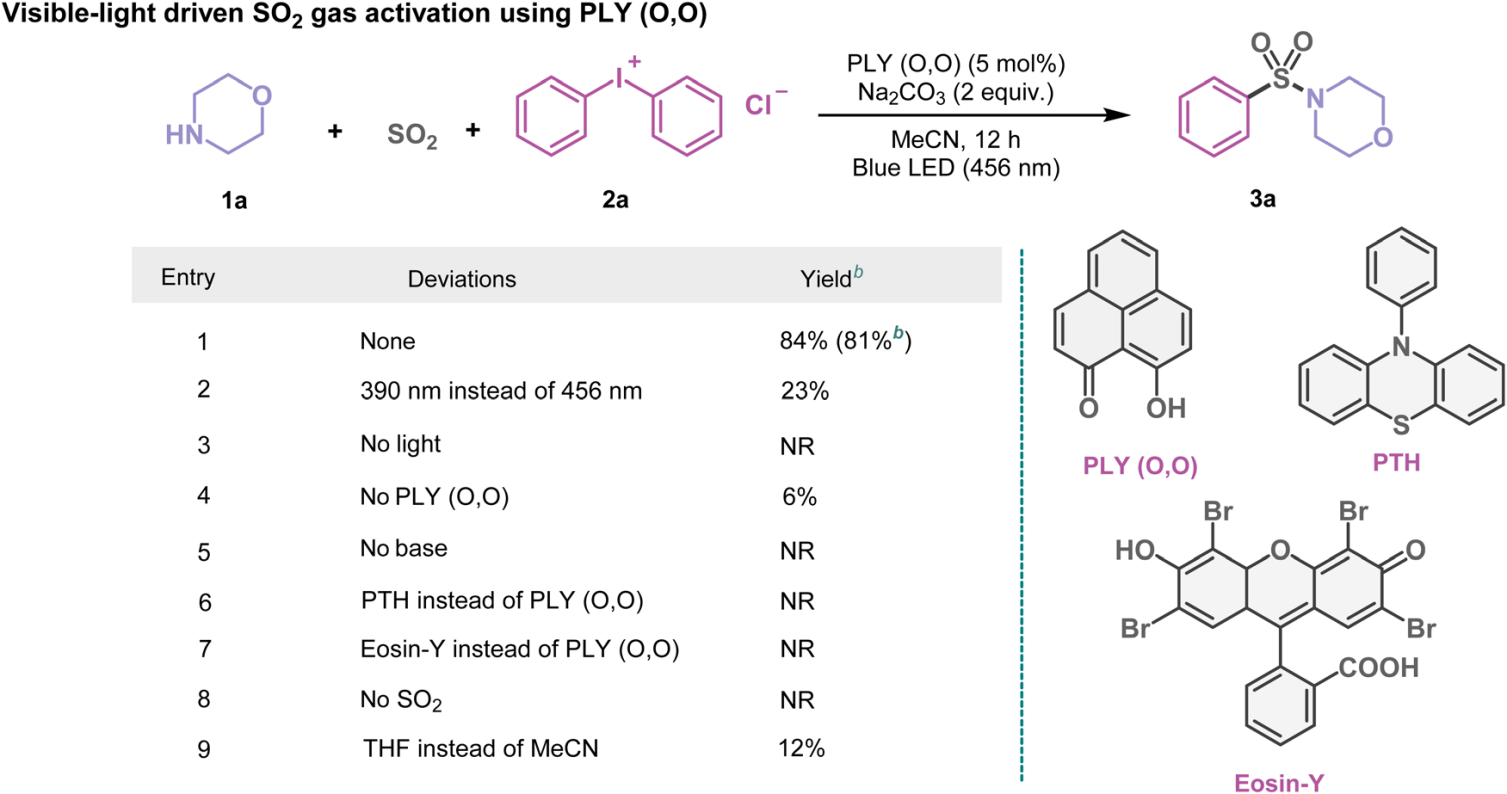

a Reaction conditions: 1a (0.48 mmol, 1 equiv.), 2a (1.5 equiv.), PLY (O,O) (5 mol%), Na_2_CO_3_ (1.5 mL, 0.2 M) and SO_2_ (1 atm) 12 h, Kessil blue LED (456 nm). Yield was determined by ^1^H NMR spectroscopy with 1,4-dimethoxybenzene as the internal standard.

b Isolated yield. NR = no reaction. See the ESI for further details.

**Scheme 1:**
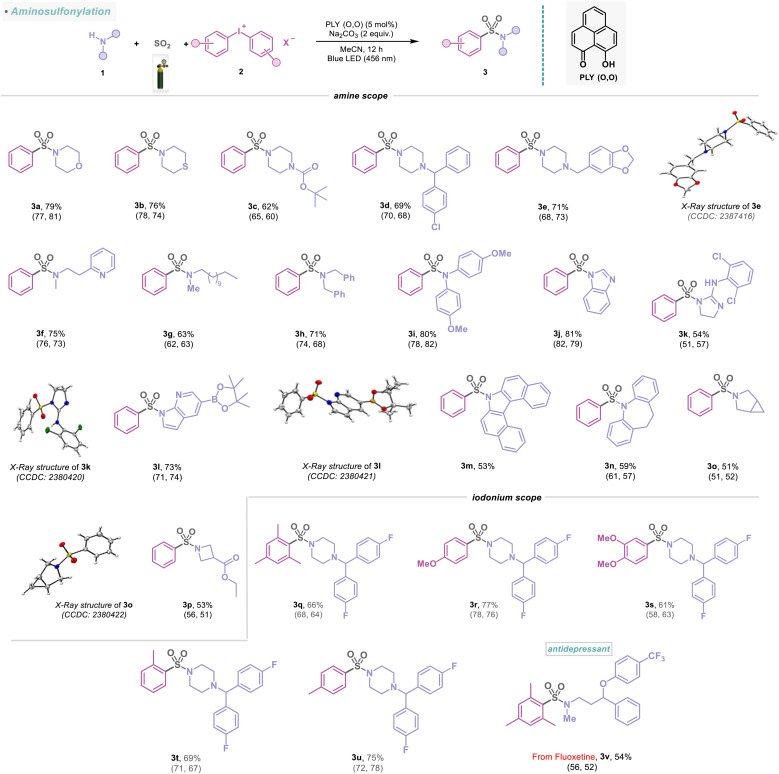
Scope of amines and iodonium salts in the visible-light-induced SO_2_ activation reaction. Reaction conditions: amines (1) (0.48 mmol, 1 equiv.), iodonium salts (2) (0.72 mmol, 1.5 equiv.), PLY (O,O) (0.024 mmol, 5 mol%), sodium carbonate (0.96 mmol, 2 equiv.), MeCN (1.5 mL, 0.2M) under SO_2_ atmosphere (1 atm) for 12 h, Kessil blue LED (456 nm). Isolated yields are average yields calculated from the yields obtained from two independent catalytic runs, shown in the parenthesis.

**Scheme 2:**
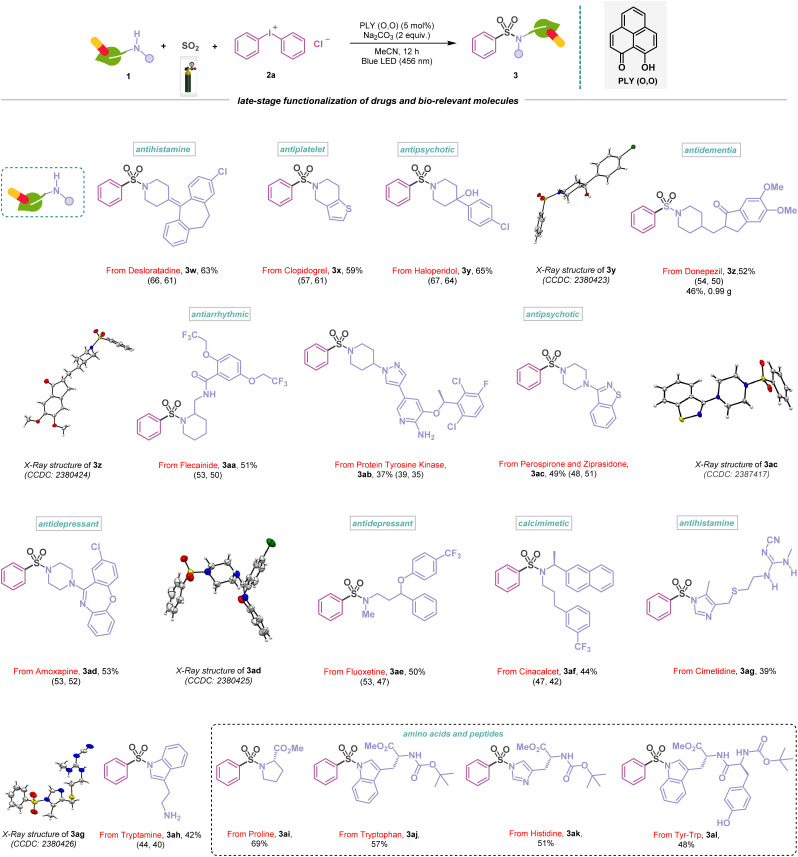
Functionalization of natural products and drugs in the visible-light-induced aminosulfonylation reaction. Reaction conditions: amines (1) (0.48 mmol, 1 equiv.), 2a (0.72 mmol, 1.5 equiv.), PLY (O,O) (0.024 mmol, 5 mol%), sodium carbonate (0.96 mmol, 2 equiv.), MeCN (1.5 mL, 0.2M) under SO_2_ atmosphere (1 atm) for 12 h, Kessil blue LED (456 nm). Isolated yields are average yields calculated from the yields obtained from two independent catalytic runs, shown in the parenthesis.

**Scheme 3:**
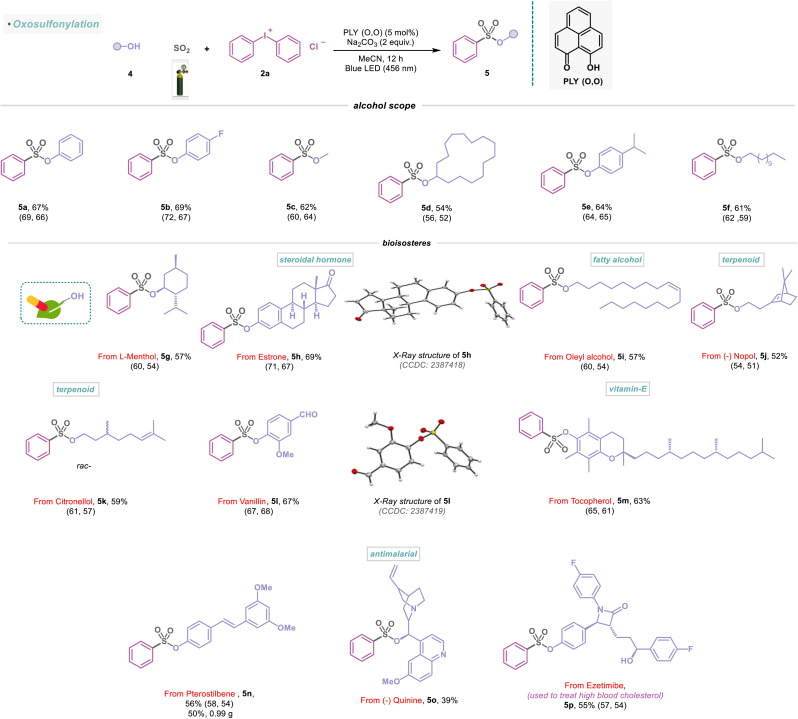
Scope of alcohols in the visible-light-induced SO_2_ activation reaction. Reaction conditions: alcohols (4) (0.48 mmol, 1 equiv.), 2a (0.72 mmol, 1.5 equiv.), PLY (O,O) (0.024 mmol, 5 mol%), sodium carbonate (0.96 mmol, 2 equiv.), MeCN (1.5 mL, 0.2M) under SO_2_ atmosphere (1 atm) for 12 h, Kessil blue LED (456 nm). Isolated yields are average yields calculated from the yields obtained from two independent catalytic runs, shown in the parenthesis.

**Scheme 4:**
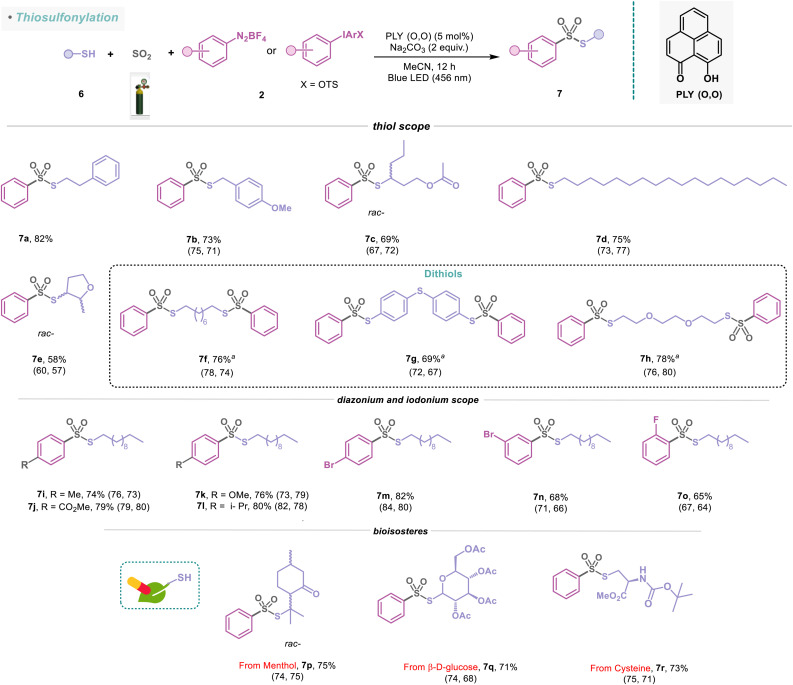
Substrate scope for the thiosulfonylation reaction. Reaction conditions: thiols (6) (0.48 mmol, 1 equiv.), 2 (0.72 mmol, 1.5 equiv.), PLY (O,O) (0.024 mmol, 5 mol%), sodium carbonate (0.96 mmol, 2 equiv.), MeCN (1.5 mL, 0.2M) under SO_2_ atmosphere (1 atm) for 12 h, Kessil blue LED (456 nm). ^*a*^Phenyl diazonium salt 2i (3 equiv), sodium carbonate (4 equiv.). For 7a–7h and 7k–7r, diazonium salts and, in the case of 7i–7j, iodonium salts were used. Isolated yields are average yields calculated from the yields obtained from two independent catalytic runs, shown in the parenthesis.

The simplicity and diversity of this metal-free PLY (O,O) catalyzed direct SO_2_ gas-functionalization prompted us to carry out a detailed evaluation of the substrate scope, which revealed broad compatibility and efficiency with a wide array of functionalized amines, alcohols, and thiols (Schemes 1–4). At first, we focused on the synthesis of sulfonamides based on the variation of amines and iodonium salts. To demonstrate that electronically and sterically unbiased substrates are capable of this reaction, morpholine (1a) and thiomorpholine (1b) afforded 76–79% yields of the corresponding products (3a–3b). Additionally, piperazine rings are common motifs in medicinal agents, and a range of these cyclic amines are substituted with *tert*-butyloxycarbonyl (BOC) (1c), aryl (1d) and heteroaryl (1e) groups were efficiently converted into sulfonamides (3c–3e, 62–71%), and the structure of 3e was further confirmed by X-ray crystallography (Scheme 1). *N*-Methyl amines (1f–1g) and dibenzyl amine (1h) also provided the corresponding sulfonamides (3f–3h) in 63–75% yields. Moreover, this approach proved competent for the sulfonylation of aromatic amine (1i) featuring an electron-donating methoxy group producing the corresponding sulfonamide (3i) in 80% yield. Significantly, this multicomponent reaction was well tolerated to amines bearing different heterocycles (1j–1l) and polycyclic skeletons (1m–1n) affording sulfonamide products (3j–3n) in 53–81% yields, which highlights the potential synthetic utility of this method in medicinal chemistry. The structures of 3k and 3l were further confirmed by X-ray crystallography (Scheme 1). In addition, the protocol is also amenable to bridgehead systems such as 3-azabicyclo[3.1.0]hexane (1o) and delivering a yield of 3o in 51%, and the structure of 3o was reconfirmed by X-ray analysis (Scheme 1). Remarkably, a strained four-membered-ring amine, such as the azetidine system (1p), afforded this transformation into 3p, with a 53% yield. The synthetic scope of the aminosulfonylation was further examined with an array of unsymmetrical diaryliodonium salts (Scheme 1). The reaction enabled a smooth conversion of a number of iodonium salts (2b–2f, see the ESI; Fig. S7†) to the corresponding sulfonamide derivatives (3q–3v) with 54–77% yield. However, our catalytic protocol did not work for primary amines. To highlight the compatibility of the aminosulfonylation reaction with pharmaceutically relevant molecules, we explored different amine derivatives that are either active pharmaceutical ingredients (APIs) or closely related derivatives (Scheme 2). For example, a series of complex substituted piperidine derivatives allowed the incorporation of amine fragments from antihistamine (desloratadine, 3w, 63%), antiplatelet (clopidogrel, 3x, 59%), antipsychotic (haloperidol, 3y, 65%), antidementia (donepezil, 3z, 52%), and antiarrhythmic (flecainide, 3aa, 51%) agents. In addition to this, the molecular structures of 3y and 3z were confirmed by X-ray crystallography (Scheme 2). To further demonstrate the synthetic practicality of this metal-free, redox-neutral SO_2_ functionalization, the five mmol scale-up reaction smoothly delivered product 3z in 46% (0.99 g) under standard reaction conditions (Scheme 2). Notably, the active pharmaceutical ingredients (APIs) of the protein-tyrosine kinase responsible for regulating cell functions, such as cell-to-cell signaling, growth, differentiation, *etc.*, have also been functionalized (3ab, 37%). In addition, piperazines that feature in the atypical antipsychotics perospirone and ziprasidone (1w), as well as antidepressant amoxapine (1x), resulted in the desired products (3ac–3ad) in 49–53% yield and were reconfirmed with an X-ray study (Scheme 2). Indeed, acyclic *N*-methyl amines, which are the APIs in the antidepressant fluoxetine (1y) and calcimimetic cinacalcet (1z), were also viable candidates, affording 44–50% yield of the corresponding products (3ae–3af). Likewise, we observed that antihistamine (cimetidine, 3ag, 39%) and antibipolar (tryptamine, 3ah, 42%) agents were also effectively used in this transformation and were reconfirmed by X-ray analysis (Scheme 2). The preparation of a diverse set of sulfonamides derived from proline (1ac), tryptophan (1ad), and histidine (1ae) derivatives were successful and further demonstrated the functional group tolerance of this photocatalytic method (3ai–3ak, 51–69%). In addition, 3al was formed in a 48% yield, demonstrating the activation of the dipeptide (Tyr–Trp, Scheme 2).

Having explored different amines and their substitution patterns, we now turned our attention to achieving the photocatalyzed sulfonylation of alcohols (Scheme 3). To the best of our knowledge, alcohol as a nucleophile with SO_2_ gas to synthesize sulfonate ester, a common fragment in many pharmaceuticals and other bioactive compounds, remained less explored.^21,22^ Gratifyingly, under the standard reaction conditions, aromatic and aliphatic alcohols (4a–4f) were successfully functionalized, providing efficient access to sulfonate ester derivatives (5a–5f) with 54–69% yield (Scheme 3). In addition, naturally occurring monoterpene l-menthol (4g) could be incorporated to provide the desired sulfonate ester product (5g) in 57% yield. Remarkably, estrone, a steroidal hormone, was found to be a viable substrate in the sulfonylation reaction (5h, 69%) and reconfirmed by X-ray analysis (Scheme 3). The natural product, oleyl alcohol (4i), as well as 4j and 4k were converted to the corresponding sulfonate esters (5i–5k) in 52–59% yield (Scheme 3). Vanillin (used as a flavoring agent) engaged in this transformation, provided the desired product (5l) in 67% yield and was studied with X-ray analysis (Scheme 3). Furthermore, we synthesized the sulfonate ester derivatives (5m–5o) from vitamin E-derived α-tocopherol, commercially available antioxidant pterostilbene, and antimalarial drug quinine with 39–63% yield under metal-free conditions (Scheme 3). Furthermore, the transformation of 4n into 5n was successfully conducted on a 5 mmol scale (5n, 50%, 0.99 g). We next attempted to extend this strategy to functionalize other important drugs, such as ezetimibe, used to treat high blood cholesterol, affording the desired products 5p in 55% isolated yield (Scheme 3).

To further demonstrate the applicability of this method toward the formation of SO_2_-rich molecules from readily accessible thiols, we undertook the synthesis of several thiosulfonate molecules with diazonium and iodonium salts under standard reaction conditions (Scheme 4). Thiosulfonates constitute sources of sulfur in organic synthesis owing to their powerful capabilities as sulfenylating agents.^48^ They are employed in the polymer industry, photographic processes, bioanalytical and biomedical research, and the development of biosensors.^49^ In this protocol, aliphatic thiols such as 6a and 6b were well tolerated, delivering the corresponding thiosulfonates (7a–7b) with 73–82% yield. The branched thiol 6c and long-chain aliphatic thiol (6d) were converted to the corresponding sulfonylated products (7c–7d) in 69–75% isolated yield. The reaction was generally quite efficient for heterocyclic substrates (7e, 58%). Disubstituted aliphatic and aromatic thiols were also adaptable with this protocol, yielding the thiosulfonate products (7f–7h) in 69–78% yields (Scheme 4). We further established that the sulfonylation protocol is efficient for aryl(2,4,6-trimethoxyphenyl)iodonium salts (2g–2h, see the ESI; Fig. S7†), which furnished the desired thiosulfonate products (7i–7j) in 74–79% yields with 1-undecanethiol (6i). Under the standard reaction conditions, diazonium salts containing electron-donating and electron-withdrawing groups at the *para*-position provided 76–82% yields of the sulfonylated products (7k–7m). Furthermore, we inquired whether the position of the substituents in diazonium salts has any influence on the outcome of this PLY (O,O) catalyzed sulfonylation reaction (Scheme 4). Accordingly, *meta*-substituted bromo (2m) and *ortho*-substituted fluoro substrates (2n) were evaluated for the sulfonylation reaction, resulting in 65–68% isolated yields of the corresponding products (7n-7o). Finally, we sought to apply our thiosulfonylation concept to bio-relevant thiol derivatives with phenyl diazonium salt (2i). Thiol derived from naturally occurring bioactive compounds such as menthol, having medicinal benefits, for example, relieving joint and muscle pain, afforded 7p in 75% yield. Also, carbohydrate and amino acid derivatives such as β-d-glucose (6k) and cysteine (6l) were successfully employed for this photocatalytic reaction, affording 7q–7r in 71–73% yields (Scheme 4). These examples highlight the broad tolerance of this strategy to structural complexity and the unique advantages of amines, alcohols, and thiols as synthons in a programmable synthetic sequence, potentially enabling the synthesis of valuable libraries of sulfonamides, sulfonate esters, and thiosulfonates that can be utilized as pharmacophores or subjected to further derivatization.

**Fig. 2:**
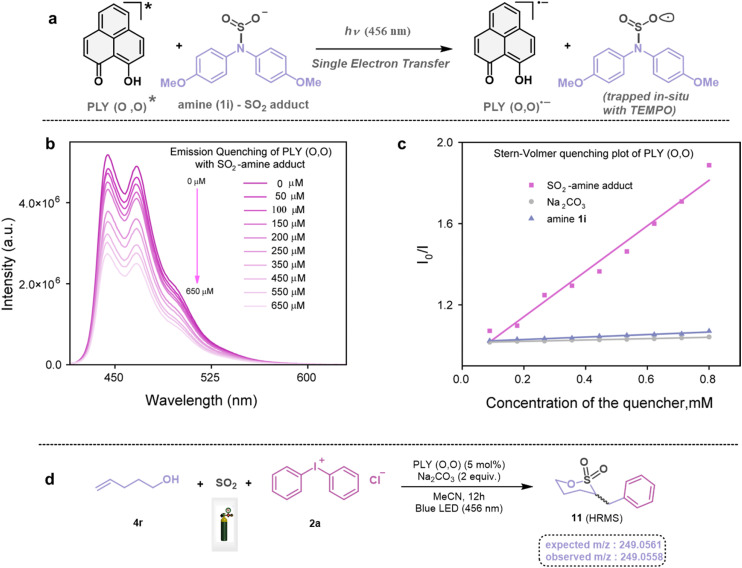
(a) Photoinduced generation of PLY (O,O) radical anions. (b) Steady-state emission spectra of PLY (O,O) with the gradual addition of the SO_2_-amine adduct. (c) Stern–Volmer quenching plot of PLY (O,O) with different quenchers, SO_2_-amine adduct, amine (1i), and base (Na_2_CO_3_). (d) Radical clock experiment.

**Fig. 3:**
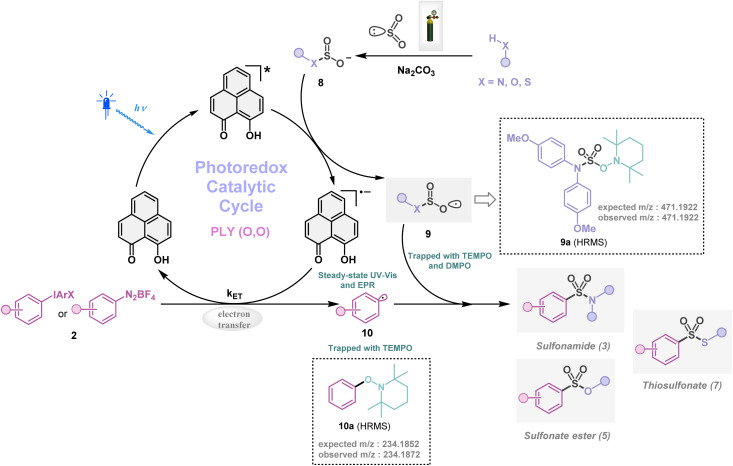
Proposed mechanistic pathway for organophotocatalytic SO_2_ activation.

Next, we investigated the plausible mechanism of this reaction, and we proposed the pathway outlined in Fig. 3 based on various spectroscopic investigations and control studies. The catalytic cycle begins with irradiation of PLY (O,O) with a blue LED (456 nm), giving rise to an excited state species PLY (O,O)*, which can function as a potent single-electron oxidant 
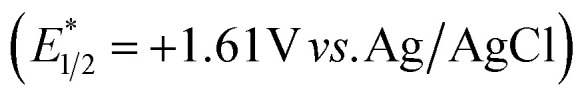
 as known in the literature.^50–53^ Simultaneously, the amine can form an adduct with SO_2_, and in the presence of a base, it can result in an anionic amine-SO_2_ adduct which is known in the literature.^17–19^ To realize such adduct formation between SO_2_ and amine in the present case, UV-Vis spectroscopic studies were conducted. When SO_2_ gas was bubbled into the solution of amine (1i) and base Na_2_CO_3_ in acetonitrile, the solution colour changed from colourless to orange (see the ESI, Fig. S15†). The UV-Vis absorption kinetic studies show that the 260–300 nm band intensity increases with time (see the ESI, Fig. S16†).^18,20^ This background knowledge prompted us to propose an electron transfer from the anionic amine/alcohol/thiol-SO_2_ adduct (8) to generate PLY (O,O)˙^−^ and sulfonyl radical intermediate (9). The interaction of the SO_2_-amine adduct with the photoexcited PLY (O,O)* was studied using steady-state fluorescence experiments. After the gradual addition of the SO_2_-amine adduct (50–650 μM), we observed the quenching of PLY (O,O) emission, as shown in Fig. 2b. The Stern–Volmer plot showed that PLY (O,O) was gradually quenched near the diffusion limit (*k*_q_ = 3.88 × 10^9^ M^−1^s^−1^) on the addition of the SO_2_-amine adduct, and linear correlation was found^51–53^ (Fig. 2c). This type of emission quenching is indicative of an excited state electron transfer process and implies that the SO_2_-amine adduct is the sole quencher of the excited state of the photocatalyst. In addition to this, the generation of PLY (O,O)˙^−^ was established by the steady-state absorption experiments (see the ESI, Fig. S17 and S18†) and EPR spectroscopic studies (see the ESI for details, Fig. S19 and S20†). During the absorption study, a new band with vibronic features in the region 460–540 nm appeared under constant irradiation with an external 456 nm LED, consistent with the generation of PLY-based radical species (see the ESI, Fig. S17† for details).^51–53^ This may be attributed to the electron transfer from the anionic SO_2_-amine adduct to the excited state of PLY (O,O), forming a PLY-based radical anion. In addition to this, the X-band electron paramagnetic resonance (EPR) spectrum was recorded at 77 K with a fresh solution of PLY (O,O) and the SO_2_-amine adduct of 1i in MeCN with 456 nm light irradiation for 10 min, which reconfirmed the generation of a PLY-based radical anion with a *g* value of 2.0071 (see the ESI, Fig. S19† for details),^51–53^ while, in another control reaction between PLY (O,O) and the SO_2_-amine adduct of 1i in MeCN without any light irradiation, we did not observe any EPR signal (see the ESI, Fig. S20†). Therefore, from these spectroscopic studies, it may be concluded that there is an electron transfer between the SO_2_-amine adduct and photoexcited PLY (O,O), and thus the SO_2_-amine adduct serves as the source of electrons, *i.e.*, the photoreductant, in the aminosulfonylation reaction. To shed further light onto this mechanistic proposal, we were successful in trapping several sulfonyl radical intermediates formed from the SO_2_-amine/SO_2_-alcohol/SO_2_-thiol adduct using (2,2,6,6-tetramethylpiperidin-1-yl)oxidanyl (TEMPO) or BHT (butylated hydroxytoluene) or 5,5-dimethyl-1-pyrroline *N*-oxide (DMPO) (9a–9e) and were characterized by HRMS (High-Resolution Mass Spectrometry), and see the ESI, Fig. S8–S12† for details. These observations lend further credence to the proposed photoinduced electron transfer events outlined in Fig. 3. Subsequently, the reduced PLY (O,O)˙^−^ can undergo a single electron transfer (SET) to the aryl source (2) (aryl diazonium or diaryliodonium salts), resulting in an aryl radical (10) which has been proposed based on our earlier studies.^47,50,51^ A TEMPO trapping experiment was performed, and the resultant TEMPO-trapped aryl radical intermediate (10a) was characterized by HRMS, which highlighted the generation of aryl radical species in the PLY (O,O) catalyzed sulfonylation reaction (see the ESI, Fig. S13†). Furthermore, a radical clock experiment was conducted using 4-penten-1-ol (4r) with diphenyliodonium chloride (2a), which provided the cyclized product 3-benzyl-1,2-oxathiolane 2,2-dioxide (11), characterized with HRMS (Fig. 2d, for further details, see the ESI, Fig. S14†) confirming the radical-mediated pathway. The catalytic cycle is finally closed by radical–radical recombination between 9 and 10, which affords the SO_2_-containing products (3, 5, and 7). Notably, the electron transfer between photoexcited PLY (O,O) and alcohol-SO_2_, as well as the thiol-SO_2_ adduct, was reconfirmed with fluorescence quenching experiments and *in situ* UV-Vis study (see the ESI for further details, Fig. S18, S24 and S25†).

## Conclusions

In this work, we present a highly efficient, one-step redox-neutral strategy for direct SO_2_ gas functionalization without transition metal-based catalysts under surrogate-free conditions. This method utilizes a photoexcited phenalenyl (PLY) ligand to synthesize sulfonamides, thiosulfonates, and sulfonate esters. This strategy highlights the synthetic utility of direct SO_2_ functionalization reactions along with the tolerance of various functional groups and motifs, such as pyridine, thiophene, ketone, thiazole, imine, amide, ether, nitrile, aldehyde, alkene, quinoline, and ester associated with complex molecules. The examples presented showcase a variety of amines, alcohols, and thiols incorporated in this study that are either active pharmaceutical ingredients (APIs), or closely related derivatives of antihistamine, antiplatelet, antipsychotic, antidementia, antidepressant, antioxidant, antimalarial, and antibipolar drugs which can be effectively converted into corresponding sulfonamides, sulfonate esters, and thiosulfonates. Such transformations highlight the value of developing useful chemicals out of toxic gas (SO_2_). We envision this method's utility in medicinal chemistry for the facile synthesis of a variety of organosulfur compounds.

## Experimental Section

### General considerations

All chemicals were purchased and used as received. The ^1^H and ^13^C{^1^H} NMR spectra were recorded on 400 and 500 MHz spectrometers in CDCl_3_ with residual undeuterated solvent (CDCl_3_, 7.26/77.0) as an internal standard. Chemical shifts (*δ*) are given in ppm, and *J* values are given in Hz. All chemical shifts are reported in ppm using tetramethylsilane as a reference. Chemical shifts (*δ*) downfield from the reference standard were assigned positive values. Column chromatography, including thin-layer chromatography (TLC), was performed over silica gel (Merck silica gel 100–200 mesh). Evaporation of solvents was performed under reduced pressure using a rotary evaporator. High-resolution mass spectrometry (HRMS) was performed on a Bruker maXis impact. All the glassware and NMR tubes used for the experiments were kept in an oven at 120 °C overnight (12 h).

### General procedure for the SO_2_ functionalization of amines

Amines 1a–1af (0.48 mmol, 1.0 equiv.), 2 (0.72 mmol, 1.5 equiv.), PLY (O,O) (5 mol%, 0.024 mmol) and Na_2_CO_3_ (0.96 mmol, 2 equiv.) were taken in an oven-dry 25 mL high-pressure *J*-Young tube with a Teflon cap equipped with a stir bar. Subsequently, 1.5 mL MeCN was added to the reaction mixture, and the tube was closed properly. In the Schlenk line, a freeze–pump–thaw cycle was applied twice to maintain an inert atmosphere. Next, SO_2_ was purged into the reaction mixture. The reaction tube was closed properly and placed under blue LED irradiation at 456 nm for 12 h. After completion of the reaction, the product was extracted in 25 mL ethyl acetate and dried over anhydrous sodium sulfate. The solvent was removed under reduced pressure, and the crude product was purified by column chromatography on silica gel (100–200 mesh) using a hexane/EtOAc mixture to obtain the pure desired products, which were characterized by NMR spectroscopy.

### General procedure for the SO_2_ functionalization of alcohols

Alcohols 4a–4p (0.48 mmol, 1.0 equiv.), 2a (0.72 mmol, 1.5 equiv.), PLY (O,O) (5 mol%, 0.024 mmol) and Na_2_CO_3_ (0.96 mmol, 2 equiv.) were taken in an oven-dried 25 mL high-pressure *J*-Young tube with a Teflon cap equipped with a stir bar. Subsequently, 1.5 mL MeCN was added to the reaction mixture, and the tube was closed. In the Schlenk line, a freeze–pump–thaw cycle was applied twice to maintain an inert atmosphere. Next, SO_2_ was purged into the reaction mixture. The reaction tube was closed properly and placed under blue LED irradiation at 456 nm for 12 h. After completion of the reaction, the product was extracted in 25 mL ethyl acetate and dried over anhydrous sodium sulfate. The solvent was removed under reduced pressure, and the crude product was purified by column chromatography on silica gel (100–200 mesh) using a hexane/EtOAc mixture to obtain the pure desired products, which were characterized by NMR spectroscopy.

### General procedure for the SO_2_ functionalization of thiols

Thiols 6a–6l (0.48 mmol, 1.0 equiv.), 2 (0.72 mmol, 1.5 equiv.), PLY (O,O) (5 mol%, 0.024 mmol) and Na_2_CO_3_ (0.96 mmol, 2 equiv.) were taken in an oven-dried 25 mL high-pressure *J*-Young tube with a Teflon cap equipped with a stir bar. Subsequently, 1.5 mL MeCN was added to the reaction mixture, and the tube was closed properly. In the Schlenk line, a freeze–pump–thaw cycle was applied twice to maintain an inert atmosphere. Next, SO_2_ was purged into the reaction mixture. The reaction tube was closed properly and placed under blue LED irradiation at 456 nm for 12 h. After completion of the reaction, the product was extracted in 25 mL ethyl acetate and dried over anhydrous sodium sulfate. The solvent was removed under reduced pressure, and the crude product was purified by column chromatography on silica gel (100–200 mesh) using a hexane/EtOAc mixture to obtain the pure desired products, which were characterized by NMR spectroscopy.

## Data availability

All experimental procedures, characterization details, and copies of NMR spectra for all compounds related to this article have been uploaded as part of the ESI.† CCDC 2387416 (for 3e), 2380420 (for 3k), 2380421 (for 3l), 2380422 (for 3o), 2380423 (for 3y), 2380424 (for 3z), 2387417 (for 3ac), 2380425 (for 3ad), 2380426 (for 3ag), 2387418 (for 5h), and 2387419 (for 5l) contain the supplementary crystallographic data for this paper. These data can be obtained free of charge *via*https://www.ccdc.cam.ac.uk/structures/, or by emailingda-ta_request@ccdc.cam.ac.uk, or by contacting The Cambridge Crystallographic Data Centre, 12 Union Road, Cambridge CB2 1EZ, UK; fax: +44 1223 336033.

## Author contributions

S. K. M. and P. D. conceived the idea of this work. P. D. carried out synthetic and catalytic experiments. P. B. was involved in catalytic experiments and control experiments. D. J. and P. P. D. carried out the experiments for starting material synthesis. S. M. contributed to crystallization and X-ray structure determination. S. K. M. supervised the overall work. The manuscript was written through the contributions of all authors. All authors have approved the final version of the manuscript.

## Conflicts of interest

The authors declare no conflict of interest.

## Supplementary Material

SC-OLF-D4SC08380F-s001

SC-OLF-D4SC08380F-s002
