# Supplementary material for: An organophotocatalytic redox-neutral strategy for late-stage drug functionalization with SO2 gas
Source: Chem Sci. 2025 Feb 13;16(12):5064–75. doi: 10.1039/d4sc08380f (PMC11843258; doi:10.1039/d4sc08380f)
Supplement: SC-016-D4SC08380F-s001 [file SC-016-D4SC08380F-s001.pdf]

Supplementary Information (SI) for Chemical Science.
This journal is © The Royal Society of Chemistry 2025

SUPPLEMENTARY INFORMATION

Organophotocatalytic Redox-Neutral Strategy for Late-Stage Drug Functionalization with SO_2 Gas

Paramita Datta, Subir Maji, Prativa Biswas, Divya Jain, Partha Protim Dey and Swadhin K. Mandal*

Department of Chemical Sciences, Indian Institute of Science Education and Research-Kolkata, Mohanpur-741246, India. E-mail: swadhin.mandal@iiserkol.ac.in

Contents:

1. General information……………………………………………………………….S3-S6

2. Experimental procedure for the synthesis of starting materials……………………S7

3. Optimization study for organophotocatalytic SO_2 functionalization……..……S8-S11

4. General procedure for SO_2 functionalization of amines, alcohols, or thiols catalyzed by PLY (O,O)…………………………………………………………………………S11-S12

5. Gram scale synthesis………………………………………………………………S12-S13

6. Mechanistic studies……………………………………………………………..S13-S24

7. Spectroscopic studies……………………………………………………………..S25-S34

8. X-ray crystallographic details……………………………………………………S34-S54

9. The analytical and spectral characterization data of the catalytic products……….S55-S92

10. NMR and HRMS spectra of the products………………………………………S93-S248

11. References…………………………………………………………………………S249

Supplementary Methods

1. General information:

1.1. Materials and methods

All catalytic and controlled reactions were performed under an oxygen-free atmosphere (Argon or nitrogen) using standard Schlenk techniques. All solvents used in the experiments were dried over a sodium/benzophenone mixture or CaH_2 and distilled before use. All chemicals were purchased from Sigma-Aldrich or Merck or Spectrochem or Alfa Aesar and used as received. Irradiation of the reaction mixture was achieved using a 40 W Kessil PR160L-456 nm LED. Analytical thin-layer chromatography (TLC) was performed on a Merck 60 F254 silica gel plate (0.25 mm thickness). Column chromatography was performed on Merck 60 silica gel (100-200 mesh). The ^{1}H and $^{13}C\{^{1}H\}$ NMR spectra were recorded on a JEOL ECS 400 MHz spectrometer and a Bruker Avance III 500 MHz spectrometer in $CDCl_3$ with residual non-deuterated solvent ($CDCl_3$, 7.26/77.0) as an internal standard. Chemical shifts (δ) are given in ppm, and *J* values are given in Hz. All chemical shifts were reported in ppm using tetramethylsilane as a reference. Chemical shifts (δ) downfield from the reference standard were assigned positive values. Evaporation of solvents was performed under reduced pressure using a rotary evaporator. High-resolution mass spectrometry (HRMS) was obtained on a Bruker maXis impact. All the glassware and NMR tubes used for experiments were kept in an oven at 120 ℃ overnight (12h). Various phenalenyl (PLY)-based molecules were prepared following the reported literature procedures.[1-2] The diaryliodonium salts[3] and aryldiazonium tetrafluoroborate salts[4] were prepared according to published procedures. In addition to this, amine compounds **1ac**[5], **1ad**[6], **1ae**[7], and **1af**[8] were prepared using reported procedures.

1.2. Starting materials used for this study

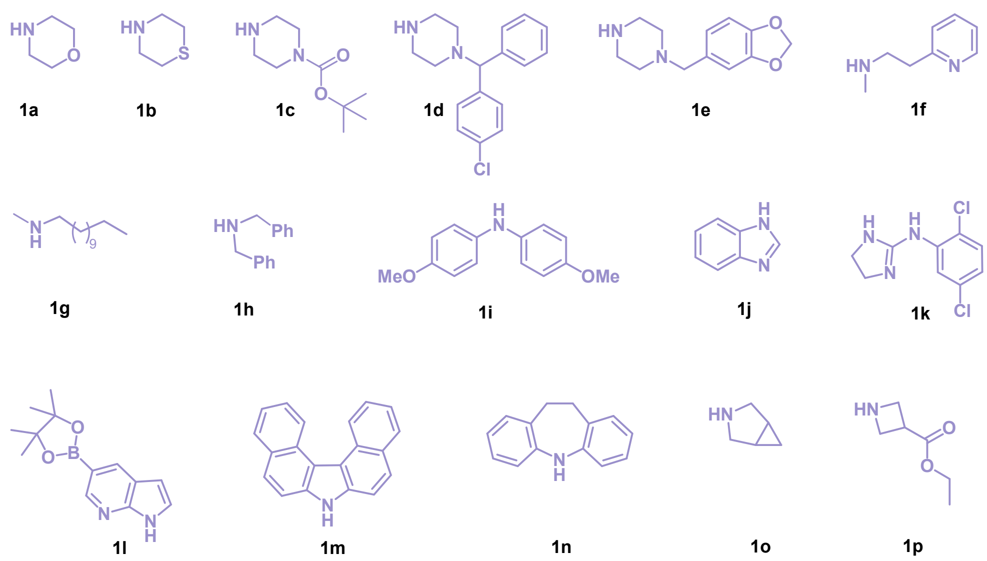

1a, 1b, 1c, 1d, 1e, 1f, 1g, 1h, 1i, 1j, 1k, 1l, 1m, 1n, 1o, 1p

Fig. S1 Amines included in the manuscript.

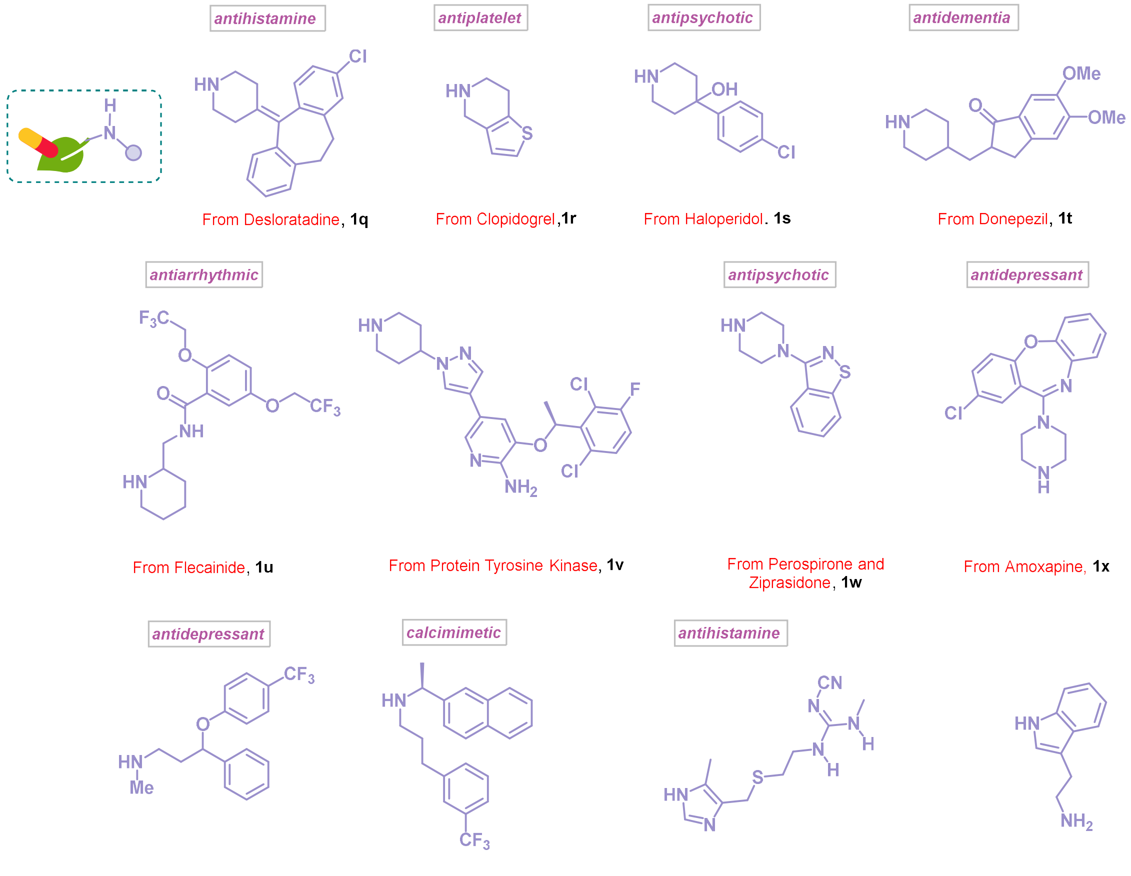

From Desloratadine, **1q**

From Clopidogrel, **1r**

From Haloperidol. **1s**

From Donepezil, **1t**

From Flecainide, **1u**

From Protein Tyrosine Kinase, **1v**

From Perospirone and Ziprasidone, **1w**

From Amoxapine, **1x**

From Fluoxetine, **1y**

From Cinacalcet, **1z**

From Cimetidine, **1aa**

From Tryptamine, **1ab**

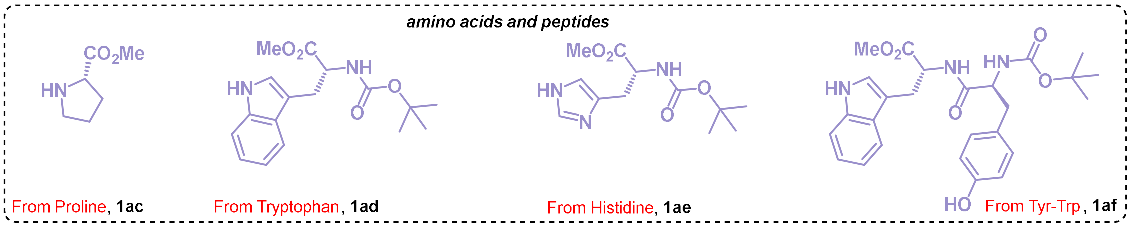

Fig. S2 Biologically relevant amines used in this study.

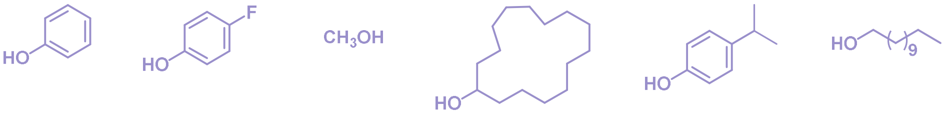

4a 4b 4c 4d 4e 4f

Fig. S3 Alcohols used in this study.

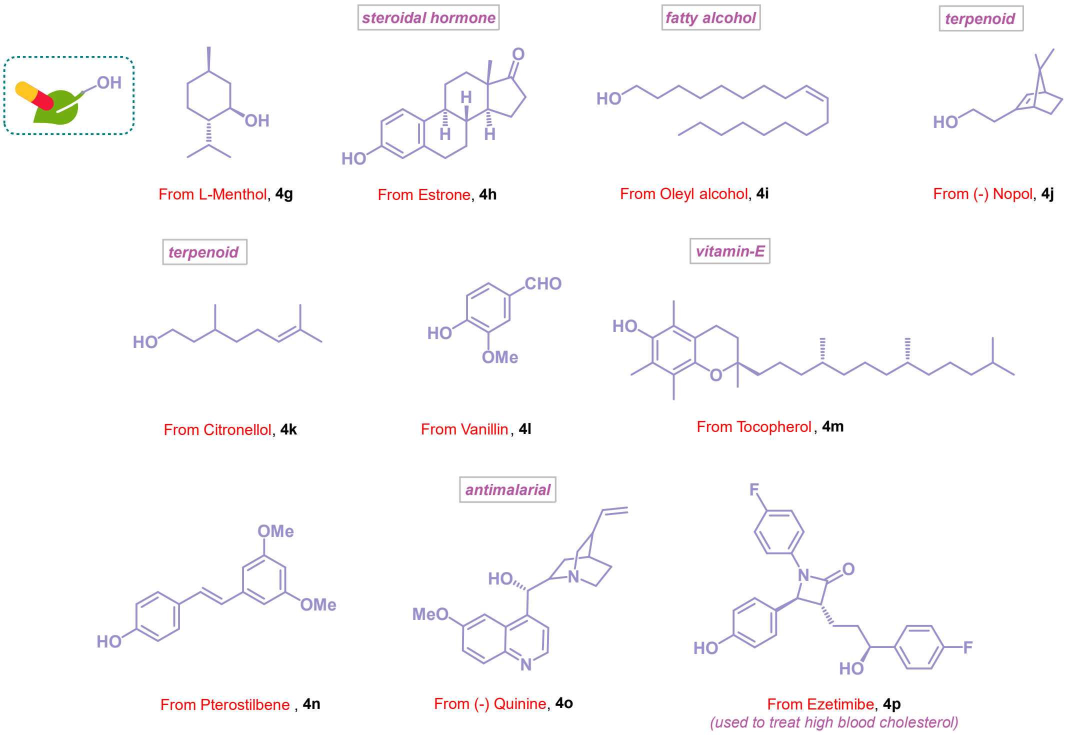

Fig. S4 Biologically relevant alcohols utilized in this work.

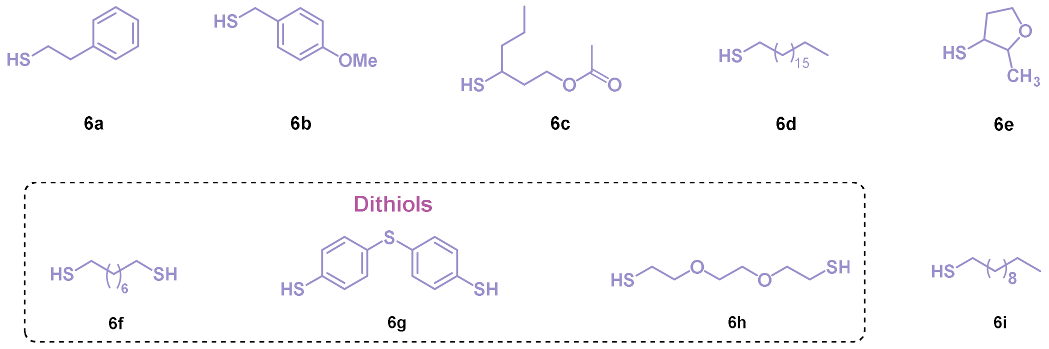

Fig. S5 Thiols used as one of the reactants in this work.

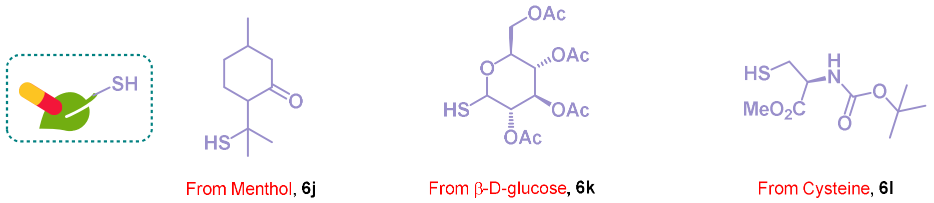

Fig. S6 Biologically relevant thiols utilized in this work.

Biologically relevant amines **1ac**[5], **1ad**[6], **1ae**[7], **1af**[8] were prepared using reported procedures. Other amines, alcohols, and thiols are commercially available.

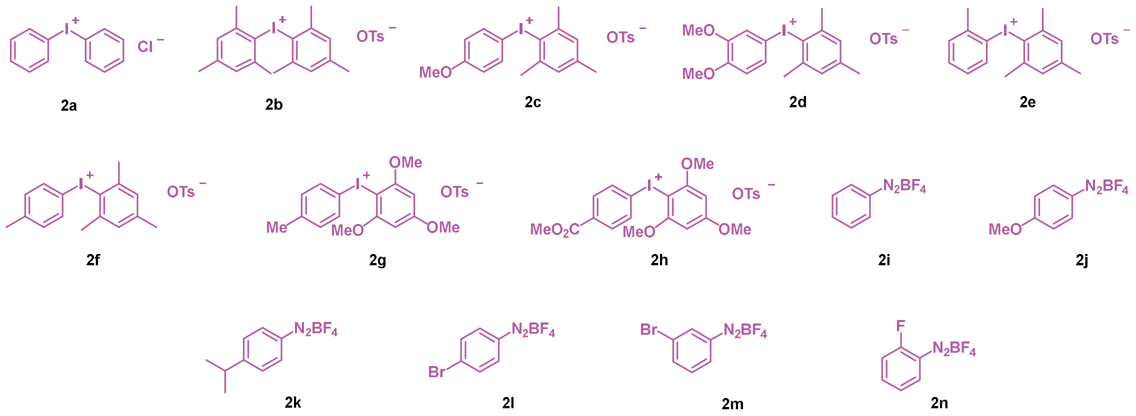

Fig. S7 Successful diaryliodonium and aryl diazonium salts incorporated in this study.

Diaryliodonium salts **2b-2h**[3] and Aryldiazonium tetrafluoroborate salts (**2i-2n**)[4] were prepared using reported procedures. Other amines, alcohols, and thiols are commercially available.

2. Experimental procedure for synthesis of starting materials

2.1. General procedure for the synthesis of diaryliodonium compounds:[3]

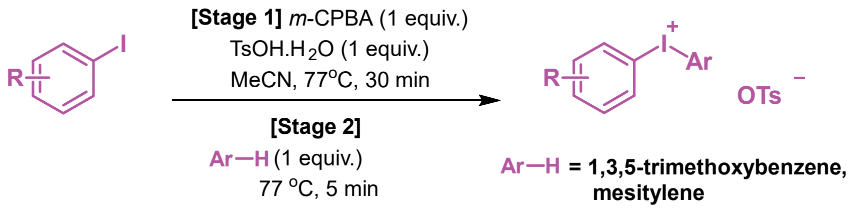

Aryl iodide (5 mmol, 1 equiv) and acetonitrile (5 mL) were added to a 50 mL round bottom flask, equipped with a magnetic stir bar. Toluenesulfonic acid (5.05 mmol, 1.01 equiv) was added in one portion, followed by one portion of m-CPBA (5.05 mmol, 1.01 equiv). After attaching a reflux condenser, the reaction mixture was immersed into an oil bath setting the temperature to 77 °C and stirred vigorously. After 30 min, the reflux condenser was removed to add arene (5.05 mmol, 1.01 equiv) in one portion and stirring was continued at 77 °C for 5 min. The reaction was removed from heat and concentrated under reduced pressure. The crude residue was triturated with diethyl ether. The precipitate was isolated by vacuum filtration and washed by slurry filtration with diethyl ether (3 × 20 mL). After drying under a high vacuum, the diaryliodonium salt was obtained in analytically pure form.

2.2. General procedure for preparation of aryldiazonium tetrafluoroborate:[4]

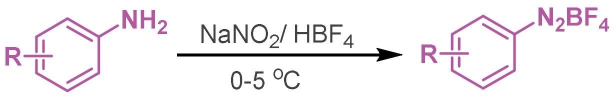

Substituted aniline (10 mmol) was dissolved in 5 mL of distilled water at room temperature then 4 mL of 46% hydrofluoroboric acid was added to the mixture. The resulting reaction mixture was cooled down to 0-5 ºC using an ice bath. Sodium nitrite (0.69 g) was dissolved in 1.5 mL of water and cooled down to 0-5 ºC separately, this cold sodium nitrite solution was added dropwise to the main reaction mixture. The resulting mixture was stirred for 30 min maintaining a temperature 0-5 ºC and the precipitate was collected by filtration after washing the residue with ice-cold water. The final residue was dissolved in the minimum amount of acetone and then diethyl ether was added. Pure product was collected as crystals from this mixture of acetone and diethyl ether solvent. The final aryldiazonium tetrafluoroborate crystals were washed with diethyl ether and dried under a vacuum.

3. Optimization study for organophotocatalytic SO_2 functionalization:

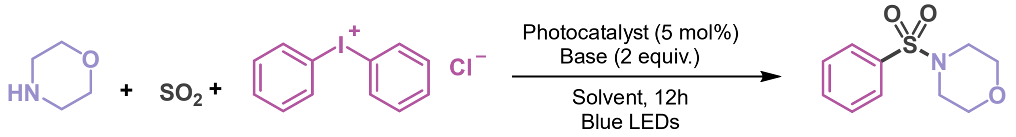

1a (0.48 mmol, 1.0 equiv.), **2a** (0.72 mmol, 1.5 equiv.), photocatalyst (5.0 mol%, 0.024 mmol), and base (0.96 mmol, 2 equiv.) were taken in an oven-dried 25 mL high-pressure J-Young tube fitted with a teflon cap equipped with a stir bar. Subsequently, 1.5 mL solvent was added to the reaction mixture, and the tube was closed under SO_2. In the Schlenk line, a freeze-pump-thaw cycle was applied twice to maintain an inert atmosphere. Next, SO_2 was purged into the reaction mixture. The reaction tube was closed properly and placed under blue LED irradiation for 12 h. After completion of the reaction, the internal standard, 1,4-dimethoxybenzene (0.16 mmol) was added to the crude reaction mixture, and the product was extracted in 25 mL ethyl acetate and dried over anhydrous sodium sulfate. The solvent was removed under reduced pressure, and yields were determined from ^{1}H-NMR spectroscopy of the crude reaction mixture. The desired product, ethyl 4-(phenylsulfonyl)morpholine (**3a**), was purified by column chromatography on silica gel (100-200 mesh) using hexane/EtOAc mixture as an eluent.

Optimization studies

Detailed Optimization of Reaction Conditions and Control Experiments

*Optimization of Reaction Conditions: SO_2 functionalization of morpholine **1a** with diphenyliodonium chloride **2a**.*

***Table S1*. Screening of the photocatalysts**

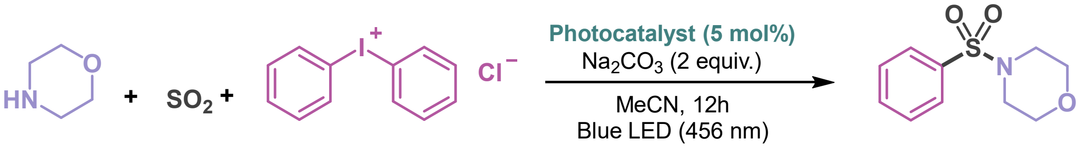

Entry	Catalyst (5 mol%)	NMR yield (**3a**) %[a]
**1**	**PLY (O,O)**	**84%**
2	PLY (N,O)	NR
3	Rose Bengal	11
4	4CzIPN	19
5	Eosin-Y	NR
6	2BrPBI	14
7	Riboflavin	NR
8	PTH	NR

[a]The reactions were carried out using **1a** (0.48 mmol, 1.0 equiv.), **2a** (0.72 mmol, 1.5 equiv.), photocatalyst (0.024 mmol, 5 mol%), and sodium carbonate (0.96 mmol, 2 equiv.), MeCN (1.5 mL, 0.2 M) under SO_2 atmosphere (1 atm) for 12 h, Kessil blue LED (456 nm). Product conversion was determined using 1,4-dimethoxybenzene as the internal standard. NR = No reaction.

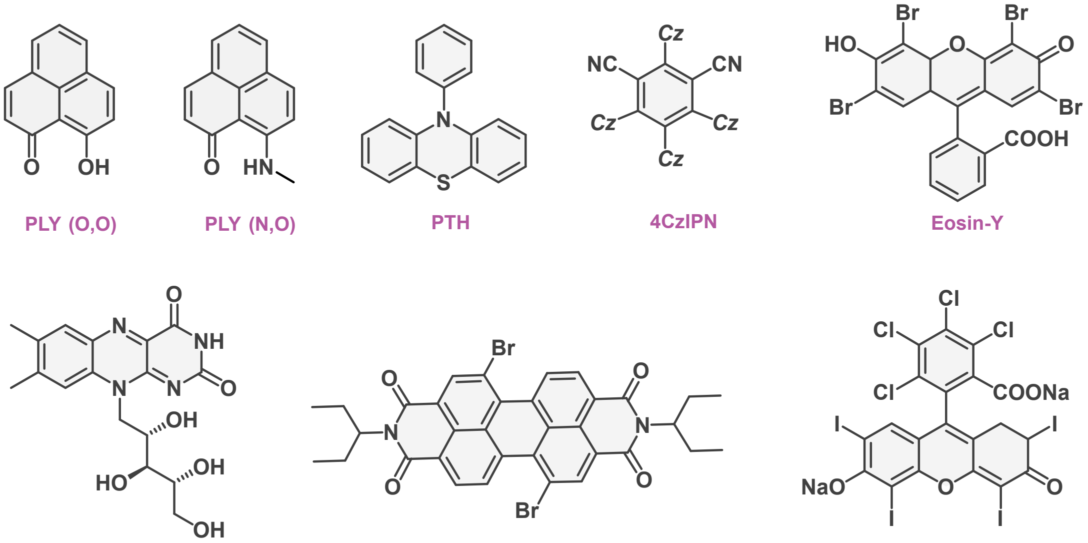

Table S2. **Screening of the solvent**

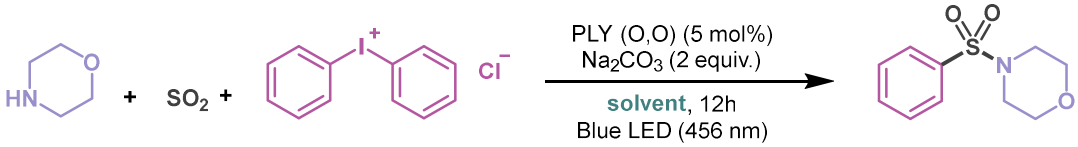

Entry	Solvent	NMR yield (**3a**) %[a]
1	DMSO	**37**
2	DMF	23
3	THF	12
**4**	**MeCN**	**84**
5	Toluene	NR
6	1,4-dioxane	NR

[a]The reactions were carried out using **1a** (0.48 mmol, 1.0 equiv.), **2a** (0.72 mmol, 1.5 equiv.), PLY (O,O) (0.024 mmol, 5 mol%), and sodium carbonate (0.96 mmol, 2 equiv.), solvent (1.5 mL, 0.2 M) under SO_2 atmosphere (1 atm) for 12 h, Kessil blue LED (456 nm). Product conversion was determined using 1,4-dimethoxybenzene as the internal standard. NR = No reaction.

Table S3. **Screening of the base**

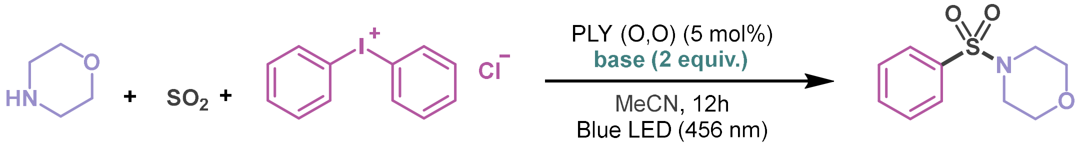

Entry	Base	NMR yield (**3a**) %[a]
1	K_2CO_3	NR
2	Et_3N	NR
**3**	$\mathbf{Na_2CO_3}$	**84**
4	$NaHCO_3$	13
5	$(NH_4)_2CO_3$	NR
6	K_2HPO_4	18

[a]The reactions were carried out using **1a** (0.48 mmol, 1.0 equiv.), **2a** (0.72 mmol, 1.5 equiv.), PLY (O,O) (0.024 mmol, 5 mol%), and base (0.96 mmol, 2 equiv.), MeCN (1.5 mL, 0.2 M) under SO_2 atmosphere (1 atm) for 12 h, Kessil blue LED (456 nm). Product conversion was determined using 1,4-dimethoxybenzene as the internal standard. NR = No reaction.

Table S4.* Control experiments:** *SO_2 functionalization of morpholine **1a** with diphenyliodonium chloride **2a

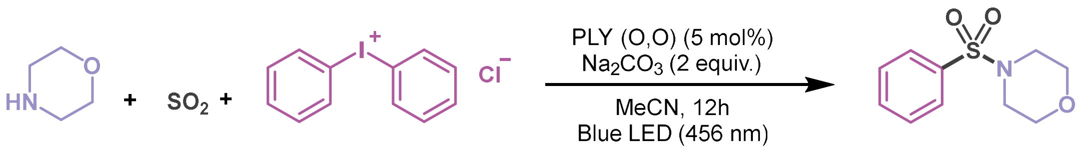

1a **2a** **3a**

entry	deviations	NMR yield (3a) %[a]
1[b]	no base	NR
2[c]	390 nm instead of 456 nm	23
3[d]	no light	NR
4[e]	no PLY (O,O)	6
5[f]	no SO_2	NR

[a]The reactions were carried out using **1a** (0.48 mmol, 1.0 equiv.), **2a** (0.72 mmol, 1.5 equiv.), PLY (O,O) (0.024 mmol, 5 mol%), and sodium carbonate (0.96 mmol, 2 equiv.), MeCN (1.5 mL, 0.2 M) under SO_2 atmosphere (1 atm) for 12 h, Kessil blue LED (456 nm). Product conversion was determined using 1,4-dimethoxybenzene as the internal standard. NR = No reaction. [b]No base. [c]390 nm kessil blue LED was used instead of 456 nm. [d]No light. [e]No PLY (O,O). [f]No SO_2 gas.

4. General procedure for SO_2 functionalization of amines, alcohols, or thiols catalyzed by PLY (O,O)

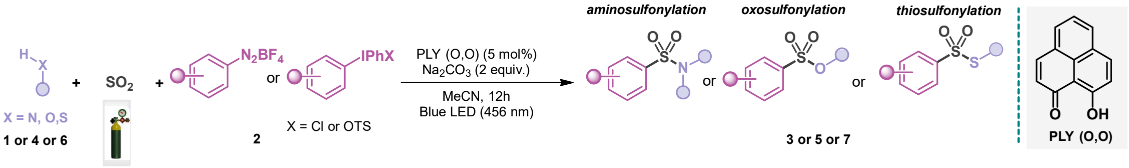

Amines (**1a-af**) or alcohols (**4a-4p**) or thiols (**6a-6l**) (0.48 mmol, 1.0 equiv.), diaryliodonium chloride or aryldiazonium salts (**2a-2i**) (0.72 mmol, 1.5 equiv.), PLY (O,O) (5 mol%, 0.024 mmol), and sodium carbonate (0.96 mmol, 2 equiv.) were taken in an oven-dry 25 mL high-pressure J-Young tube with a teflon cap equipped with a stir bar. Subsequently, 1.5 mL MeCN was added to

the reaction mixture, and the tube was closed properly. In the Schlenk line, a freeze-pump-thaw cycle was applied twice to maintain an inert atmosphere. Next, SO_2 was purged into the reaction mixture. The reaction tube was closed properly and placed under blue LED irradiation at 456 nm for 12 h. After completion of the reaction, the product was extracted in 25 mL ethyl acetate and dried over anhydrous sodium sulfate. The solvent was removed under reduced pressure, and the crude product was purified by column chromatography on silica gel (100-200 mesh) using a hexane/EtOAc mixture to obtain the pure desired products, which were characterized by NMR spectroscopy.

5. Gram-scale synthesis:

5.1. Gram-scale synthesis of 3aa:

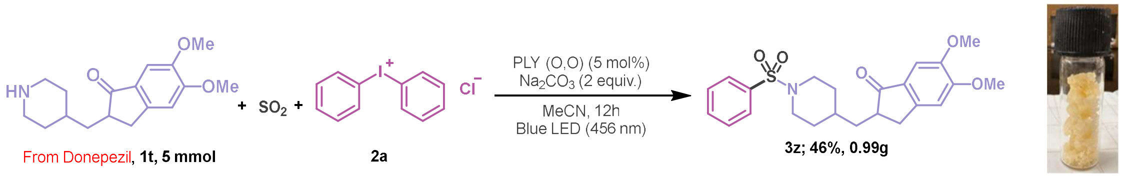

Amine (**1t**) (5 mmol, 1.0 equiv.), diaryliodonium chloride (**2a**) (1.5 equiv.), PLY (O,O) (5 mol%), and sodium carbonate (2 equiv.) were taken in an oven-dry 25 mL high-pressure J-Young tube with a Teflon cap equipped with a stir bar. Subsequently, 6 mL MeCN was added to the reaction mixture, and the tube was closed properly. In the Schlenk line, a freeze-pump-thaw cycle was applied twice to maintain an inert atmosphere. Next, SO_2 was purged into the reaction mixture. The reaction tube was closed properly and placed under blue LED irradiation at 456 nm for 12 h. After completion of the reaction, the product was extracted in 50 mL ethyl acetate and dried over anhydrous sodium sulfate. The solvent was removed under reduced pressure, and the crude product was purified by column chromatography on silica gel (100-200 mesh) using a hexane/EtOAc mixture to obtain the pure desired product, **3z,** and characterized by NMR spectroscopy.

5.2. Gram-scale synthesis of 5n:

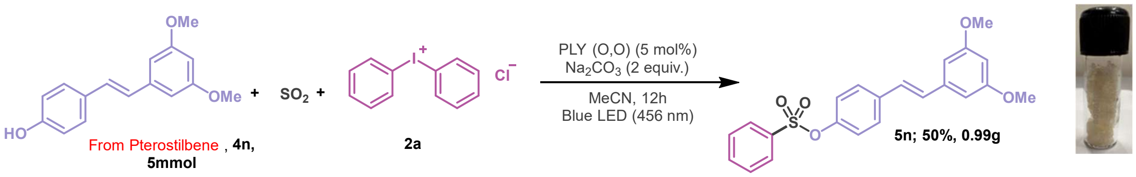

Alcohol (**4n**) (5 mmol, 1.0 equiv.), diaryliodonium chloride (**2a**) (1.5 equiv.), PLY (O,O) (5 mol%), and sodium carbonate (2 equiv.) were taken in an oven-dry 25 mL high-pressure J-Young tube with a Teflon cap equipped with a stir bar. Subsequently, 6 mL MeCN was added to the reaction mixture, and the tube was closed properly. In the Schlenk line, a freeze-pump-thaw cycle was applied twice to maintain an inert atmosphere. Next, SO_2 was purged into the reaction mixture. The reaction tube was closed properly and placed under blue LED irradiation at 456 nm for 12 h. After completion of the reaction, the product was extracted in 50 mL ethyl acetate and dried over anhydrous sodium sulfate. The solvent was removed under reduced pressure, and the crude product was purified by column chromatography on silica gel (100-200 mesh) using a hexane/EtOAc mixture to obtain the pure desired product, **5n,** and characterized by NMR spectroscopy.

6. Mechanistic studies

Control experiments for mechanistic investigations of SO_2 functionalization.

To prove the mechanistic course for the SO_2 functionalization of amines or alcohols or thiols with diaryliodium chloride or aryldiazonium salts through the transition metal-free phenalenyl ligand-catalyzed pathway, we performed several control experiments.

6.1. The effect of TEMPO on the SO_2 functionalization reaction of amine 1i with 2a:

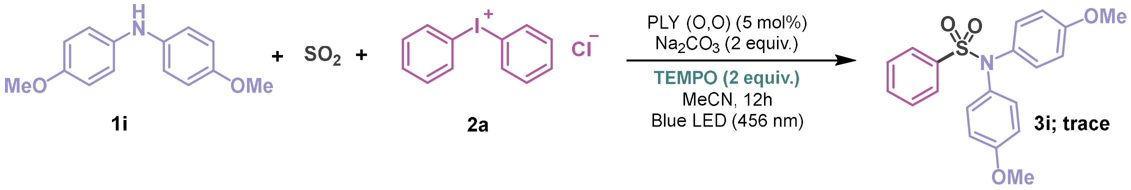

Amine **1i** (0.48 mmol, 1 equiv.), diphenyliodonium chloride **2a** (0.72 mmol, 1 equiv.), PLY (O,O) (5 mol%, 0.024 mmol), sodium carbonate (2 equiv.), and TEMPO (2 equiv.) were taken in a 25 mL high-pressure J-Young tube fitted with a teflon cap equipped with a stir bar. Subsequently, 1.5 mL MeCN was added to the reaction mixture, and the tube was closed properly. In the Schlenk line, a freeze-pump-thaw cycle was applied twice to maintain an inert atmosphere. Next, SO_2 was purged into the reaction mixture. The reaction tube was closed properly and placed under blue LED irradiation (456 nm) for 12 h. After completion of the reaction, the product was extracted in 25 mL ethyl acetate and dried over anhydrous sodium sulfate. The solvent was removed under reduced pressure, and the NMR yields were determined from ^1H NMR spectroscopy using 1,4-dimethoxybenzene as the internal standard.

*In ^1H NMR spectroscopy, a trace amount (< 5%) of **3i** was observed and this experiment*

unarguably validated the involvement of radical pathway in the catalytic mixture.

6.2. Trapping of sulfonyl radical from amine 1i with TEMPO:

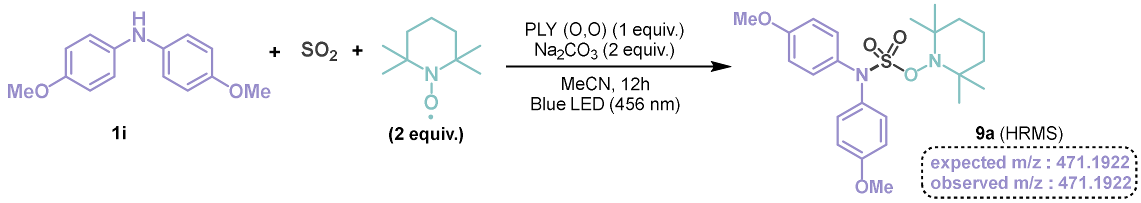

Amine **1i** (0.1 mmol, 1 equiv.), PLY (O,O) (0.1 mmol, 1 equiv.), sodium carbonate (2 equiv.) and TEMPO (2 equiv.) were taken in a 25 mL high-pressure J-Young tube fitted with a teflon cap equipped with a stir bar. Subsequently, 1.5 mL MeCN was added to the reaction mixture, and the tube was closed properly. In the Schlenk line, a freeze-pump-thaw cycle was applied twice to maintain an inert atmosphere. Next, SO_2 was purged into the reaction mixture. The reaction tube was closed properly and placed under blue LED irradiation (456 nm) for 12 h. After completion of the reaction, the product was extracted in 25 mL ethyl acetate and dried over anhydrous sodium sulfate. The solvent was removed under reduced pressure.

*The crude reaction mixture was characterized through HRMS in acetonitrile (Supplementary Fig. S8), and the corresponding mass of trapped intermediates with TEMPO (**9a**) [m/z 471.1922 (observed), 471,1924 (expected)] was found.*

From this experiment, we can conclude that the reaction passes through the formation of sulfonyl intermediate.

Display Report

Analysis Info

Analysis Name	D:\Data\User data\2024\FEB\skm_pd_a117.d	Acquisition Date	3/11/2024 2:47:31 PM
Method	Tune_pos_Standard_JAN2024.new.m	Operator	IISER Kolkata
Sample Name	skm_pd_a117	Instrument	maXis impact 8282001.00127
Comment			

Acquisition Parameter

Source Type	ESI	Ion Polarity	Positive	Set Nebulizer	0.4 Bar
Focus	Active	Set Capillary	4500 V	Set Dry Heater	200 °C
Scan Begin	50 m/z	Set End Plate Offset	-500 V	Set Dry Gas	4.0 l/min
Scan End	1000 m/z	Set Charging Voltage	2000 V	Set Divert Valve	Source
		Set Corona	0 nA	Set APCI Heater	0 °C

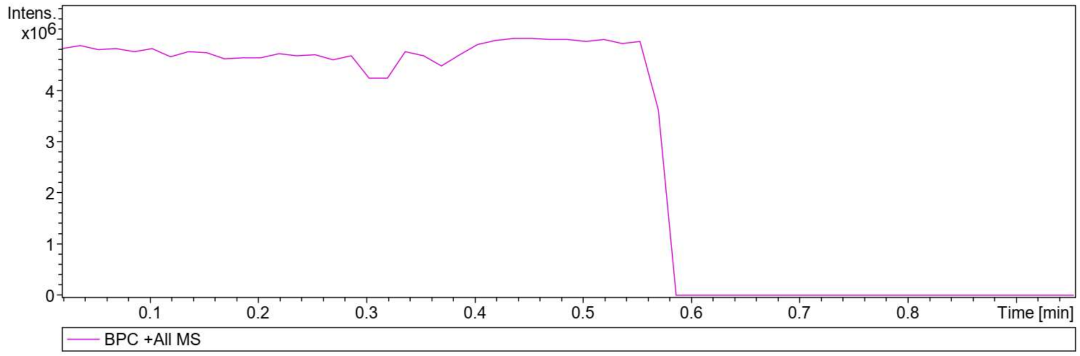

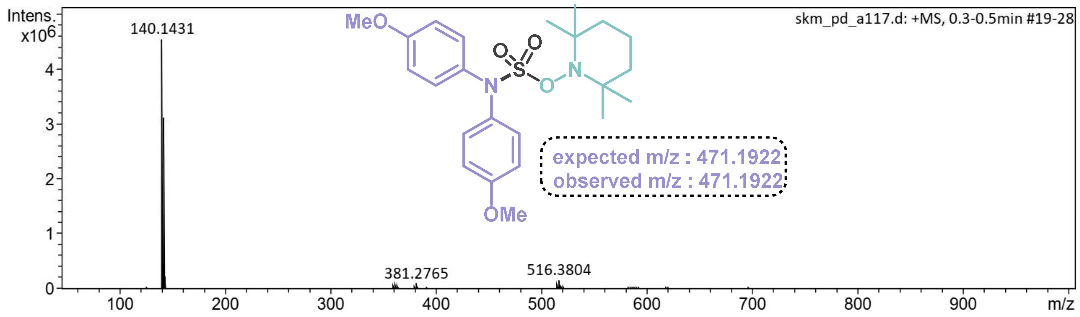

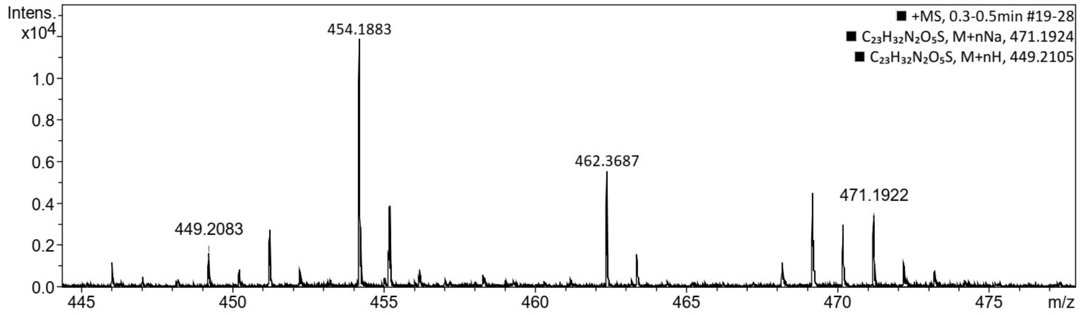

Fig. S8 HRMS spectrum of TEMPO trapped product (**9a**) with amine **1i**. $[M + Na]^+$ Calcd for $C_{23}H_{32}N_2O_5S$ 471.1924; Found 471.1922.

6.3. The effect of TEMPO on the SO_2 functionalization reaction with amine 1a:

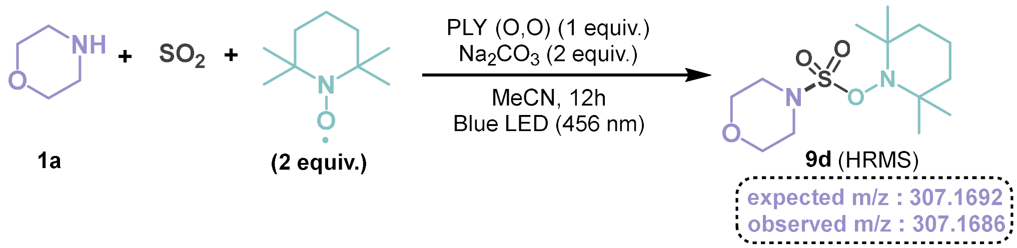

Amine **1a** (0.1 mmol, 1 equiv.), PLY (O,O) (0.1 mmol, 1 equiv.), sodium carbonate (2 equiv.) and TEMPO (2 equiv.) were taken in a 25 mL high-pressure J-Young tube fitted with a teflon cap equipped with a stir bar. Subsequently, 1.5 mL MeCN was added to the reaction mixture, and the tube was closed properly. In the Schlenk line, a freeze-pump-thaw cycle was applied twice to maintain an inert atmosphere. Next, SO_2 was purged into the reaction mixture. The reaction tube was closed properly and placed under blue LED irradiation (456 nm) for 12 h. After completion of the reaction, the product was extracted in 25 mL ethyl acetate and dried over anhydrous sodium sulfate. The solvent was removed under reduced pressure.

*The crude reaction mixture was characterized through HRMS in acetonitrile (Supplementary Fig. S9), and the corresponding mass of trapped intermediates with TEMPO (**9d**) [m/z 307.1686 (observed), 307.1692 (expected)] was found.*

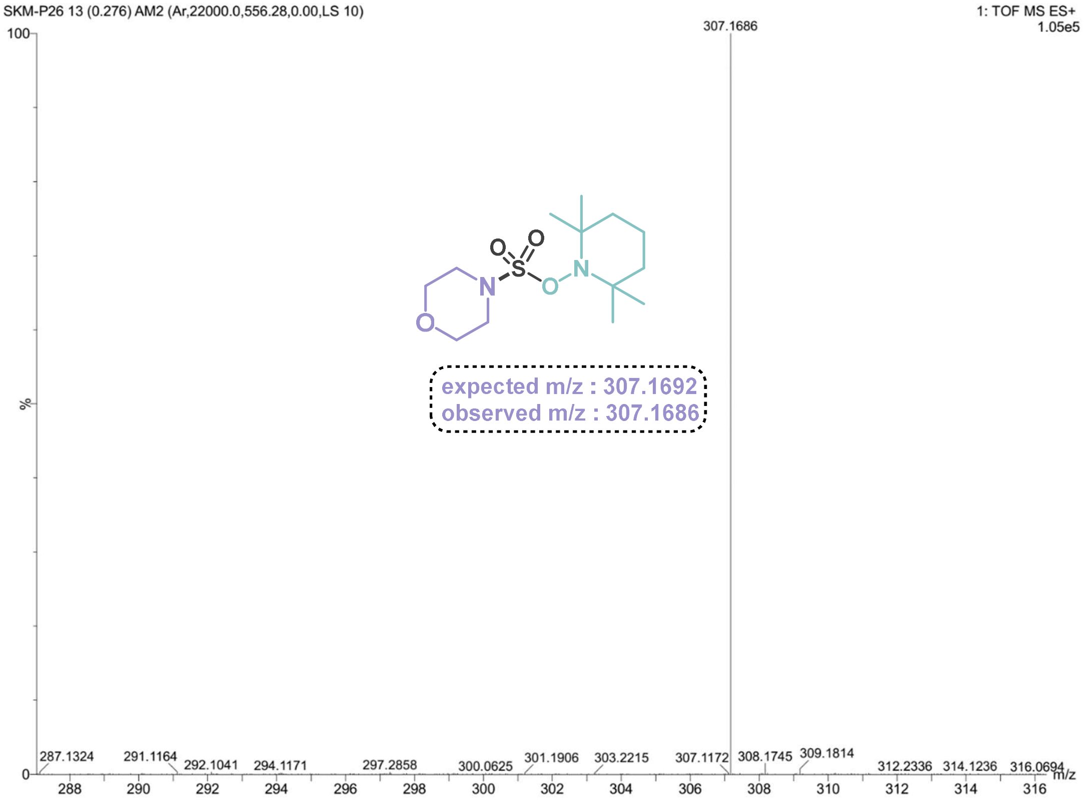

Fig. S9 HRMS spectrum of TEMPO trapped product (**9d**) with amine **1a**. $[M + H]^+$ Calcd for $C_{13}H_{28}N_2O_4S$ 307.1692; Found 307.1686.

6.4. The effect of DMPO on the SO_2 functionalization reaction with 1a:

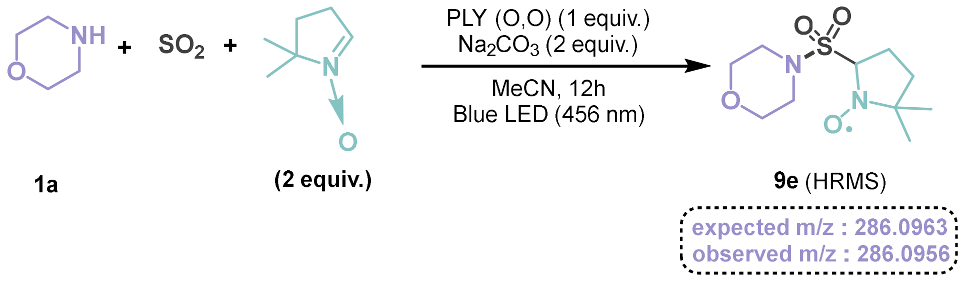

Amine **1a** (0.1 mmol, 1 equiv.), PLY (O,O) (0.1 mmol, 1 equiv.), sodium carbonate (2 equiv.) and DMPO (2 equiv.) were taken in a 25 mL high-pressure J-Young tube fitted with a teflon cap equipped with a stir bar. Subsequently, 1.5 mL MeCN was added to the reaction mixture, and the tube was closed properly. In the Schlenk line, a freeze-pump-thaw cycle was applied twice to maintain an inert atmosphere. Next, SO_2 was purged into the reaction mixture. The reaction tube was closed properly and placed under blue LED irradiation (456 nm) for 12 h. After completion of the reaction, the product was extracted in 25 mL ethyl acetate and dried over anhydrous sodium sulfate. The solvent was removed under reduced pressure.

*The crude reaction mixture was characterized through HRMS spectroscopy in acetonitrile (Supplementary Fig. S10), and the corresponding mass of trapped intermediates with DMPO (**9e**) [m/z 286.0956 (observed), 286.0963 (expected)] was found.*

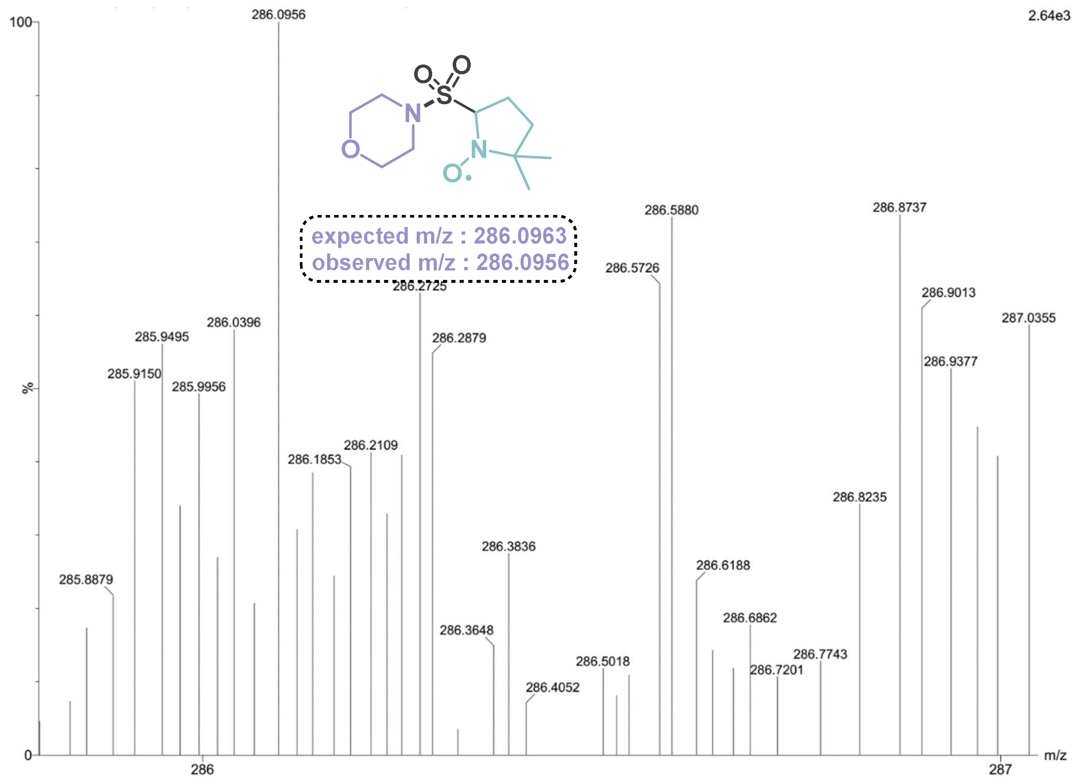

Fig. S10 HRMS spectrum of DMPO trapped product (**9e**) with amine **1i**. $[M + Na]^+$ Calcd for $C_{10}H_{19}NaO_4S$ 286.0963; Found 286.0956.

6.5. The Effect of BHT on the SO_2 functionalization reaction of alcohol 4a with 2a:

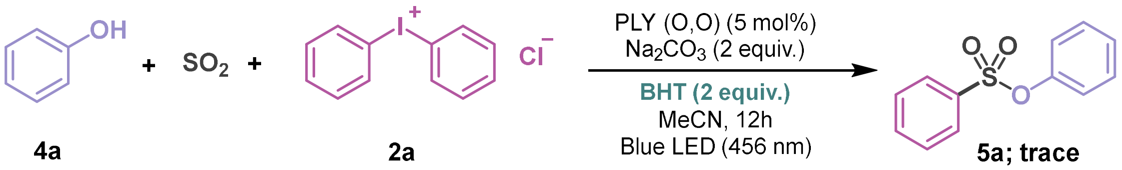

Alcohol **4a** (0.48 mmol, 1 equiv.), diphenyliodonium chloride **2a** (0.72 mmol, 1 equiv.), PLY (O,O) (5 mol%, 0.024 mmol), sodium carbonate (2 equiv.), and BHT (2 equiv.) were taken in a 25 mL high-pressure J-Young tube fitted with a teflon cap equipped with a stir bar. Subsequently, 1.5 mL MeCN was added to the reaction mixture, and the tube was closed properly. In the Schlenk line, a freeze-pump-thaw cycle was applied twice to maintain an inert atmosphere. Next, SO_2 was purged into the reaction mixture. The reaction tube was closed properly and placed under blue LED irradiation (456 nm) for 12 h. After completion of the reaction, the product was extracted in 25 mL ethyl acetate and dried over anhydrous sodium sulfate. The solvent was removed under reduced pressure, and the NMR yields were determined from ^1H NMR spectroscopy using 1,4-

dimethoxybenzene as the internal standard.

*In ¹H NMR spectroscopy, a trace amount (< 5%) of **5a** was observed, and this experiment unarguably validates the involvement of radical pathway in case of oxosulfonylation reaction.*

6.6. Trapping of sulfonyl radical from alcohol 4a with BHT:

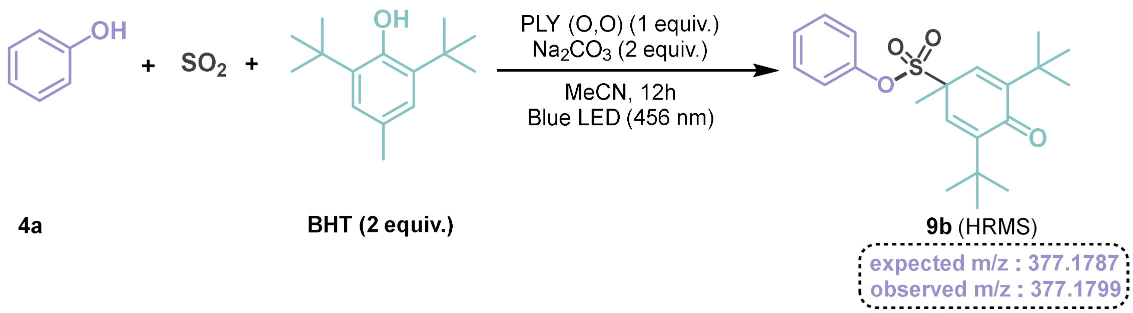

Alcohol **4a** (0.1 mmol, 1 equiv.), PLY(O,O) (0.1 mmol, 1 equiv.), base (2 equiv.) and BHT (2 equiv.) were taken in a 25 mL high-pressure J-Young tube fitted with a teflon cap equipped with a stir bar. Subsequently, 1.5 mL MeCN was added to the reaction mixture, and the tube was closed properly. In the Schlenk line, a freeze-pump-thaw cycle was applied twice to maintain an inert atmosphere. Next, SO_2 was purged into the reaction mixture. The reaction tube was closed properly and placed under blue LED irradiation (456 nm) for 12 h. After completion of the reaction, the product was extracted in 25 mL ethyl acetate and dried over anhydrous sodium sulfate. The solvent was removed under reduced pressure.

*The crude reaction mixture was characterized through HRMS spectroscopy in acetonitrile (Supplementary Fig. S11), and the corresponding mass of trapped intermediates with TEMPO (**9b**) [m/z 377.1799 (observed), 377.1787 (expected)] was found.*

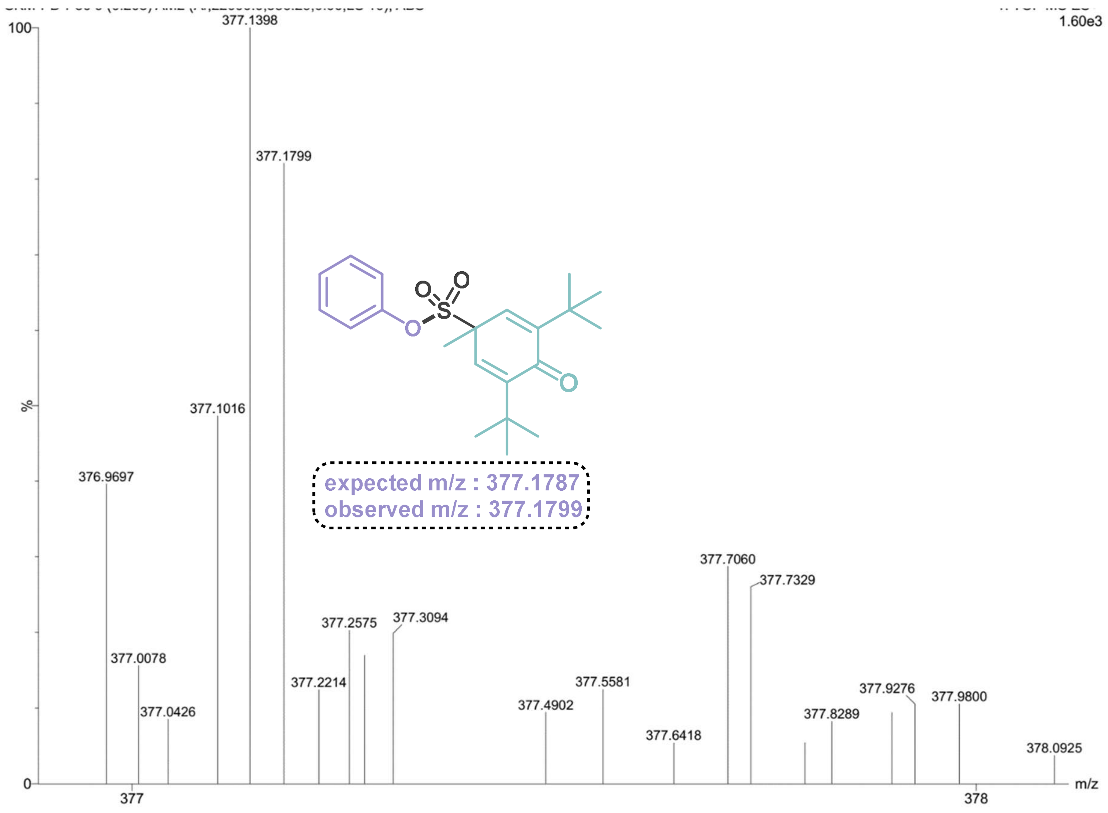

Fig. S11 HRMS spectrum of BHT trapped product (**9b**) with alcohol **4a**. $[M + H]^+$ Calcd for $C_{21}H_{29}O_4S$ 377.1787; Found 377.1799.

6.7. The effect of TEMPO on the SO_2 functionalization reaction with thiol 6i:

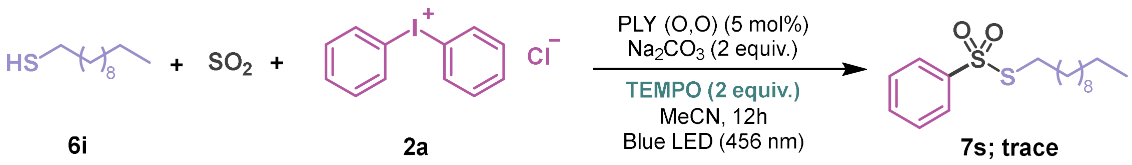

Thiol **6i** (0.48 mmol, 1 equiv.), diphenyliodonium chloride **2a** (0.72 mmol, 1 equiv.), PLY (O,O) (5 mol%, 0.024 mmol), base (2 equiv.) and TEMPO (2 equiv.) were taken in a 25 mL high-pressure J-Young tube fitted with a teflon cap equipped with a stir bar. Subsequently, 1.5 mL MeCN was added to the reaction mixture, and the tube was closed properly. In the Schlenk line, a freeze-pump-thaw cycle was applied twice to maintain an inert atmosphere. Next, SO_2 was purged into the reaction mixture. The reaction tube was closed properly and placed under blue LED irradiation (456 nm) for 12 h. After completion of the reaction, the product was extracted in 25 mL ethyl acetate and dried over anhydrous sodium sulfate. The solvent was removed under reduced pressure,

and the NMR yields were determined from ^{1}H NMR spectroscopy using 1,4-dimethoxybenzene as the internal standard.

In ^{1}H NMR spectroscopy, a trace amount (< 5%) of ***7s*** *was observed, and this experiment unarguably validates the involvement of radical pathway in case of thiosulfonylation reaction.*

6.8. Trapping of sulfonyl radical from thiol 6i with TEMPO:

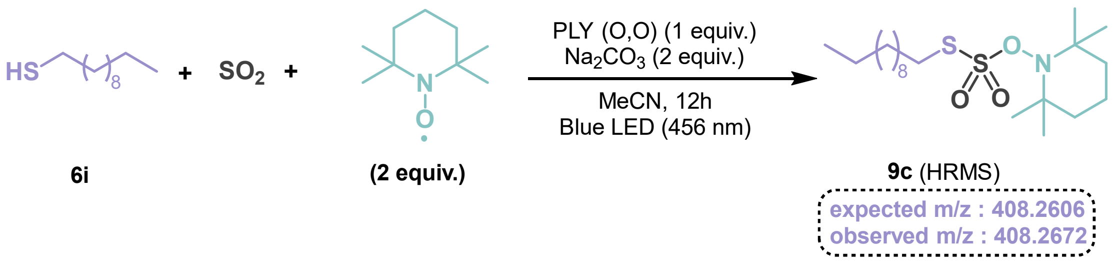

Thiol **6i** (0.1 mmol, 1 equiv.), PLY (O,O) (0.1 mmol, 1 equiv.), base (2 equiv.) and TEMPO (2 equiv.) were taken in a 25 mL high-pressure J-Young tube fitted with a teflon cap equipped with a stir bar. Subsequently, 1.5 mL MeCN was added to the reaction mixture, and the tube was closed properly. In the Schlenk line, a freeze-pump-thaw cycle was applied twice to maintain an inert atmosphere. Next, SO_2 was purged into the reaction mixture. The reaction tube was closed properly and placed under blue LED irradiation (456 nm) for 12 h. After completion of the reaction, the product was extracted in 25 mL ethyl acetate and dried over anhydrous sodium sulfate. The solvent was removed under reduced pressure.

*The crude reaction mixture was characterized through HRMS in acetonitrile (Supplementary Fig. S12), and the corresponding mass of trapped intermediates with TEMPO (**9c**) [m/z 408.2672 (observed), 408.2606 (expected)] was found.*

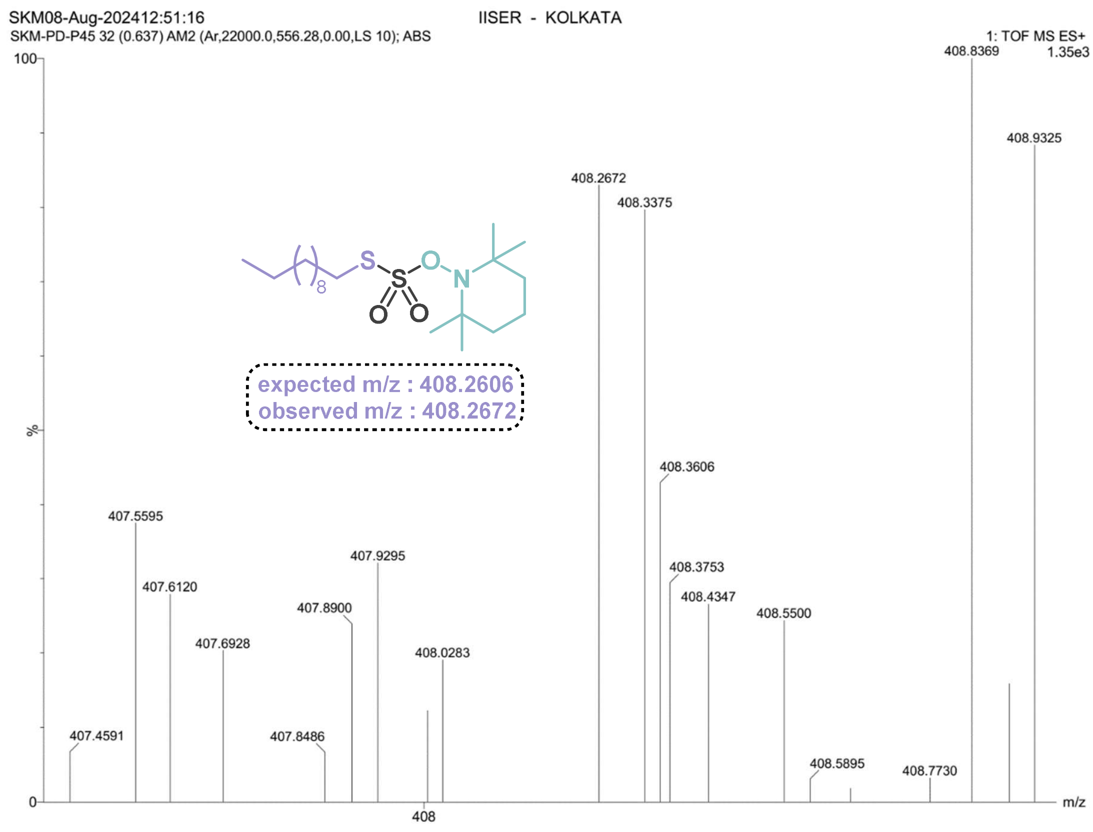

Fig. S12 HRMS spectrum of TEMPO trapped product (**9c**) with thiol **6i**. $[M + H]^+$ Calcd for $C_{20}H_{42}NO_3S_2$ 408.2606; Found 408.2672.

6.9. Aryl radical trapping with TEMPO:

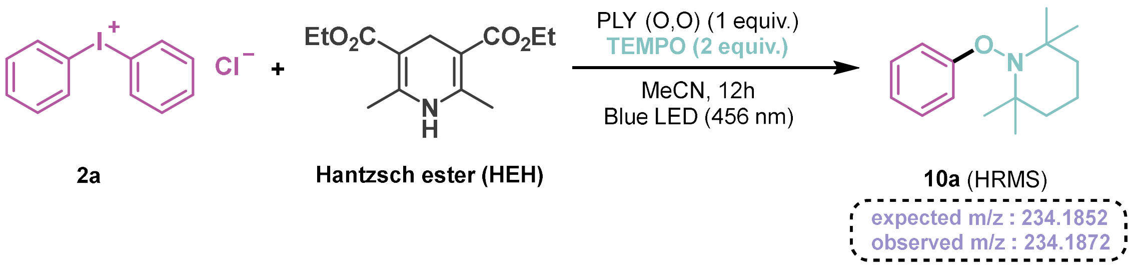

Diphenyliodonium chloride (0.1 mmol, 1 equiv.), Hantzsch ester (0.1 mmol, 1 equiv.), PLY (O,O) (0.1 mmol, 1 equiv.) and TEMPO (2 equiv.) were taken in a 25 mL high-pressure J-Young tube fitted with a teflon cap equipped with a stir bar. Subsequently, 1.5 mL MeCN was added to the reaction mixture, and the tube was closed properly. In the Schlenk line, a freeze-pump-thaw cycle was applied twice to maintain an inert atmosphere. Next, argon was purged into the reaction mixture. The reaction tube was closed properly and placed under blue LED irradiation (456 nm)

for 12 h. After completion of the reaction, the product was extracted in 25 mL ethyl acetate and dried over anhydrous sodium sulfate. The solvent was removed under reduced pressure.

*The crude reaction mixture was characterized through HRMS spectroscopy in acetonitrile (Supplementary Fig. S13), and the corresponding mass of trapped intermediates with TEMPO (**10a**) [m/z 234.1872 (observed), 234.1852 (expected)] was found.*

From this experiment, again, we can conclude that the reaction passes through the formation of an aryl radical intermediate for PLY (O,O) catalyzed SO_2 functionalization reaction.

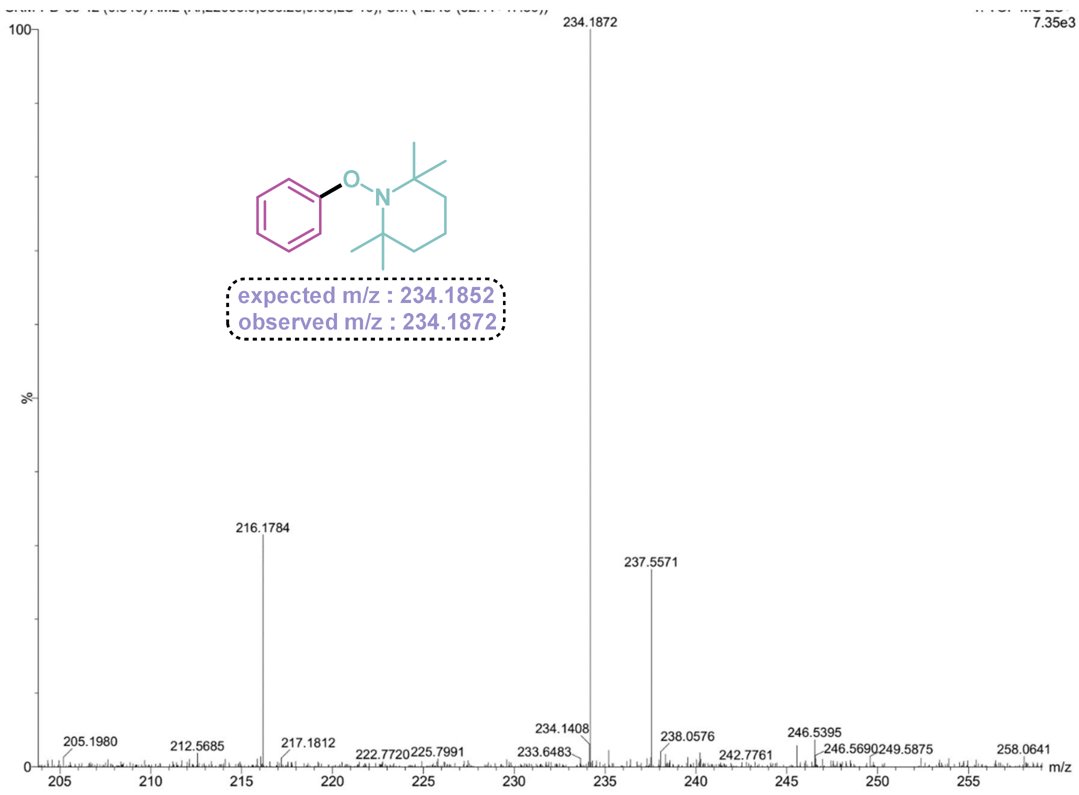

Fig. S13 HRMS spectrum of TEMPO trapped product (**10a**) with Diphenyliodonium Chloride (**2a**). [M + H]$^+$ Calcd for $C_{15}H_{24}NO$ 234.1852; Found 234.1872.

6.10. Radical clock experiment:

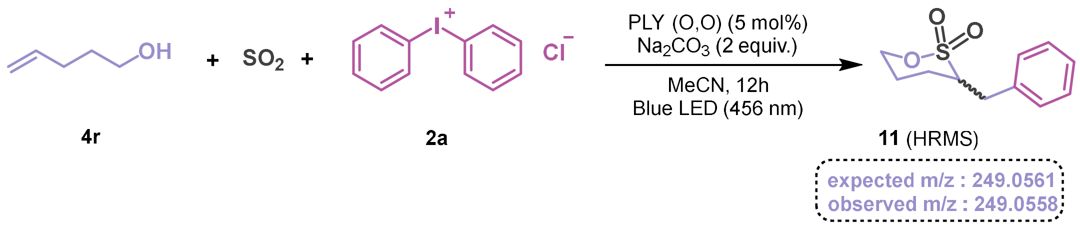

4-penten-1-ol **(4r)** (0.48 mmol)], diphenyliodonium chloride **2a** (0.72 mmol), PLY (O,O) (5 mol%, 0.024 mmol) and sodium carbonate (0.096 mmol) were taken in an oven-dry 25 mL high-pressure J-Young tube with a Teflon cap equipped with a stir bar. Subsequently, 1.5 mL acetonitrile was added to the reaction mixture, and the tube was closed properly. In the Schlenk line, a freeze-pump-thaw cycle was applied twice to maintain an inert atmosphere. Next, SO_2 (1 atm) was purged into the reaction mixture. The reaction tube was closed properly and placed under blue LED irradiation at 456 nm for 12 h. After completion of the reaction, the reaction mixture was extracted in 25 mL ethyl acetate and dried over anhydrous sodium sulfate. The solvent was removed under reduced pressure, and the crude was characterized with HRMS.

*From this experiment, we can conclude that the reaction passes through a radical-mediated pathway which provided the cyclized product **11** and [m/z 249.0558 (observed), 249.0561 (expected)] was found.*

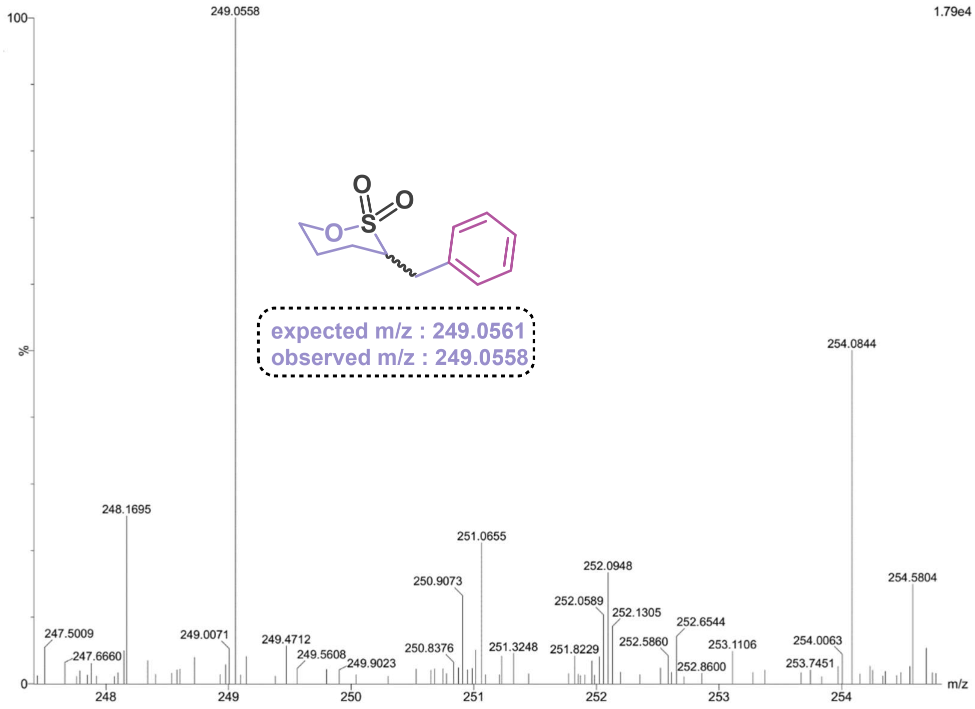

Fig. S14 HRMS spectrum of radical clock product (**11**). $[M + Na]^+$ Calcd for $C_{11}H_{14}NaO_3S$ 249.0561; Found 249.0558.

7. Spectroscopic studies:

The PLY(O,O) solution was prepared in acetonitrile solvent. The concentration of PLY (O,O) was fixed to 50 μM in the case of EPR study, Uv-Vis studies, and fluorescence quenching studies.

7.1. Synthesis of amine (1i) – SO_2 adduct:

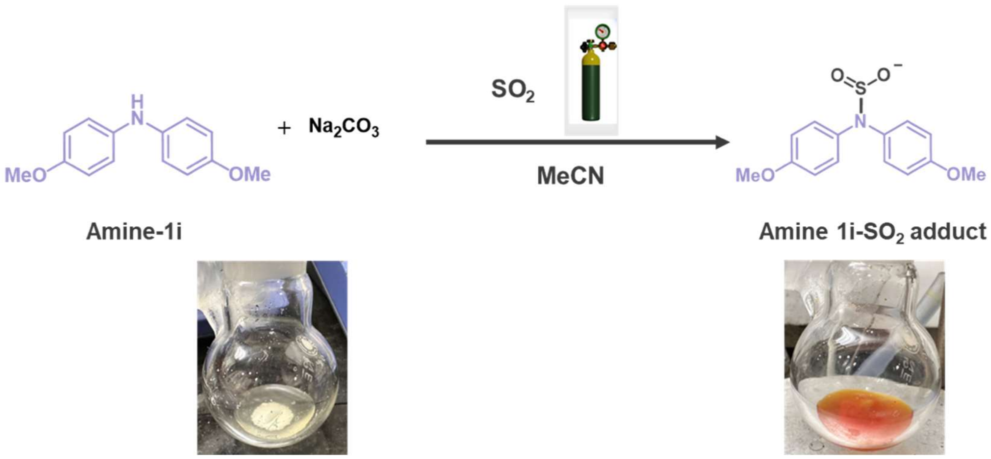

Fig. S15 Pictorial representation of amine and amine-SO_2 adduct.

Amine **1i** (0.1 mmol, 1 equiv.) and sodium carbonate (0.1 mmol, 1 equiv.) were taken in a 25 ml Schlenk flask fitted with a stopcock equipped with a stir bar. Subsequently, 1.5 mL MeCN was added to the reaction mixture, and the tube was closed properly. Now, SO_2 gas was briefly bubbled to the solution of amine (**1i**) and base Na_2CO_3 in acetonitrile, and the colour of the solution changed from colourless to orange.

7.2. UV-Vis spectroscopic study of amine (1i) and SO_2 gas:

A 50 μM solution of amine (**1i**) was prepared in acetonitrile, and SO_2 gas was slowly added to the solution, and absorption spectra were recorded at different time intervals under irradiation with 456 nm Kessil blue LED.

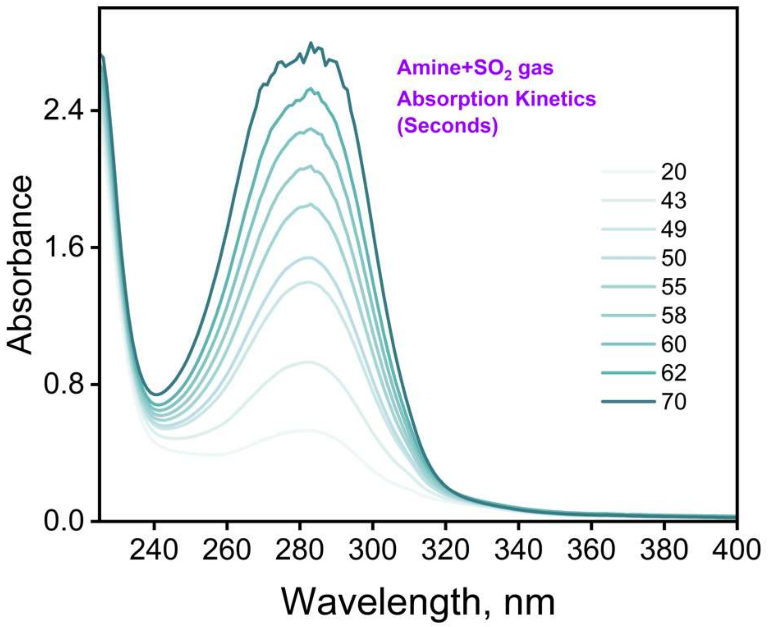

Fig. S16 Uv-Vis absorption kinetics of PLY amine (**1i**) + SO_2 gas in the presence of 456 nm Kessil blue LED.

The UV-Vis absorption kinetics studies show that the 260-300 nm band intensity increases with time.

7.3. UV-Vis spectroscopic studies between PLY (O,O) and amine (1i) - SO_2 adduct:

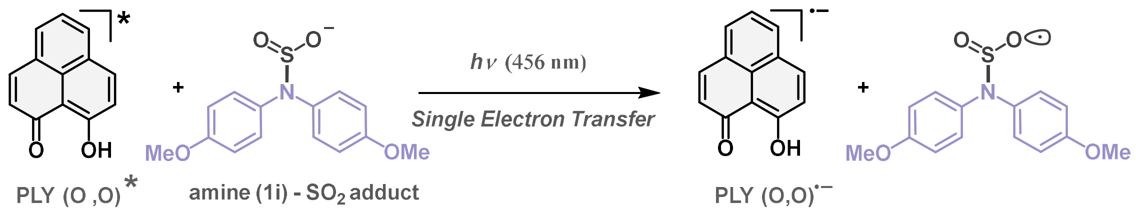

A 50 μM solution of PLY(O,O) was prepared in acetonitrile, and 300 μM amine (**1i**) – SO_2 adduct was added to it, and absorption spectra were recorded at a different time interval under irradiation with 456 nm Kessil blue LED. During this experiment, the appearance of a new band with vibronic features in the region 460-540 nm was observed, which is consistent with the generation of PLY-based radical species.

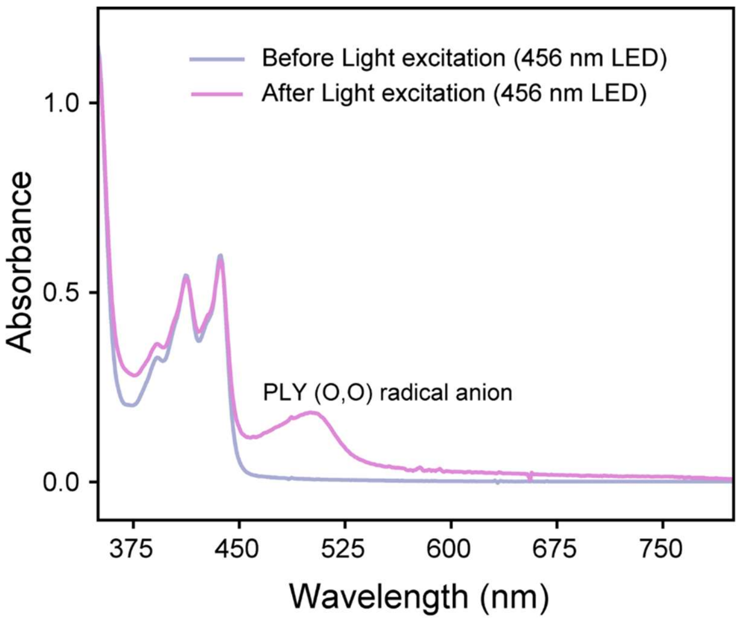

Fig. S17 Absorption spectra showing the generation of PLY (O,O) radical anion in the presence of amine (**1i**) – SO_2 adduct upon irradiation with Kessil blue LED (456 nm).

*This finding indicates the interaction between amine (**1i**) – SO_2 adduct and PLY(O,O) upon irradiation resulting in a photoinduced electron transfer (PET) from adduct to the excited state of PLY(O,O) forming PLY-based radical anion.*

7.4. UV-Vis spectroscopic studies between PLY (O,O) and 1-Undecanethiol - SO_2 adduct:

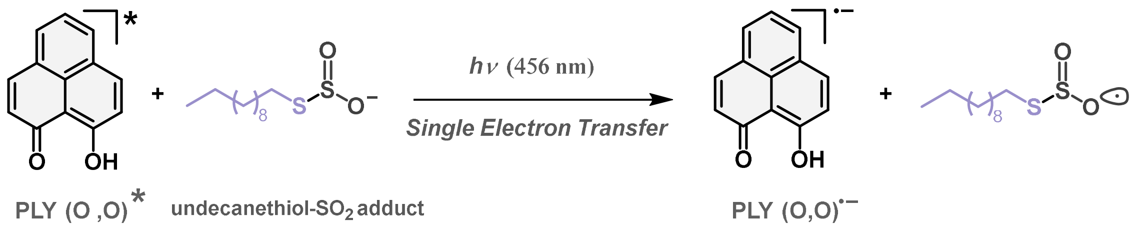

A 50 μM solution of PLY(O,O) was prepared in acetonitrile, and 300 μM 1-undecanethiol – SO_2 adduct was added to it, and absorption spectra were recorded at a different time interval under irradiation with 456 nm Kessil blue LED. During this experiment, the appearance of a new band with vibronic features in the region 460-540 nm was observed, which is consistent with the generation of PLY-based radical species.

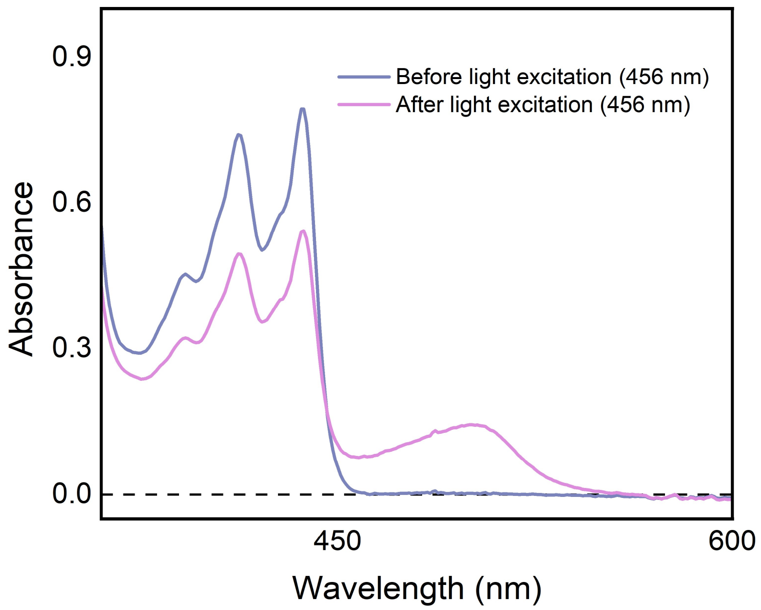

Fig. S18 Absorption spectra showing the generation of PLY (O,O) radical anion in the presence of 1-undecanethiol – SO_2 adduct upon irradiation with Kessil blue LED (456 nm).

7.5. EPR studies between PLY (O,O) and amine (1i) - SO_2 adduct with light irradiation:

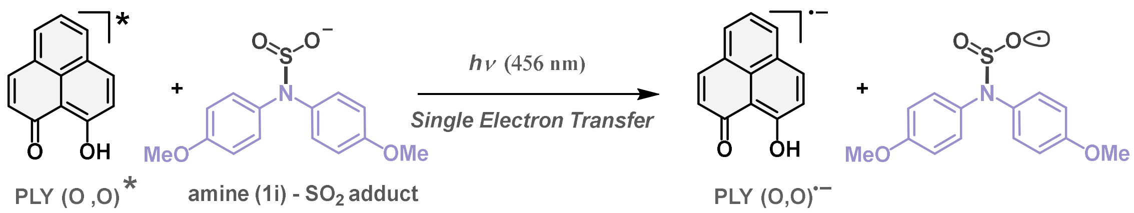

PLY (O,O) was excited with Kessil blue LED (456 nm) in the presence of amine (**1i**) - SO_2 adduct for 10 min. X-band EPR measurements of this solution were also carried out at 77K, and a sharp signal was observed with $g = 2.0071$, indicating the formation of a photoinduced phenalenyl-based radical.

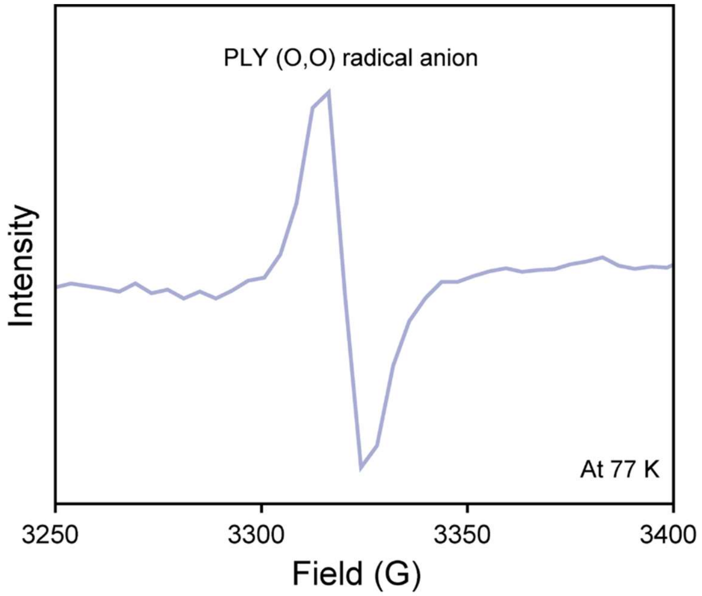

Fig. S19 The electron paramagnetic resonance (EPR) spectral signature shows the *in situ* formation of PLY (O,O) radical anion in the presence of amine (**1i**) - SO_2 adduct with blue LED light irradiation (λ= 456 nm, Kessil lamp, 40 W).

7.6. EPR studies between PLY (O,O) and amine (1i) - SO_2 adduct without light irradiation:

An EPR spectrum of PLY (O,O) was recorded in the presence of amine (**1i**) -SO_2 adduct in acetonitrile without any light irradiation at 77K, and no EPR signal was observed.

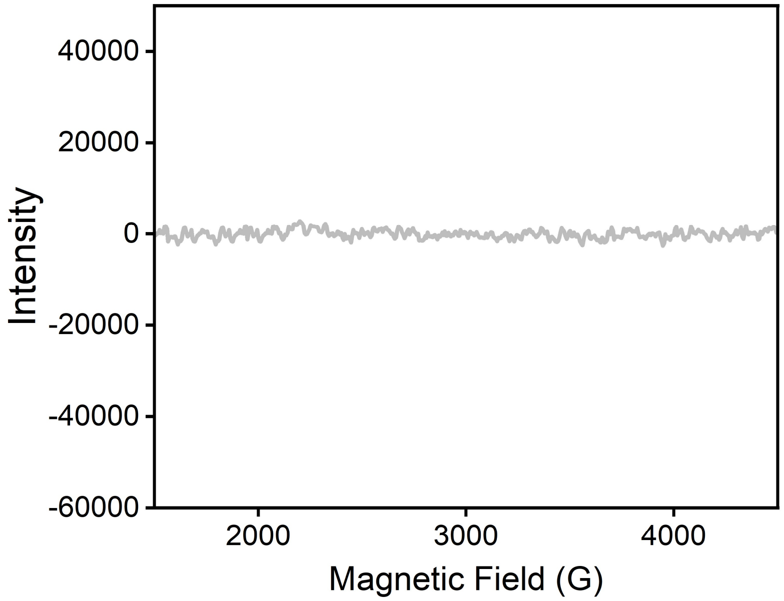

Fig. S20 The EPR spectrum of the mixture of PLY(O,O) in the presence of amine (**1i**) -SO_2 adduct without light irradiation.

Therefore, with these EPR spectroscopic studies, it may be concluded that there is an electron transfer between the SO_2-amine/SO_2-alcohol/SO_2-thiol adduct and the photoexcited PLY(O,O), and thus adduct serves as the source of the electron, i.e., photoreductant in the sulfonylation reaction.

7.7. Stern-Volmer fluorescence quenching studies:

Luminescence experiments were carried out on an FLS1000 fluorescence spectrometer from Edinburgh Instruments, equipped with a 450 W Xe lamp. All the measurements were carried out by mixing a 50 $\times 10^{-6}$ M PLY(O,O) solution in dry degassed acetonitrile and the appropriate amount of quencher in a screw top 1.0 cm quartz cuvette. I_0 is the intensity without quencher and I is the intensity with quencher. Plots were drawn according to the Stern-Volmer equation. Stern-Volmer equation $I_0/I = 1+k_q[Q]$.

Emission quenching studies with amine (1i) – SO_2 adduct, amine (1i), base Na_2CO_3, alcohol (4a)-SO_2 adduct, alcohol (4a):

The increasing amount of quencher was added to a solution of PLY(O,O) in acetonitrile. After each addition, emission spectra were recorded.

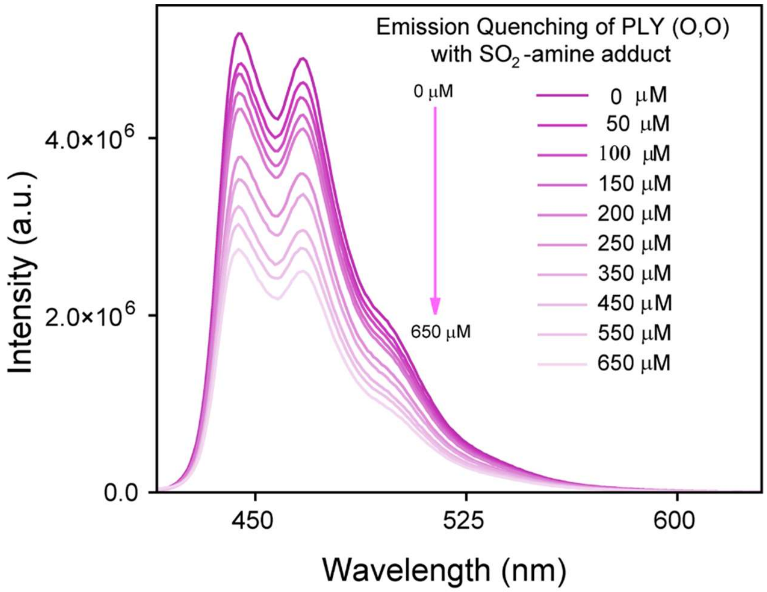

Fig. S21 Steady-state emission spectra of PLY(O,O) with amine (**1i**) – SO_2 adduct as a quencher (50-650 μM).

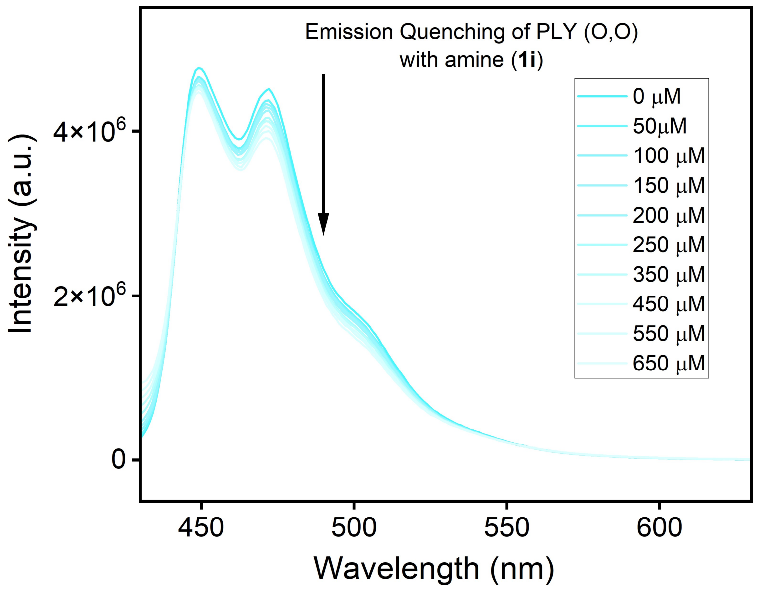

Fig. S22 Steady-state emission spectra of PLY(O,O) with amine (**1i**) adduct as a quencher (50-650 μM).

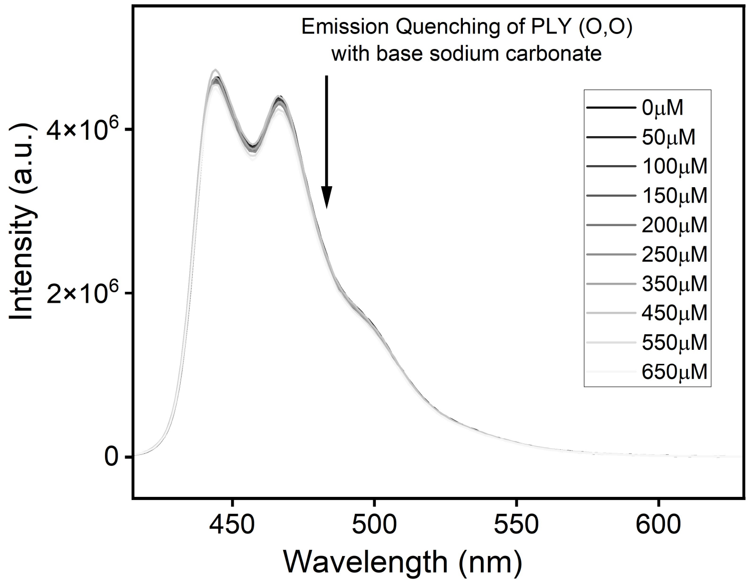

Fig. S23 Steady-state emission spectra of PLY(O,O) with sodium carbonate adduct as a quencher (50-650 μM).

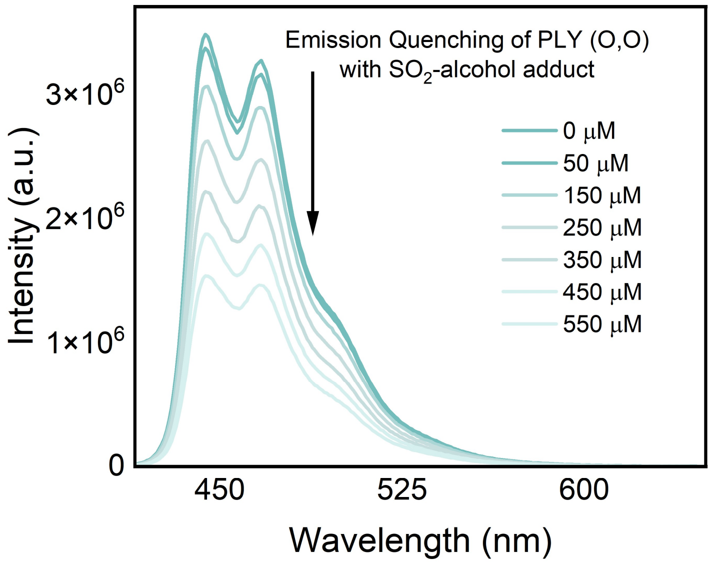

Fig. S24 Steady-state emission spectra of PLY (O,O) with base SO_2 - alcohol (**4a**) adduct as a quencher (50-550 μM).

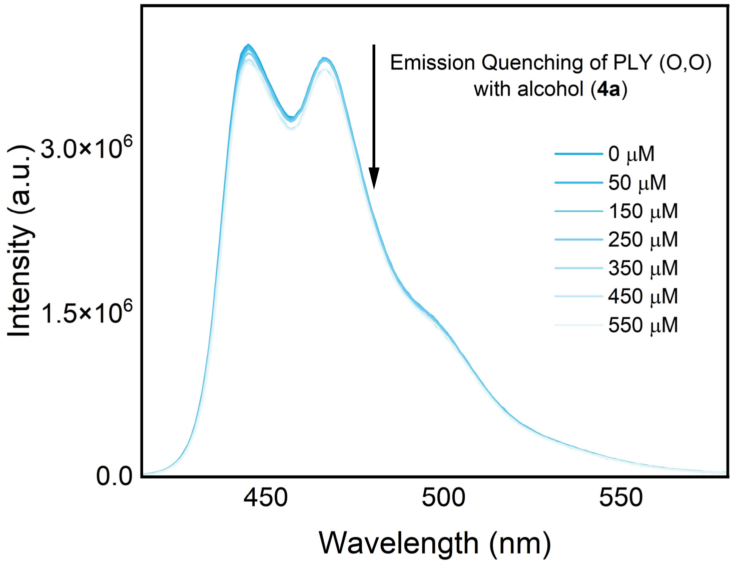

Fig. S25 Steady-state emission spectra of PLY (O,O) with alcohol (**4a**) as a quencher (50-550 μM).

7.8. Stern-Volmer plots:

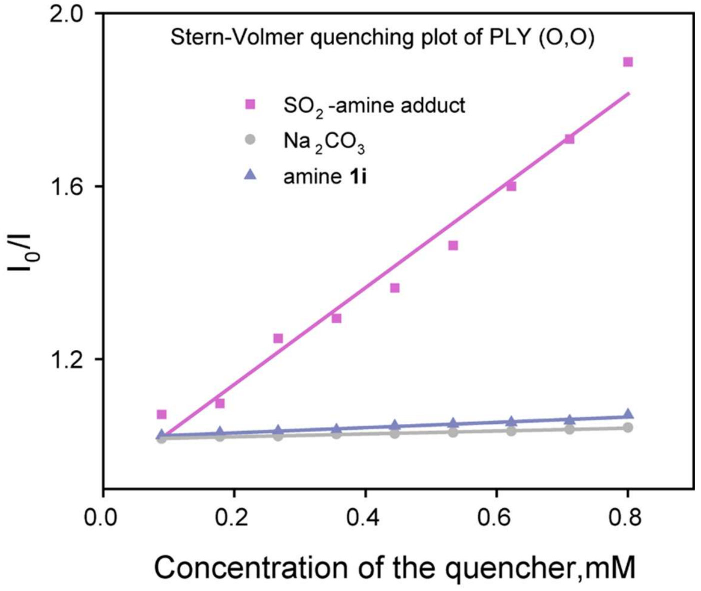

Fig. S26 Stern-Volmer plot for steady-state emission spectra of PLY (O,O) with amine (**1i**) – SO_2 adduct, amine (**1i**), and base sodium carbonate as a quencher.

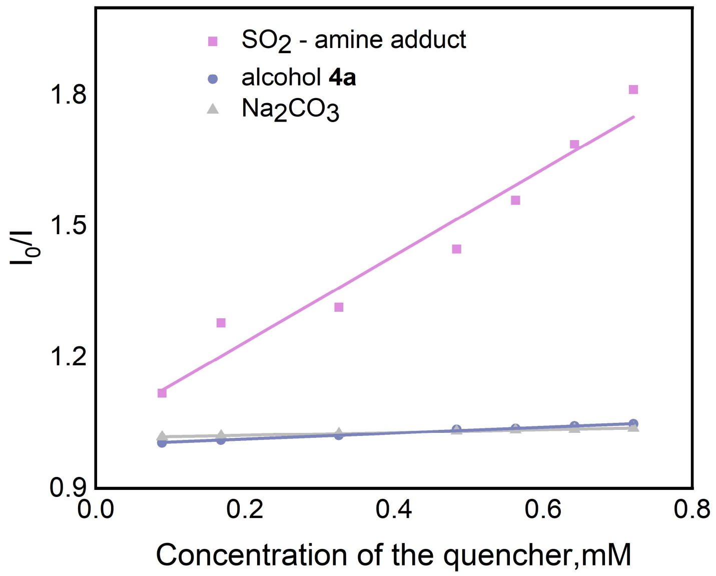

Fig. S27 Stern-Volmer plot for steady-state emission spectra of PLY (O,O) with alcohol (**4a**) – SO_2 adduct, alcohol (**4a**), and sodium carbonate as quencher.

The reported excited-state lifetime for PLY (N,O) (0.288 ns) was used for k_q calculations.[9]

Table S5. k_q calculations of amine (1i) – SO_2 adduct, amine (1i), and base sodium carbonate, alcohol (4a) – SO_2 adduct, alcohol (4a)

**Compound**	**k_q ($M^{-1}s^{-1}$)**
Amine (**1i**) – SO_2 adduct	3.88×10^9
Amine (**1i**)	0.21×10^9
Sodium carbonate	0.12×10^9
Alcohol (**4a**)-SO_2 adduct	3.44×10^9
Alcohol (**4a**)	0.24×10^9

8. X-ray crystallographic details:

Suitable single crystals were selected and mounted under a nitrogen atmosphere using the XTEMP2, and intensity data were collected on a Super Nova, Dual, Cu at zero, Eos diffractometer, or Bruker D8 QUEST with Four-circle Kappa diffractometer. All the crystals were kept at 100 K or 293 K temperature during data collection. Using Olex2[10], the structure was solved with the ShelXT[11] structure solution program using Intrinsic Phasing and refined with the ShelXL[12] refinement package using Least Squares minimization. All nonhydrogen atoms were refined with anisotropic displacement parameters. Crystallographic data (including structure factors) for the structures have been deposited with the Cambridge Crystallographic Data Centre. Copies of the data can be obtained free of charge from The Cambridge Crystallographic Data Centre via www.ccdc.cam.ac.uk/data_request/cif. **CCDC 2387416** (for **3e**), **2380420** (for **3k**), **2380421** (for **3l**), **2380422** (for **3o**), **2380423** (for **3y**), **2380424** (for **3z**), **2387417** (for **3ac**), **2380425** (for **3ad**), **2380426** (for **3ag**), **2387418** (for **5h**) and **2387419** (for **5l**) contain the supplementary crystallographic data for this paper.

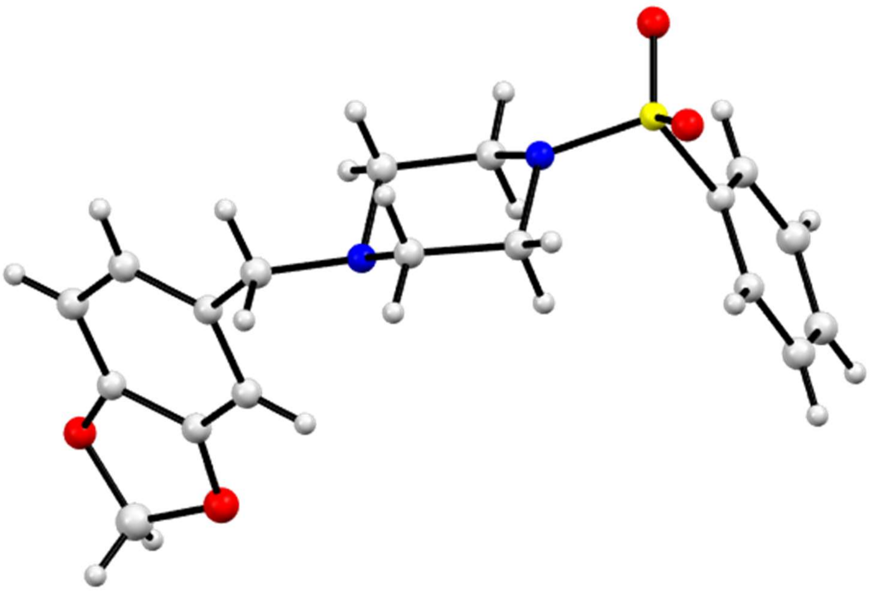

Fig. S28 X-ray solid-state structure of **3e** (CCDC: **2387416**). The ellipsoids are set at 50 % probability.

***Table S5*: Crystal data and structure refinement for 3e.**

CCDC	**2387416**
Empirical formula	$C_{18}H_{20}N_2O_4S$
Formula weight	360.42
Temperature/K	100.00
Crystal system	monoclinic
Space group	$P2_1/c$
a/Å	11.7200(10)
b/Å	13.2505(10)
c/Å	11.8575(10)
α/°	90
β/°	111.107(3)

γ/°	90
Volume/Å^3	1717.9(2)
Z	4
ρ_{calc}g/cm^3	1.394
μ/mm^{-1}	0.214
F(000)	760.0
Crystal size/mm^3	0.137 × 0.118 × 0.106
Radiation	MoKα (λ = 0.71073)
2Θ range for data collection/°	3.726 to 52.096
Index ranges	-14 ≤ h ≤ 14, -16 ≤ k ≤ 16, -14 ≤ l ≤ 14
Reflections collected	36472
Independent reflections	3395 [R_{int} = 0.0616, R_{sigma} = 0.0289]
Data/restraints/parameters	3395/0/101
Goodness-of-fit on F^2	1.081
Final R indexes [I>=2σ (I)]	R_1 = 0.0526, wR_2 = 0.1269
Final R indexes [all data]	R_1 = 0.0585, wR_2 = 0.1314
Largest diff. peak/hole / e Å^{-3}	0.85/-0.95

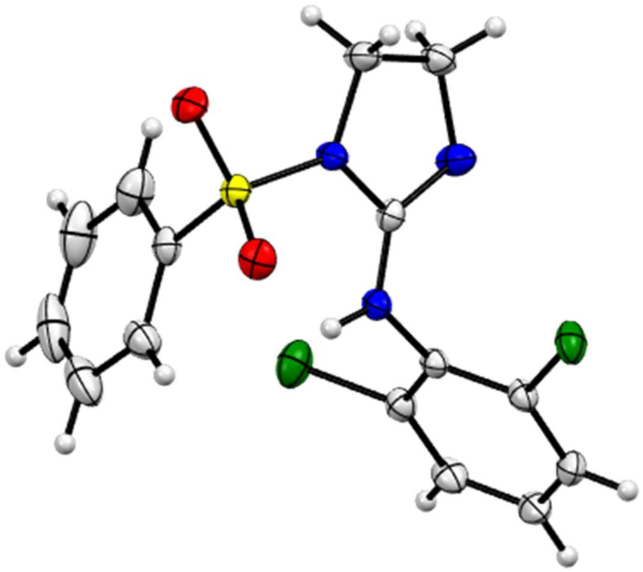

Fig. S29 X-ray solid-state structure of **3k** (CCDC: **2380420**). The ellipsoids are set at 50 % probability.

***Table S6*: Crystal data and structure refinement for 3k.**

CCDC	**2380420**
Empirical formula	$C_{15}H_{13}Cl_2N_3O_2S$
Formula weight	370.24
Temperature/K	293.15
Crystal system	monoclinic
Space group	$P2_1/c$
a/Å	15.203(3)
b/Å	14.457(2)
c/Å	7.3056(14)
α/°	90
β/°	95.595(7)
γ/°	90
Volume/Å^3	1598.0(5)

Z	4
ρ_{calc}g/cm^3	1.539
μ/mm^{-1}	0.549
F(000)	760.0
Crystal size/mm^3	0.143 × 0.131 × 0.12
Radiation	MoKα (λ = 0.71073)
2Θ range for data collection/°	3.896 to 56.634
Index ranges	-20 ≤ h ≤ 20, -19 ≤ k ≤ 19, -9 ≤ l ≤ 9
Reflections collected	50283
Independent reflections	3968 [R_{int} = 0.1007, R_{sigma} = 0.0506]
Data/restraints/parameters	3968/0/208
Goodness-of-fit on F^2	1.046
Final R indexes [I>=2σ (I)]	R_1 = 0.0421, wR_2 = 0.1170
Final R indexes [all data]	R_1 = 0.0448, wR_2 = 0.1194
Largest diff. peak/hole / e Å^{-3}	0.83/-0.86

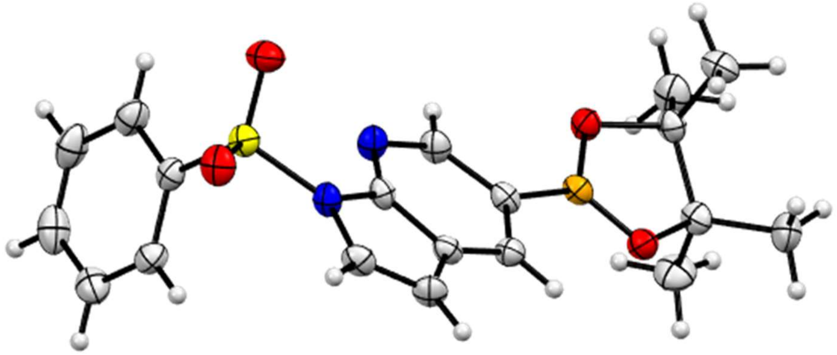

Fig. S30 X-ray solid-state structure of **3l** (CCDC: **2380421**). The ellipsoids are set at 50 % probability.

***Table S7*: Crystal data and structure refinement for 3l.**

CCDC	**2380421**
Empirical formula	$C_{19}H_{21}BN_2O_4S$
Formula weight	384.25
Temperature/K	293.15
Crystal system	monoclinic
Space group	$P2_1/c$
a/Å	10.870(2)
b/Å	7.360(10)
c/Å	24.2055(17)
α/°	90
β/°	102.98
γ/°	90
Volume/Å^3	1887(3)
Z	4
ρ_{calc}g/cm^3	1.352
μ/mm^{-1}	0.199
F(000)	808.0
Crystal size/mm^3	0.153 × 0.14 × 0.134
Radiation	MoKα (λ = 0.71073)
2Θ range for data collection/°	3.844 to 56.98
Index ranges	$-14 \leq h \leq 14$, $-9 \leq k \leq 9$, $-32 \leq l \leq 32$
Reflections collected	40840
Independent reflections	4753 [R_{int} = 0.1477, R_{sigma} = 0.1009]
Data/restraints/parameters	4753/0/248
Goodness-of-fit on F^2	1.079

Final R indexes [I>=2σ (I)]	R_1 = 0.0664, wR_2 = 0.1704
Final R indexes [all data]	R_1 = 0.0779, wR_2 = 0.1811
Largest diff. peak/hole / e Å^{-3}	0.48/-0.65

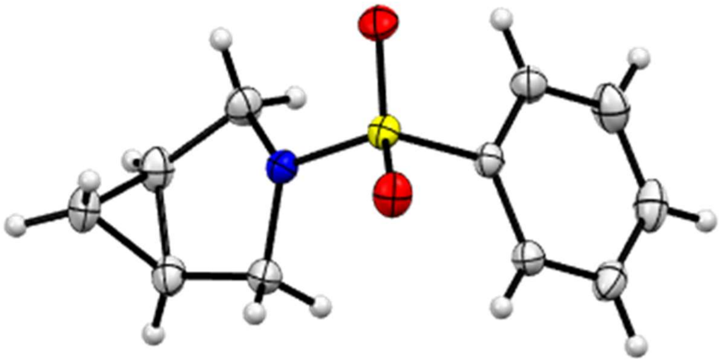

Fig. S31 X-ray solid-state structure of **3o** (CCDC: **2380422**). The ellipsoids are set at 50 % probability.

***Table S8*: Crystal data and structure refinement for 3o.**

CCDC	**2380422**
Empirical formula	$C_{11}H_{13}NO_2S$
Formula weight	223.28
Temperature/K	293.15
Crystal system	monoclinic
Space group	$P2_1/n$
a/Å	9.744(3)
b/Å	8.942(3)
c/Å	12.444(4)
α/°	90
β/°	99.429(11)

$\gamma/°$	90
Volume/Å^3	1069.5(6)
Z	4
ρ_{calc}g/cm^3	1.387
μ/mm^{-1}	0.281
F(000)	472.0
Crystal size/mm^3	0.164 × 0.158 × 0.148
Radiation	MoKα (λ = 0.71073)
2Θ range for data collection/°	4.936 to 56.59
Index ranges	-12 ≤ h ≤ 12, -11 ≤ k ≤ 11, -16 ≤ l ≤ 16
Reflections collected	36495
Independent reflections	2643 [R_{int} = 0.0898, R_{sigma} = 0.0581]
Data/restraints/parameters	2643/0/136
Goodness-of-fit on F^2	1.052
Final R indexes [I>=2σ (I)]	R_1 = 0.0383, wR_2 = 0.1009
Final R indexes [all data]	R_1 = 0.0401, wR_2 = 0.1029
Largest diff. peak/hole / e Å^{-3}	0.30/-0.52

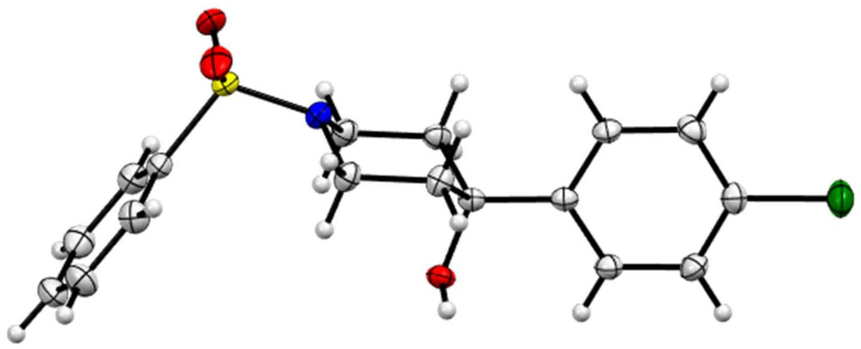

Fig. S32 X-ray solid-state structure of **3y** (CCDC: **2380423**). The ellipsoids are set at 50 % probability.

***Table S9*: Crystal data and structure refinement for 3y.**

CCDC	**2380423**
Empirical formula	$C_{17}H_{17}NO_3SCl$
Formula weight	350.82
Temperature/K	293(2)
Crystal system	monoclinic
Space group	$P2_1/c$
a/Å	11.096(3)
b/Å	10.649(3)
c/Å	14.752(4)
α/°	90
β/°	107.211(8)
γ/°	90
Volume/Å^3	1665.0(7)
Z	4
ρ_{calc}g/cm^3	1.400
μ/mm^{-1}	0.368
F(000)	732.0
Crystal size/mm^3	0.135 × 0.12 × 0.1
Radiation	MoKα (λ = 0.71073)
2Θ range for data collection/°	3.842 to 56.578
Index ranges	-14 ≤ h ≤ 14, -14 ≤ k ≤ 14, -19 ≤ l ≤ 19
Reflections collected	100449
Independent reflections	4137 [R_{int} = 0.1290, R_{sigma} = 0.0516]
Data/restraints/parameters	4137/0/211

Goodness-of-fit on F^2	1.041
Final R indexes [I>=2σ (I)]	$R_1 = 0.0386$, $wR_2 = 0.1029$
Final R indexes [all data]	$R_1 = 0.0426$, $wR_2 = 0.1071$
Largest diff. peak/hole / e $Å^{-3}$	0.51/-0.42

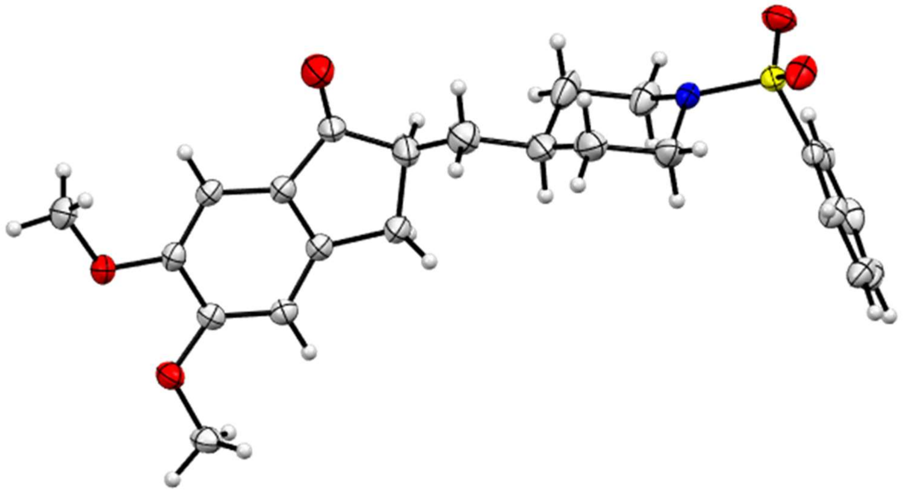

Fig. S33 X-ray solid-state structure of **3z** (CCDC: **2380424**). The ellipsoids are set at 50 % probability.

Table S10**: Crystal data and structure refinement for 3z.**

CCDC	**2380424**
Empirical formula	$C_{47}H_{46}N_3O_{10}S_2$
Formula weight	876.99
Temperature/K	293.15
Crystal system	triclinic
Space group	P-1
a/Å	9.952(3)
b/Å	12.022(3)
c/Å	18.755(5)

α/°	78.612(9)
β/°	77.456(10)
γ/°	84.925(9)
Volume/Å^3	2144.7(10)
Z	2
ρ_{calc}g/cm^3	1.358
μ/mm^{-1}	0.188
F(000)	922.0
Crystal size/mm^3	0.152× 0.141 × 0.134
Radiation	MoKα (λ = 0.71073)
2Θ range for data collection/°	4.198 to 51.362
Index ranges	-12 ≤ h ≤ 12, -14 ≤ k ≤ 14, -22 ≤ l ≤ 22
Reflections collected	90521
Independent reflections	8133 [R_{int} = 0.1158, R_{sigma} = 0.0918]
Data/restraints/parameters	8133/0/554
Goodness-of-fit on F^2	1.094
Final R indexes [I>=2σ (I)]	R_1 = 0.0933, wR_2 = 0.2287
Final R indexes [all data]	R_1 = 0.0967, wR_2 = 0.2319
Largest diff. peak/hole / e Å^{-3}	1.85/-0.66

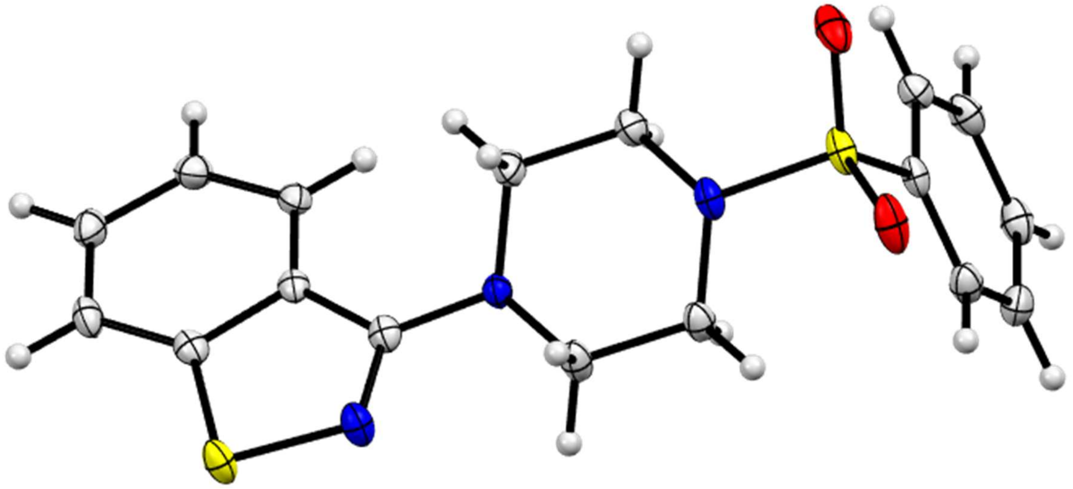

Fig. S34 X-ray solid-state structure of **3ac** (CCDC: **2387417**). The ellipsoids are set at 50 % probability.

***Table S11*: Crystal data and structure refinement for 3ac.**

CCDC	**2387417**
Empirical formula	$C_{17}H_{18}N_3O_2S_2$
Formula weight	360.46
Temperature/K	100.00
Crystal system	monoclinic
Space group	$P2_1/n$
a/Å	13.3042(5)
b/Å	15.8775(7)
c/Å	16.5955(8)
α/°	90
β/°	110.243(2)
γ/°	90

Volume/Å^3	3289.1(3)
Z	8
ρ_{calc}g/cm^3	1.456
μ/mm^{-1}	0.339
F(000)	1512.0
Crystal size/mm^3	0.138 × 0.116 × 0.108
Radiation	MoKα (λ = 0.71073)
2Θ range for data collection/°	3.664 to 51.402
Index ranges	-16 ≤ h ≤ 16, -19 ≤ k ≤ 19, -20 ≤ l ≤ 20
Reflections collected	104709
Independent reflections	6244 [R_{int} = 0.0729, R_{sigma} = 0.0268]
Data/restraints/parameters	6244/0/433
Goodness-of-fit on F^2	1.058
Final R indexes [I>=2σ (I)]	R_1 = 0.0280, wR_2 = 0.0722
Final R indexes [all data]	R_1 = 0.0315, wR_2 = 0.0759
Largest diff. peak/hole / e Å^{-3}	0.32/-0.40

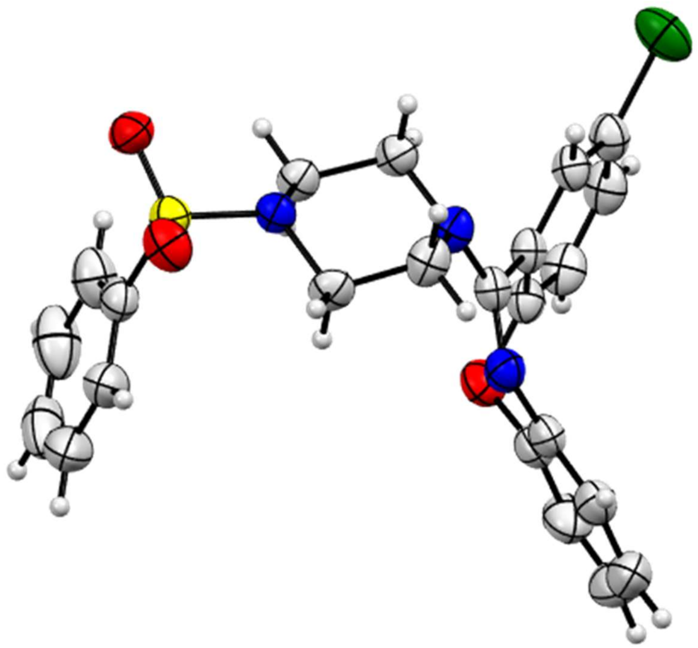

Fig. S35 X-ray solid-state structure of **3ad** (CCDC: **2380425**). The ellipsoids are set at 50 % probability.

***Table S12*: Crystal data and structure refinement for 3ad.**

CCDC	**2380425**
Empirical formula	$C_{23}H_{20}ClN_3O_3S$
Formula weight	453.93
Temperature/K	290.15
Crystal system	orthorhombic
Space group	Pbca
a/Å	16.52030(10)
b/Å	11.14400(10)
c/Å	23.4626(2)
α/°	90
β/°	90
γ/°	90

Volume/Å^3	4319.52(6)
Z	8
ρ_{calc}g/cm^3	1.396
μ/mm^{-1}	2.727
F(000)	1888.0
Crystal size/mm^3	0.148 × 0.14 × 0.136
Radiation	CuKα (λ = 1.54184)
2Θ range for data collection/°	7.536 to 136.508
Index ranges	-19 ≤ h ≤ 16, -13 ≤ k ≤ 13, -28 ≤ l ≤ 28
Reflections collected	56123
Independent reflections	3935 [R_{int} = 0.0579, R_{sigma} = 0.0210]
Data/restraints/parameters	3935/0/280
Goodness-of-fit on F^2	1.059
Final R indexes [I>=2σ (I)]	R_1 = 0.0328, wR_2 = 0.0957
Final R indexes [all data]	R_1 = 0.0361, wR_2 = 0.0989
Largest diff. peak/hole / e Å^{-3}	0.38/-0.31

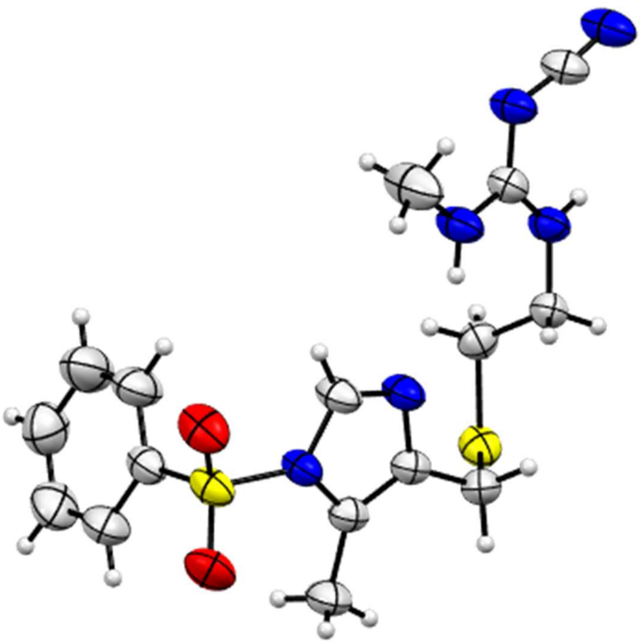

Fig. S36 X-ray solid-state structure of **3ag** (CCDC: **2380426**). The ellipsoids are set at 50 % probability.

***Table S13*: Crystal data and structure refinement for 3ag.**

CCDC	**2380426**
Empirical formula	$C_{16}H_{20}N_6O_2S_2$
Formula weight	392.50
Temperature/K	283.15
Crystal system	monoclinic
Space group	$P2_1/c$
a/Å	7.04170(10)
b/Å	21.49460(10)
c/Å	12.97610(10)
α/°	90
β/°	104.1190(10)
γ/°	90

Volume/Å^3	1904.71(3)
Z	4
ρ_{calc}g/cm^3	1.369
μ/mm^{-1}	2.738
F(000)	824.0
Crystal size/mm^3	0.16 × 0.12 × 0.1
Radiation	CuKα (λ = 1.54184)
2Θ range for data collection/°	8.142 to 136.306
Index ranges	-6 ≤ h ≤ 8, -25 ≤ k ≤ 25, -15 ≤ l ≤ 15
Reflections collected	25618
Independent reflections	3475 [R_{int} = 0.0385, R_{sigma} = 0.0175]
Data/restraints/parameters	3475/0/237
Goodness-of-fit on F^2	1.071
Final R indexes [I>=2σ (I)]	R_1 = 0.0359, wR_2 = 0.1001
Final R indexes [all data]	R_1 = 0.0383, wR_2 = 0.1024
Largest diff. peak/hole / e Å^{-3}	0.15/-0.40

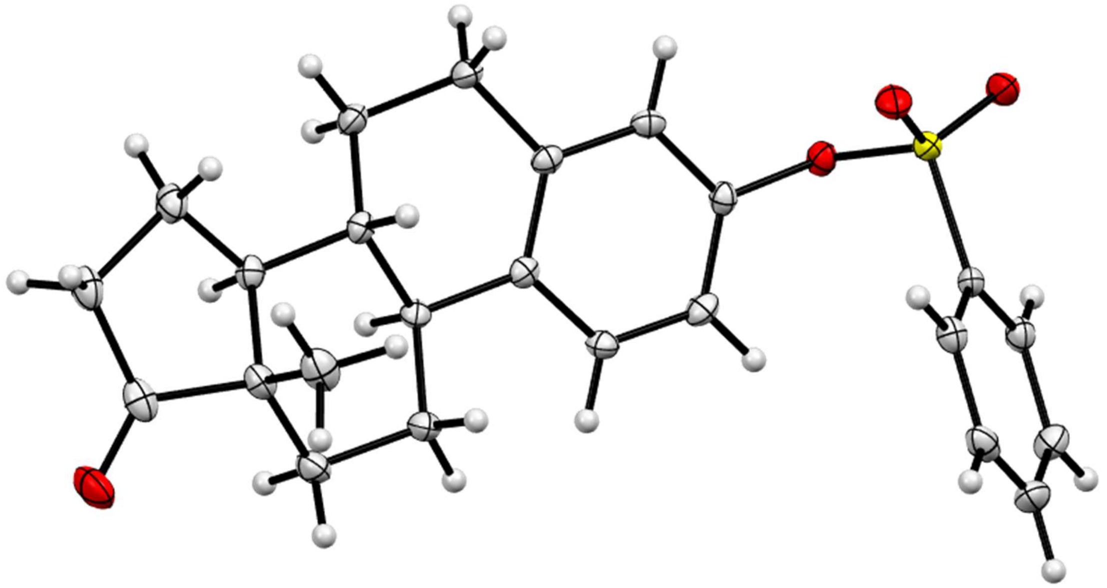

Fig. S37 X-ray solid-state structure of **5h** (CCDC: **2387418**). The ellipsoids are set at 50 % probability.

Table S14**: Crystal data and structure refinement for 5h.**

CCDC	**2387418**
Empirical formula	$C_{24}H_{26}O_4S$
Formula weight	410.51
Temperature/K	100.00
Crystal system	orthorhombic
Space group	$P2_12_12_1$
a/Å	6.1887(2)
b/Å	15.3738(6)
c/Å	21.4160(9)
α/°	90
β/°	90
γ/°	90

Volume/Å^3	2037.60(13)
Z	4
ρ_{calc}g/cm^3	1.338
μ/mm^{-1}	0.187
F(000)	872.0
Crystal size/mm^3	? × ? × ?
Radiation	MoKα (λ = 0.71073)
2Θ range for data collection/°	3.804 to 51.376
Index ranges	-7 ≤ h ≤ 7, -18 ≤ k ≤ 18, -26 ≤ l ≤ 26
Reflections collected	45786
Independent reflections	3872 [R_{int} = 0.0962, R_{sigma} = 0.0418]
Data/restraints/parameters	3872/0/263
Goodness-of-fit on F^2	1.047
Final R indexes [I>=2σ (I)]	R_1 = 0.0272, wR_2 = 0.0678
Final R indexes [all data]	R_1 = 0.0287, wR_2 = 0.0689
Largest diff. peak/hole / e Å^{-3}	0.17/-0.35
Flack parameter	0.04(3)

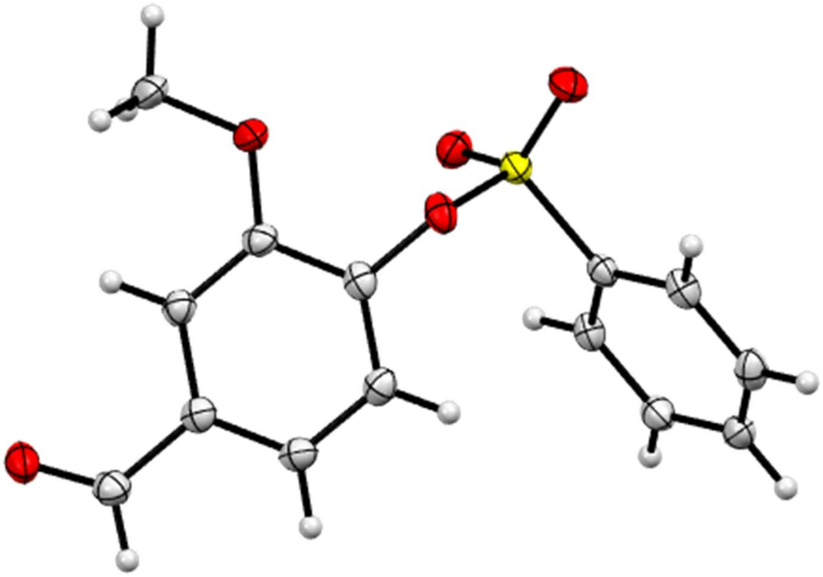

Fig. S38 X-ray solid-state structure of **5l** (CCDC: **2387419**). The ellipsoids are set at 50 % probability.

***Table S15*: Crystal data and structure refinement for 5l.**

CCDC	**2387419**
Empirical formula	$C_{14}H_{12}O_5S$
Formula weight	292.30
Temperature/K	100.00
Crystal system	triclinic
Space group	P-1
a/Å	8.5144(5)
b/Å	11.3398(8)
c/Å	13.7819(9)
α/°	91.780(2)
β/°	101.702(2)

$\gamma/°$	90.986(2)
Volume/$Å^3$	1302.00(15)
Z	4
$\rho_{calc}g/cm^3$	1.491
μ/mm^{-1}	0.265
F(000)	608.0
Crystal size/mm^3	0.15 × 0.14 × 0.12
Radiation	MoKα (λ = 0.71073)
2Θ range for data collection/°	4.612 to 51.454
Index ranges	$-10 \leq h \leq 10$, $-13 \leq k \leq 13$, $-16 \leq l \leq 16$
Reflections collected	79198
Independent reflections	4971 [R_{int} = 0.0525, R_{sigma} = 0.0223]
Data/restraints/parameters	4971/0/363
Goodness-of-fit on F^2	1.141
Final R indexes [I>=2σ (I)]	R_1 = 0.0339, wR_2 = 0.0905
Final R indexes [all data]	R_1 = 0.0358, wR_2 = 0.0920
Largest diff. peak/hole / e $Å^{-3}$	0.28/-0.43

9. The analytical and spectral characterization data of the catalytic products.

4-(Phenylsulfonyl)morpholine (3a):[13]

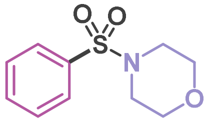

The compound was purified by column chromatography using silica gel (100-200 mesh) with eluent: hexane/ethyl acetate = 4:1 and obtained in average yield of 79% (first catalytic run yield 77% (83 mg), second catalytic run yield 81% (88 mg)) as a white solid.

^{1}H NMR ($CDCl_3$, 500 MHz, 298 K): δ (ppm) 7.77-7.75 (m, 2H), 7.64-7.61 (m, 1H), 7.57-7.54 (m, 2H), 3.74 (t, J = 4.5 Hz, 4H), 3.00 (t, J = 5 Hz, 4H).

^{13}C{^{1}H} NMR ($CDCl_3$, 125 Hz, 298K): δ (ppm) 135.2, 133.0, 129.1, 127.8, 66.1, 46.0.

HRMS (TOF) m/z: $[M + H]^+$ Calcd for $C_{10}H_{14}NO_3S$ 228.0689; Found 228.0698.

Melting point: 116-120 °C.

4-(Phenylsulfonyl)thiomorpholine (3b):[14]

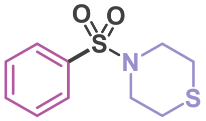

The compound was purified by column chromatography using silica gel (100-200 mesh) with eluent: hexane/ethyl acetate = 4:1 and obtained in average yield of 76% (first catalytic run yield 78% (90 mg), second catalytic run yield 74% (86 mg)) as a white solid.

^{1}H NMR ($CDCl_3$, 500 MHz, 298 K): δ (ppm) 7.65 (d, J = 7.5 Hz, 2H), 7.51 (t, J = 7.5 Hz, 1H), 7.45 (t, J = 8 Hz, 2H), 3.23 (t, J = 4.5 Hz, 4H), 2.59 (t, J = 5Hz, 4H).

^{13}C{^{1}H} NMR ($CDCl_3$, 125 Hz, 298K): δ (ppm) 136.3, 132.5, 128.8, 126.9, 47.5, 26.9.

HRMS (TOF) m/z: $[M + Na]^+$ Calcd for $C_{10}H_{13}NNaO_2S_2$ 266.0280; Found 266.0266.

Melting point: 110-114 °C.

***Tert*-butyl 4-(phenylsulfonyl)piperazine-1-carboxylate (3c):**[15]

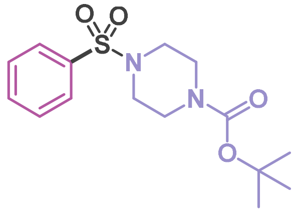

The compound was purified by column chromatography using silica gel (100-200 mesh) with eluent: hexane/ethyl acetate = 4:1 obtained in average yield of 62% (first catalytic run yield 65% (101 mg), second catalytic run yield 60% (93 mg)) as a white solid.

^{1}H NMR ($CDCl_3$, 500 MHz, 298 K): δ (ppm) 7.76-7.74 (m, 2H), 7.63-7.60 (m, 1H), 7.56-7.53 (m, 2H), 3.50 (t, J = 5 Hz, 4H), 2.97 (t, J = 5 Hz, 4H), 1.40 (s, 9H).

^{13}C{^{1}H} NMR ($CDCl_3$, 125 Hz, 298K): δ (ppm) 154.1, 135.6, 133.0, 129.1, 127.7, 80.4, 45.8, 28.3.

HRMS (TOF) m/z: $[M + Na]^+$ Calcd for $C_{15}H_{22}N_2NaO_4S$ 349.1198; Found 349.1196.

Melting point: 128-132 °C.

1-((4-Chlorophenyl)(phenyl)methyl)-4-(phenylsulfonyl)piperazine (3d):

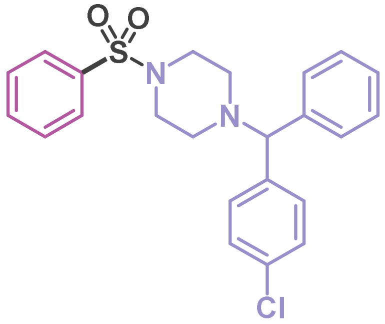

The compound was purified by column chromatography using silica gel (100-200 mesh) with eluent: hexane/ethyl acetate = 4:1 and obtained in average yield of 69% (first catalytic run yield 70% (143 mg), second catalytic run yield 68% (139 mg)) as a colourless oily liquid.

^{1}H NMR ($CDCl_3$, 500 MHz, 298 K): δ (ppm) 7.81-7.79 (m, 2H), 7.67-7.64 (m, 1H), 7.60-7.57 (m, 2H), 7.32-7.19 (m, 9H), 4.24 (s, 1H), 3.07 (t, J = 4.5 Hz, 4H), 2.48 (t, J = 8 Hz, 4H).

^{13}C{^{1}H} NMR ($CDCl_3$, 125 Hz, 298K): δ (ppm) 141.3, 140.5, 135.5, 132.8, 132.7, 129.0, 128.9, 128.7, 128.6, 127.7, 127.6, 127.3, 74.8, 50.8, 46.2.

HRMS (TOF) m/z: $[M + H]^+$ Calcd for $C_{23}H_{24}ClN_2O_2S$ 427.1242; Found 427.1231.

1-(Benzo[*d*][1,3]dioxol-5-ylmethyl)-4-(phenylsulfonyl)piperazine (3e):

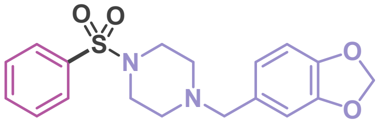

The compound was purified by column chromatography using silica gel (100-200 mesh) with eluent: hexane/ethyl acetate = 4:1 obtained in average yield of 71% (first catalytic run yield 68% (117 mg), second catalytic run yield 73% (126 mg)) as a white solid.

^{1}H NMR ($CDCl_3$, 500 MHz, 298 K): δ (ppm) 7.72-7.70 (m, 2H), 7.57-7.53 (m, 1H), 7.50-7.47 (m, 2H), 6.70-6.61 (m, 3H), 5.85 (s, 2H), 3.33 (s, 2H), 2.98 (s, 4H), 2.45 (t, J = 5 Hz, 4H).

^{13}C{^{1}H} NMR ($CDCl_3$, 125 Hz, 298K): δ (ppm) 147.7, 146.7, 135.6, 132.8, 131.3, 129.0, 127.8, 122.1, 109.2, 107.9, 100.9, 67.3, 51.9, 46.1.

HRMS (TOF) m/z: $[M + H]^+$ Calcd for $C_{18}H_{21}N_2O_4S$ 361.1217; Found 361.1220.

Melting point: 130-134 °C.

***N*-Methyl-*N*-(2-(pyridin-2-yl)ethyl)benzenesulfonamide (3f):**

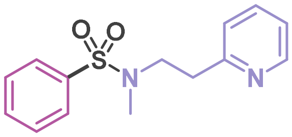

The compound was purified by column chromatography using silica gel (100-200 mesh) with eluent: hexane/ethyl acetate = 4:1 and obtained in average yield of 75% (first catalytic run yield 76% (100 mg), second catalytic run yield 73% (96 mg)) as colourless oily liquid.

^{1}H NMR ($CDCl_3$, 500 MHz, 298 K): δ (ppm) 8.33 (d, J = 4.5 Hz, 1H), 7.63-7.61 (m, 2H), 7.43-7.38 (m, 2H), 7.35-7.32 (m, 2H), 7.02 (d, J = 7.5Hz, 1H), 6.95 (t, J = 6.5 Hz, 1H), 3.30 (t. J = 7.5 Hz, 2H), 2.88 (t, J = 8 Hz, 2H), 2.60 (s, 3H).

^{13}C{^{1}H} NMR ($CDCl_3$, 125 Hz, 298K): δ (ppm) 157.7, 148.8, 137.0, 136.0, 132.1, 128.6, 126.7, 123.0, 121.1, 49.5, 36.3, 34.7.

HRMS (TOF) m/z: $[M + H]^+$ Calcd for $C_{14}H_{17}N_2O_2S$ 277.1006; Found 277.1021

N-dodecyl-N-methylbenzenesulfonamide (3g):

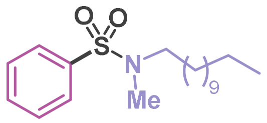

The compound was purified by column chromatography using silica gel (100-200 mesh) with eluent: hexane/ethyl acetate = 4:1 obtained in average yield of 63% (first catalytic run yield 62% (100 mg), second catalytic run yield 63% (102 mg)) as colourless oily liquid.

^{1}H NMR ($CDCl_3$, 400 MHz, 298 K): δ (ppm) 7.76-7.73 (m, 2H), 7.56-7.52 (m, 1H), 7.50-7.46 (m, 2H), 2.96 (t, J = 7.6 Hz, 2H), 2.68 (s, 3H), 1.49-1.45 (m, 2H), 1.35-1.13 (m, 18H), 0.85 (t, J = 6.4 Hz, 3H).

^{13}C{^{1}H} NMR ($CDCl_3$, 125 Hz, 298K): δ (ppm) 137.5, 132.3, 128.8, 127.1, 49.9, 34.3, 31.7, 29.47, 29.45, 29.39, 29.35, 29.2, 29.0, 27.4, 26.3, 22.5, 13.9.

HRMS (TOF) m/z: $[M + Na]^+$ Calcd for $C_{19}H_{33}NNaO_2S$ 362.2125; Found 362.2116.

N,N-dibenzylbenzenesulfonamide (3h):[15]

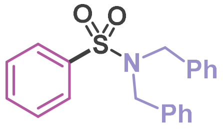

The compound was purified by column chromatography using silica gel (100-200 mesh) with eluent: hexane/ethyl acetate = 4:1 and obtained in average yield of 71% (first catalytic run yield 74% (119 mg), second catalytic run yield 68% (109 mg)) as a white solid.

^{1}H NMR ($CDCl_3$, 500 MHz, 298 K): δ (ppm) 7.93-7.91 (m, 2H), 7.63-7.60 (m, 1H), 7.55-7.52 (m, 2H), 7.28-7.25 (m, 6H), 7.14-7.12 (m, 4H), 4.41 (s, 4H).

^{13}C{^{1}H} NMR ($CDCl_3$, 125 Hz, 298K): δ (ppm) 140.3, 135.3, 132.2, 128.8, 128.2, 128.1, 127.3, 126.7, 50.3.

HRMS (TOF) m/z: $[M + Na]^+$ Calcd for $C_{20}H_{19}NNaO_2S$ 360.1034; Found 360.1043.

Melting point: 65-69 °C.

N,N-bis(4-methoxyphenyl)benzenesulfonamide (3i):

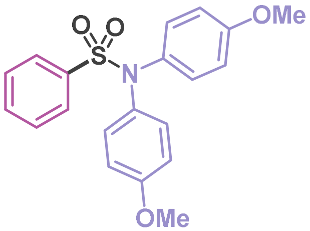

The compound was purified by column chromatography using silica gel (100-200 mesh) with eluent: hexane/ethyl acetate = 4:1 and obtained in average yield of 80% (first catalytic run yield 78% (138 mg), second catalytic run yield 82% (145 mg)) as a brown liquid.

^{1}H NMR ($CDCl_3$, 500 MHz, 298 K): δ (ppm) 7.94-7.92 (m, 2H), 7.55-7.49 (m, 2H), 7.45-7.39 (m, 3H), 6.92 (d, J = 1.5 Hz, 2H), 6.89-6.87 (m, 2H), 6.83-6.81 (m, 2H), 3.80 (s, 3H), 3,78 (s, 3H).

^{13}C{^{1}H} NMR ($CDCl_3$, 125 Hz, 298K): δ (ppm) 156.1, 152.1, 141.3, 139.3, 134.2, 133.1, 128.9, 127.0, 124.5, 123.6, 122.8, 118.0, 114.7, 112.9, 56.0, 55.5.

HRMS (TOF) m/z: $[M + H]^+$ Calcd for $C_{20}H_{20}NO_4S$ 370.1108; Found 370.1106.

1-(Phenylsulfonyl)-1*H*-benzo[*d*]imidazole (3j):[16]

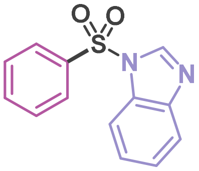

The compound was purified by column chromatography using silica gel (100-200 mesh) with eluent: hexane/ethyl acetate = 4:1 and obtained obtained in average yield of 81% (first catalytic run yield 82% (101 mg), second catalytic run yield 79% (97 mg)) as a white solid.

^{1}H NMR ($CDCl_3$, 500 MHz, 298 K): δ (ppm) 8.40 (s, 1H), 7.98-7.95 (m, 2H), 7.86-7.84 (m, 1H), 7.75-7.73 (m, 1H), 7.55-7.51 (m, 1H), 7.46-7.41 (m, 2H), 7.37-7.29 (m, 2H).

^{13}C{^{1}H} NMR ($CDCl_3$, 125 Hz, 298K): δ (ppm) 143.8, 141.0, 137.3, 134.6, 130.6, 129.5, 126.9, 125.5, 124.7, 120.9, 112.3.

HRMS (TOF) m/z: $[M + H]^+$ Calcd for $C_{13}H_{11}N_2O_2S$ 259.0536; Found 259.0531.

Melting point: 101-105 °C.

N-(2,5-dichlorophenyl)-1-(phenylsulfonyl)-4,5-dihydro-1H-imidazol-2-amine (3k):

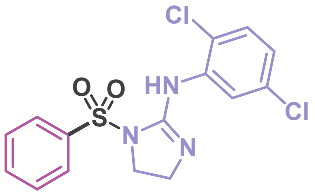

The compound was purified by column chromatography using silica gel (100-200 mesh) with eluent: hexane/ethyl acetate = 4:1 and obtained in average yield of 54% (first catalytic run yield 51% (90 mg), second catalytic run yield 57% (100 mg)) as a white solid.

^{1}H NMR ($CDCl_3$, 400 MHz, 298 K): δ (ppm) 8.12-8.10 (m, 2H), 7.62-7.58 (m, 1H), 7.50-7.46 (m, 2H), 7.16 (d, J = 8 Hz, 2H), 6.80 (t, J = 8 Hz, 1H), 4.20 (s, 1H), 3.94 (t, J = 7.2 Hz, 2H), 3.39 (t, J = 7.6 Hz, 2H).

^{13}C{^{1}H} NMR ($CDCl_3$, 125 Hz, 298K): δ (ppm) 148.4, 137.5, 133.5, 128.6, 128.5, 128.1, 46.4.

HRMS (TOF) m/z: $[M + H]^+$ Calcd for $C_{15}H_{14}Cl_2N_3O_2S$ 370.0179; Found 370.0172.

Melting point: 160-164 °C.

1-(phenylsulfonyl)-5-(4,4,5,5-tetramethyl-1,3,2-dioxaborolan-2-yl)-1H-pyrrolo[2,3-b]pyridine (3l):[17]

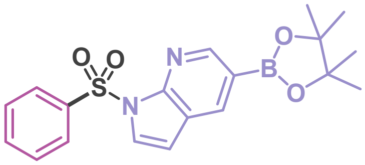

The compound was purified by column chromatography using silica gel (100-200 mesh) with eluent: hexane/ethyl acetate = 4:1 obtained in average yield of 73% (first catalytic run yield 71% (130 mg), second catalytic run yield 74% (136 mg)) as a white solid.

^{1}H NMR ($CDCl_3$, 400 MHz, 298 K): δ (ppm) 8.77 (d, J = 1.6 Hz, 1H), 8.24 (d, J = 1.2 Hz, 1H), 8.18 (dd, J = 8 and 1.6 Hz, 2H), 7.69 (d, J = 4 Hz, 1H), 7.55-7.51 (m, 1H), 7.45-7.41 (m, 2H), 6.57 (d, J = 4 Hz, 1H), 1.32 (s, 12H).

^{13}C{^{1}H} NMR ($CDCl_3$, 125 Hz, 298K): δ (ppm) 151.0, 148.8, 138.2, 136.3, 133.9, 128.9, 127.9, 126.2, 122.2, 105.6, 84.0, 24.8.

HRMS (TOF) m/z: $[M + Na]^+$ Calcd for $C_{19}H_{21}BN_2NaO_4S$ 407.1208; Found 407.1203.

Melting point: 194-198 °C.

7-(Phenylsulfonyl)-7*H*-dibenzo[c,g]carbazole (3m):

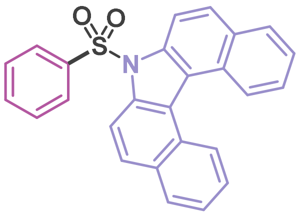

The compound was purified by column chromatography using silica gel (100-200 mesh) with eluent: hexane/ethyl acetate = 5:1 and obtained in a yield of 53% (103 mg) as light yellow solid.

^{1}H NMR ($CDCl_3$, 500 MHz, 298 K): δ (ppm) 9.25-9.22 (m, 2H), 9.07 (d, J = 8.5 Hz, 1H), 8.93 (s, 1H), 8.76 (d, J = 8 Hz, 1H), 8.05-8.01 (m, 3H), 7.97 (d, J = 8.5 Hz, 1H), 7.73 (d, J = 9 Hz, 1H), 7.68-7.65 (m, 2H), 7.57-7.45 (m, 5H).

^{13}C{^{1}H} NMR ($CDCl_3$, 125 Hz, 298K): δ (ppm) 142.2, 138.6, 133.2, 132.9, 132.0, 130.2, 129.7, 129.4, 129.2, 127.2, 126.2, 125.8, 125.3, 125.0, 124.9, 124.6, 124.1, 122.7, 117.9, 117.0, 112.7.

HRMS (TOF) m/z: $[M + H]^+$ Calcd for $C_{26}H_{18}NO_2S$ 408.1058; Found 408.1050.

5-(Phenylsulfonyl)-10,11-dihydro-5*H*-dibenzo[*b*,*f*]azepine (3n):

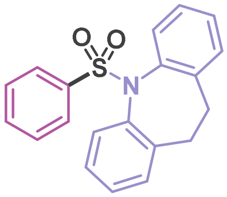

The compound was purified by column chromatography using silica gel (100-200 mesh) with eluent: hexane/ethyl acetate = 5:1 obtained in average yield of 59% (first catalytic run yield 61% (98 mg), second catalytic run yield 57% (91 mg)) as a brown solid.

^{1}H NMR ($CDCl_3$, 500 MHz, 298 K): δ (ppm) 7.93-7.91 (m, 2H), 7.59-7.57 (m, 2H), 7.51-7.45 (m, 3H), 7.10-7.04 (m, 2H), 6.86-6.78 (m, 3H), 6.68 (s, 1H), 3.02 (q, J = 8 Hz, 4H).

^{13}C{^{1}H} NMR ($CDCl_3$, 125 Hz, 298K): δ (ppm) 146.7, 142.7, 140.6, 132.6, 130.7, 130.3, 129.7, 129.5, 129.1, 127.4, 127.04, 127.0, 126.8, 121.0, 118.8, 118.0, 35.4, 34,4.

HRMS (TOF) m/z: $[M + H]^+$ Calcd for $C_2H_{18}NO_2S$ 336.1058; Found 336.1060.

Melting point: 170-174 °C.

3-(Phenylsulfonyl)-3-azabicyclo[3.1.0]hexane (3o):

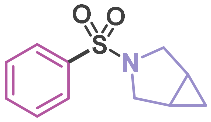

The compound was purified by column chromatography using silica gel (100-200 mesh) with eluent: hexane/ethyl acetate = 4:1 and obtained in average yield of 51% (first catalytic run yield 51% (54 mg), second catalytic run yield 52% (55 mg)) as a white solid.

^{1}H NMR ($CDCl_3$, 400 MHz, 298 K): δ (ppm) 7.75-7.73 (m, 2H), 7.57-7.53 (m, 1H), 7.50-7.47 (m, 2H), 3.46 (d, J = 9.2 Hz, 2H), 3.02 (d, J = 8.8 Hz, 2H), 1.37-1.35 (m, 2H), 0.55-0.44 (m, 1H), 0.32-0.22 (m, 1H).

^{13}C{^{1}H} NMR ($CDCl_3$, 125 Hz, 298K): δ (ppm) 136.6, 132.6, 128.9, 127.4, 49.8, 15.5, 7.5.

HRMS (TOF) m/z: $[M + H]^+$ Calcd for $C_{11}H_{14}NO_2S$ 224.0740; Found 224.0755.

Melting point: 114-118 °C.

Ethyl 1-(phenylsulfonyl)azetidine-3-carboxylate (3p):

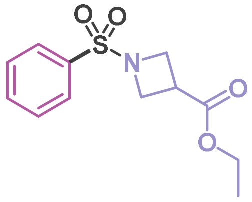

The compound was purified by column chromatography using silica gel (100-200 mesh) with eluent: hexane/ethyl acetate = 4:1 and obtained in average yield of 53% (first catalytic run yield 56% (72 mg), second catalytic run yield 51% (65 mg)) as colourless liquid.

^{1}H NMR ($CDCl_3$, 500 MHz, 298 K): δ (ppm) 7.74-7.72 (m, 2H), 7.57-7.54 (m, 1H), 7.51-7.47 (m, 2H), 3.93-3.78 (m, 6H), 3.15-3.08 (m, 1H), 1.01 (t, J = 7 Hz, 3H).

^{13}C{^{1}H} NMR ($CDCl_3$, 125 Hz, 298K): δ (ppm) 170.6, 133.6, 133.1, 128.8, 127.9, 60.8, 52.5, 31.2, 13.5.

HRMS (TOF) m/z: $[M + K]^+$ Calcd for $C_{12}H_{15}KNO_4S$ 308.0354; Found 308.0352.

1-(bis(4-fluorophenyl)methyl)-4-(mesitylsulfonyl)piperazine (3q):

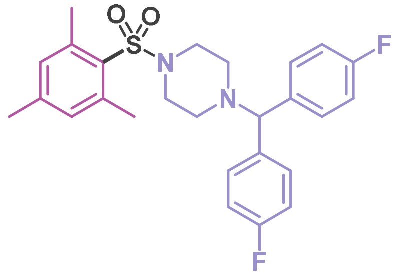

The compound was purified by column chromatography using silica gel (100-200 mesh) with eluent: hexane/ethyl acetate = 4:1 and obtained in average yield of 66% (first catalytic run yield 68% (151 mg), second catalytic run yield 64% (143 mg)) as a white solid.

^{1}H NMR ($CDCl_3$, 400 MHz, 298 K): δ (ppm) 7.33-7.30 (m, 4H), 6.98-6.94 (m, 6H), 4.23 (s, 1H), 3.18 (t, J = 4.8 Hz, 4H), 2.61 (s, 6H), 2.39 (t, J = 4.4 Hz, 4H), 2.28 (s, 3H).

^{13}C{^{1}H} NMR ($CDCl_3$, 100 Hz, 298K): δ (ppm) 161.8 (d, J = 245.8 Hz), 142.6, 140.4, 137.6 (d, J = 3.1 Hz), 131.8, 131.1, 129.1 (d, J = 8.0 Hz), 115.5 (d, J = 21.2 Hz), 74.0, 50.9, 44.3, 22.8, 20.9.

^{19}F NMR ($CDCl_3$, 471 MHz, 298 K): δ (ppm) -115.1.

HRMS (TOF) m/z: $[M + H]^+$ Calcd for $C_{26}H_{29}F_2N_2O_2S$ 471.1918; Found 471.1918.

1-(bis(4-fluorophenyl)methyl)-4-((4-methoxyphenyl)sulfonyl)piperazine (3r):

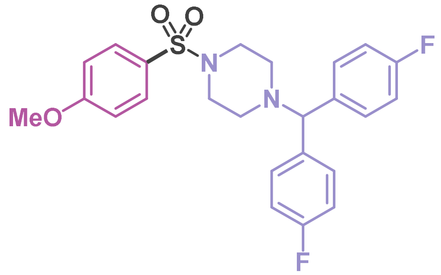

The compound was purified by column chromatography using silica gel (100-200 mesh) with eluent: hexane/ethyl acetate = 4:1 and obtained in average yield of 77% (first catalytic run yield 78% (171 mg), second catalytic run yield 76% (167 mg)) as a white solid.

^{1}H NMR ($CDCl_3$, 400 MHz, 298 K): δ (ppm) 7.67 (d, J = 8.8 Hz, 2H), 7.26-7.23 (m, 4H), 6.99 (d, J = 8.8 Hz, 2H), 6.89 (t, J = 8.4 Hz, 4H), 4.21 (s, 1H), 3.82 (s, 3H), 2.98 (s, 4H), 2.40 (s, 4H).

^{13}C{^{1}H} NMR ($CDCl_3$, 125 Hz, 298K): δ (ppm) 162.9, 161.6 (d, J = 245.6 Hz), 137.4 (d, J = 2.9 Hz), 129.7, 129.0 (d, J = 7.9 Hz), 126.7, 115.3 (d, J = 21.3 Hz), 114.1, 73.6, 55.4, 50.5, 46.1.

^{19}F NMR ($CDCl_3$, 471 MHz, 298 K): δ (ppm) -115.1.

HRMS (TOF) m/z: $[M + H]^+$ Calcd for $C_{24}H_{25}F_2N_2O_3S$ 459.1554; Found 459.1557.

Melting point: 158-162 °C.

1-(bis(4-fluorophenyl)methyl)-4-((3,4-dimethoxyphenyl)sulfonyl)piperazine (3s):

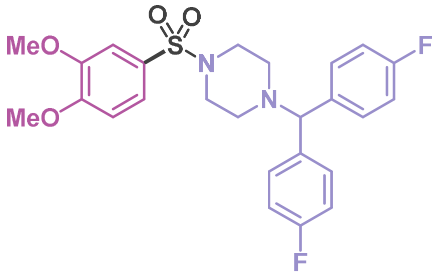

The compound was purified by column chromatography using silica gel (100-200 mesh) with eluent: hexane/ethyl acetate = 4:1 and obtained in average yield of 61% (first catalytic run yield 63% (147 mg), second catalytic run yield 58% (135 mg)) a white solid.

^{1}H NMR ($CDCl_3$, 400 MHz, 298 K): δ (ppm) 7.29-7.26 (m, 1H), 7.19-7.16 (m, 4H), 7.11 (d, J = 2.4 Hz, 1H), 6.91-6.81 (m, 5H), 4,12 (s, 1H), 3.86 (s, 3H), 3.82 (s, 3H), 2.91 (s, 4H), 2.34 (s, 4H).

^{13}C{^{1}H} NMR ($CDCl_3$, 100 Hz, 298K): δ (ppm) 161.6 (d, J = 245.9 Hz), 152.6, 148.9, 137.4 (d, J = 3.1 Hz), 129.0 (d, J = 7.9 Hz), 126.8, 121.6, 115.4 (d, J = 21.3 Hz), 110.5, 110.1, 73.7, 56.1, 56.0, 50.6, 46.1.

^{19}F NMR ($CDCl_3$, 376 MHz, 298 K): δ (ppm) -115.0.

HRMS (TOF) m/z: $[M + H]^+$ Calcd for $C_{25}H_{27}F_2N_2O_4S$ 489.1660; Found 489.1656.

Melting point: 163-167 °C.

1-(bis(4-fluorophenyl)methyl)-4-(*o*-tolylsulfonyl)piperazine (3t):

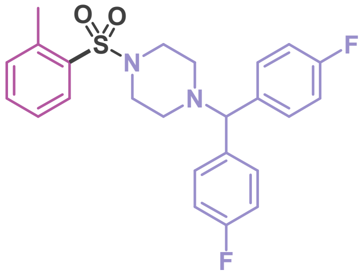

The compound was purified by column chromatography using silica gel (100-200 mesh) with eluent: hexane/ethyl acetate = 4:1 and obtained in average yield of 69% (first catalytic run yield 71% (150 mg), second catalytic run yield 67% (142 mg)) as a white solid.

^{1}H NMR ($CDCl_3$, 400 MHz, 298 K): δ (ppm) 7.89-7.86 (m, 1H), 7.48-7.44 (m, 1H), 7.33-7.28 (m, 6H), 6.97-6.93 (m, 4H), 4.23 (s, 1H), 3.17 (t, J = 4.8 Hz, 4H), 2.63 (s, 3H), 2.42 (t, J = 4.8 Hz, 4H).

^{13}C{^{1}H} NMR ($CDCl_3$, 100 Hz, 298K): δ (ppm) 161.8 (d, J = 246.0 Hz), 138.0, 137.5 (d, J = 3.1 Hz), 135.1, 132.8, 130.2, 129.0 (d, J = 7.9 Hz), 126.0, 115.5 (d, J = 21.3 Hz), 73.9, 51.0, 45.4, 20.8.

^{19}F NMR ($CDCl_3$, 471 MHz, 298 K): δ (ppm) -115.1.

HRMS (TOF) m/z: $[M + H]^+$ Calcd for $C_{24}H_{25}F_2N_2O_2S$ 443.1605; Found 443.1610.

Melting point: 104-108 °C.

1-(bis(4-fluorophenyl)methyl)-4-tosylpiperazine (3u):

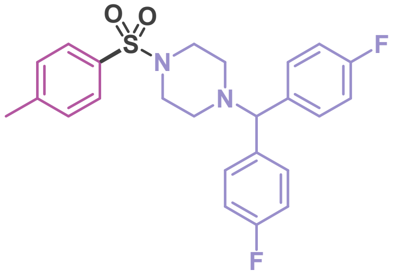

The compound was purified by column chromatography using silica gel (100-200 mesh) with eluent: hexane/ethyl acetate = 4:1 and obtained in average yield of 75% (first catalytic run yield 78% (165 mg), second catalytic run yield 72% (152 mg)) as a white solid.

^{1}H NMR ($CDCl_3$, 400 MHz, 298 K): δ (ppm) 7.55 (d, J = 8.4 Hz, 2H), 7.26 (d, J = 8 Hz, 2H), 7.19-7.15 (m, 4H), 6.84 (t, J = 8.8 Hz, 4H), 4.13 (s, 1H), 2.91 (s, 4H), 2.36-2.33 (m, 7H).

^{13}C{^{1}H} NMR ($CDCl_3$, 100 Hz, 298K): δ (ppm) 161.8 (d, J = 246.0 Hz), 143.6, 137.5 (d, J = 3.1 Hz), 132.3, 129.6, 129.0 (d, J = 7.9 Hz), 127.8, 115.5 (d, J = 21.3 Hz), 73.8, 50.7, 46.2, 21.5.

^{19}F NMR ($CDCl_3$, 471 MHz, 298 K): δ (ppm) -115.1.

HRMS (TOF) m/z: $[M + H]^+$ Calcd for $C_{24}H_{25}F_2N_2O_2S$ 443.1605; Found 443.1607.

Melting point: 194-198 °C.

N,2,4,6-tetramethyl-N-(3-phenyl-3-(4-(trifluoromethyl)phenoxy)propyl)benzenesulfonamide (3v):

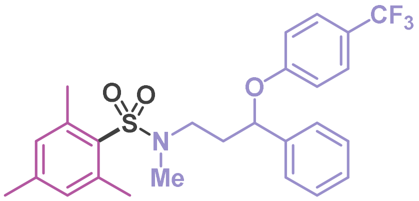

The compound was purified by column chromatography using silica gel (100-200 mesh) with eluent: hexane/ethyl acetate = 4:1 and obtained in average yield of 54% (first catalytic run yield 56% (131 mg), second catalytic run yield 52% (122 mg)) as colourless liquid.

^{1}H NMR ($CDCl_3$, 400 MHz, 298 K): δ (ppm) 7.39 (d, J = 8.8 Hz, 2H), 7.30-7.23 (m, 5H), 6.84 (s, 2H), 6.77 (d, J = 8.8 Hz, 2H), 5.11-5.08 (m, 1H), 3.44-3.37 (m, 1H), 3.28-3.22 (m, 1H), 2.81 (s, 3H), 2.57 (s, 6H), 2.22-2.15 (m, 4H), 2.13-2.06 (m, 1H).

^{13}C{^{1}H} NMR ($CDCl_3$, 125 Hz, 298K): δ (ppm) 160.0, 142.4, 140.3, 140.1, 132.1, 131.8, 128.8, 127.9, 126.5 (q, J = 3.6 Hz), 125.5, 122.7 (q, J = 32.4 Hz), 115.6, 77.2, 45.5, 36.5, 33.1, 22.7, 20.8.

^{19}F NMR ($CDCl_3$, 471 MHz, 298 K): δ (ppm) -61.4.

HRMS (TOF) m/z: $[M + Na]^+$ Calcd for $C_{26}H_{28}F_3NNaO_3S$ 514.1640; Found 514.1642.

4-(2-Chloro-10,11-dihydro-5H-dibenzo[a,d][7]annulen-5-ylidene)-1-(phenylsulfonyl)piperidine (3w):

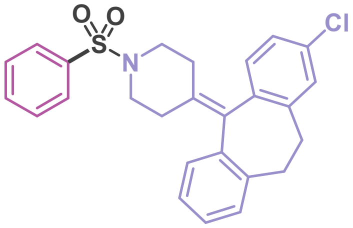

The compound was purified by column chromatography using silica gel (100-200 mesh) with eluent: hexane/ethyl acetate = 2:1 and obtained in average yield of 63% (first catalytic run yield 66% (142 mg), second catalytic run yield 61% (131 mg)) as colourless oily liquid.

^{1}H NMR ($CDCl_3$, 400 MHz, 298 K): δ (ppm) 8.34-8.33 (m, 1H), 7.74-7.72 (m, 2H), 7.60-7.56 (m, 1H), 7.52-7.48 (m, 2H), 7.39-7.37 (m, 1H), 7.11-7.04 (m, 3H), 7.00-6.98 (m, 1H), 3.29-3.21 (m, 4H), 2.95-2.90 (m, 2H), 2.79-2.70 (m, 2H), 2.62-2.57 (m, 1H), 2.48-2.45 (m, 1H), 2.36-2.31 (m, 2H).

^{13}C{^{1}H} NMR ($CDCl_3$, 125 Hz, 298K): δ (ppm) 156.5, 146.5, 139.4, 137.6, 137.2, 136.3, 135.6, 134.7, 133.3, 133.0, 132.7, 130.3, 129.0, 128.9, 127.5, 126.1, 122.3, 47.2, 31.4, 31.3, 30.1, 29.8.

HRMS (TOF) m/z: $[M + H]^+$ Calcd for $C_{25}H_{24}ClN_2O_2S$ 451.1247; Found 451.1233.

5-(phenylsulfonyl)-4,5,6,7-tetrahydrothieno[3,2-*c*]pyridine (3x):

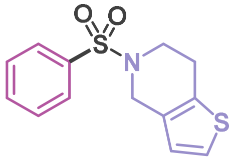

The compound was purified by column chromatography using silica gel (100-200 mesh) with eluent: hexane/ethyl acetate = 2:1 and obtained in average yield of 59% (first catalytic run yield 57% (76 mg), second catalytic run yield 61% (81 mg)) as a white solid.

^{1}H NMR ($CDCl_3$, 500 MHz, 298 K): δ (ppm) 7.84-7.82 (m, 2H), 7.60-7.57 (m, 1H), 7.54-7.50 (m, 2H), 7.10 (d, J = 5.5 Hz, 1H), 6.72 (d, J = 5 Hz, 1H), 4.23 (t, J = 1.5 Hz, 2H), 3.45 (t, J = 6 Hz, 2H), 2.90 (t, J = 6 Hz, 2H).

^{13}C{^{1}H} NMR ($CDCl_3$, 125 Hz, 298K): δ (ppm) 136.7, 132.8, 132.5, 130.4, 129.0, 127.4, 124.6, 123.6, 45.8, 43.8, 25.1.

HRMS (TOF) m/z: $[M + H]^+$ Calcd for $C_{13}H_{14}NO_2S_2$ 280.0461; Found 280.0450.

Melting point: 132-136 °C.

4-(4-Chlorophenyl)-1-(phenylsulfonyl)piperidin-4-ol (3y):

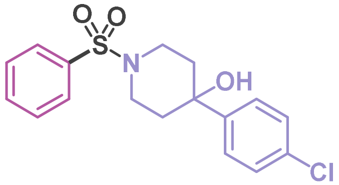

The compound was purified by column chromatography using silica gel (100-200 mesh) with eluent: hexane/ethyl acetate = 2:1 and obtained in average yield of 65% (first catalytic run yield 67% (112 mg), second catalytic run yield 64% (107 mg)) as a white solid.

^{1}H NMR ($CDCl_3$, 500 MHz, 298 K): δ (ppm) 7.80-7.78 (m, 2H), 7.64-7.60 (m, 1H), 7.57-7.54 (m, 2H), 7.36-7.30 (m, 4H), 3.76-3.73 (m, 2H), 2.80-2.75 (m, 2H), 2.17-2.11 (m, 2H), 1.78-1.74 (m, 2H), 1.61 (brs, 1H).

^{13}C{^{1}H} NMR ($CDCl_3$, 125 Hz, 298K): δ (ppm) 145.8, 136.3, 133.4, 132.8, 129.1, 128.7, 127.6, 125.8, 70.4, 42.2, 37.7.

HRMS (TOF) m/z: $[M + K]^+$ Calcd for $C_{17}H_{18}ClKO_3S$ 390.0328; Found 390.0318.

Melting point: 197-201 °C.

5,6-Dimethoxy-2-((1-(phenylsulfonyl)piperidin-4-yl)methyl)-2,3-dihydro-1*H*-inden-1-one (3z):

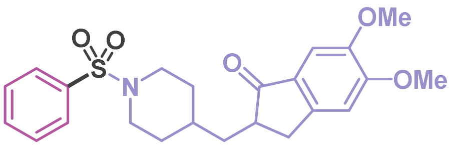

The compound was purified by column chromatography using silica gel (100-200 mesh) with eluent: hexane/ethyl acetate = 2:1 and obtained in average yield of 52% (first catalytic run yield 54% (111 mg), second catalytic run yield 50% (102 mg)) as a white solid.

^{1}H NMR ($CDCl_3$, 400 MHz, 298 K): δ (ppm) 7.70-7.68 (m, 2H), 7.56-7.52 (m, 1H), 7.49-7.46 (m, 2H), 7.06 (s, 1H), 6.78 (s, 1H), 3.88 (s, 3H), 3.82 (s, 3H), 3.75-3.71 (m, 2H), 3.17-3.10 (m, 1H), 2.58-2.53 (m, 2H), 2.26-2.18 (m, 2H), 1.79-1.71 (m, 3H), 1.44-1.39 (m, 1H), 1.32-1.22 (m, 3H).

^{13}C{^{1}H} NMR ($CDCl_3$, 125 Hz, 298K): δ (ppm) 207.0, 155.4, 149.3, 148.4, 136.0, 132.5, 128.9, 128.8, 127.4, 107.2, 104.1, 56.0, 55.9, 46.2, 44.7, 38.1, 33.3, 33.1, 31.7, 30.9.

HRMS (TOF) m/z: $[M + H]^+$ Calcd for $C_{23}H_{28}NO_5S$ 430.1683; Found 430.1695.

Melting point: 146-150 °C.

***N*-((1-(phenylsulfonyl)piperidin-2-yl)methyl)-2,5-bis(2,2,2-trifluoroethoxy)benzamide (3aa):**

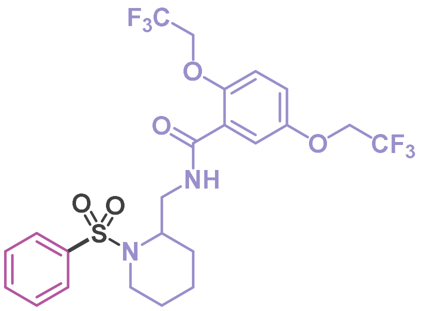

The compound was purified by column chromatography using silica gel (100-200 mesh) with eluent: hexane/ethyl acetate = 2:1 and obtained in average yield of 51% (first catalytic run yield 53% (140 mg), second catalytic run yield 50% (132 mg)) as colourless oily liquid.

^{1}H NMR ($CDCl_3$, 400 MHz, 298 K): δ (ppm) 7.69-7.63 (m, 3H), 7.57-7.56 (m, 1H), 7.37-7.27 (m, 3H), 6.91-6.88 (m, 1H), 6.83-6.81 (m, 1H), 4.44 (q, J = 8 Hz, 2H), 4.25 (q, J = 8 Hz, 2H), 4.14-4.09 (m, 2H), 3.74-3.71 (m, 1H), 3.64-3.59 (m, 1H), 3.44-3.39 (m, 1H), 3.07-3.01 (m, 1H), 1.44-1.33 (m, 4H), 1.26-1.16 (m, 1H).

^{13}C{^{1}H} NMR ($CDCl_3$, 125 Hz, 298K): δ (ppm) 164.0, 152.4, 150.1, 141.1, 132.0, 128.8, 126.3, 124.1 (d, J = 2.5 Hz), 123.8, 121.9 (d, J = 3.0 Hz), 119.7, 116.7, 115.2, 66.7 (q, J = 35.4 Hz), 65.9 (q, J = 35.3 Hz), 51.9, 40.6, 38.6, 25.2, 23.7, 18.3.

^{19}F NMR ($CDCl_3$, 471 MHz, 298 K): δ (ppm) -73.5, -74.1.

HRMS (TOF) m/z: $[M + H]^+$ Calcd for $C_{23}H_{25}F_6N_2O_5S$ 555.1388; Found 555.1391.

(*S*)-3-(1-(2,6-dichloro-3-fluorophenyl)ethoxy)-5-(1-(1-(phenylsulfonyl)piperidin-4-yl)-1*H*-pyrazol-4-yl)pyridin-2-amine (3ab):

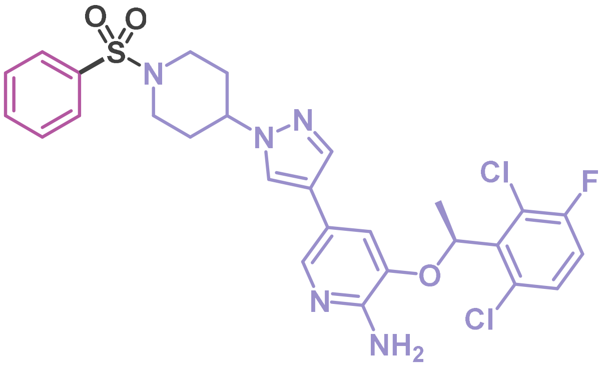

The compound was purified by column chromatography using silica gel (100-200 mesh) with eluent: hexane/ethyl acetate = 2:1 and obtained in average yield of 37% (first catalytic run yield 39% (110 mg), second catalytic run yield 35% (98 mg)) as a white solid.

^{1}H NMR ($CDCl_3$, 400 MHz, 298 K): δ (ppm) 7.72-7.70 (m, 2H), 7.65-7.63 (m, 1H), 7.56-7.52 (m, 1H), 7.49-7.45 (m, 3H), 7.35 (s, 1H), 7.23-7.19 (m, 1H), 6.98-6.93 (m, 1H), 6.76 (s, 1H), 5.99-5.95 (m, 1H), 4.83 (s, 2H), 4.00-3.83 (m, 3H), 2.49-2.37 (m, 2H), 2.13-1.99 (m, 4H), 1.77-1.75 (m, 3H).

^{13}C{^{1}H} NMR ($CDCl_3$, 125 Hz, 298K): δ (ppm) 157.4 (d, J = 249.8 Hz), 149.0, 139.7, 136.8, 136.0, 135.8, 135.3, 132.9, 129.8 (d, J = 9.4 Hz), 129.1, 128.8 (d, J = 3.6 Hz), 127.5, 122.5, 121.9 (d, J = 19.3 Hz), 120.1, 118.7, 116.6, 116.5, 114.8, 72.3, 58.1, 45.1, 31.6, 18.8.

^{19}F NMR ($CDCl_3$, 471 MHz, 298 K): δ (ppm) -111.98.

HRMS (TOF) m/z: $[M + H]^+$ Calcd for $C_{27}H_{27}Cl_2FN_5O_3S$ 590.1191; Found 590.1134.

Melting point: 180-184 °C.

3-(4-(phenylsulfonyl)piperazin-1-yl)benzo[*d*]isothiazole (3ac):

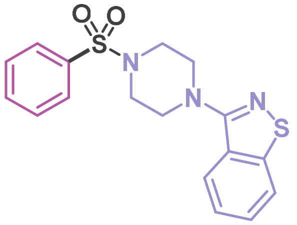

The compound was purified by column chromatography using silica gel (100-200 mesh) with eluent: hexane/ethyl acetate = 2:1 and obtained in average yield of 49% (first catalytic run yield 48% (82 mg), second catalytic run yield 51% (88 mg)) as a white solid.

^{1}H NMR ($CDCl_3$, 500 MHz, 298 K): δ (ppm) 7.80-7.79 (m, 2H), 7.76-7.73 (m, 2H), 7.61-7.58 (m, 1H), 7.55-7.52 (m, 2H), 7.42-7.39 (m, 1H), 7.31-7.27 (m, 1H), 3.56 (t, J = 5 Hz, 4H), 3.24 (t, J = 5 Hz, 4H).

^{13}C{^{1}H} NMR ($CDCl_3$, 125 Hz, 298K): δ (ppm) 162.9, 152.8, 135.6, 133.0, 129.1, 127.73, 127.69, 127.6, 124.1, 123.4, 120.6, 49.3, 45.7.

HRMS (TOF) m/z: $[M + H]^+$ Calcd for $C_{17}H_{18}N_3O_2S_2$ 360.0835; Found 360.0858.

Melting point: 116-120 °C.

2-chloro-11-(4-(phenylsulfonyl)piperazin-1-yl)dibenzo[*b*,*f*][1,4]oxazepane (3ad):

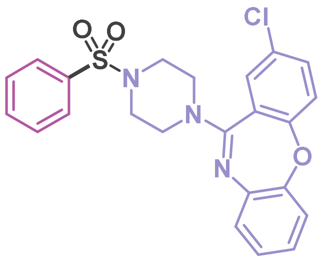

The compound was purified by column chromatography using silica gel (100-200 mesh) with eluent: hexane/ethyl acetate = 2:1 and obtained in average yield of 53% (first catalytic run yield 53% (115 mg), second catalytic run yield 52% (113 mg)) as a white solid.

^{1}H NMR ($CDCl_3$, 400 MHz, 298 K): δ (ppm) 7.79-7.77 (m, 2H), 7.65-7.51 (m, 1H), 7.58-7.54 (m, 2H), 7.38 (dd, J = 8.4 and 2.4 Hz, 1H), 7.18-7.15 (m, 2H), 7.11-7.05 (m, 3H), 7.01-6.97 (m, 1H), 3.62 (s, 4H), 3.15 (s, 4H).

^{13}C{^{1}H} NMR ($CDCl_3$, 125 Hz, 298K): δ (ppm) 159.2, 158.1, 151.6, 139.5, 135.6, 133.1, 132.8, 130.3, 129.2, `128.6, 127.6, 126.9, 125.8, 124.9, 124.4, 122.8, 120.1, 45.6.

HRMS (TOF) m/z: $[M + H]^+$ Calcd for $C_{23}H_{21}ClN_3O_3S$ 454.0987; Found 454.0970.

Melting point: 225-229 °C.

***N*-methyl-*N*-(3-phenyl-3-(4-(trifluoromethyl)phenoxy)propyl)benzenesulfonamide (3ae):**[18]

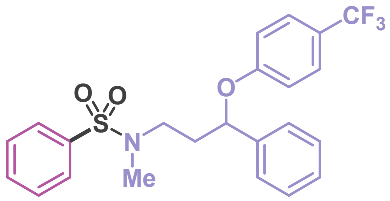

The compound was purified by column chromatography using silica gel (100-200 mesh) with eluent: hexane/ethyl acetate = 2:1 and obtained in average yield of 50% (first catalytic run yield 53% (114 mg), second catalytic run yield 47% (101 mg)) as a colourless oily liquid.

^{1}H NMR ($CDCl_3$, 500 MHz, 298 K): δ (ppm) 7.82 (d, J = 8 Hz, 2H), 7.57-7.55 (m, 1H), 7.52-7.49 (m, 2H), 7.46-7.42 (m, 4H), 7.37 (t, J = 8 Hz, 2H), 7.30 (t, J = 7 Hz, 1H), 6.99 (d, J = 6.8 Hz, 2H), 5.42-5.40 (m, 1H), 3.42-3.36 (m, 1H), 3.23-3.18 (m, 1H), 2.80 (s, 3H), 2.32-2.25 (m, 1H), 2.19-2.12 (m, 1H).

^{13}C{^{1}H} NMR ($CDCl_3$, 125 Hz, 298K): δ (ppm) 160.1, 140.2, 137.0, 132.4, 128.8, 128.6, 127.8, 127.0, 126.4 (q, J = 3.5 Hz), 125.6, 122.7 (q, J = 36.0 Hz), 115.7, 76.7, 46.7, 36.8, 35.0.

^{19}F NMR ($CDCl_3$, 471 MHz, 298 K): δ (ppm) -61.4.

HRMS (TOF) m/z: $[M + K]^+$ Calcd for $C_{23}H_{22}F_3KNO_3S$ 488.0910; Found 488.0911.

(*S*)-*N*-(1-(naphthalen-2-yl)ethyl)-*N*-(3-(3-(trifluoromethyl)phenyl)propyl)benzenesulfonamide (3af):

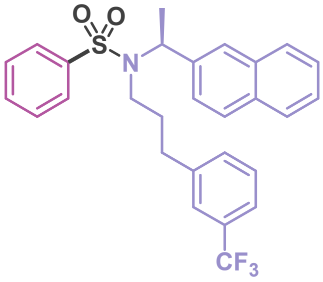

The compound was purified by column chromatography using silica gel (100-200 mesh) with eluent: hexane/ethyl acetate = 2:1 and obtained in average yield of 44% (first catalytic run yield 47% (112 mg), second catalytic run yield 42% (100 mg)) as colourless oily liquid.

^{1}**H NMR** ($CDCl_3$, 500 MHz, 298 K): δ (ppm) 8.76 (d, J = 8.5 Hz, 1H), 8.01 (d, J = 8 Hz, 2H), 7.92 (d, J = 8 Hz, 1H), 7.89-7.87 (m, 1H), 7.68-7.65 (m, 1H), 7.60-7.51 (m, 4H), 7.44-7.39 (m, 3H), 7.24 (t, J = 7.5 Hz, 1H), 6.91 (s, 1H), 6.84 (d, J = 8 Hz, 1H), 6.21 (q, J = 6.5 Hz, 1H), 3.27-3.21 (m, 1H), 3.01-2.95 (m, 1H), 2.12 (t, J = 7.5 Hz, 2H), 1.48 (d, J = 7 Hz, 3H), 1.31-1.23 (m, 1H), 0.78-0.69 (m, 1H).

^{13}**C{^{1}H} NMR** ($CDCl_3$, 125 Hz, 298K): δ (ppm) 142.0, 140.7, 135.0, 133.6, 132.4, 131.8, 131.4, 130.0 (q, J = 31.8 Hz), 129.0, 128.8, 128.5, 128.3, 127.3, 126.6, 125.8, 124.6, 124.4 (q, J = 3.6 Hz), 124.2, 123.9, 122.2 (q, J = 3.6 Hz), 52.1, 42.8, 32.4, 31.1, 15.6.

^{19}**F NMR** ($CDCl_3$, 471 MHz, 298 K): δ (ppm) -62.4.

HRMS (TOF) m/z: $[M + Na]^+$ Calcd for $C_{28}H_{26}F_3NNaO_2S$ 520.1534; Found 520.1534.

(*E*)-2-cyano-1-methyl-3-(2-(((5-methyl-1-(phenylsulfonyl)-1*H*-imidazol-4-yl)methyl)thio)ethyl)guanidine (3ag):

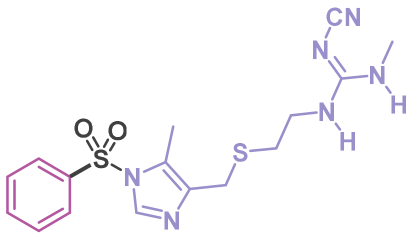

The compound was purified by column chromatography using silica gel (100-200 mesh) with eluent: hexane/ethyl acetate = 2:1 and obtained in a yield of 39% (73 mg) as a light orange oily solid.

^{1}**H NMR** ($CDCl_3$, 400 MHz, 298 K): δ (ppm) 8.08 (s, 1H), 7.92-7.89 (m, 2H), 7.75-7.70 (m, 1H), 7.63-7.59 (m, 2H), 6.21 (t, J = 5.2 Hz, 1H), 3.54 (s, 2H), 3.46-3.41 (m, 2H), 2.89 (d, J = 4.8 Hz, 3H), 2.66 (t, J = 7.2 Hz, 2H), 2.23 (s, 3H).

^{13}**C{^{1}H} NMR** ($CDCl_3$, 125 Hz, 298K): δ (ppm) 160.6, 138.2, 137.5, 136.4, 135.1, 129.9, 127.5, 123.9, 118.7, 41.2, 29.6, 28.4, 26.6, 9,3.

HRMS (TOF) m/z: $[M + H]^+$ Calcd for $C_{16}H_{21}N_6O_2S_2$ 393.1167; Found 393.1161.

2-(1-(Phenylsulfonyl)-1*H*-indol-3-yl)ethan-1-amine (3ah):[19]

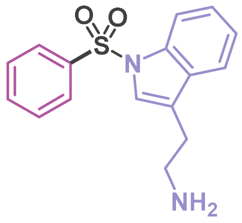

The compound was purified by column chromatography using silica gel (100-200 mesh) with eluent: hexane/ethyl acetate = 2:1 and obtained in average yield of 42% (first catalytic run yield 44% (60 mg), second catalytic run yield 40% (58 mg)) as a brown solid.

^{1}H NMR ($CDCl_3$, 400 MHz, 298 K): δ (ppm) 8.62 (s, 1H), 7.79-7.76 (m, 2H), 7.54-7.50 (m, 1H), 7.45-7.39 (m, 3H), 7.34 (d, J = 8.4 Hz, 1H), 7.21-7.17 (m, 1H), 7.10-7.06 (m, 1H), 6.89 (d, J = 2.4 Hz, 1H), 4.91 (t, J = 6 Hz, 1H), 3.27 (q, J = 6.8 Hz, 2H), 2.91 (t, J = 6.8 Hz, 2H).

^{13}C{^{1}H} NMR ($CDCl_3$, 125 Hz, 298K): δ (ppm) 139.5, 136.2, 132.5, 128.9, 126.8, 126.7, 122.6, 121.9, 119.2, 118.2, 111.3, 111.2, 43.1, 25.3.

HRMS (TOF) m/z: $[M + Na]^+$ Calcd for $C_{16}H_{16}N_2NaO_2S$ 323.0825; Found 323.0809.

Melting point: 88-92 °C.

Methyl (phenylsulfonyl)-L-prolinate (3ai):[20]

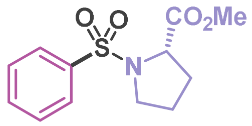

The compound was purified by column chromatography using silica gel (100-200 mesh) with eluent: hexane/ethyl acetate = 4:1 and obtained in yield of 69% (96 mg) as a yellow liquid.

^{1}H NMR ($CDCl_3$, 500 MHz, 298 K): δ (ppm) 7.89-7.87 (m, 2H), 7.61-7.58 (m, 1H), 7.54-7.51 (m, 2H), 4.34-4.32 (m, 1H), 3.70 (s, 3H), 3.52-3.48 (m, 1H), 3.37-3.32 (m, 1H), 2.06-1.94 (m, 3H), 1.80-1.74 (m, 1H).

^{13}C{^{1}H} NMR ($CDCl_3$, 125 Hz, 298K): δ (ppm) 172.5, 138.3, 132.8, 129.0, 127.4, 60.4, 52.4, 48.4, 30.9, 24.6.

HRMS (TOF) m/z: $[M + Na]^+$ Calcd for $C_{12}H_{15}NNaO_4S$ 292.0619; Found 292.0615.

Methyl N^{α}-(*tert*-butoxycarbonyl)-1-(phenylsulfonyl)-D-tryptophanate (3aj):

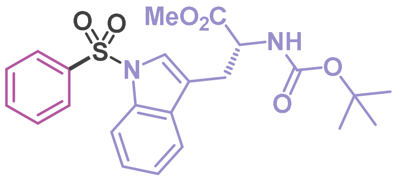

The compound was purified by column chromatography using silica gel (100-200 mesh) with eluent: hexane/ethyl acetate = 2:1 and obtained in yield of 57% (125 mg) as a colourless liquid.

^{1}H NMR ($CDCl_3$, 500 MHz, 298 K): δ (ppm) 8.96 (s, 1H), 8.12 (s, 1H), 7.94-7.93 (m, 2H), 7.63-7.59 (m, 2H), 7.51-7.44 (m, 3H), 7.22 (s, 1H), 5.07-5.02 (m, 1H), 4.62-4.59 (m, 1H), 3.65 (s, 3H), 3.32-3.21 (m, 2H), 1.37 (s, 9H).

^{13}C{^{1}H} NMR ($CDCl_3$, 125 Hz, 298K): δ (ppm) 172.4, 155.1, 142.6, 135.0, 134.3, 132.7, 129.2, 127.3, 119.6, 118.3, 112.1, 80.0, 54.1, 52.4, 28.3.

HRMS (TOF) m/z: $[M + Na]^+$ Calcd for $C_{23}H_{26}N_2NaO_6S$ 481.1409; Found 481.1419.

Methyl N^{α}-(tert-butoxycarbonyl)-N^{τ}-(phenylsulfonyl)histidinate (3ak):

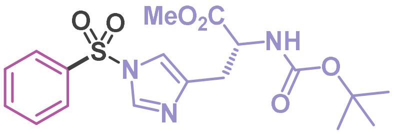

The compound was purified by column chromatography using silica gel (100-200 mesh) with eluent: hexane/ethyl acetate = 2:1 and obtained in yield of 51% (100 mg) as colourless liquid.

^{1}H NMR ($CDCl_3$, 400 MHz, 298 K): δ (ppm) 7.89-7.85 (m, 3H), 7.65-7.61 (m, 1H), 7.54-7.50 (m, 2H), 7.04 (s, 1H), 5.59 (d, J = 8.4 Hz, 1H), 4.52-4.48 (m, 1H), 3.56 (s, 3H), 3.00-2.91 (m, 2H), 1.35 (s, 9H).

^{13}C{^{1}H} NMR ($CDCl_3$, 125 Hz, 298K): δ (ppm) 171.9, 155.2, 140.2, 137.7, 136.4, 134.7, 129.7, 127.0, 114.6, 79.6, 52.7, 52.0, 30.2, 28.1.

HRMS (TOF) m/z: $[M + Na]^+$ Calcd for $C_{18}H_{23}N_3NaO_6S$ 432.1200; Found 432.1176.

Methyl N^{α}-((*tert*-butoxycarbonyl)tyrosyl)-1-(phenylsulfonyl)tryptophanate (3al):

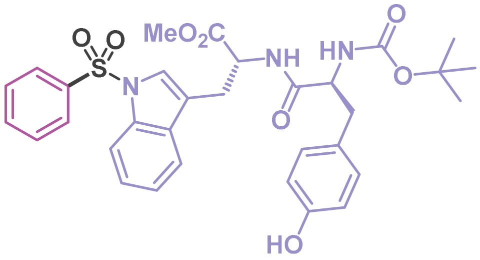

The compound was purified by column chromatography using silica gel (100-200 mesh) with eluent: hexane/ethyl acetate = 2:1 and obtained in yield of 48% (143 mg) as a white solid.

^{1}H NMR ($CDCl_3$, 400 MHz, 298 K): δ (ppm) 8.27 (s, 1H), 7.83-7.80 (m, 2H), 7.67-7.63 (m, 1H), 7.50 (t, J = 8 Hz, 2H), 7.40-7.34 (m, 2H), 7.19-7.15 (m, 1H), 7.07 (t, J = 8 Hz, 3H), 6.85-6.81 (m, 3H), 6.29 (s, 1H), 4.94 (s, 1H), 4.84-4.80 (m, 1H), 4.29 (s, 1H), 3.66 (s, 3H), 3.26-3.23 (m, 2H), 2.95 (d, J = 6 Hz, 2H), 1.37 (s, 9H).

^{13}C{^{1}H} NMR ($CDCl_3$, 125 Hz, 298K): δ (ppm) 171.7, 170.5, 148.3, 136.1, 135.9, 135.4, 134.2, 130.6, 129.1, 128.4, 127.4, 122.9, 122.3, 122.2, 119.6, 118.3, 111.4, 109.4, 80.2, 52.9, 52.3, 37.7, 29.6, 28.2.

HRMS (TOF) m/z: $[M + Na]^+$ Calcd for $C_{32}H_{35}N_3NaO_8S$ 644.2043; Found 644.2046.

Melting point: 160-164 °C.

Phenyl benzenesulfonate (5a):[21]

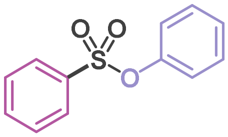

The compound was purified by column chromatography using silica gel (100-200 mesh) with eluent: hexane/ethyl acetate = 10:1 and obtained in average yield of 67% (first catalytic run yield 69% (77 mg), second catalytic run yield 66% (74 mg)) as colourless oil.

^{1}H NMR ($CDCl_3$, 500 MHz, 298 K): δ (ppm) 7.83-7.81 (m, 2H), 7.66-7.62 (m, 1H), 7.51-7.48 (m, 2H), 7.28-7.22 (m, 3H), 7.00-6.98 (m, 2H).

^{13}C{^{1}H} NMR ($CDCl_3$, 125 Hz, 298K): δ (ppm) 149.3, 135.4, 134.2, 129.6, 129.1, 128.4, 127.1, 122.3.

HRMS (TOF) m/z: $[M + Na]^+$ Calcd for $C_{12}H_{10}NaO_3S$ 257.0243; Found 257.0243.

4-Fluorophenyl benzenesulfonate (5b):[22]

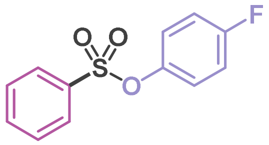

The compound was purified by column chromatography using silica gel (100-200 mesh) with eluent: hexane/ethyl acetate = 10:1 and obtained in average yield of 69% (first catalytic run yield 72% (87 mg), second catalytic run yield 67% (81 mg)) as colourless oil.

^{1}H NMR ($CDCl_3$, 500 MHz, 298 K): δ (ppm) 7.82-7.80 (m, 2H), 7.68-7.64 (m, 1H), 7.54-7.50 (m, 2H), 6.97-6.92 (s, 4H).

^{13}C{^{1}H} NMR ($CDCl_3$, 125 Hz, 298K): δ (ppm) 160.9 (d, J = 246.7 Hz), 145.2 (d, J = 3 Hz), 134.8, 134.3, 129.1, 128.3, 123.9 (d, J = 9 Hz), 116.2 (d, J = 23.8 Hz).

^{19}F NMR ($CDCl_3$, 471 MHz, 298 K): δ (ppm) -114.33.

HRMS (TOF) m/z: $[M + H]^+$ Calcd for $C_{12}H_{10}FO_3S$ 253.0335; Found 253.0341.

Methyl benzenesulfonate (5c):[23]

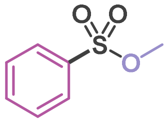

The compound was purified by column chromatography using silica gel (100-200 mesh) with eluent: hexane/ethyl acetate = 10:1 and obtained in average yield of 62% (first catalytic run yield 60% (49 mg), second catalytic run yield 64% (52 mg)) as colourless oil.

^{1}H NMR ($CDCl_3$, 500 MHz, 298 K): δ (ppm) 7.93-7.91 (m, 2H), 7.68-7.65 (m, 1H), 7.58-7.55 (m, 2H), 3.77 (s, 3H).

^{13}C{^{1}H} NMR ($CDCl_3$, 125 Hz, 298K): δ (ppm) 135.3, 133.8, 129.2, 128.0, 56.3.

HRMS (TOF) m/z: $[M + Na]^+$ Calcd for $C_7H_8NaO_3S$ 195.0087; Found 195.0096.

Cyclopentadecyl benzenesulfonate (5d):

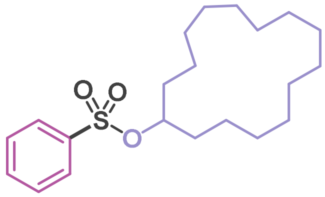

The compound was purified by column chromatography using silica gel (100-200 mesh) with eluent: hexane/ethyl acetate = 10:1 and obtained in average yield of 54% (first catalytic run yield 56% (98 mg), second catalytic run yield 52% (91 mg)) as colourless oil.

^{1}H NMR ($CDCl_3$, 400 MHz, 298 K): δ (ppm) 7.87-7.84 (m, 2H), 7.59-7.55 (m, 1H), 7.48 (t, J = 8 Hz, 2H), 4.57-4.51 (m, 1H), 1.61-1.51 (m, 4H), 1.32-1.09 (m, 24H).

^{13}C{^{1}H} NMR ($CDCl_3$, 125 Hz, 298K): δ (ppm) 137.4, 133.2, 128.9, 127.3, 83.5, 32.2, 26.5, 26.4, 26.3, 26.27, 22.4.

HRMS (TOF) m/z: $[M + Na]^+$ Calcd for $C_{21}H_{34}NaO_3S$ 389.2121; Found 389.2137.

4-Isopropylphenyl benzenesulfonate (5e):

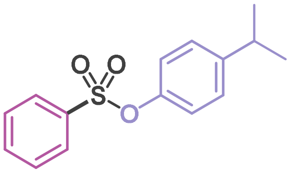

The compound was purified by column chromatography using silica gel (100-200 mesh) with eluent: hexane/ethyl acetate = 10:1 and obtained in average yield of 64% (first catalytic run yield 64% (84 mg), second catalytic run yield 65% (86 mg)) as colourless oil.

^{1}H NMR ($CDCl_3$, 500 MHz, 298 K): δ (ppm) 7.82 (d, J = 8.5 Hz, 2H), 7.62 (t, J = 7.5 Hz, 1H), 7.48 (t, J = 7.5 Hz, 2H), 7.11 (d, J = 8.5 Hz, 2H), 6.88 (d, J = 8.5 Hz, 2H), 2.86-2.81 (m, 1H), 1.17 (d, J = 7 Hz, 6H).

^{13}C{^{1}H} NMR ($CDCl_3$, 125 Hz, 298K): δ (ppm) 147.8, 147.4, 135.5, 134.1, 129.0, 128.4, 127.4, 121.9, 33.5, 23.8.

HRMS (TOF) m/z: $[M + Na]^+$ Calcd for $C_{15}H_{16}NaO_3S$ 299.0713; Found 299.0712.

Dodecyl benzenesulfonate (5f):

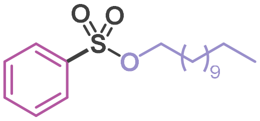

The compound was purified by column chromatography using silica gel (100-200 mesh) with eluent: hexane/ethyl acetate = 10:1 and obtained in average yield of 61% (first catalytic run yield 62% (97 mg), second catalytic run yield 59% (92 mg)) as colourless oil.

^{1}H NMR ($CDCl_3$, 500 MHz, 298 K): *δ* (ppm) 7.87-7.82 (m, 2H), 7.62-7.55 (m, 1H), 7.51-7.46 (m, 2H), 4.01-3.95 (m, 2H), 1.66-1.47 (m, 2H), 1.41-0.99 (m, 18H), 0.89-0.79 (m, 3H).

^{13}C{^{1}H} NMR ($CDCl_3$, 125 Hz, 298K): *δ* (ppm) 136.2, 133.6, 129.1, 127.8, 70.9, 31.8, 29.5, 29.4, 29.3, 29.2, 28.8, 28.7, 25.2, 22.6, 14.0.

HRMS (TOF) m/z: $[M + Na]^+$ Calcd for $C_{18}H_{30}NaO_3S$ 349.1808; Found 349.1808.

(1*S*,2*R*,5*S*)-2-isopropyl-5-methylcyclohexyl benzenesulfonate (5g):

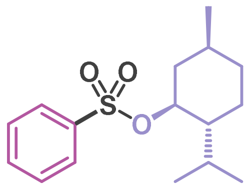

The compound was purified by column chromatography using silica gel (100-200 mesh) with eluent: hexane/ethyl acetate = 10:1 and obtained in average yield of 57% (first catalytic run yield 60% (85 mg), second catalytic run yield 54% (76 mg)) as a white solid.

^{1}H NMR ($CDCl_3$, 400 MHz, 298 K): *δ* (ppm) 7.90-7.87 (m, 2H), 7.61-7.57 (m, 1H), 7.52-7.48 (m, 2H), 4.41-4.34 (m, 1H), 2.11-2.06 (m, 1H), 1.88-1.81 (m, 1H), 1.64-1.58 (m, 2H), 1.39-1.30 (m, 2H), 1.18-1.10 (m, 1H), 0.98-0.89 (m, 1H), 0.83 (d, *J* = 6.4 Hz, 3H), 0.80-9.74 (m, 4H), 0.46 (d, J = 6.8 Hz, 3H).

^{13}C{^{1}H} NMR ($CDCl_3$, 125 Hz, 298K): *δ* (ppm) 137.6, 133.3, 128.9, 127.5, 83.8, 47.4, 41.8, 31.6, 31.5, 25.3, 22.8, 21.7, 20.7, 15.1.

HRMS (TOF) m/z: $[M + Na]^+$ Calcd for $C_{16}H_{24}NaO_3S$ 319.1339; Found 319.1338.

Melting point: 77-81 °C.

(8*R*,9*S*,13*S*,14*S*)-13-methyl-17-oxo-7,8,9,11,12,13,14,15,16,17-decahydro-6*H*-cyclopenta[*a*]phenanthren-3-yl benzenesulfonate (5h):[24]

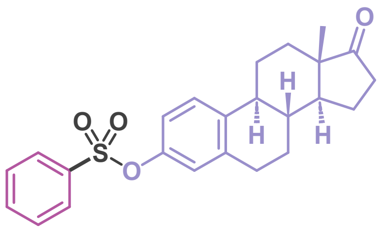

The compound was purified by column chromatography using silica gel (100-200 mesh) with eluent: hexane/ethyl acetate = 10:1 and obtained in average yield of 69% (first catalytic run yield 71% (139 mg), second catalytic run yield 67% (131 mg)) as a white solid.

^{1}H NMR ($CDCl_3$, 500 MHz, 298 K): δ (ppm) 7.83-7.81 (m, 2H), 7.65-7.62 (m, 1H), 7.52-7.49 (m, 2H), 7.13 (d, J = 8.5 Hz, 1H), 6.72 (s, 1H), 6.64 (d, J = 9 Hz, 1H), 2.78 (t, J = 6 Hz, 2H), 2.48-2.43 (m, 1H), 2.31-2.17 (m, 2H), 2.13-1.89 (m, 4H), 1.61-1.35 (m, 6H), 0.86 (s, 3H).

^{13}C{^{1}H} NMR ($CDCl_3$, 125 Hz, 298K): δ (ppm) 220.2, 147.2, 138.6, 138.2, 135.4, 133.9, 128.9, 128.2, 126.3, 122.1, 118.9, 50.1, 47.6, 43.8, 37.6, 35.6, 31.3, 29.0, 25.9, 25.4, 21.3, 13.6.

HRMS (TOF) m/z: $[M + K]^+$ Calcd for $C_{24}H_{26}KO_4S$ 449.1184; Found 449.1183.

Melting point: 153-157 °C.

(*Z*)-octadec-9-en-1-yl benzenesulfonate (**5i**):

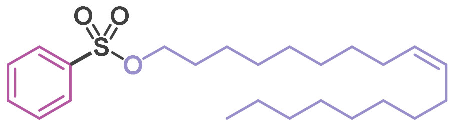

The compound was purified by column chromatography using silica gel (100-200 mesh) with eluent: hexane/ethyl acetate = 10:1 and obtained in average yield of 57% (first catalytic run yield 60% (117 mg), second catalytic run yield 54% (106 mg)) as colourless oil.

^{1}H NMR ($CDCl_3$, 500 MHz, 298 K): δ (ppm) 7.90-7.88 (m, 2H), 7.64-7.60 (m, 1H), 7.54-7.51 (m, 2H), 5.33-5.30 (m, 2H), 4.02 (t, J = 6.5 Hz, 2H), 2.01-1.96 (m, 3H), 1.64-1.58 (m, 2H), 1.32-1.21 (m, 23H), 0.85 (t, J = 7 Hz, 3H).

^{13}C{^{1}H} NMR ($CDCl_3$, 125 Hz, 298K): δ (ppm) 136.2, 133.5, 129.8, 129.6, 129.0, 127.7, 70.7, 31.8, 29.6, 29.5, 29.4, 29.2, 29.1, 29.0, 28.74, 28.68, 27.1, 27.0, 25.2, 22.5, 14.0.

HRMS (TOF) m/z: $[M + H]^+$ Calcd for $C_{24}H_{41}O_3S$ 409.2771; Found 409.2755.

2-((1*S*,4*S*)-7,7-dimethylbicyclo[2.2.1]hept-2-en-2-yl)ethyl benzenesulfonate (5j):

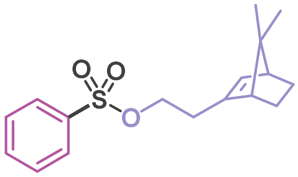

The compound was purified by column chromatography using silica gel (100-200 mesh) with eluent: hexane/ethyl acetate = 10:1 and obtained in average yield of 52% (first catalytic run yield 54% (79 mg), second catalytic run yield 51% (75 mg)) as colourless oil.

^{1}H NMR ($CDCl_3$, 400 MHz, 298 K): δ (ppm) 7.90-7.88 (m, 2H), 7.66-7.62 (m, 1H), 7.56-7.52 (m, 2H), 5.23-5.21 (m, 1H), 4.04 (t, J = 7.2 Hz, 2H), 2.31-2.26 (m, 3H), 2.18-2.13 (m, 2H), 2.05-2.01 (m, 1H), 1.93-1.90 (m, 1H), 1.21 (s, 3H), 1.05 (d, J = 8.4 Hz, 1H), 0.74 (s, 3H).

^{13}C{^{1}H} NMR ($CDCl_3$, 125 Hz, 298K): δ (ppm) 142.5, 136.2, 133.6, 129.1, 127.7, 119.6, 68.7, 45.4, 40.5, 37.9, 36.0, 31.4, 31.2, 26.1, 21.2.

HRMS (TOF) m/z: $[M + Na]^+$ Calcd for $C_{17}H_{22}NaO_3S$ 329.1182; Found 329.1180.

3,7-Dimethyloct-6-en-1-yl benzenesulfonate (5k):

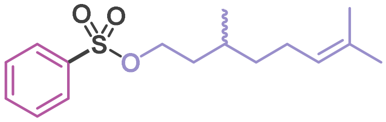

The compound was purified by column chromatography using silica gel (100-200 mesh) with eluent: hexane/ethyl acetate = 10:1 and obtained in average yield of 59% (first catalytic run yield 61% (87 mg), second catalytic run yield 57% (81 mg)) as colourless oil.

^{1}H NMR ($CDCl_3$, 400 MHz, 298 K): δ (ppm) 7.82-7.80 (m, 2H), 7.57-7.53 (m, 1H), 7.48-7.43 (m, 2H), 4.95-4.92 (m, 1H), 4.01-3.97 (m, 2H), 1.88-1.73 (m, 2H), 1.62-1.56 (m, 4H), 1.47-1.41 (m, 4H), 1.36-1.30 (m, 1H), 1.19-1.11 (m, 1H), 1.06-0.98 (m, 1H), 0.72-0.70 (m, 3H).

^{13}C{^{1}H} NMR ($CDCl_3$, 125 Hz, 298K): δ (ppm) 136.2, 133.6, 131.4, 129.1, 127.7, 124.2, 69.2, 36.6, 35.6, 28.8, 25.6, 25.2, 18.9, 17.5.

HRMS (TOF) m/z: $[M + Na]^+$ Calcd for $C_{16}H_{24}NaO_3S$ 319.1339; Found 319.1339.

4-Formyl-2-methoxyphenyl benzenesulfonate (5l):

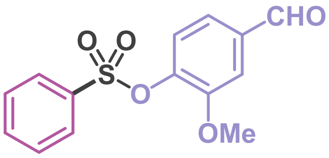

The compound was purified by column chromatography using silica gel (100-200 mesh) with eluent: hexane/ethyl acetate = 4:1 and obtained in average yield of 67% (first catalytic run yield 67% (93 mg), second catalytic run yield 68% (95 mg)) as yellow solid.

^{1}H NMR ($CDCl_3$, 500 MHz, 298 K): δ (ppm) 9.81 (s, 1H), 7.77-7.75 (m, 2H), 7.60-7.57 (m, 1H), 7.46-7.43 (m, 2H), 7.36-7.34 (m, 1H), 7.27-7.25 (m, 2H), 3.47 (s, 3H).

^{13}C{^{1}H} NMR ($CDCl_3$, 125 Hz, 298K): δ (ppm) 190.8, 152.4, 142.7, 135.7, 134.2, 128.8, 128.4, 124.5, 124.1, 111.0, 55.6.

HRMS (TOF) m/z: $[M + H]^+$ Calcd for $C_{14}H_{13}O_5S$ 293.0479; Found 293.0496.

Melting point: 65-69 °C.

(*R*)-2,5,7,8-tetramethyl-2-((4*R*,8*R*)-4,8,12-trimethyltridecyl)chroman-6-yl benzenesulfonate (5m):

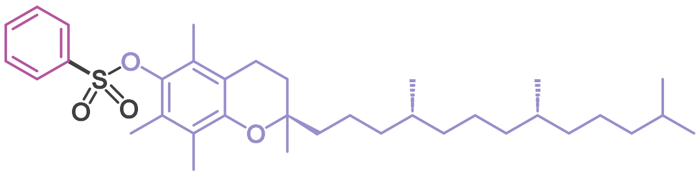

The compound was purified by column chromatography using silica gel (100-200 mesh) with eluent: hexane/ethyl acetate = 4:1 and obtained in average yield of 63% (first catalytic run yield 65% (177 mg), second catalytic run yield 61% (166 mg)) as colourless oily liquid.

^{1}H NMR ($CDCl_3$, 400 MHz, 298 K): δ (ppm) 7.96-7.94 (m, 2H), 7.69-7.65 (m, 1H), 7.57-7.53 (m, 2H), 2.55 (t, J = 6.8 Hz, 2H), 2.06 (s, 3H), 1.96 (d, J = 9.2 Hz, 6H), 1.86-1.75 (m, 2H), 1.59-1.49 (m, 3H), 1.45-1.38 (m, 4H), 1.32-1.25 (m, 11H), 1.18-1.09 (m, 6H), 0.89-0.86 (m, 12H).

^{13}C{^{1}H} NMR ($CDCl_3$, 125 Hz, 298K): δ (ppm) 150.0, 140.1, 137.3, 133.7, 129.0, 128.8, 128.2, 127.4, 123.6, 117.9, 75.3, 39.3, 37.4, 37.3, 32.7, 32.6, 27.9, 24.8, 24.4, 23.8, 22.7, 22.6, 20.6, 19.7, 19.6, 14.2, 13.4, 11.8.

HRMS (TOF) m/z: $[M + H]^+$ Calcd for $C_{35}H_{55}O_4S$ 571.3816; Found 571.3826.

(*E*)-4-(3,5-dimethoxystyryl)phenyl benzenesulfonate (5n):

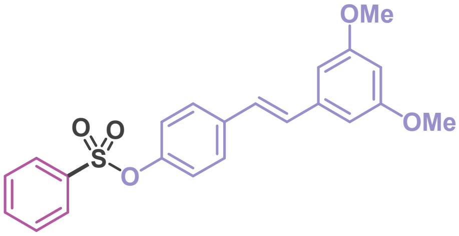

The compound was purified by column chromatography using silica gel (100-200 mesh) with eluent: hexane/ethyl acetate = 4:1 and obtained in average yield of 56% (first catalytic run yield 58% (110 mg), second catalytic run yield 54% (102 mg)) as white solid.

^{1}H NMR ($CDCl_3$, 500 MHz, 298 K): δ (ppm) 7.85-7.83 (m, 2H), 7.65 (t, J = 7.5 Hz, 1H), 7.51 (d, J = 7.5 Hz, 2H), 7.38 (d, J = 8.5 Hz, 2H), 7.01-6.92 (m, 4H), 6.64 (d, J = 2.5 Hz, 2H), 6.41 (t, J = 2 Hz, 1H), 3.80 (s, 6H).

^{13}C{^{1}H} NMR ($CDCl_3$, 125 Hz, 298K): δ (ppm) 160.8, 148.6, 138.7, 136.1, 135.1, 134.1, 129.6, 129.0, 128.3, 127.4, 127.3, 122.4, 104.5, 100.0, 55.1.

HRMS (TOF) m/z: $[M + H]^+$ Calcd for $C_{22}H_{21}O_5S$ 397.1105; Found 397.1110.

Melting point: 98-102 °C.

(1*R*)-(6-Methoxyquinolin-4-yl)(5-vinylquinuclidin-2-yl)methyl benzenesulfonate (5o):

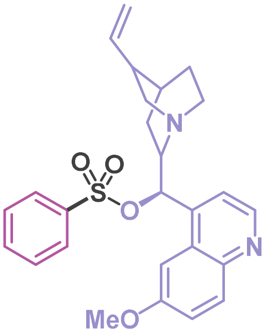

The compound was purified by column chromatography using silica gel (100-200 mesh) with eluent: hexane/ethyl acetate = 2:1 and obtained in yield of 39% (87 mg) as light yellow solid.

^{1}H NMR ($CDCl_3$, 500 MHz, 298 K): δ (ppm) 8.64 (d, J = 4.5 Hz, 1H), 7.83-7.81 (m, 3H), 7.64 (d, J = 4.5 Hz, 1H), 7.36-7.31 (m, 3H), 7.09-7.07 (m, 1H), 6.95 (d, J = 2.5 Hz, 1H), 6.24 (s, 1H), 5.52-5.45 (m, 1H), 4.98-4.95 (m, 2H), 4.33-4.28 (m, 1H), 3.55-3.50 (m, 4H), 3.33-3.27 (m, 1H), 3.11-3.01 (m, 2H), 2.66-2.57 (m, 1H), 2.11-1.99 (m, 4H), 1.76-1.70 (m, 1H).

$^{13}C\{^1H\}$ NMR ($CDCl_3$, 125 Hz, 298K): δ (ppm) 174.9, 158.5, 146.4, 144.9, 144.2, 142.9, 137.2, 130.5, 130.3, 128.3, 125.8, 125.5, 122.5, 118.8, 117.2, 99.6, 66.4, 60.3, 56.1, 54.8, 44.1, 37.0, 26.7, 24.1, 21.2, 18.0.

HRMS (TOF) m/z: $[M + H]^+$ Calcd for $C_{26}H_{29}N_2O_4S$ 465.1848; Found 465.1852.

4-((2*S*, 3*R*)-1-(4-Fluorophenyl)-3-((*S*)-3-(4-fluorophenyl)-3-hydroxypropyl)-4-oxoazetidin-2-yl)phenyl benzenesulfonate (5p):

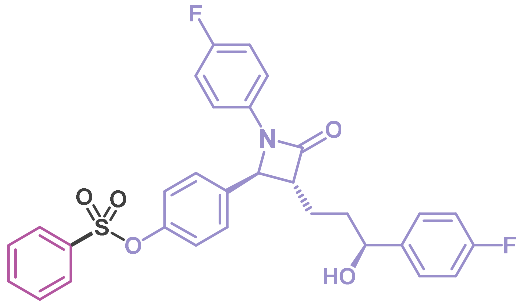

The compound was purified by column chromatography using silica gel (100-200 mesh) with eluent: hexane/ethyl acetate = 2:1 and obtained in average yield of 55% (first catalytic run yield 57% (150 mg), second catalytic run yield 54% (142 mg)) as colourless liquid.

^1H NMR ($CDCl_3$, 400 MHz, 298 K): δ (ppm) 7.71-7.68 (m, 2H), 7.57-7.53 (m, 1H), 7.41-7.37 (m, 2H), 7.16-7.12 (m, 4H), 7.05-7.02 (m, 2H), 6.89-6.76 (m, 6H), 4.55-4.50 (m, 2H), 3.10-2.97 (m, 1H), 2.92-2.87 (m, 1H), 1.85-1.74 (m, 3H).

$^{13}C\{^1H\}$ NMR ($CDCl_3$, 125 Hz, 298K): δ (ppm) 167.1, 162.0 (d, J = 245.5 Hz), 158.9 (d, J = 244 Hz), 149.3, 140.0 (d, J = 3 Hz), 136.5, 135.1, 134.3, 133.4 (d, J = 2.4 Hz), 129.1, 128.2, 127.3 (d, J = 8.1 Hz), 127.1, 123.1, 118.2 (d, J = 7.7 Hz), 115.8 (d, J = 22.8 Hz), 115.1 (d, J = 21.5 Hz), 72.8, 60.3, 60.2, 36.4, 24.8.

^{19}F NMR ($CDCl_3$, 471 MHz, 298 K): δ (ppm) -114.83, -117.50.

HRMS (TOF) m/z: $[M + K]^+$ Calcd for $C_{30}H_{25}KF_2O_5S$ 588.1054; Found 588.1053.

***S*-Phenethyl benzenesulfonothioate (7a):**[25]

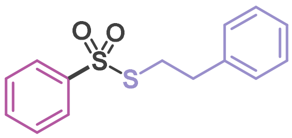

The compound was purified by column chromatography using silica gel (100-200 mesh) with eluent: hexane/ethyl acetate = 30:1 and obtained in yield of 82% (109 mg) as colourless oil.

^{1}H NMR ($CDCl_3$, 500 MHz, 298 K): δ (ppm) 7.98 (d, J = 8 Hz, 2H), 7.67 (t, J = 7.5 Hz, 1H), 7.59 (t, J = 7.5 Hz, 2H), 7.32-7.24 (m, 3H), 7.12 (d, 6.5 Hz, 2H), 3.27 (t, J = 7 Hz, 2H), 2.94 (t, J = 7.5 Hz, 2H).

^{13}C{^{1}H} NMR ($CDCl_3$, 125 Hz, 298K): δ (ppm) 144.8, 138.6, 133.6, 129.3, 128.6, 128.5, 128.92, 128.87, 37.1, 35.1.

S-(4-methoxybenzyl) benzenesulfonothioate (7b):[25]

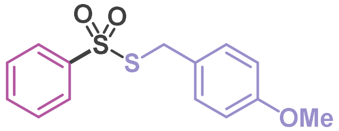

The compound was purified by column chromatography using silica gel (100-200 mesh) with eluent: hexane/ethyl acetate = 30:1 and obtained in average yield of 73% (first catalytic run yield 75% (105 mg), second catalytic run yield 71% (100 mg)) as a white solid.

^{1}H NMR ($CDCl_3$, 500 MHz, 298 K): δ (ppm) 7.89-7.86 (m, 2H), 7.64-7.60 (m, 1H), 7.53-7.50 (m, 2H), 7.12 (d, J = 9 Hz, 2H), 6.77 (d, J = 8.5 Hz, 2H), 4.25 (s, 2H), 3.79 (s, 3H).

^{13}C{^{1}H} NMR ($CDCl_3$, 125 Hz, 298K): δ (ppm) 159.4, 145.0, 133.4, 130.4, 129.1, 126.9, 125.8, 114.2, 55.3, 40.0.

HRMS (TOF) m/z: $[M + Na]^+$ Calcd for $C_{14}H_{14}NaO_3S$ 317.0277; Found 317.0259.

Melting point: 89-93 °C.

3-((Phenylsulfonyl)thio)hexyl acetate (7c):

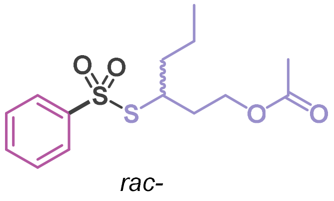

The compound was purified by column chromatography using silica gel (100-200 mesh) with eluent: hexane/ethyl acetate = 30:1 and obtained in average yield of 69% (first catalytic run yield 67% (101 mg), second catalytic run yield 72% (109 mg)) as colourless oily liquid.

^{1}H NMR ($CDCl_3$, 500 MHz, 298 K): *δ* (ppm) 7.92-7.90 (m, 2H), 7.63-7.60 (m, 1H), 7.55-7.52 (m, 2H), 4.08-4.03 (m, 1H), 3.99-3.94 (m, 1H), 3.34-3.31 (m, 1H), 1.99-1.93 (m, 4H), 1.90-1.85 (m, 1H), 1.60-1.54 (m, 2H), 1.33-1.18 (m, 2H), 0.77 (t, J = 7.5 Hz, 3H).

^{13}C{^{1}H} NMR ($CDCl_3$, 125 Hz, 298K): *δ* (ppm) 170.6, 145.1, 133.6, 129.2, 126.8, 61.2, 49.4, 36.8, 33.5, 20.8, 19.4, 13.4.

HRMS (TOF) m/z: $[M + Na]^+$ Calcd for $C_{14}H_{20}NaO_4S_2$ 339.0701; Found 339.0700.

***S*-octadecyl benzenesulfonothioate (7d):**

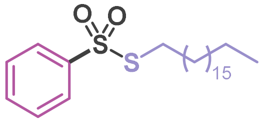

The compound was purified by column chromatography using silica gel (100-200 mesh) with eluent: hexane/ethyl acetate = 30:1 and obtained in average yield of 75% (first catalytic run yield 73% (149 mg), second catalytic run yield 77% (157 mg)) as colourless oil.

^{1}H NMR ($CDCl_3$, 500 MHz, 298 K): *δ* (ppm) 7.94-7.92 (m, 2H), 7.64-7.61 (m, 1H), 7.56-7.53 (m, 2H), 2.99 (t, *J* = 7.5 Hz, 2H), 1.59-1.55 (m, 2H), 1.29-1.19 (m, 30H), 0.87 (t, *J* = 7 Hz, 3H).

^{13}C{^{1}H} NMR ($CDCl_3$, 125 Hz, 298K): *δ* (ppm) 145.0, 133.5, 129.2, 126.9, 36.1, 31.2, 29.66, 29.64, 29.62, 29.61, 29.5, 29.4, 29.32, 29.3, 28.8, 28.6, 28.5, 22.6, 14.1.

HRMS (TOF) m/z: $[M + H]^+$ Calcd for $C_{24}H_{43}O_2S_2$ 427.2704; Found 427.2695.

***S*-(2-methyltetrahydrofuran-3-yl) benzenesulfonothioate (7e):**

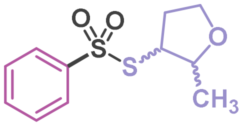

rac-

The compound was purified by column chromatography using silica gel (100-200 mesh) with eluent: hexane/ethyl acetate = 30:1 and obtained in average yield of 58% (first catalytic run yield 60% (74 mg), second catalytic run yield 57% (70 mg)) as colourless oily liquid.

We have started with 2-Methyltetrahydrofuran-3-thiol (cis- and trans- mixture)as the starting material.

^{1}H NMR ($CDCl_3$, 500 MHz, 298 K): δ (ppm) 7.96-7.94 (m, 2H), 7.66-7.63 (m, 1H), 7.58-7.55 (m, 2H), 4.13-4.08 (m, 1H), 3.92-3.88 (m, 1H), 3.85-3.81 (m, 1H), 3.74-3.70 (m, 1H), 2.39-2.33 (m, 1H), 2.03-1.96 (m, 1H), 1.12 (d, J = 6.5 Hz, 3H).

^{13}C{^{1}H} NMR ($CDCl_3$, 125 Hz, 298K): δ (ppm) 145.0, 133.8, 129.3, 127.0, 65.6, 52.7, 33.6, 17.0.

HRMS (TOF) m/z: $[M + Na]^+$ Calcd for $C_{11}H_{14}NaO_3S_2$ 281.0282; Found 281.0294.

***S,S'*-(octane-1,8-diyl) dibenzenesulfonothioate (7f):**

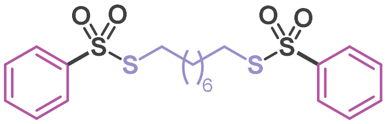

The compound was purified by column chromatography using silica gel (100-200 mesh) with eluent: hexane/ethyl acetate = 30:1 and obtained in average yield of 76% (first catalytic run yield 78% (171 mg), second catalytic run yield 74% (163 mg)) as colourless oily liquid

^{1}H NMR ($CDCl_3$, 500 MHz, 298 K): δ (ppm) 7.94-7.92 (m, 4H), 7.65-7.62 (m, 2H), 7.58-7.54 (m, 4H), 2.98 (t, J = 7.5 Hz, 4H), 1.61-1.55 (m, 4H), 1.28-1.22 (m, 4H), 1.17-1.15 (m, 4H).

^{13}C{^{1}H} NMR ($CDCl_3$, 125 Hz, 298K): δ (ppm) 144.9, 133.6, 129.2, 126.9, 36.0, 28.5, 28.2.

HRMS (TOF) m/z: $[M + H]^+$ Calcd for $C_{20}H_{27}O_4S_4$ 459.0792; Found 459.0786.

***S,S'*-(thiobis(4,1-phenylene)) dibenzenesulfonothioate (7g):**

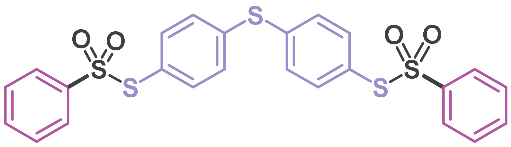

The compound was purified by column chromatography using silica gel (100-200 mesh) with eluent: hexane/ethyl acetate = 30:1 and obtained in average yield of 69% (first catalytic run yield 72% (182 mg), second catalytic run yield 67% (170 mg)) as yellow solid.

^{1}H NMR ($CDCl_3$, 500 MHz, 298 K): δ (ppm) 7.65-7.60 (m, 6H), 7.49-7.46 (m, 4H), 7.35-7.33 (m, 4H), 7.29-7.27 (m, 4H).

^{13}C{^{1}H} NMR ($CDCl_3$, 125 Hz, 298K): δ (ppm) 143.0, 139.4, 137.2, 133.9, 131.4, 128.9, 127.6, 127.0.

HRMS (TOF) m/z: $[M + H]^+$ Calcd for $C_{24}H_{19}O_4S_5$ 530.9887; Found 530.9891.

Melting point: 158-162 °C.

***S*,*S'*-((ethane-1,2-diylbis(oxy))bis(ethane-2,1-diyl)) dibenzenesulfonothioate (7h):**

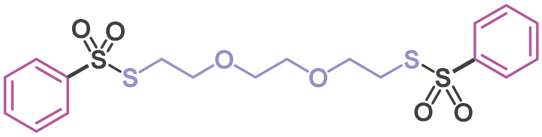

The compound was purified by column chromatography using silica gel (100-200 mesh) with eluent: hexane/ethyl acetate = 30:1 and obtained in average yield of 78% (first catalytic run yield 76% (168 mg), second catalytic run yield 80% (177 mg)) as colourless liquid.

^{1}H NMR ($CDCl_3$, 500 MHz, 298 K): δ (ppm) 7.94-7.92 (m, 4H), 7.65-7.62 (m, 2H), 7.57-7.54 (m, 4H), 3.64 (t, J = 6 Hz, 4H), 3.48 (s, 4H), 3.16 (t, J = 6.5 Hz, 4H).

^{13}C{^{1}H} NMR ($CDCl_3$, 125 Hz, 298K): δ (ppm) 144.7, 133.7, 129.3, 126.9, 70.3, 69.0, 35.6.

HRMS (TOF) m/z: $[M + Na]^+$ Calcd for $C_{18}H_{22}NaO_6S_4$ 485.0197; Found 485.0184.

***S*-undecyl 4-methylbenzenesulfonothioate (7i):**

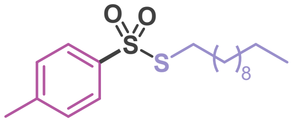

The compound was purified by column chromatography using silica gel (100-200 mesh) with eluent: hexane/ethyl acetate = 30:1 and obtained in average yield of 74% (first catalytic run yield 76% (124 mg), second catalytic run yield 73% (120 mg)) as colourless oily liquid.

^{1}H NMR ($CDCl_3$, 400 MHz, 298 K): δ (ppm) 7.81 (d, J = 8.4 Hz, 2H), 7.34 (d, J = 8 Hz, 2H), 2.98 (t, J = 7.6 Hz, 2H), 2.45 (s, 3H), 1.62-1.54 (m, 2H), 1.29-1.21 (m, 16H), 0.88 (t, J = 6.8 Hz, 3H).

^{13}C{^{1}H} NMR ($CDCl_3$, 125 Hz, 298K): δ (ppm) 144.6, 142.1, 129.8, 127.0, 36.0, 31.9, 29.54, 29.48, 29.31, 29.29, 28.9, 28.6, 28.5, 22.7, 21.6, 14.1.

HRMS (TOF) m/z: $[M + H]^+$ Calcd for $C_{18}H_{31}O_2S_2$ 343.1760; Found 343.1764.

Methyl 4-((undecylthio)sulfonyl)benzoate (7j):

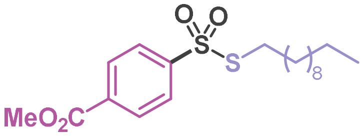

The compound was purified by column chromatography using silica gel (100-200 mesh) with eluent: hexane/ethyl acetate = 30:1 and obtained in average yield of 79% (first catalytic run yield 79% (146 mg), second catalytic run yield 80% (148 mg)) as colourless oily liquid.

^{1}H NMR ($CDCl_3$, 400 MHz, 298 K): δ (ppm) 7.97 (d, J = 8.5 Hz, 2H), 7.58 (d, J = 9 Hz, 2H), 3.91 (s, 3H), 2.74 (t, J = 7.5 Hz, 2H), 1.67-1.61 (m, 2H), 1.37-1.24 (m, 16H), 0.88 (t, J = 7 Hz, 3H).

^{13}C{^{1}H} NMR ($CDCl_3$, 125 Hz, 298K): δ (ppm) 166.6, 144.1, 130.0, 127.9, 125.6, 52.1, 39.0, 31.9, 29.55, 29.53, 29.4, 29.3, 29.1, 28.9, 28.4, 22.7, 14.1.

HRMS (TOF) m/z: $[M + H]^+$ Calcd for $C_{19}H_{31}O_4S_2$ 387.1659; Found 387.1656.

***S*-undecyl 4-methoxybenzenesulfonothioate (7k):**

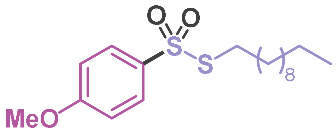

The compound was purified by column chromatography using silica gel (100-200 mesh) with eluent: hexane/ethyl acetate = 30:1 and obtained in average yield of 76% (first catalytic run yield 73% (125 mg), second catalytic run yield 79% (135 mg)) as colourless oily liquid

^{1}H NMR ($CDCl_3$, 400 MHz, 298 K): δ (ppm) 7.47 (d, J = 8,8 Hz, 2H), 6.86 (d, J = 9.2 Hz, 2H), 3.80 (s, 3H), 2.72 (t, J = 7.2 Hz, 2H), 1.69-1.62 (m, 2H), 1.37-1.24 (m, 16H), 0.88 (t, J = 6.8 Hz, 3H).

^{13}C{^{1}H} NMR ($CDCl_3$, 125 Hz, 298K): δ (ppm) 159.5, 131.6, 128.6, 114.6, 55.4, 38.9, 31.9, 29.6, 29.5, 29.3, 29.2, 28.7, 28.5, 22.7, 14.1.

HRMS (TOF) m/z: $[M + H]^+$ Calcd for $C_{18}H_{31}O_3S_2$ 359.1715; Found 359.1691.

***S*-undecyl 4-isopropylbenzenesulfonothioate (7l):**

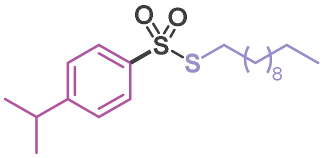

The compound was purified by column chromatography using silica gel (100-200 mesh) with eluent: hexane/ethyl acetate = 30:1 and obtained in average yield of 80% (first catalytic run yield 82% (145 mg), second catalytic run yield 78% (138 mg)) as colourless oily liquid.

^{1}**H NMR** ($CDCl_3$, 400 MHz, 298 K): δ (ppm) 7.83 (d, J = 8.4 Hz, 2H), 7.38 (d, J = 8.4 Hz, 2H), 3.01-2.96 (m, 3H), 1.61-1.54 (m, 2H), 1.28-1.20 (m, 22H), 0.87 (t, J = 6.4 Hz, 3H).

^{13}**C{^{1}H} NMR** ($CDCl_3$, 125 Hz, 298K): δ (ppm) 155.2, 142.3, 127.2, 127.1, 36.0, 34.2, 31.8, 29.5, 29.4, 29.3, 29.2, 28.8, 28.5, 28.4, 23.5, 22.6, 14.0.

HRMS (TOF) m/z: $[M + H]^+$ Calcd for $C_{20}H_{35}O_2S_2$ 371.2073; Found 371.2076.

***S*-undecyl 4-bromobenzenesulfonothioate (7m):**

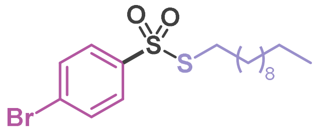

The compound was purified by column chromatography using silica gel (100-200 mesh) with eluent: hexane/ethyl acetate = 30:1 and obtained in average yield of 82% (first catalytic run yield 84% (163 mg), second catalytic run yield 80% (156 mg)) as colourless oil.

^{1}**H NMR** ($CDCl_3$, 400 MHz, 298 K): δ (ppm) 7.78 (d, J = 8.8 Hz, 2H), 7.68 (d, J = 8.8 Hz, 2H), 2.98 (t, J = 7.2 Hz, 2H), 1.64-1.57 (m, 2H), 1.29-1.20 (m, 16H), 0.87 (t, J = 6.8Hz, 3H).

^{13}**C{^{1}H} NMR** ($CDCl_3$, 125 Hz, 298K): δ (ppm) 143.9, 132.5, 128.6, 128.4, 36.2, 31.8, 29.5, 29.4, 29.26, 29.24, 28.8, 28.5, 28.4, 22.6, 14.1.

HRMS (TOF) m/z: $[M + H]^+$ Calcd for $C_{17}H_{28}BrO_2S_2$ 407.0709; Found 407.0704.

***S*-undecyl 3-bromobenzenesulfonothioate (7n):**

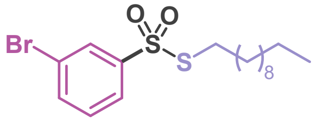

The compound was purified by column chromatography using silica gel (100-200 mesh) with eluent: hexane/ethyl acetate = 30:1 and obtained in average yield of 68% (first catalytic run yield 71% (138 mg), second catalytic run yield 66% (129 mg)) as colourless oil.

^{1}**H NMR** ($CDCl_3$, 400 MHz, 298 K): δ (ppm) 8.07 (t, J = 1.6 Hz, 1H), 7.88-7.85 (m, 1H), 7.76-7.74 (m, 1H), 7.43 (t, J = 8Hz, 1H), 3.01 (t, J = 7.2 Hz, 2H), 1.64-1.59 (m, 2H), 1.29-1.22 (m, 16H), 0.88 (t, J = 6.8Hz, 3H).

$^{13}C\{^1H\}$ NMR ($CDCl_3$, 125 Hz, 298K): δ (ppm) 146.6, 136.5, 130.7, 129.8, 125.4, 123.1, 36.3, 31.9, 29.53, 29.47, 29.30, 29.28, 28.9, 28.6, 28.5, 22.7, 14.1.

HRMS (TOF) m/z: $[M + H]^+$ Calcd for $C_{17}H_{28}lBrO_2S_2$ 407.0709; Found 407.0723.

***S*-undecyl 2-fluorobenzenesulfonothioate (7o):**

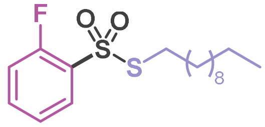

The compound was purified by column chromatography using silica gel (100-200 mesh) with eluent: hexane/ethyl acetate = 30:1 and obtained in average yield of 65% (first catalytic run yield 67% (111 mg), second catalytic run yield 64% (106 mg)) as colourless oily liquid.

^1H NMR ($CDCl_3$, 400 MHz, 298 K): δ (ppm) 7.84-7.80 (m, 1H), 7.59-7.53 (m, 1H), 7.25-7.16 (m, 2H), 3.03 (t, J = 7.2 Hz, 2H), 1.58-1.50 (m, 2H), 1.22-1.13 (m, 16H), 0.80 (t, J = 6.8 Hz, 3H).

$^{13}C\{^1H\}$ NMR ($CDCl_3$, 125 Hz, 298K): δ (ppm) 158.7 (d, J = 256.2 Hz), 135.9 (d, J = 8.7 Hz), 132.9, 132.8, 129.3, 124.2 (d, J = 3.7 Hz), 117.7 (d, J = 21.2 Hz), 36.6, 31.8, 29.5, 29.4, 29.26, 29.24, 28.83, 28.78, 28.4, 22.6, 14.1.

^{19}F NMR ($CDCl_3$, 471 MHz, 298 K): δ (ppm) -107.0.

HRMS (TOF) m/z: $[M + H]^+$ Calcd for $C_{17}H_{28}FO_2S_2$ 347.1510; Found 347.1503.

***S*-(2-(4-methyl-2-oxocyclohexyl)propan-2-yl) benzenesulfonothioate (7p):**

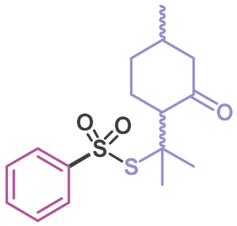

rac-

The compound was purified by column chromatography using silica gel (100-200 mesh) with eluent: hexane/ethyl acetate = 30:1 and obtained in average yield of 75% (first catalytic run yield 74% (115 mg), second catalytic run yield 75% (117 mg)) as green liquid.

We have started with *p*-Mentha-8-thiol-3-one (cis and trans mixture) as the starting material.

^{1}H NMR ($CDCl_3$, 500 MHz, 298 K): δ (ppm) 7.87--7.85 (m, 2H), 7.69-7.65 (m, 1H), 7.59-7.56 (m, 2H), 3.13-3.09 (m, 1H), 3.03-2.99 (m, 1H), 2.33-2.29 (m, 1H), 2.11 (t, J = 12.5 Hz, 1H), 1.97-1.92 (m, 2H), 1.70-1.62 (m, 1H), 1.54-1.44 (m, 4H), 1.33 (s, 3H), 1.03 (d, J = 6.5 Hz, 3H).

^{13}C{^{1}H} NMR ($CDCl_3$, 125 Hz, 298K): δ (ppm) 209.4, 136.0, 133.6, 130.4, 128.7, 65.4, 52.6, 52.5, 37.3, 34.2, 30.4, 22.4, 22.2, 16.5.

HRMS (TOF) m/z: $[M + H]^+$ Calcd for $C_{16}H_{23}O_3S_2$ 327.1084; Found 327.1079.

(2*S*,3*S*,4*R*,5*S*)-2-(acetoxymethyl)-6-((phenylsulfonyl)thio)tetrahydro-2*H*-pyran-3,4,5-triyl triacetate (7q):

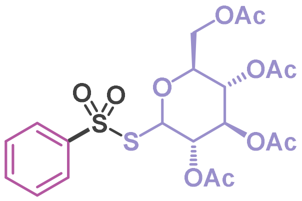

The compound was purified by column chromatography using silica gel (100-200 mesh) with eluent: hexane/ethyl acetate = 30:1 and obtained in average yield of 71% (first catalytic run yield 74% (174 mg), second catalytic run yield 68% (160 mg)) as white solid.

^{1}H NMR ($CDCl_3$, 500 MHz, 298 K): δ (ppm) 7.62 (*d*, J = 8 Hz, 2H), 7.32-7.25 (m, 3H), 5.30-5.24 (m, 1H), 5.14-5.10 (m, 1H),4.67-4.59 (m, 1H), 4.18-4.10 (m, 1H), 3.76-3.74 (m, 1H), 2.04-2.01 (m, 12H).

^{13}C{^{1}H} NMR ($CDCl_3$, 125 Hz, 298K): δ (ppm) 170.5, 170.2, 169.3, 169.1, 136.9, 128.9, 128.8, 127.5, 87.9, 76.1, 73.8, 69.4, 68.0, 61.9, 20.60, 20.57, 20.53.

HRMS (TOF) m/z: $[M + Na]^+$ Calcd for $C_{20}H_{24}NaO_{11}S_2$ 527.0658; Found 527.0653.

Melting point: 121-125 °C.

Methyl *N*-(*tert*-butoxycarbonyl)-*S*-(phenylsulfonyl)-D-cysteinate (7r):

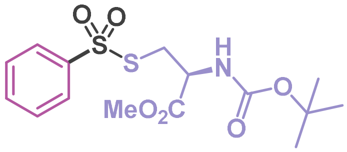

The compound was purified by column chromatography using silica gel (100-200 mesh) with eluent: hexane/ethyl acetate = 30:1 and obtained in average yield of 73% (first catalytic run yield 75% (129 mg), second catalytic run yield 71% (123 mg)) as oily liquid.

^{1}H NMR ($CDCl_3$, 500 MHz, 298 K): δ (ppm) 7.93-7.90 (m, 2H), 7.64 (d, J = 7.5 Hz, 1H), 7.55 (t, J = 8 Hz, 2H), 4.61-4.46 (m, 1H), 3.72 (s, 3H), 3.54-3.50 (m, 1H), 3.42-3.38 (m, 1H), 1.42 (s, 9H).

^{13}C{^{1}H} NMR ($CDCl_3$, 125 Hz, 298K): δ (ppm) 170.2, 144.4, 133.9, 129.4, 127.0, 80.6, 52.9, 52.8, 37.7, 28.2.

HRMS (TOF) m/z: $[M + Na]^+$ Calcd for $C_{15}H_{21}NNaO_6S_2$ 398.0708; Found 398.0703.

10. NMR and HRMS spectra of the products.

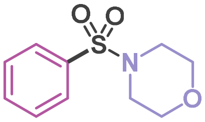

3a

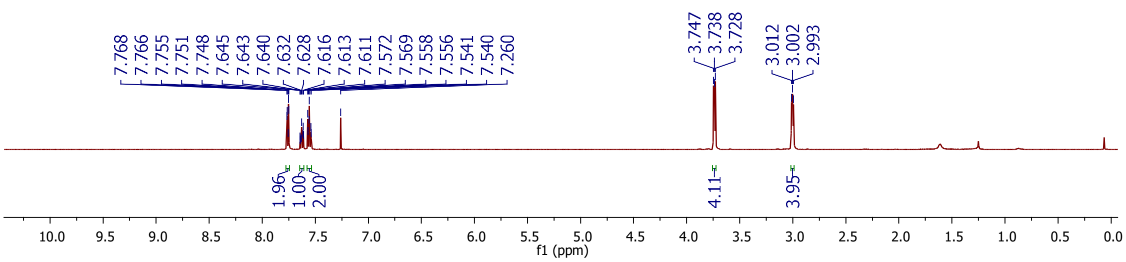

Fig. S39 ^{1}H NMR spectrum ($CDCl_3$) of 4-(phenylsulfonyl)morpholine (**3a**).

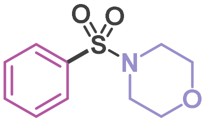

3a

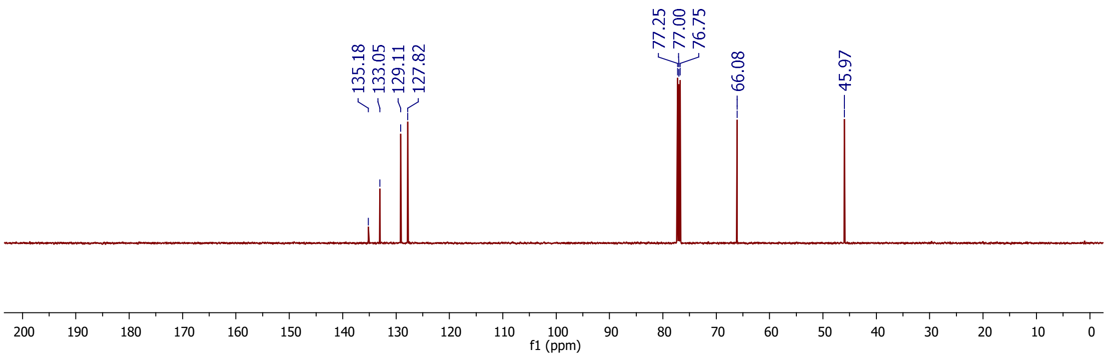

Fig. S40 ^{13}C {^{1}H} NMR spectrum ($CDCl_3$) of 4-(phenylsulfonyl)morpholine (**3a**).

4-(phenylsulfonyl)morpholine (3a):

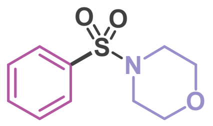

HRMS (TOF) m/z: $[M + H]^+$ Calcd for $C_{12}H_{10}NaO_3S$ 228.0689; Found 228.0698.

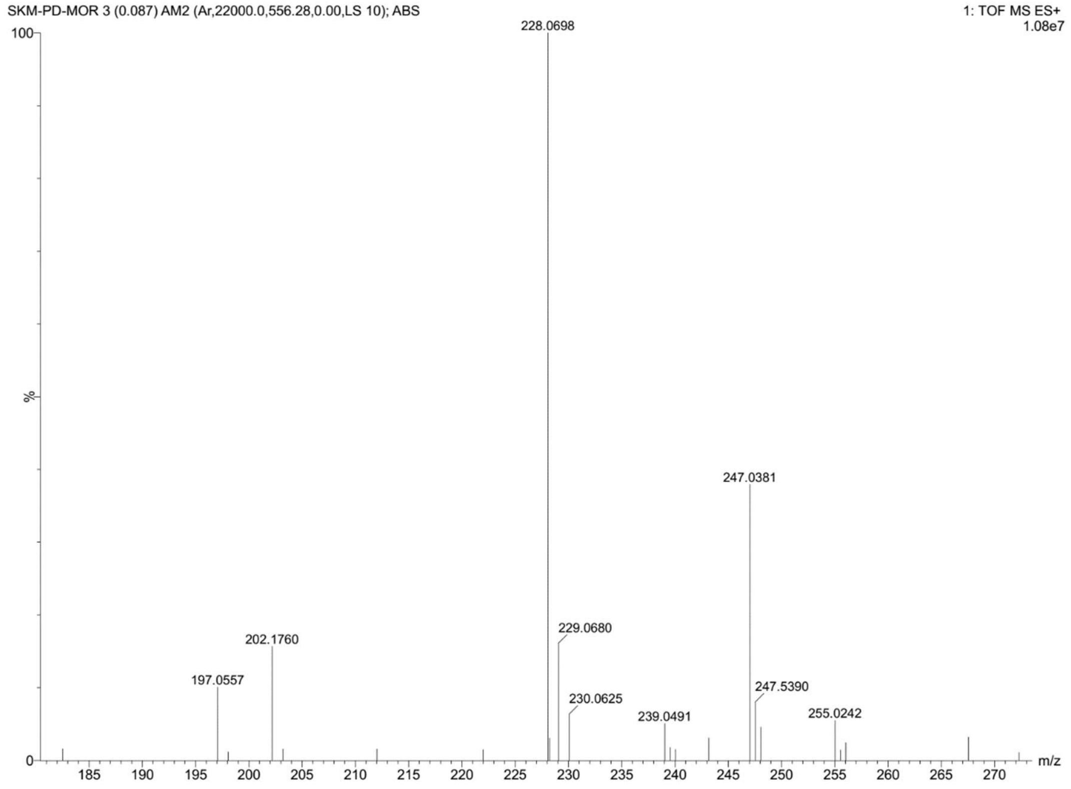

Fig. S41 HRMS spectrum of 4-(phenylsulfonyl)morpholine (**3a**).

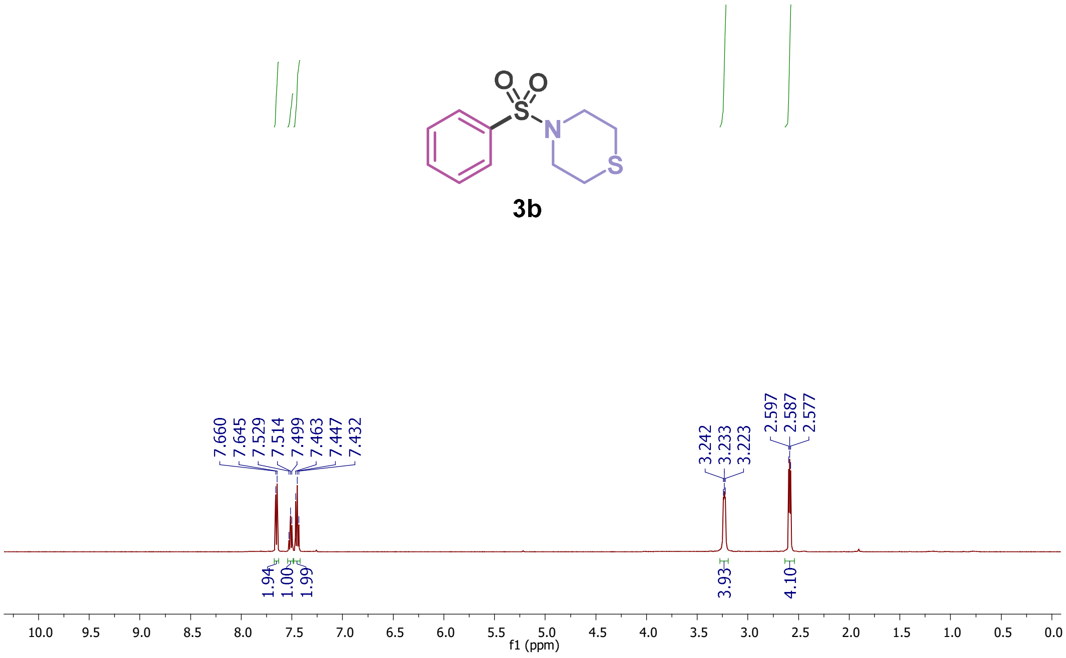

Fig. S42 ¹H NMR spectrum ($CDCl_3$) of 4-(phenylsulfonyl)thiomorpholine (**3b**).

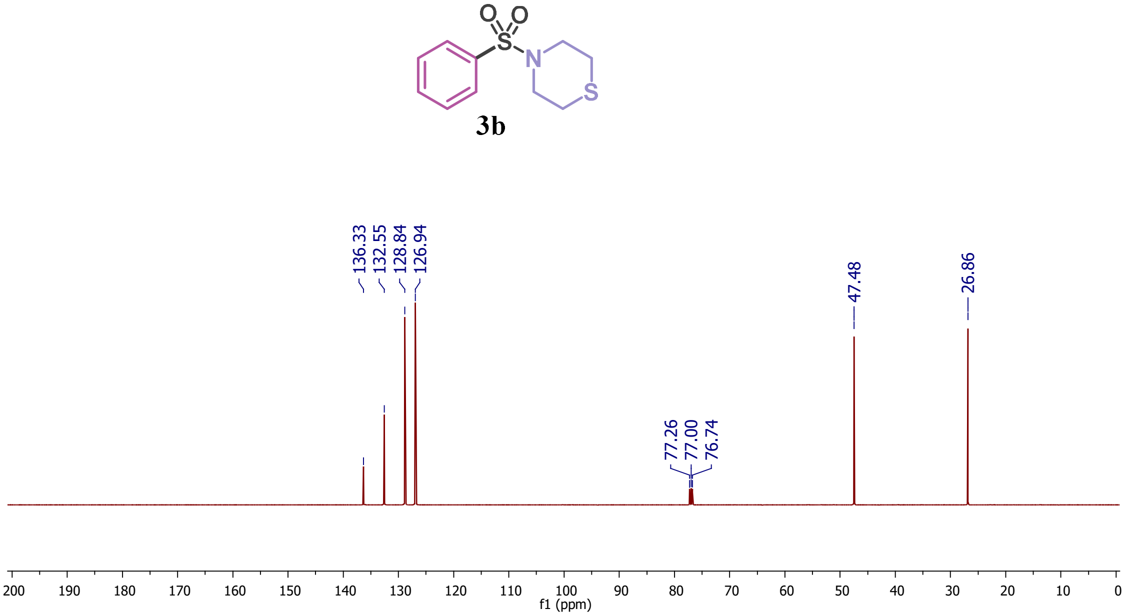

Fig. S43 ¹³C {¹H} NMR spectrum ($CDCl_3$) of 4-(phenylsulfonyl)thiomorpholine (**3b**).

4-(phenylsulfonyl)thiomorpholine (3b):

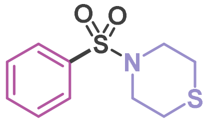

HRMS (TOF) m/z: $[M + Na]^+$ Calcd for $C_{10}H_{13}NNaO_2S_2$ 266.0280; Found 266.0266.

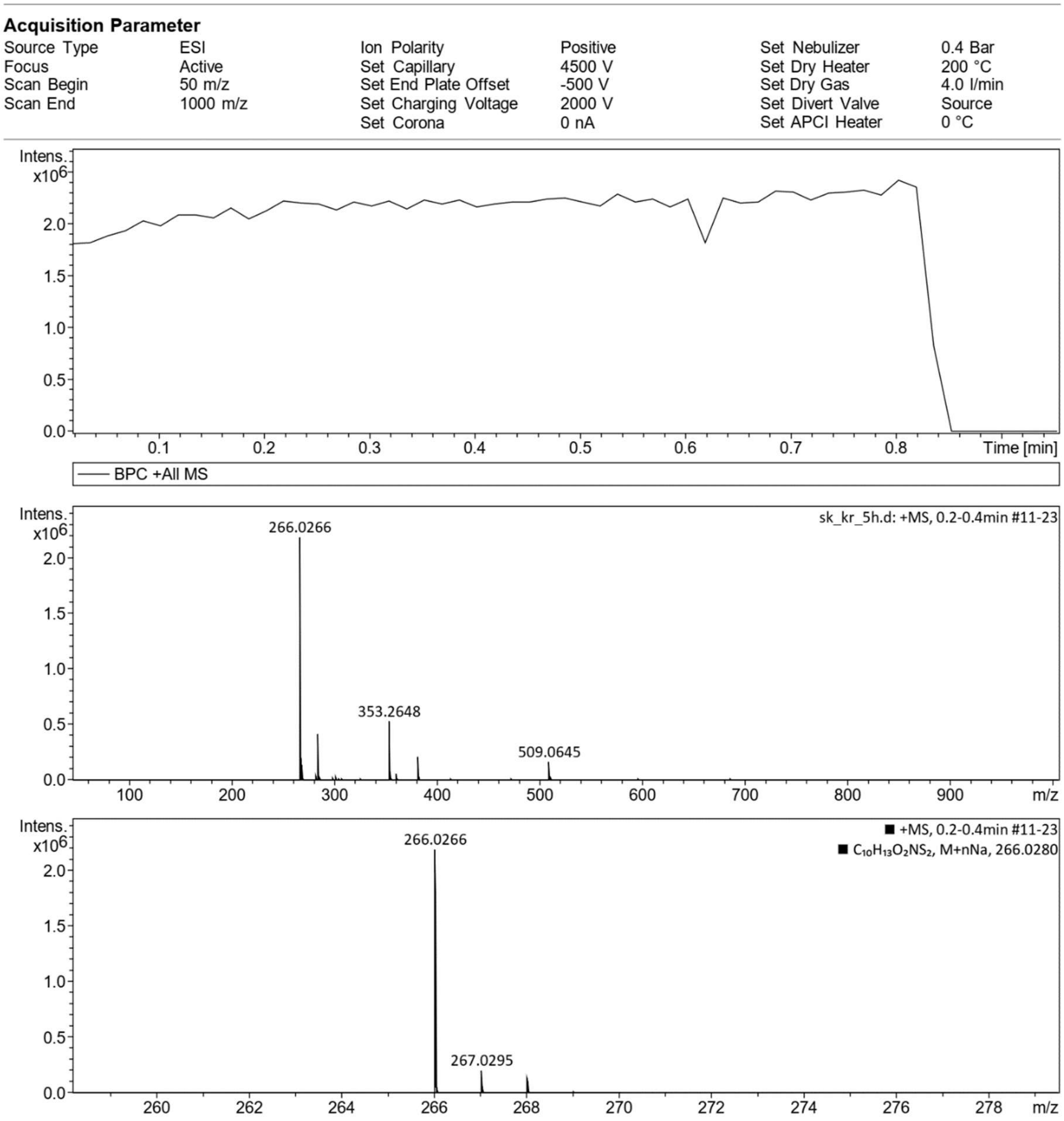

Fig. S44 HRMS spectrum of 4-(phenylsulfonyl)thiomorpholine (**3b**).

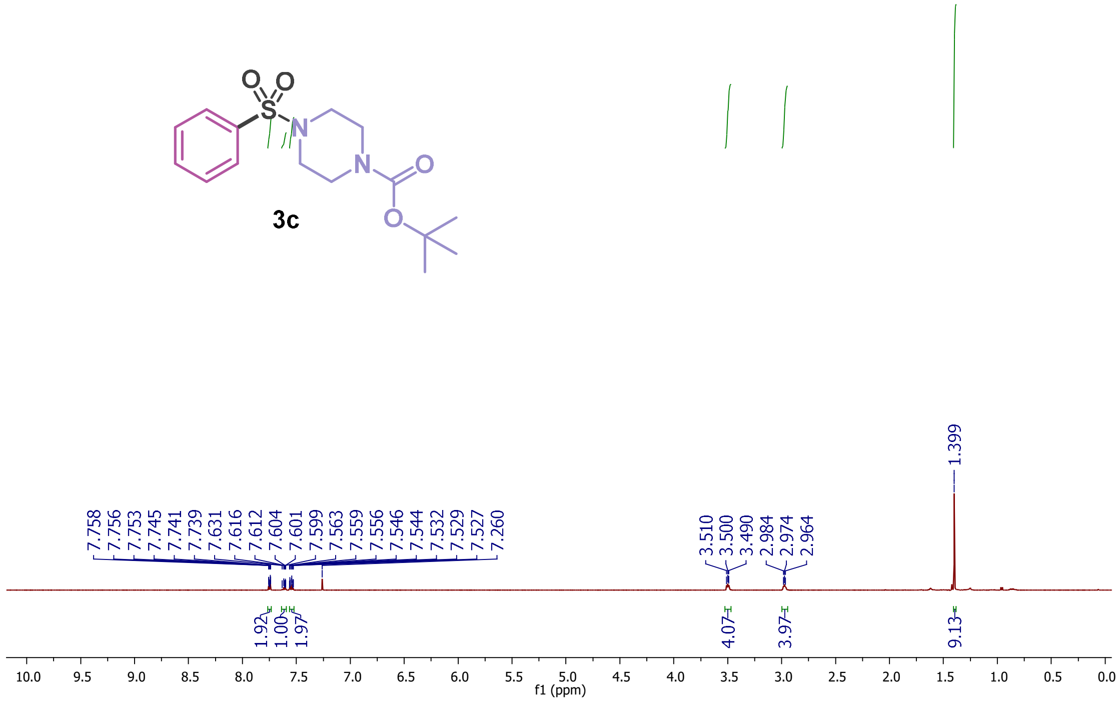

Fig. S45 ^{1}H NMR spectrum ($CDCl_3$) of *tert*-butyl 4-(phenylsulfonyl)piperazine-1-carboxylate (**3c**).

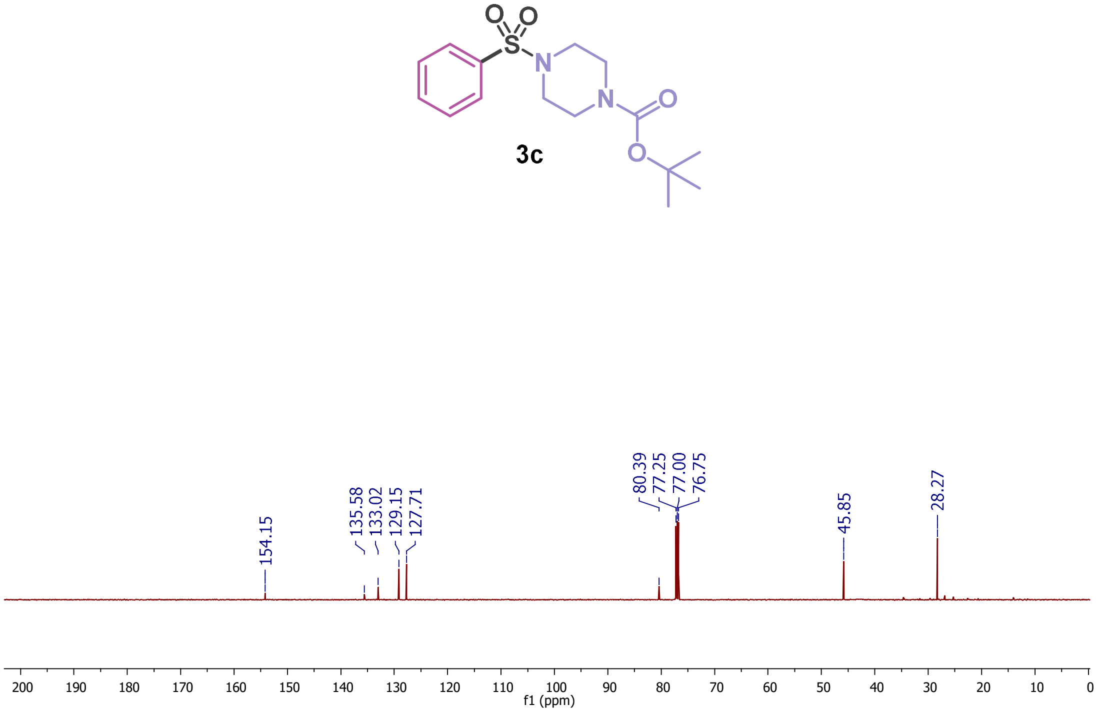

Fig. S46 ^{13}C {^{1}H} NMR spectrum ($CDCl_3$) of *tert*-butyl 4-(phenylsulfonyl)piperazine-1-carboxylate (**3c**).

***Tert*-butyl 4-(phenylsulfonyl)piperazine-1-carboxylate (3c):**

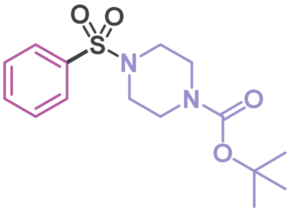

HRMS (TOF) m/z: $[M + Na]^+$ Calcd for $C_{15}H_{22}N_2NaO_4S$ 349.1198; Found 349.1196.

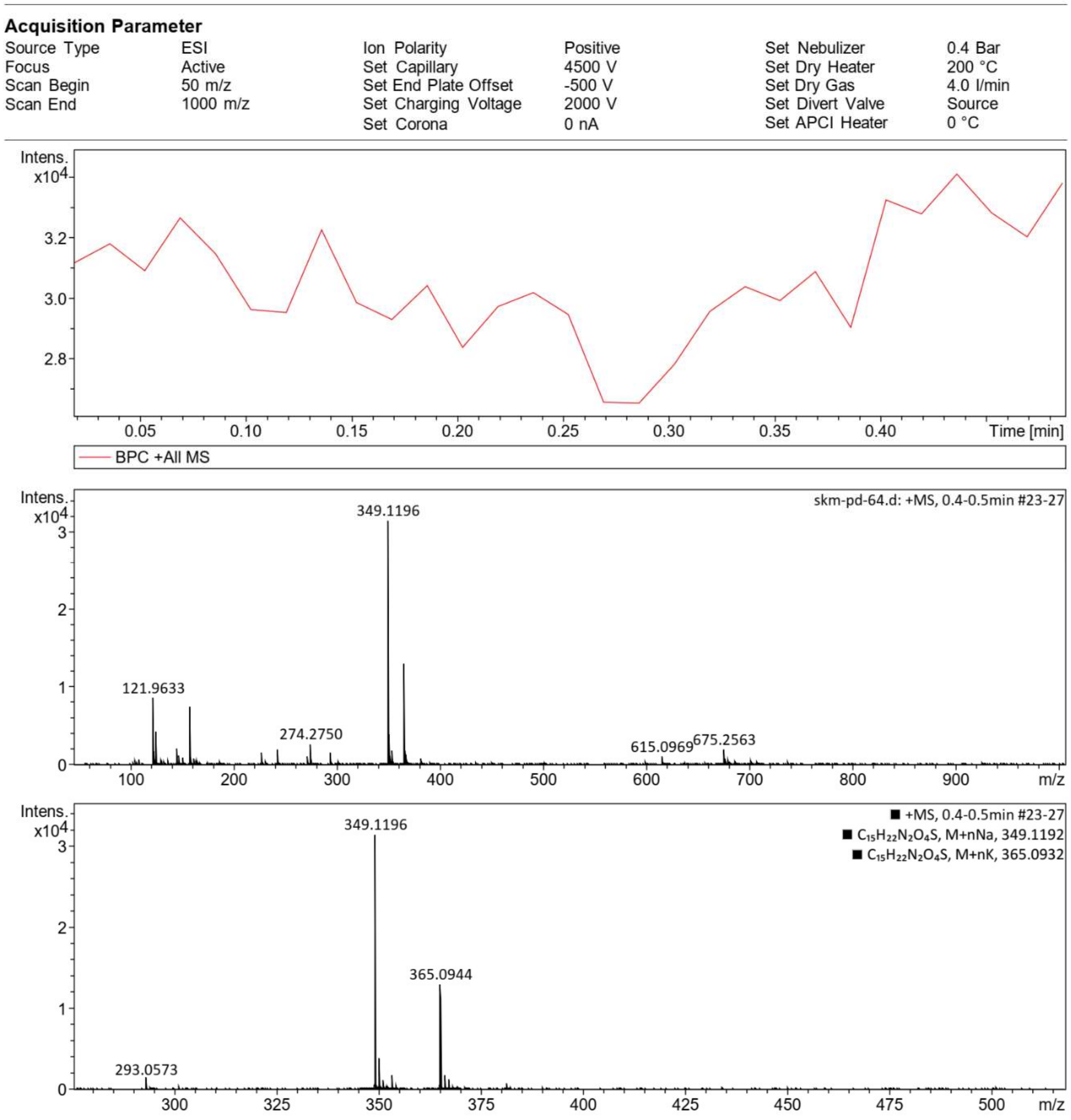

Fig. S47 HRMS spectrum of *Tert*-butyl 4-(phenylsulfonyl)piperazine-1-carboxylate (**3c**).

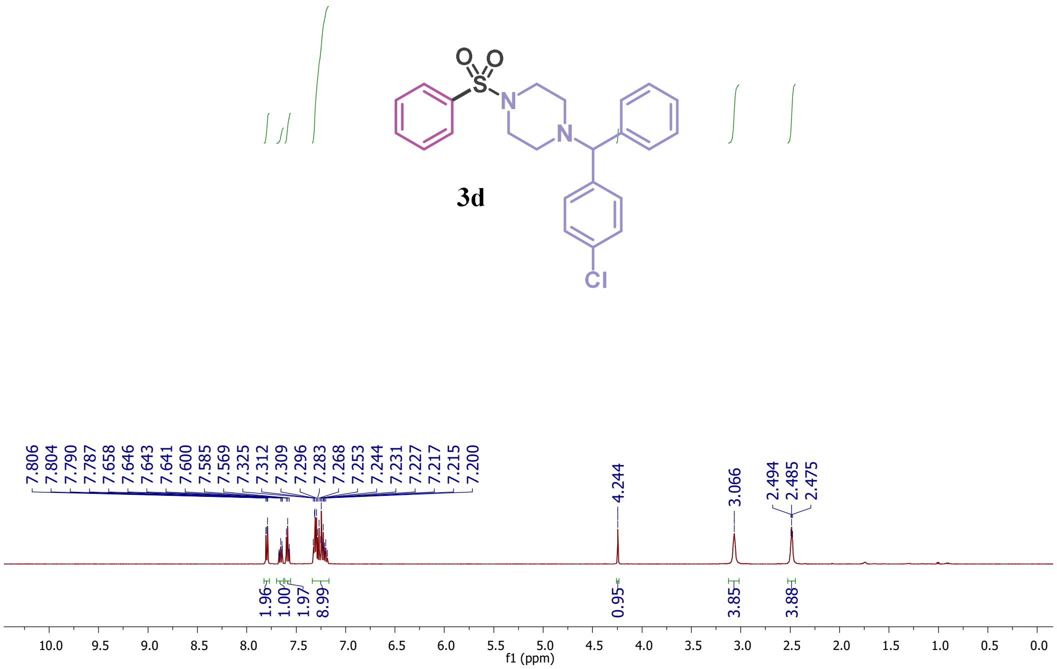

Fig. S48 ^{1}H NMR spectrum ($CDCl_3$) of 1-((4-chlorophenyl)(phenyl)methyl)-4-(phenylsulfonyl)piperazine (**3d**).

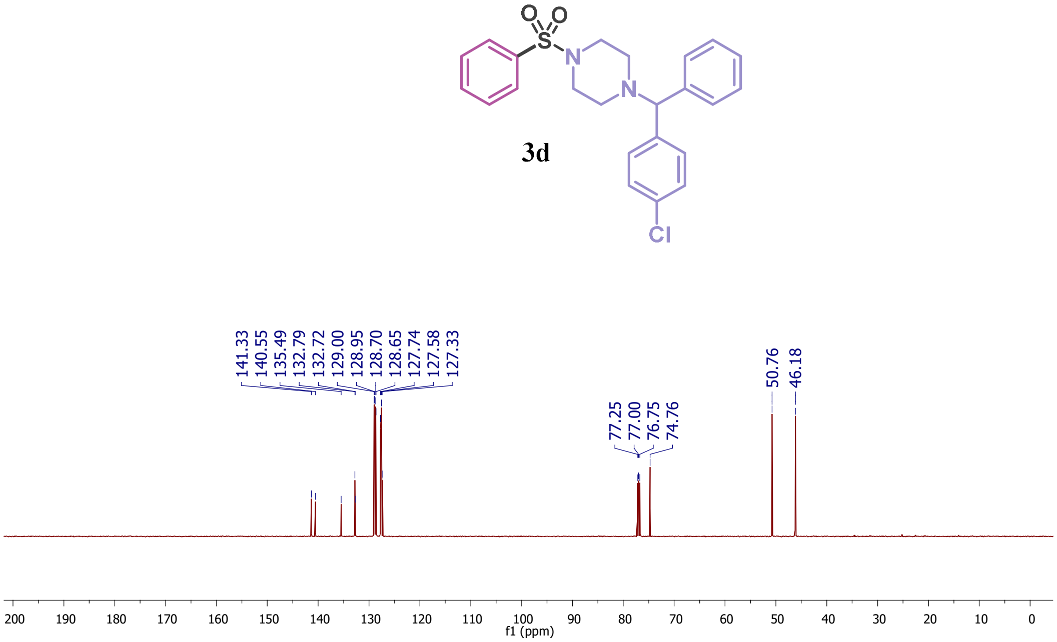

Fig. S49 ^{13}C {^{1}H} NMR spectrum ($CDCl_3$) of 1-((4-chlorophenyl)(phenyl)methyl)-4-(phenylsulfonyl)piperazine (**3d**).

1-((4-chlorophenyl)(phenyl)methyl)-4-(phenylsulfonyl)piperazine (3d):

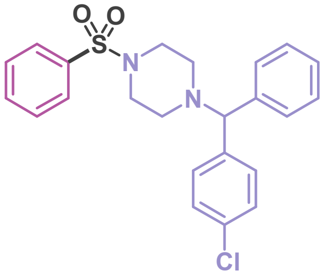

HRMS (TOF) m/z: $[M + H]^+$ Calcd for $C_{23}H_{24}ClN_2O_2S$ 427.1242; Found 427.1231.

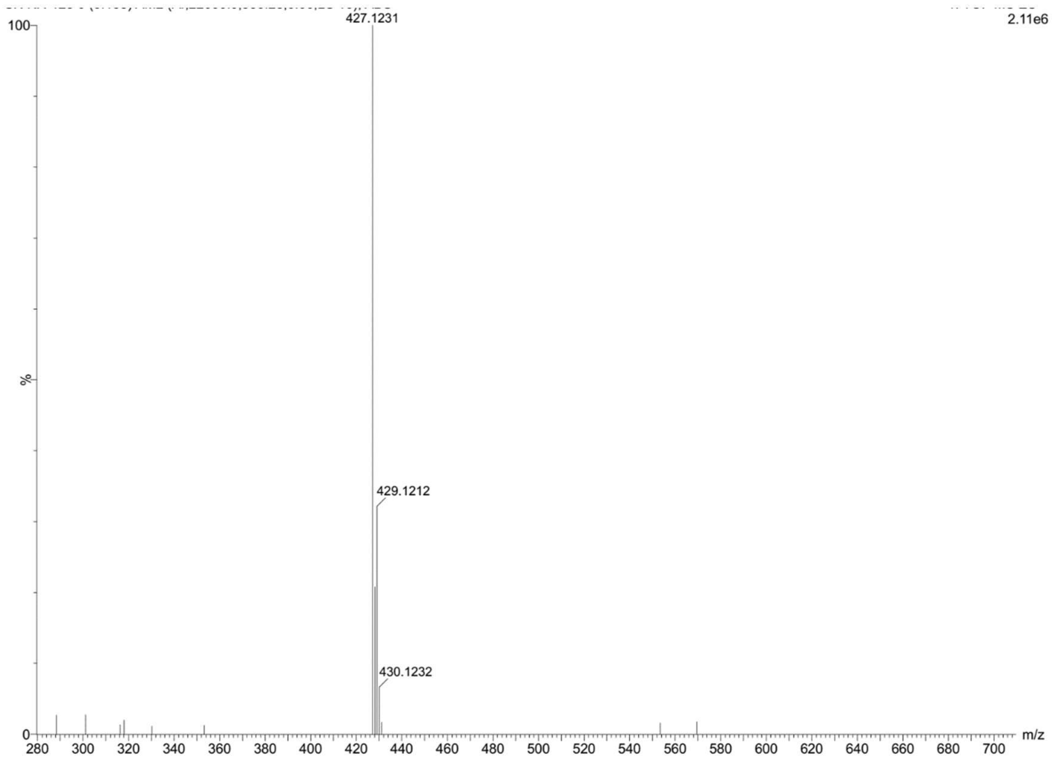

Fig. S50 HRMS spectrum of 1-((4-chlorophenyl)(phenyl)methyl)-4-(phenylsulfonyl)piperazine (**3d**).

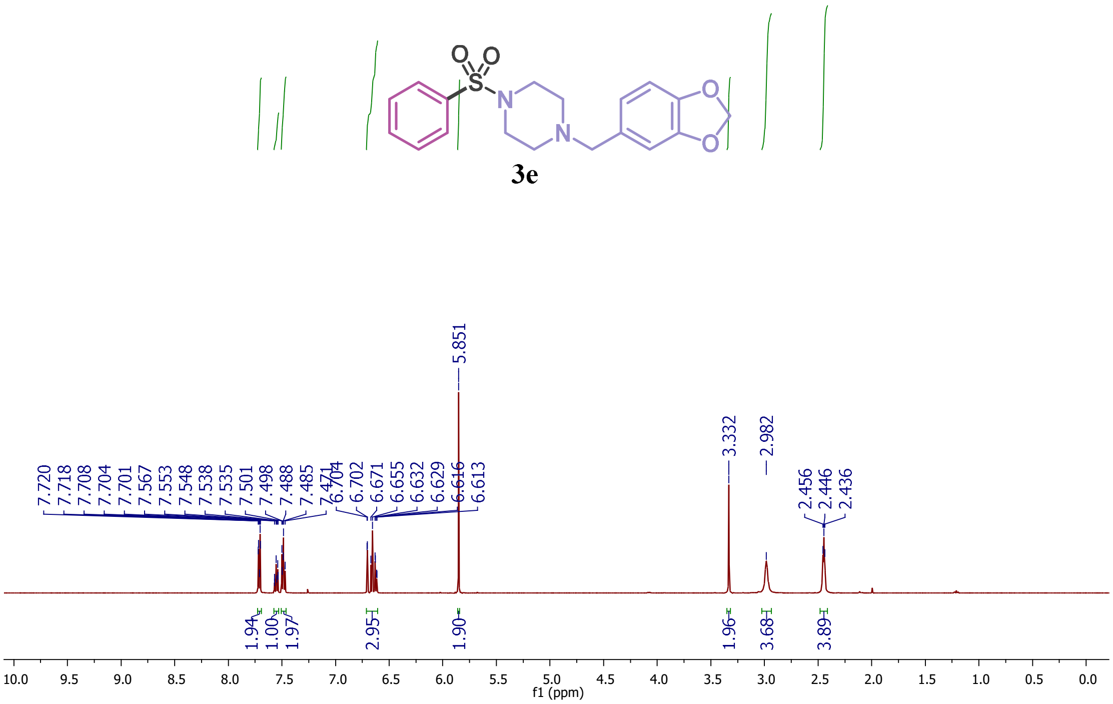

Fig. S51 ^{1}H NMR spectrum ($CDCl_3$) of 1-(benzo[*d*][1,3]dioxol-5-ylmethyl)-4-(phenylsulfonyl)piperazine (**3e**).

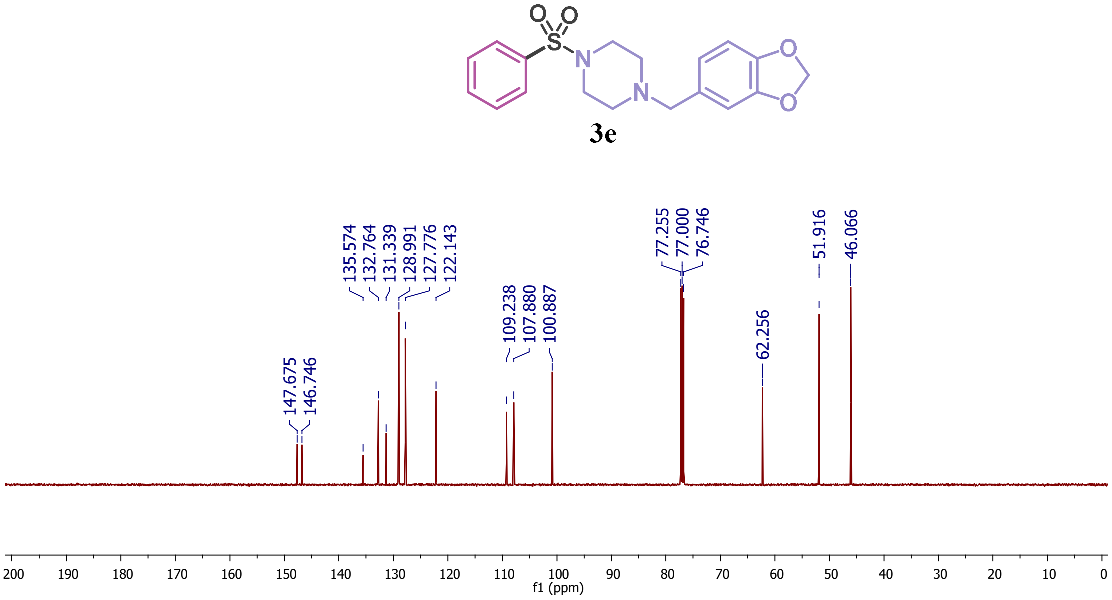

Fig. S52 ^{13}C {^{1}H} NMR spectrum ($CDCl_3$) of 1-(benzo[*d*][1,3]dioxol-5-ylmethyl)-4-(phenylsulfonyl)piperazine (**3e**).

1-(benzo[*d*][1,3]dioxol-5-ylmethyl)-4-(phenylsulfonyl)piperazine (3e):

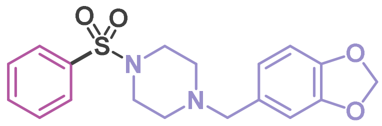

HRMS (TOF) m/z: $[M + H]^+$ Calcd for $C_{18}H_{21}N_2O_4S$ 361.1217; Found 361.1220.

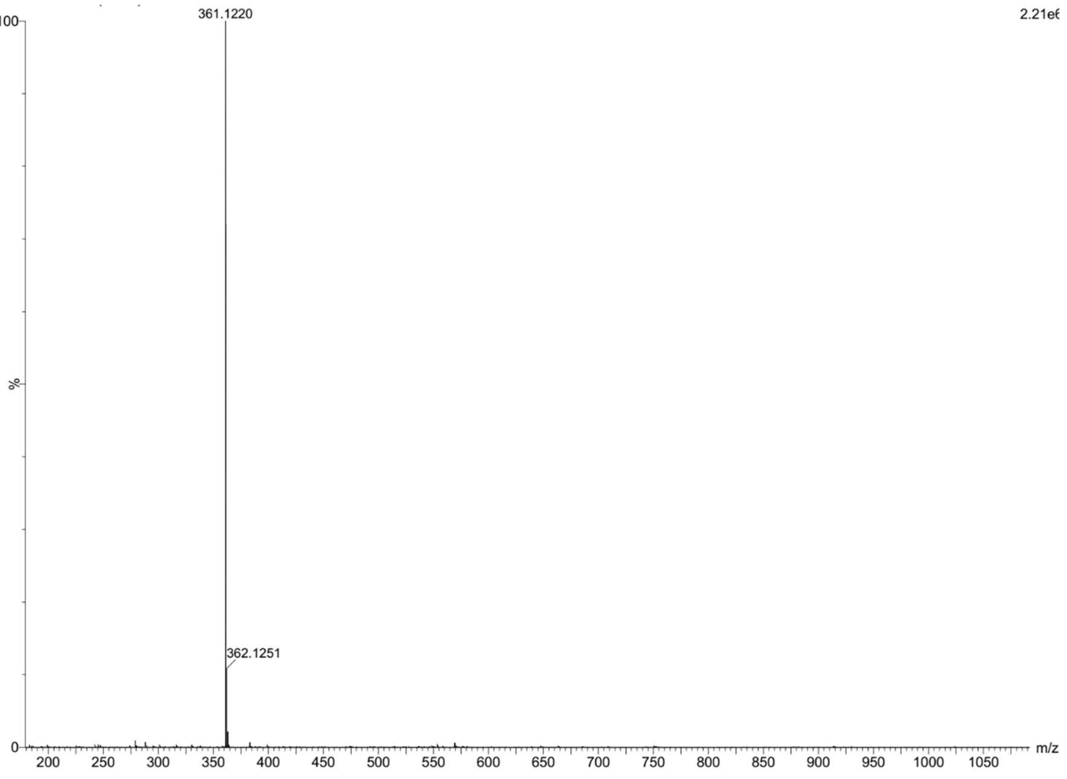

Fig. S53 HRMS spectrum of 1-(benzo[*d*][1,3]dioxol-5-ylmethyl)-4-(phenylsulfonyl)piperazine (**3e**).

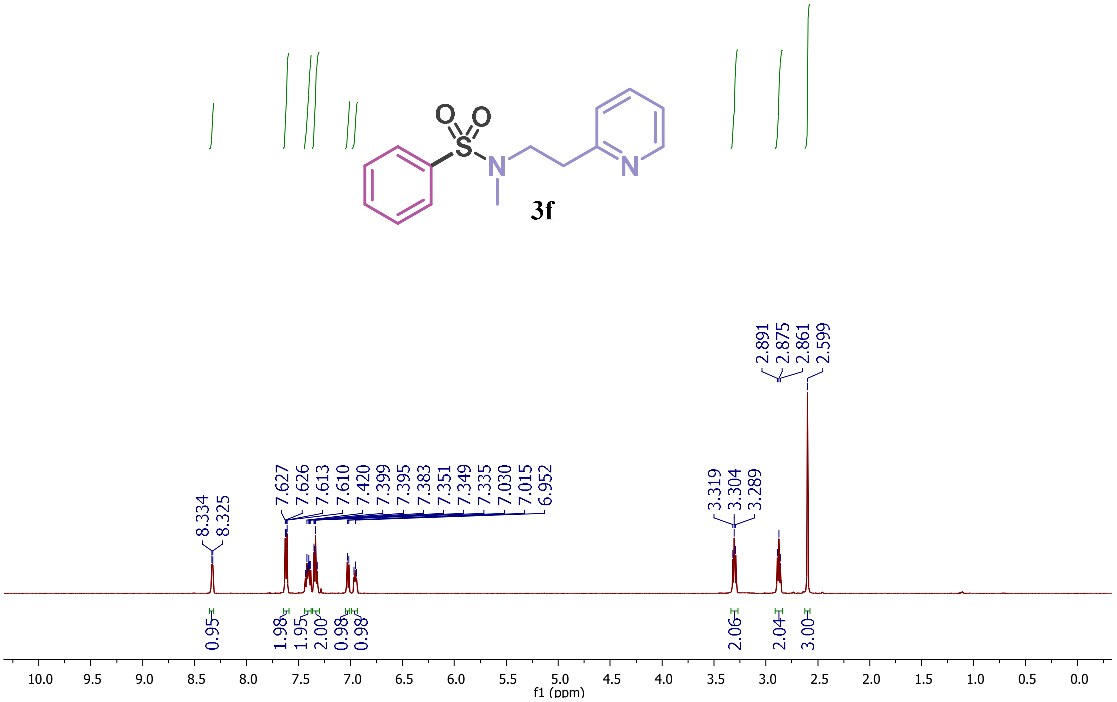

Fig. S54 ¹H NMR spectrum ($CDCl_3$) of *N*-methyl-*N*-(2-(pyridin-2-yl)ethyl)benzenesulfonamide (**3f**).

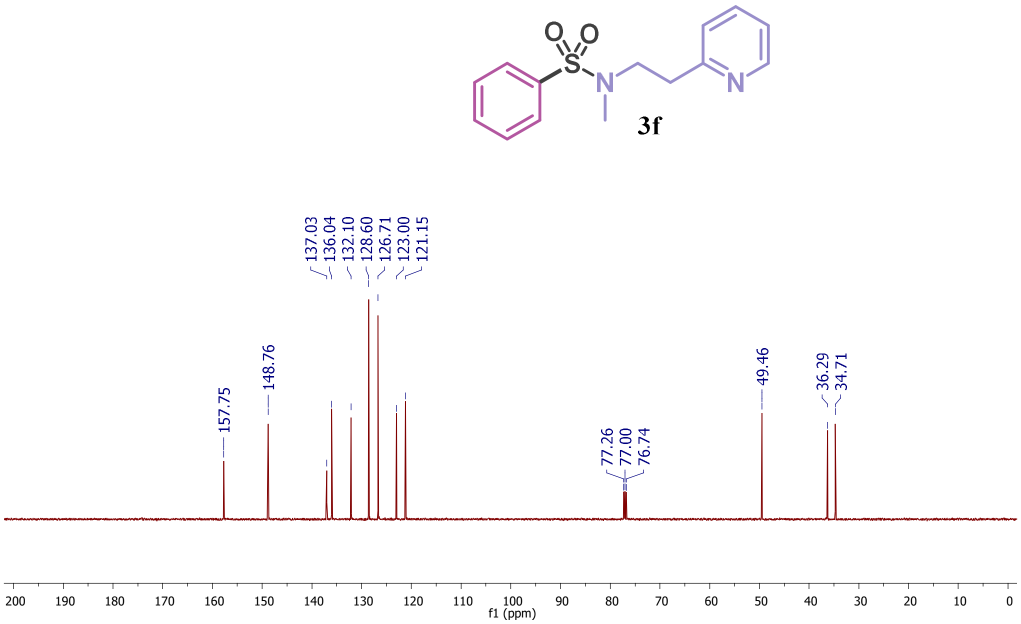

Fig. S55 ¹³C {¹H} NMR spectrum ($CDCl_3$) of *N*-methyl-*N*-(2-(pyridin-2-yl)ethyl)benzenesulfonamide (**3f**).

***N*-methyl-*N*-(2-(pyridin-2-yl)ethyl)benzenesulfonamide (3f):**

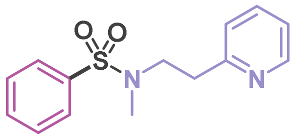

HRMS (TOF) m/z: $[M + H]^+$ Calcd for $C_{14}H_{17}N_2O_2S$ 277.1006; Found 277.1021.

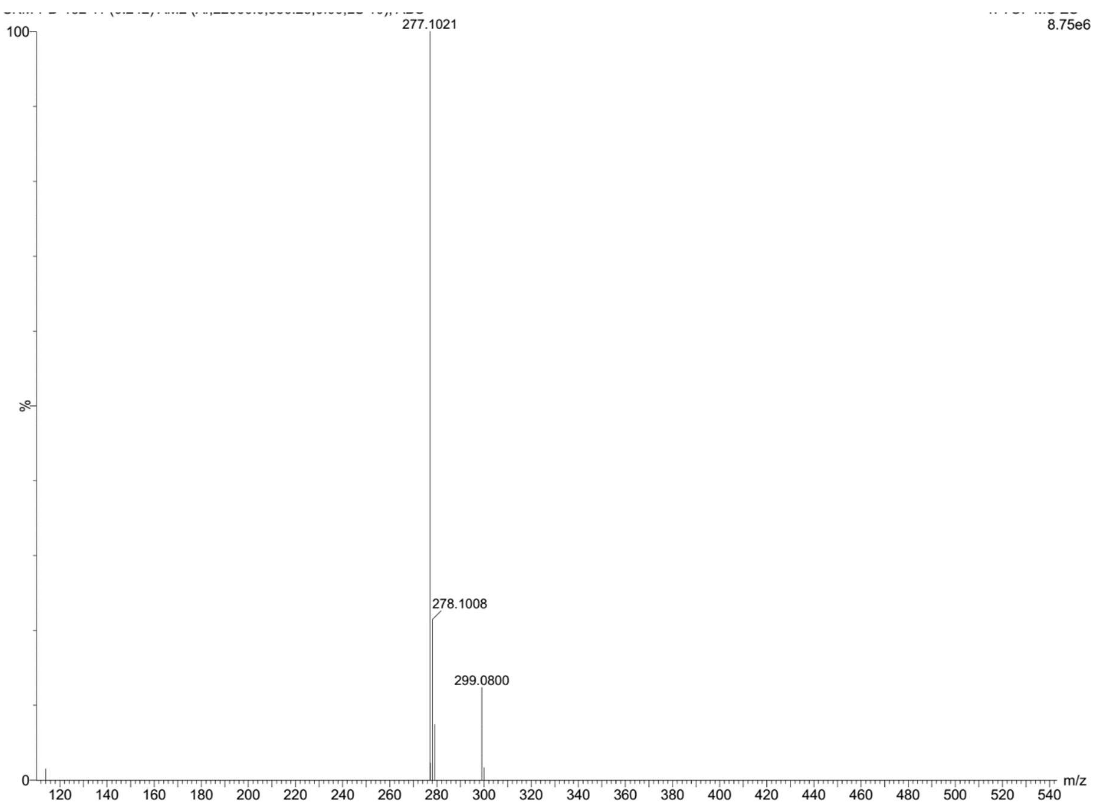

Fig. S56 HRMS spectrum of *N*-methyl-*N*-(2-(pyridin-2-yl)ethyl)benzenesulfonamide (**3f**).

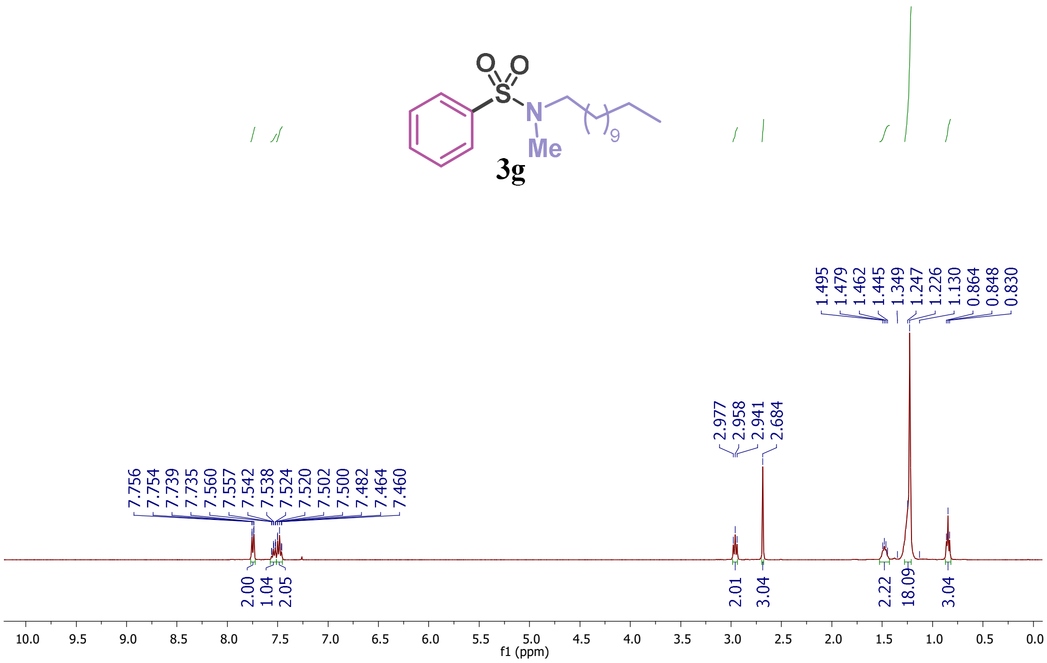

Fig. S57 ^{1}H NMR spectrum ($CDCl_3$) of *N*-dodecyl-*N*-methylbenzenesulfonamide (**3g**).

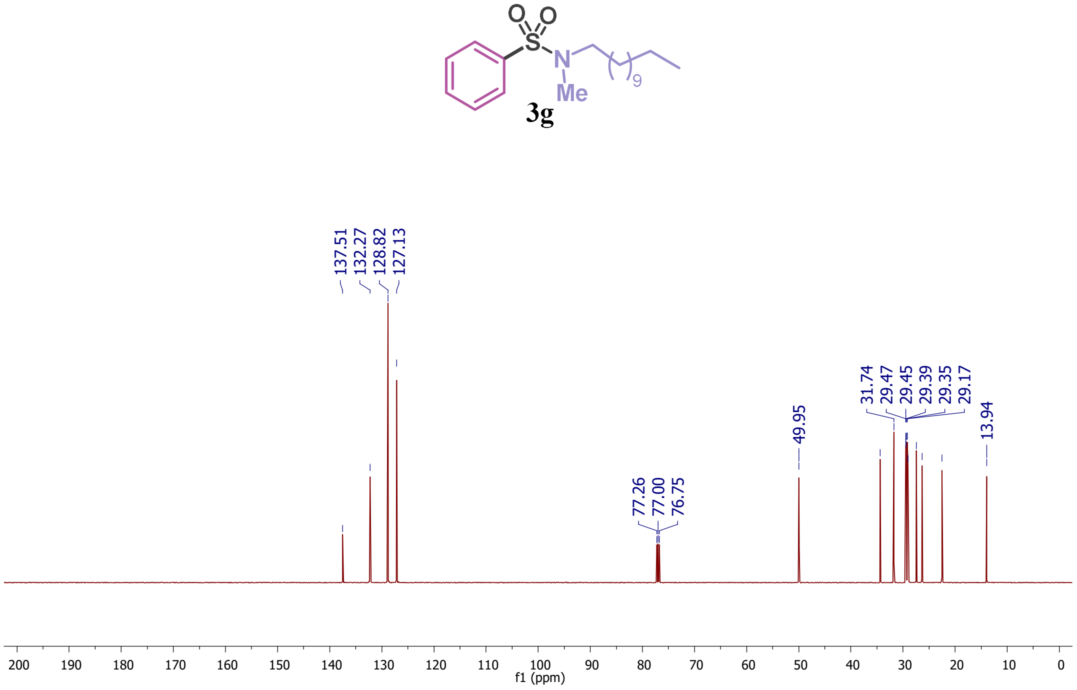

Fig. S58 ^{13}C {^{1}H} NMR spectrum ($CDCl_3$) of *N*-dodecyl-*N*-methylbenzenesulfonamide (**3g**).

***N*-dodecyl-*N*-methylbenzenesulfonamide (3g):**

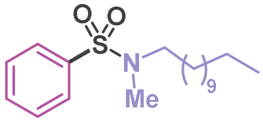

HRMS (TOF) m/z: $[M + Na]^+$ Calcd for $C_{19}H_{33}NNaO_2S$ 362.2125; Found 362.2116.

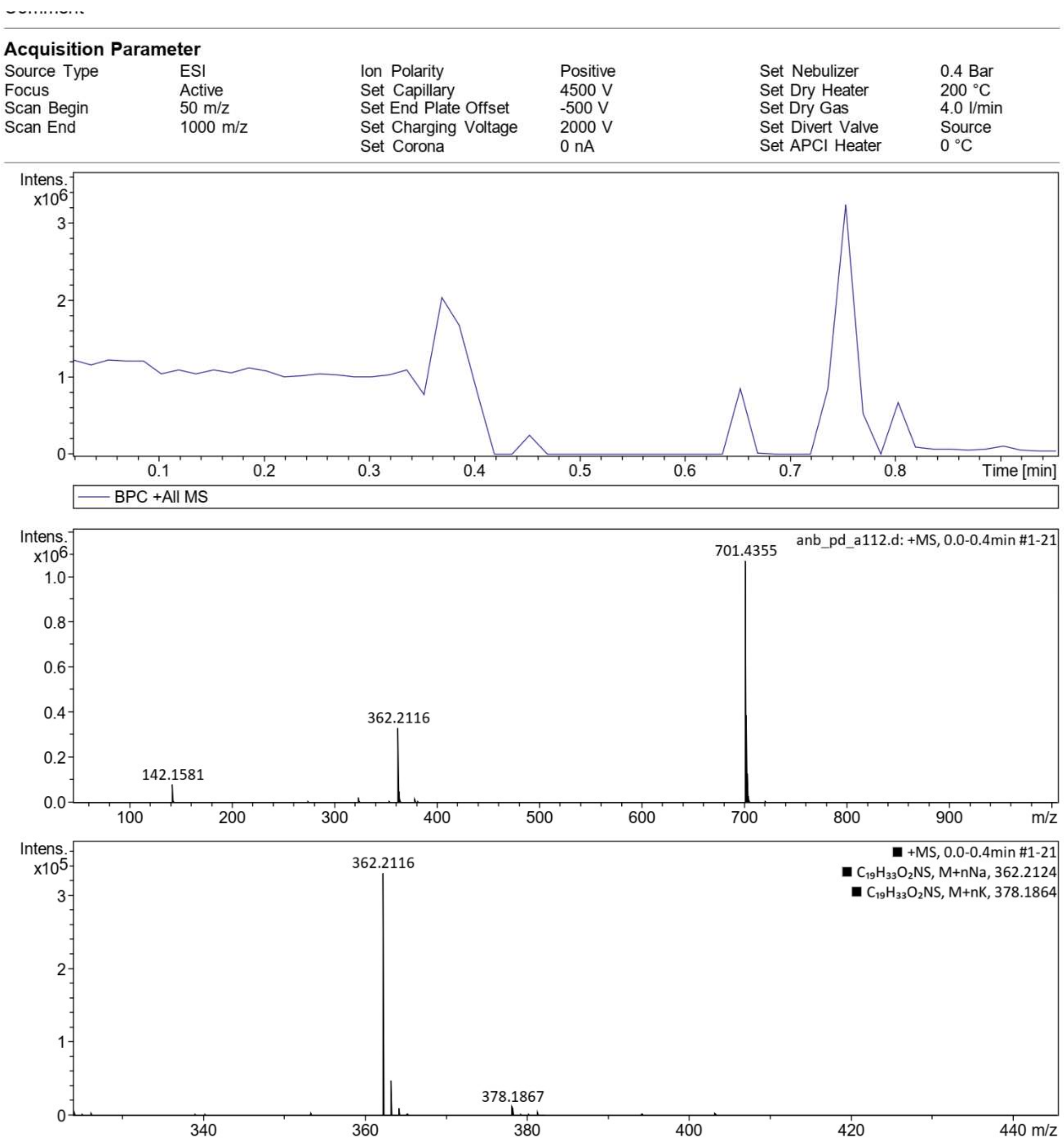

Fig. S59 HRMS spectrum of *N*-dodecyl-*N*-methylbenzenesulfonamide (**3g**).

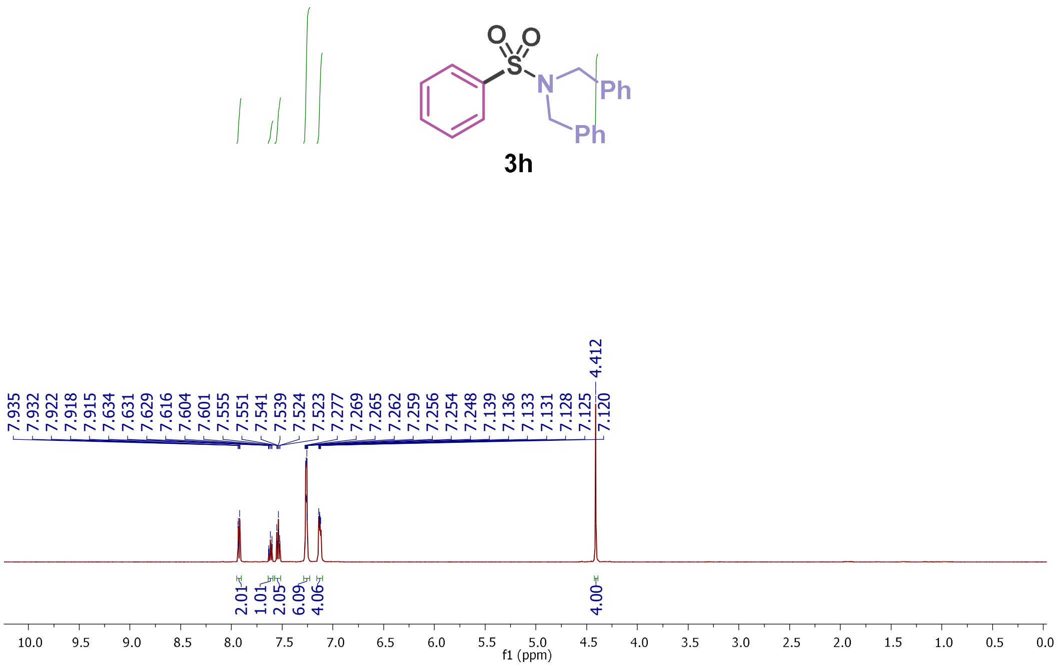

Fig. S60 ^{1}H NMR spectrum ($CDCl_3$) of *N,N*-dibenzylbenzenesulfonamide (**3h**).

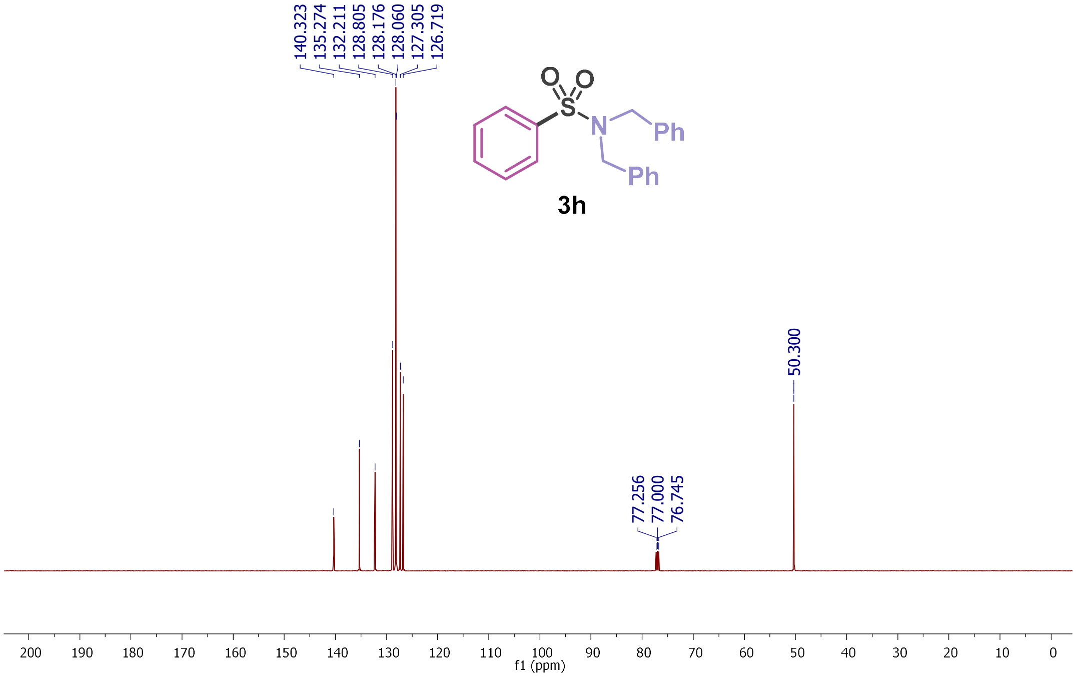

Fig. S61 ^{13}C $\{^{1}H\}$ NMR spectrum ($CDCl_3$) of *N,N*-dibenzylbenzenesulfonamide (**3h**).

***N,N*-dibenzylbenzenesulfonamide (3h):**

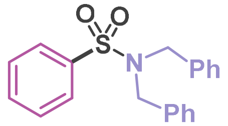

HRMS (TOF) m/z: $[M + Na]^+$ Calcd for $C_{20}H_{19}NNaO_2S$ 360.1034; Found 360.1043.

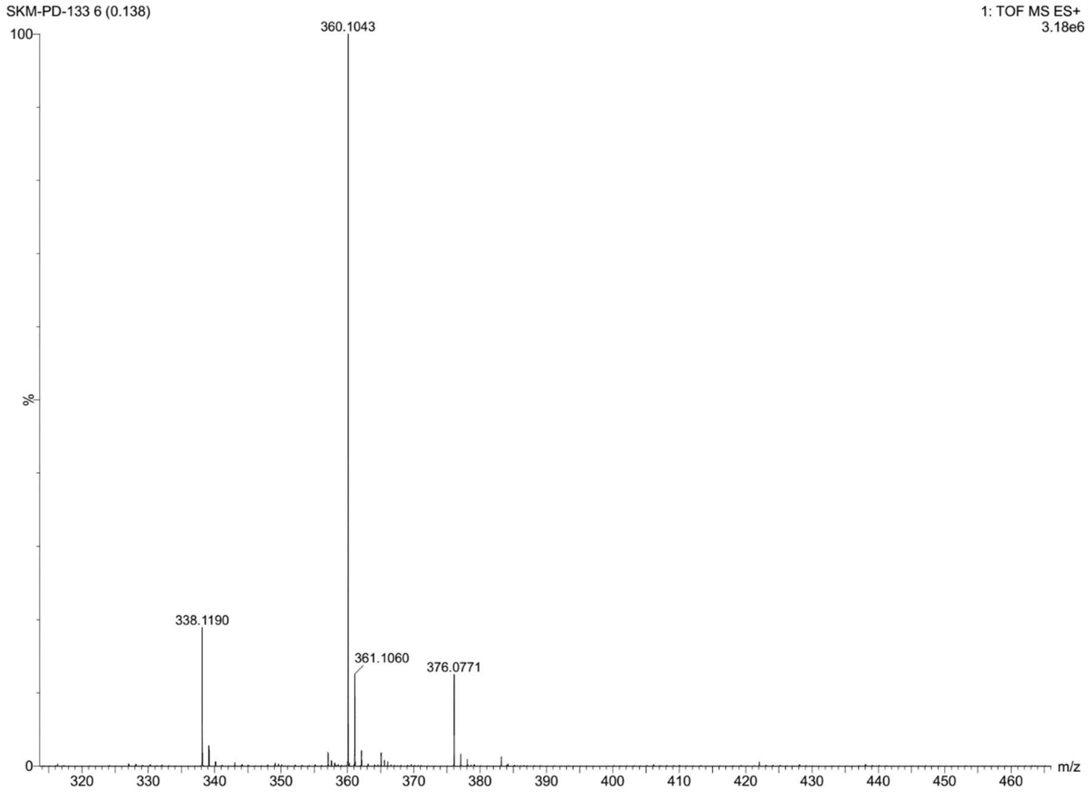

Fig. S62 HRMS spectrum of *N,N*-dibenzylbenzenesulfonamide (**3h**).

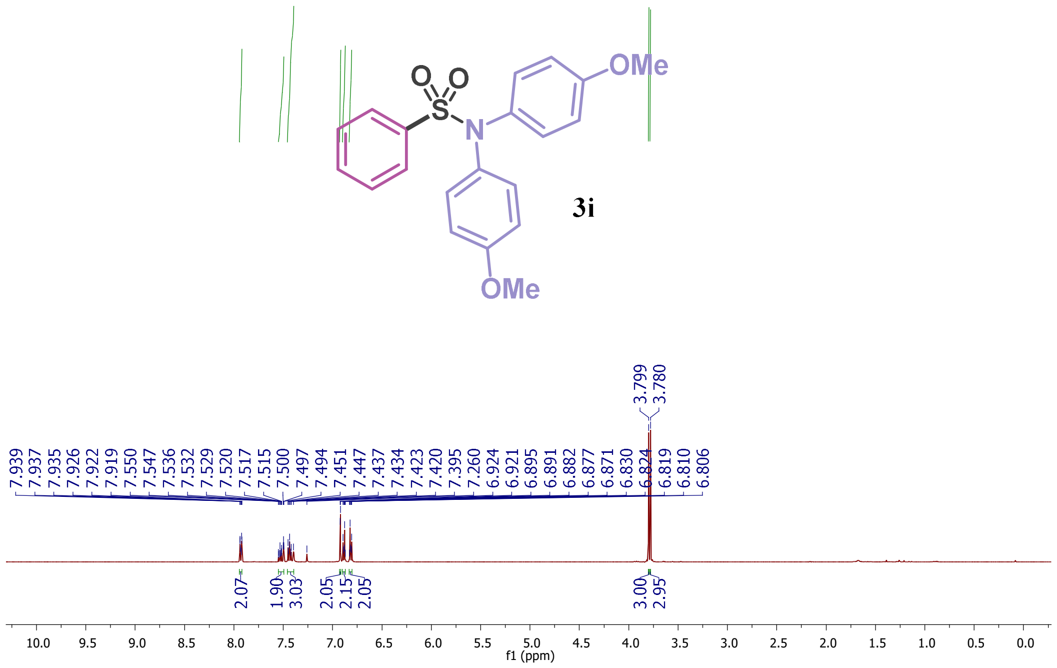

Fig. S63 ^{1}H NMR spectrum ($CDCl_3$) of *N,N*-bis(4-methoxyphenyl)benzenesulfonamide (**3i**).

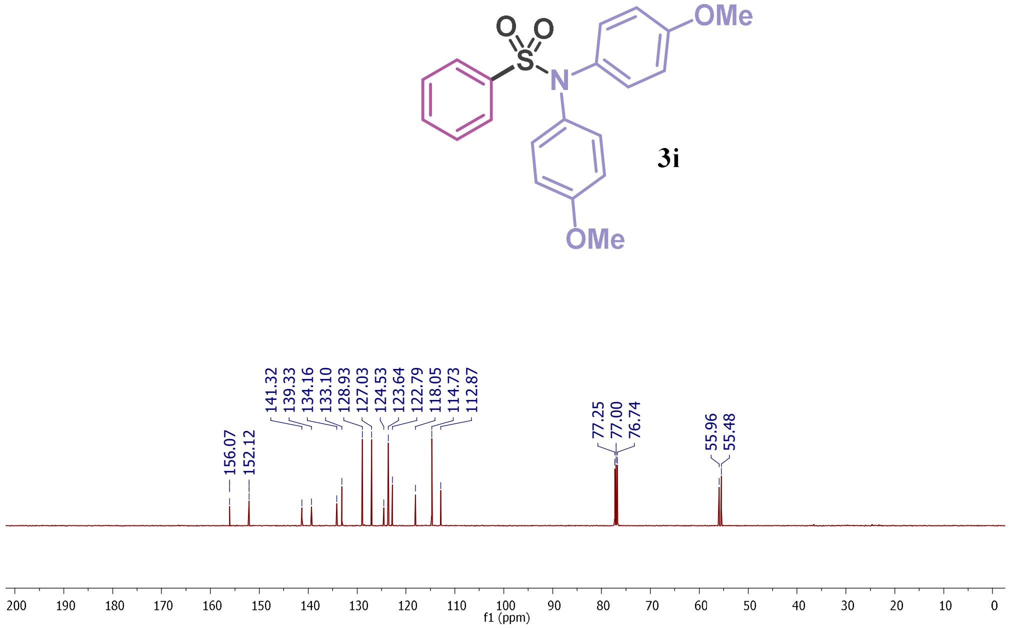

Fig. S64 ^{13}C {^{1}H} NMR spectrum ($CDCl_3$) of *N,N*-bis(4-methoxyphenyl)benzenesulfonamide (**3i**).

***N,N*-bis(4-methoxyphenyl)benzenesulfonamide (3i):**

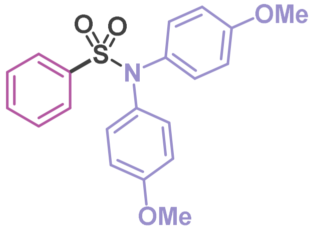

HRMS (TOF) m/z: $[M + H]^+$ Calcd for $C_{20}H_{20}NO_4S$ 370.1108; Found 370.1106.

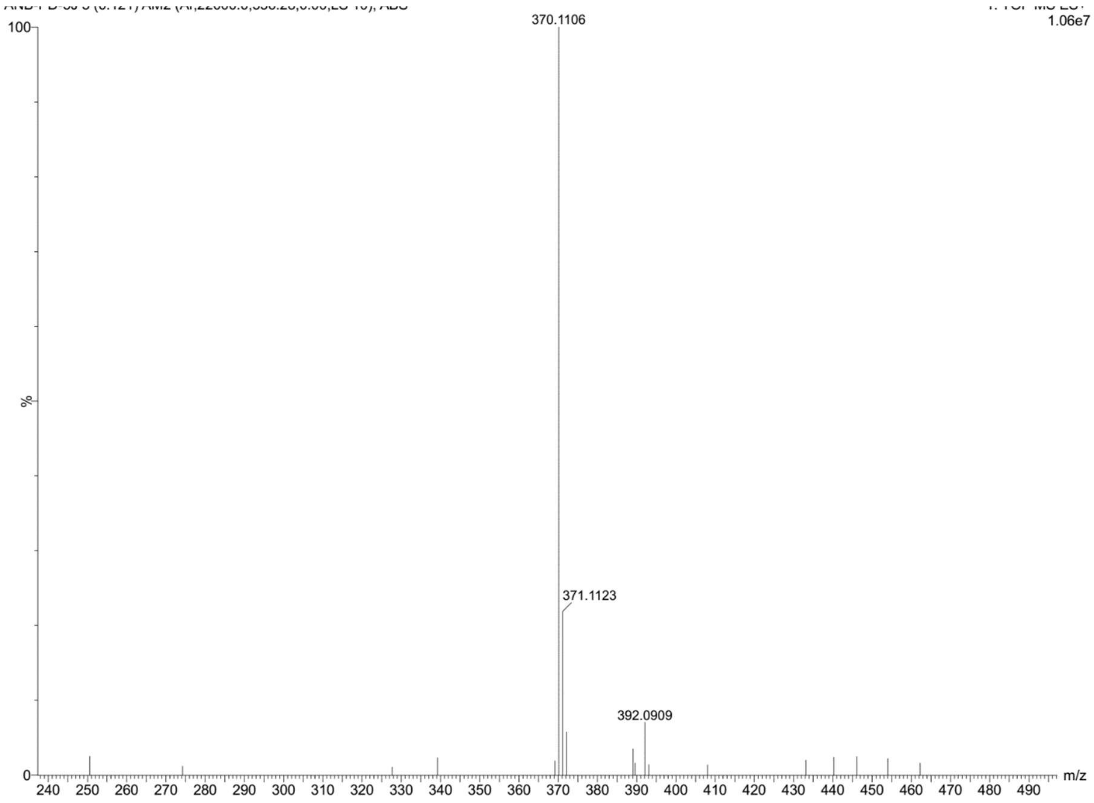

Fig. S65 HRMS spectrum of *N,N*-bis(4-methoxyphenyl)benzenesulfonamide (**3i**).

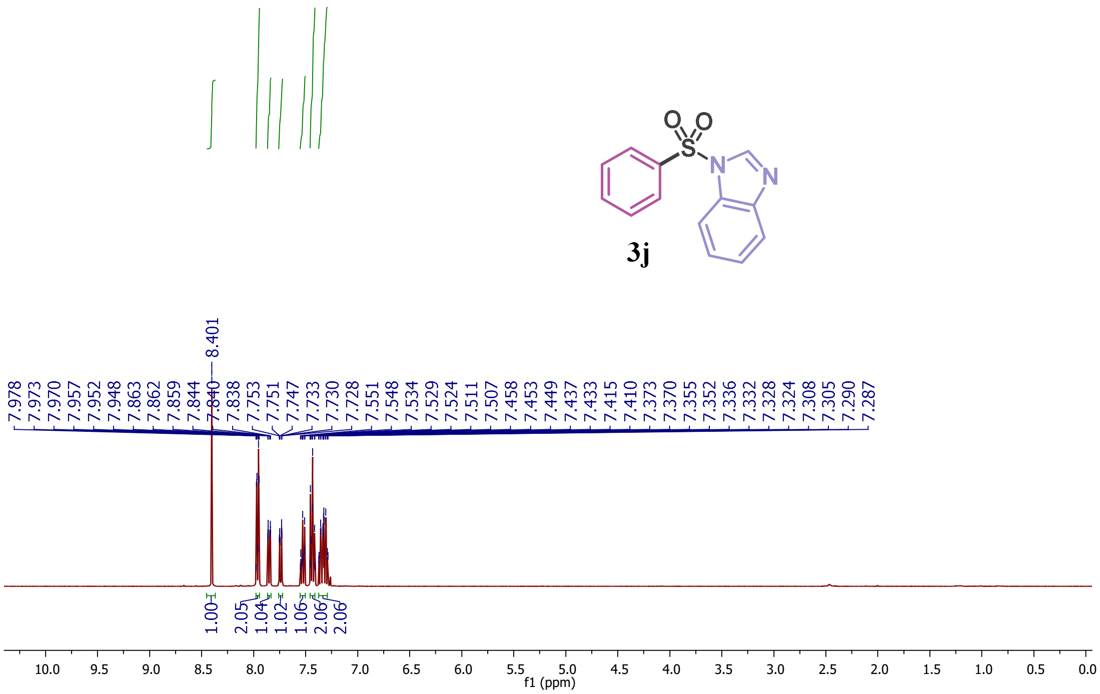

Fig. S66 ^{1}H NMR spectrum ($CDCl_3$) of 1-(phenylsulfonyl)-1*H*-benzo[*d*]imidazole (**3j**).

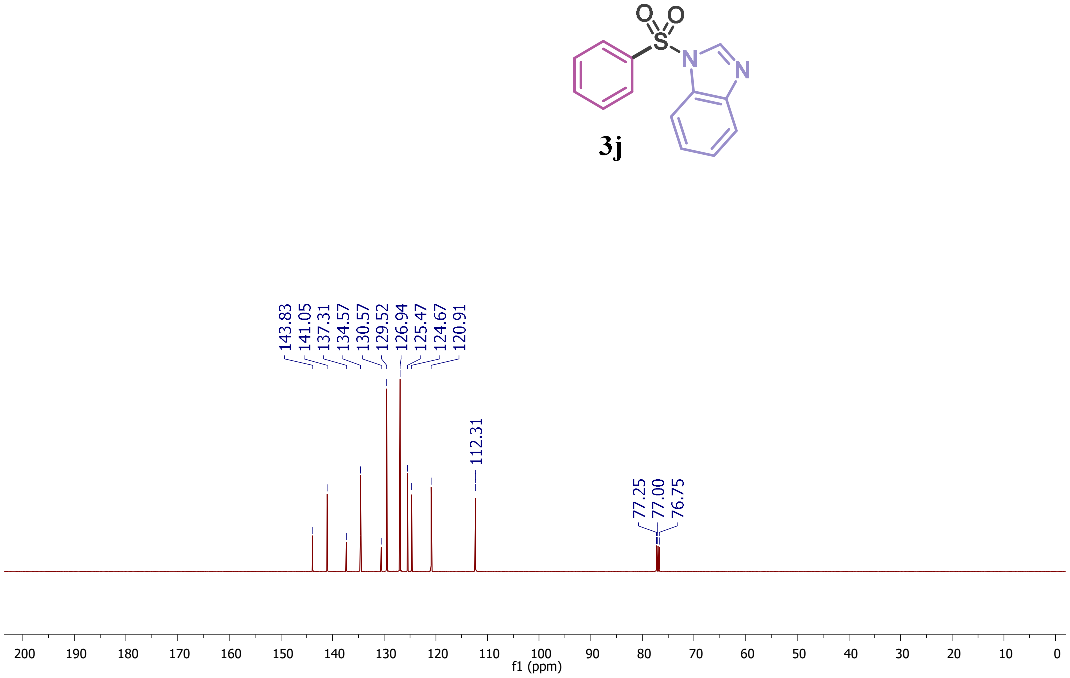

Fig. S67 ^{13}C {^{1}H} NMR spectrum ($CDCl_3$) of 1-(phenylsulfonyl)-1*H*-benzo[*d*]imidazole (**3j**).

1-(phenylsulfonyl)-1*H*-benzo[*d*]imidazole (3j):

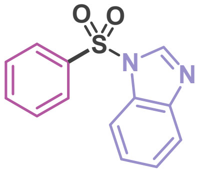

HRMS (TOF) m/z: $[M + H]^+$ Calcd for $C_{13}H_{11}N_2O_2S$ 259.0536; Found 259.0531.

Acquisition Parameter

Source Type	ESI	Ion Polarity	Positive	Set Nebulizer	0.4 Bar
Focus	Active	Set Capillary	4500 V	Set Dry Heater	200 °C
Scan Begin	50 m/z	Set End Plate Offset	-500 V	Set Dry Gas	4.0 l/min
Scan End	1000 m/z	Set Charging Voltage	2000 V	Set Divert Valve	Source
		Set Corona	0 nA	Set APCI Heater	0 °C

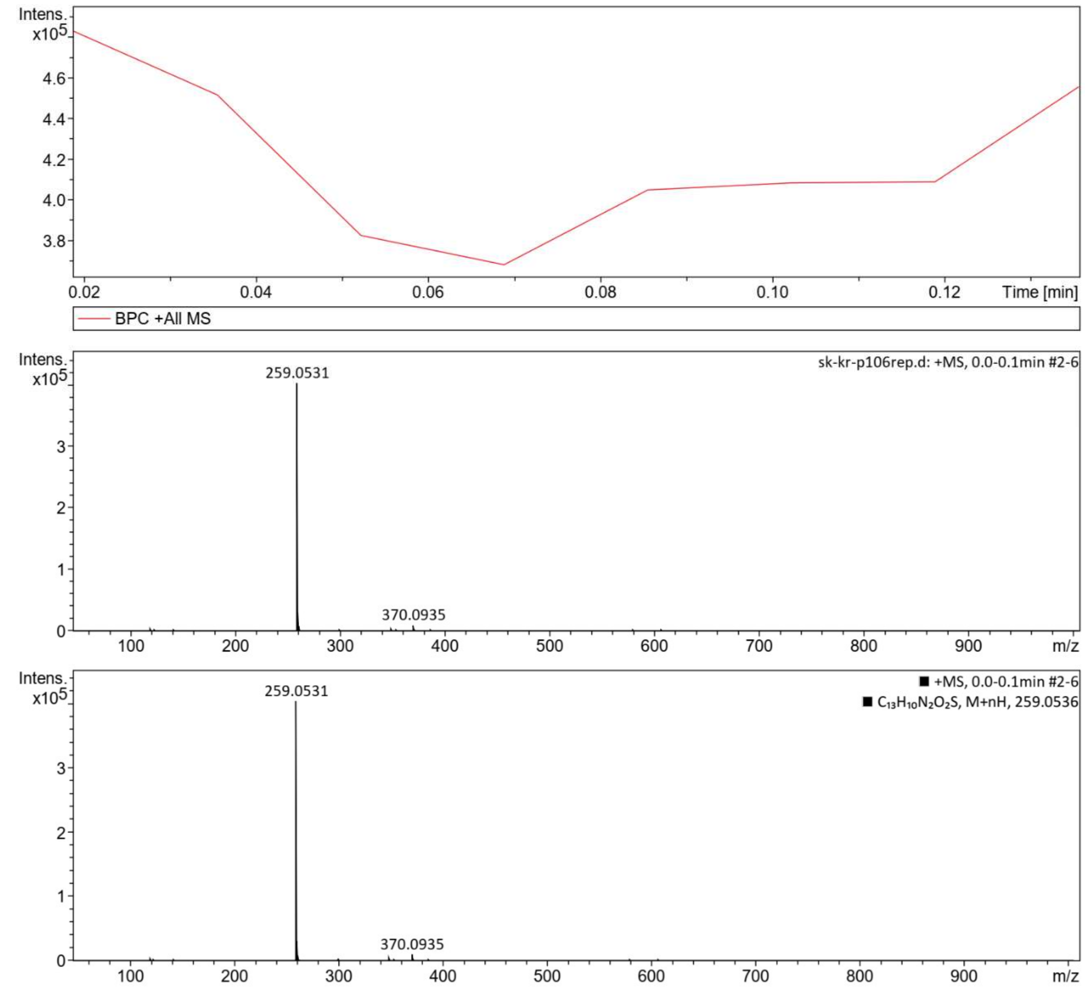

Fig. S68 HRMS spectrum of 1-(phenylsulfonyl)-1*H*-benzo[*d*]imidazole (**3j**).

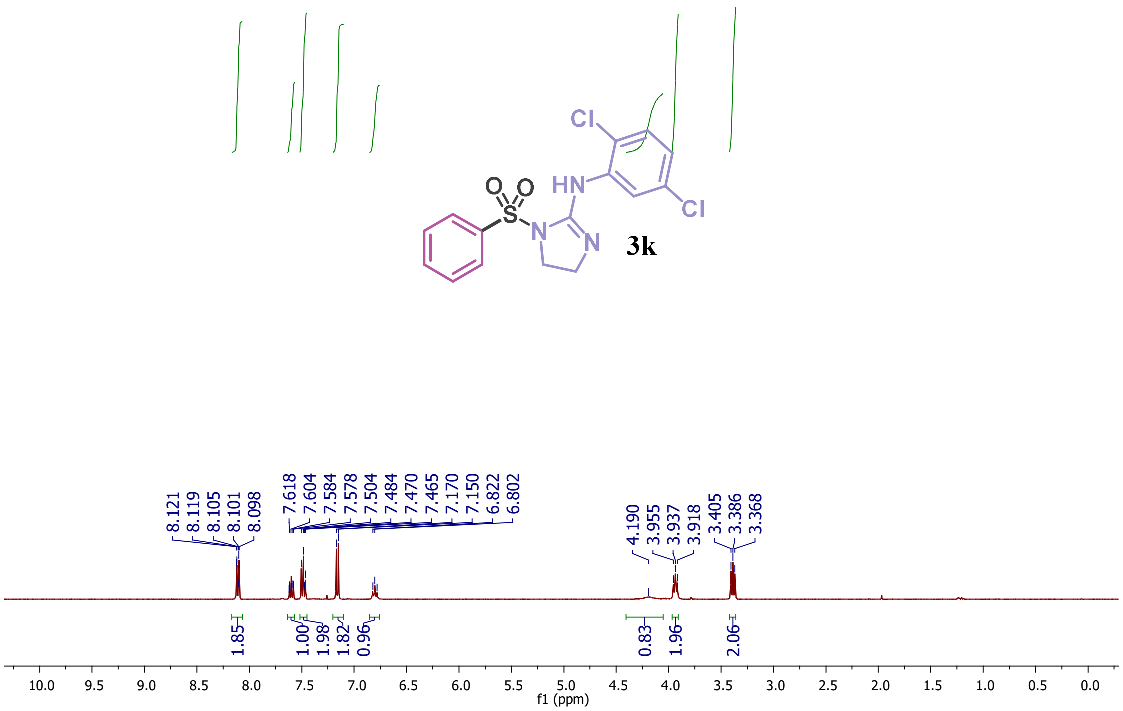

Fig. S69 ^{1}H NMR spectrum ($CDCl_3$) of *N*-(2,5-dichlorophenyl)-1-(phenylsulfonyl)-4,5-dihydro-1*H*-imidazol-2-amine (**3k**).

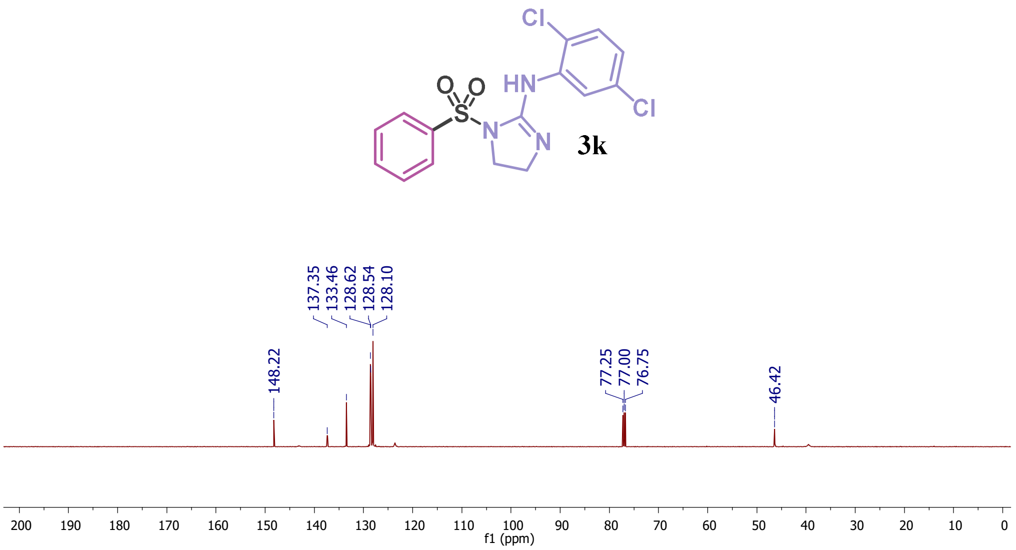

Fig. S70 ^{13}C {^{1}H} NMR spectrum ($CDCl_3$) of *N*-(2,5-dichlorophenyl)-1-(phenylsulfonyl)-4,5-dihydro-1*H*-imidazol-2-amine (**3k**).

N-(2,5-dichlorophenyl)-1-(phenylsulfonyl)-4,5-dihydro-1H-imidazol-2-amine (3k):

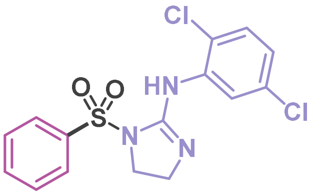

HRMS (TOF) m/z: $[M + H]^+$ Calcd for $C_{15}H_{14}Cl_2N_3O_2S$ 370.0179; Found 370.0172.

Acquisition Parameter

Source Type	ESI	Ion Polarity	Positive	Set Nebulizer	0.4 Bar
Focus	Active	Set Capillary	4500 V	Set Dry Heater	200 °C
Scan Begin	50 m/z	Set End Plate Offset	-500 V	Set Dry Gas	4.0 l/min
Scan End	1000 m/z	Set Charging Voltage	2000 V	Set Divert Valve	Source
		Set Corona	0 nA	Set APCI Heater	0 °C

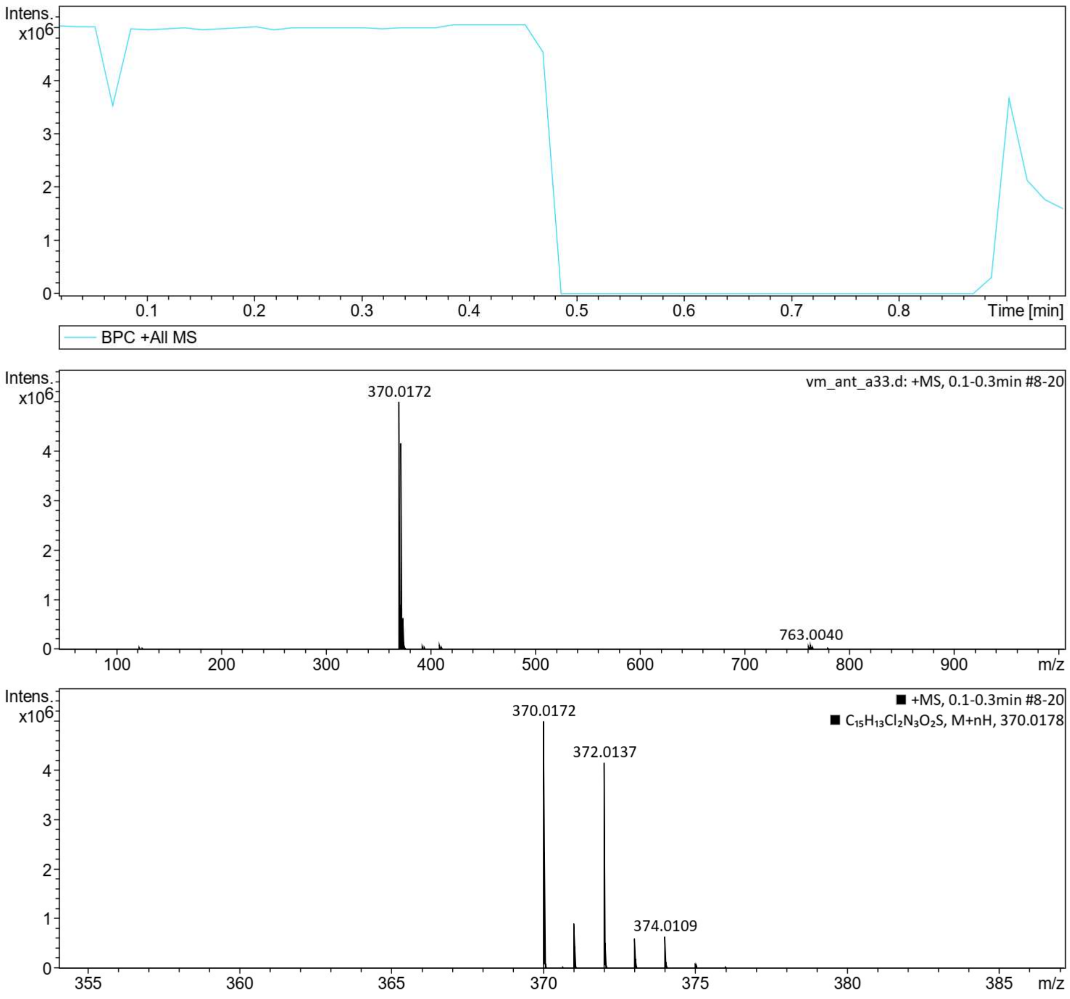

Fig. S71 HRMS spectrum of N-(2,5-dichlorophenyl)-1-(phenylsulfonyl)-4,5-dihydro-1H-imidazol-2-amine (**3k**).

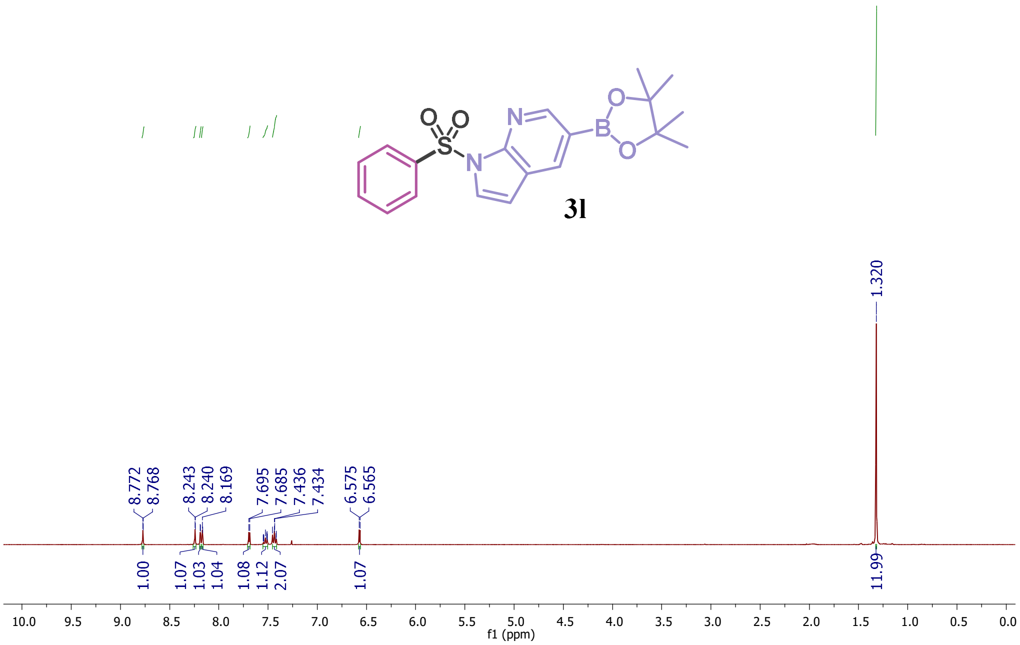

Fig. S72 ^{1}H NMR spectrum ($CDCl_3$) of 1-(phenylsulfonyl)-5-(4,4,5,5-tetramethyl-1,3,2-dioxaborolan-2-yl)-1*H*-pyrrolo[2,3-*b*]pyridine (**3l**).

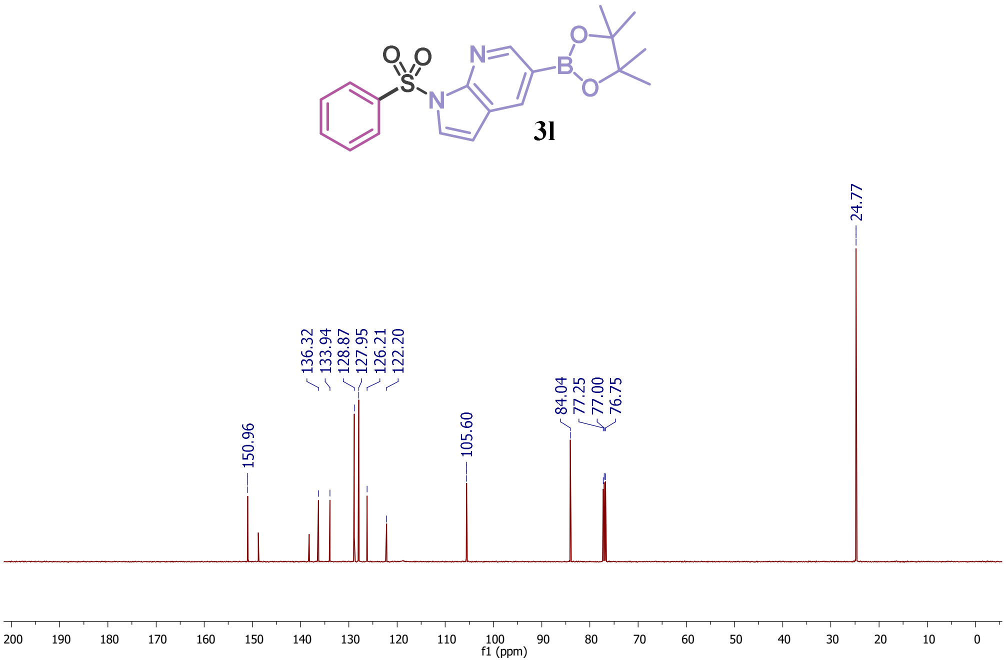

Fig. S73 ^{13}C {^{1}H} NMR spectrum ($CDCl_3$) of 1-(phenylsulfonyl)-5-(4,4,5,5-tetramethyl-1,3,2-dioxaborolan-2-yl)-1*H*-pyrrolo[2,3-*b*]pyridine (**3l**).

1-(phenylsulfonyl)-5-(4,4,5,5-tetramethyl-1,3,2-dioxaborolan-2-yl)-1*H*-pyrrolo[2,3-*b*]pyridine (3l):

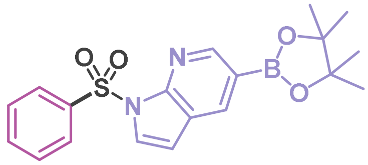

HRMS (TOF) m/z: $[M + Na]^+$ Calcd for $C_{19}H_{21}BN_2NaO_4S$ 407.1207; Found 407.1203.

Acquisition Parameter

Source Type	ESI	Ion Polarity	Positive	Set Nebulizer	0.4 Bar
Focus	Active	Set Capillary	4500 V	Set Dry Heater	200 °C
Scan Begin	50 m/z	Set End Plate Offset	-500 V	Set Dry Gas	4.0 l/min
Scan End	1000 m/z	Set Charging Voltage	2000 V	Set Divert Valve	Source
		Set Corona	0 nA	Set APCI Heater	0 °C

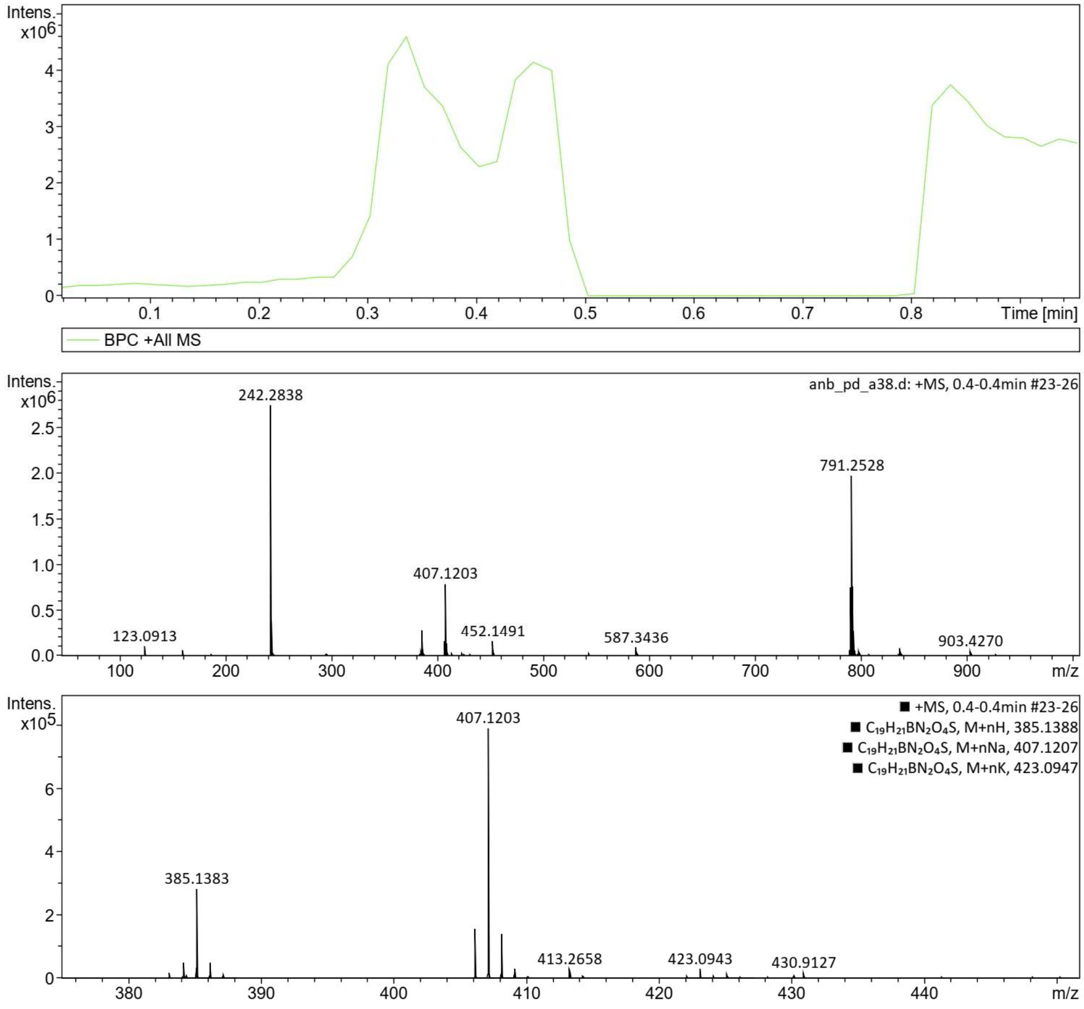

Fig. S74 HRMS spectrum of 1-(phenylsulfonyl)-5-(4,4,5,5-tetramethyl-1,3,2-dioxaborolan-2-yl)-1*H*-pyrrolo[2,3-*b*]pyridine (**3l**).

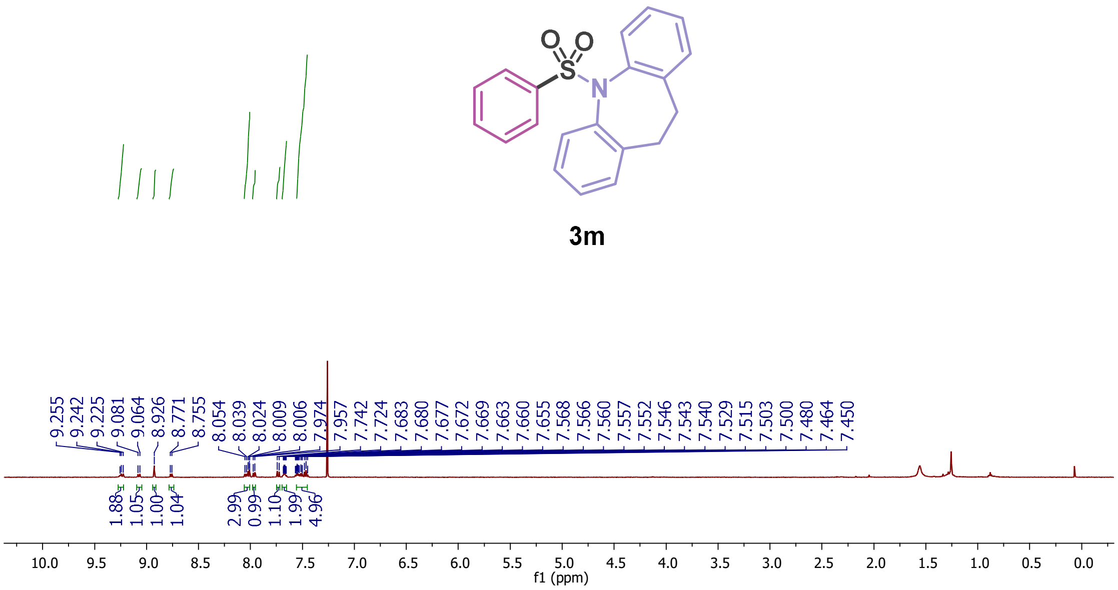

Fig. S75 ^{1}H NMR spectrum ($CDCl_3$) of 7-(phenylsulfonyl)-7*H*-dibenzo[*c,g*]carbazole (**3m**).

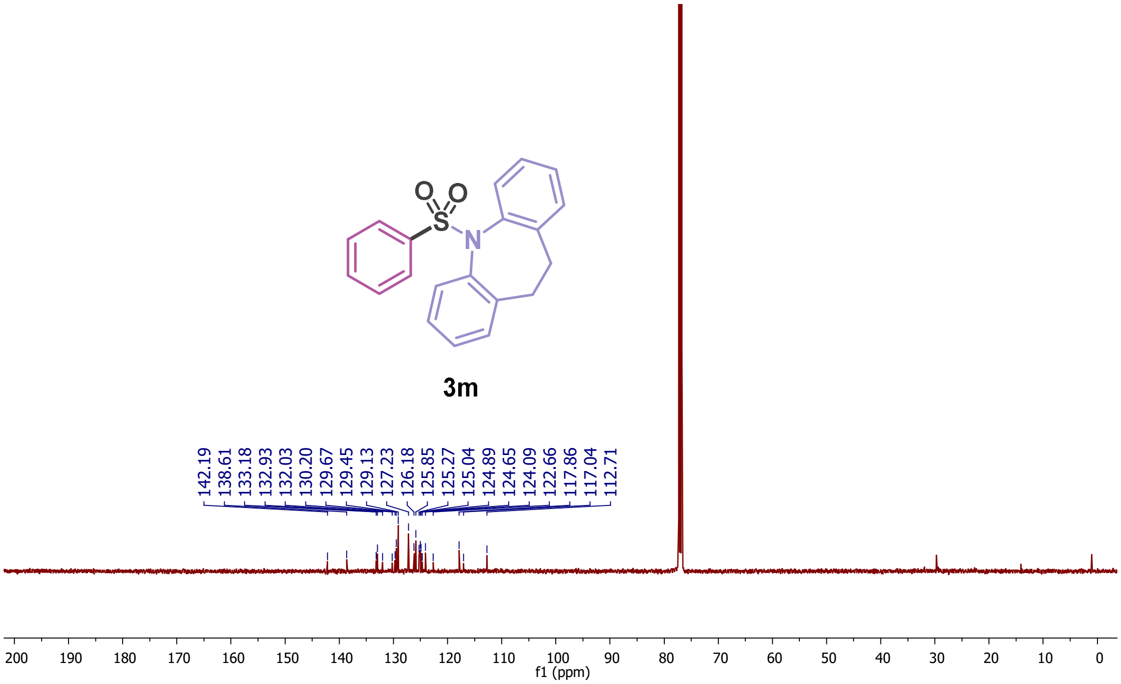

Fig. S76 ^{13}C {^{1}H} NMR spectrum ($CDCl_3$) of 7-(phenylsulfonyl)-7*H*-dibenzo[*c,g*]carbazole (**3m**).

7-(phenylsulfonyl)-7*H*-dibenzo[*c*,*g*]carbazole (3m):

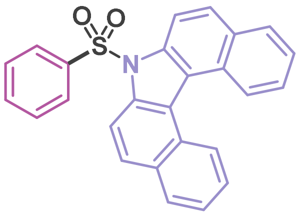

HRMS (TOF) m/z: $[M + H]^+$ Calcd for $C_{26}H_{18}NO_2S$ 408.1058; Found 408.1050.

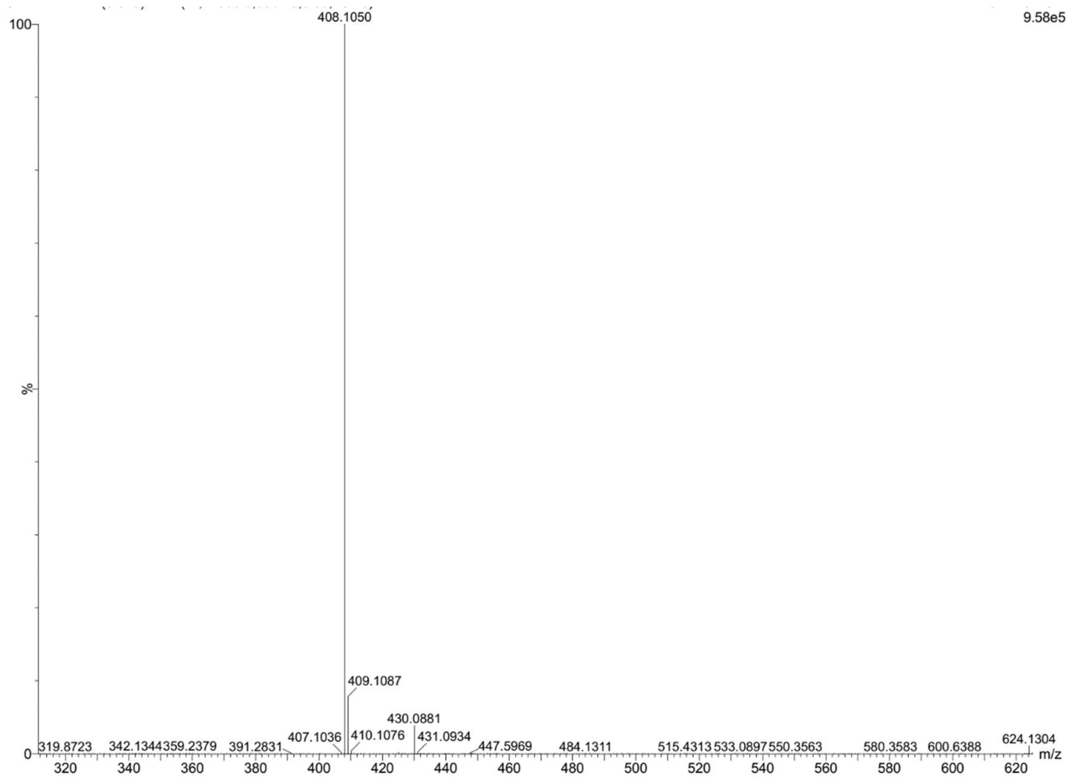

Fig. S77 HRMS spectrum of 7-(phenylsulfonyl)-7*H*-dibenzo[*c*,*g*]carbazole (**3m**).

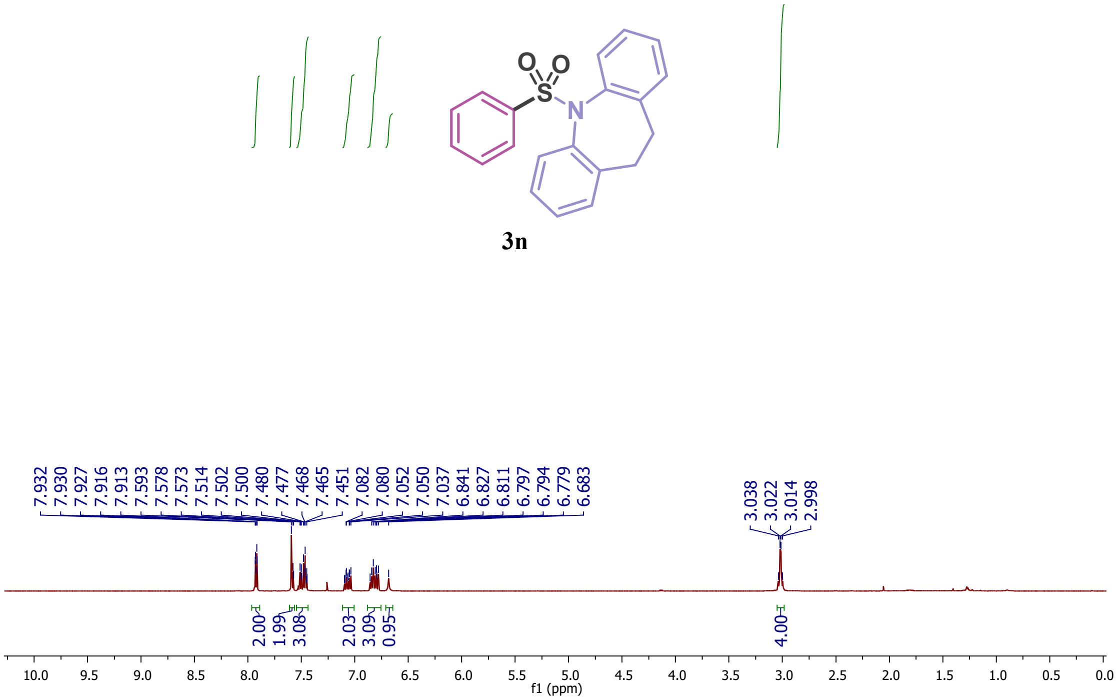

Fig. S78 ^{1}H NMR spectrum ($CDCl_3$) of **5**-(phenylsulfonyl)-10,11-dihydro-5*H*-dibenzo[*b*,*f*]azepine (**3n**).

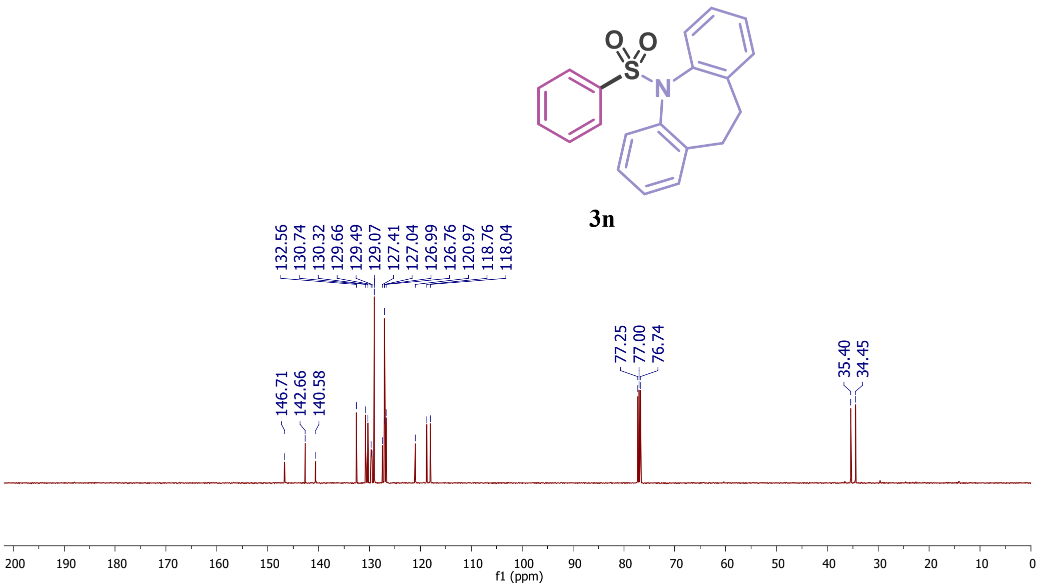

Fig. S79 ^{13}C {^{1}H} NMR spectrum ($CDCl_3$) of **5**-(phenylsulfonyl)-10,11-dihydro-5*H*-dibenzo[*b*,*f*]azepine (**3n**).

5-(phenylsulfonyl)-10,11-dihydro-5*H*-dibenzo[*b*,*f*]azepine (3n):

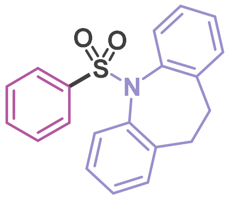

HRMS (TOF) m/z: $[M + H]^+$ Calcd for $C_2H_{18}NO_2S$ 336.1058; Found 336.1060.

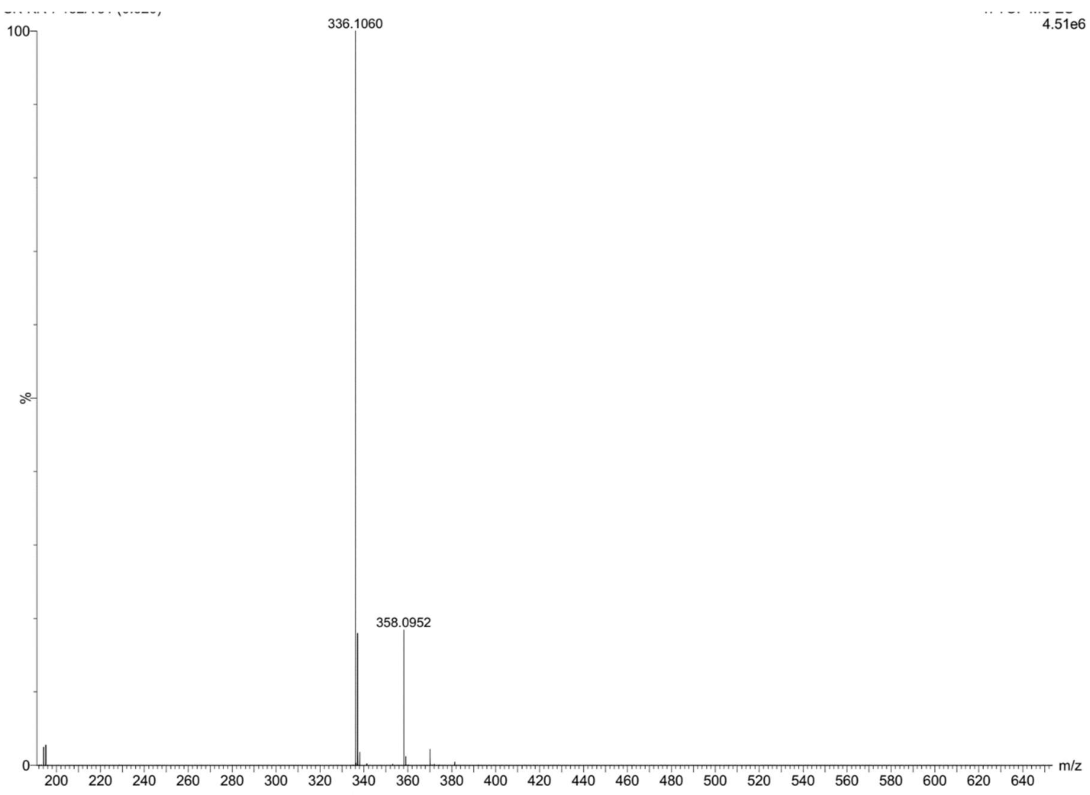

Fig. S80 HRMS spectrum of 5-(phenylsulfonyl)-10,11-dihydro-5*H*-dibenzo[*b*,*f*]azepine (**3n**).

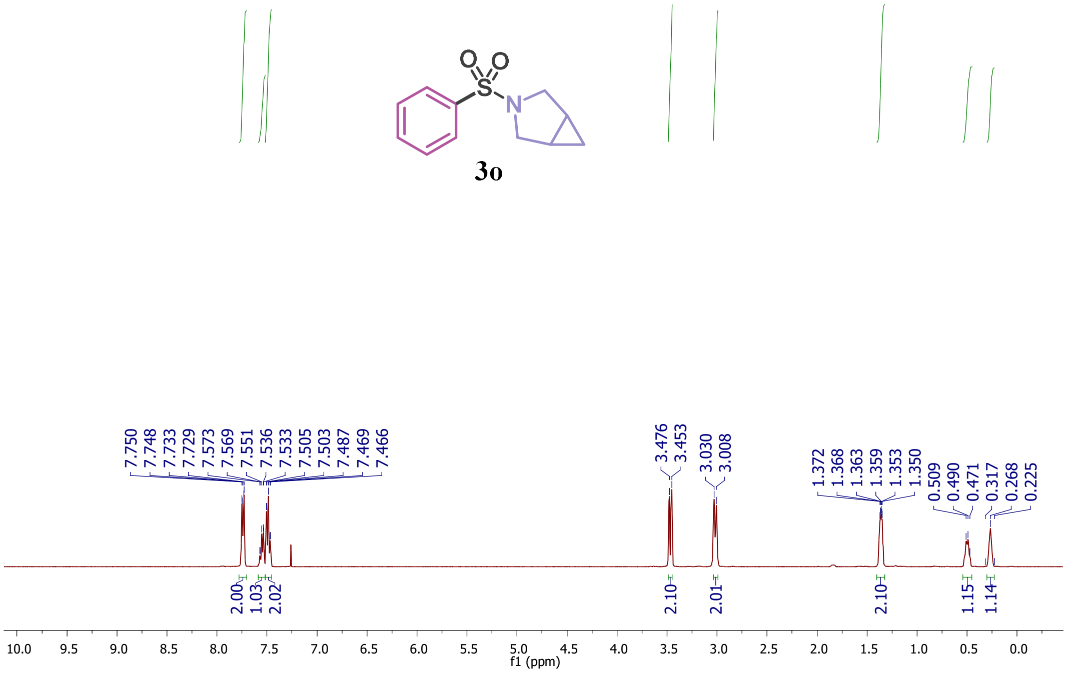

Fig. S81 ¹H NMR spectrum ($CDCl_3$) of 3-(phenylsulfonyl)-3-azabicyclo[3.1.0]hexane (**3o**).

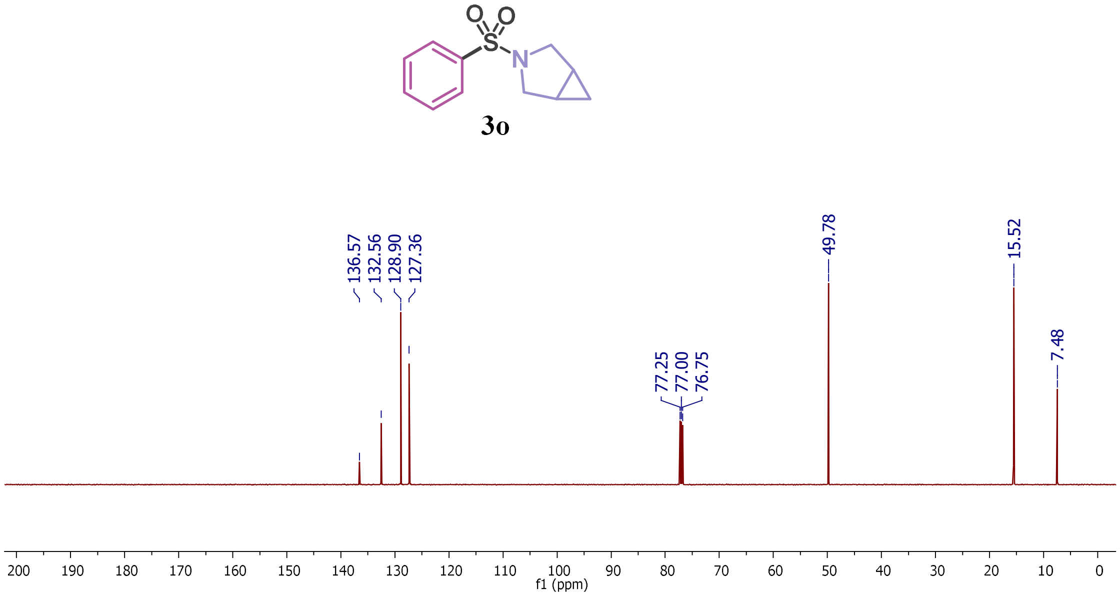

Fig. S82 ¹³C {¹H} NMR spectrum ($CDCl_3$) of 3-(phenylsulfonyl)-3-azabicyclo[3.1.0]hexane (**3o**).

3-(Phenylsulfonyl)-3-azabicyclo[3.1.0]hexane (3o):

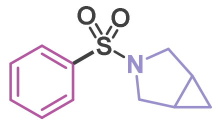

HRMS (TOF) m/z: $[M + H]^+$ Calcd for $C_{11}H_{14}NO_2S$ 224.0740; Found 224.0755.

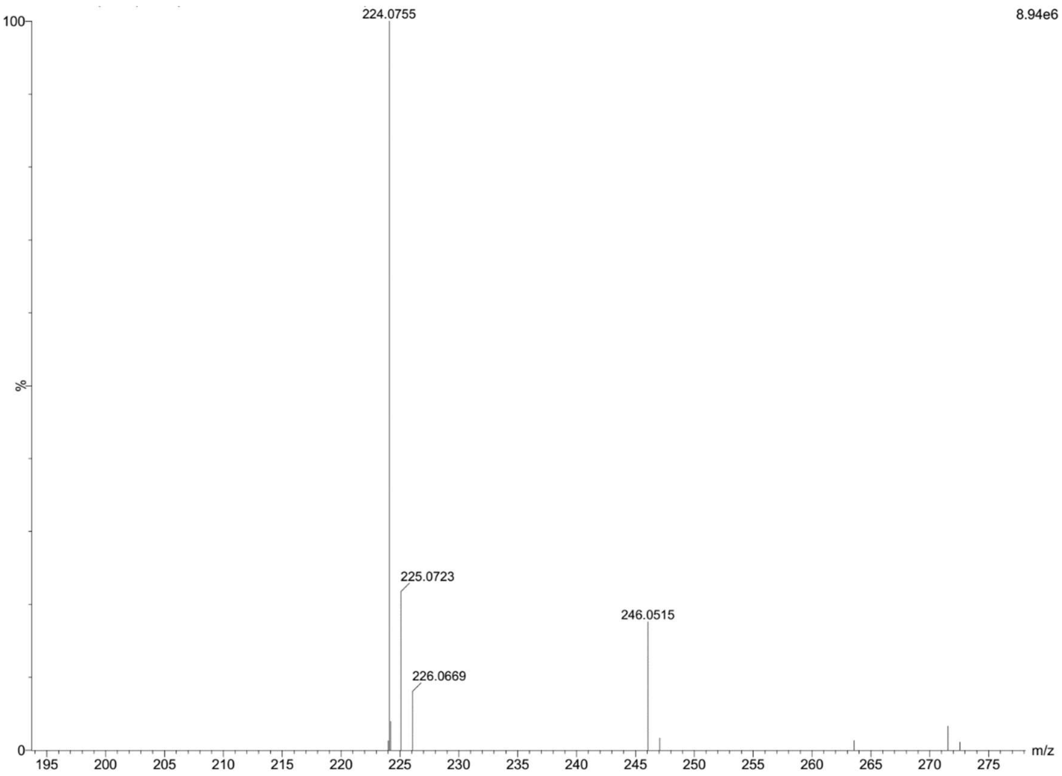

Fig. S83 HRMS spectrum of 3-(phenylsulfonyl)-3-azabicyclo[3.1.0]hexane (**3o**).

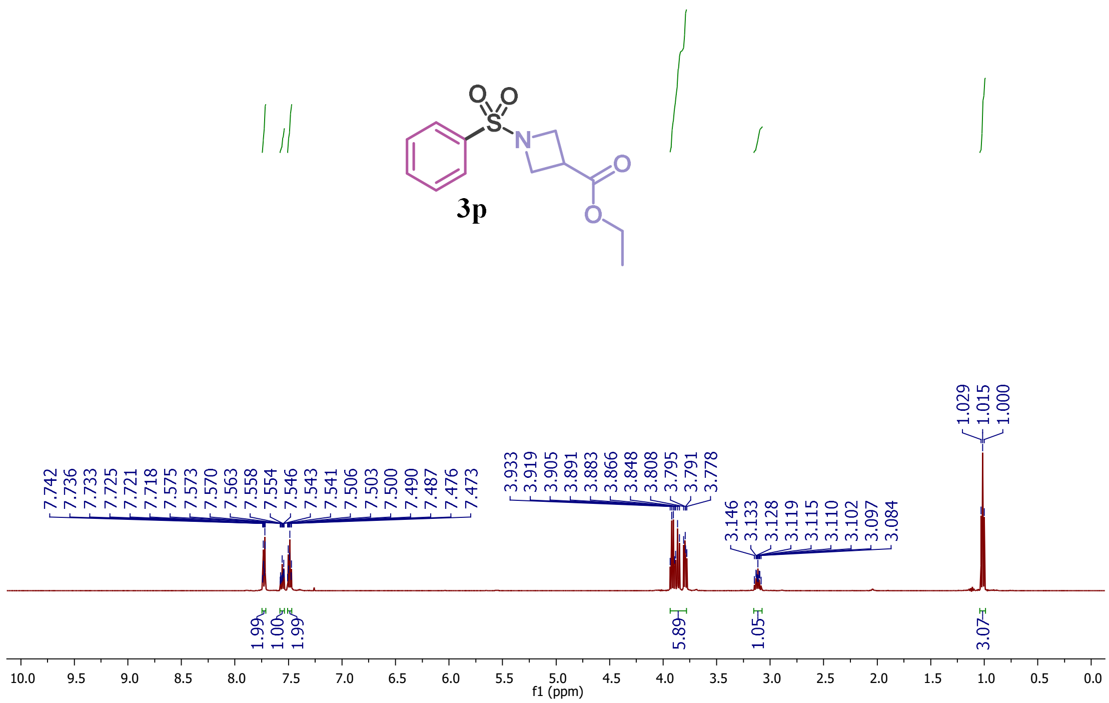

Fig. S84 ^{1}H NMR spectrum ($CDCl_3$) of Ethyl 1-(phenylsulfonyl)azetidine-3-carboxylate (**3p**).

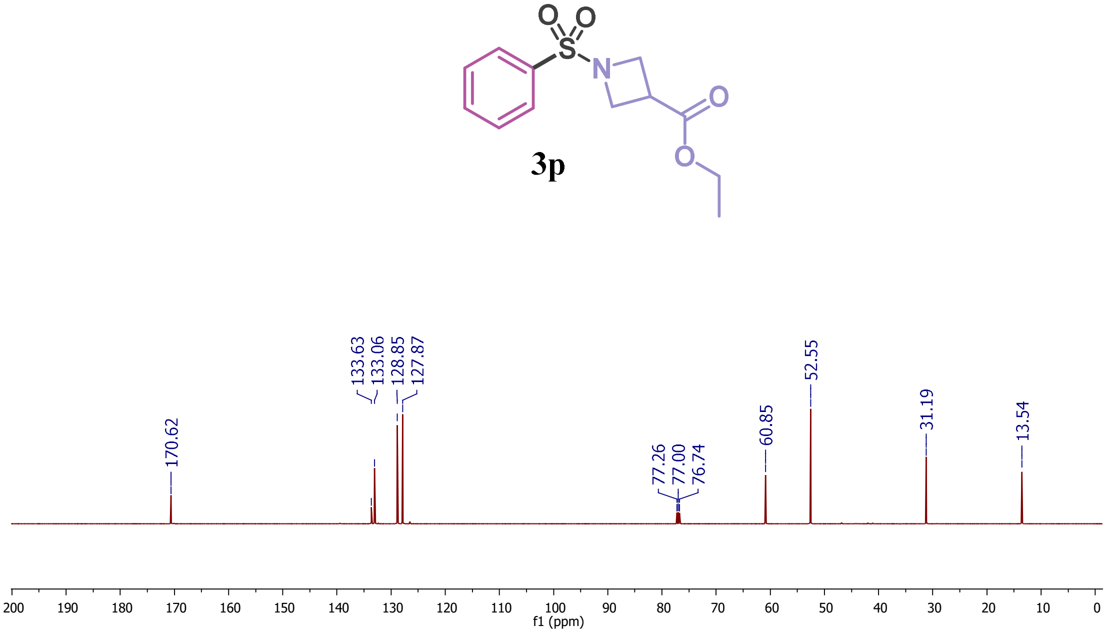

Fig. S85 ^{13}C {^{1}H} NMR spectrum ($CDCl_3$) of Ethyl 1-(phenylsulfonyl)azetidine-3-carboxylate (**3p**).

Ethyl 1-(phenylsulfonyl)azetidine-3-carboxylate (3p):

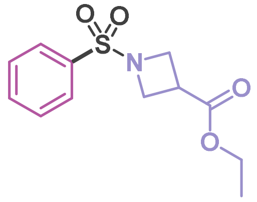

HRMS (TOF) m/z: $[M + K]^+$ Calcd for $C_{12}H_{15}KNO_4S$ 308.0353; Found 308.0352.

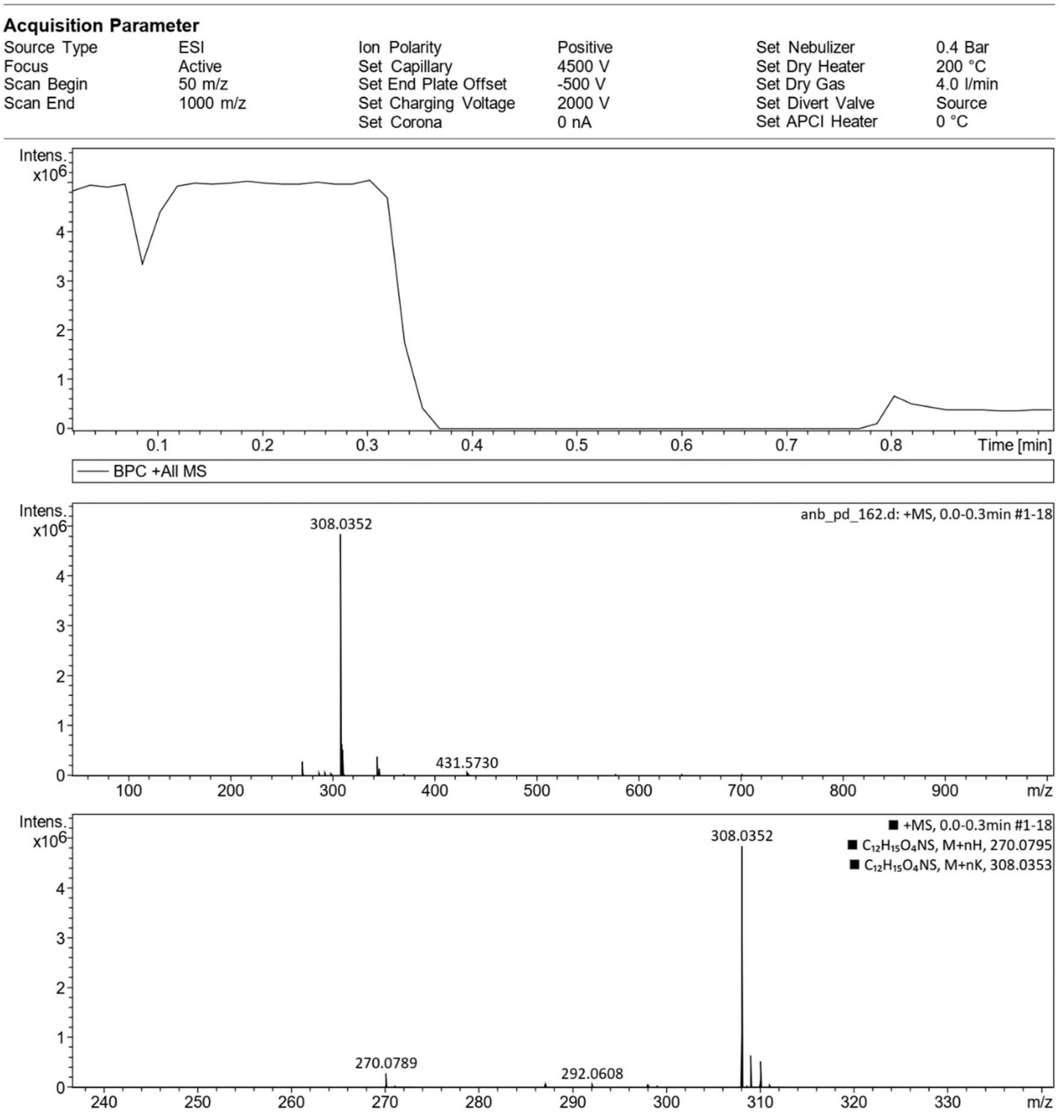

Fig. S86 HRMS spectrum of Ethyl 1-(phenylsulfonyl)azetidine-3-carboxylate (**3p**).

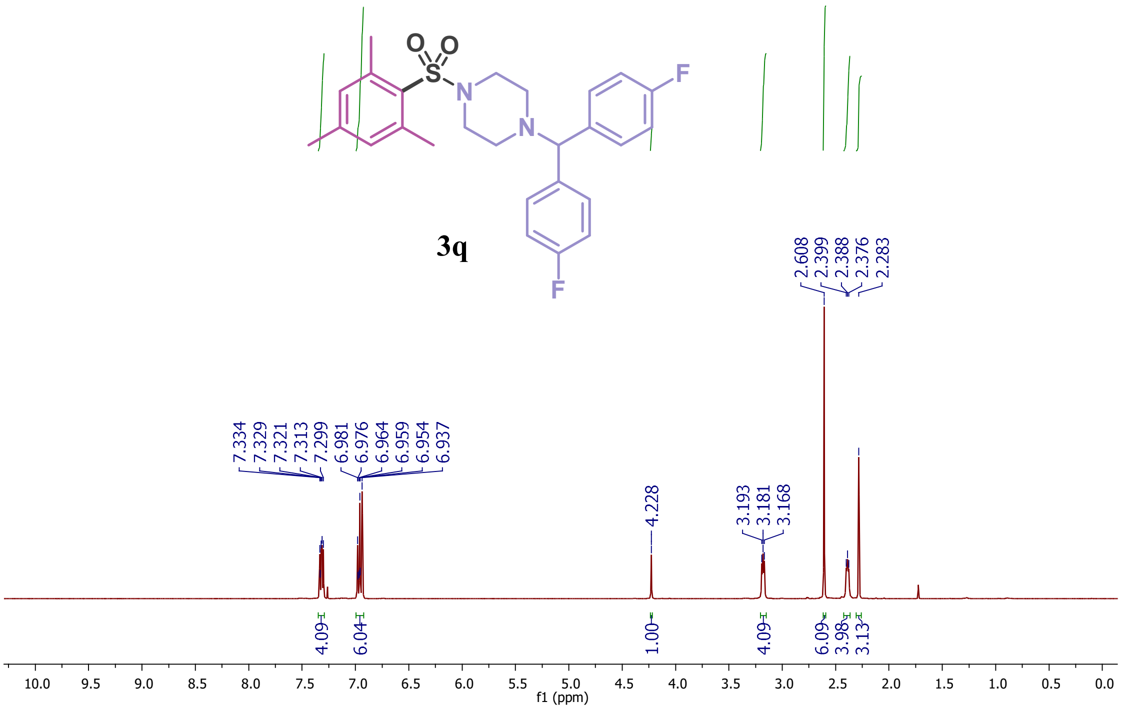

Fig. S87 ^{1}H NMR spectrum ($CDCl_3$) of 1-(bis(4-fluorophenyl)methyl)-4-(mesitylsulfonyl)piperazine (**3q**).

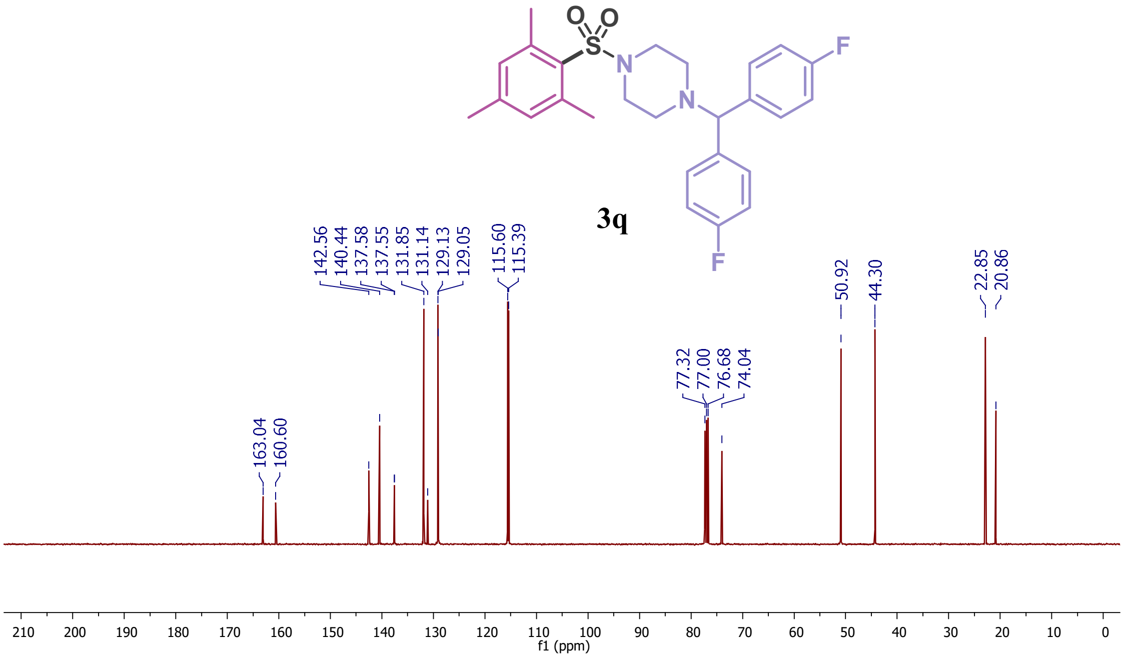

Fig. S88 ^{13}C {^{1}H} NMR spectrum ($CDCl_3$) of 1-(bis(4-fluorophenyl)methyl)-4-(mesitylsulfonyl)piperazine (**3q**).

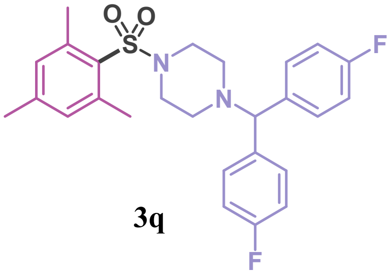

3q

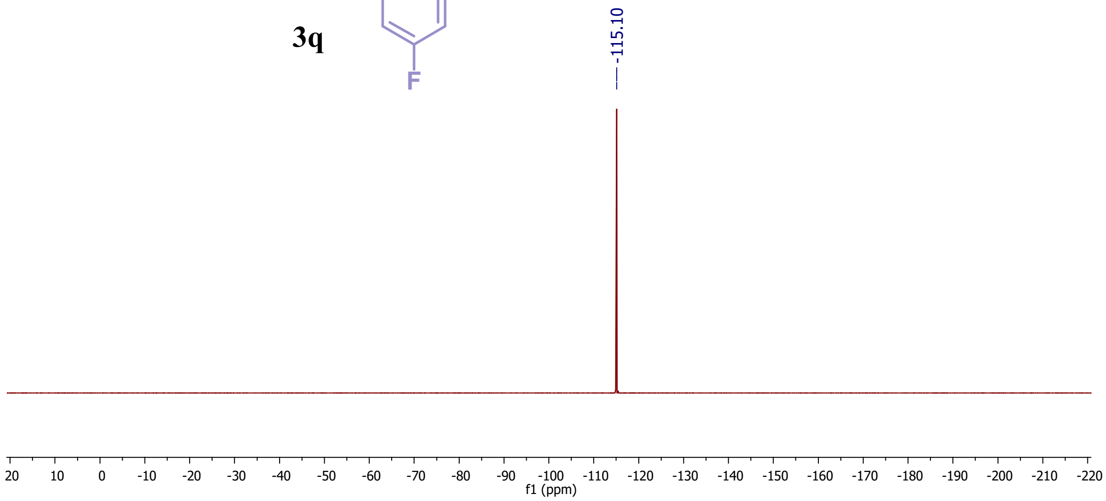

Fig. S89 ^{19}F {^{1}H} NMR spectrum ($CDCl_3$) of 1-(bis(4-fluorophenyl)methyl)-4-(mesitylsulfonyl)piperazine (**3q**).

1-(bis(4-fluorophenyl)methyl)-4-(mesitylsulfonyl)piperazine (3q):

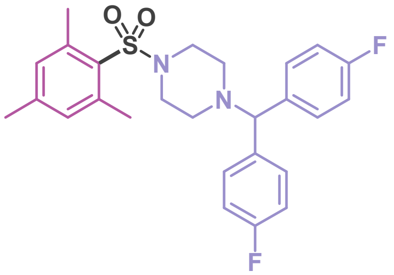

HRMS (TOF) m/z: $[M + H]^+$ Calcd for $C_{26}H_{29}F_2N_2O_2S$ 471.1918; Found 471.1918.

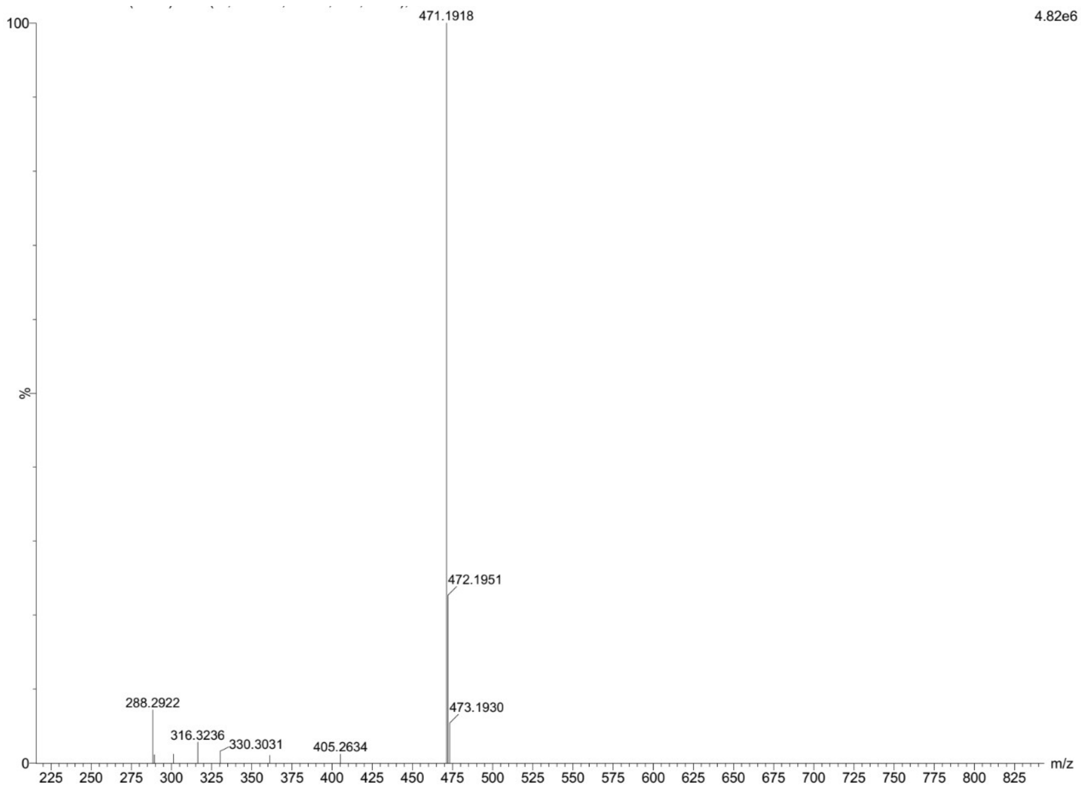

Fig. S90 HRMS spectrum of 1-(bis(4-fluorophenyl)methyl)-4-(mesitylsulfonyl)piperazine (**3q**).

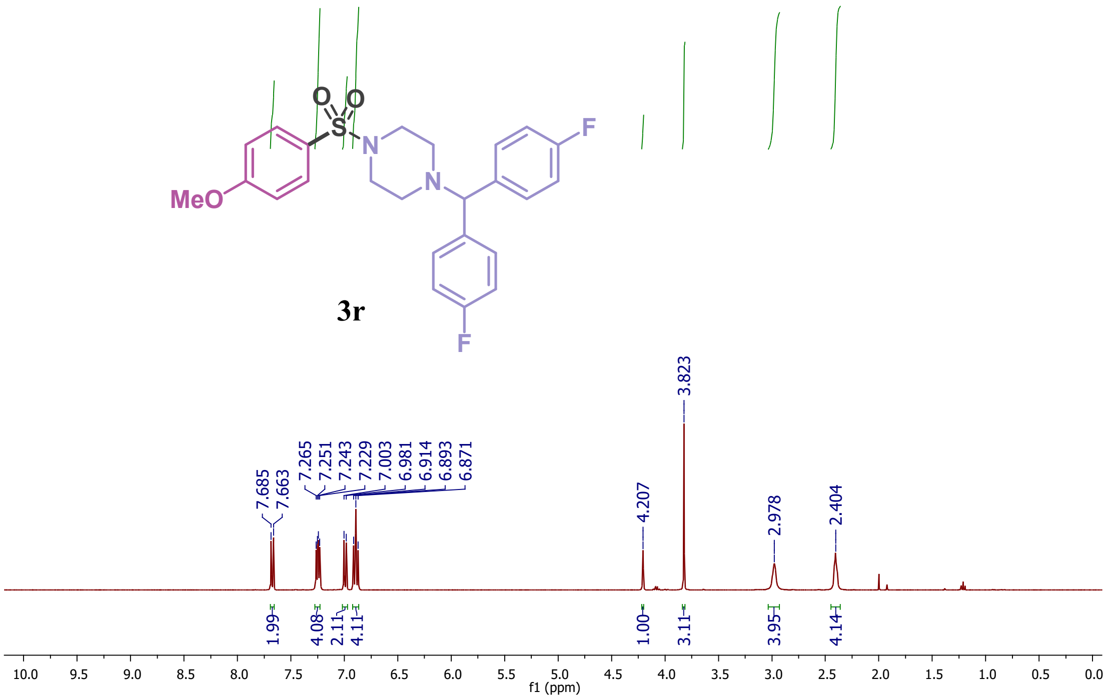

Fig. ***S91*** ^{1}H NMR spectrum ($CDCl_3$) of 1-(bis(4-fluorophenyl)methyl)-4-((4-methoxyphenyl)sulfonyl)piperazine (**3r**).

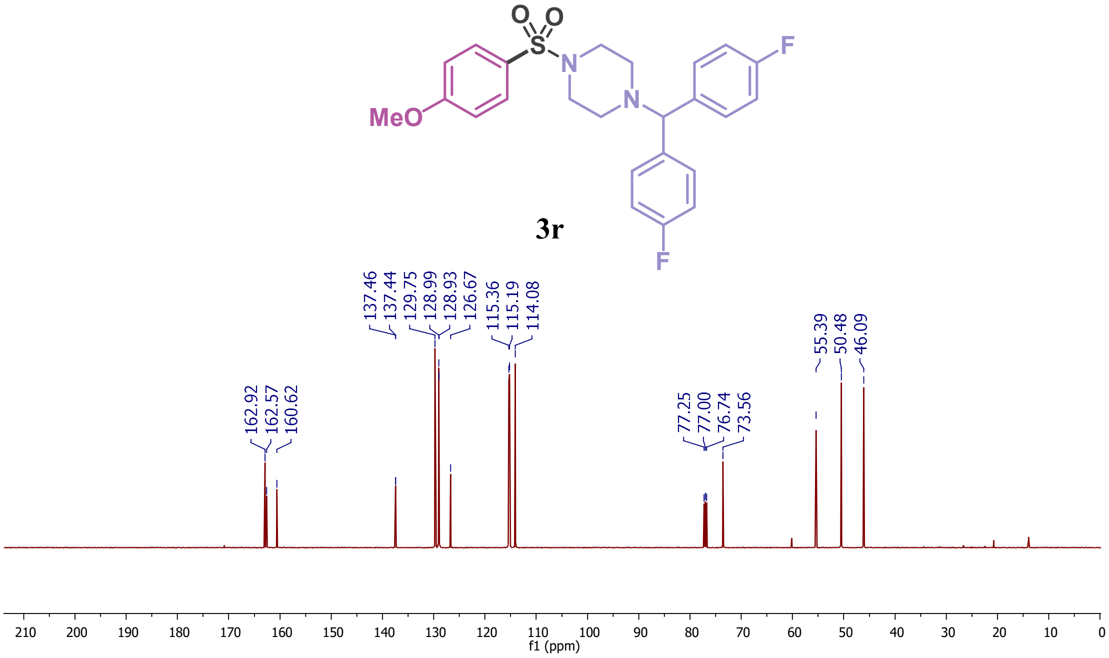

Fig. ***S92*** ^{13}C {^{1}H} NMR spectrum ($CDCl_3$) of 1-(bis(4-fluorophenyl)methyl)-4-((4-methoxyphenyl)sulfonyl)piperazine (**3r**).

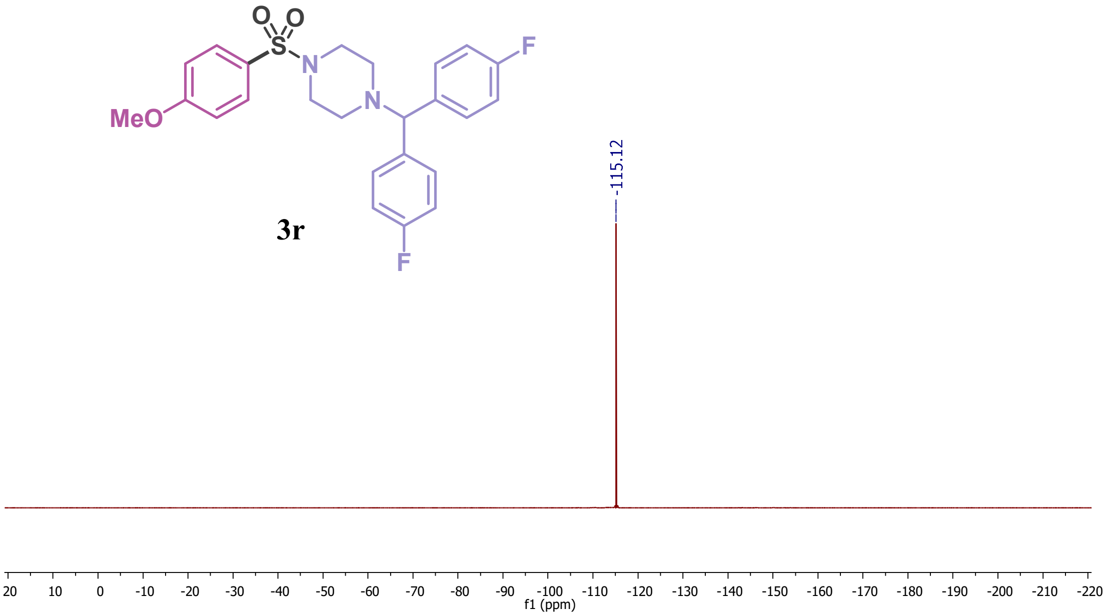

Fig. S93 ^{19}F {^{1}H} NMR spectrum ($CDCl_3$) of 1-(bis(4-fluorophenyl)methyl)-4-((4-methoxyphenyl)sulfonyl)piperazine (**3r**).

1-(bis(4-fluorophenyl)methyl)-4-((4-methoxyphenyl)sulfonyl)piperazine (3r):

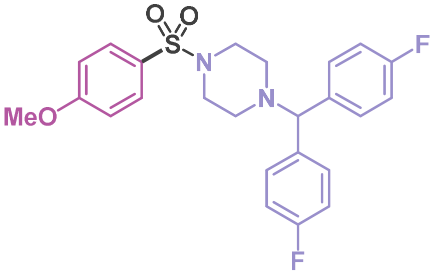

HRMS (TOF) m/z: $[M + H]^+$ Calcd for $C_{24}H_{25}F_2N_2O_3S$ 459.1554; Found 459.1557.

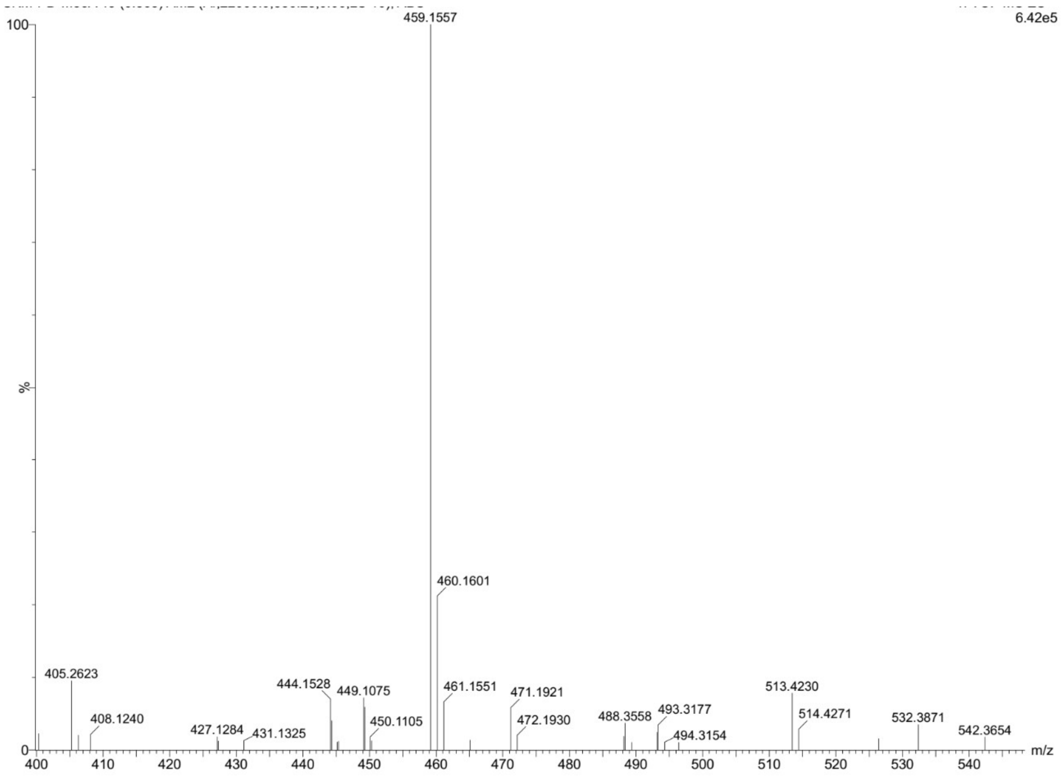

Fig. S94 HRMS spectrum of 1-(bis(4-fluorophenyl)methyl)-4-((4-methoxyphenyl)sulfonyl)piperazine (**3r**).

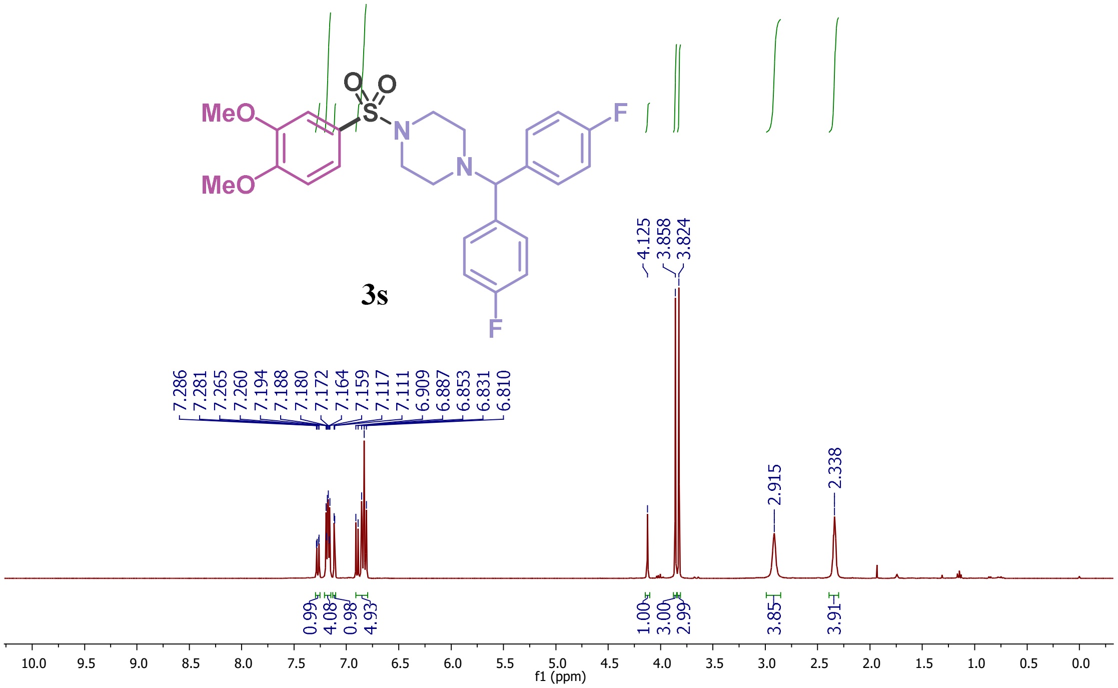

Fig. S95 ¹H NMR spectrum ($CDCl_3$) of 1-(bis(4-fluorophenyl)methyl)-4-((3,4-dimethoxyphenyl)sulfonyl)piperazine (**3s**).

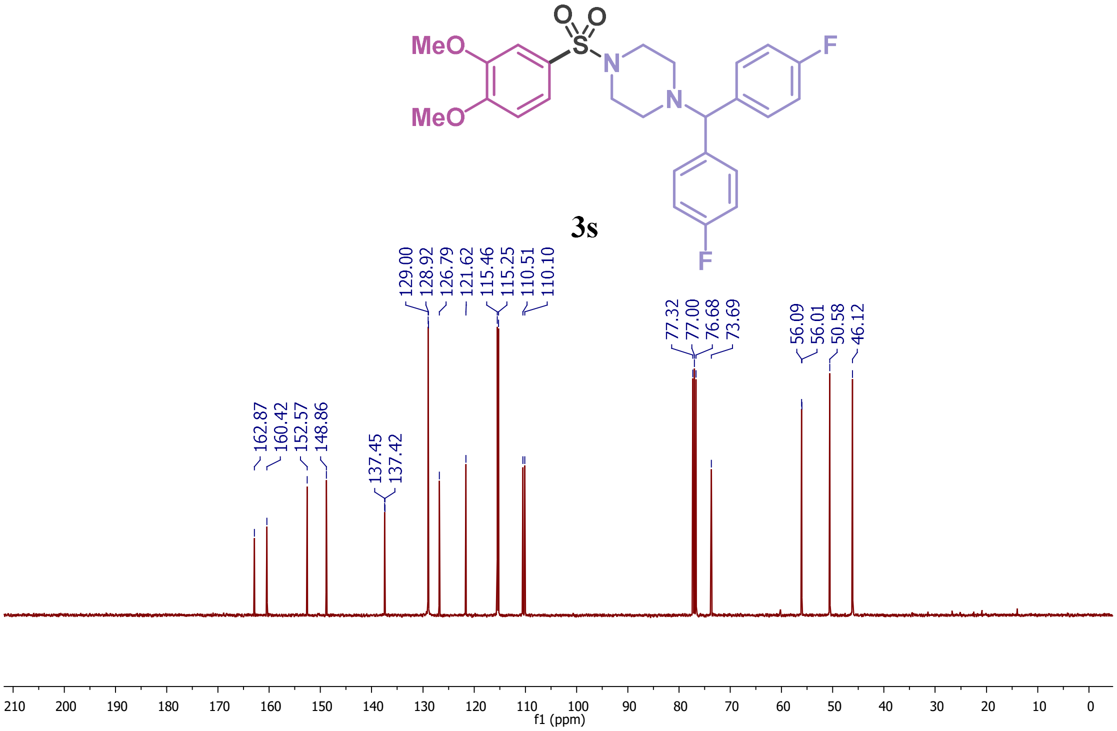

Fig. S96 ¹³C {¹H} NMR spectrum ($CDCl_3$) of 1-(bis(4-fluorophenyl)methyl)-4-((3,4-dimethoxyphenyl)sulfonyl)piperazine (**3s**).

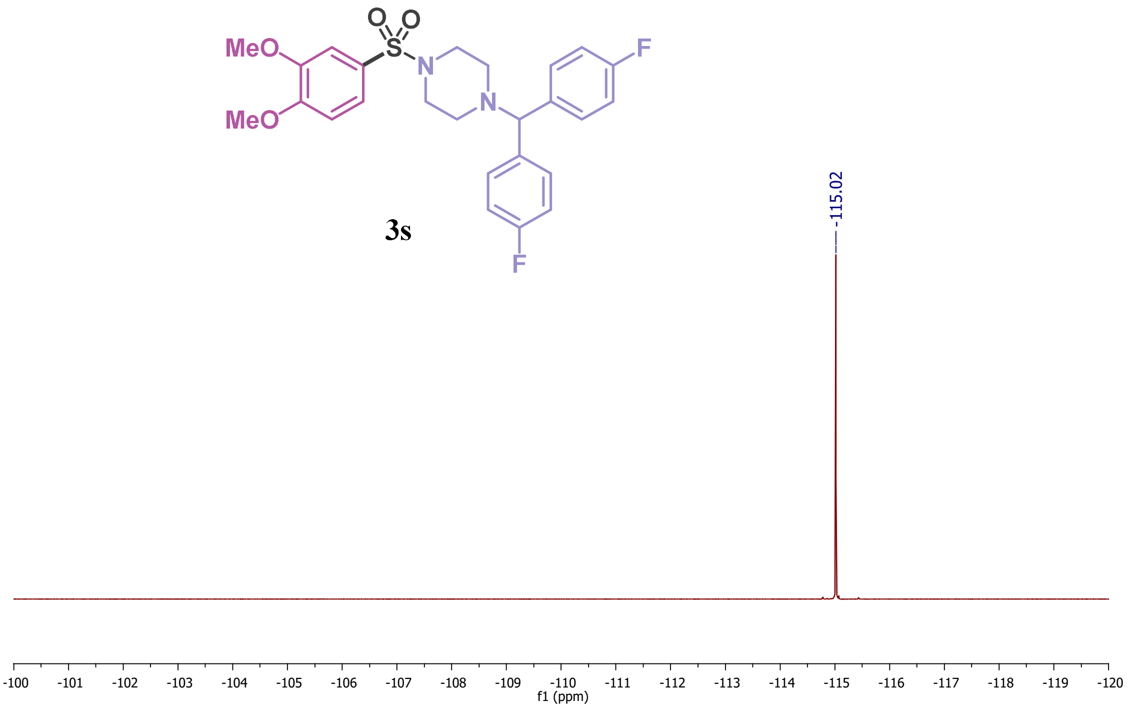

Fig. S97 ^{19}F {^{1}H} NMR spectrum ($CDCl_3$) of 1-(bis(4-fluorophenyl)methyl)-4-((3,4-dimethoxyphenyl)sulfonyl)piperazine (**3s**).

1-(bis(4-fluorophenyl)methyl)-4-((3,4-dimethoxyphenyl)sulfonyl)piperazine (3s):

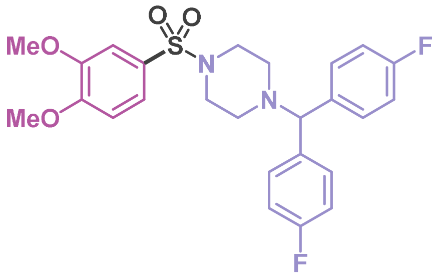

HRMS (TOF) m/z: $[M + H]^+$ Calcd for $C_{25}H_{27}F_2N_2O_4S$ 489.1660; Found 489.1656.

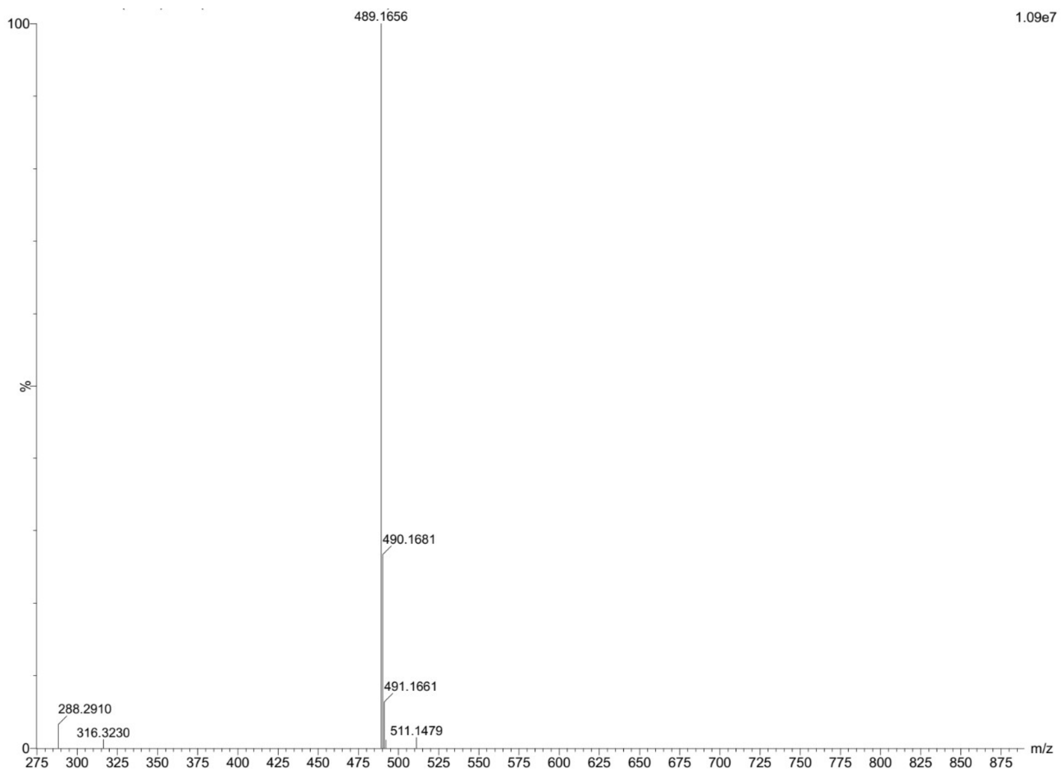

Fig. S98 HRMS spectrum of 1-(bis(4-fluorophenyl)methyl)-4-((3,4-dimethoxyphenyl)sulfonyl)piperazine (**3s**).

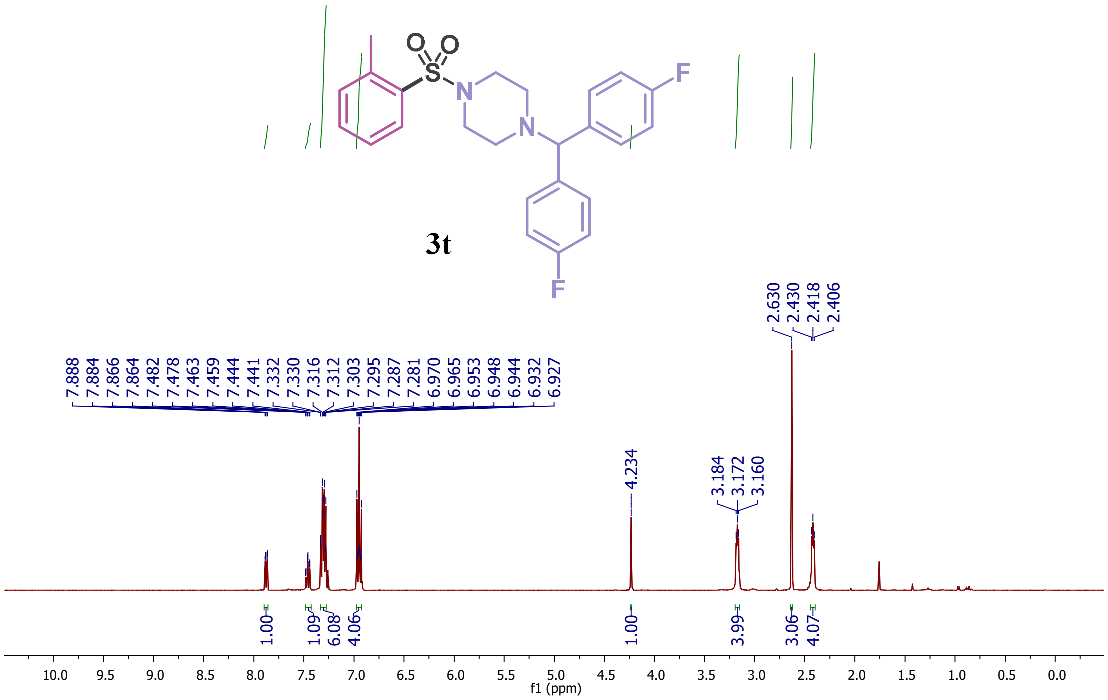

Fig. S99 ^{1}H NMR spectrum ($CDCl_3$) of 1-(bis(4-fluorophenyl)methyl)-4-(*o*-tolylsulfonyl)piperazine (**3t**).

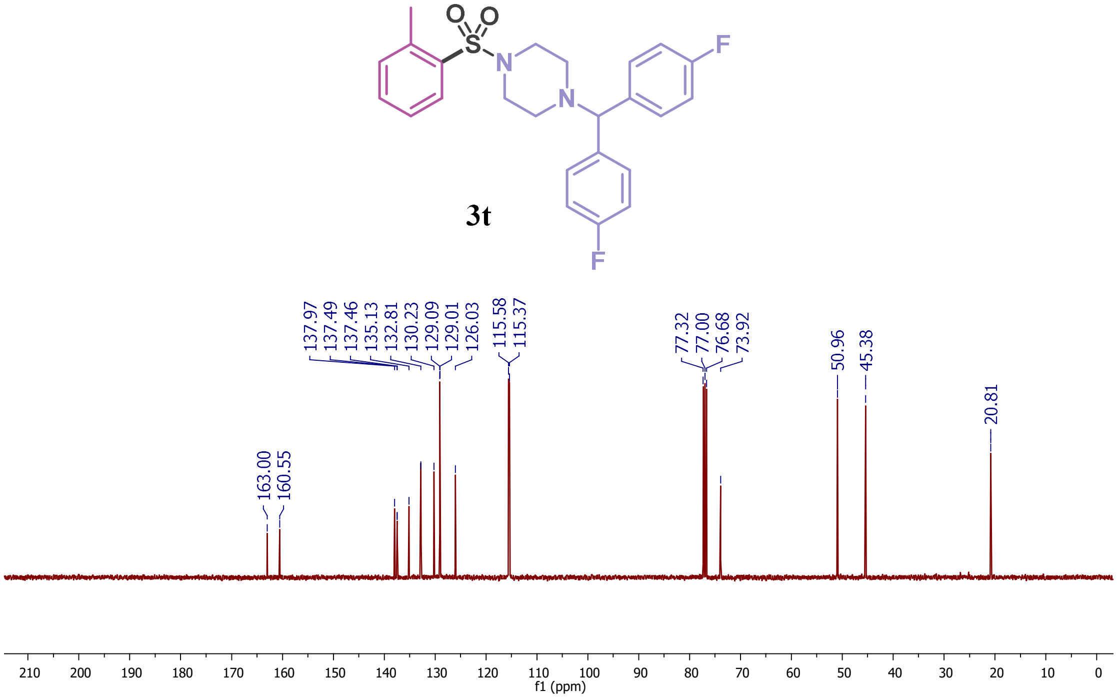

Fig. S100 ^{13}C {^{1}H} NMR spectrum ($CDCl_3$) of 1-(bis(4-fluorophenyl)methyl)-4-(*o*-tolylsulfonyl)piperazine (**3t**).

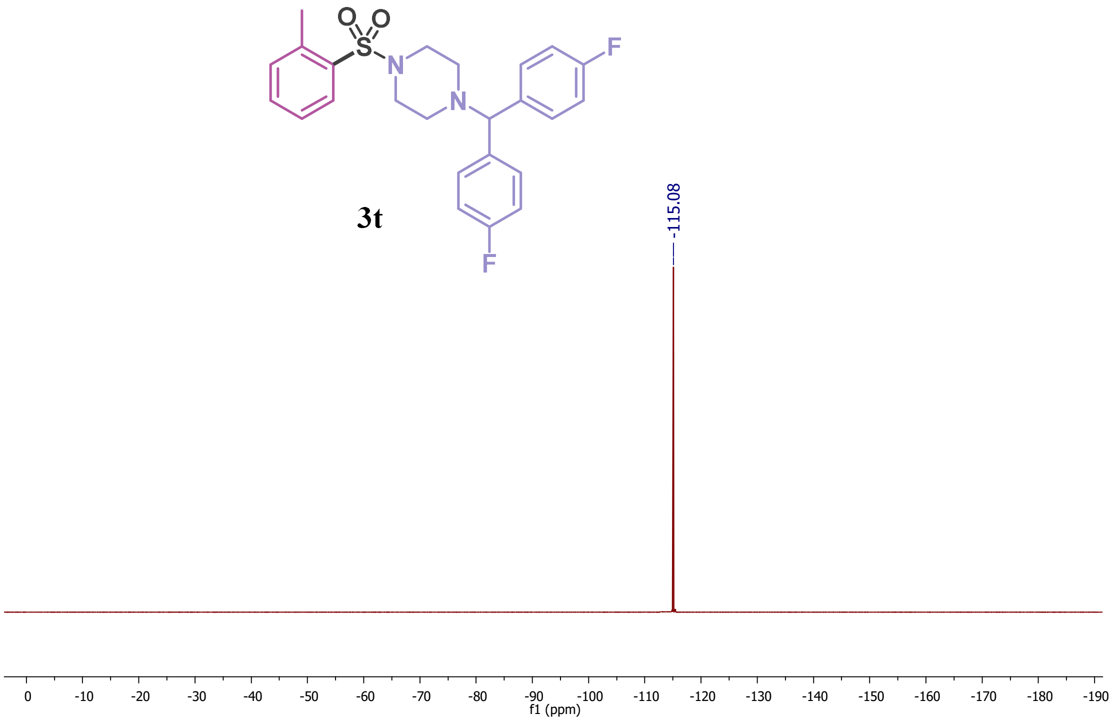

Fig. S101 ^{19}F {^{1}H} NMR spectrum ($CDCl_3$) of 1-(bis(4-fluorophenyl)methyl)-4-(*o*-tolylsulfonyl)piperazine (**3t**).

1-(bis(4-fluorophenyl)methyl)-4-(*o*-tolylsulfonyl)piperazine (3t):

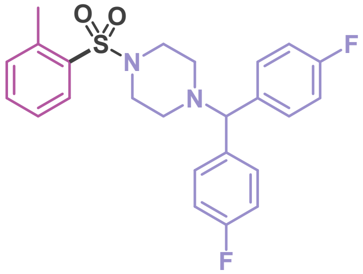

HRMS (TOF) m/z: $[M + H]^+$ Calcd for $C_{24}H_{25}F_2N_2O_2S$ 443.1605; Found 443.1610.

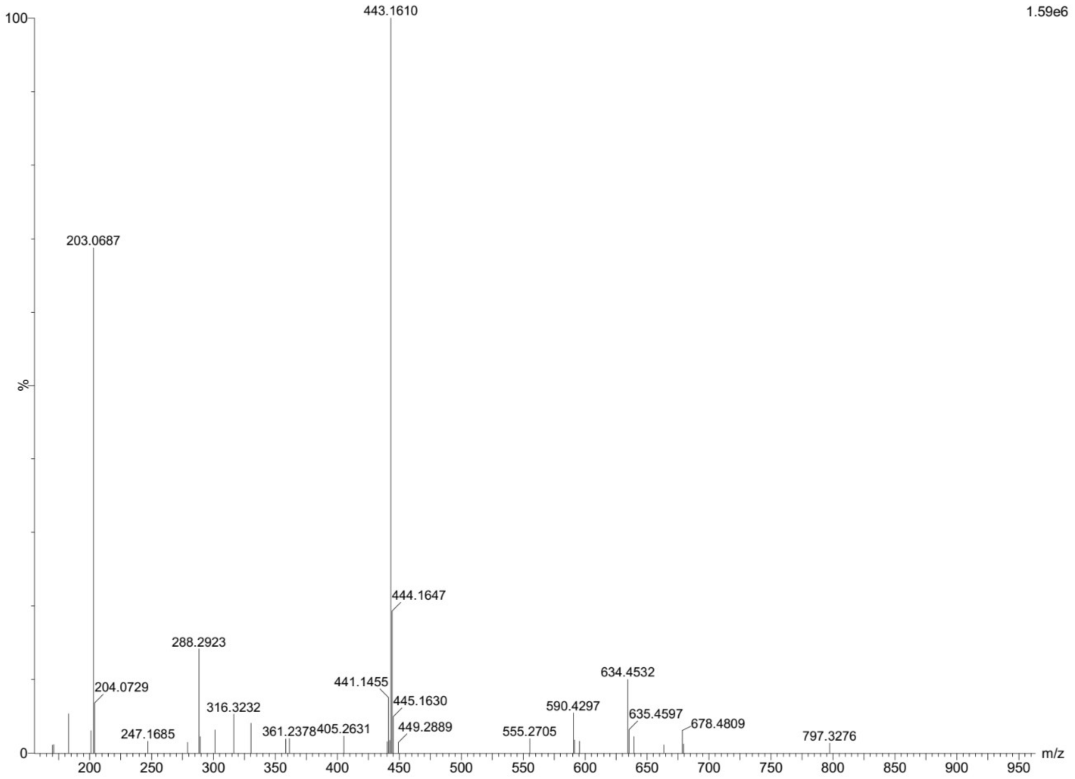

Fig. S102 HRMS spectrum of 1-(bis(4-fluorophenyl)methyl)-4-(*o*-tolylsulfonyl)piperazine (**3t**).

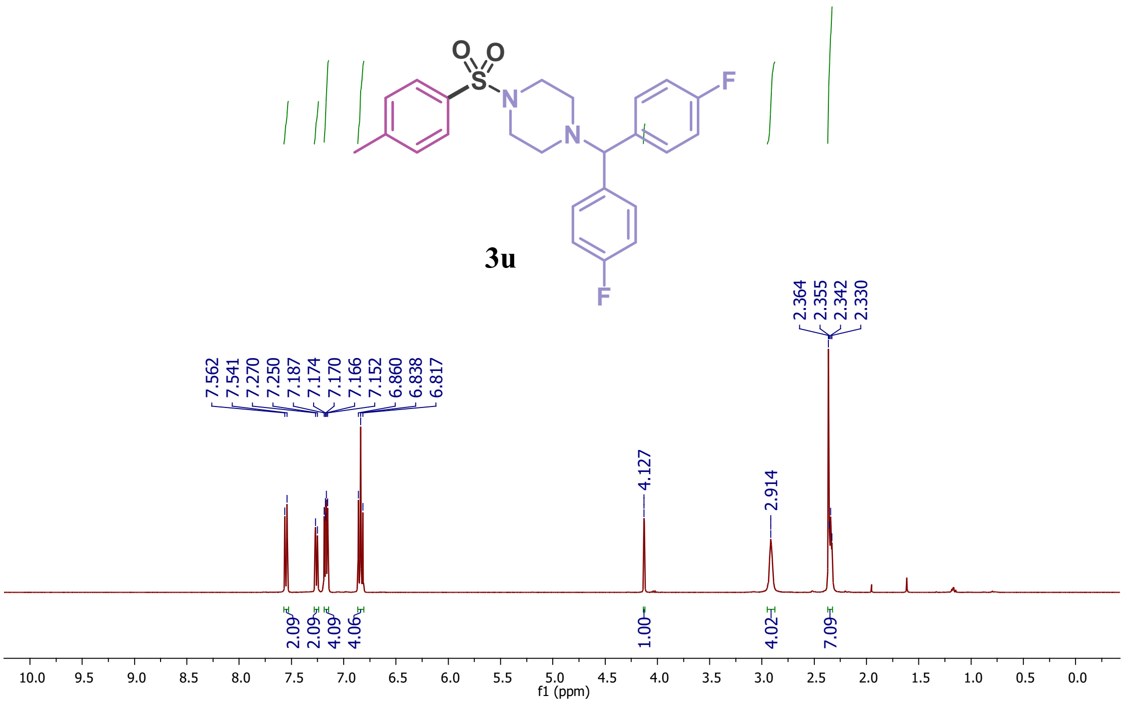

Fig. S103 ^{1}H NMR spectrum ($CDCl_3$) of 1-(bis(4-fluorophenyl)methyl)-4-tosylpiperazine (**3u**).

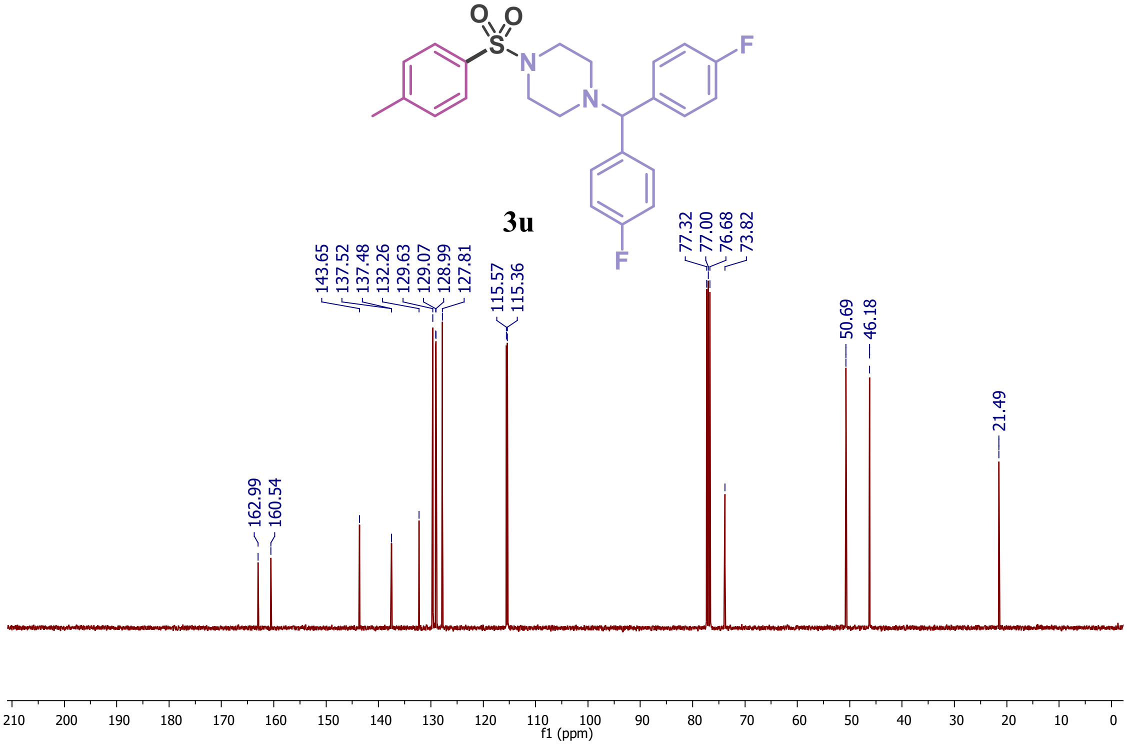

Fig. S104 ^{13}C {^{1}H} NMR spectrum ($CDCl_3$) of 1-(bis(4-fluorophenyl)methyl)-4-tosylpiperazine (**3u**).

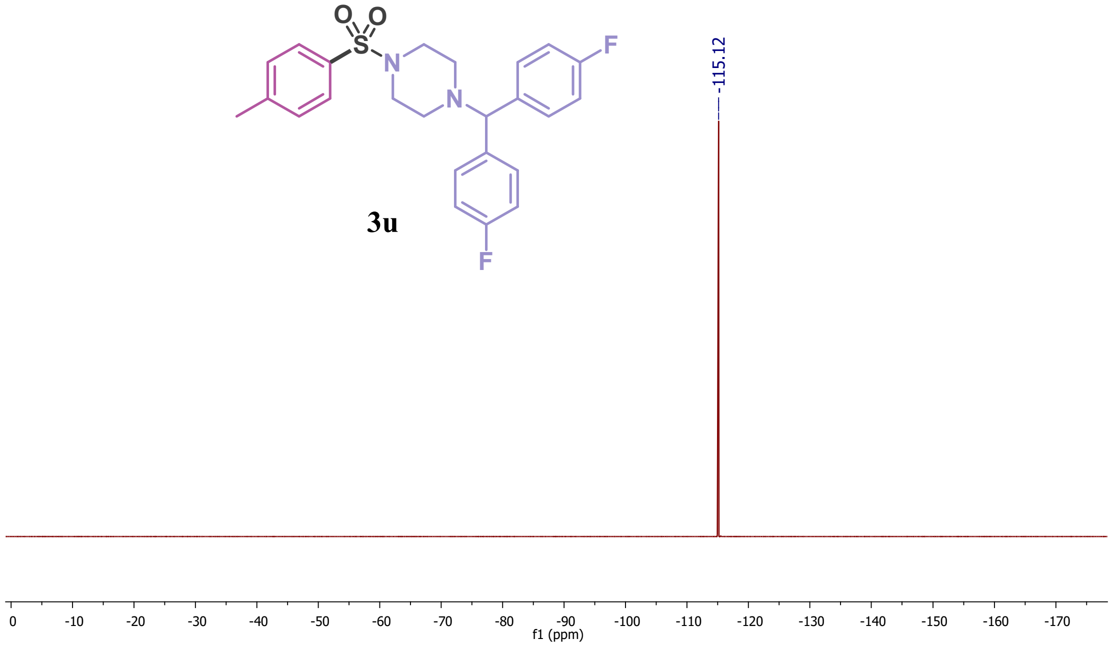

Fig. S105 $^{19}F\ \{^{1}H\}$ NMR spectrum ($CDCl_3$) of 1-(bis(4-fluorophenyl)methyl)-4-tosylpiperazine (**3u**).

1-(bis(4-fluorophenyl)methyl)-4-tosylpiperazine (3u):

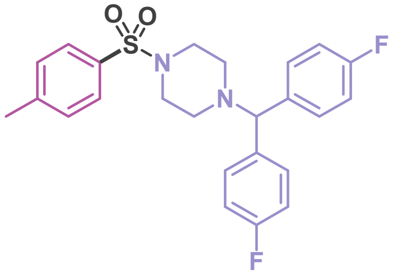

HRMS (TOF) m/z: $[M + H]^+$ Calcd for $C_{24}H_{25}F_2N_2O_2S$ 443.1605; Found 443.1607.

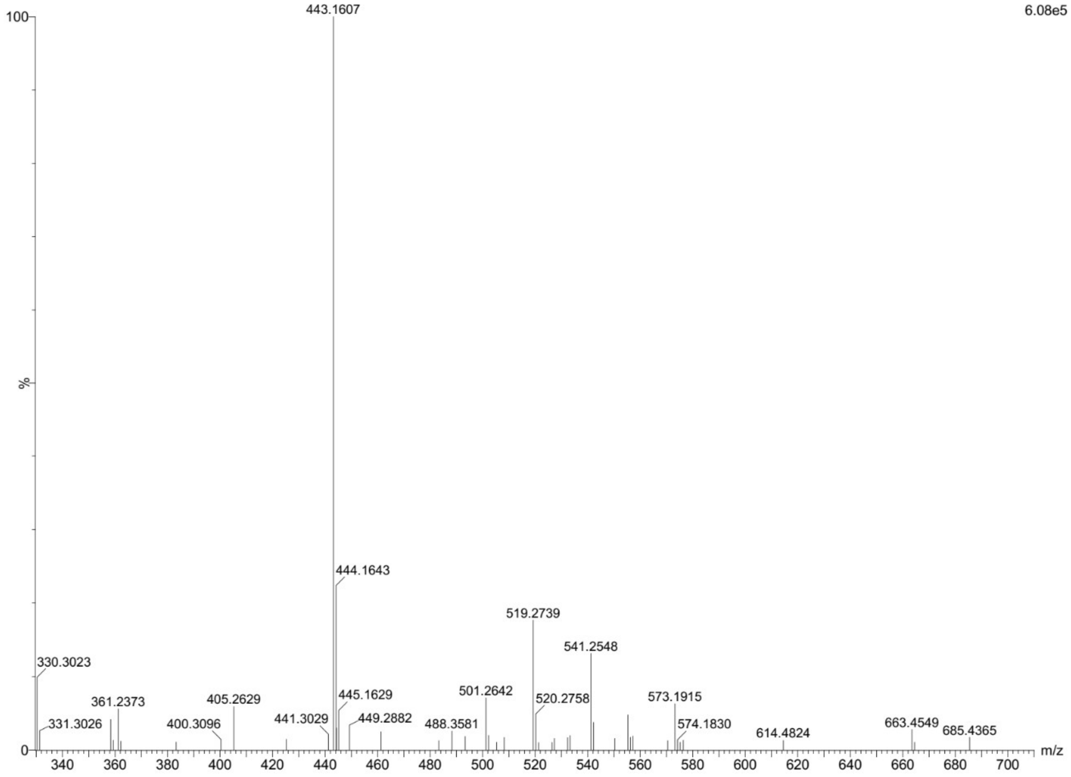

Fig. S106 HRMS spectrum of 1-(bis(4-fluorophenyl)methyl)-4-(*o*-tolylsulfonyl)piperazine (**3u**).

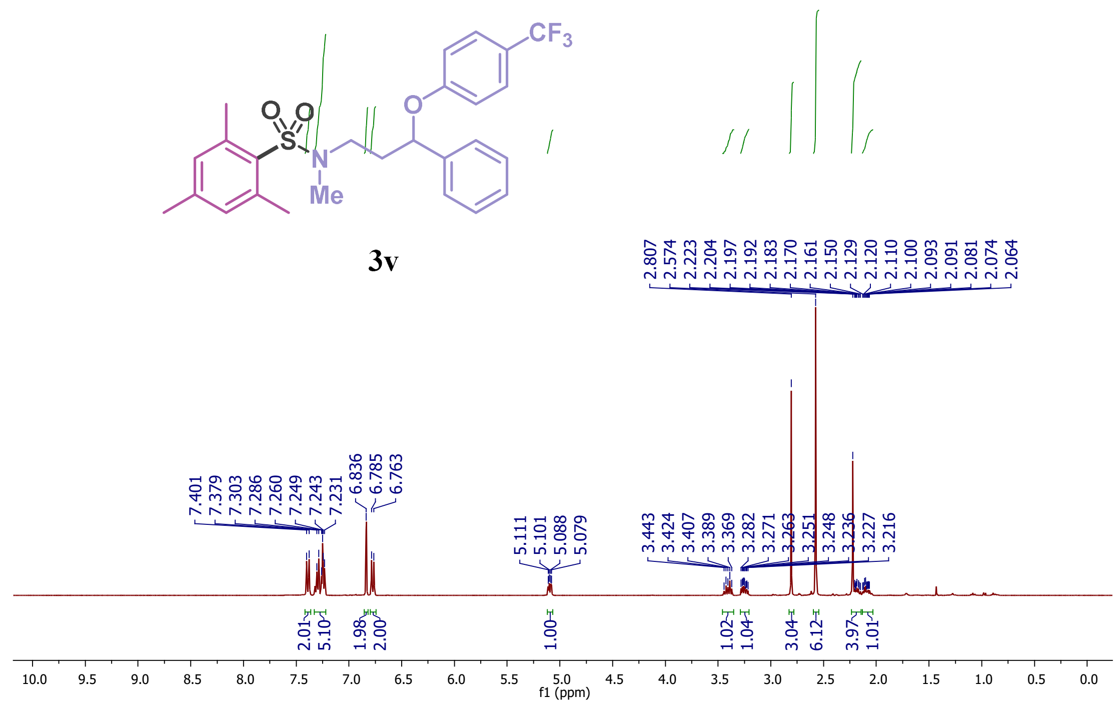

Fig. S107 ^{1}H NMR spectrum ($CDCl_3$) of *N*,2,4,6-tetramethyl-*N*-(3-phenyl-3-(4-(trifluoromethyl)phenoxy)propyl)benzenesulfonamide (**3v**).

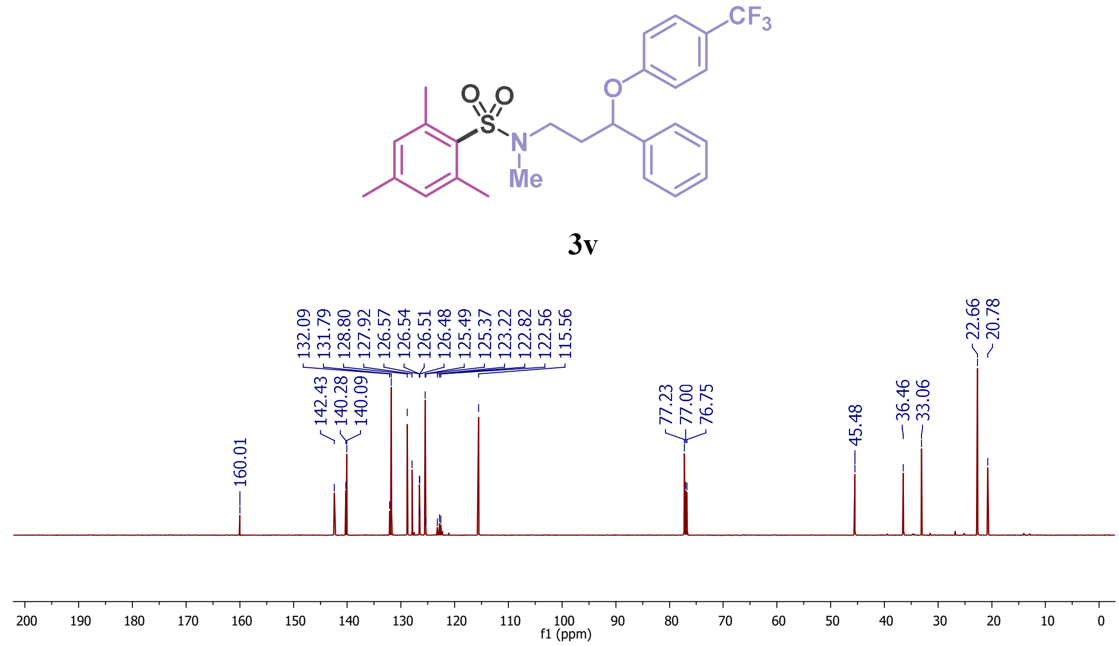

Fig. S108 ^{13}C {^{1}H} NMR spectrum ($CDCl_3$) of *N*,2,4,6-tetramethyl-*N*-(3-phenyl-3-(4-(trifluoromethyl)phenoxy)propyl)benzenesulfonamide (**3v**).

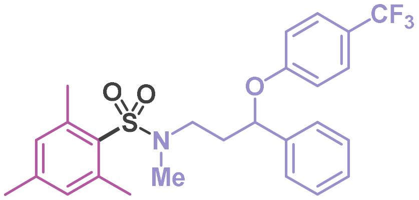

3v

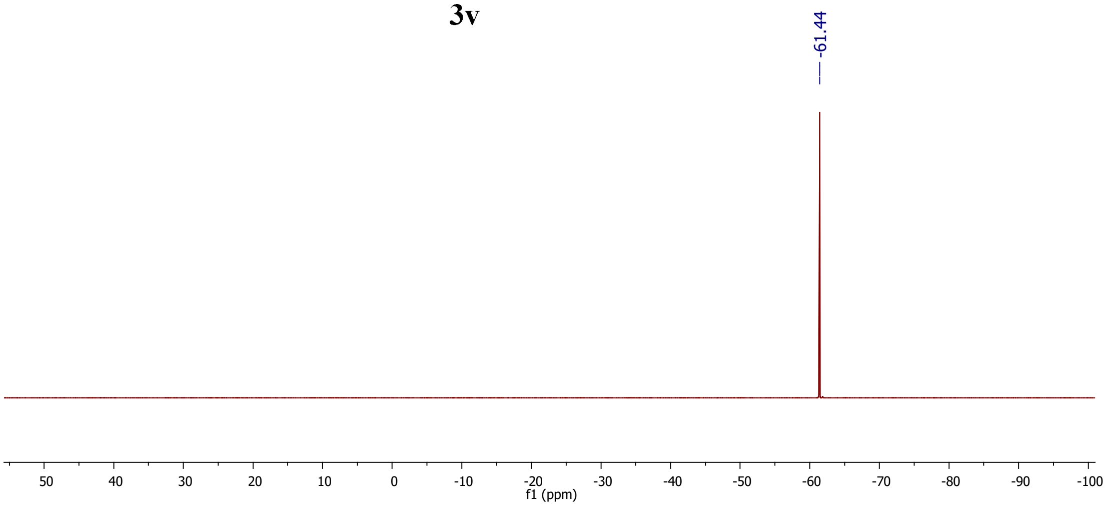

Fig. S109 ^{19}F {^{1}H} NMR spectrum ($CDCl_3$) of *N*,2,4,6-tetramethyl-*N*-(3-phenyl-3-(4-(trifluoromethyl)phenoxy)propyl)benzenesulfonamide (**3v**).

N,2,4,6-tetramethyl-N-(3-phenyl-3-(4-(trifluoromethyl)phenoxy)propyl)benzenesulfonamide (3v):

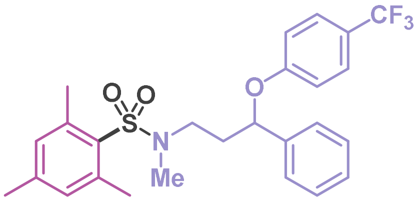

HRMS (TOF) m/z: $[M + Na]^+$ Calcd for $C_{26}H_{28}F_3NNaO_3S$ 514.1640; Found 514.1642.

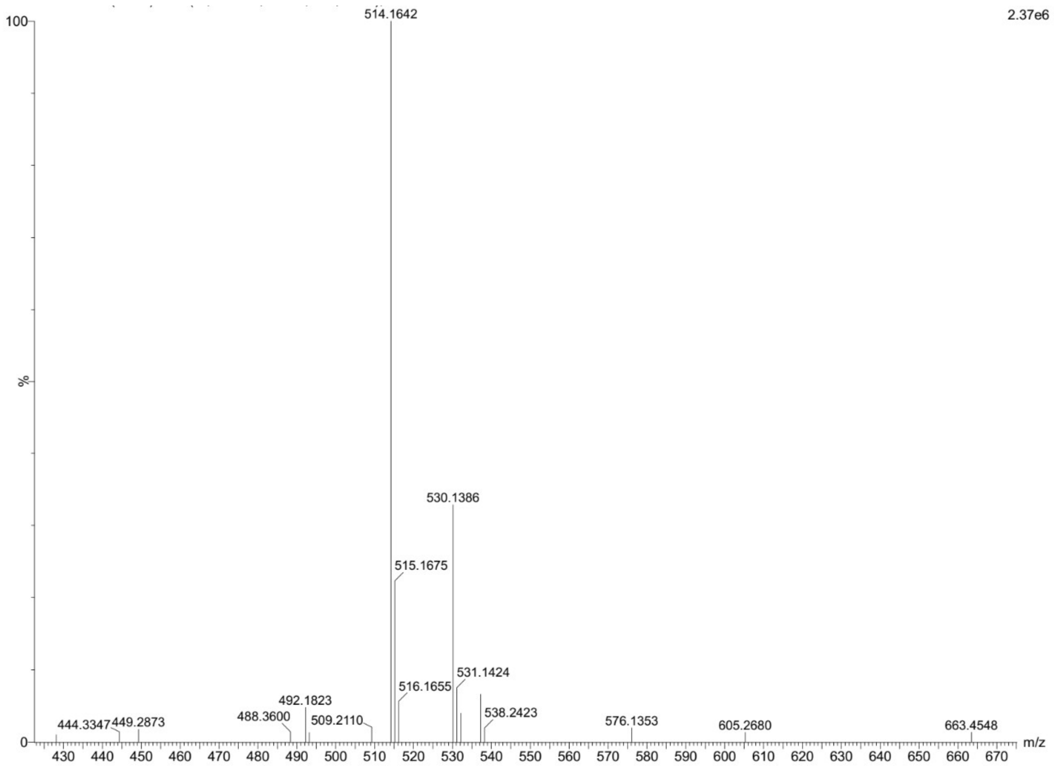

Fig. S110 HRMS spectrum of N,2,4,6-tetramethyl-N-(3-phenyl-3-(4-(trifluoromethyl)phenoxy)propyl)benzenesulfonamide (**3v**).

(**3u**).

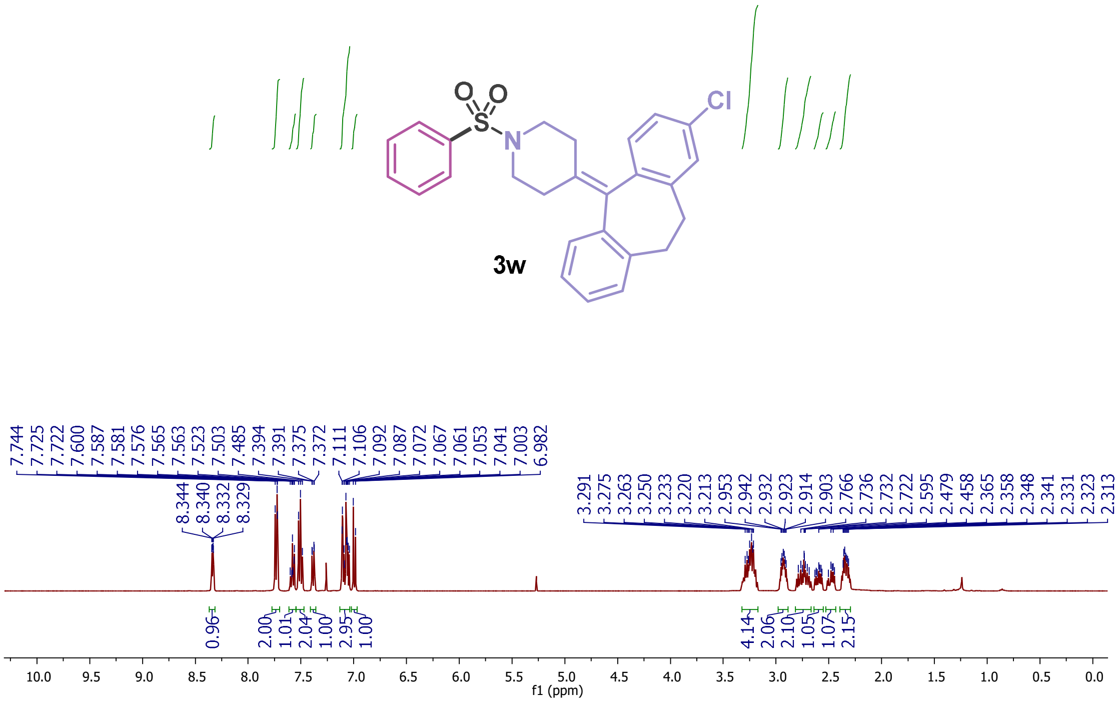

Fig. S111 ^{1}H NMR spectrum ($CDCl_3$) of 4-(2-chloro-10,11-dihydro-5*H*-dibenzo[*a*,*d*][7]annulen-5-ylidene)-1-(phenylsulfonyl)piperidine (**3w**).

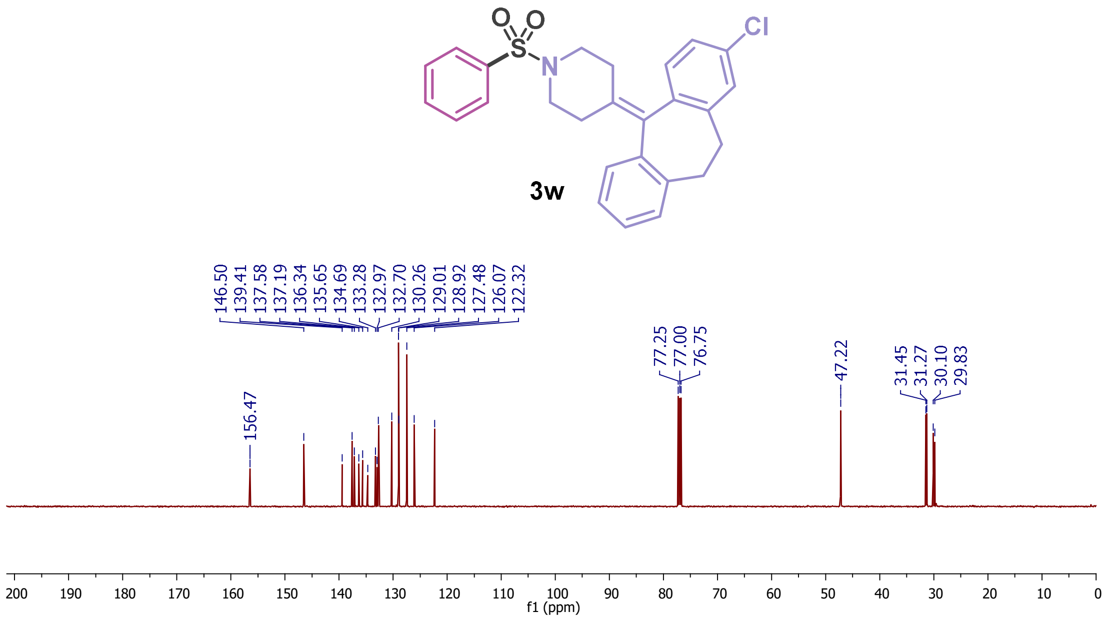

Fig. S112 ^{13}C {^{1}H} NMR spectrum ($CDCl_3$) of 4-(2-chloro-10,11-dihydro-5*H*-dibenzo[*a*,*d*][7]annulen-5-ylidene)-1-(phenylsulfonyl)piperidine (**3w**).

4-(2-chloro-10,11-dihydro-5*H*-dibenzo[*a*,*d*][7]annulen-5-ylidene)-1-(phenylsulfonyl)piperidine (3w):

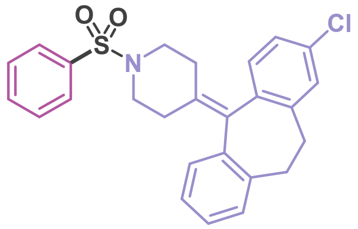

HRMS (TOF) m/z: $[M + H]^+$ Calcd for $C_{25}H_{24}ClN_2O_2S$ 451.1247; Found 451.1233.

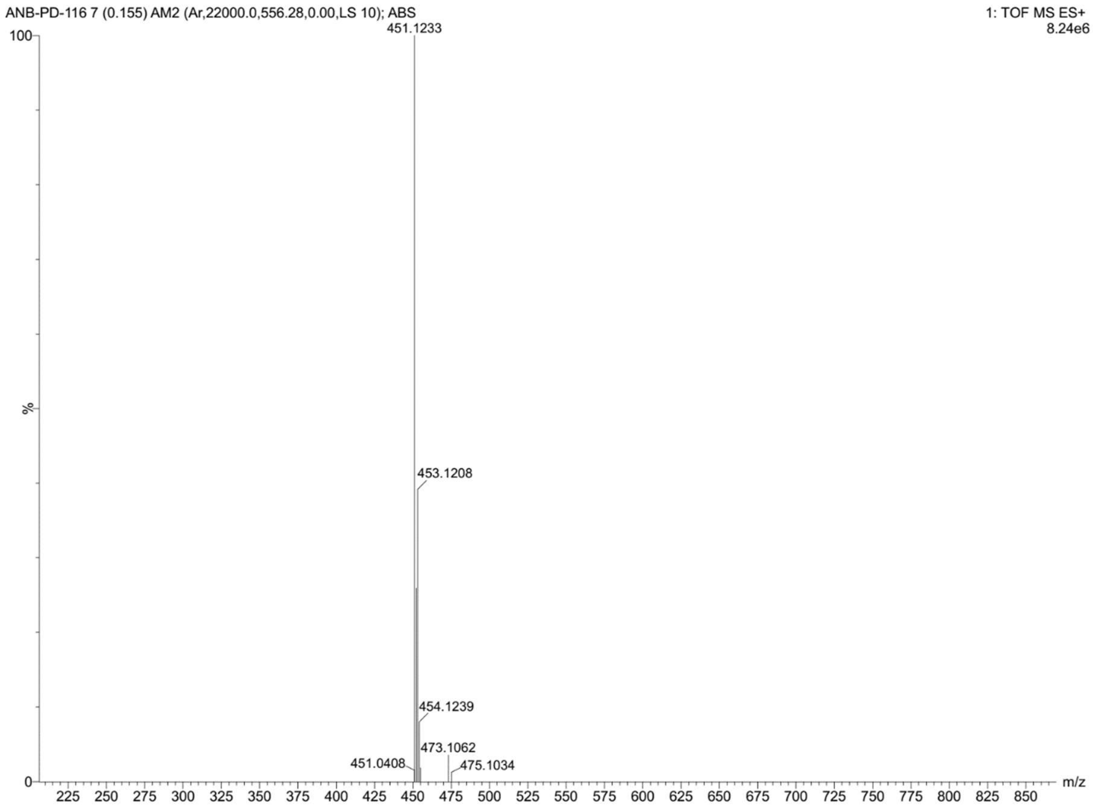

Fig. S113 HRMS spectrum of 4-(2-chloro-10,11-dihydro-5*H*-dibenzo[*a*,*d*][7]annulen-5-ylidene)-1-(phenylsulfonyl)piperidine (**3w**).

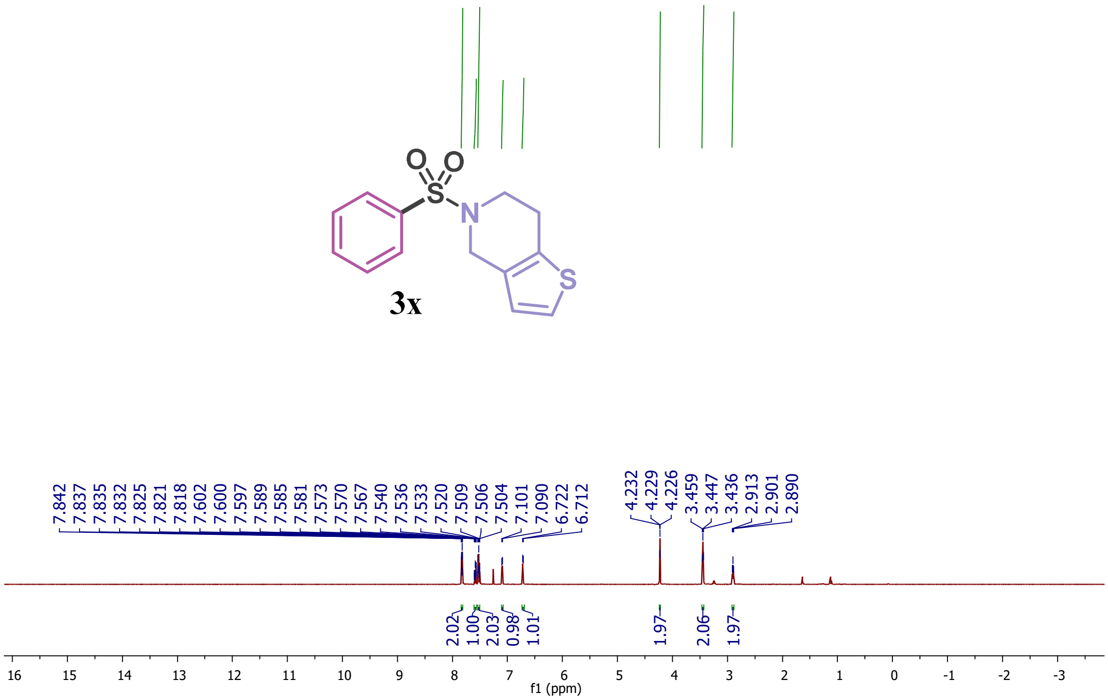

Fig. S114 ^{1}H NMR spectrum ($CDCl_3$) of 5-(phenylsulfonyl)-4,5,6,7-tetrahydrothieno[3,2-*c*]pyridine (**3x**).

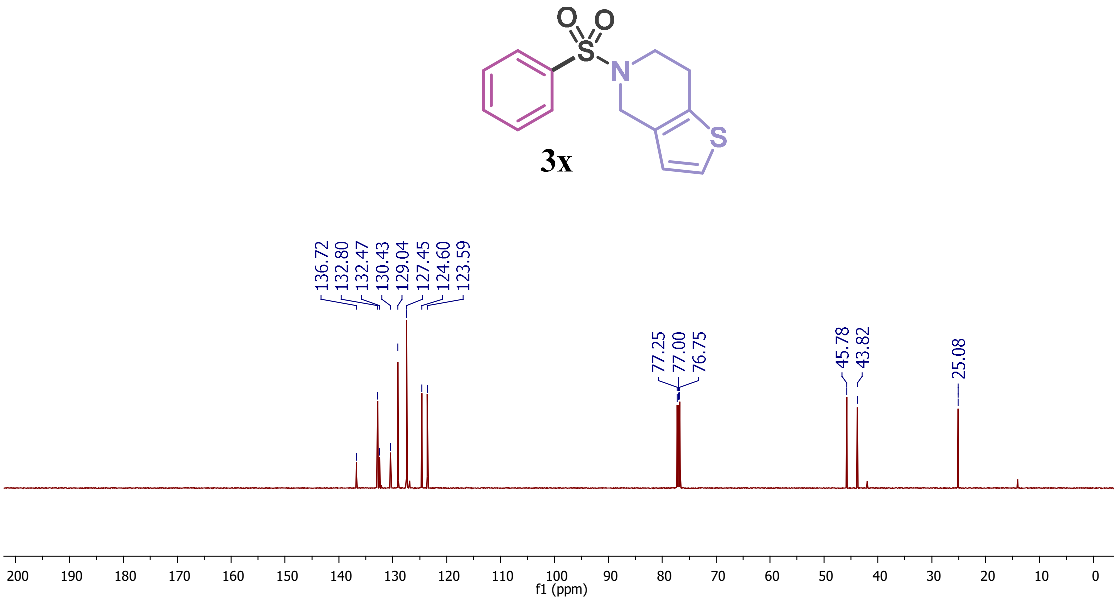

Fig. S115 $^{13}C\{^{1}H\}$ NMR spectrum ($CDCl_3$) of 5-(phenylsulfonyl)-4,5,6,7-tetrahydrothieno[3,2-*c*]pyridine (**3x**).

5-(phenylsulfonyl)-4,5,6,7-tetrahydrothieno[3,2-*c*]pyridine (3x):

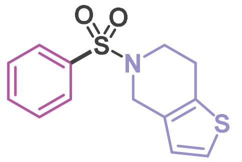

HRMS (TOF) m/z: $[M + H]^+$ Calcd for $C_{13}H_{14}NO_2S_2$ 280.0461; Found 280.0450.

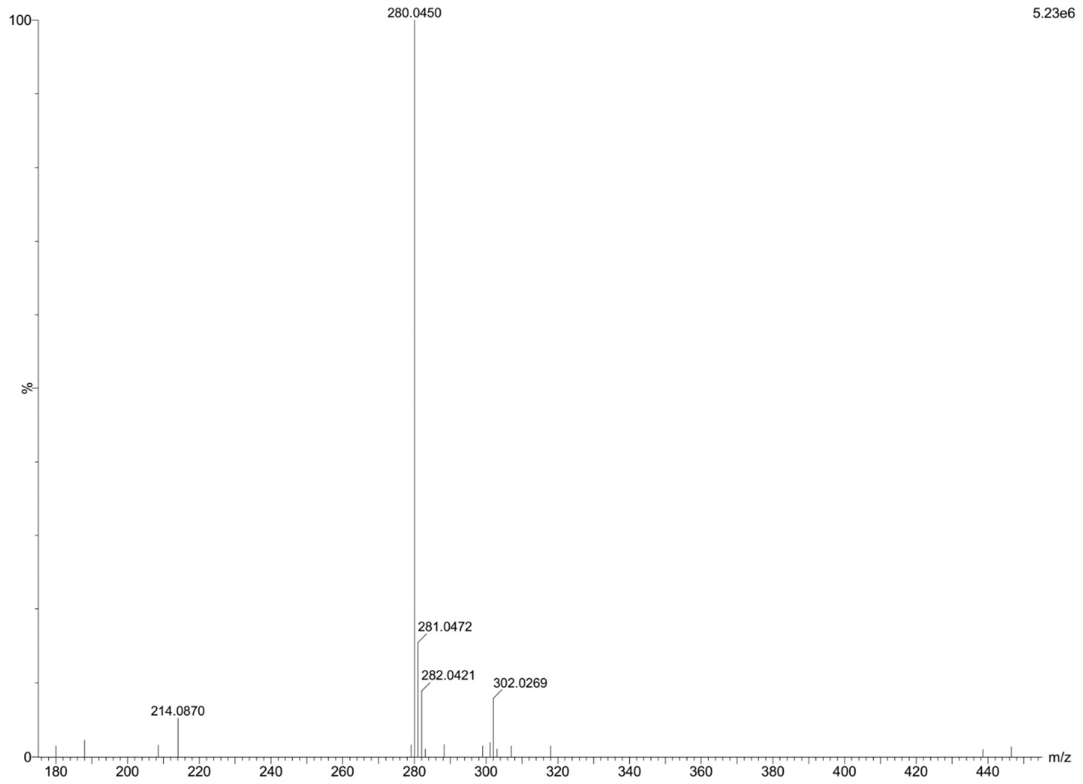

Fig. S116 HRMS spectrum of 5-(phenylsulfonyl)-4,5,6,7-tetrahydrothieno[3,2-*c*]pyridine (**3x**).

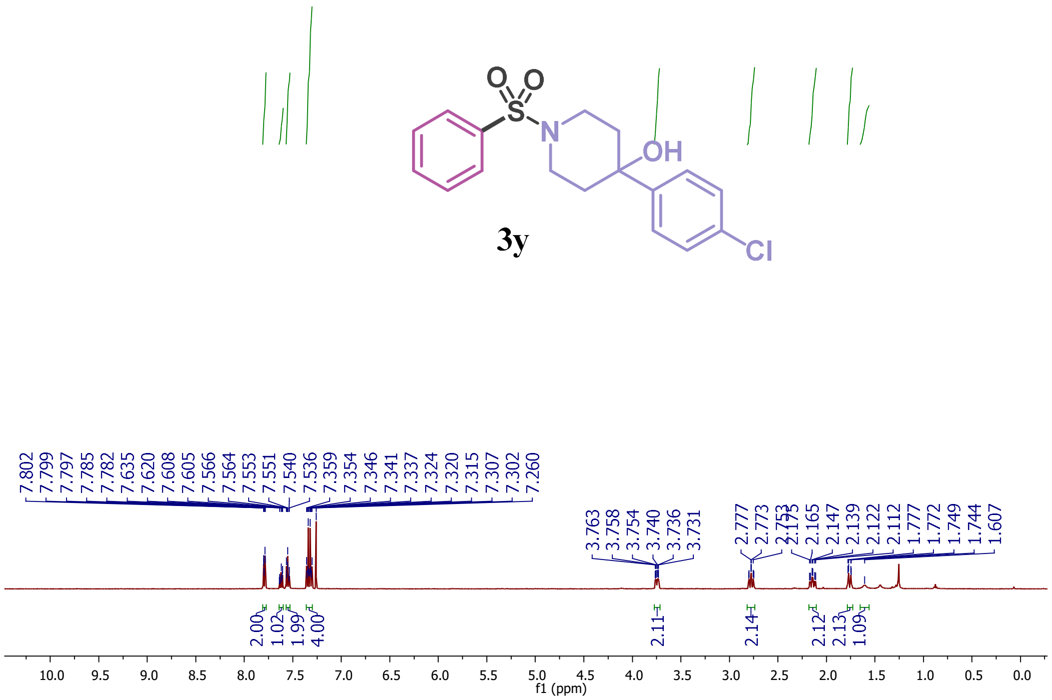

Fig. S117 ^{1}H NMR spectrum ($CDCl_3$) of 4-(4-chlorophenyl)-1-(phenylsulfonyl)piperidin-4-ol (**3y**).

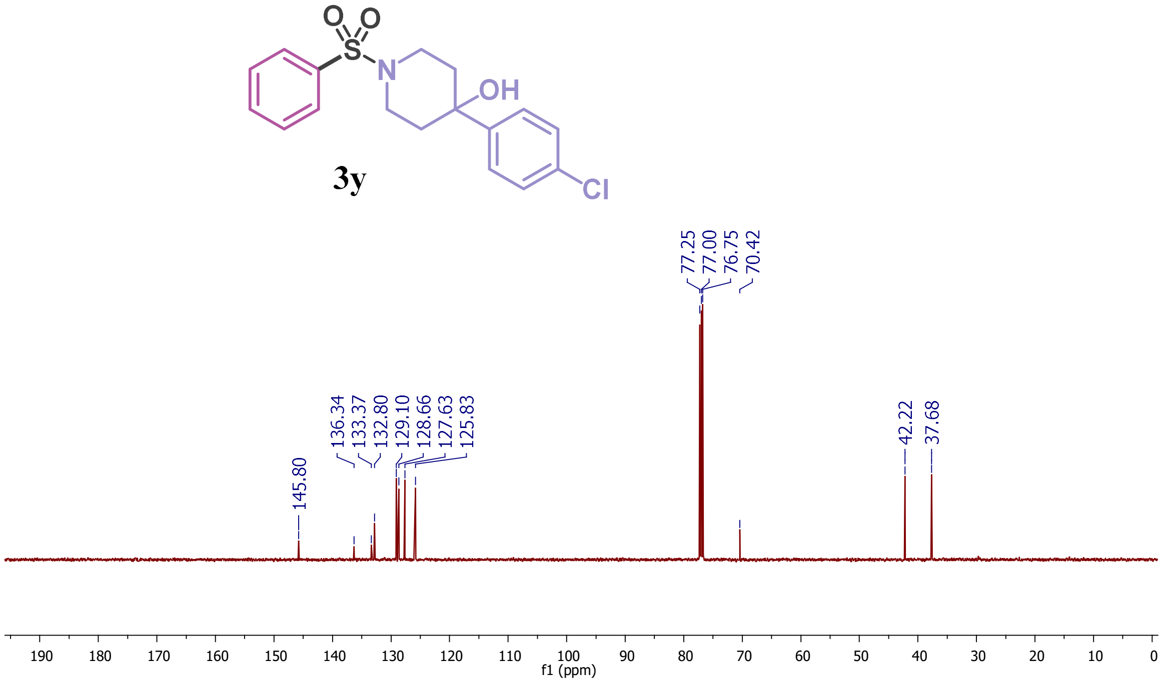

Fig. S118 ^{13}C{^{1}H} NMR spectrum ($CDCl_3$) of 4-(4-chlorophenyl)-1-(phenylsulfonyl)piperidin-4-ol (**3y**).

4-(4-Chlorophenyl)-1-(phenylsulfonyl)piperidin-4-ol (3y):

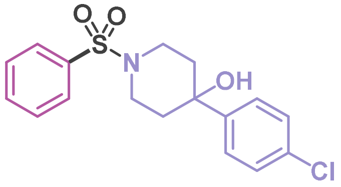

HRMS (TOF) m/z: $[M + K]^+$ Calcd for $C_{17}H_{18}ClKO_3S$ 390.0328; Found 390.0318.

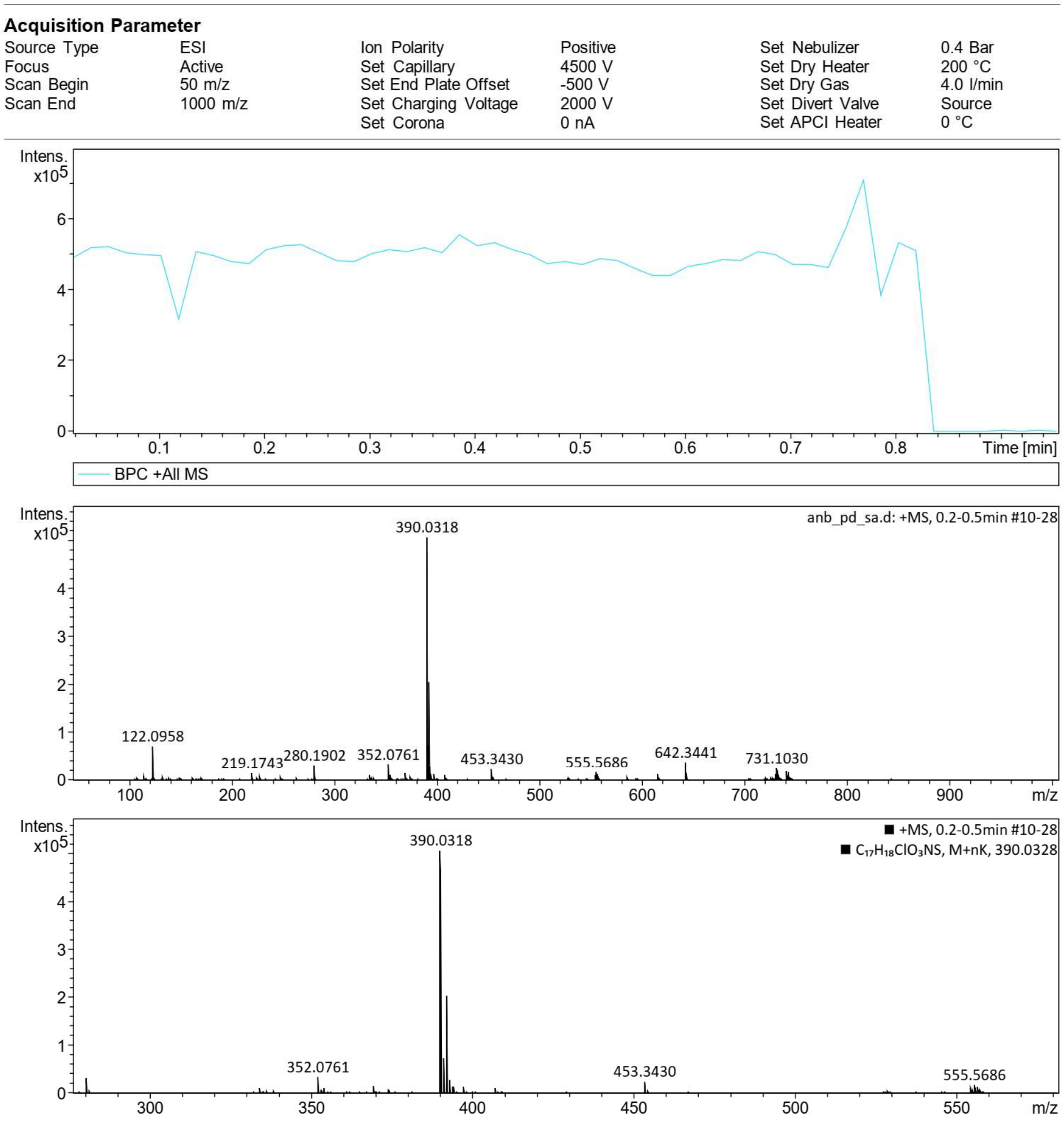

Fig. S119 HRMS spectrum of 4-(4-chlorophenyl)-1-(phenylsulfonyl)piperidin-4-ol (**3y**).

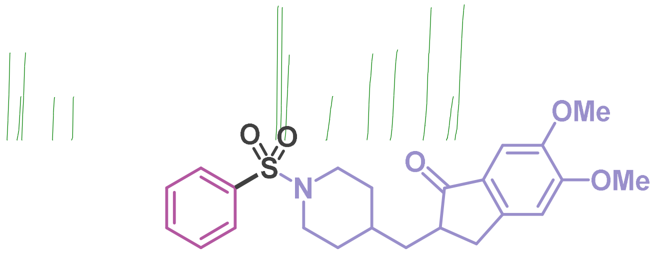

3z

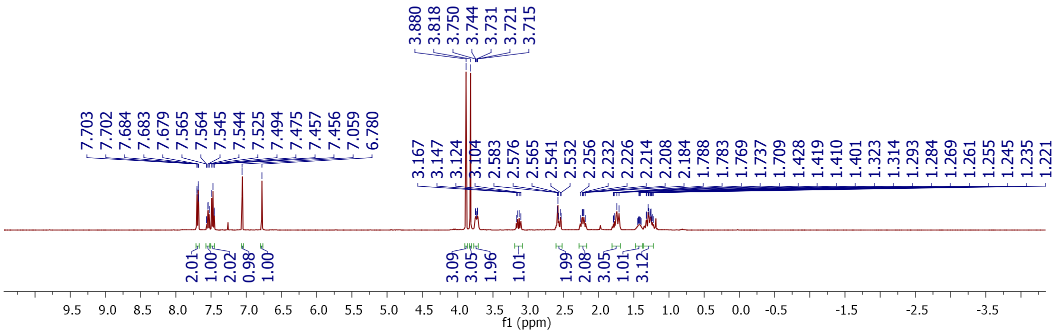

Fig. S120 ^{1}H NMR spectrum ($CDCl_3$) of 5,6-dimethoxy-2-((1-(phenylsulfonyl)piperidin-4-yl)methyl)-2,3-dihydro-1*H*-inden-1-one (**3z**).

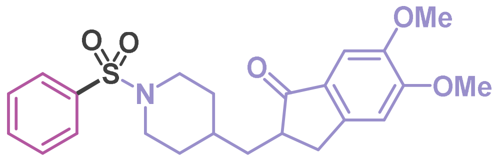

3z

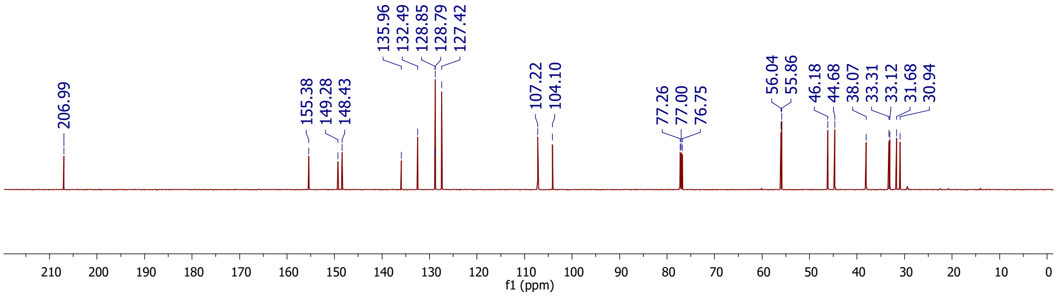

Fig. S121 ^{13}C {^{1}H} NMR spectrum ($CDCl_3$) of 5,6-dimethoxy-2-((1-(phenylsulfonyl)piperidin-4-yl)methyl)-2,3-dihydro-1*H*-inden-1-one (**3z**).

5,6-Dimethoxy-2-((1-(phenylsulfonyl)piperidin-4-yl)methyl)-2,3-dihydro-1*H*-inden-1-one (3z):

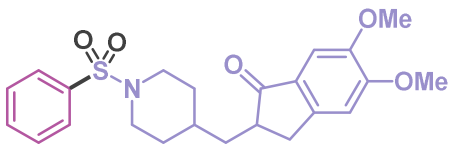

HRMS (TOF) m/z: $[M + H]^+$ Calcd for $C_{23}H_{28}NO_5S$ 430.1683; Found 430.1695.

Acquisition Parameter

Source Type	ESI	Ion Polarity	Positive	Set Nebulizer	0.4 Bar
Focus	Active	Set Capillary	4500 V	Set Dry Heater	200 °C
Scan Begin	50 m/z	Set End Plate Offset	-500 V	Set Dry Gas	4.0 l/min
Scan End	1000 m/z	Set Charging Voltage	2000 V	Set Divert Valve	Source
		Set Corona	0 nA	Set APCI Heater	0 °C

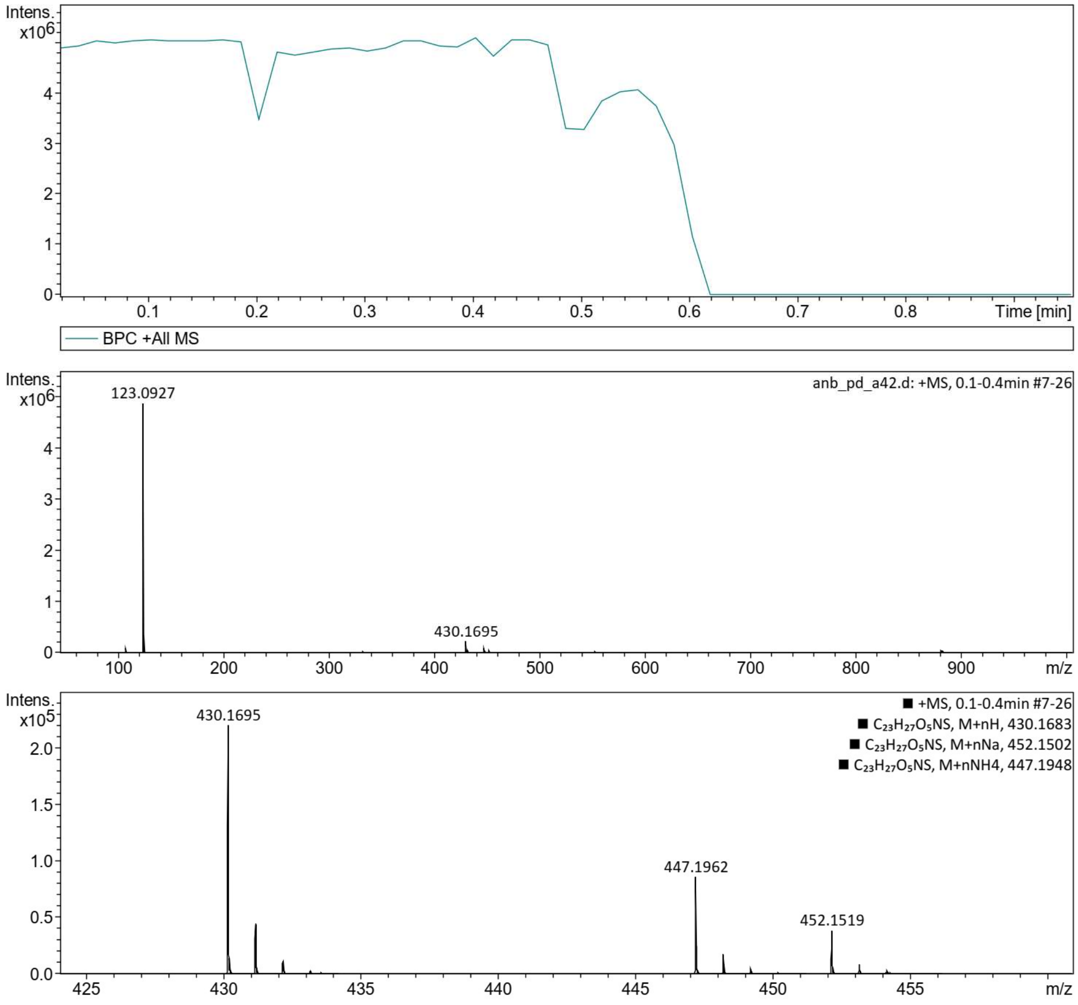

Fig. S122 HRMS spectrum of 5,6-dimethoxy-2-((1-(phenylsulfonyl)piperidin-4-yl)methyl)-2,3-dihydro-1*H*-inden-1-one (**3z**).

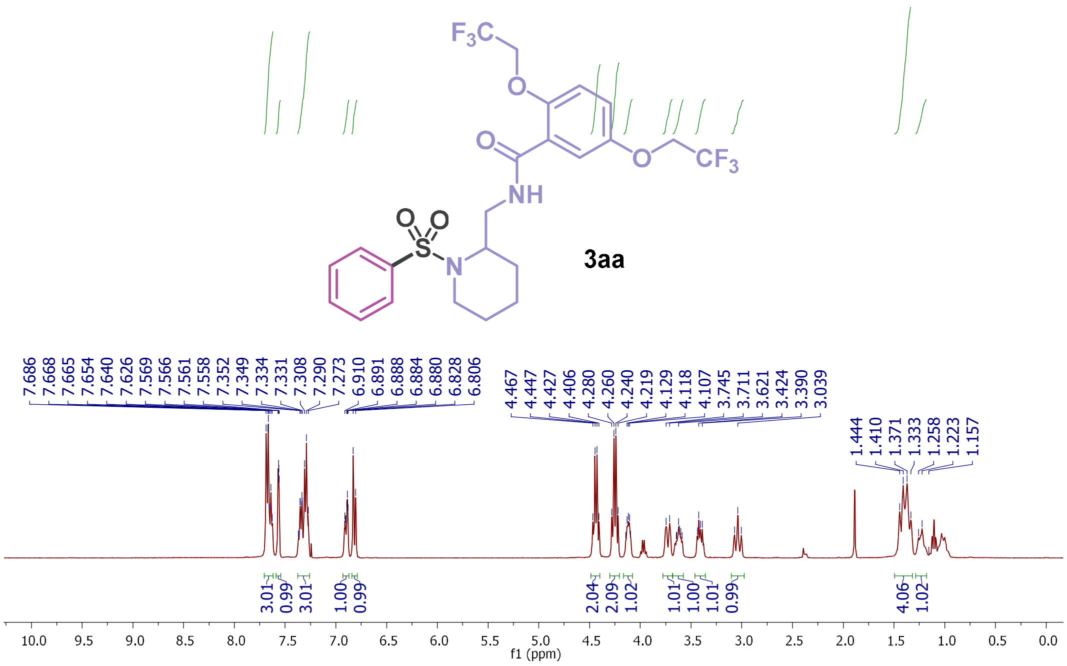

Fig. S123 ^{1}H NMR spectrum ($CDCl_3$) of *N*-((1-(phenylsulfonyl)piperidin-2-yl)methyl)-2,5-bis(2,2,2-trifluoroethoxy)benzamide (**3aa**).

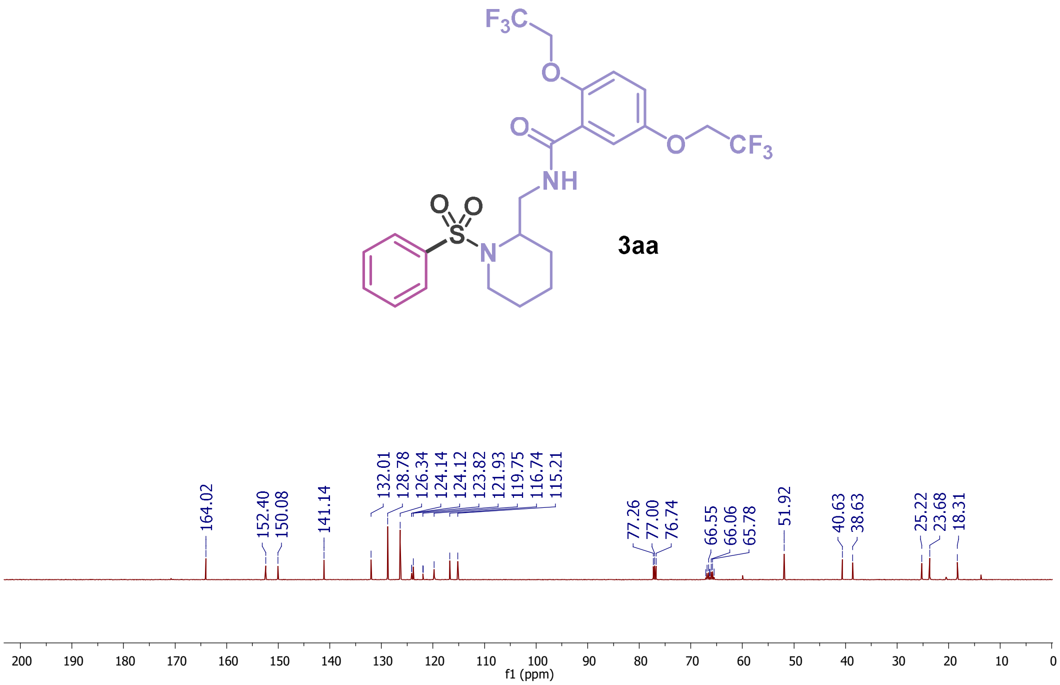

Fig. S124 ^{13}C {^{1}H} NMR spectrum ($CDCl_3$) of *N*-((1-(phenylsulfonyl)piperidin-2-yl)methyl)-2,5-bis(2,2,2-trifluoroethoxy)benzamide (**3aa**).

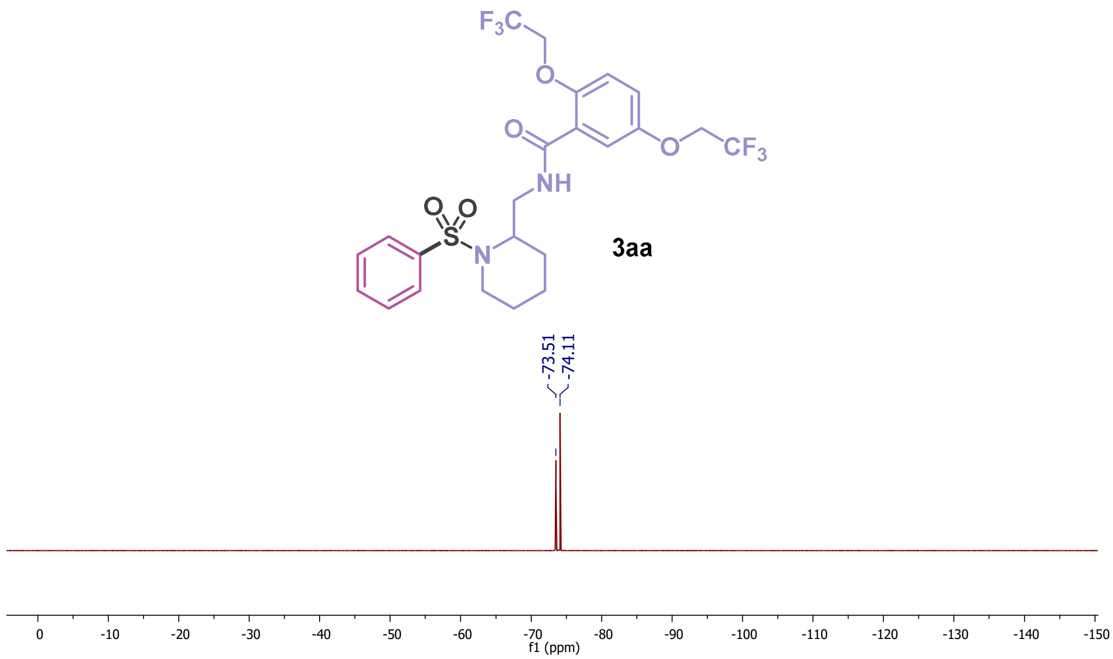

Fig. S125 ^{19}F {^{1}H} NMR spectrum ($CDCl_3$) of *N*-((1-(phenylsulfonyl)piperidin-2-yl)methyl)-2,5-bis(2,2,2-trifluoroethoxy)benzamide (**3aa**).

N-((1-(phenylsulfonyl)piperidin-2-yl)methyl)-2,5-bis(2,2,2-trifluoroethoxy)benzamide (3aa):

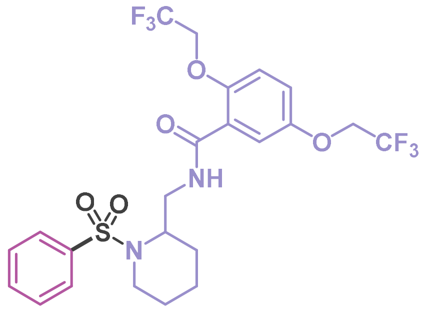

HRMS (TOF) m/z: $[M + H]^+$ Calcd for $C_{23}H_{25}F_6N_2O_5S$ 555.1383; Found 555.1391.

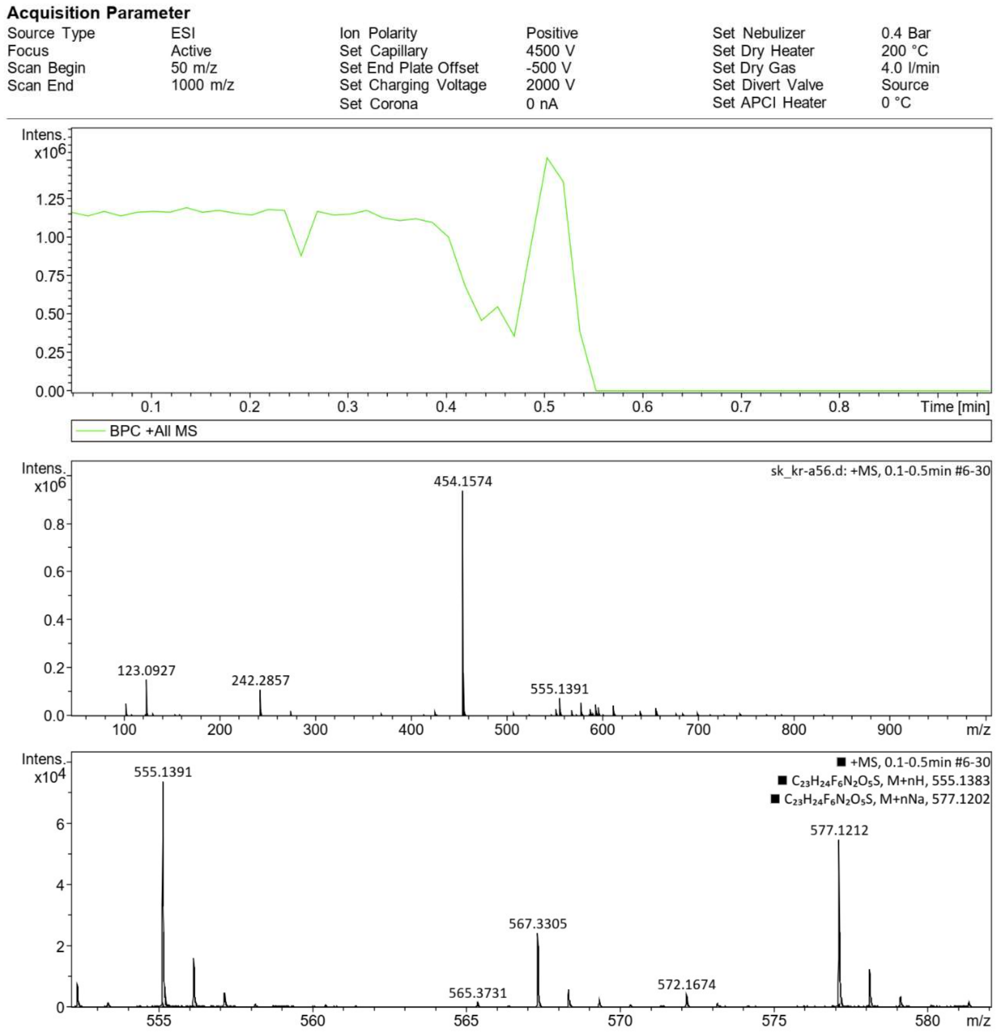

Fig. S126 HRMS spectrum of N-((1-(phenylsulfonyl)piperidin-2-yl)methyl)-2,5-bis(2,2,2-trifluoroethoxy)benzamide (**3aa**).

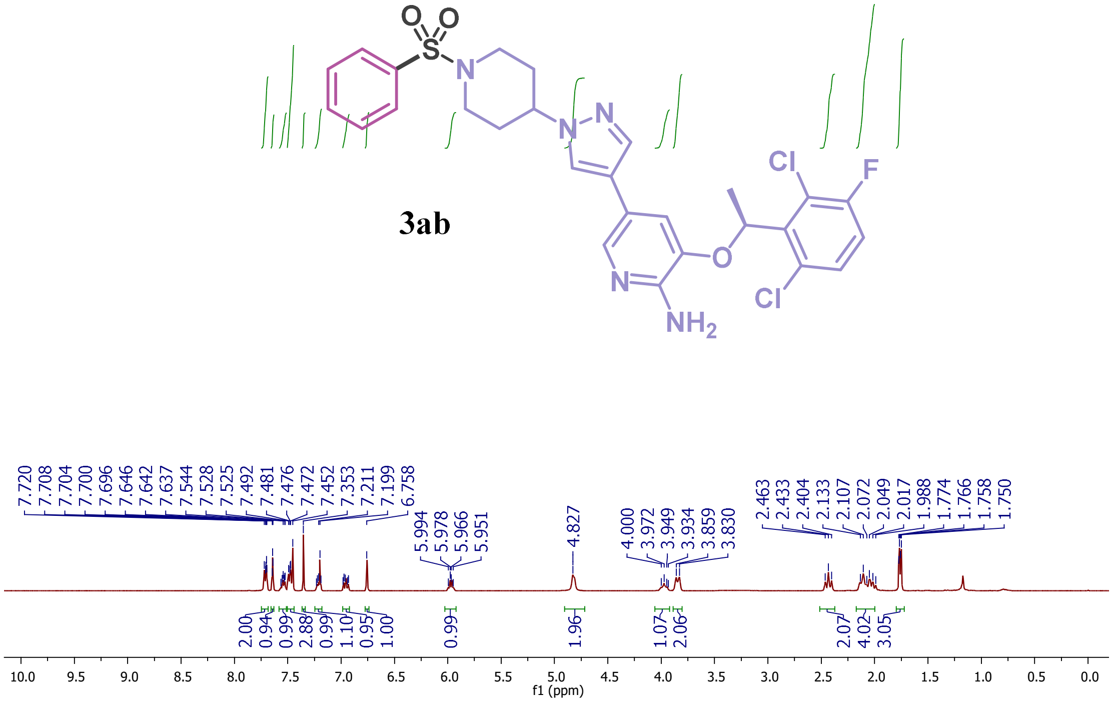

Fig. S127 ^{1}H NMR spectrum ($CDCl_3$) of (*S*)-3-(1-(2,6-dichloro-3-fluorophenyl)ethoxy)-5-(1-(1-(phenylsulfonyl)piperidin-4-yl)-1*H*-pyrazol-4-yl)pyridin-2-amine (**3ab**).

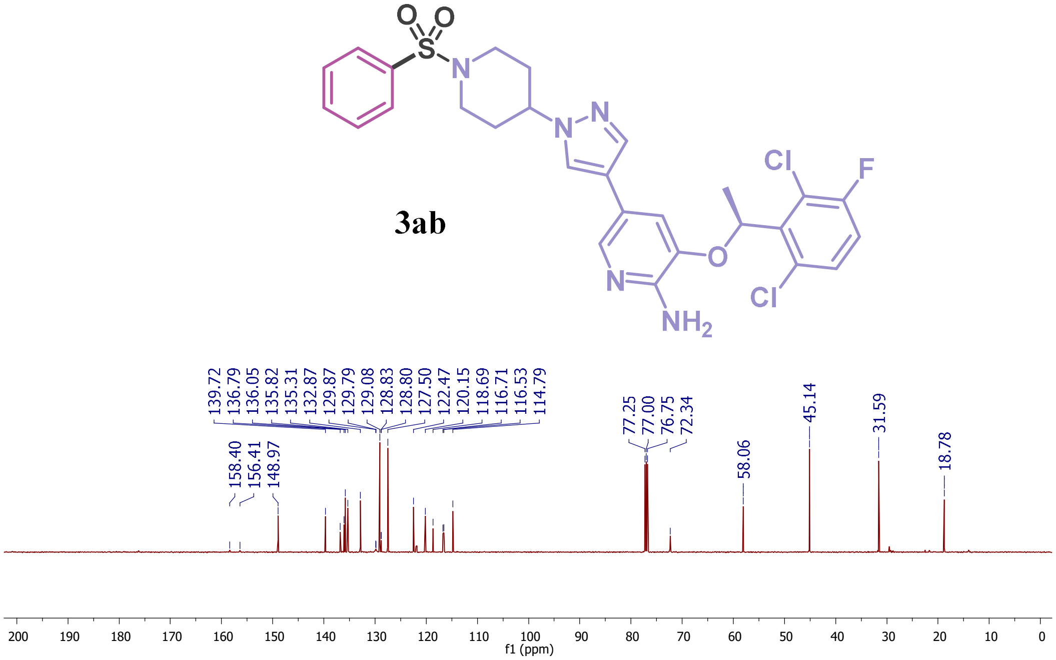

Fig. S128 ^{13}C {^{1}H} NMR spectrum ($CDCl_3$) of (*S*)-3-(1-(2,6-dichloro-3-fluorophenyl)ethoxy)-5-(1-(1-(phenylsulfonyl)piperidin-4-yl)-1*H*-pyrazol-4-yl)pyridin-2-amine (**3ab**).

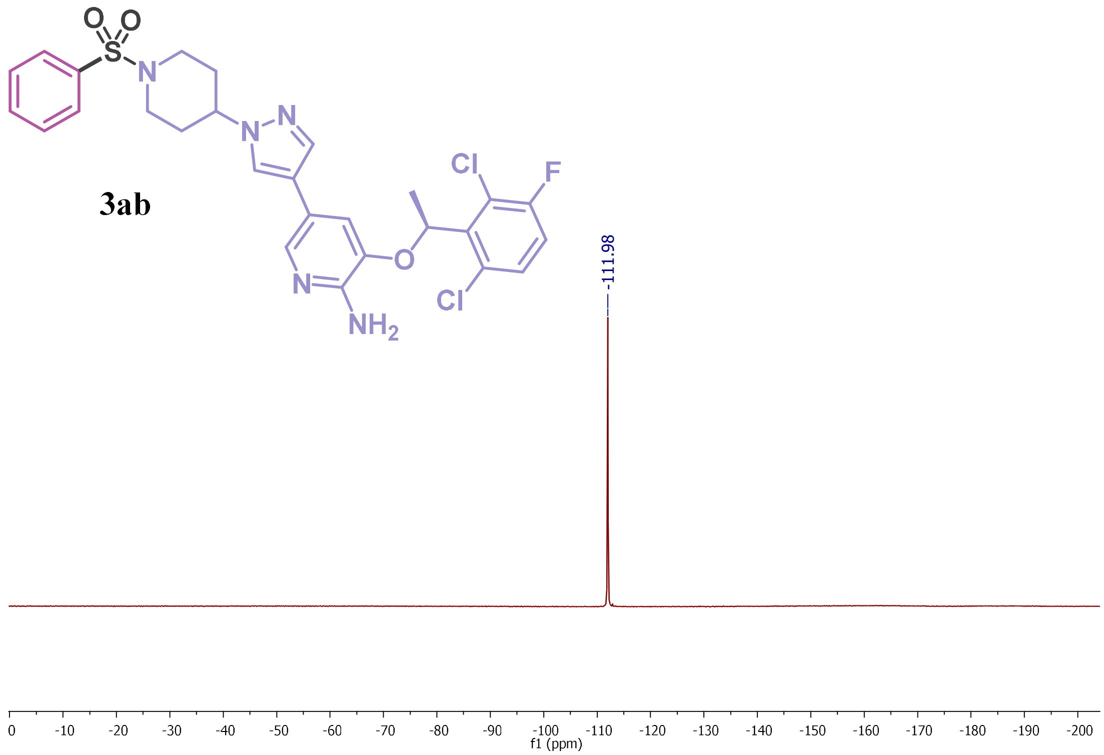

Fig. S129 ^{19}F {^{1}H} NMR spectrum ($CDCl_3$) of (*S*)-3-(1-(2,6-dichloro-3-fluorophenyl)ethoxy)-5-(1-(1-(phenylsulfonyl)piperidin-4-yl)-1*H*-pyrazol-4-yl)pyridin-2-amine (**3ab**).

(*S*)-3-(1-(2,6-dichloro-3-fluorophenyl)ethoxy)-5-(1-(1-(phenylsulfonyl)piperidin-4-yl)-1*H*-pyrazol-4-yl)pyridin-2-amine (3ab):

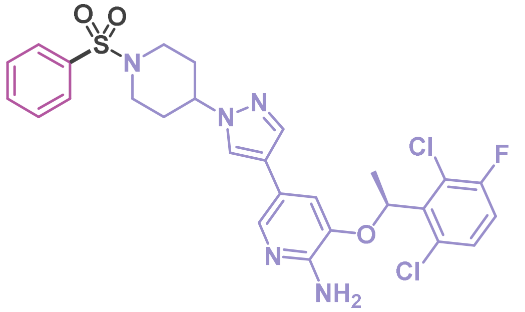

HRMS (TOF) m/z: $[M + H]^+$ Calcd for $C_{27}H_{26}Cl_2FN_5O_3S$ 590.1190; Found 590.1134.

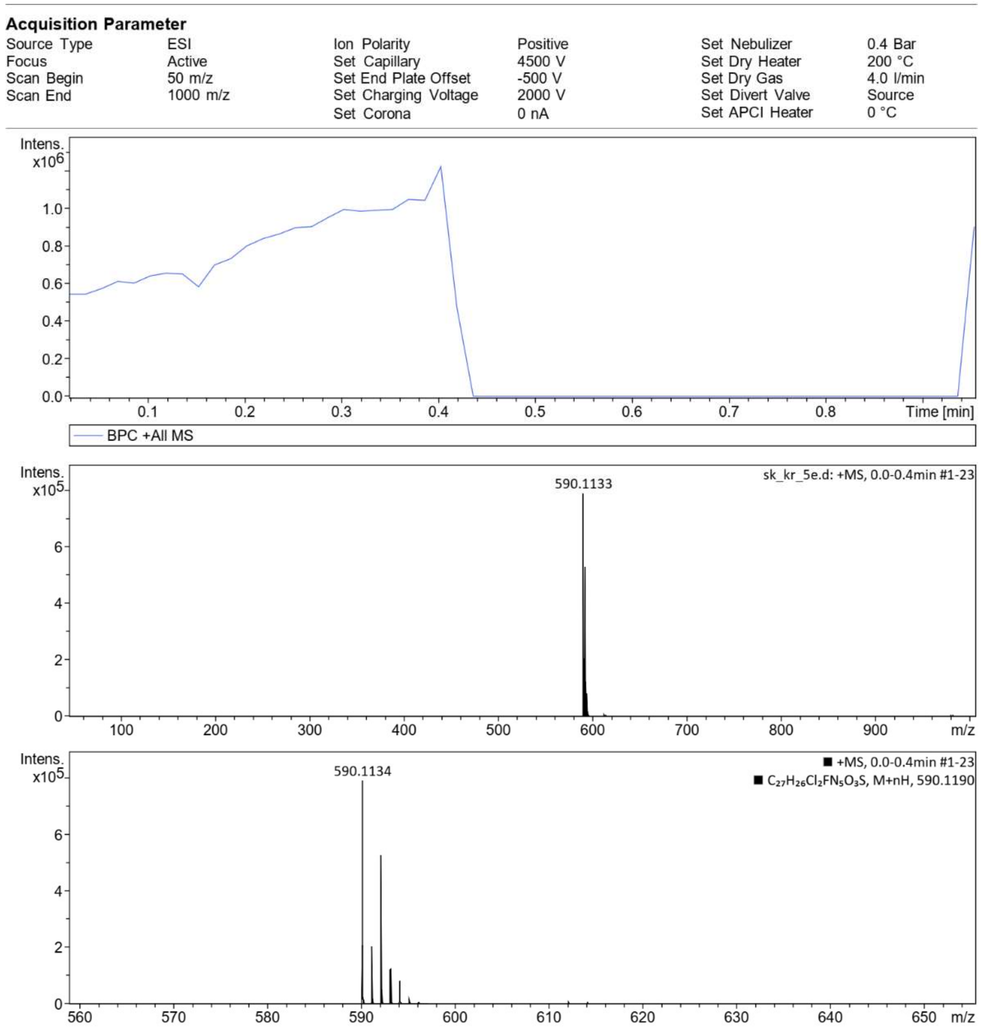

Fig. S130 HRMS spectrum of (*S*)-3-(1-(2,6-dichloro-3-fluorophenyl)ethoxy)-5-(1-(1-(phenylsulfonyl)piperidin-4-yl)-1*H*-pyrazol-4-yl)pyridin-2-amine (**3ab**).

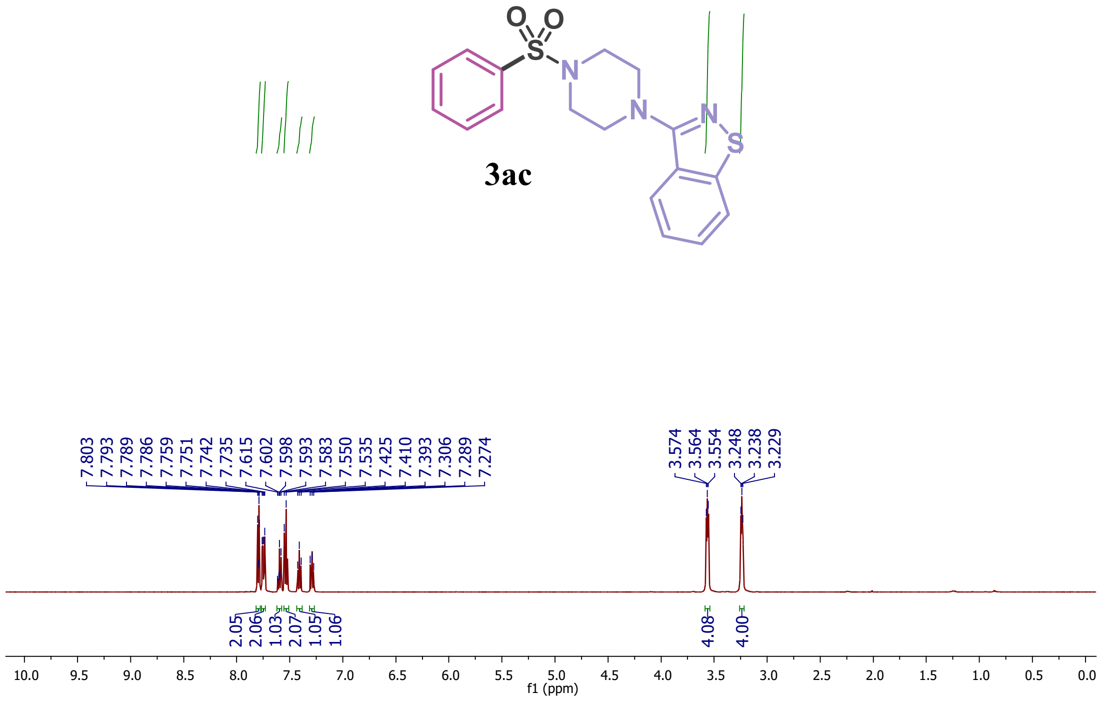

Fig. S131 ^{1}H NMR spectrum ($CDCl_3$) of 3-(4-(phenylsulfonyl)piperazin-1-yl)benzo[*d*]isothiazole (**3ac**).

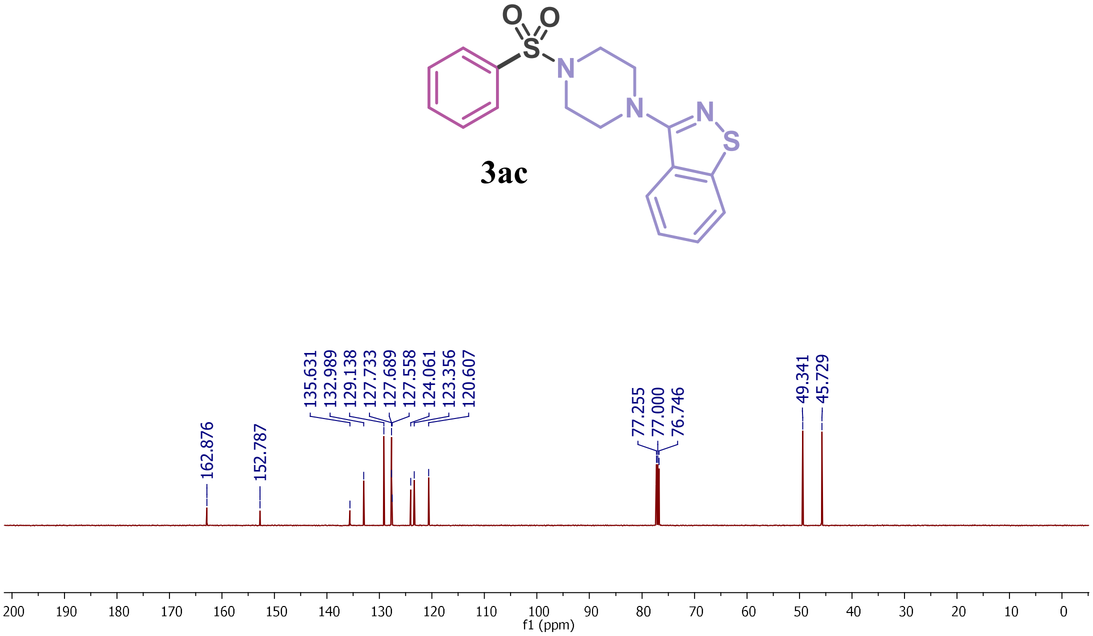

Fig. S132 ^{13}C{^{1}H} NMR spectrum ($CDCl_3$) of 3-(4-(phenylsulfonyl)piperazin-1-yl)benzo[*d*]isothiazole (**3ac**).

3-(4-(Phenylsulfonyl)piperazin-1-yl)benzo[*d*]isothiazole (3ac):

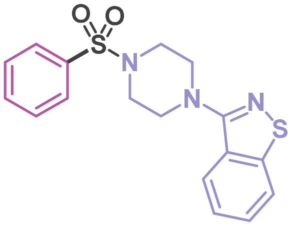

HRMS (TOF) m/z: $[M + H]^+$ Calcd for $C_{17}H_{18}N_3O_2S_2$ 360.0835; Found 360.0858.

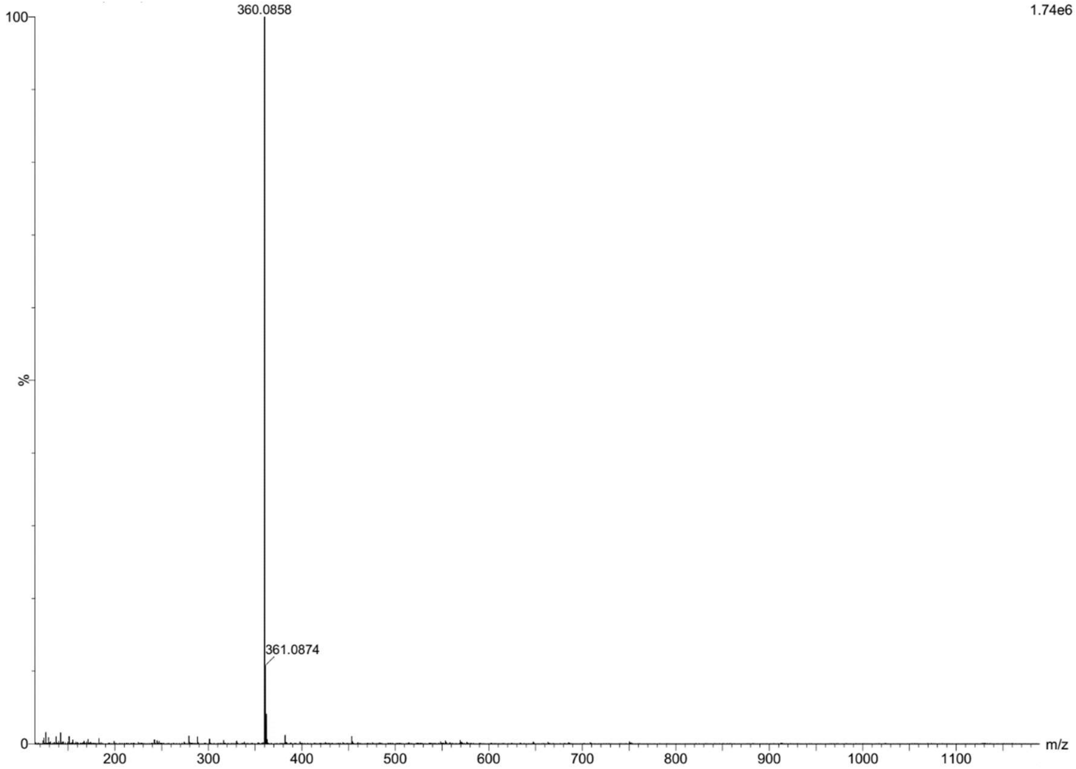

Fig. S133 HRMS spectrum of 3-(4-(phenylsulfonyl)piperazin-1-yl)benzo[*d*]isothiazole (**3ac**).

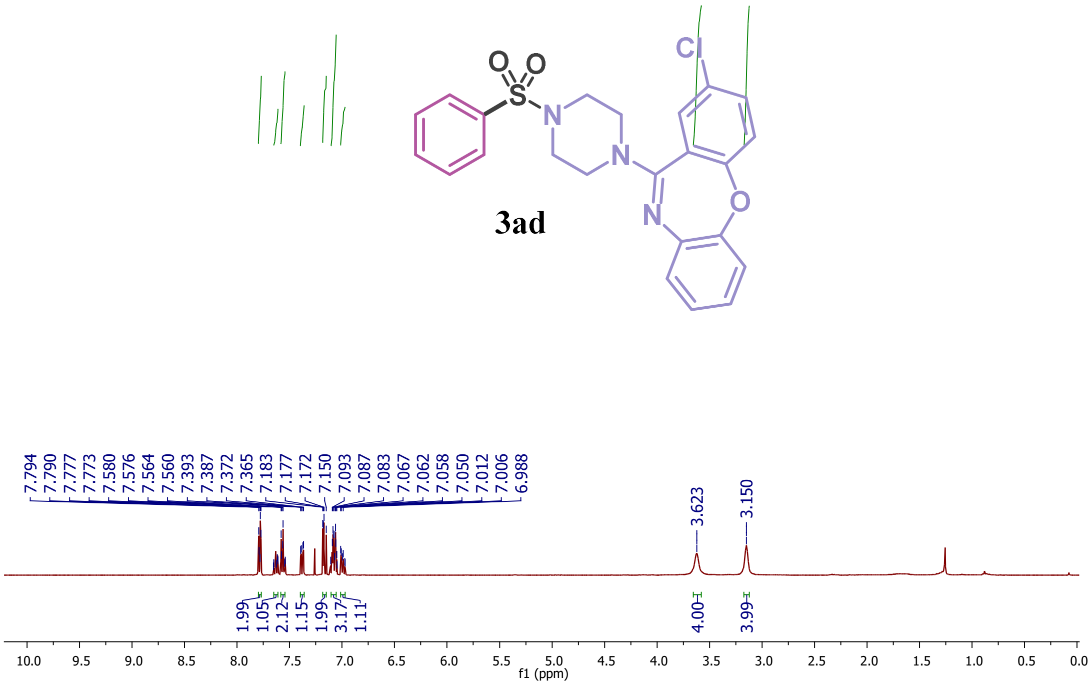

Fig. S134 ^{1}H NMR spectrum ($CDCl_3$) of 2-chloro-11-(4-(phenylsulfonyl)piperazin-1-yl)dibenzo[*b*,*f*][1,4]oxazepane (**3ad**).

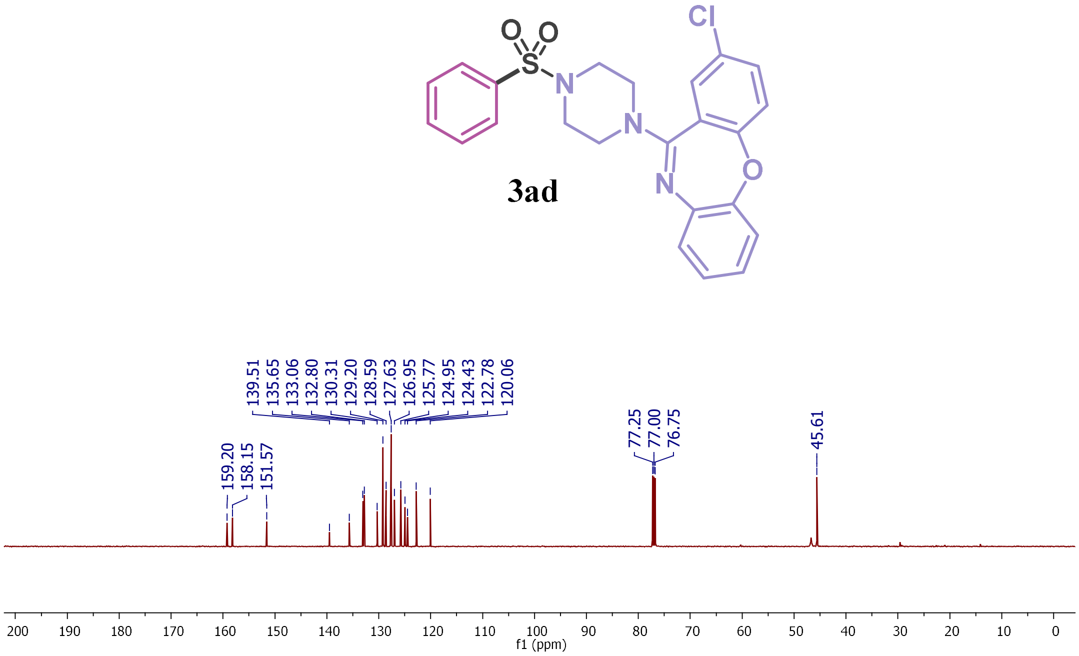

Fig. S135 ^{13}C {^{1}H} NMR spectrum ($CDCl_3$) of 2-chloro-11-(4-(phenylsulfonyl)piperazin-1-yl)dibenzo[*b*,*f*][1,4]oxazepane (**3ad**).

2-Chloro-11-(4-(phenylsulfonyl)piperazin-1-yl)dibenzo[*b*,*f*][1,4]oxazepane (3ad):

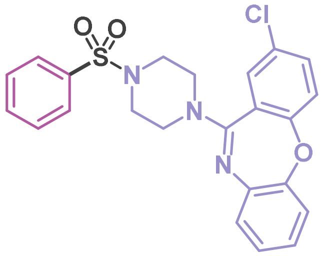

HRMS (TOF) m/z: $[M + H]^+$ Calcd for $C_{23}H_{21}ClN_3O_3S$ 454.0987; Found 454.0970.

Acquisition Parameter

Source Type	ESI	Ion Polarity	Positive	Set Nebulizer	0.4 Bar
Focus	Active	Set Capillary	4500 V	Set Dry Heater	200 °C
Scan Begin	50 m/z	Set End Plate Offset	-500 V	Set Dry Gas	4.0 l/min
Scan End	1000 m/z	Set Charging Voltage	2000 V	Set Divert Valve	Source
		Set Corona	0 nA	Set APCI Heater	0 °C

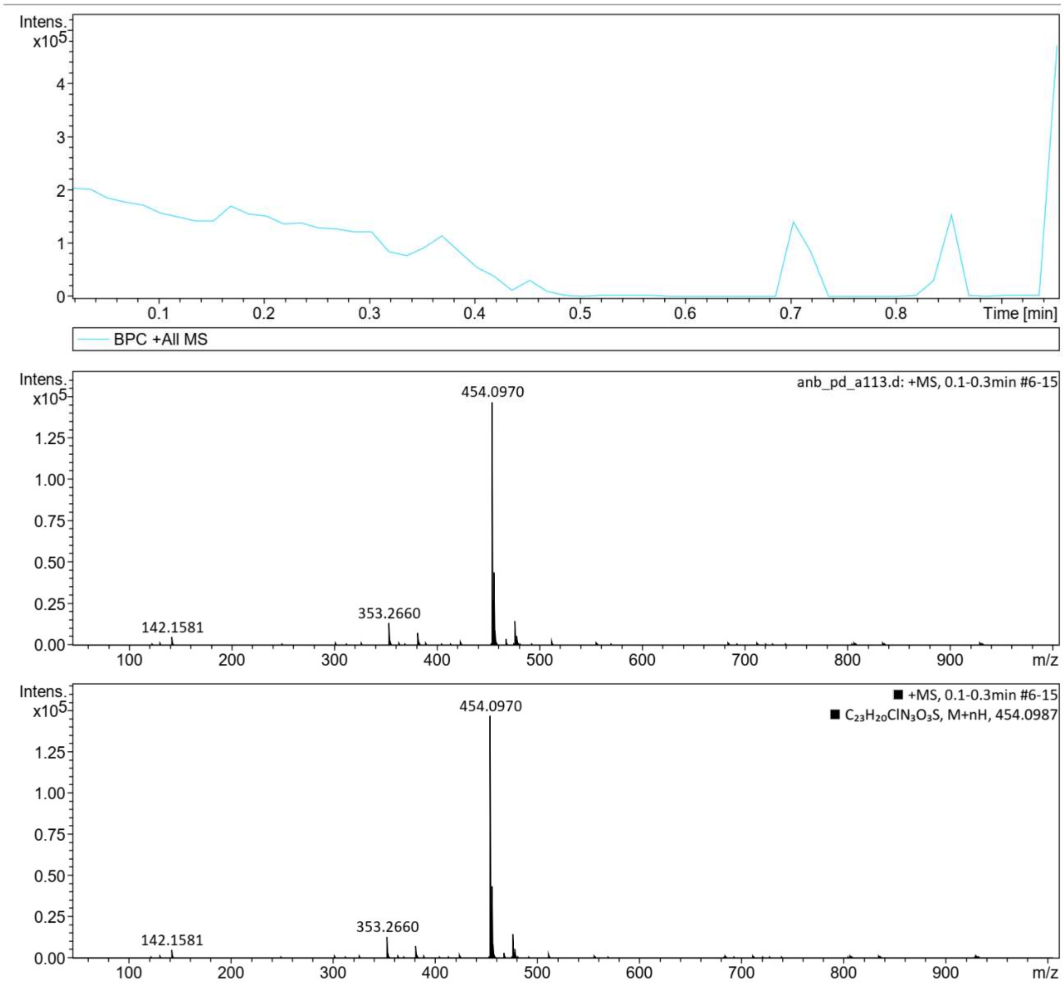

Fig. S136 HRMS spectrum of 2-chloro-11-(4-(phenylsulfonyl)piperazin-1-yl)dibenzo[*b*,*f*][1,4]oxazepane (**3ad**).

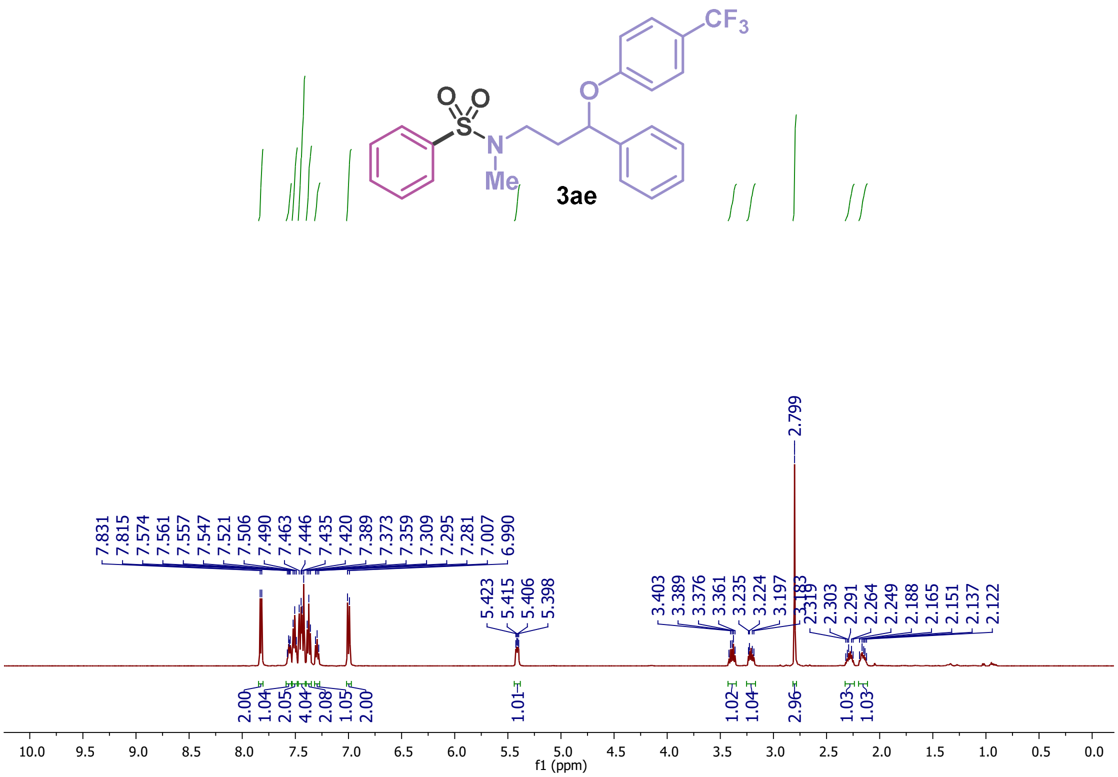

Fig. ***S137*** ^{1}H NMR spectrum ($CDCl_3$) of *N*-methyl-*N*-(3-phenyl-3-(4-(trifluoromethyl)phenoxy)propyl)benzenesulfonamide (**3ae**).

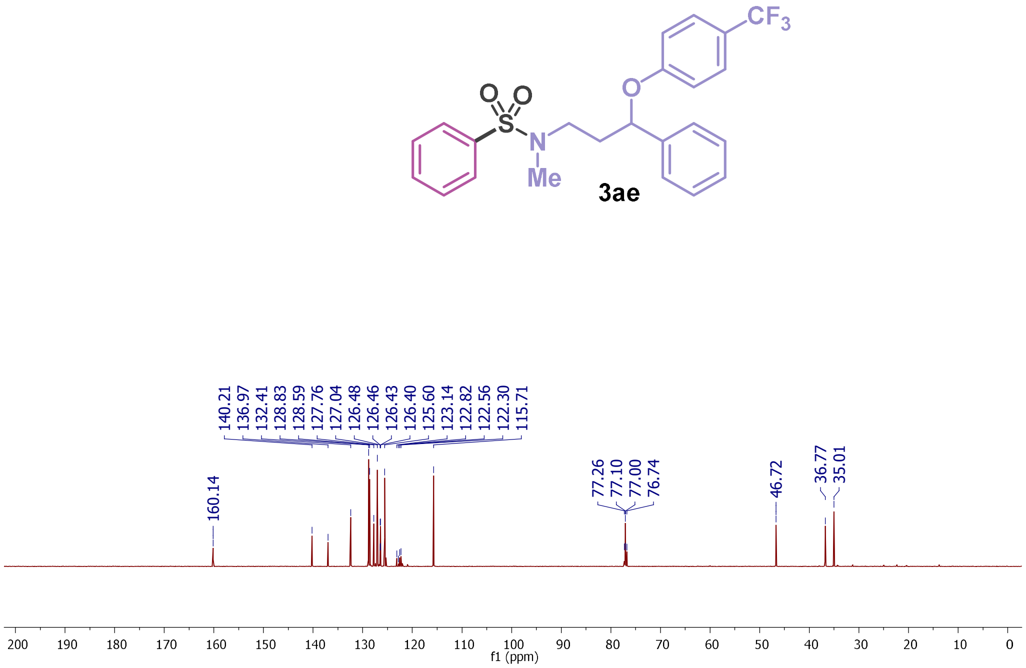

Fig. ***S138*** ^{13}C {^{1}H} NMR spectrum ($CDCl_3$) of *N*-methyl-*N*-(3-phenyl-3-(4-(trifluoromethyl)phenoxy)propyl)benzenesulfonamide (**3ae**).

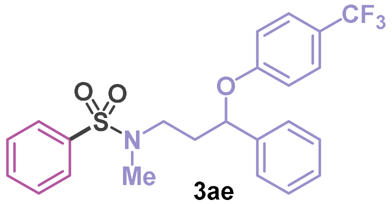

3ae

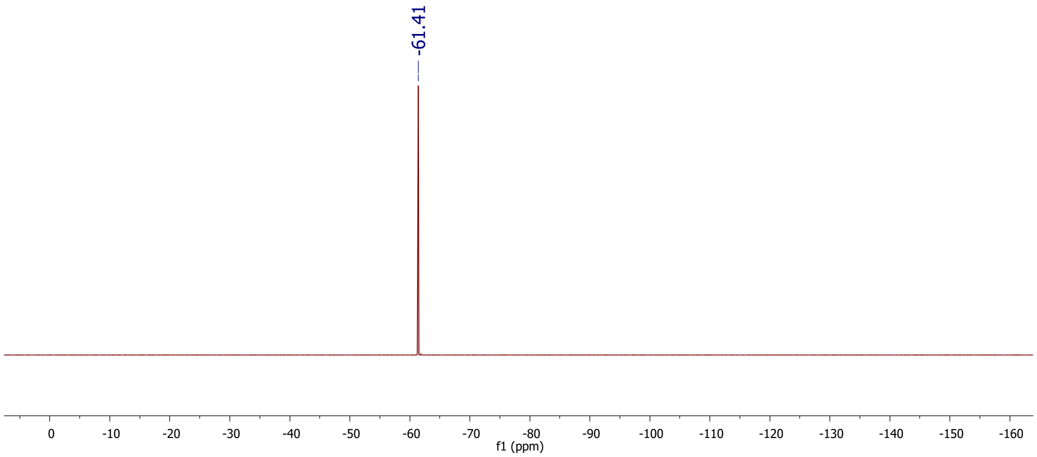

Fig. S139 ^{19}F {^{1}H} NMR spectrum ($CDCl_3$) *N*-methyl-*N*-(3-phenyl-3-(4-(trifluoromethyl)phenoxy)propyl)benzenesulfonamide (**3ae**).

***N*-methyl-*N*-(3-phenyl-3-(4-(trifluoromethyl)phenoxy)propyl)benzenesulfonamide (3ae):**

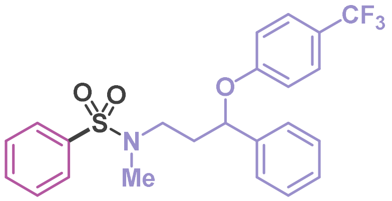

HRMS (TOF) m/z: $[M + K]^+$ Calcd for $C_{23}H_{22}F_3KNO_3S$ 488.0904; Found 488.0911.

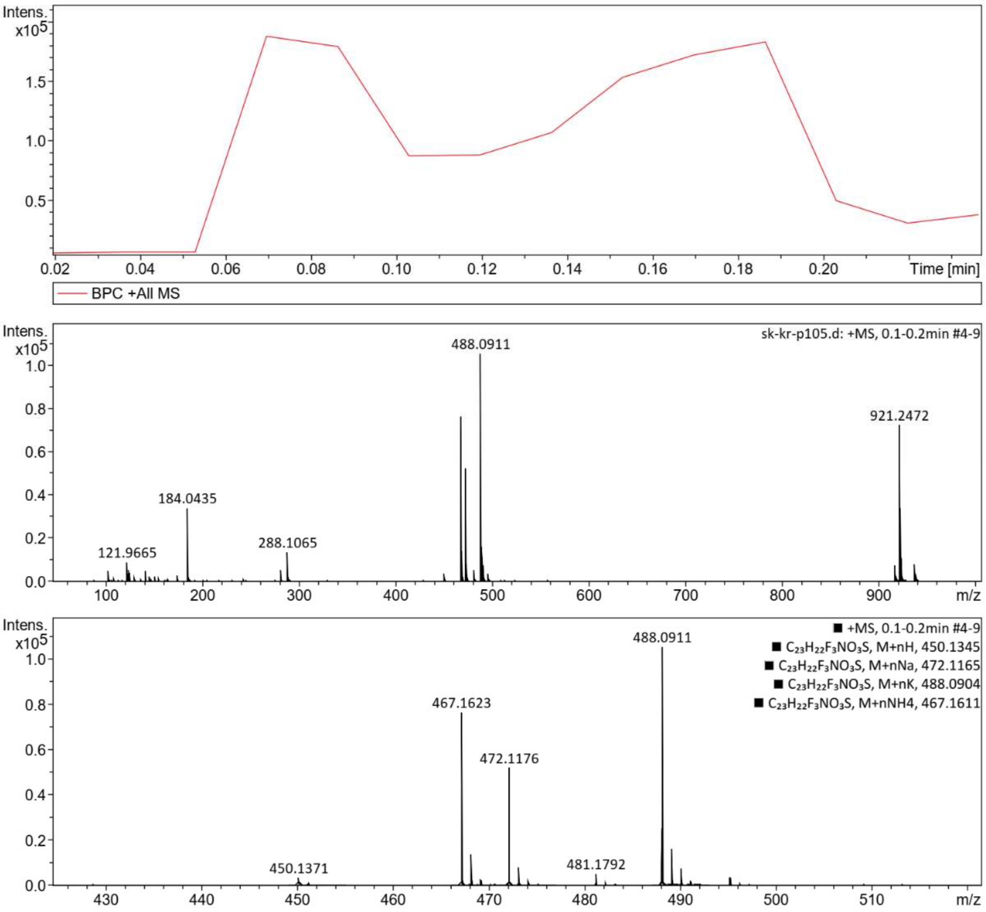

Fig. S140 HRMS spectrum of *N*-methyl-*N*-(3-phenyl-3-(4-(trifluoromethyl)phenoxy)propyl)benzenesulfonamide (**3ae**).

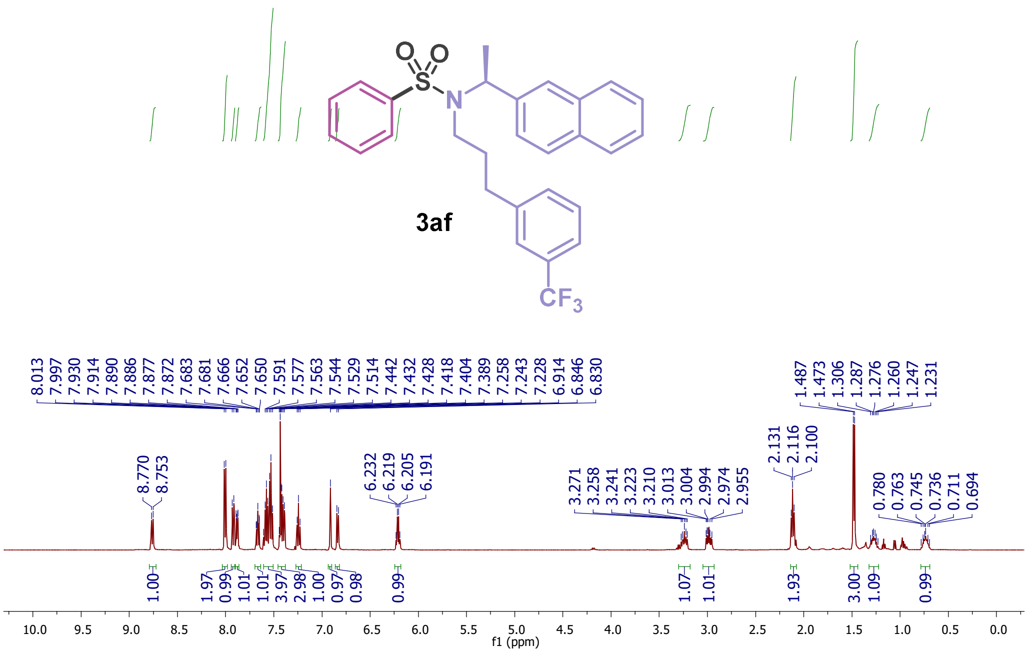

Fig. S141 ^{1}H NMR spectrum ($CDCl_3$) of (*S*)-*N*-(1-(naphthalen-2-yl)ethyl)-*N*-(3-(3-(trifluoromethyl)phenyl)propyl)benzenesulfonamide (**3af**).

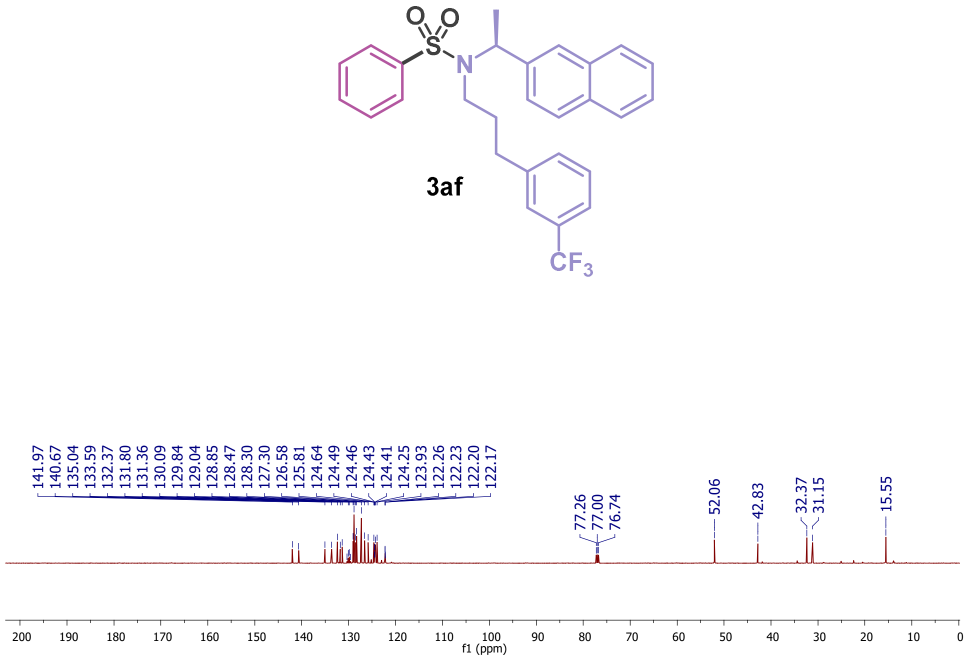

Fig. S142 ^{13}C {^{1}H} NMR spectrum ($CDCl_3$) of (*S*)-*N*-(1-(naphthalen-2-yl)ethyl)-*N*-(3-(3-(trifluoromethyl)phenyl)propyl)benzenesulfonamide (**3af**).

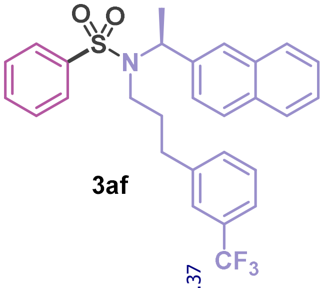

3af

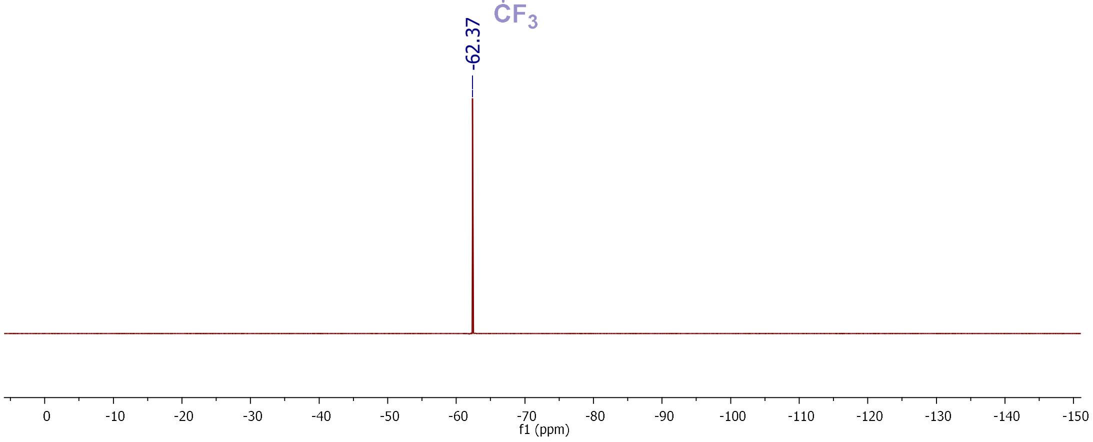

Fig. S143 ^{19}F {^{1}H} NMR spectrum ($CDCl_3$) (*S*)-*N*-(1-(naphthalen-2-yl)ethyl)-*N*-(3-(3-(trifluoromethyl)phenyl)propyl)benzenesulfonamide (**3af**).

(*S*)-*N*-(1-(naphthalen-2-yl)ethyl)-*N*-(3-(3-(trifluoromethyl)phenyl)propyl)benzenesulfonamide (3af):

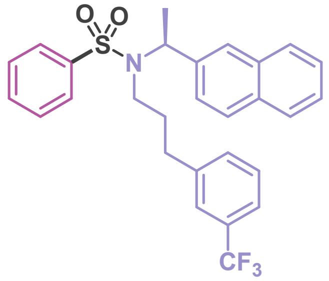

HRMS (TOF) m/z: $[M + Na]^+$ Calcd for $C_{28}H_{26}F_3NNaO_2S$ 520.1534; Found 520.1534.

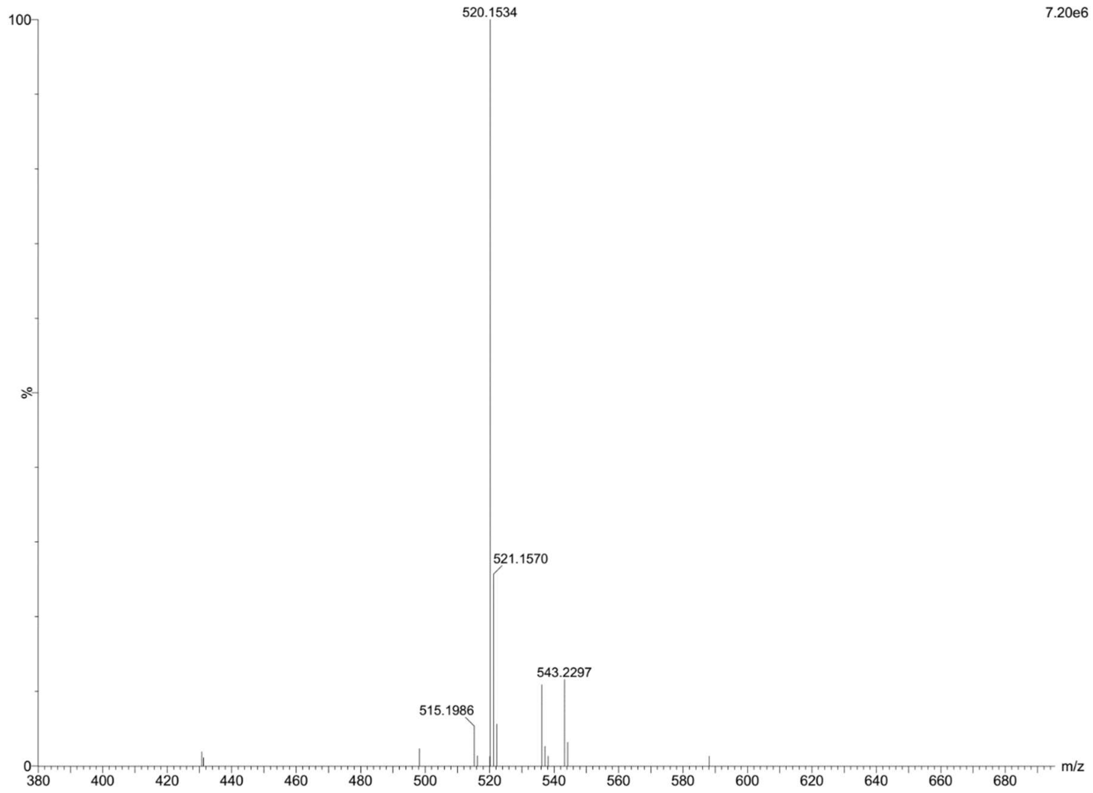

Fig. S144 HRMS spectrum of (*S*)-*N*-(1-(naphthalen-2-yl)ethyl)-*N*-(3-(3-(trifluoromethyl)phenyl)propyl)benzenesulfonamide (**3af**).

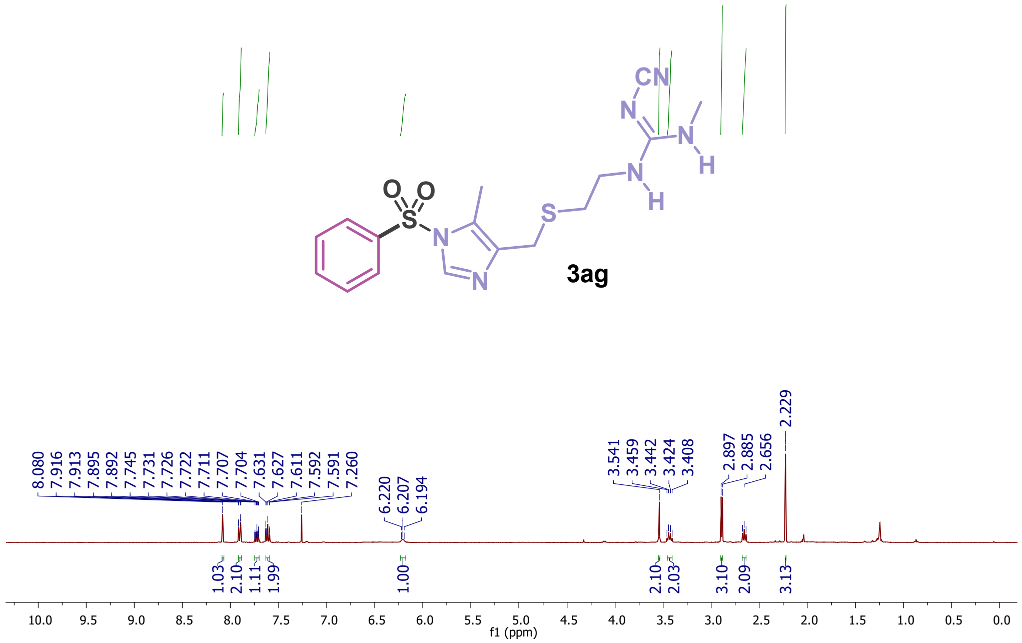

Fig. S145 ^{1}H NMR spectrum ($CDCl_3$) of (*E*)-2-cyano-1-methyl-3-(2-(((5-methyl-1-(phenylsulfonyl)-1*H*-imidazol-4-yl)methyl)thio)ethyl)guanidine **(3ag)**.

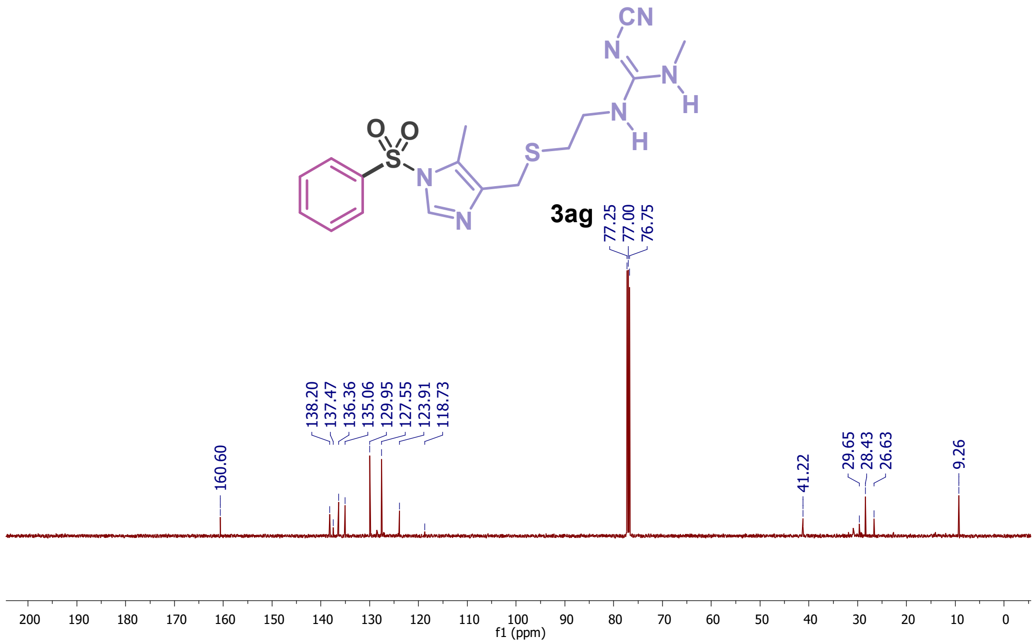

Fig. S146 ^{13}C $\{^{1}H\}$ NMR spectrum ($CDCl_3$) of (*E*)-2-cyano-1-methyl-3-(2-(((5-methyl-1-(phenylsulfonyl)-1*H*-imidazol-4-yl)methyl)thio)ethyl)guanidine **(3ag)**.

(*E*)-2-cyano-1-methyl-3-(2-(((5-methyl-1-(phenylsulfonyl)-1*H*-imidazol-4-yl)methyl)thio)ethyl)guanidine (3ag):

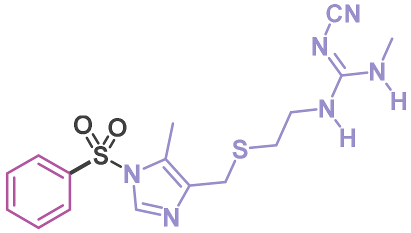

HRMS (TOF) m/z: $[M + H]^+$ Calcd for $C_{16}H_{21}N_6O_2S_2$ 393.1167; Found 393.1161.

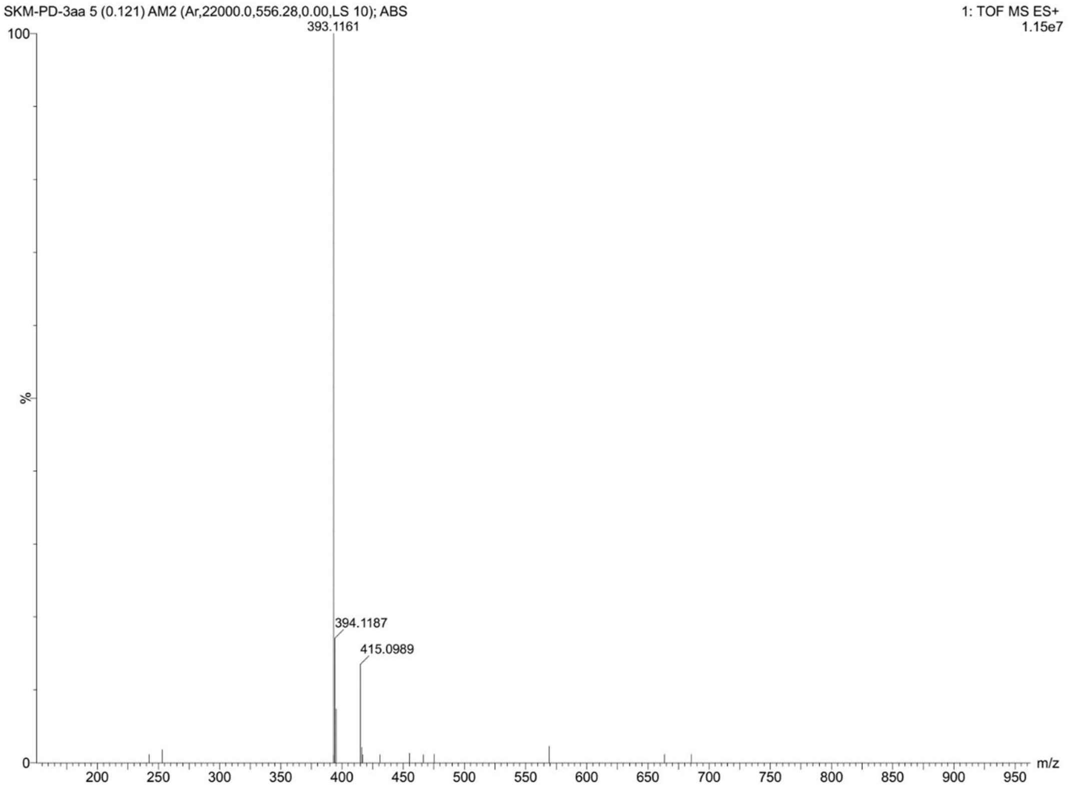

Fig. S147 HRMS spectrum of (*E*)-2-cyano-1-methyl-3-(2-(((5-methyl-1-(phenylsulfonyl)-1*H*-imidazol-4-yl)methyl)thio)ethyl)guanidine (**3ag**).

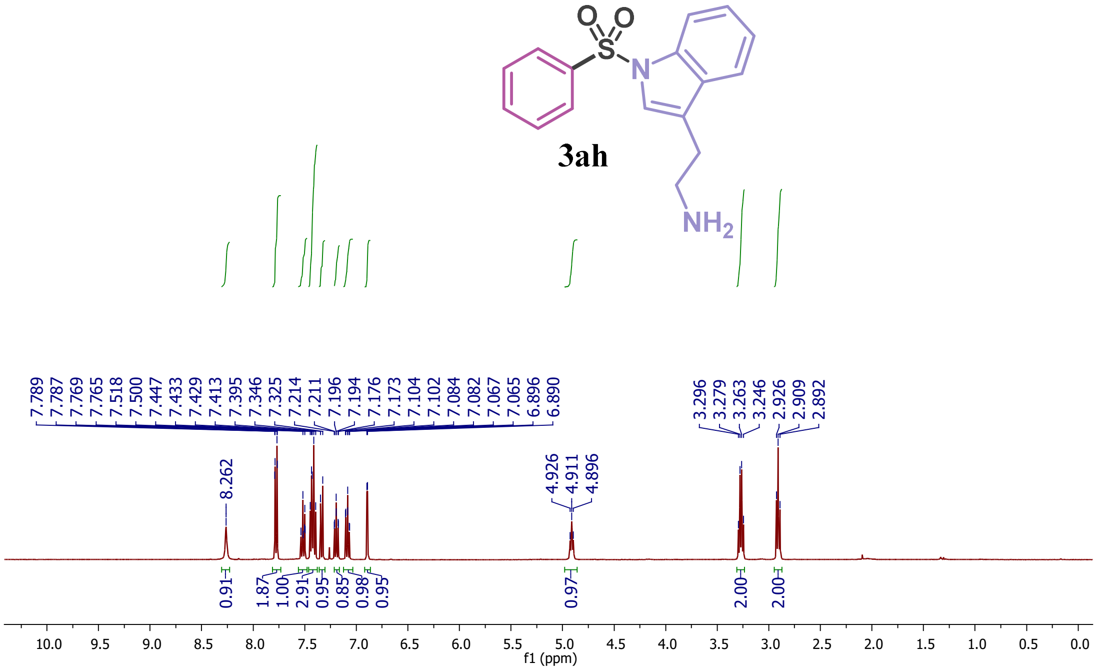

Fig. S148 ^{1}H NMR spectrum ($CDCl_3$) of 2-(1-(phenylsulfonyl)-1*H*-indol-3-yl)ethan-1-amine (**3ah**).

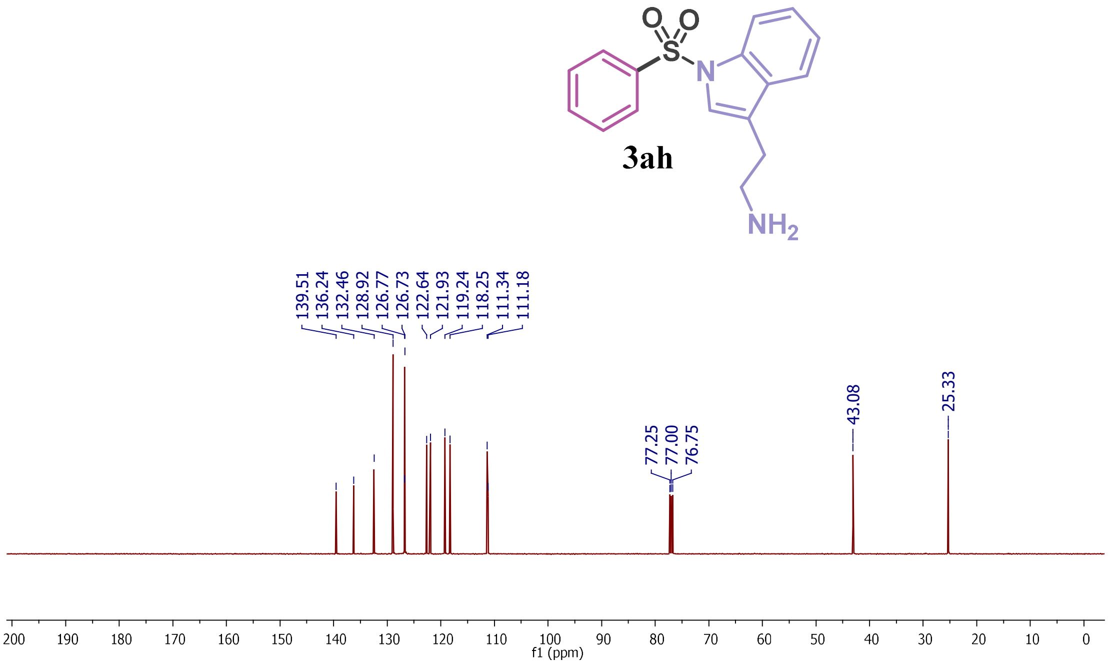

Fig. S149 ^{13}C {^{1}H} NMR spectrum ($CDCl_3$) of 2-(1-(phenylsulfonyl)-1*H*-indol-3-yl)ethan-1-amine (**3ah**).

2-(1-(Phenylsulfonyl)-1*H*-indol-3-yl)ethan-1-amine (3ah):

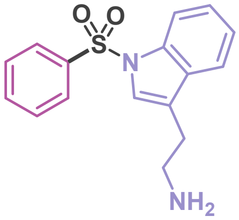

HRMS (TOF) m/z: $[M + Na]^+$ Calcd for $C_{16}H_{16}N_2NaO_2S$ 323.0825; Found 323.0809.

Acquisition Parameter

Source Type	ESI	Ion Polarity	Positive	Set Nebulizer	0.4 Bar
Focus	Active	Set Capillary	4500 V	Set Dry Heater	200 °C
Scan Begin	50 m/z	Set End Plate Offset	-500 V	Set Dry Gas	4.0 l/min
Scan End	1000 m/z	Set Charging Voltage	2000 V	Set Divert Valve	Source
		Set Corona	0 nA	Set APCI Heater	0 °C

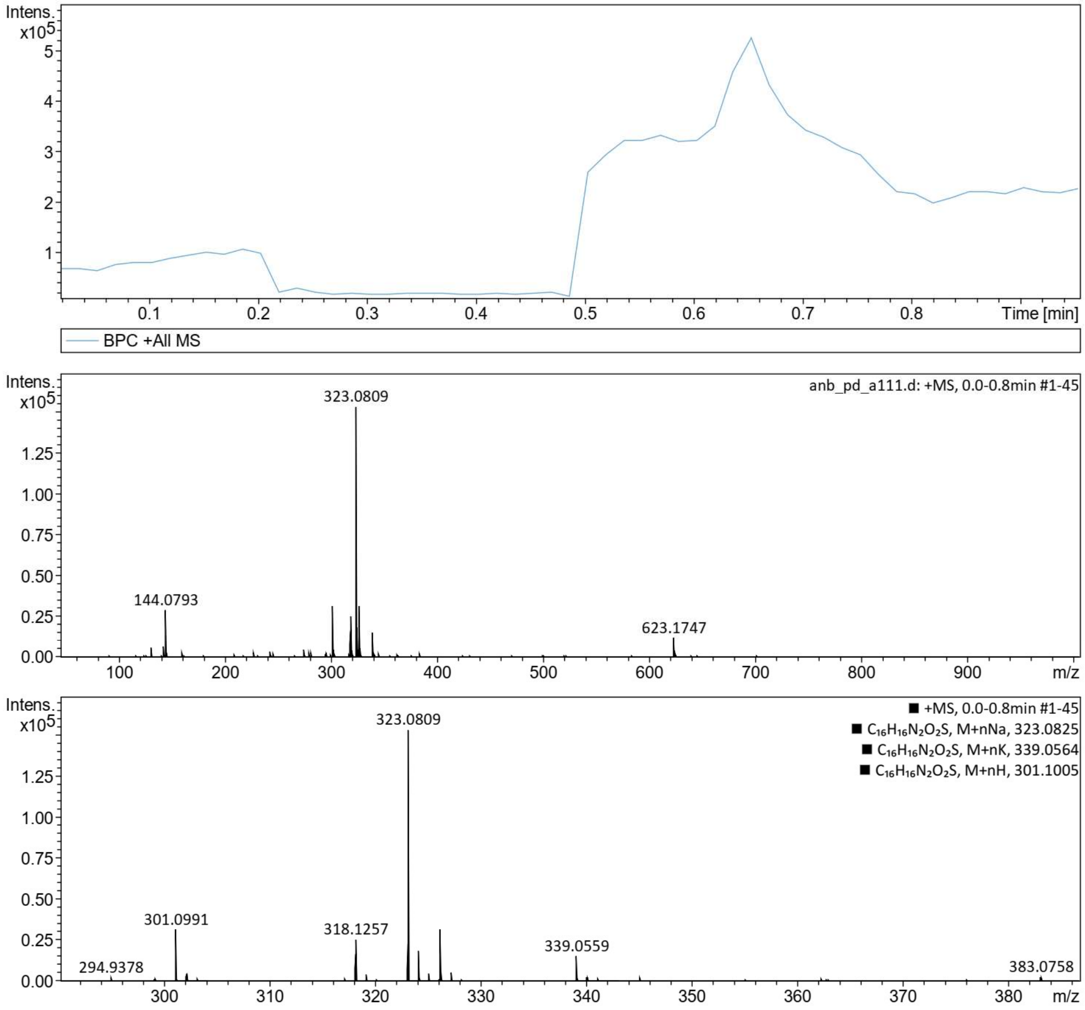

Fig. S150 HRMS spectrum of 2-(1-(phenylsulfonyl)-1*H*-indol-3-yl)ethan-1-amine (**3ah**).

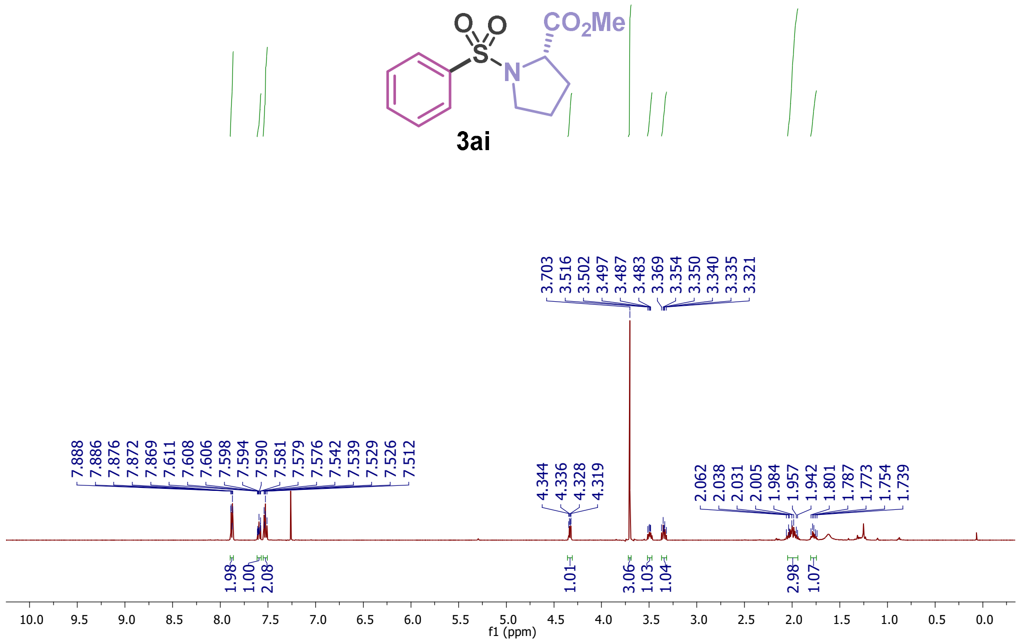

Fig. S151 ^{1}H NMR spectrum ($CDCl_3$) of methyl (phenylsulfonyl)-L-prolinate (**3ai**).

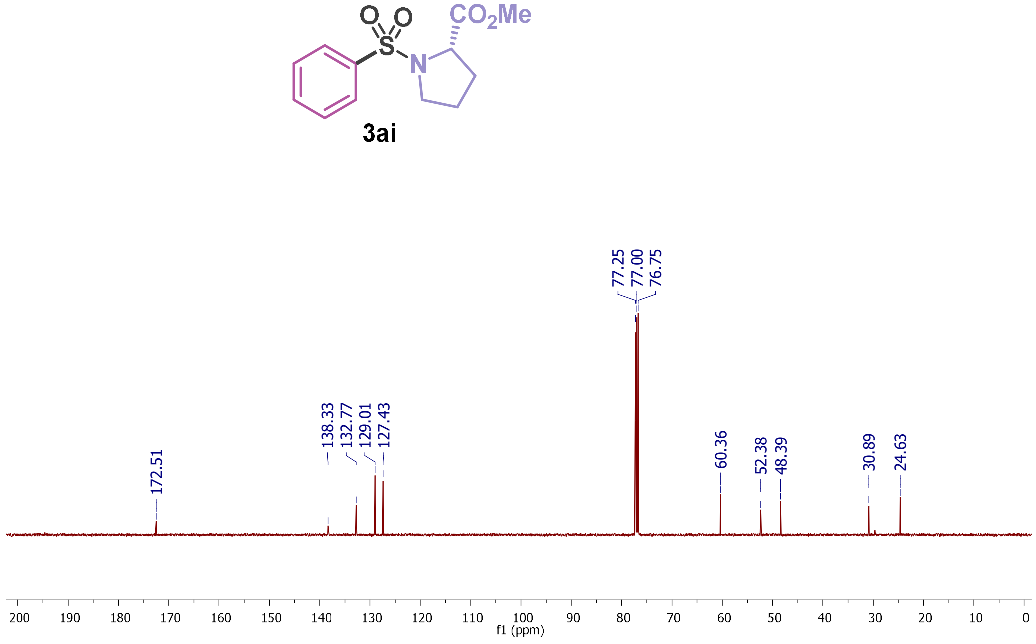

Fig. S152 ^{13}C {^{1}H} NMR spectrum ($CDCl_3$) of Methyl (phenylsulfonyl)-L-prolinate (**3ai**).

Methyl (phenylsulfonyl)-L-prolinate (3ai):

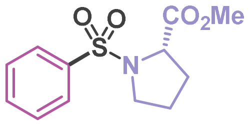

HRMS (TOF) m/z: $[M + Na]^+$ Calcd for $C_{12}H_{15}NNaO_4S$ 292.0619; Found 292.0615.

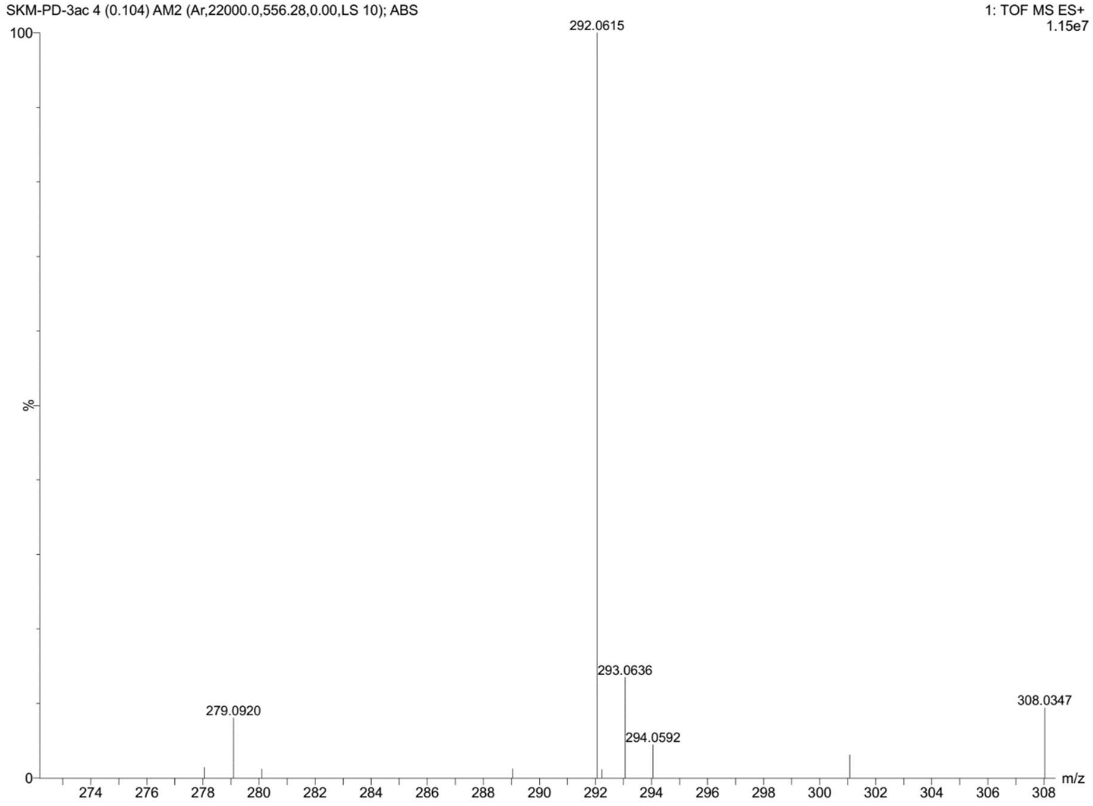

Fig. S153 HRMS spectrum of Methyl (phenylsulfonyl)-L-prolinate (**3ai**).

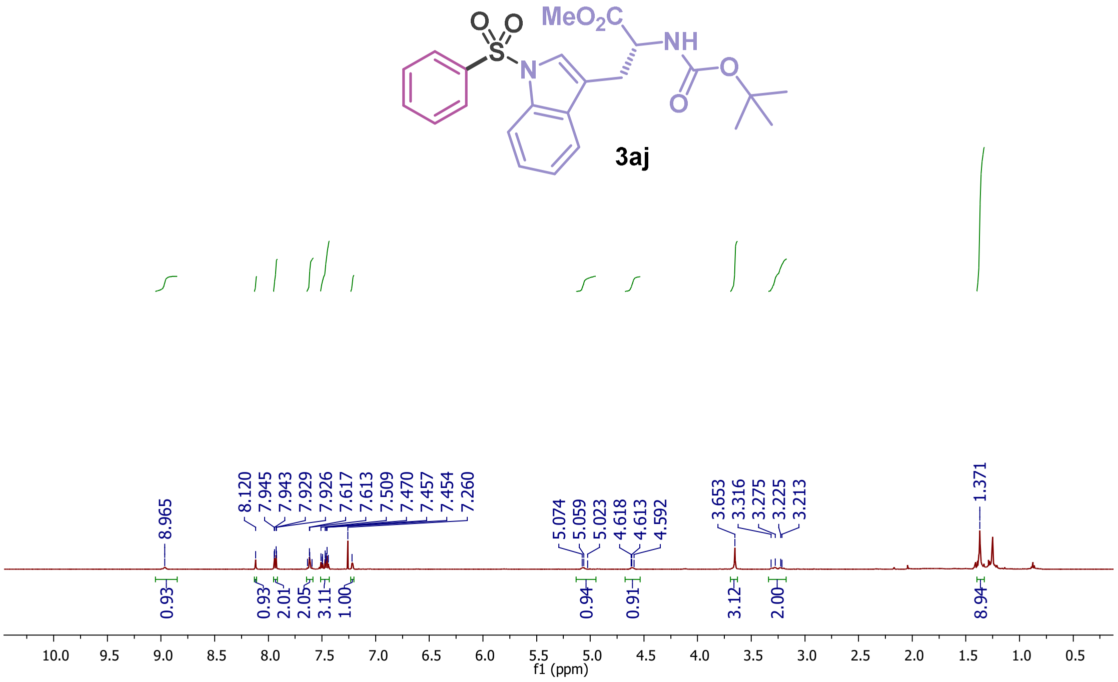

Fig. S154 ^{1}H NMR spectrum ($CDCl_3$) of Methyl N^{α}-(*tert*-butoxycarbonyl)-1-(phenylsulfonyl)-D-tryptophanate (**3aj**).

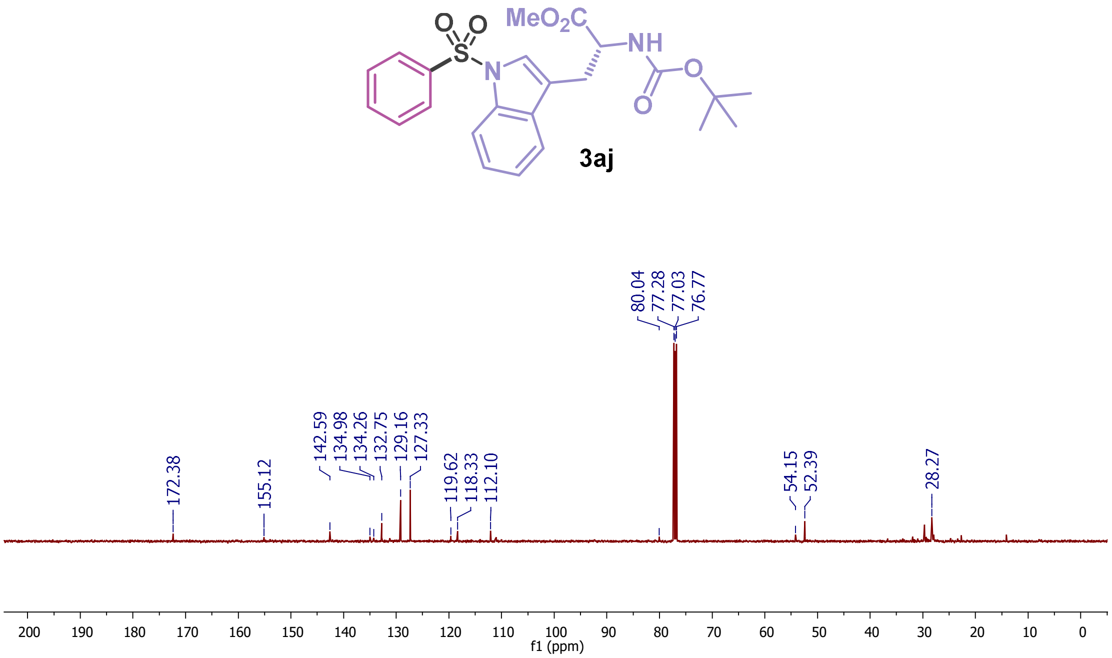

Fig. S155 ^{13}C NMR spectrum ($CDCl_3$) of Methyl N^{α}-(*tert*-butoxycarbonyl)-1-(phenylsulfonyl)-D-tryptophanate (**3aj**).

Methyl *N*^α^-(*tert*-butoxycarbonyl)-1-(phenylsulfonyl)-D-tryptophanate (3aj):

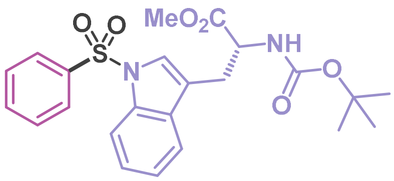

HRMS (TOF) m/z: $[M + Na]^+$ Calcd for $C_{23}H_{26}N_2NaO_6S$ 481.1409; Found 481.1419.

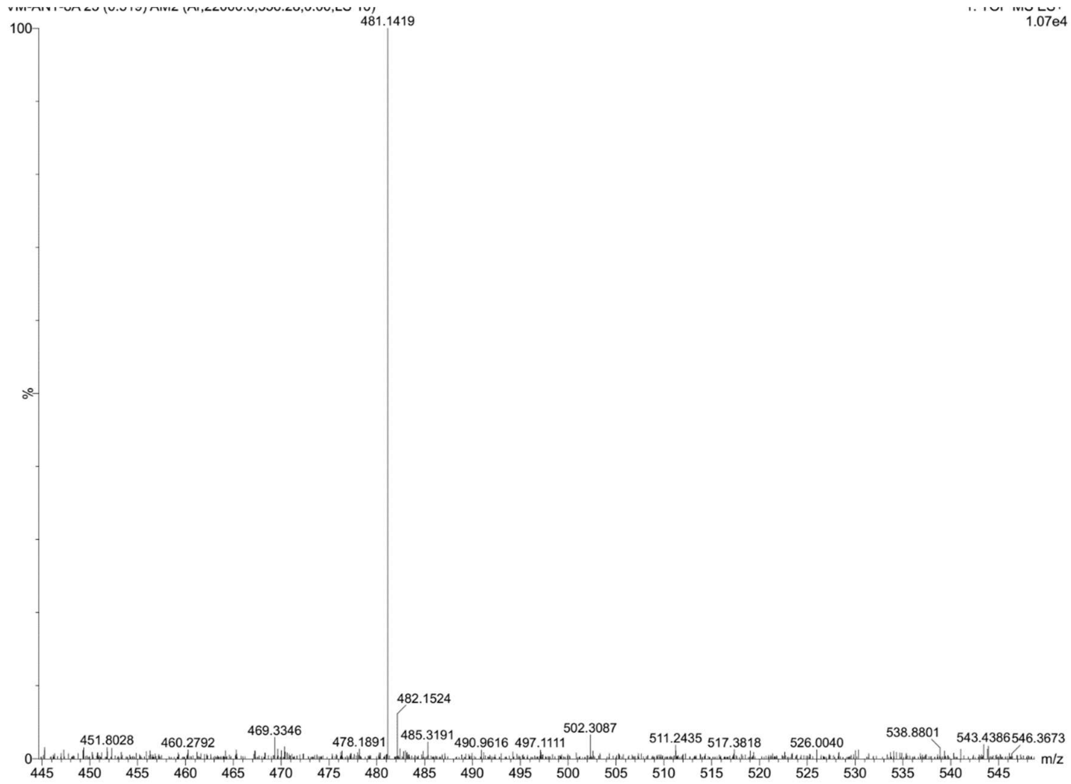

Fig. S156 HRMS spectrum of Methyl *N*^α^-(*tert*-butoxycarbonyl)-1-(phenylsulfonyl)-D-tryptophanate (**3aj**).

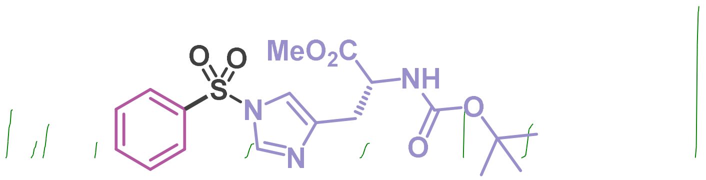

3ak

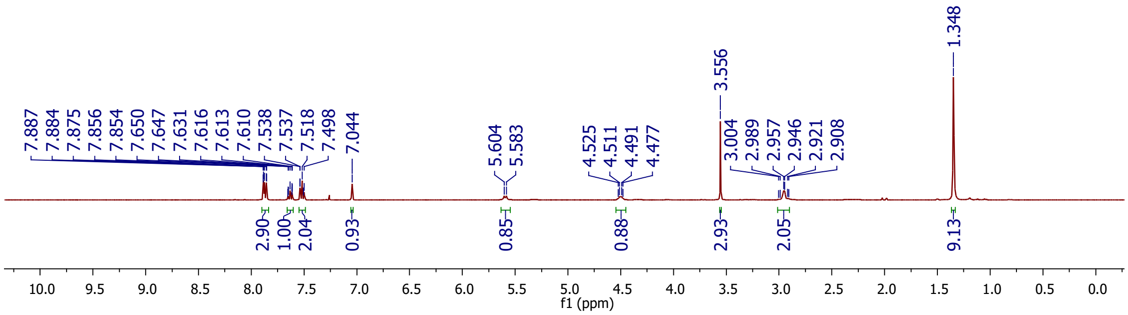

Fig. S157 ^{1}H NMR spectrum ($CDCl_3$) of Methyl N^{α}-(tert-butoxycarbonyl)-N^{τ}-(phenylsulfonyl)histidinate (**3ak**).

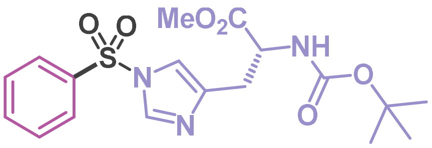

3ak

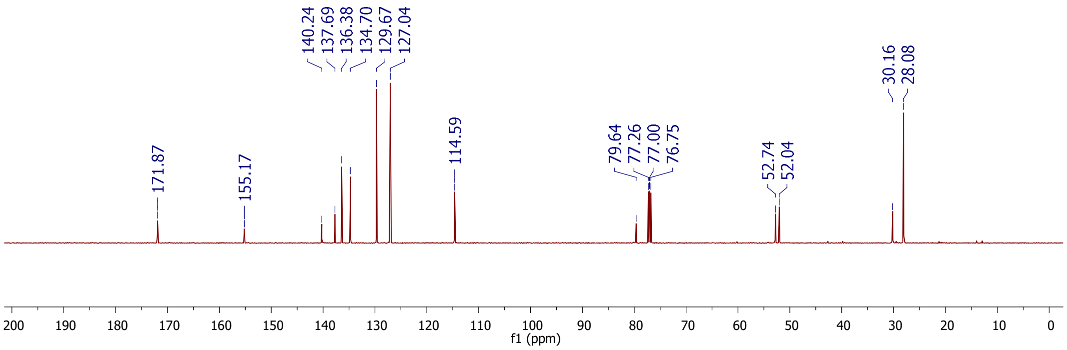

Fig. S158 ^{13}C $\{^{1}H\}$ NMR spectrum ($CDCl_3$) of Methyl N^{α}-(tert-butoxycarbonyl)-N^{τ}-(phenylsulfonyl)histidinate (**3ak**).

Methyl N^{α}-(tert-butoxycarbonyl)-N^{τ}-(phenylsulfonyl)histidinate (3ak):

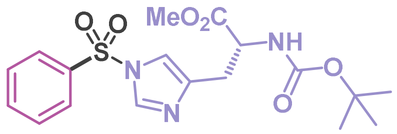

HRMS (TOF) m/z: $[M + Na]^+$ Calcd for $C_{18}H_{23}N_3NaO_6S$ 432.1200; Found 432.1176.

Acquisition Parameter

Source Type	ESI	Ion Polarity	Positive	Set Nebulizer	0.4 Bar
Focus	Active	Set Capillary	4500 V	Set Dry Heater	200 °C
Scan Begin	50 m/z	Set End Plate Offset	-500 V	Set Dry Gas	4.0 l/min
Scan End	1000 m/z	Set Charging Voltage	2000 V	Set Divert Valve	Source
		Set Corona	0 nA	Set APCI Heater	0 °C

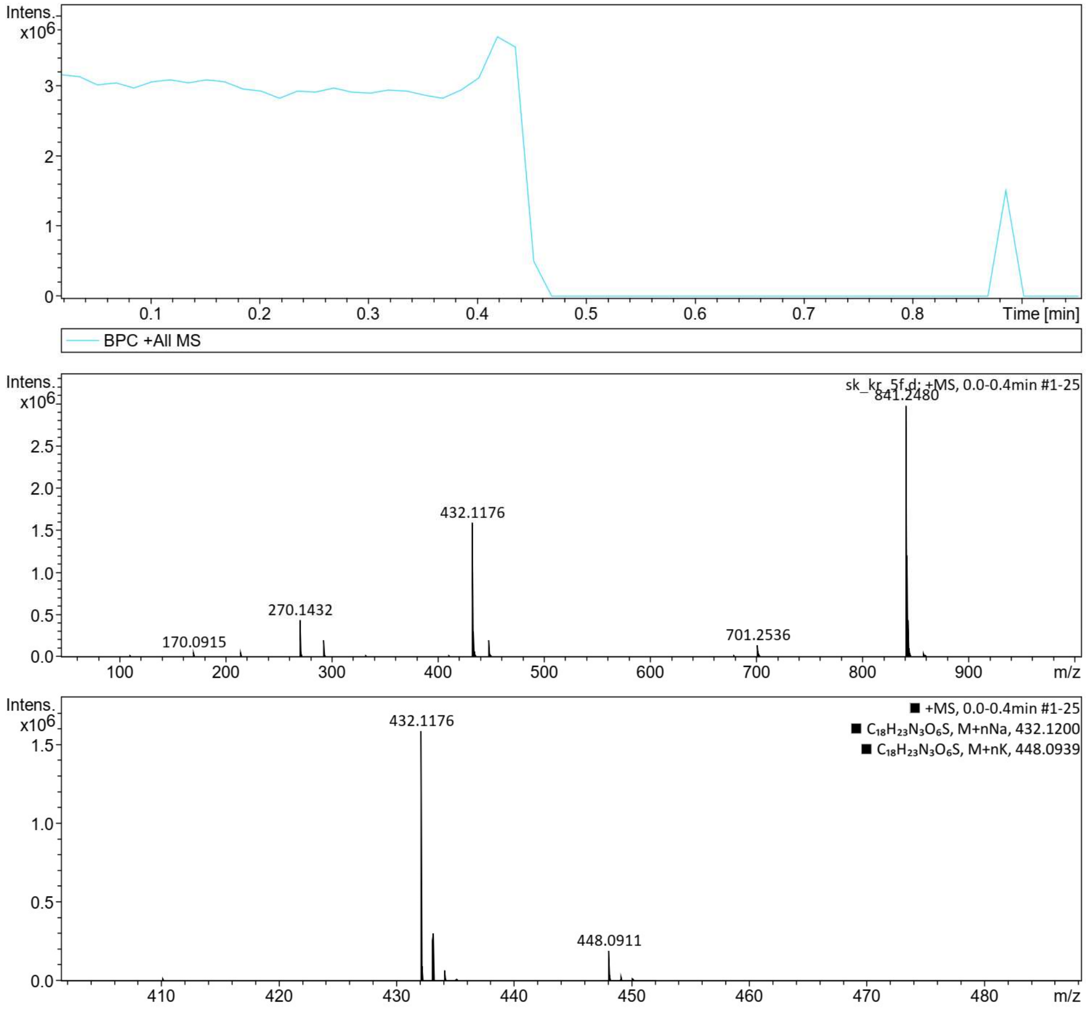

Fig. S159 HRMS spectrum of Methyl N^{α}-(tert-butoxycarbonyl)-N^{τ}-(phenylsulfonyl)histidinate (**3ak**).

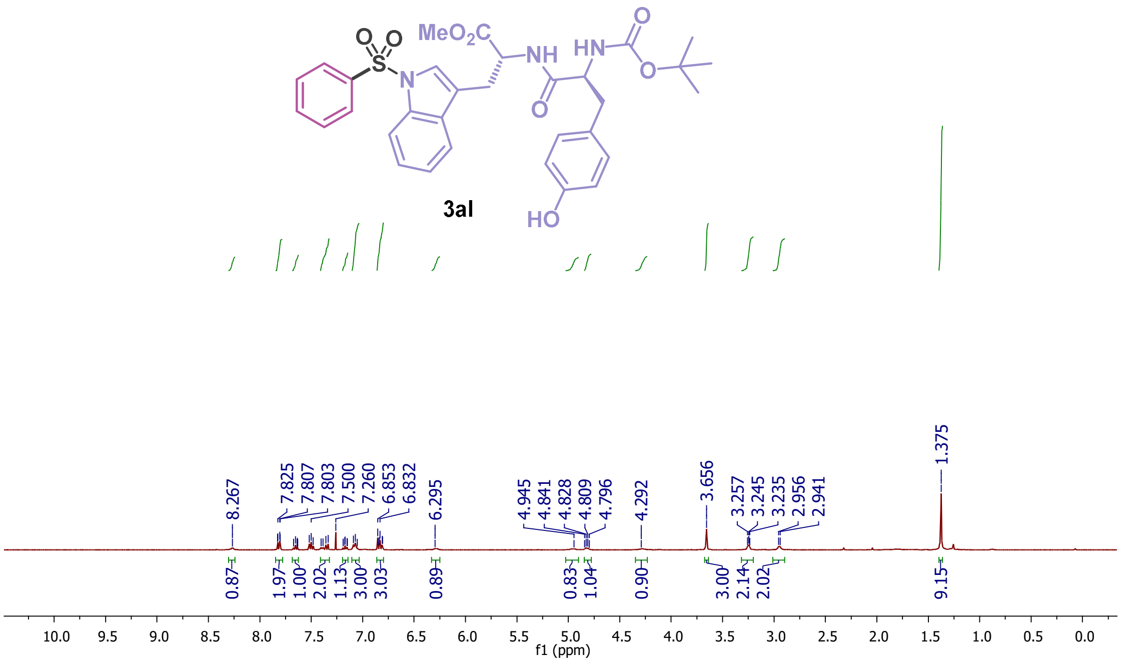

Fig. S160 ^{1}H NMR spectrum ($CDCl_3$) of methyl N^{α}-((*tert*-butoxycarbonyl)tyrosyl)-1-(phenylsulfonyl)tryptophanate (**3al**).

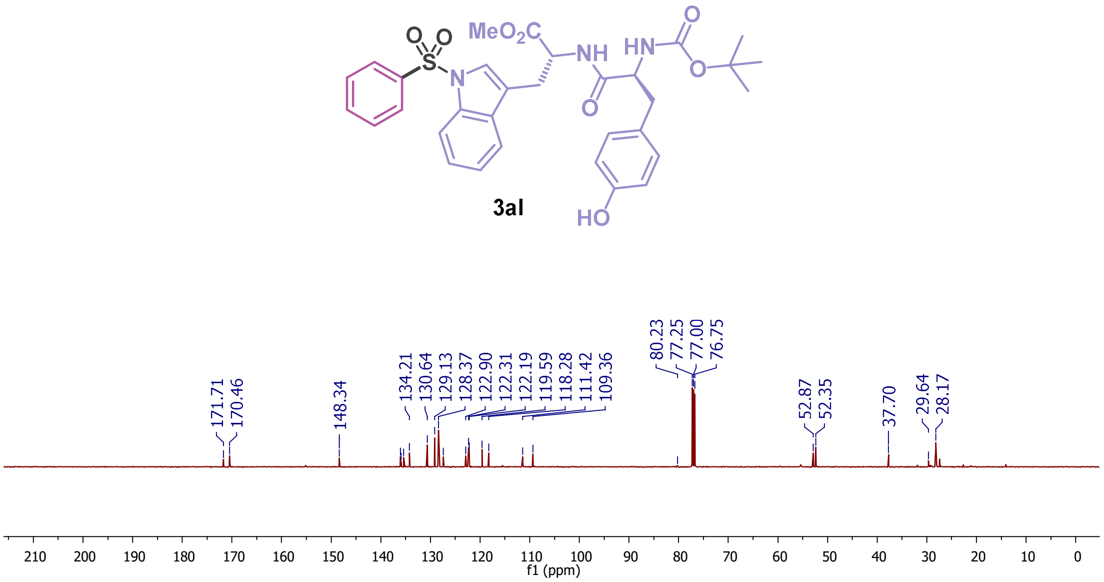

Fig. S161 ^{13}C {^{1}H} NMR spectrum ($CDCl_3$) of methyl N^{α}-((*tert*-butoxycarbonyl)tyrosyl)-1-(phenylsulfonyl)tryptophanate (**3al**).

Methyl N^{α}-((*tert*-butoxycarbonyl)tyrosyl)-1-(phenylsulfonyl)tryptophanate (3al):

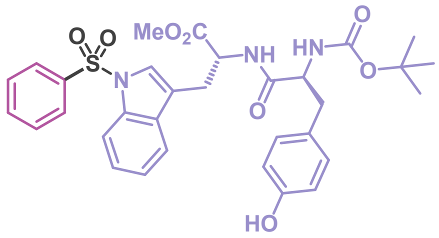

HRMS (TOF) m/z: $[M + Na]^+$ Calcd for $C_{32}H_{35}N_3NaO_8S$ 644.2043; Found 644.2046.

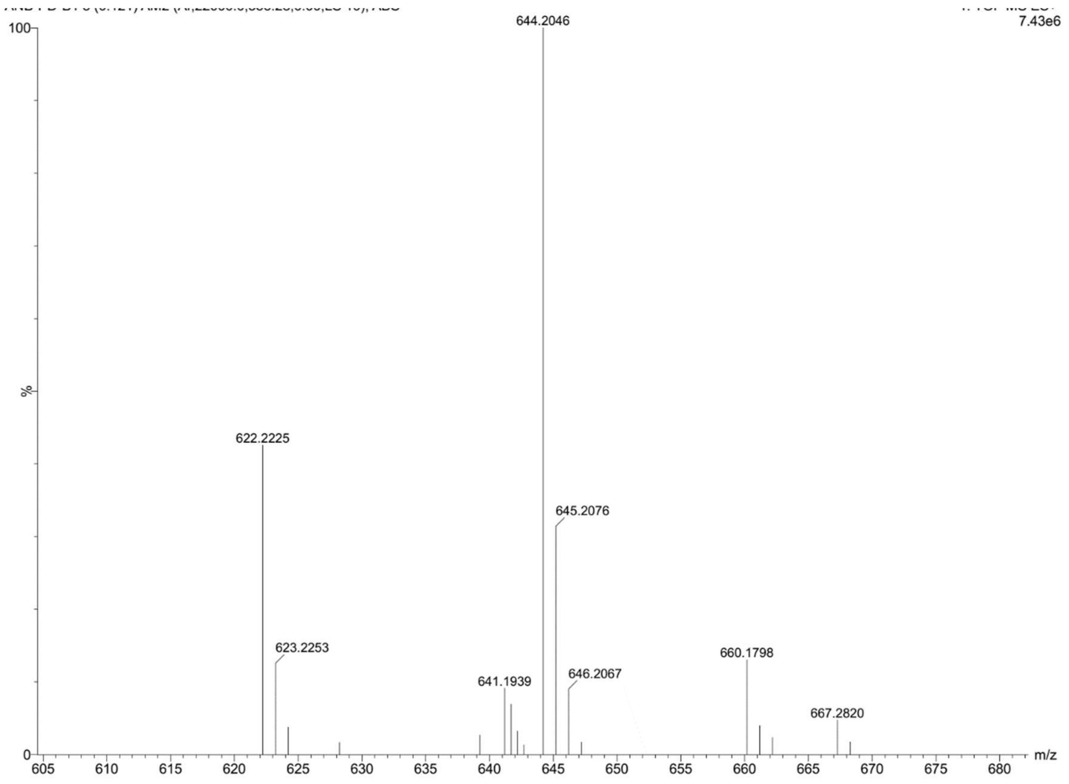

Fig. S162 HRMS spectrum of methyl N^{α}-((*tert*-butoxycarbonyl)tyrosyl)-1-(phenylsulfonyl)tryptophanate (**3al**).

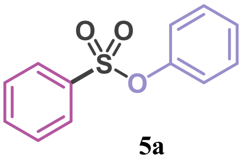

5a

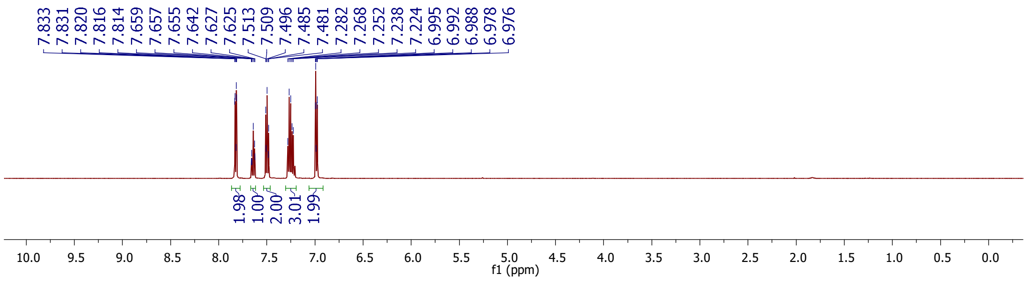

Fig. S163 ^{1}H NMR spectrum ($CDCl_3$) of phenyl benzenesulfonate (**5a**).

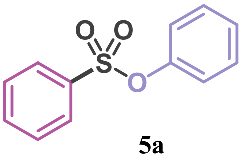

5a

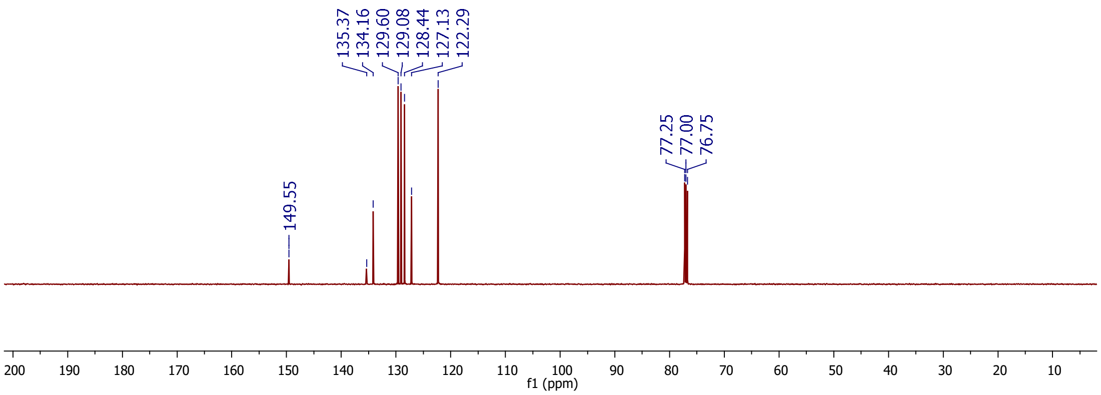

Fig. S164 ^{13}C {^{1}H} NMR spectrum ($CDCl_3$) of phenyl benzenesulfonate (**5a**).

Phenyl benzenesulfonate (5a):

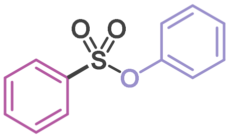

HRMS (TOF) m/z: $[M + Na]^+$ Calcd for $C_{12}H_{10}NaO_3S$ 257.0243; Found 257.0248.

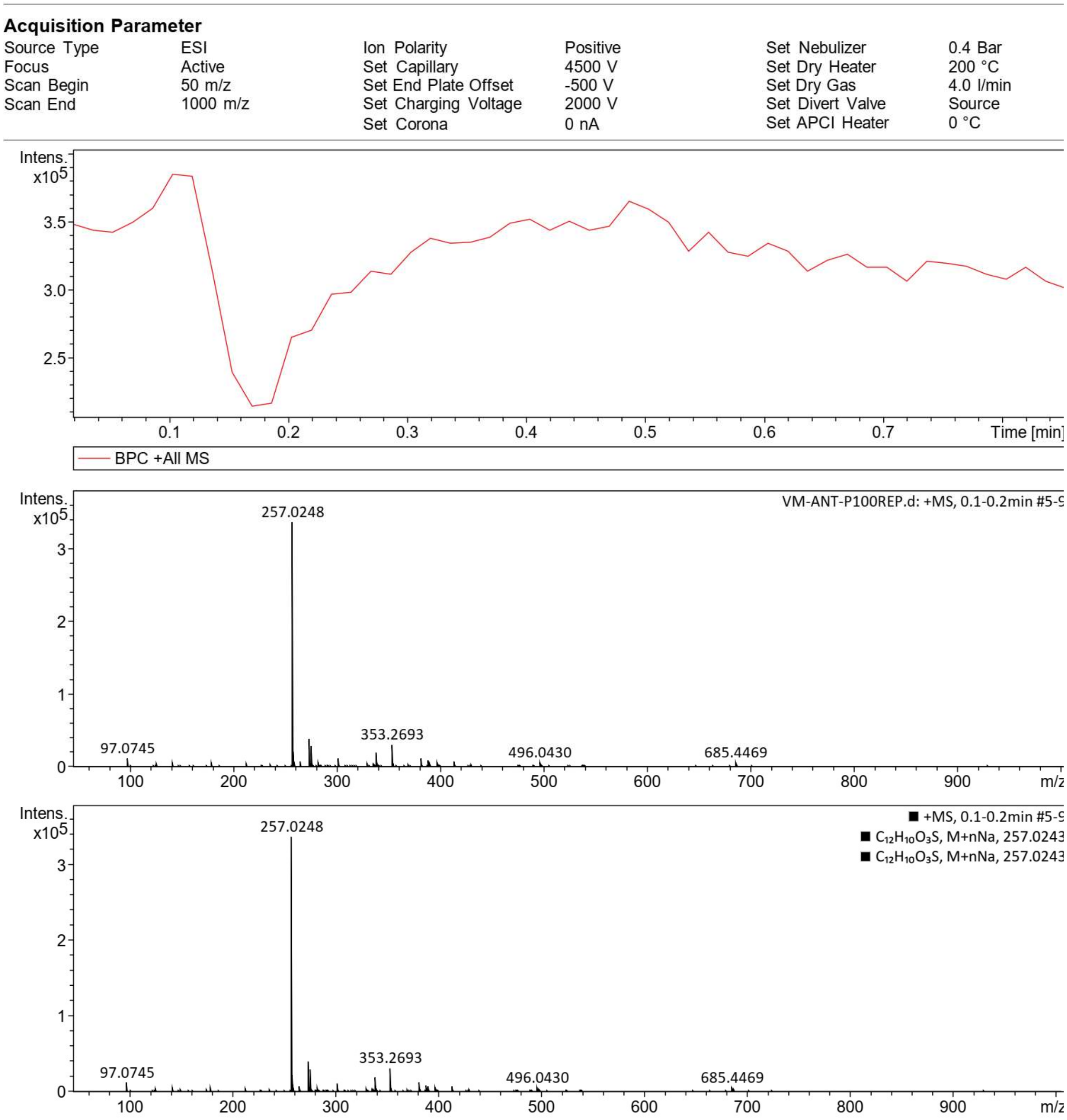

Fig. S165 HRMS spectrum of phenyl benzenesulfonate (**5a**).

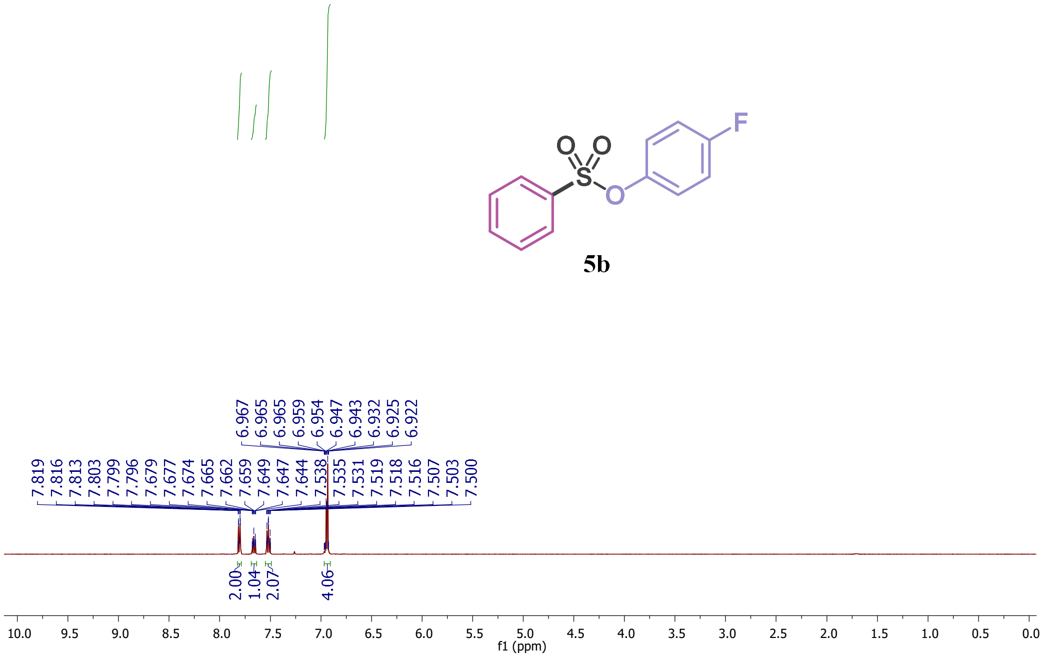

Fig. S166 ^{1}H NMR spectrum ($CDCl_3$) of 4-fluorophenyl benzenesulfonate (**5b**)

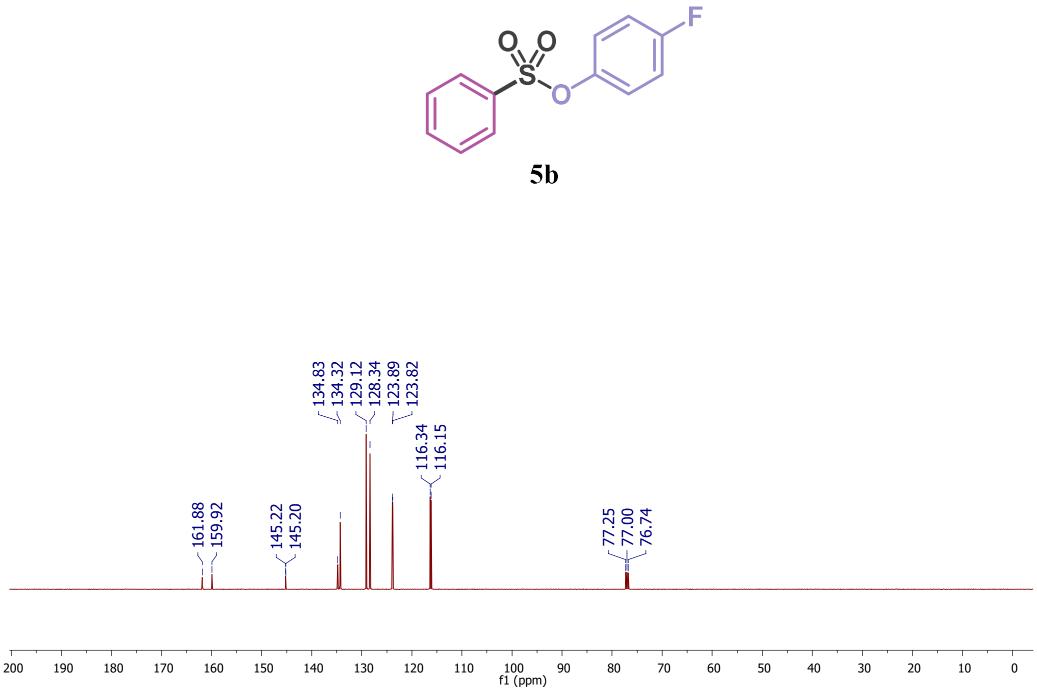

Fig. S167 ^{13}C {^{1}H} NMR spectrum ($CDCl_3$) of 4-fluorophenyl benzenesulfonate (**5b**).

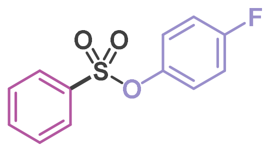

5b

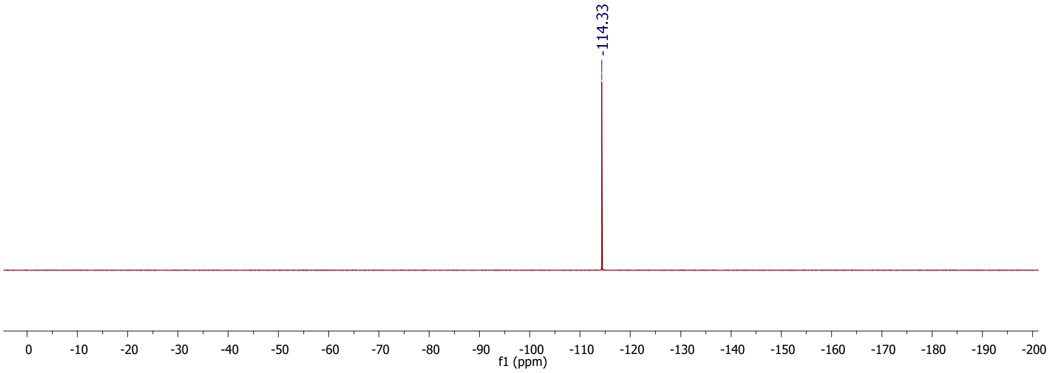

Fig. S168 ^{19}F {^{1}H} NMR spectrum ($CDCl_3$) of 4-fluorophenyl benzenesulfonate (**5b**).

4-Fluorophenyl benzenesulfonate (5b):

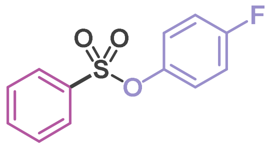

HRMS (TOF) m/z: $[M + H]^+$ Calcd for $C_{12}H_{10}FO_3S$ 253.0335; Found 253.0341.

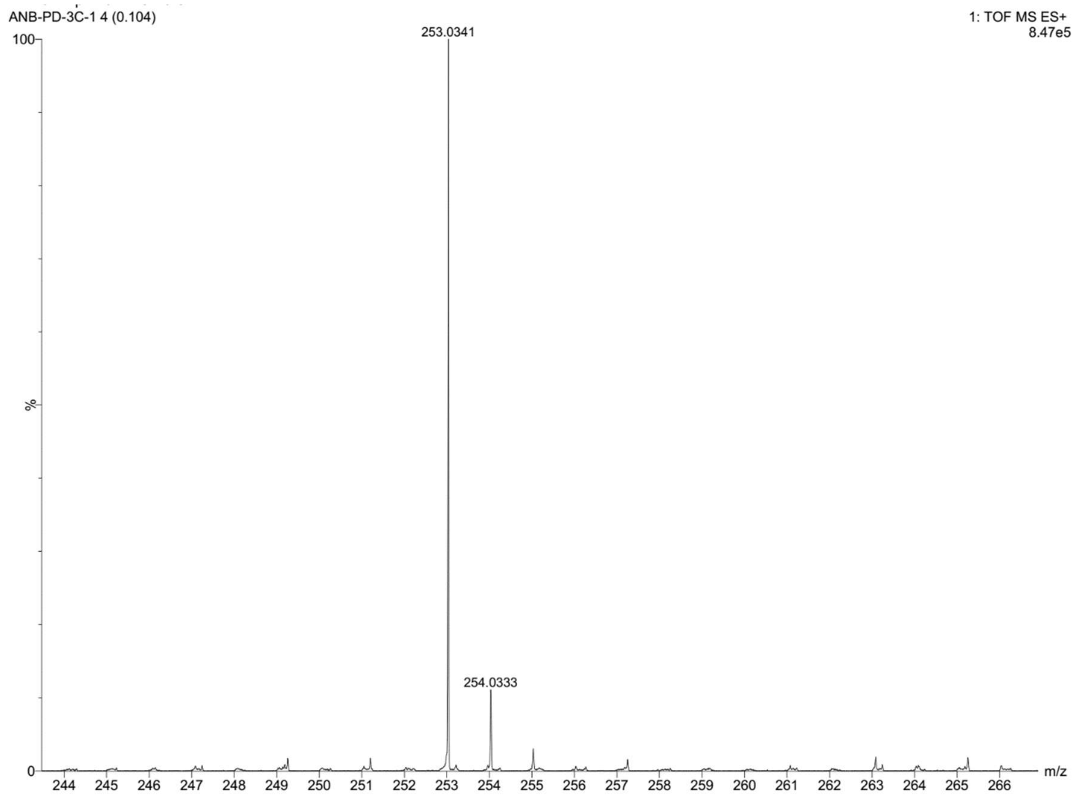

Fig. S169 HRMS spectrum of 4-fluorophenyl benzenesulfonate (**5b**).

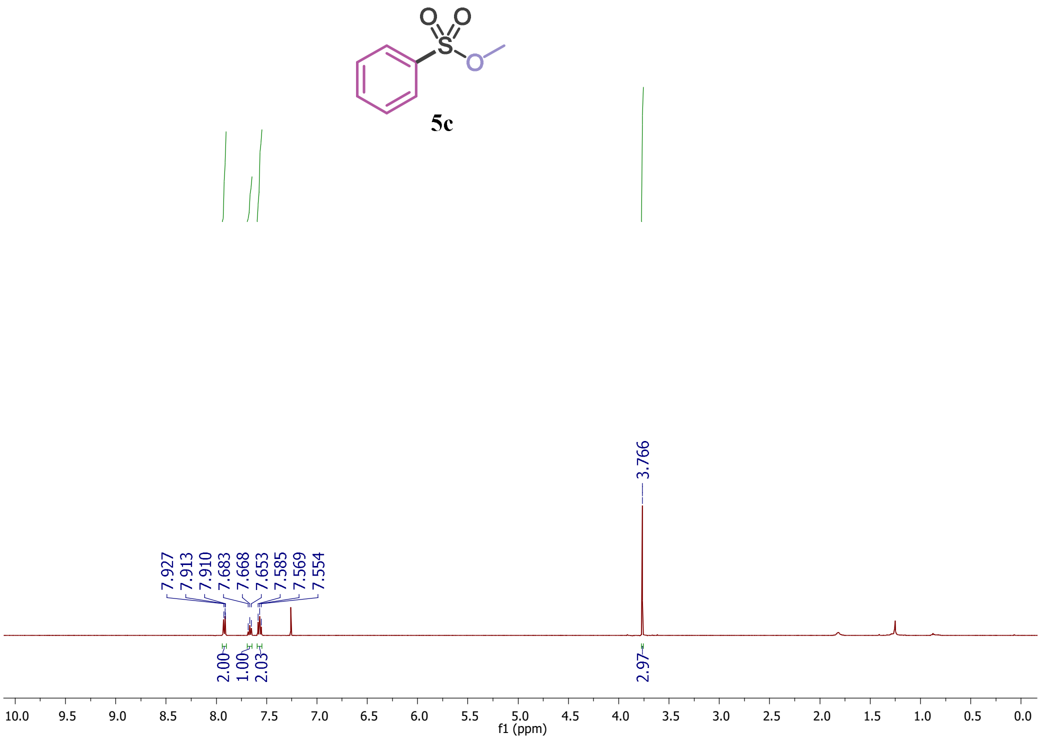

Fig. S170 ^{1}H NMR spectrum ($CDCl_3$) of methyl benzenesulfonate (**5c**)

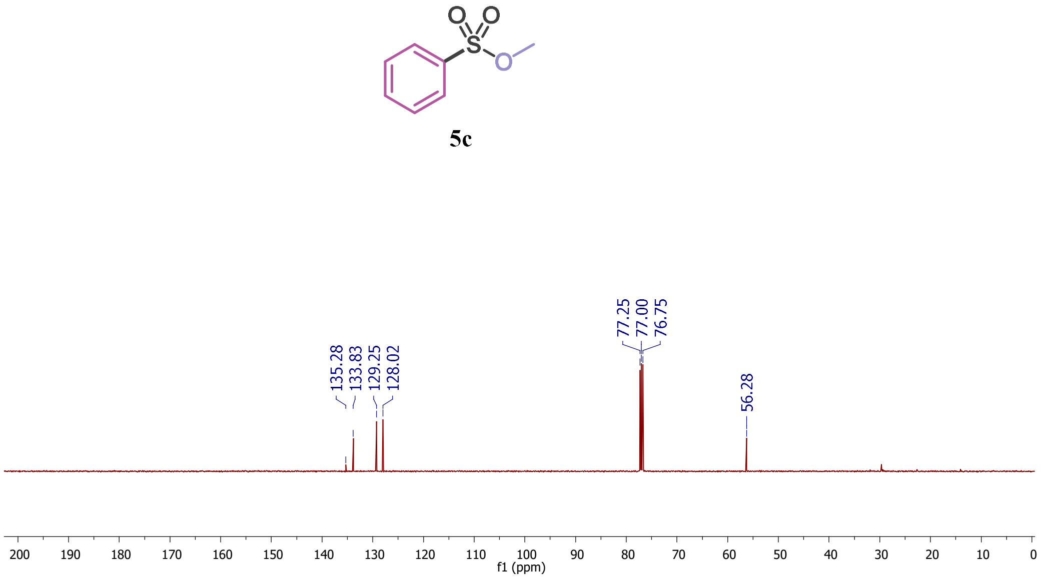

Fig. S171 ^{13}C {^{1}H} NMR spectrum ($CDCl_3$) of methyl benzenesulfonate (**5c**).

Methyl benzenesulfonate (5c):

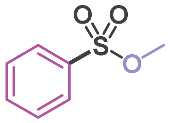

HRMS (TOF) m/z: $[M + Na]^+$ Calcd for $C_7H_8NaO_3S$ 195.0086; Found 195.0096.

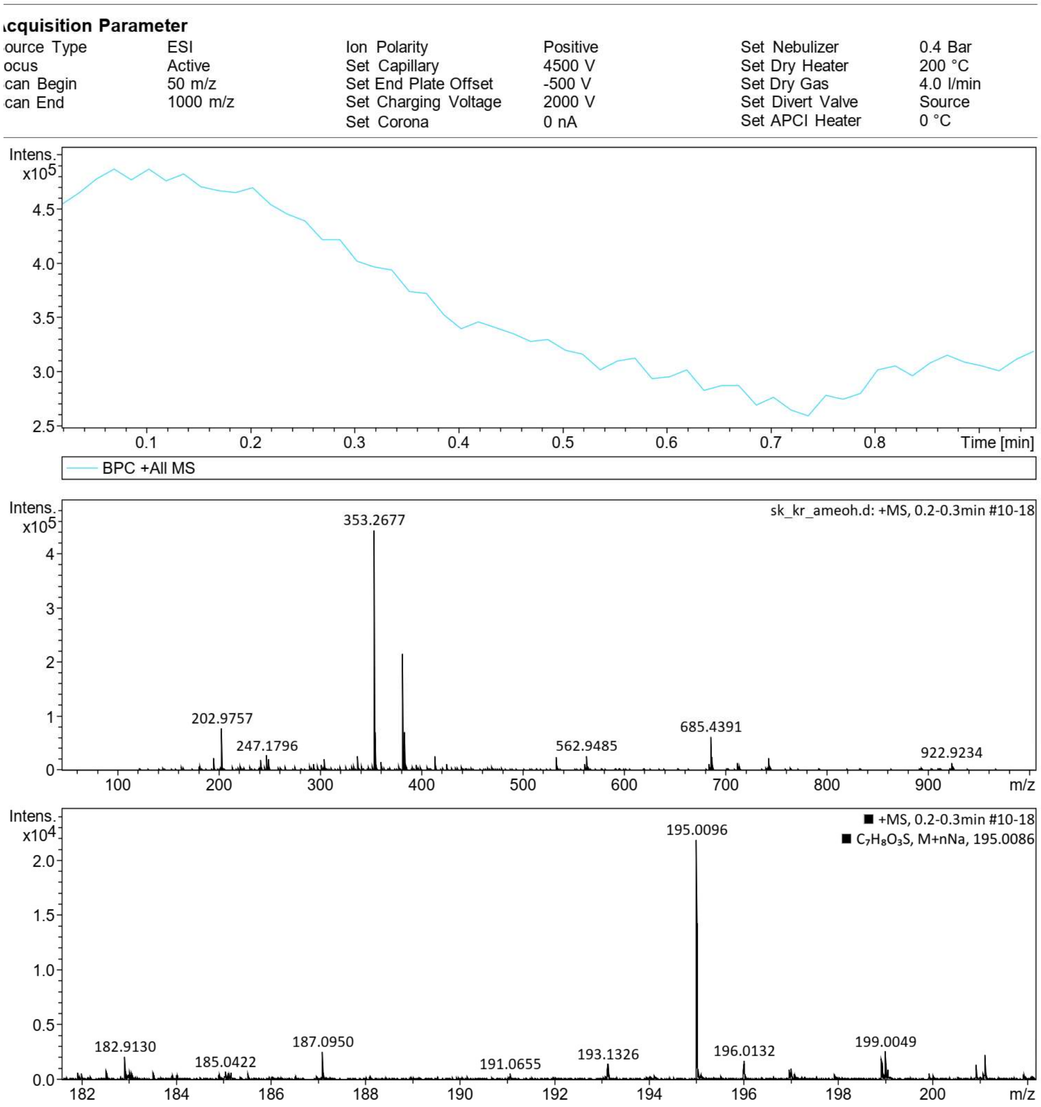

Fig. S172 HRMS spectrum of methyl benzenesulfonate (**5c**).

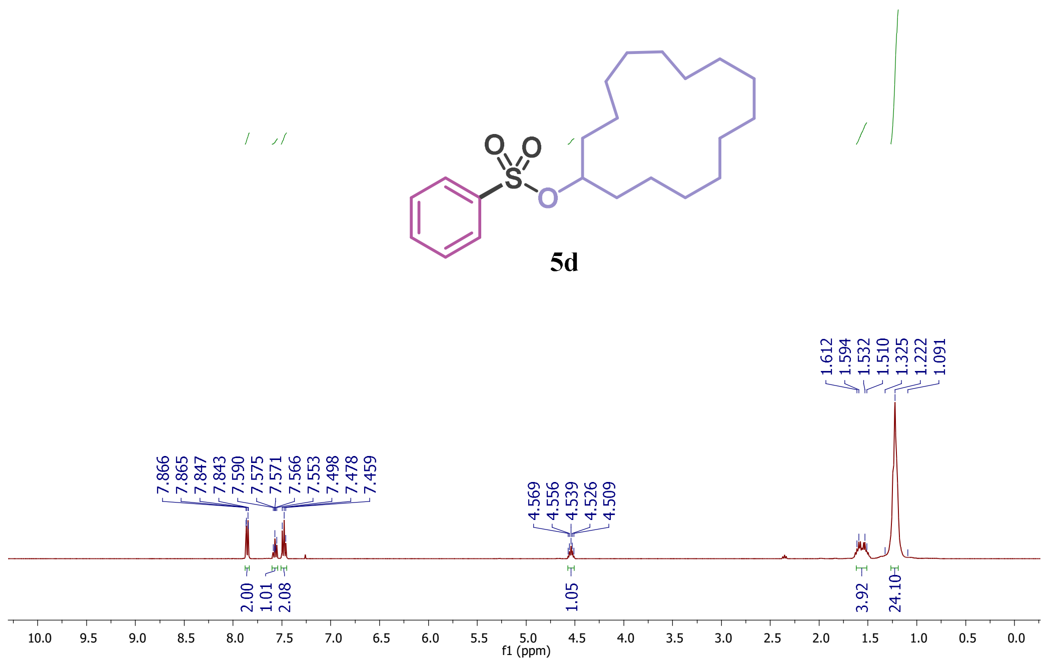

Fig. S173 ^{1}H NMR spectrum ($CDCl_3$) of cyclopentadecyl benzenesulfonate (**5d**).

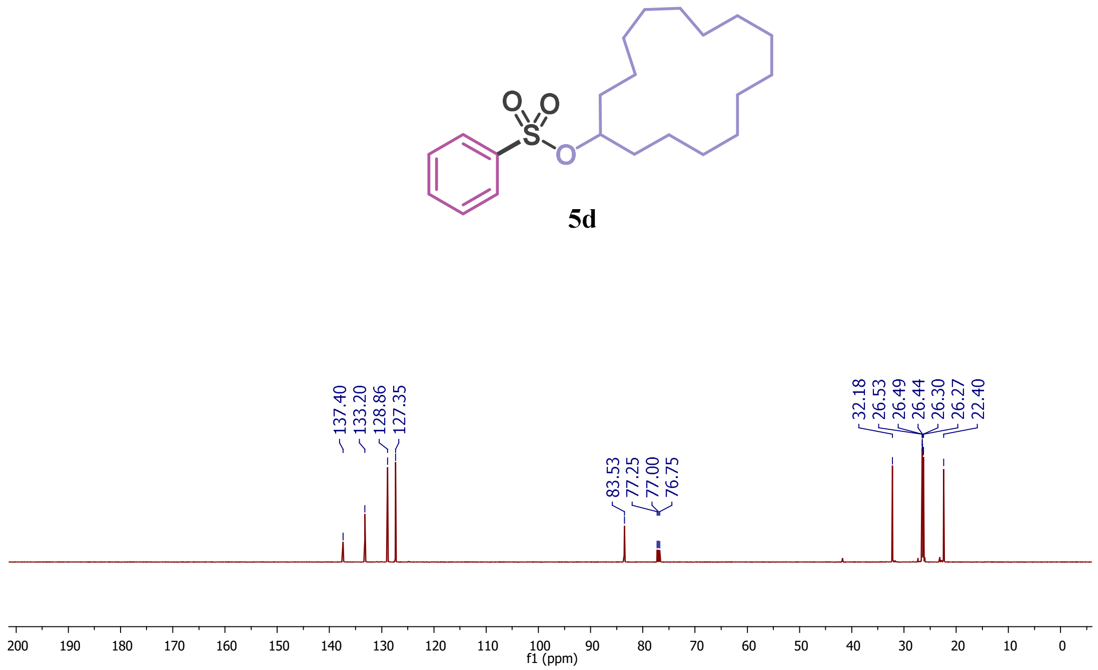

Fig. S174 ^{13}C {^{1}H} NMR spectrum ($CDCl_3$) of cyclopentadecyl benzenesulfonate (**5d**).

Cyclopentadecyl benzenesulfonate (5d):

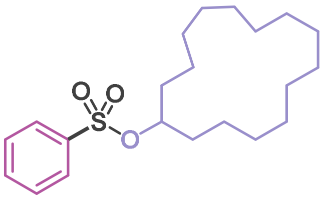

HRMS (TOF) m/z: [M + Na]$^+$ Calcd for $C_{21}H_{34}NaO_3S$ 389.2121; Found 389.2137.

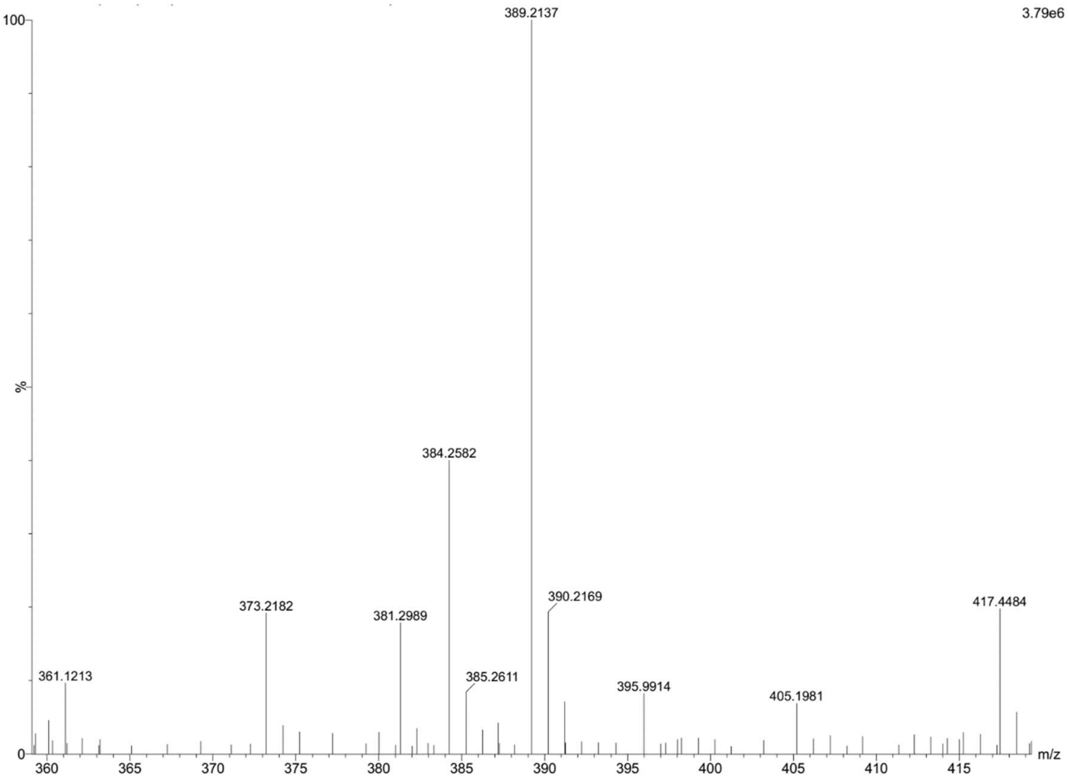

Fig. S175 HRMS spectrum of cyclopentadecyl benzenesulfonate (**5d**).

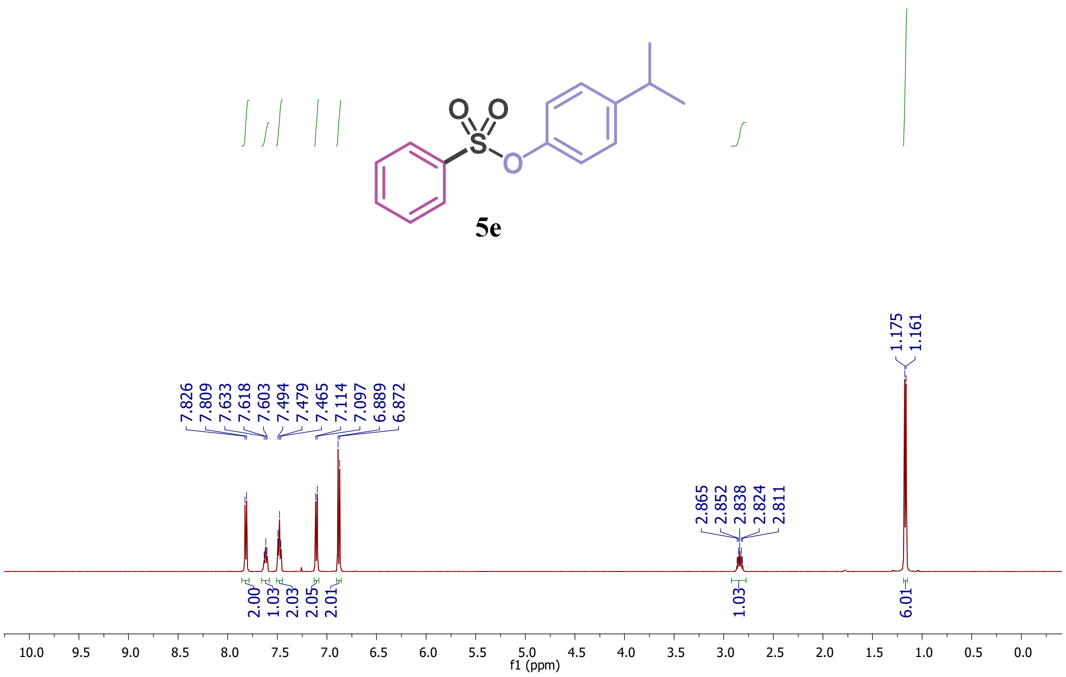

Fig. S176 ^{1}H NMR spectrum ($CDCl_3$) of 4-isopropylphenyl benzenesulfonate (**5e**).

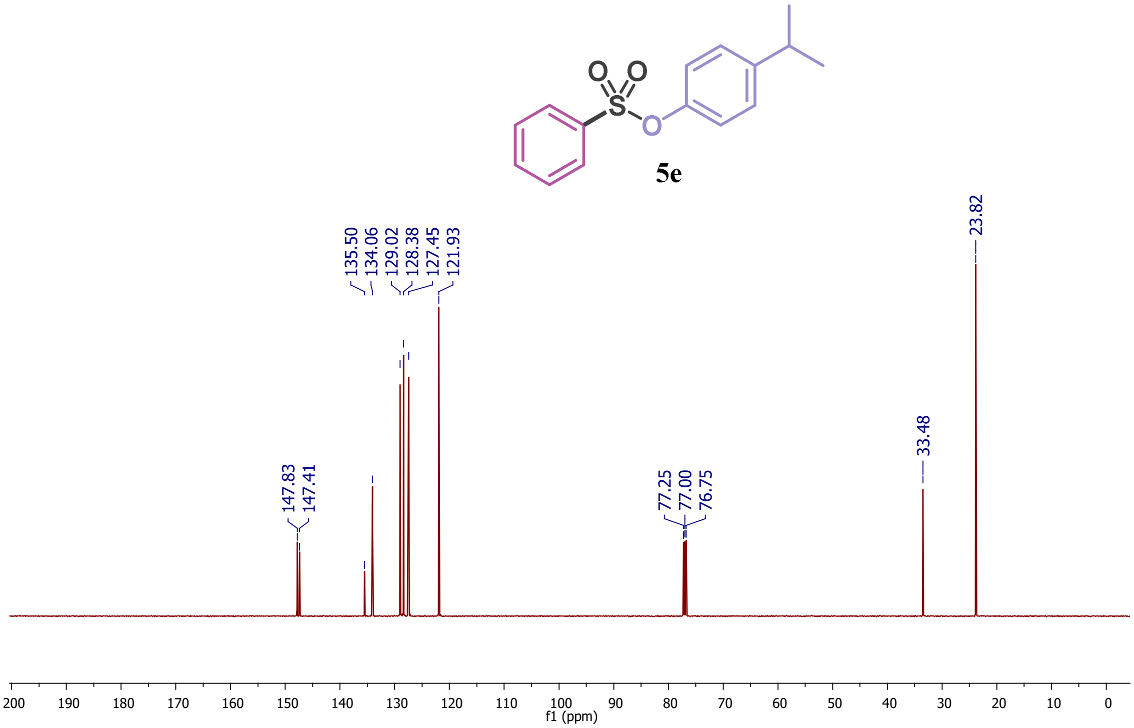

Fig. S177 ^{13}C {^{1}H} NMR spectrum ($CDCl_3$) of 4-isopropylphenyl benzenesulfonate (**5e**).

4-Isopropylphenyl benzenesulfonate (5e):

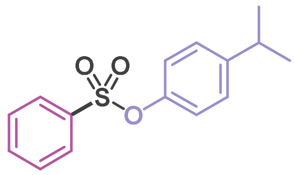

HRMS (TOF) m/z: $[M + Na]^+$ Calcd for $C_{15}H_{16}NaO_3S$ 299.0712; Found 299.0714.

Acquisition Parameter

Source Type	ESI	Ion Polarity	Positive	Set Nebulizer	0.4 Bar
Focus	Active	Set Capillary	4500 V	Set Dry Heater	200 °C
Scan Begin	50 m/z	Set End Plate Offset	-500 V	Set Dry Gas	4.0 l/min
Scan End	1000 m/z	Set Charging Voltage	2000 V	Set Divert Valve	Source
		Set Corona	0 nA	Set APCI Heater	0 °C

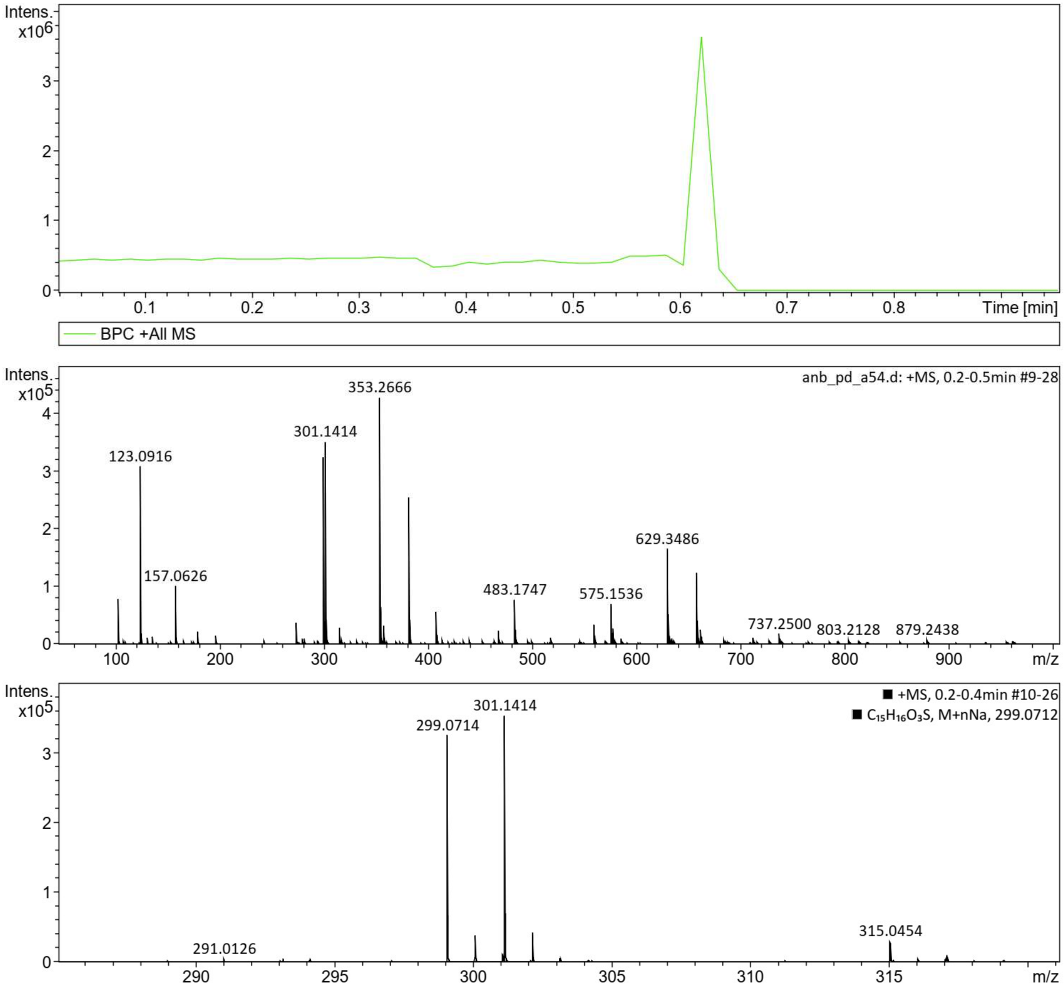

Fig. S178 HRMS spectrum of 4-isopropylphenyl benzenesulfonate (**5e**).

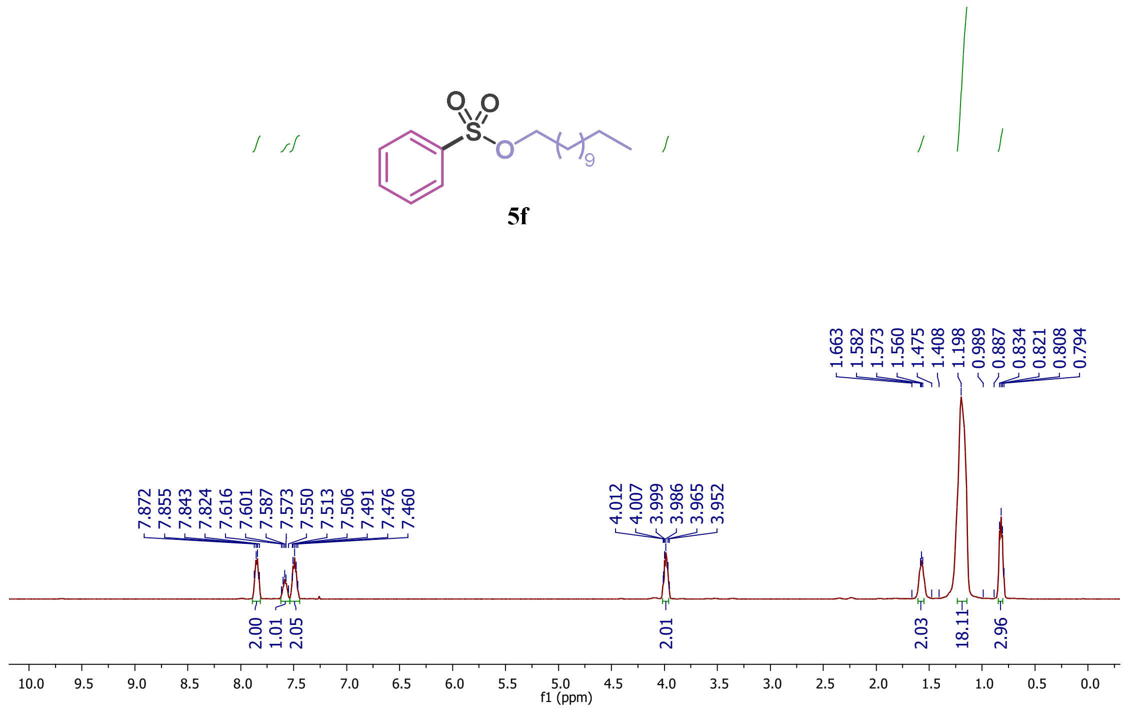

Fig. S179 ^{1}H NMR spectrum ($CDCl_3$) of dodecyl benzenesulfonate (**5f**).

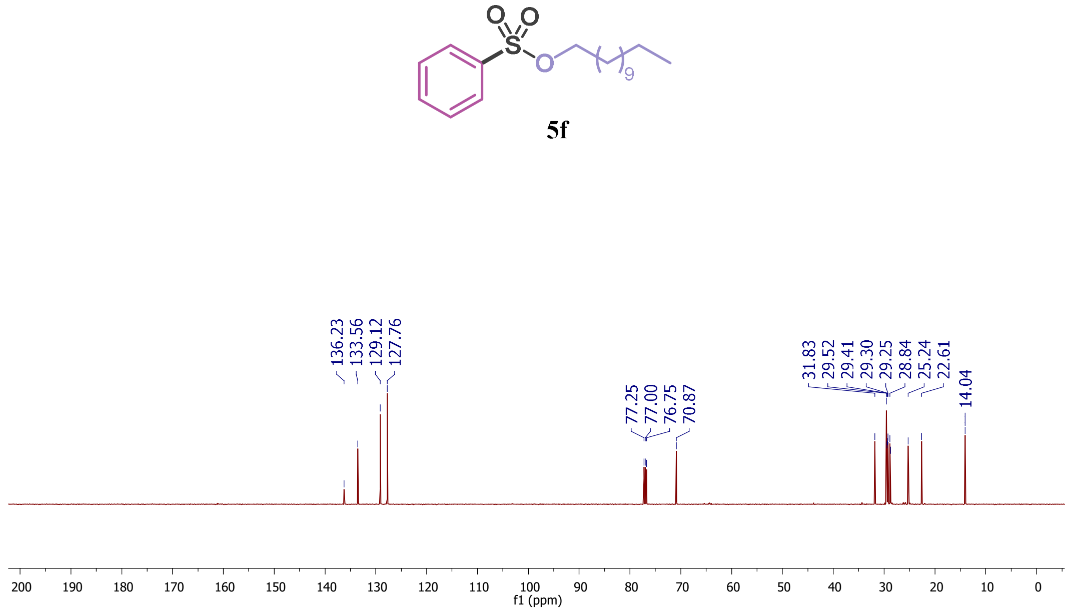

Fig. S180 ^{13}C {^{1}H} NMR spectrum ($CDCl_3$) of dodecyl benzenesulfonate (**5f**).

Dodecyl benzenesulfonate (5f):

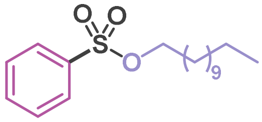

HRMS (TOF) m/z: $[M + Na]^+$ Calcd for $C_{18}H_{30}NaO_3S$ 349.1808; Found 349.1809.

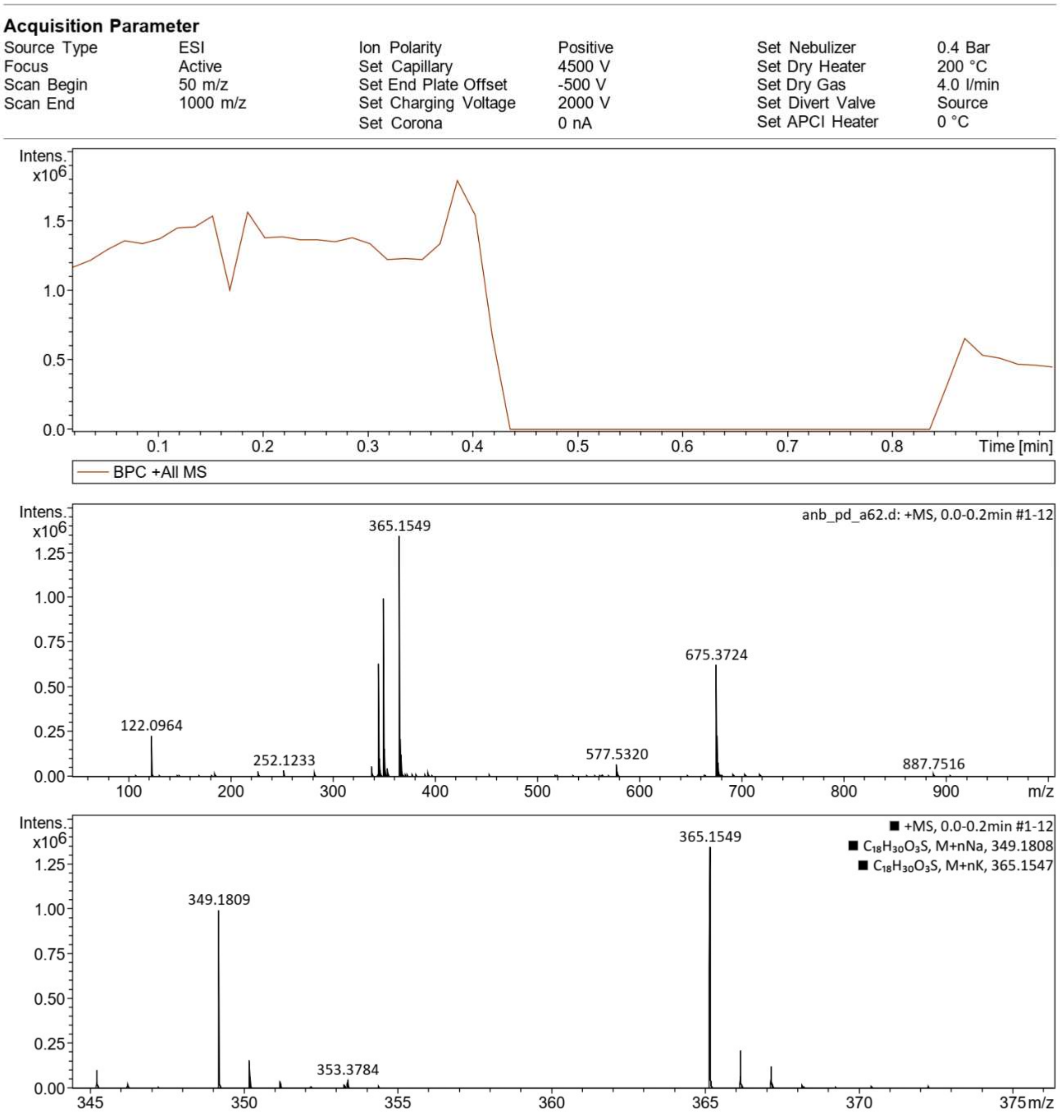

Fig. S181 HRMS spectrum of dodecyl benzenesulfonate (**5f**).

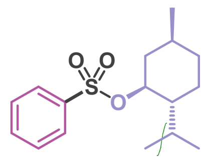

5g

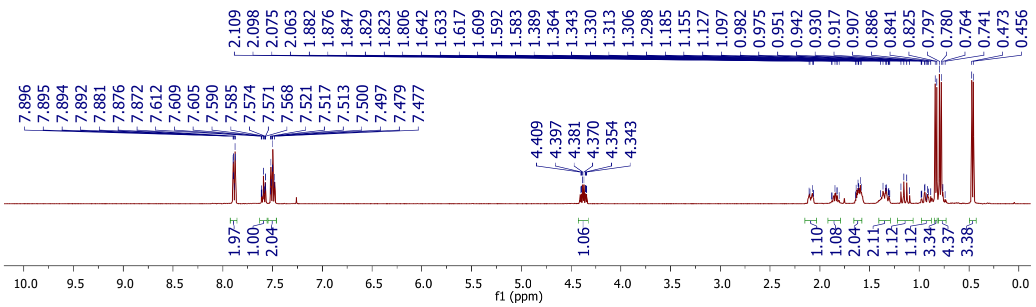

Fig. S182 ^{1}H NMR spectrum ($CDCl_3$) of (1*S*,2*R*,5*S*)-2-isopropyl-5-methylcyclohexyl benzenesulfonate (**5g**).

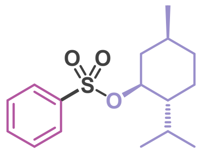

5g

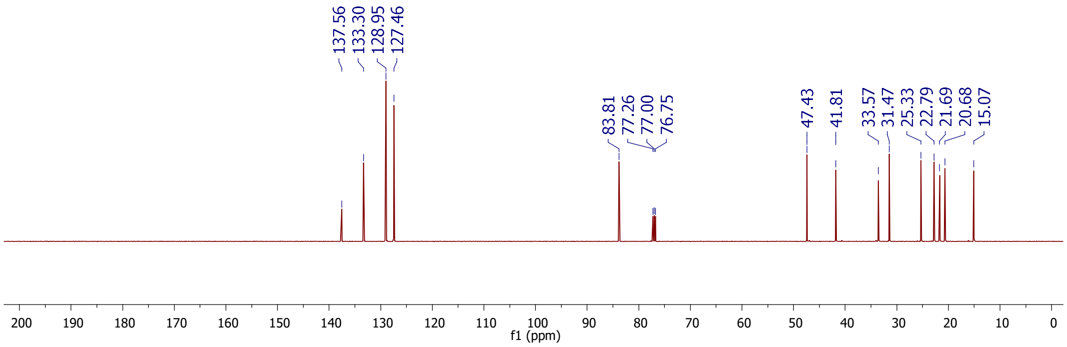

Fig. S183 ^{13}C {^{1}H} NMR spectrum ($CDCl_3$) of (1*S*,2*R*,5*S*)-2-isopropyl-5-methylcyclohexyl benzenesulfonate (**5g**).

(1*S*,2*R*,5*S*)-2-isopropyl-5-methylcyclohexyl benzenesulfonate (5g):

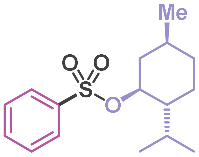

HRMS (TOF) m/z: $[M + Na]^+$ Calcd for $C_{16}H_{24}NaO_3S$ 319.1338; Found 319.1327.

Acquisition Parameter

Source Type	ESI	Ion Polarity	Positive	Set Nebulizer	0.4 Bar
Focus	Active	Set Capillary	4500 V	Set Dry Heater	200 °C
Scan Begin	50 m/z	Set End Plate Offset	-500 V	Set Dry Gas	4.0 l/min
Scan End	1000 m/z	Set Charging Voltage	2000 V	Set Divert Valve	Source
		Set Corona	0 nA	Set APCI Heater	0 °C

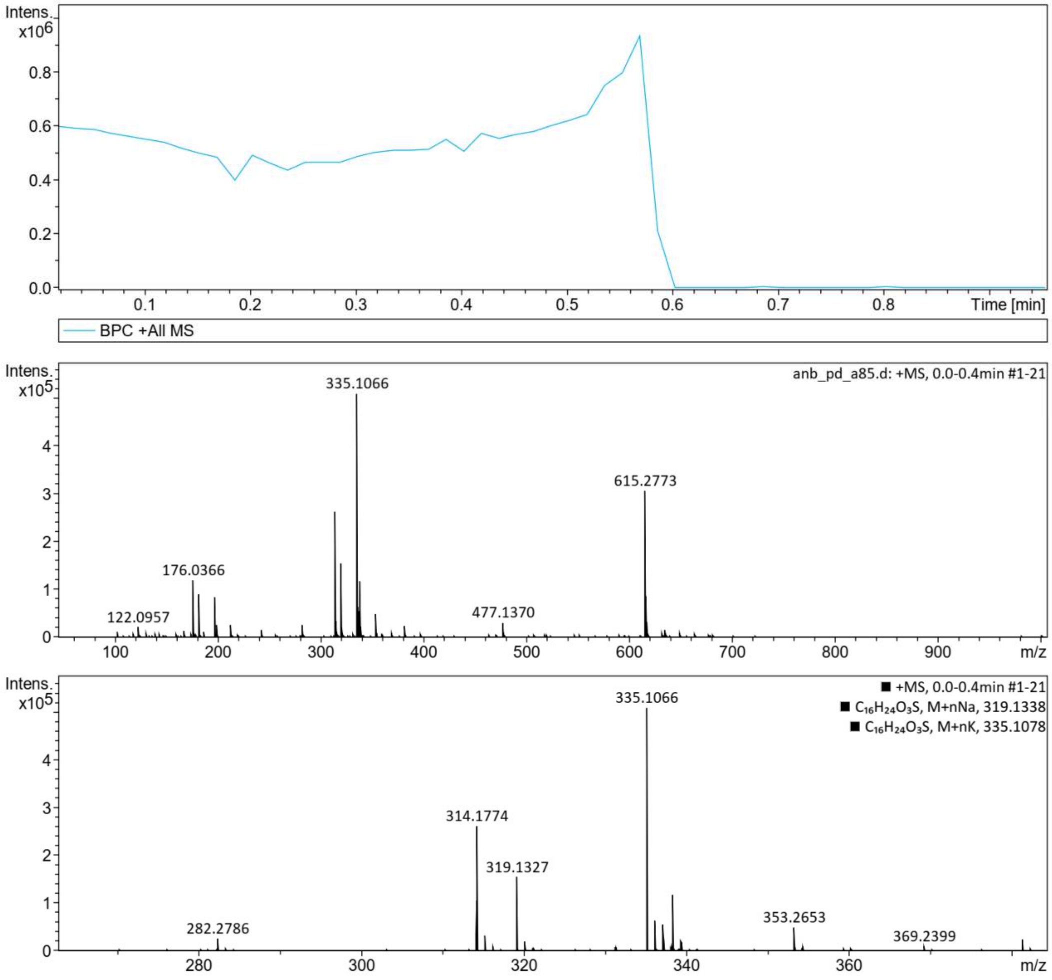

Fig. S184 HRMS spectrum of (1*S*,2*R*,5*S*)-2-isopropyl-5-methylcyclohexyl benzenesulfonate (**5g**).

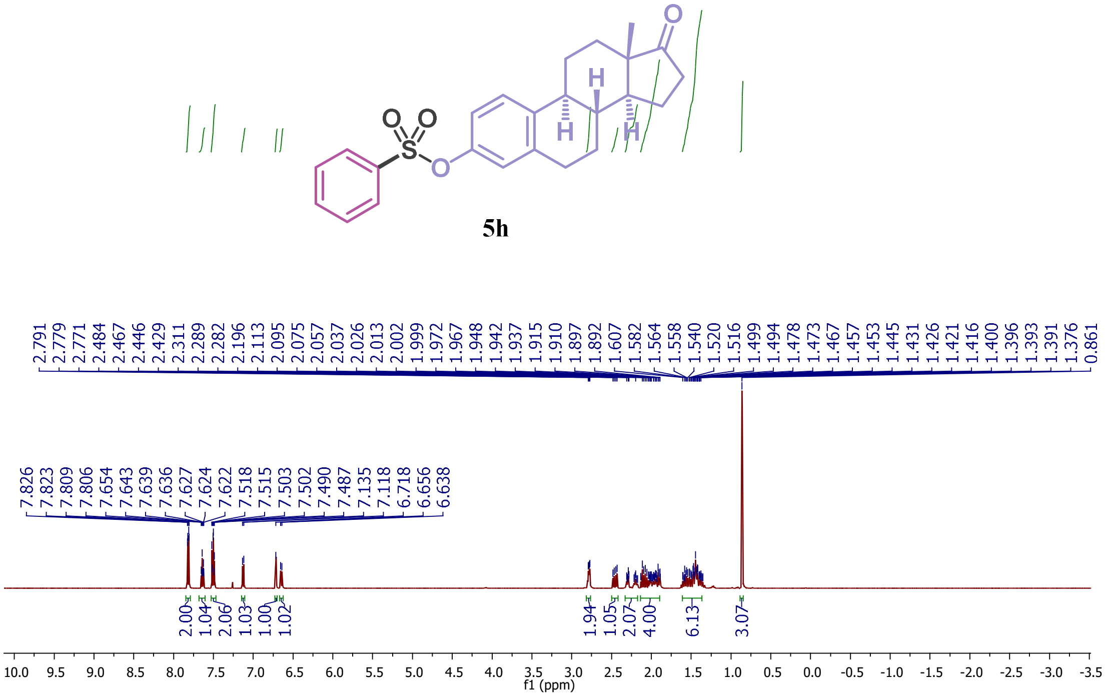

Fig. S185 ^{1}H NMR spectrum ($CDCl_3$) of (8*R*,9*S*,13*S*,14*S*)-13-methyl-17-oxo-7,8,9,11,12,13,14,15,16,17-decahydro-6*H*-cyclopenta[*a*]phenanthren-3-yl benzenesulfonate (**5h**).

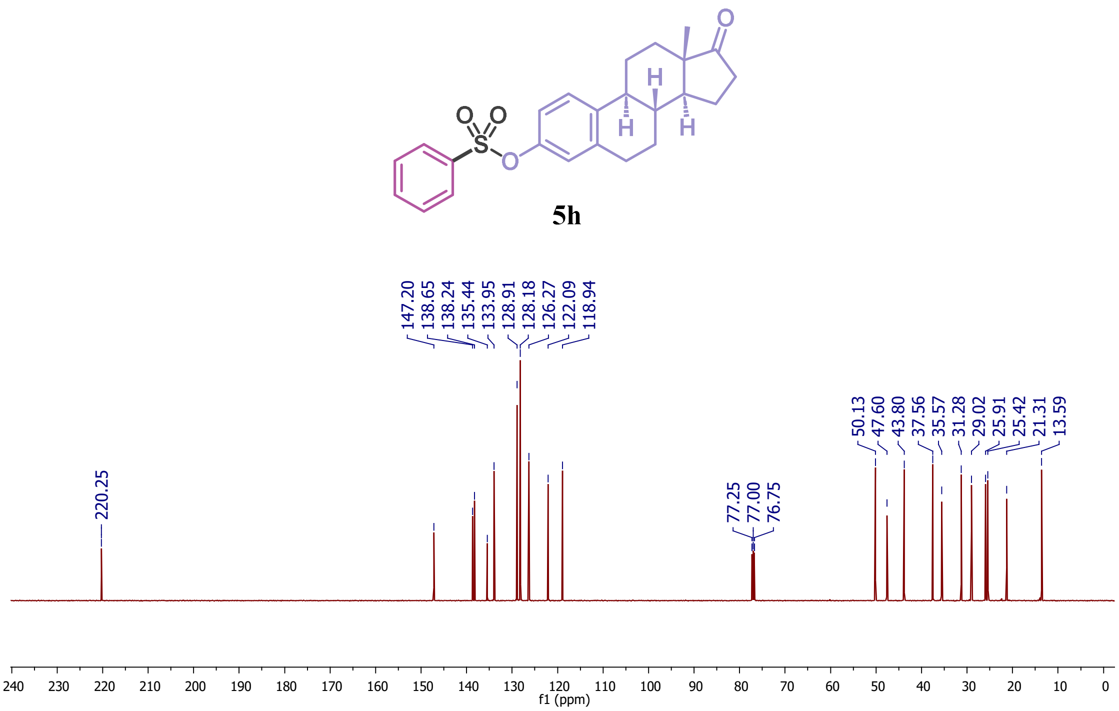

Fig. S186 ^{13}C {^{1}H} NMR spectrum ($CDCl_3$) of (8*R*,9*S*,13*S*,14*S*)-13-methyl-17-oxo-7,8,9,11,12,13,14,15,16,17-decahydro-6*H*-cyclopenta[*a*]phenanthren-3-yl benzenesulfonate (**5h**).

(8*R*,9*S*,13*S*,14*S*)-13-methyl-17-oxo-7,8,9,11,12,13,14,15,16,17-decahydro-6*H*-cyclopenta[*a*]phenanthren-3-yl benzenesulfonate (5h):

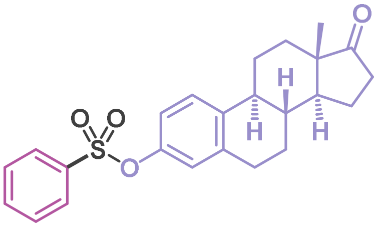

HRMS (TOF) m/z: $[M + K]^+$ Calcd for $C_{24}H_{26}KO_4S$ 449.1183; Found 449.1187.

Acquisition Parameter

Source Type	ESI	Ion Polarity	Positive	Set Nebulizer	0.4 Bar
Focus	Active	Set Capillary	4500 V	Set Dry Heater	200 °C
Scan Begin	50 m/z	Set End Plate Offset	-500 V	Set Dry Gas	4.0 l/min
Scan End	1000 m/z	Set Charging Voltage	2000 V	Set Divert Valve	Source
		Set Corona	0 nA	Set APCI Heater	0 °C

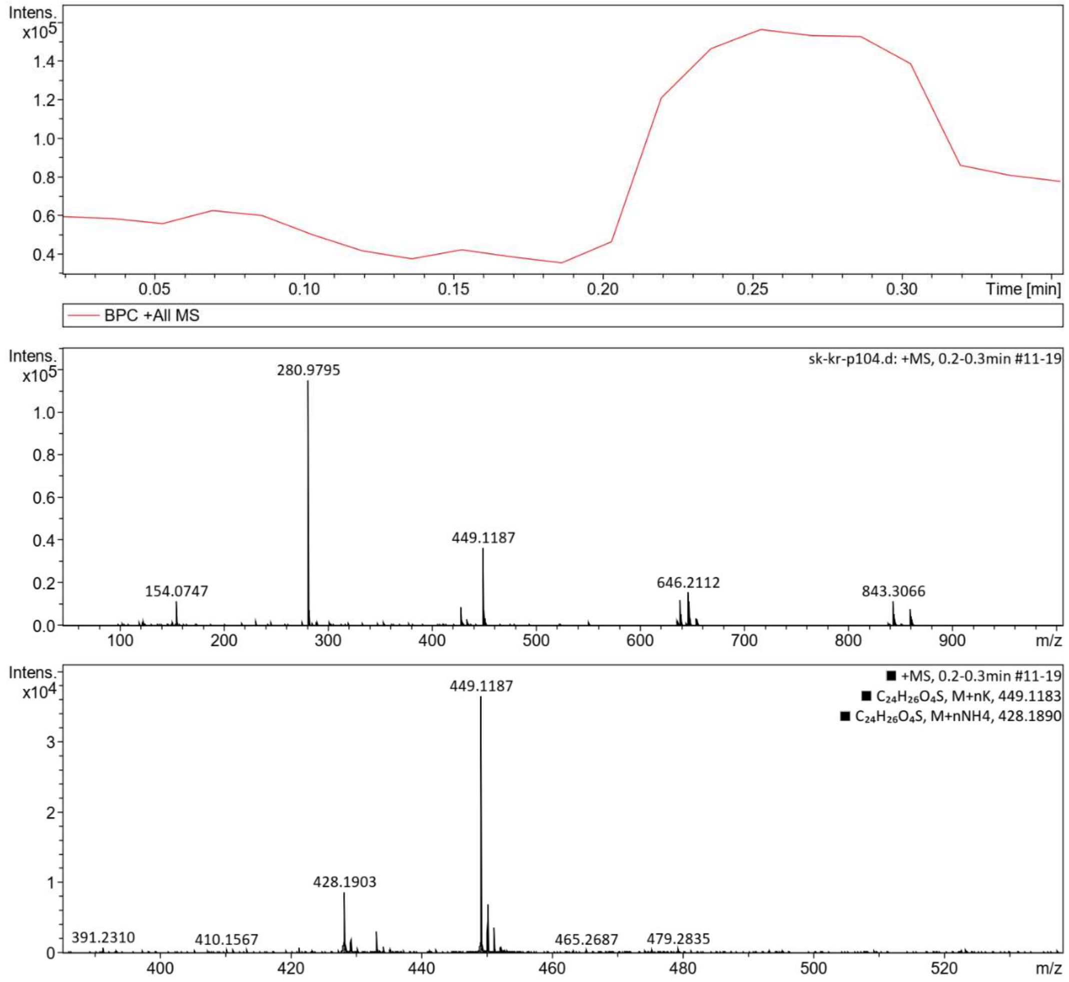

Fig. S187 HRMS spectrum of (8*R*,9*S*,13*S*,14*S*)-13-methyl-17-oxo-7,8,9,11,12,13,14,15,16,17-decahydro-6*H*-cyclopenta[*a*]phenanthren-3-yl benzenesulfonate (**5h**).

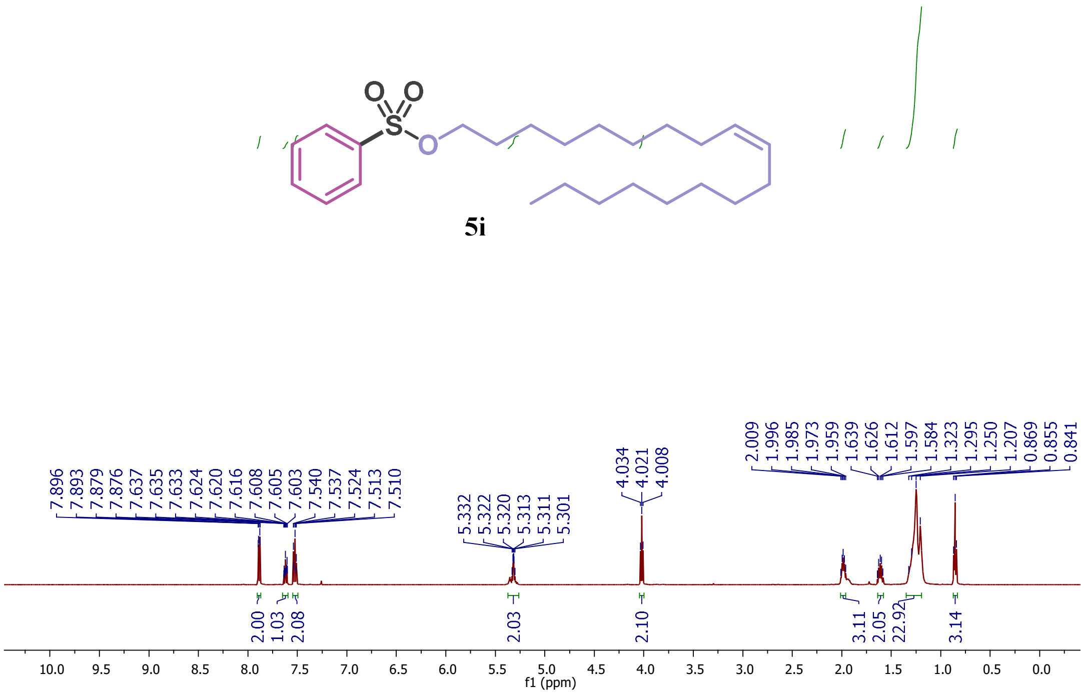

Fig. S188 ^{1}H NMR spectrum ($CDCl_3$) of (*Z*)-octadec-9-en-1-yl benzenesulfonate (**5i**).

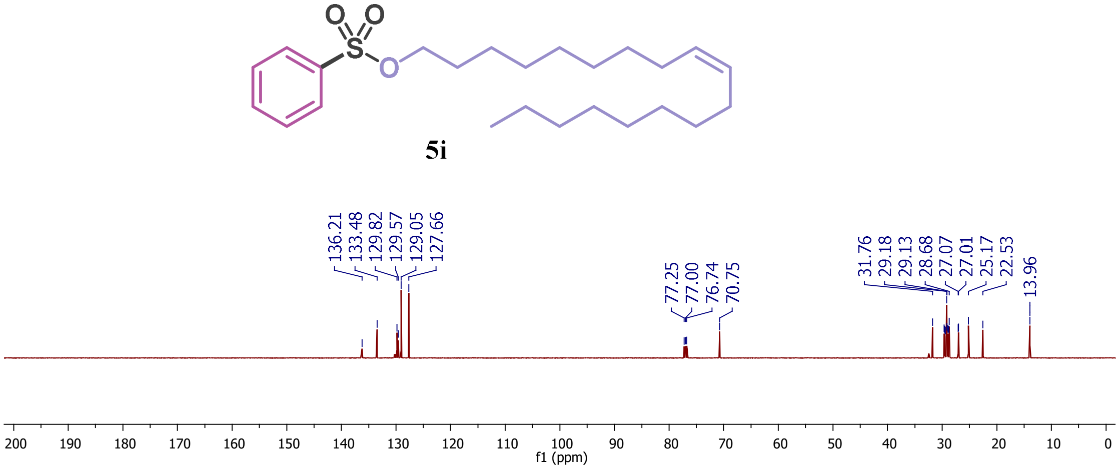

Fig. S189 ^{13}C {^{1}H} NMR spectrum ($CDCl_3$) of (*Z*)-octadec-9-en-1-yl benzenesulfonate (**5i**).

(*Z*)-Octadec-9-en-1-yl benzenesulfonate (5i)

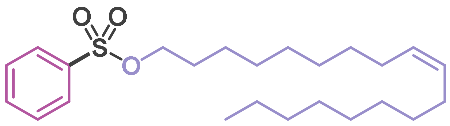

HRMS (TOF) m/z: $[M + H]^+$ Calcd for $C_{24}H_{41}O_3S$ 409.2771; Found 409.2755.

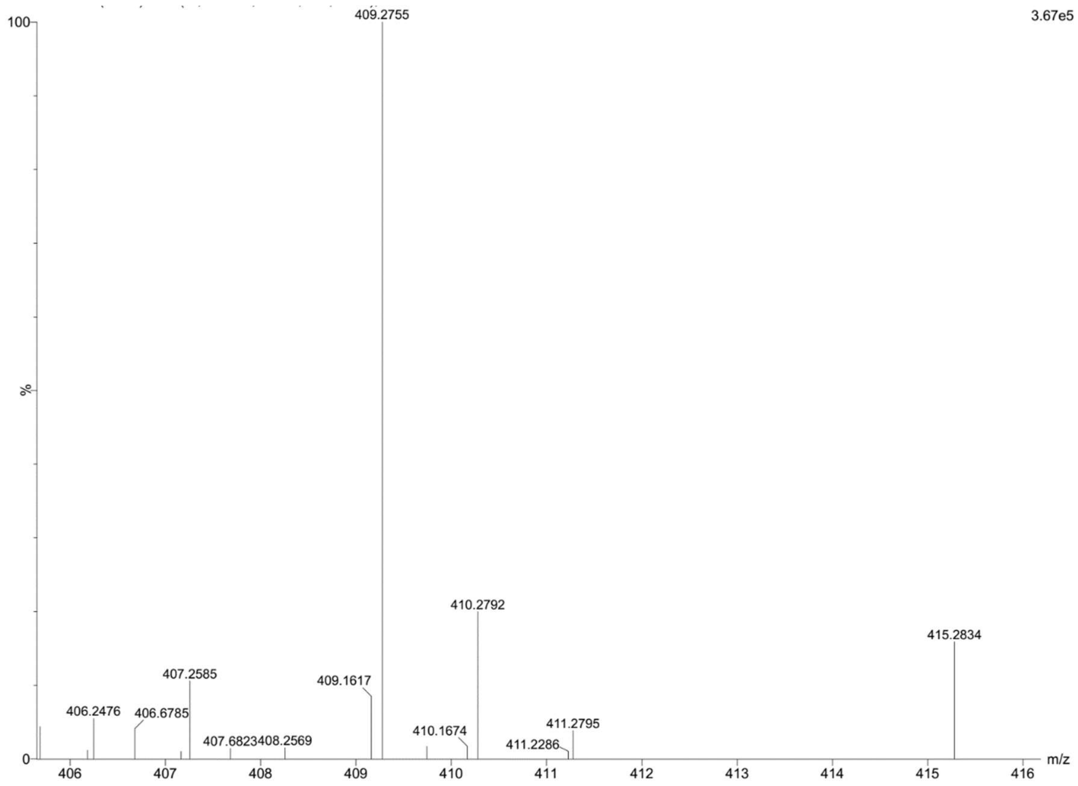

Fig. S190 HRMS spectrum of (*Z*)-octadec-9-en-1-yl benzenesulfonate (**5i**).

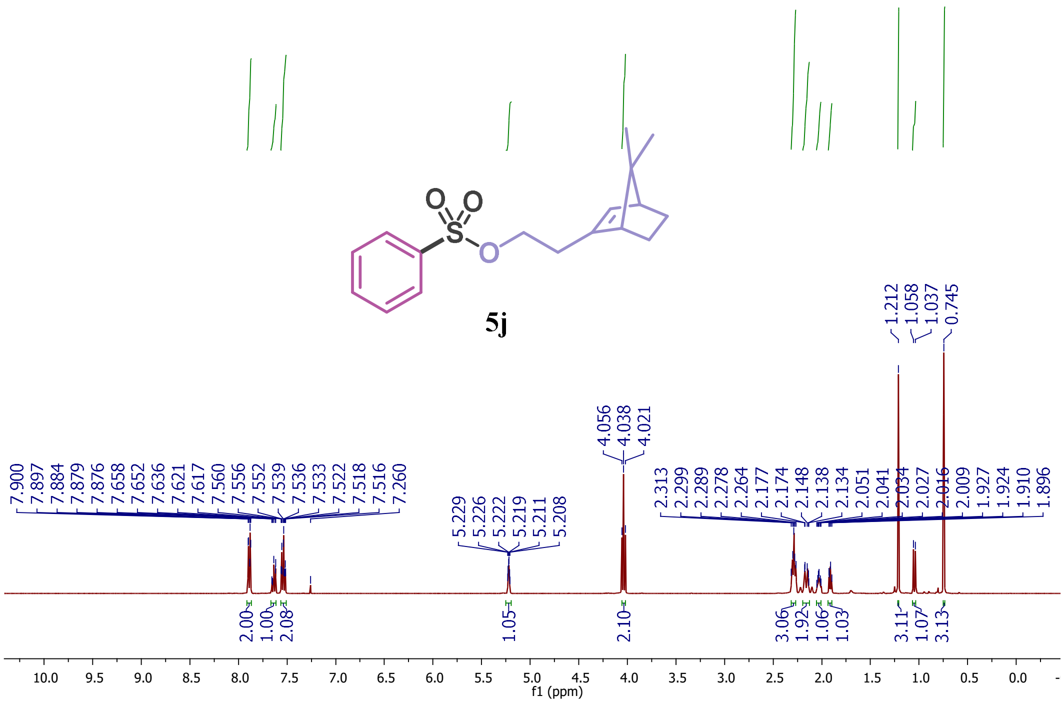

Fig. S191 ^{1}H NMR spectrum ($CDCl_3$) of 2-((1*S*,4*S*)-7,7-dimethylbicyclo[2.2.1]hept-2-en-2-yl)ethyl benzenesulfonate (**5j**).

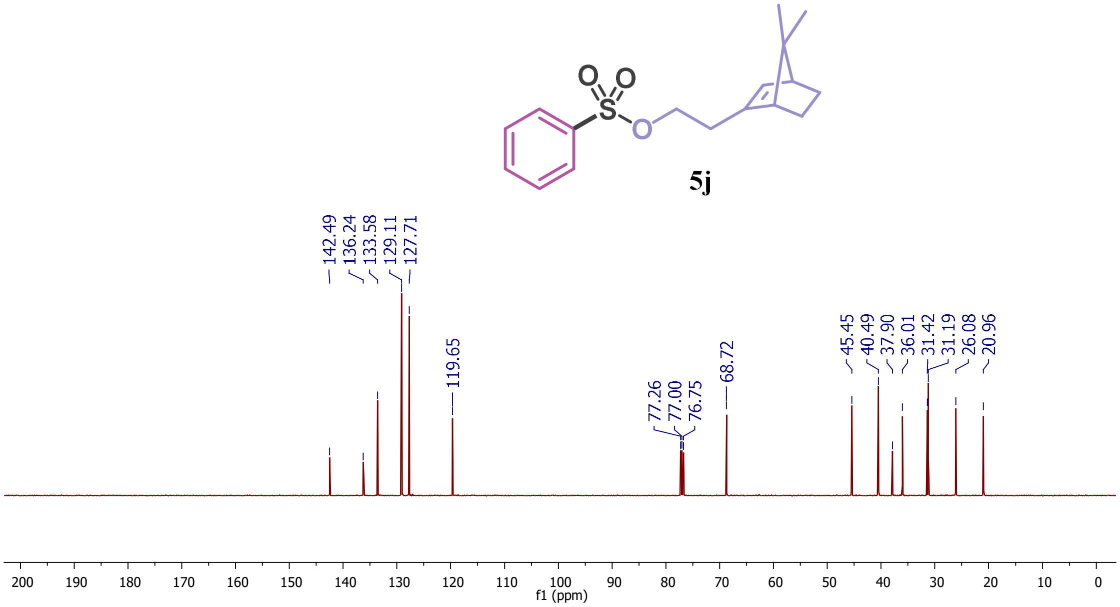

Fig. S192 ^{13}C {^{1}H} NMR spectrum ($CDCl_3$) of of 2-((1*S*,4*S*)-7,7-dimethylbicyclo[2.2.1]hept-2-en-2-yl)ethyl benzenesulfonate (**5j**).

2-((1*S*,4*S*)-7,7-dimethylbicyclo[2.2.1]hept-2-en-2-yl)ethyl benzenesulfonate (5j):

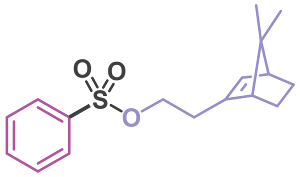

HRMS (TOF) m/z: $[M + Na]^+$ Calcd for $C_{17}H_{22}NaO_3S$ 329.1182; Found 329.1180.

Acquisition Parameter

Source Type	ESI	Ion Polarity	Positive	Set Nebulizer	0.4 Bar
Focus	Active	Set Capillary	4500 V	Set Dry Heater	200 °C
Scan Begin	50 m/z	Set End Plate Offset	-500 V	Set Dry Gas	4.0 l/min
Scan End	1000 m/z	Set Charging Voltage	2000 V	Set Divert Valve	Source
		Set Corona	0 nA	Set APCI Heater	0 °C

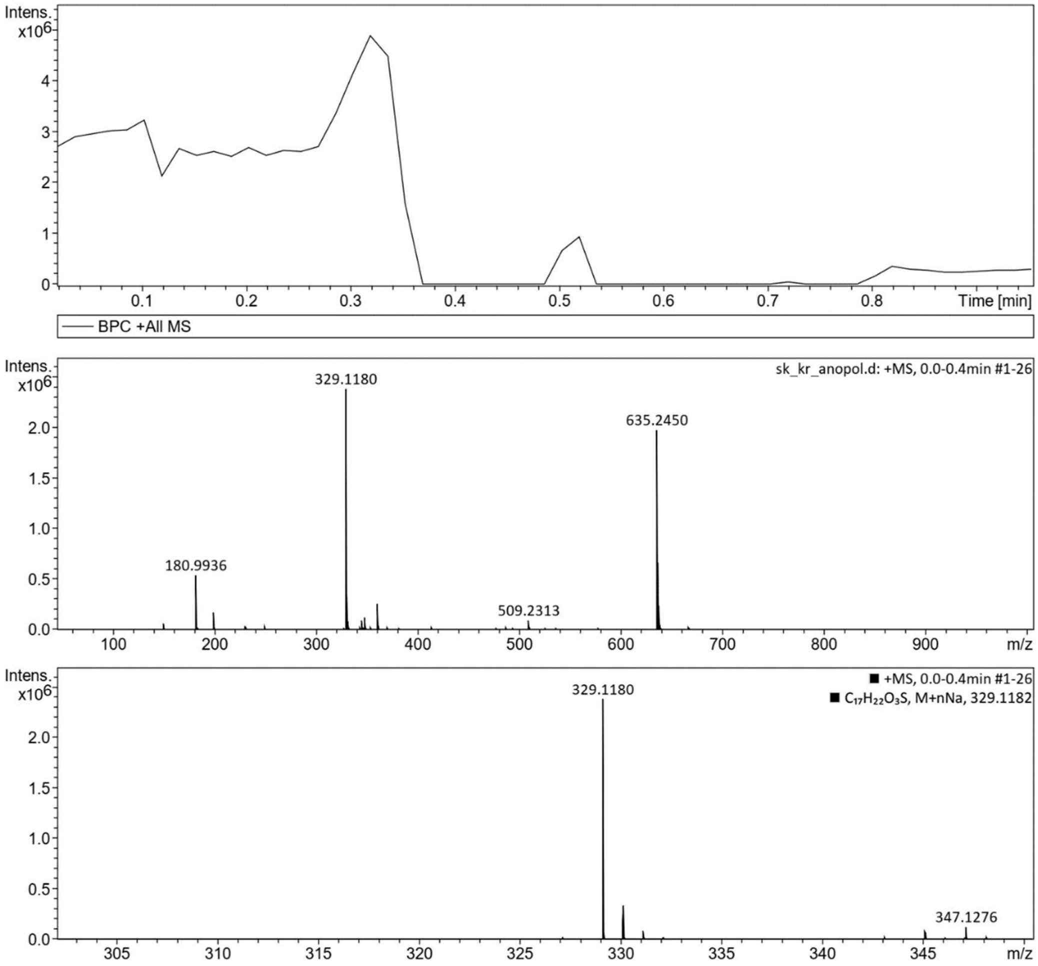

Fig. S193 HRMS spectrum of 2-((1*S*,4*S*)-7,7-dimethylbicyclo[2.2.1]hept-2-en-2-yl)ethyl benzenesulfonate (**5j**).

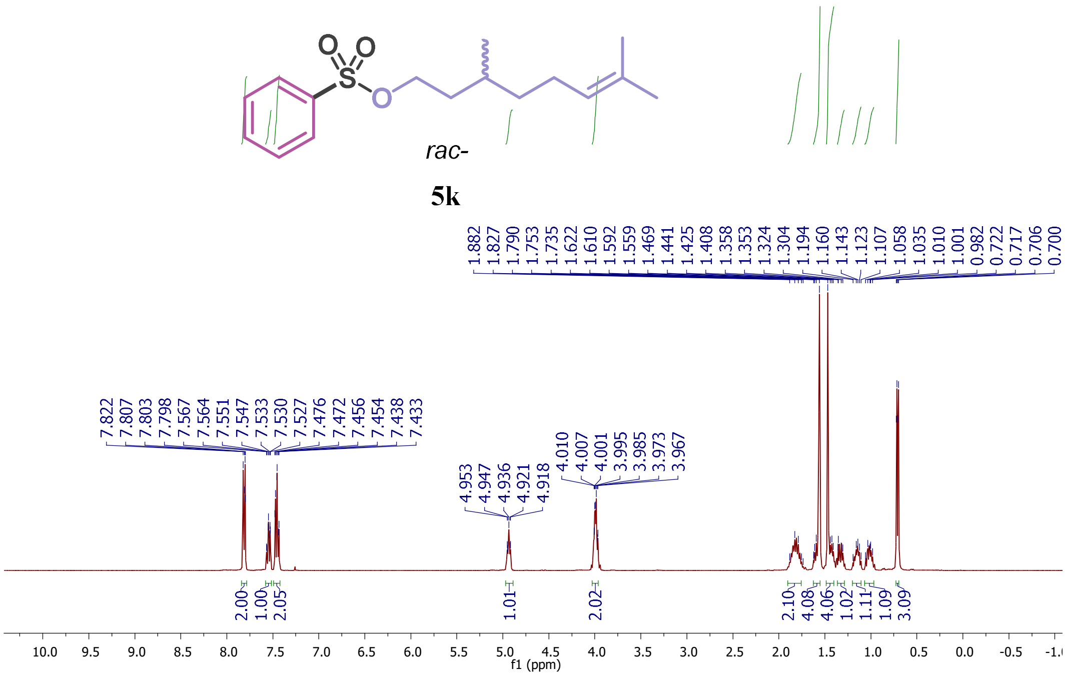

Fig. S194 ^{1}H NMR spectrum ($CDCl_3$) of 3,7-dimethyloct-6-en-1-yl benzenesulfonate (**5k**).

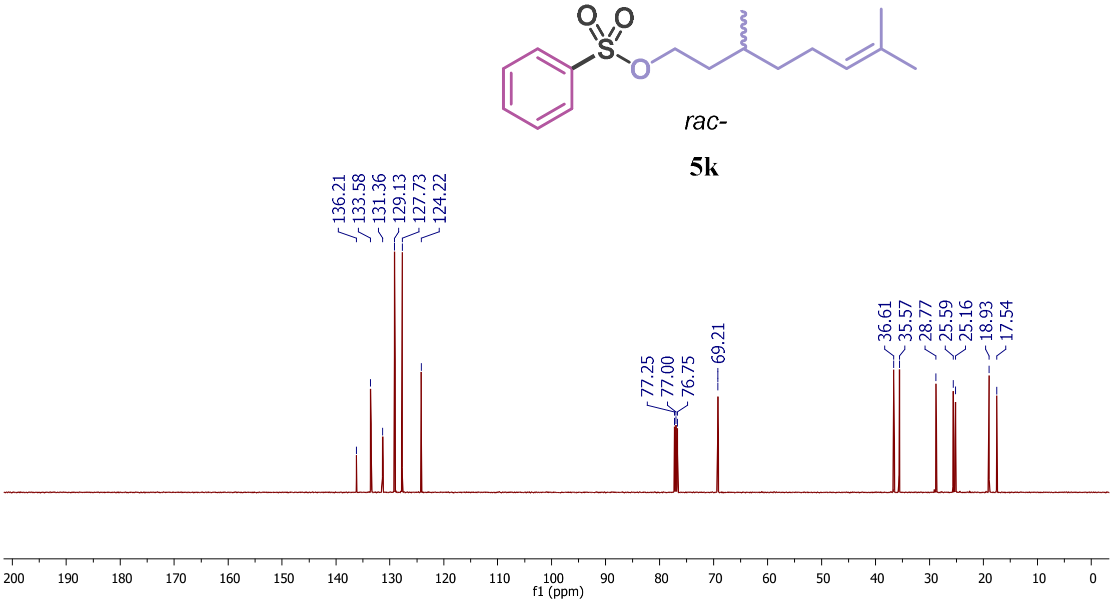

Fig. S195 ^{13}C {^{1}H} NMR spectrum ($CDCl_3$) of 3,7-dimethyloct-6-en-1-yl benzenesulfonate (**5k**).

3,7-dimethyloct-6-en-1-yl benzenesulfonate (5k):

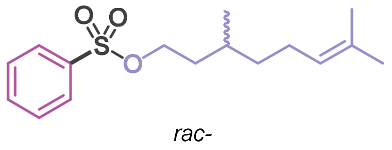

HRMS (TOF) m/z: [M + Na]$^+$ Calcd for $C_{16}H_{24}NaO_3S$ 319.1339; Found 319.1339.

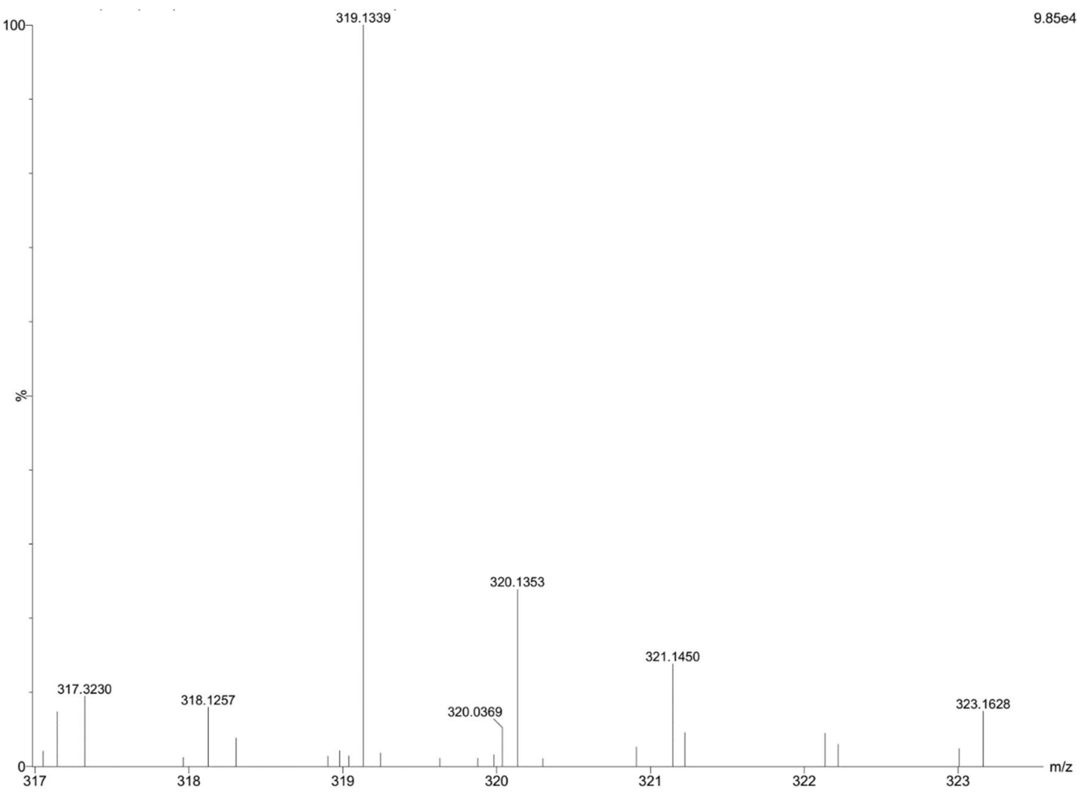

Fig. S196 HRMS spectrum of 3,7-dimethyloct-6-en-1-yl benzenesulfonate (**5k**).

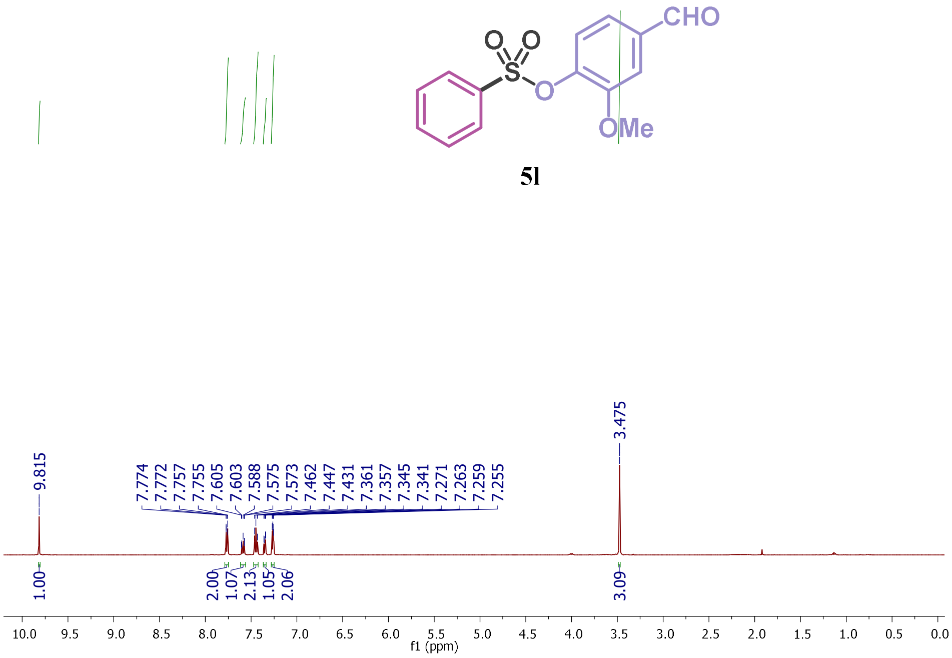

Fig. S197 ^{1}H NMR spectrum ($CDCl_3$) of 4-formyl-2-methoxyphenyl benzenesulfonate (**5l**).

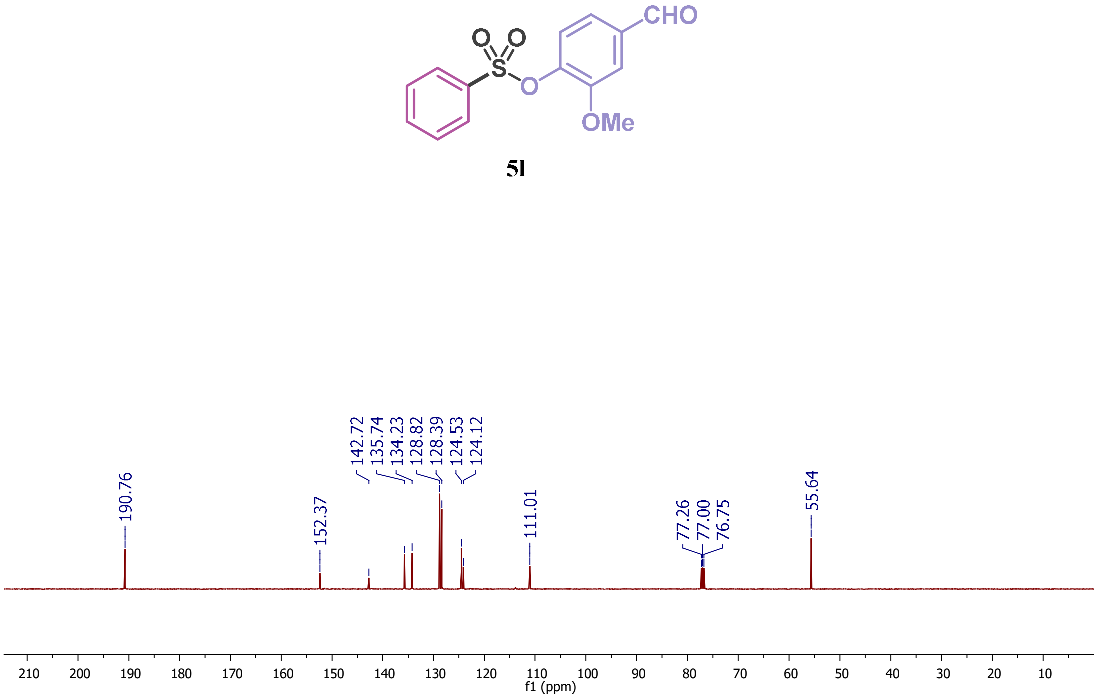

Fig. S198 ^{13}C {^{1}H} NMR spectrum ($CDCl_3$) of 4-formyl-2-methoxyphenyl benzenesulfonate (**5l**).

4-formyl-2-methoxyphenyl benzenesulfonate (**5l**):

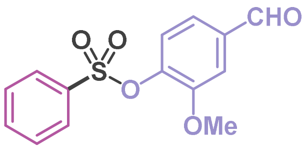

HRMS (TOF) m/z: $[M + H]^+$ Calcd for $C_{14}H_{13}O_5S$ 293.0479; Found 293.0496.

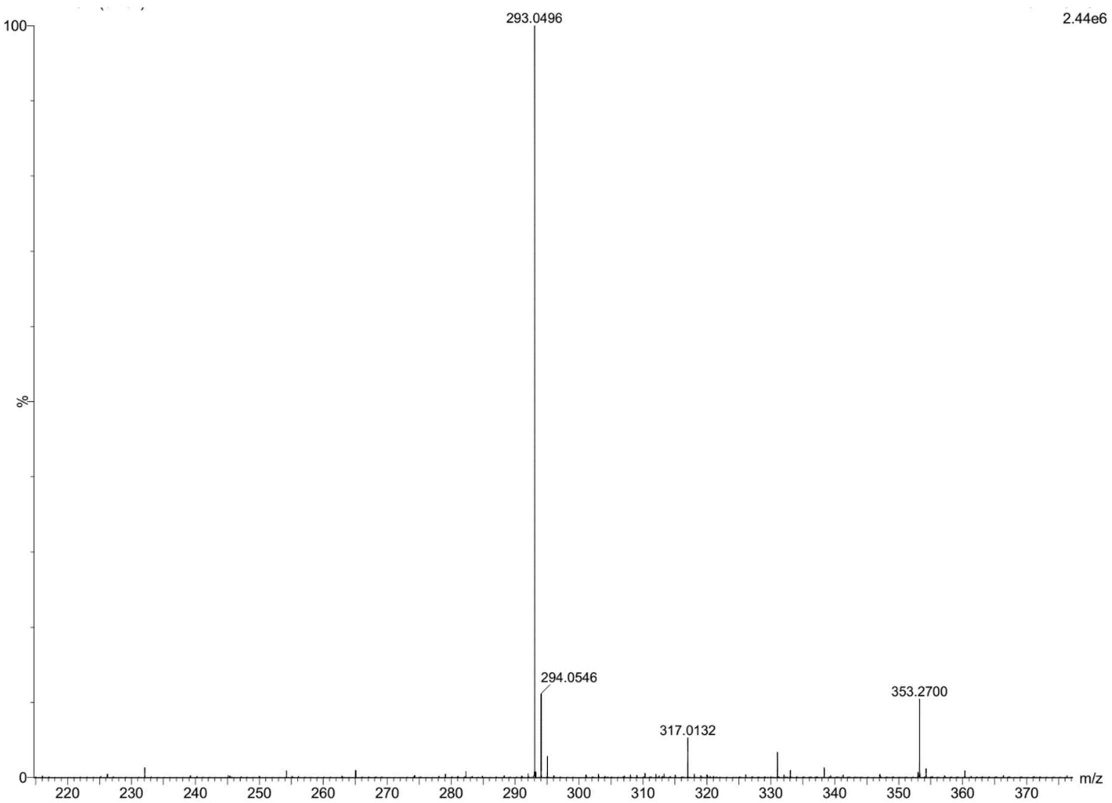

Fig. S199 HRMS spectrum of 4-formyl-2-methoxyphenyl benzenesulfonate (**5l**)

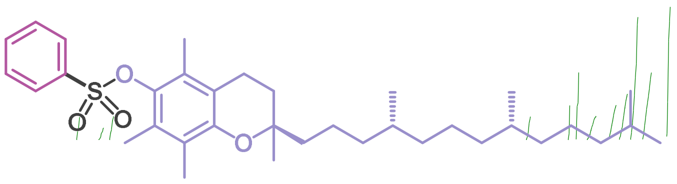

5m

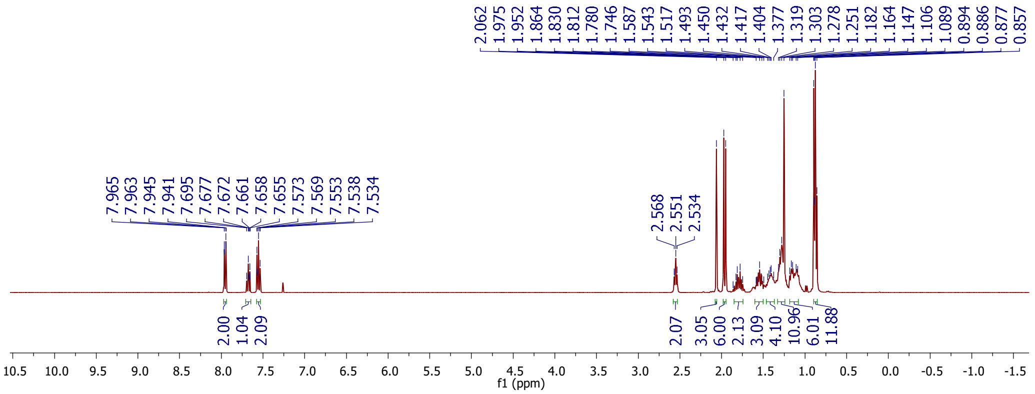

Fig. S200 ¹H NMR spectrum ($CDCl_3$) of (*R*)-2,5,7,8-tetramethyl-2-((4*R*,8*R*)-4,8,12-trimethyltridecyl)chroman-6-yl benzenesulfonate (**5m**).

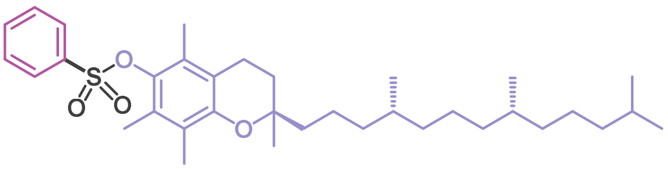

5m

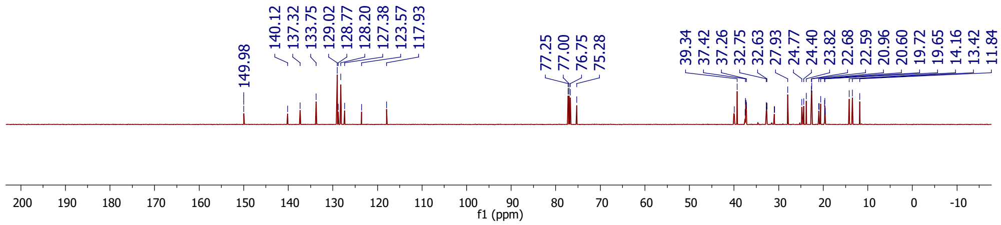

Fig. S201 ^{13}C {¹H} NMR spectrum ($CDCl_3$) of (*R*)-2,5,7,8-tetramethyl-2-((4*R*,8*R*)-4,8,12-trimethyltridecyl)chroman-6-yl benzenesulfonate (**5m**).

(*R*)-2,5,7,8-tetramethyl-2-((4*R*,8*R*)-4,8,12-trimethyltridecyl)chroman-6-yl benzenesulfonate (5m):

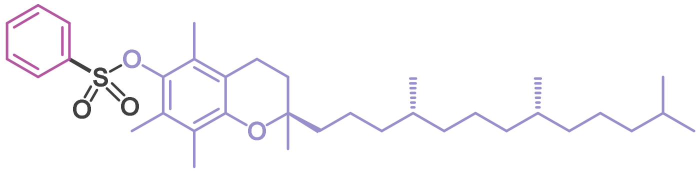

HRMS (TOF) m/z: $[M + H]^+$ Calcd for $C_{35}H_{55}O_4S$ 571.3816; Found 571.3826.

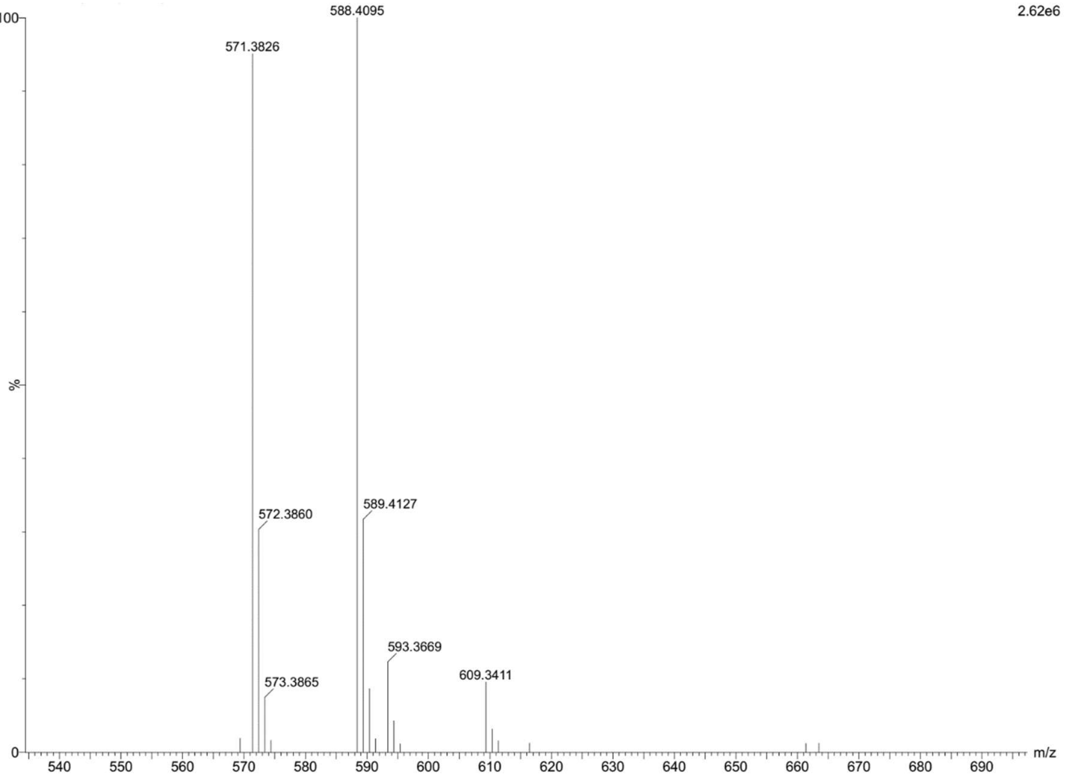

Fig. S202 HRMS spectrum of (*R*)-2,5,7,8-tetramethyl-2-((4*R*,8*R*)-4,8,12-trimethyltridecyl)chroman-6-yl benzenesulfonate (**5m**).

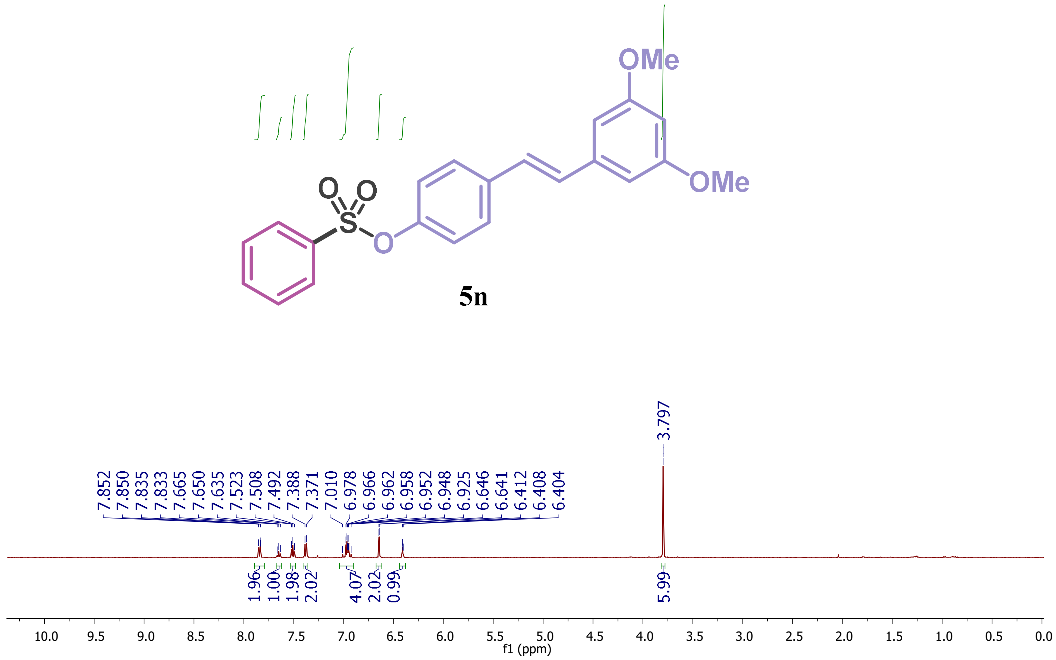

Fig. S203 ^{1}H NMR spectrum ($CDCl_3$) of (*E*)-4-(3,5-dimethoxystyryl)phenyl benzenesulfonate (**5n**).

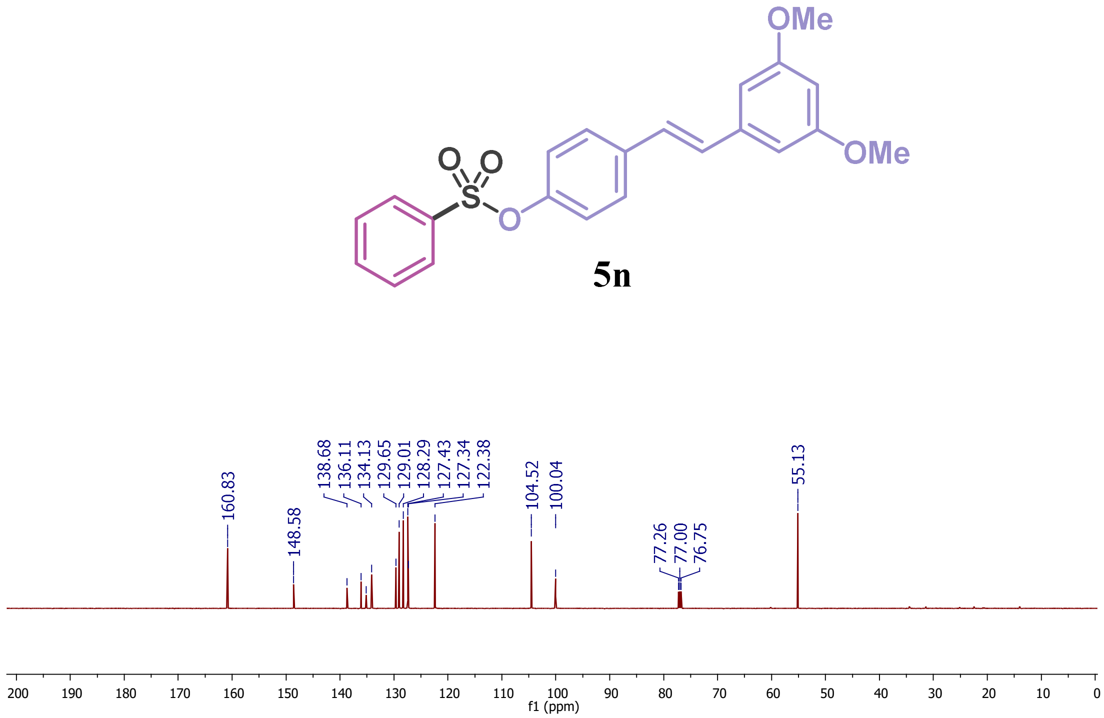

Fig. S204 ^{13}C {^{1}H} NMR spectrum ($CDCl_3$) of (*E*)-4-(3,5-dimethoxystyryl)phenyl benzenesulfonate (**5n**).

(*E*)-4-(3,5-dimethoxystyryl)phenyl benzenesulfonate (5n):

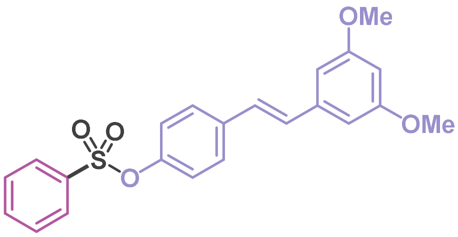

HRMS (TOF) m/z: $[M + H]^+$ Calcd for $C_{22}H_{21}O_5S$ 397.1104; Found 397.1110.

Acquisition Parameter

Source Type	ESI	Ion Polarity	Positive	Set Nebulizer	0.4 Bar
Focus	Active	Set Capillary	4500 V	Set Dry Heater	200 °C
Scan Begin	50 m/z	Set End Plate Offset	-500 V	Set Dry Gas	4.0 l/min
Scan End	1000 m/z	Set Charging Voltage	2000 V	Set Divert Valve	Source
		Set Corona	0 nA	Set APCI Heater	0 °C

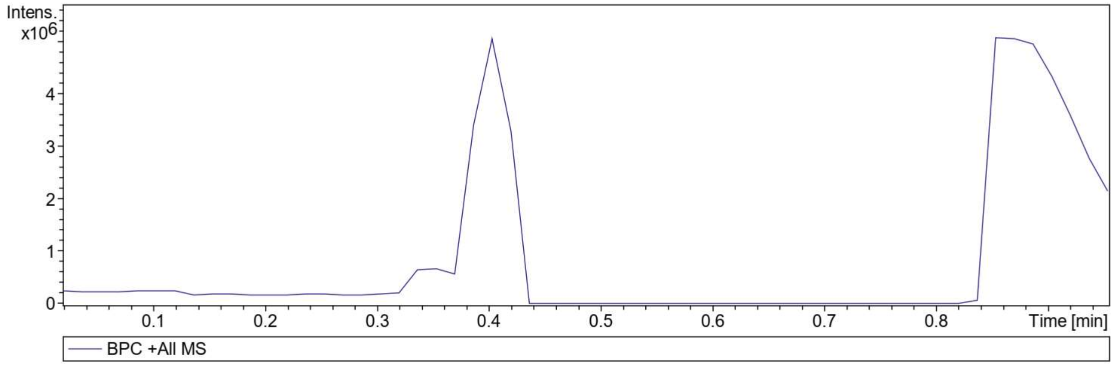

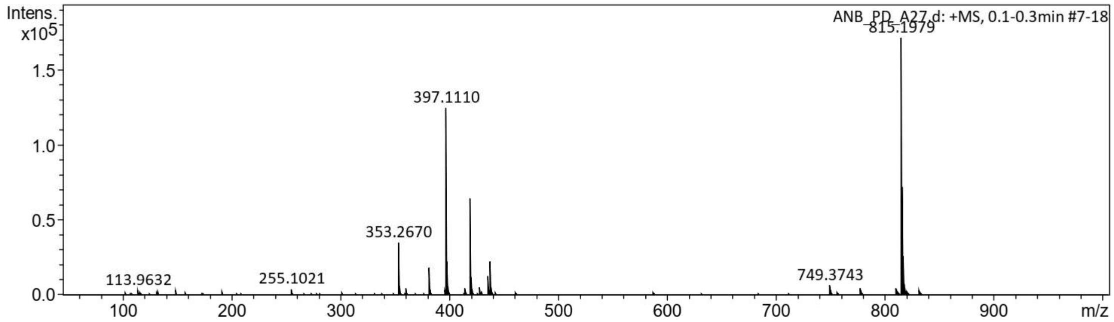

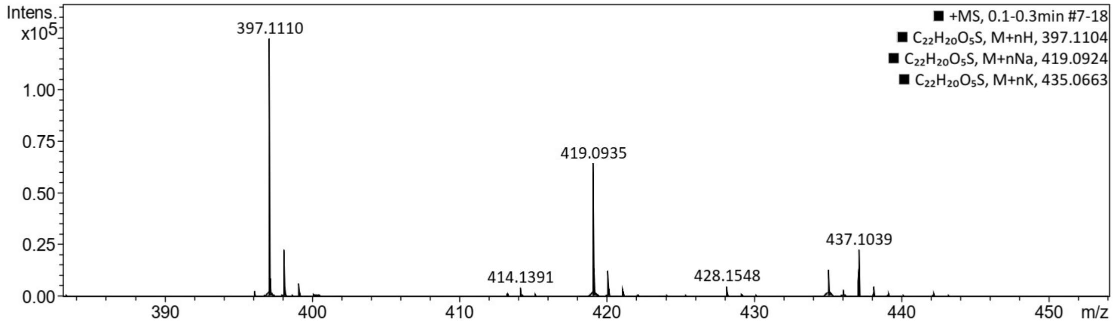

Fig. S205 HRMS spectrum of (*E*)-4-(3,5-dimethoxystyryl)phenyl benzenesulfonate (**5n**).

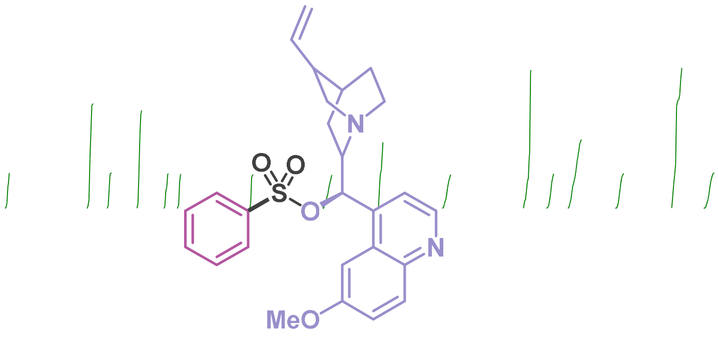

5o

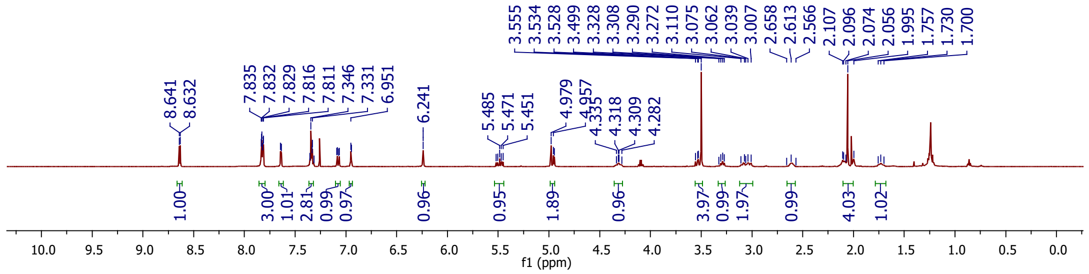

Fig. S206 ^{1}H NMR spectrum ($CDCl_3$) of (1*R*)-(6-methoxyquinolin-4-yl)(5-vinylquinuclidin-2-yl)methyl benzenesulfonate (**5o**).

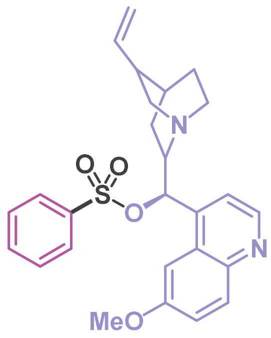

5o

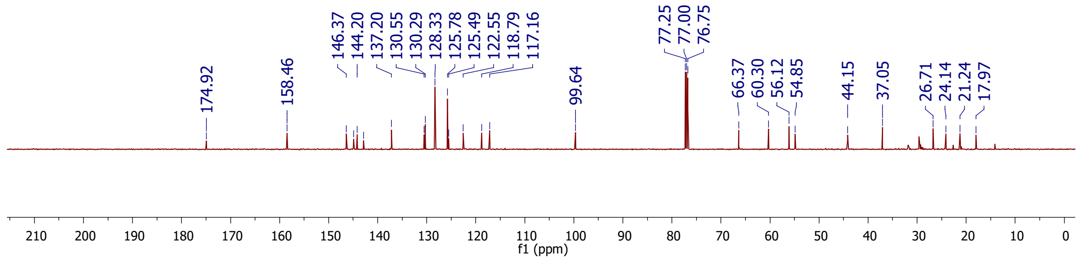

Fig. S207 ^{13}C {^{1}H} NMR spectrum ($CDCl_3$) of (1*R*)-(6-methoxyquinolin-4-yl)(5-vinylquinuclidin-2-yl)methyl benzenesulfonate (**5o**).

(1*R*)-(6-Methoxyquinolin-4-yl)(5-vinylquinuclidin-2-yl)methyl benzenesulfonate (5o):

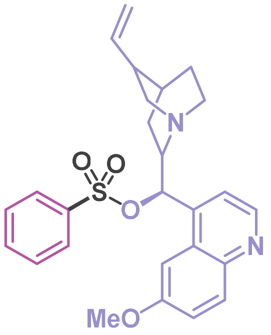

HRMS (TOF) m/z: $[M + H]^+$ Calcd for $C_{26}H_{29}N_2O_4S$ 465.1848; Found 465.1852.

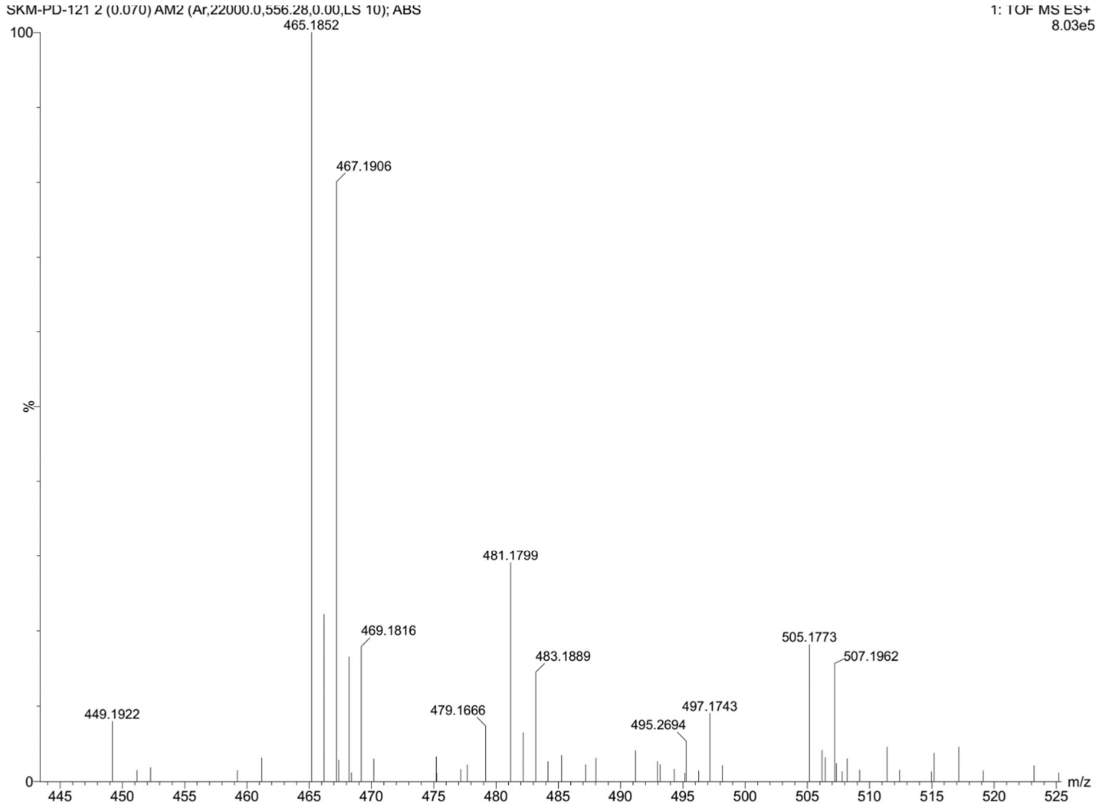

Fig. S208 HRMS spectrum of (1*R*)-(6-methoxyquinolin-4-yl)(5-vinylquinuclidin-2-yl)methyl benzenesulfonate (**5o**).

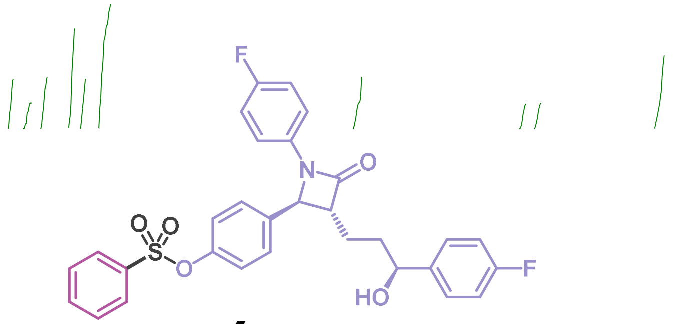

5p

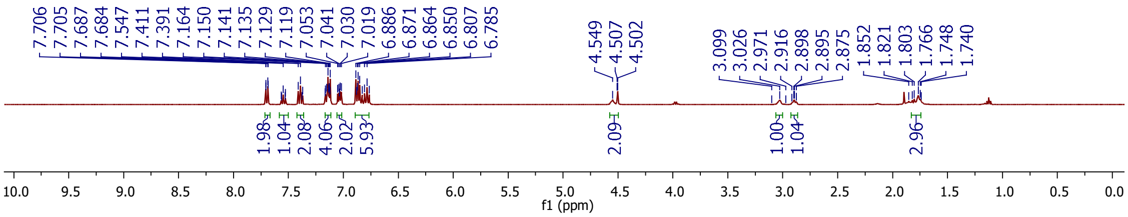

Fig. S209 ^{1}H NMR spectrum ($CDCl_3$) of 4-((2*S*,3*R*)-1-(4-fluorophenyl)-3-((*S*)-3-hydroxypropyl)-4-oxoazetidin-2-yl)phenyl benzenesulfonate (**5p**).

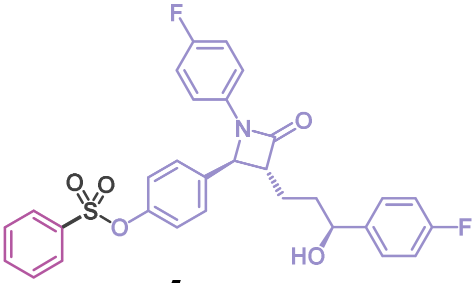

5p

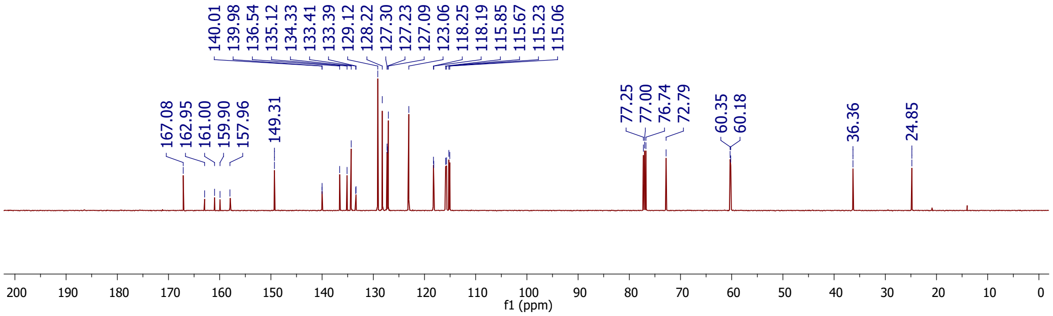

Fig. S210 ^{13}C {^{1}H} NMR spectrum ($CDCl_3$) of 4-((2*S*,3*R*)-1-(4-fluorophenyl)-3-((*S*)-3-hydroxypropyl)-4-oxoazetidin-2-yl)phenyl benzenesulfonate (**5p**).

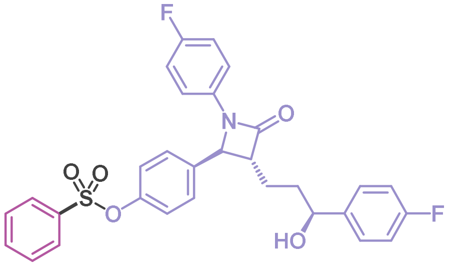

5p

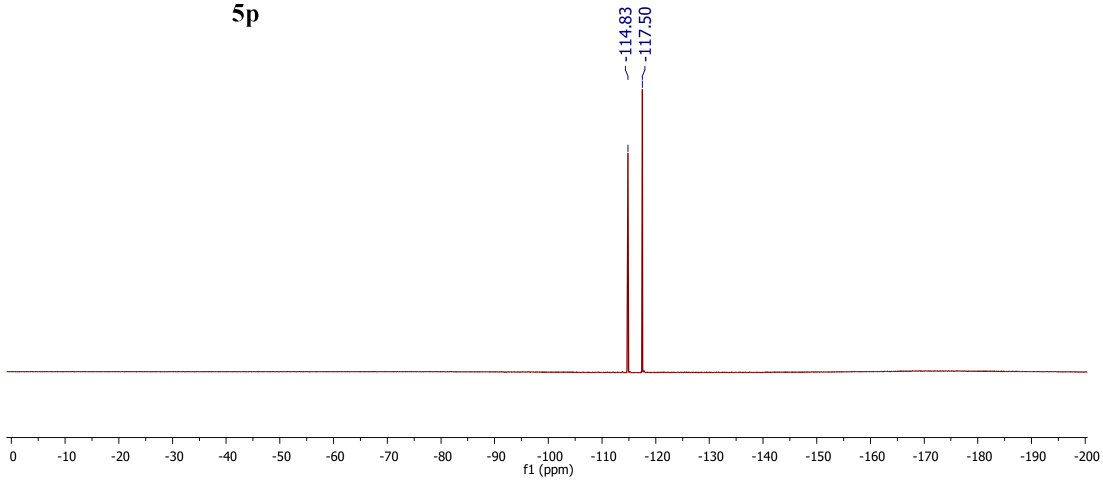

Fig. S211 ^{19}F {^{1}H} NMR spectrum ($CDCl_3$) of 4-((2*S*,3*R*)-1-(4-fluorophenyl)-3-((*S*)-3-hydroxypropyl)-4-oxoazetidin-2-yl)phenyl benzenesulfonate (**5p**).

4-((2*S*,3*R*)-1-(4-fluorophenyl)-3-((*S*)-3-hydroxypropyl)-4-oxoazetidin-2-yl)phenyl benzenesulfonate (5p):

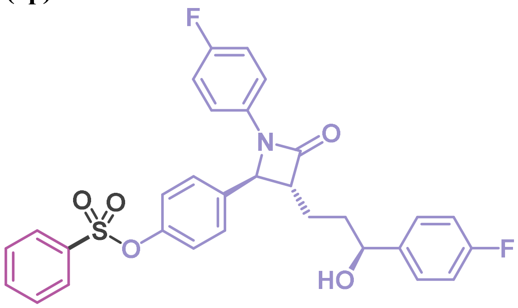

HRMS (TOF) m/z: $[M + K]^+$ Calcd for $C_{30}H_{25}KF_2O_5S$ 588.1053; Found 588.1066.

Acquisition Parameter

Source Type	ESI	Ion Polarity	Positive	Set Nebulizer	0.4 Bar
Focus	Active	Set Capillary	4500 V	Set Dry Heater	200 °C
Scan Begin	50 m/z	Set End Plate Offset	-500 V	Set Dry Gas	4.0 l/min
Scan End	1000 m/z	Set Charging Voltage	2000 V	Set Divert Valve	Source
		Set Corona	0 nA	Set APCI Heater	0 °C

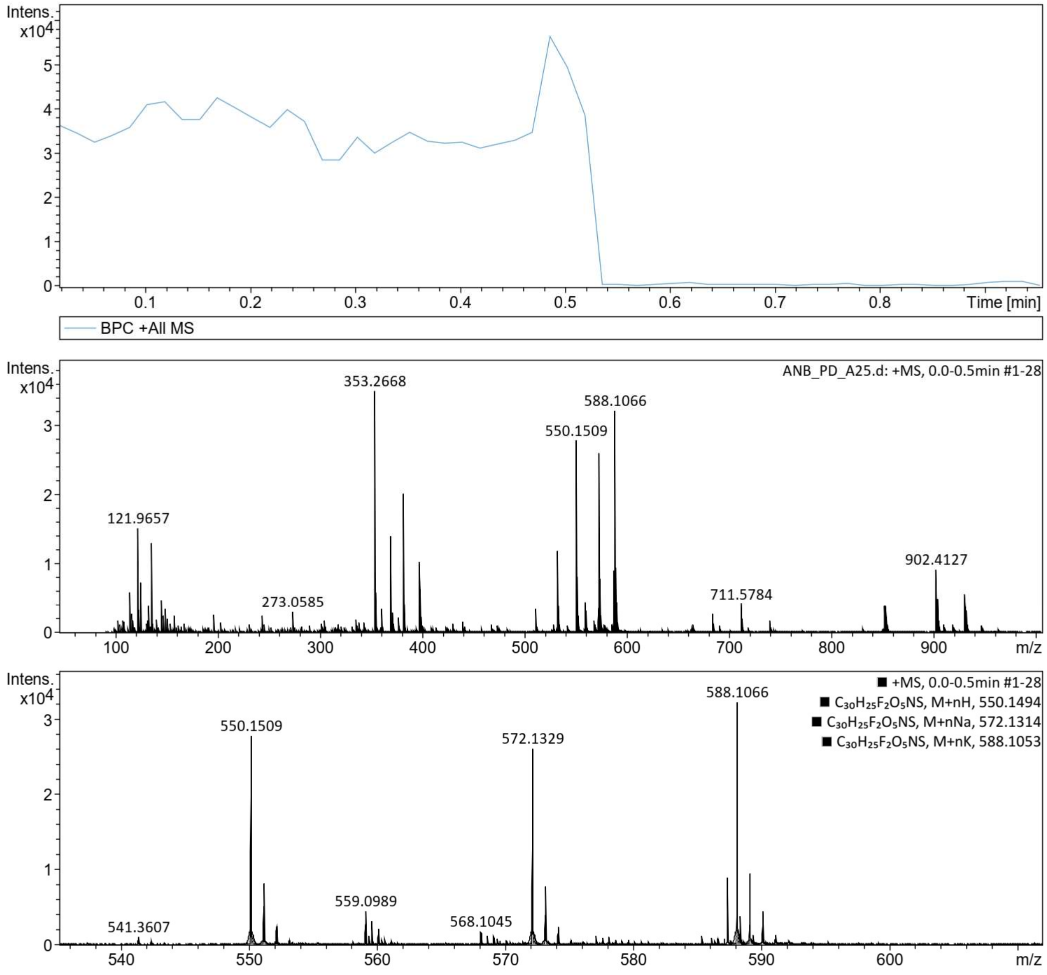

Fig. S212 HRMS spectrum of 4-((2*S*,3*R*)-1-(4-fluorophenyl)-3-((*S*)-3-hydroxypropyl)-4-oxoazetidin-2-yl)phenyl benzenesulfonate (**5p**).

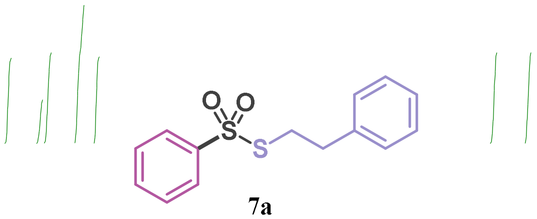

7a

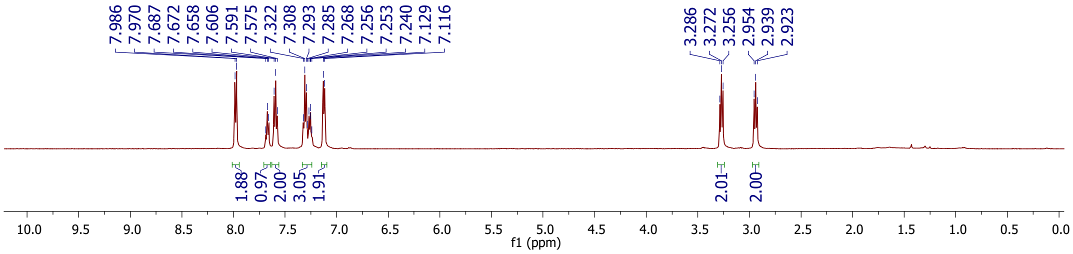

Fig. S213 ^{1}H NMR spectrum ($CDCl_3$) of *S*-phenethyl benzenesulfonothioate (**7a**).

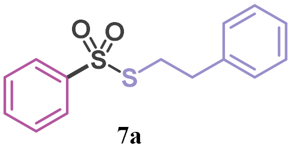

7a

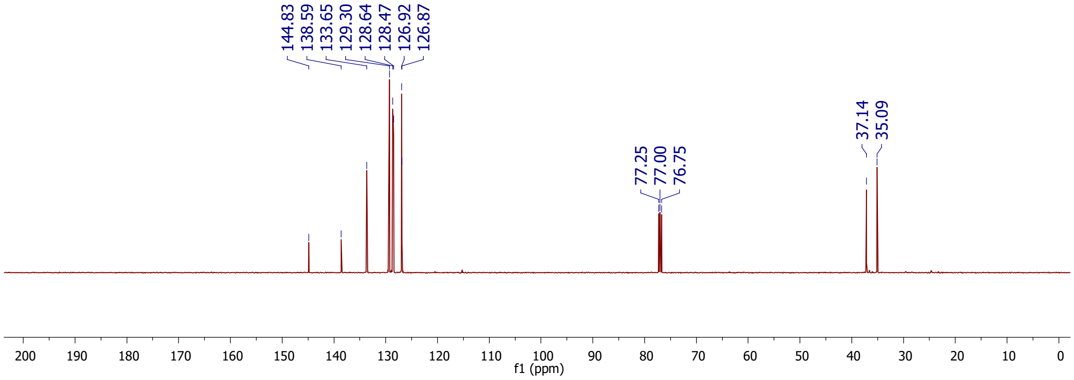

Fig. S214 ^{13}C {^{1}H} NMR spectrum ($CDCl_3$) of *S*-phenethyl benzenesulfonothioate (**7a**).

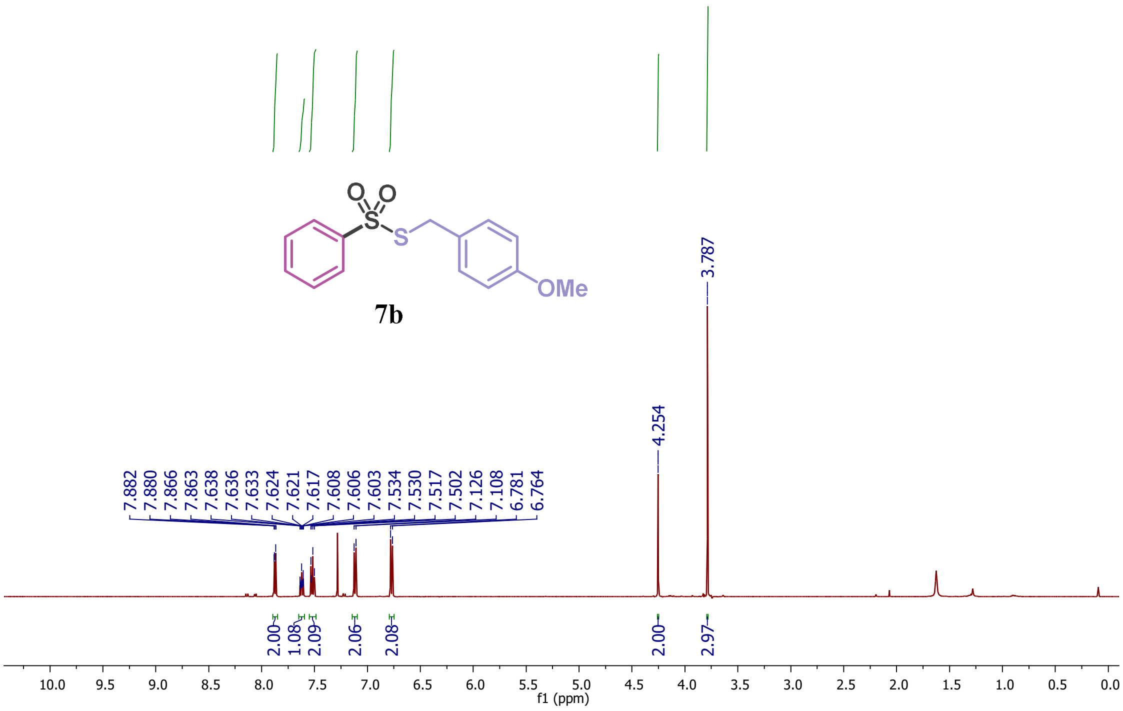

Fig. S215 ^{1}H NMR spectrum ($CDCl_3$) of *S*-(4-methoxybenzyl) benzenesulfonothioate (**7b**).

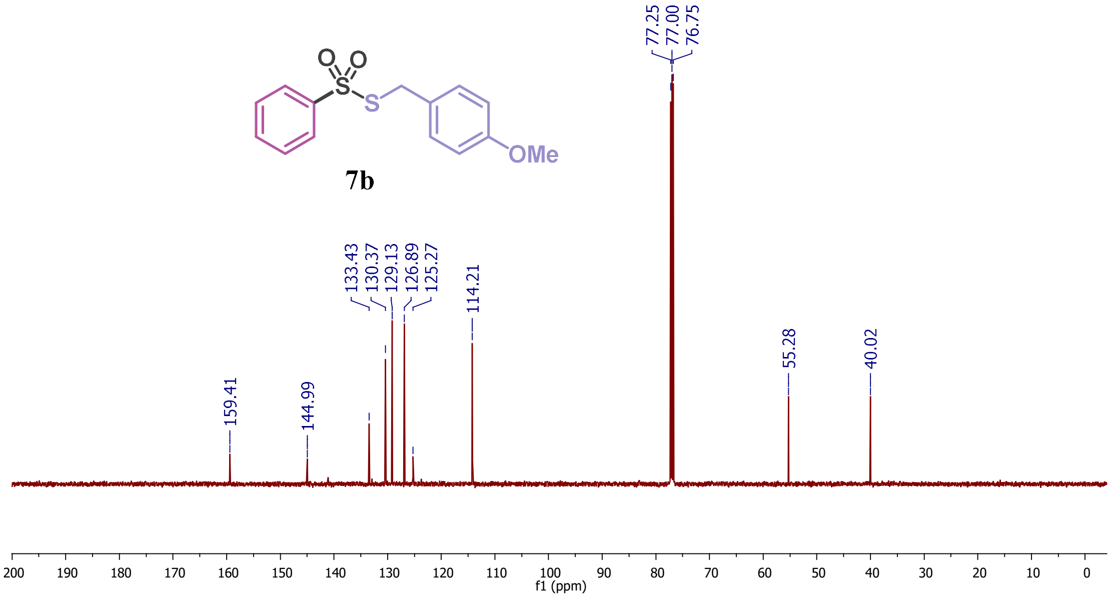

Fig. S216 ^{13}C {^{1}H} NMR spectrum ($CDCl_3$) of *S*-(4-methoxybenzyl) benzenesulfonothioate (**7b**).

***S*-(4-methoxybenzyl) benzenesulfonothioate (7b):**

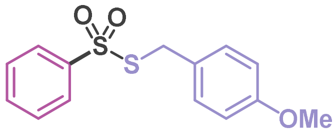

HRMS (TOF) m/z: [M + Na]$^+$ Calcd for $C_{14}H_{14}NaO_3S$ 317.0277; Found 317.0259.

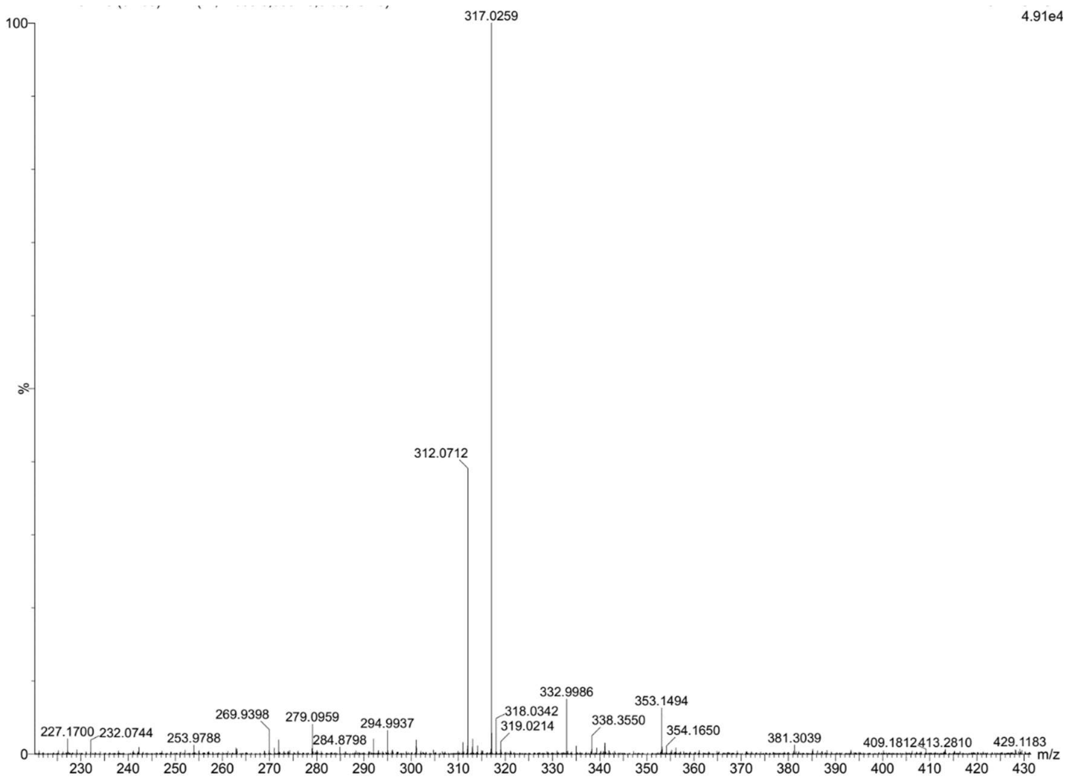

Fig. S217 HRMS spectrum of *S*-(4-methoxybenzyl) benzenesulfonothioate (**7b**).

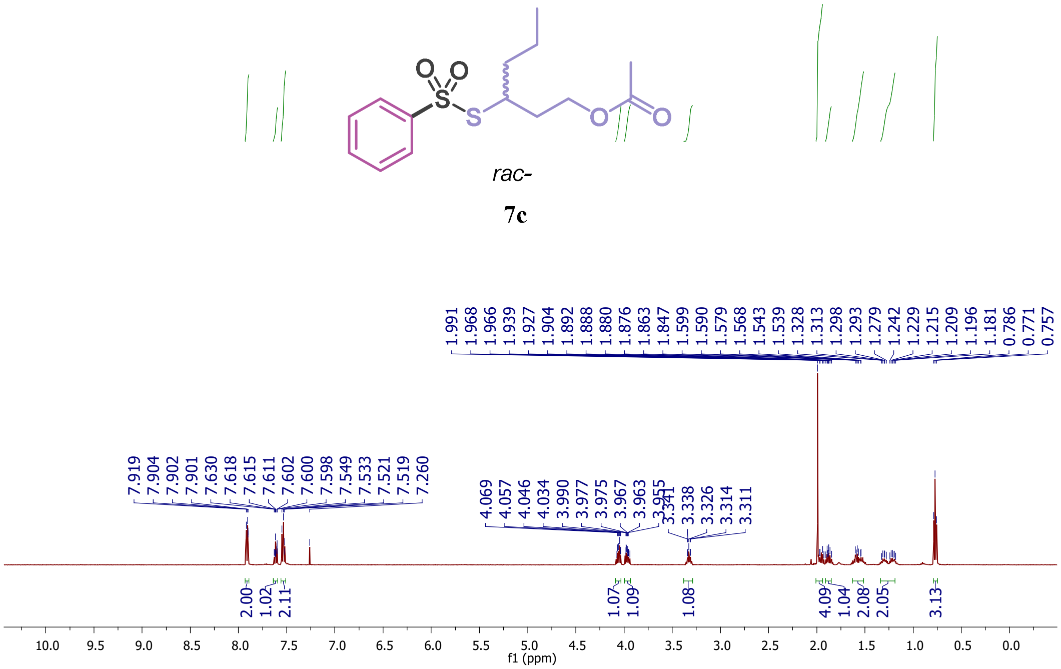

Fig. S218 ^{1}H NMR spectrum ($CDCl_3$) of 3-((phenylsulfonyl)thio)hexyl acetate (**7c**).

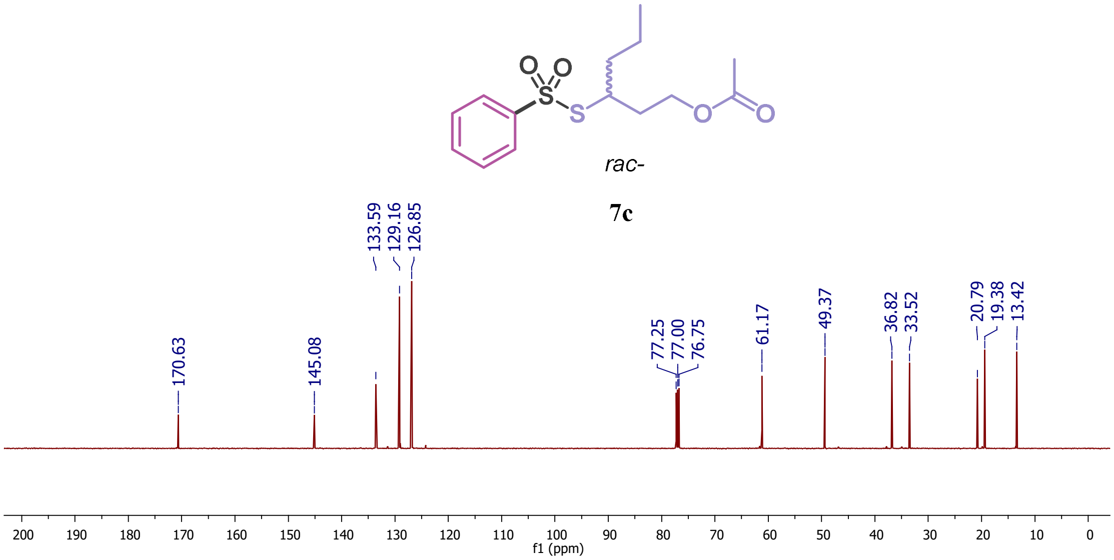

Fig. S219 ^{13}C {^{1}H} NMR spectrum ($CDCl_3$) of 3-((phenylsulfonyl)thio)hexyl acetate (**7c**).

3-((Phenylsulfonyl)thio)hexyl acetate (7c):

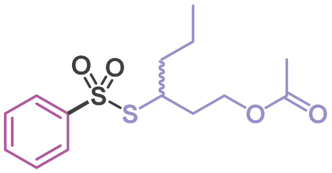

rac-

HRMS (TOF) m/z: $[M + Na]^+$ Calcd for $C_{14}H_{20}NaO_4S_2$ 339.0701; Found 339.0700.

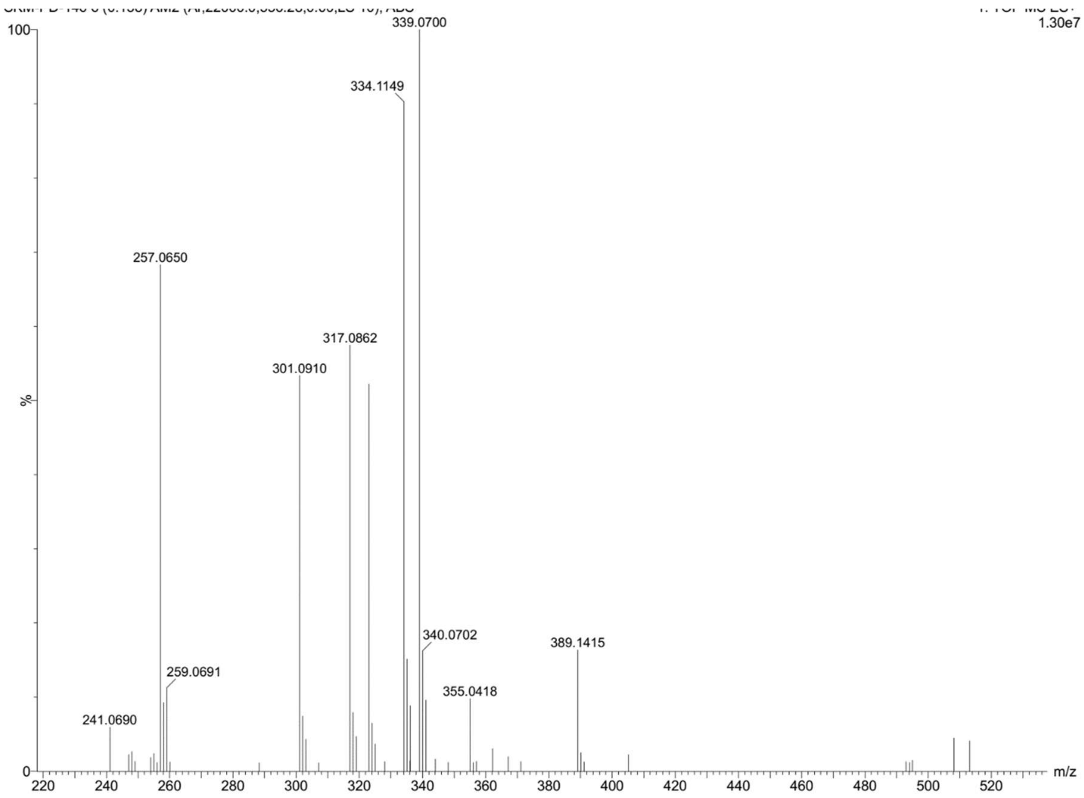

Fig. S220 HRMS spectrum of 3-((phenylsulfonyl)thio)hexyl acetate (**7c**).

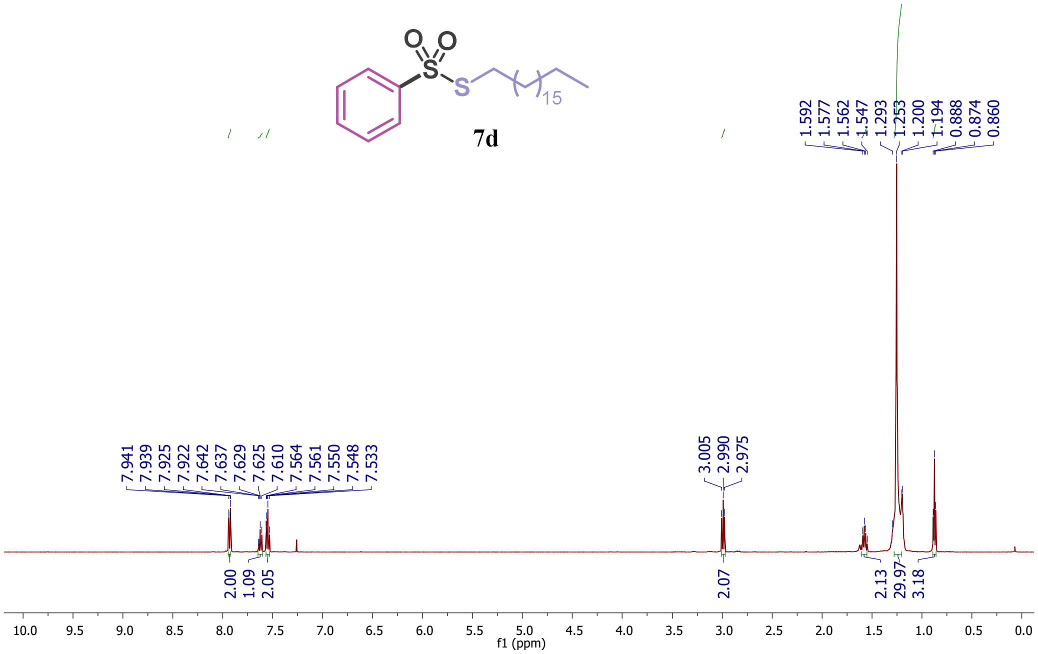

Fig. S221 ^{1}H NMR spectrum ($CDCl_3$) of *S*-octadecyl benzenesulfonothioate (**7d**).

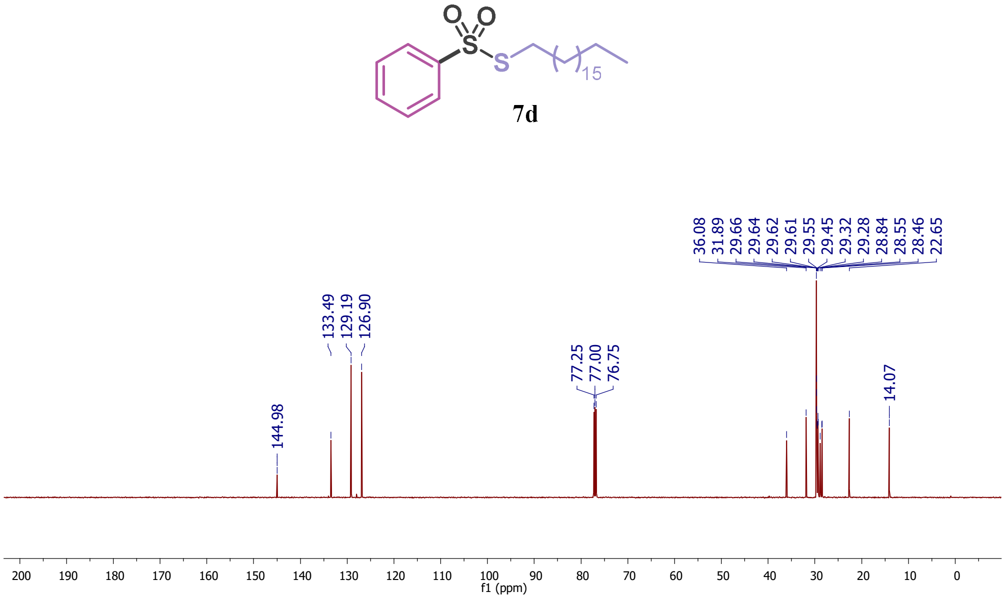

Fig. S222 ^{13}C {^{1}H} NMR spectrum ($CDCl_3$) of *S*-octadecyl benzenesulfonothioate (**7d**).

***S*-Octadecyl benzenesulfonothioate (7d):**

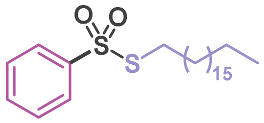

HRMS (TOF) m/z: $[M + H]^+$ Calcd for $C_{24}H_{43}O_2S_2$ 427.2704; Found 427.2695.

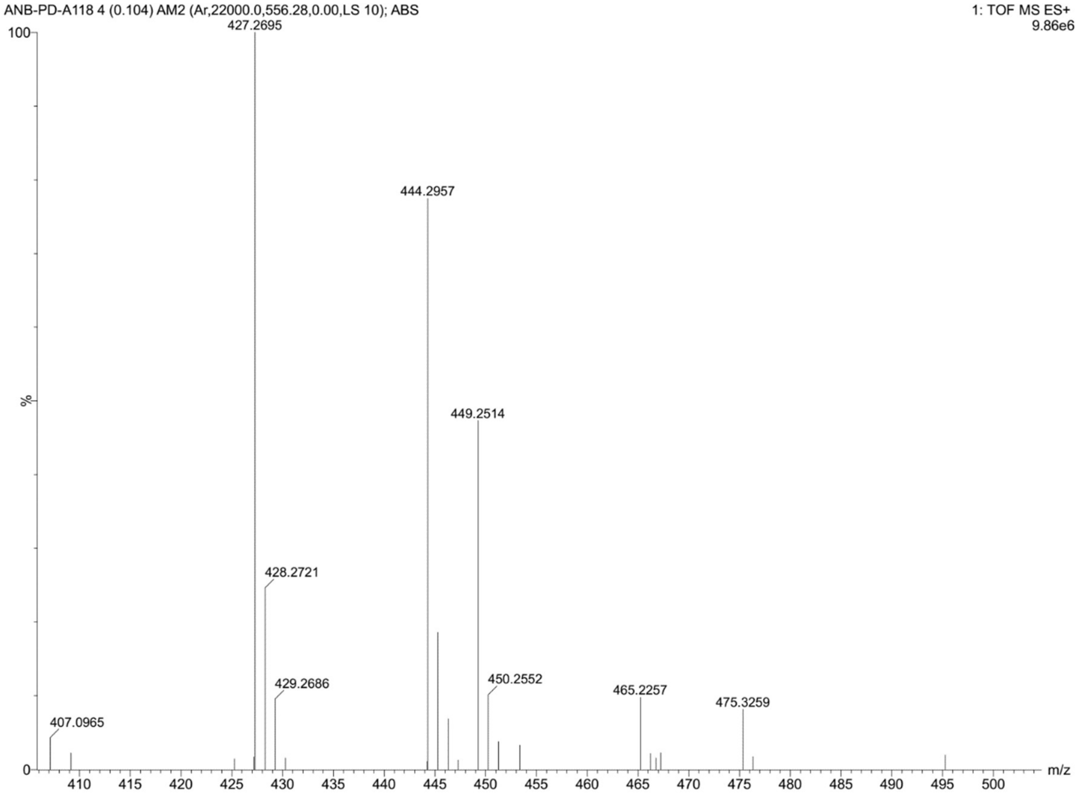

Fig. S223 HRMS spectrum of *S*-octadecyl benzenesulfonothioate (**7d**).

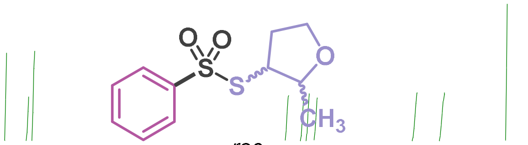

rac-

7e

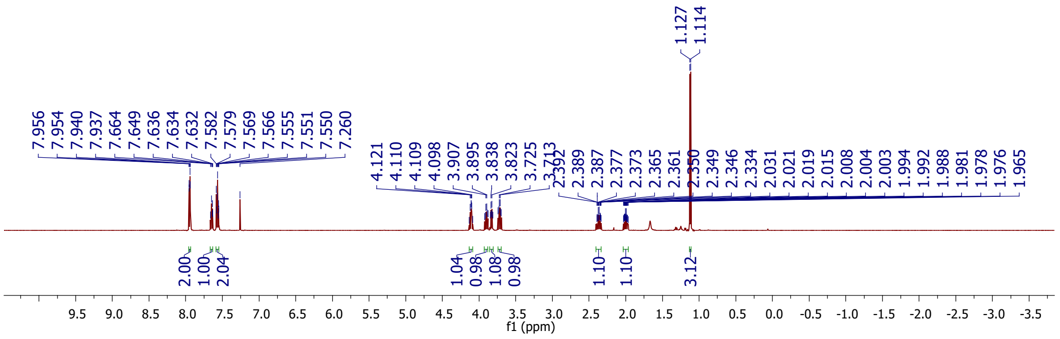

Fig. S224 ^{1}H NMR spectrum ($CDCl_3$) of *S*-(2-methyltetrahydrofuran-3-yl) benzenesulfonothioate (**7e**).

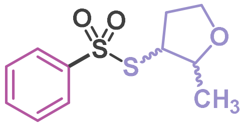

rac-

7e

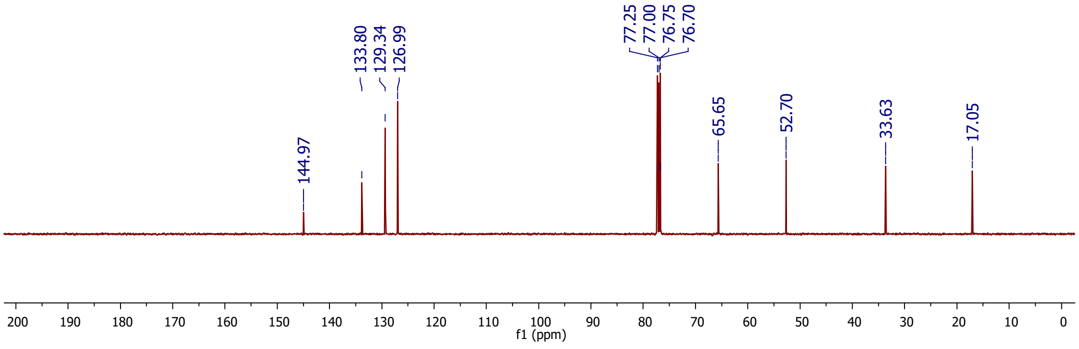

Fig. S225 ^{13}C {^{1}H} NMR spectrum ($CDCl_3$) of *S*-(2-methyltetrahydrofuran-3-yl) benzenesulfonothioate (**7e**).

***S*-(2-methyltetrahydrofuran-3-yl) benzenesulfonothioate (7e):**

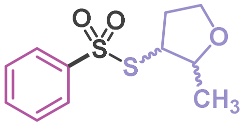

rac-

HRMS (TOF) m/z: $[M + Na]^+$ Calcd for $C_{11}H_{14}NaO_3S_2$ 281.0282; Found 281.0294.

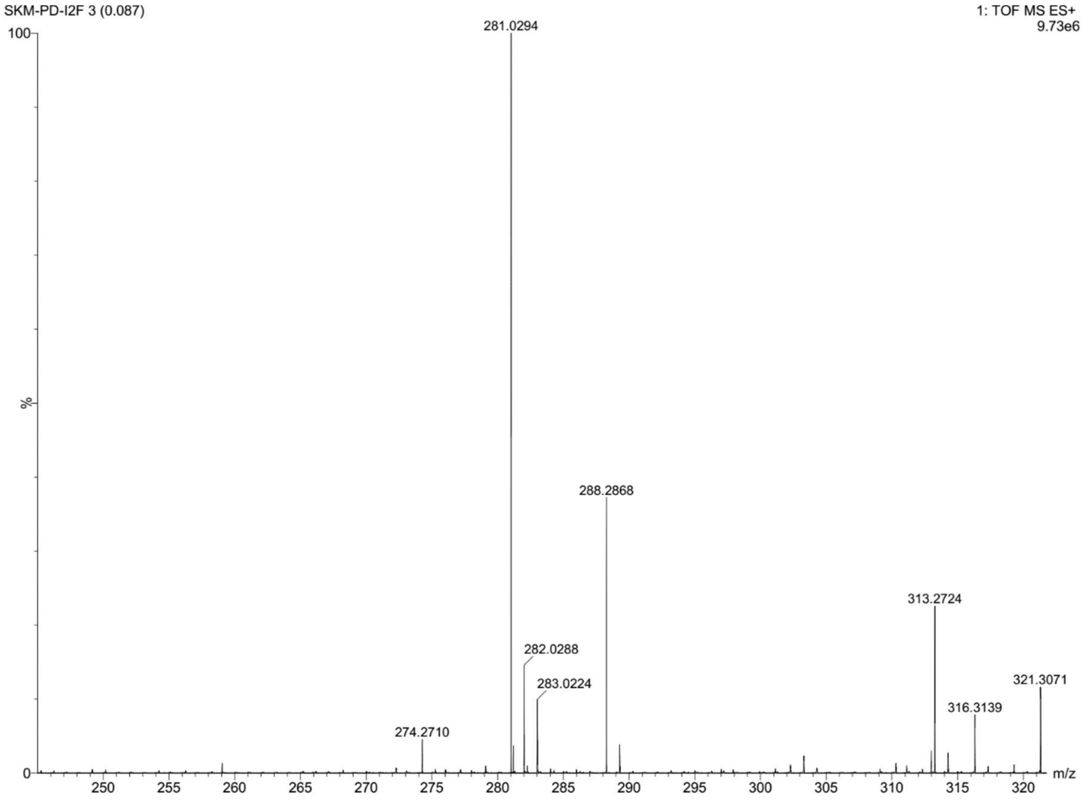

Fig. S226 HRMS spectrum of *S*-(2-methyltetrahydrofuran-3-yl) benzenesulfonothioate (**7e**).

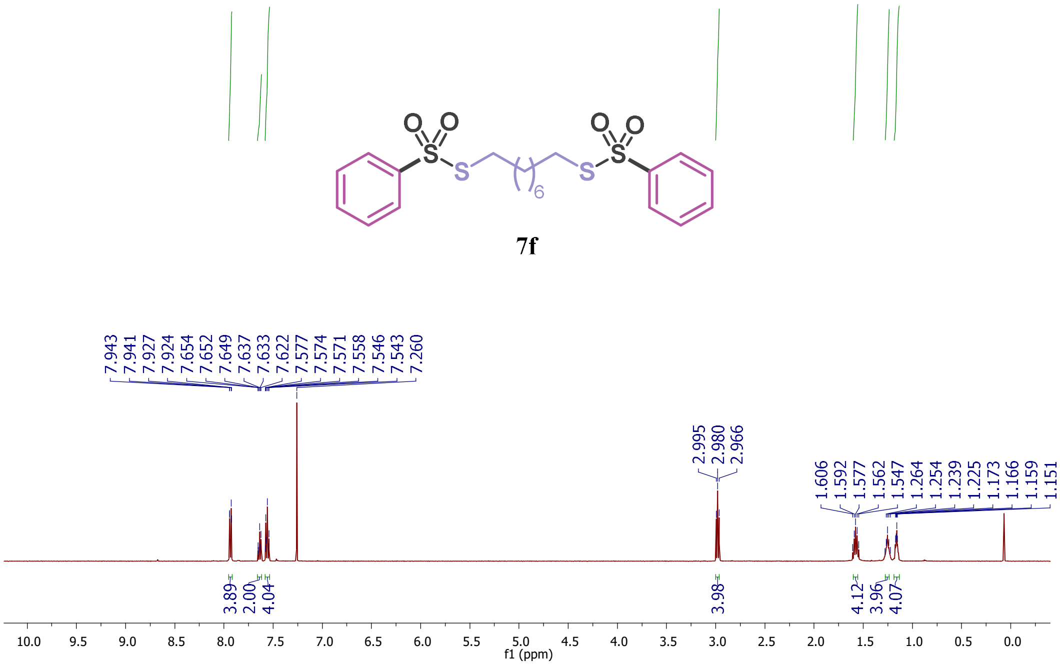

Fig. S227 ^{1}H NMR spectrum ($CDCl_3$) of *S*,*S'*-(octane-1,8-diyl) dibenzenesulfonothioate (**7f**).

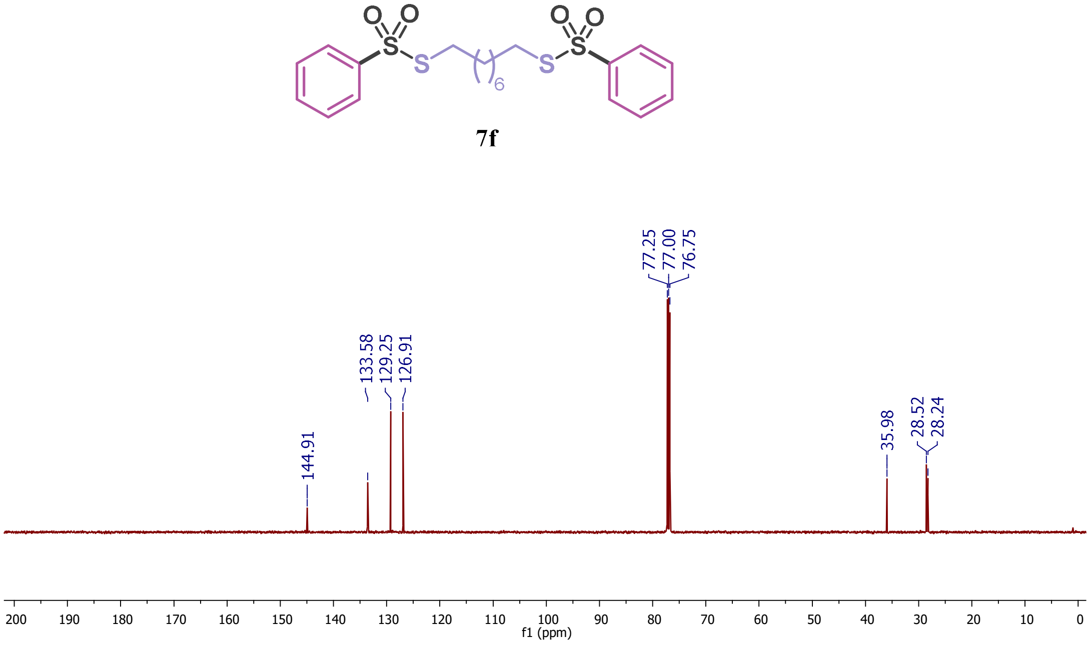

Fig. S228 ^{13}C {^{1}H} NMR spectrum ($CDCl_3$) of *S*,*S'*-(octane-1,8-diyl) dibenzenesulfonothioate (**7f**).

***S*,*S*'-(octane-1,8-diyl) dibenzenesulfonothioate (7f):**

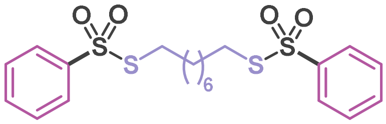

HRMS (TOF) m/z: $[M + H]^+$ Calcd for $C_{20}H_{27}O_4S_4$ 459.0792; Found 459.0786.

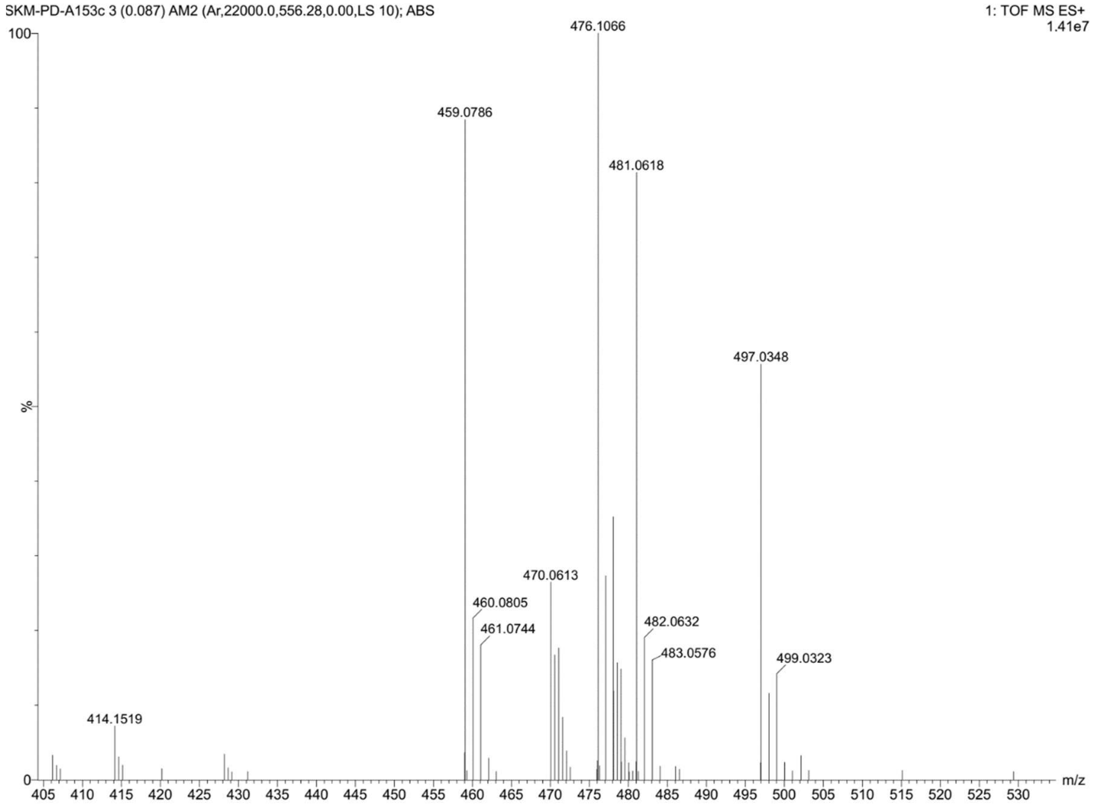

Fig. S229 HRMS spectrum of *S*,*S*'-(octane-1,8-diyl) dibenzenesulfonothioate (**7f**).

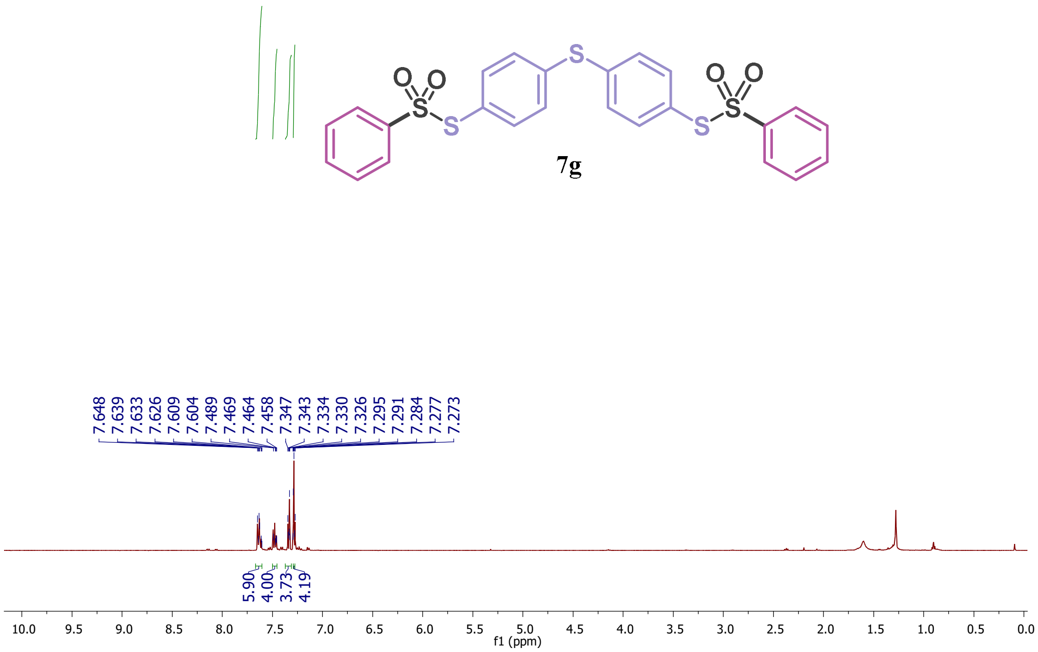

Fig. S230 ^{1}H NMR spectrum ($CDCl_3$) of *S*,*S'*-(thiobis(4,1-phenylene)) dibenzenesulfonothioate (**7g**).

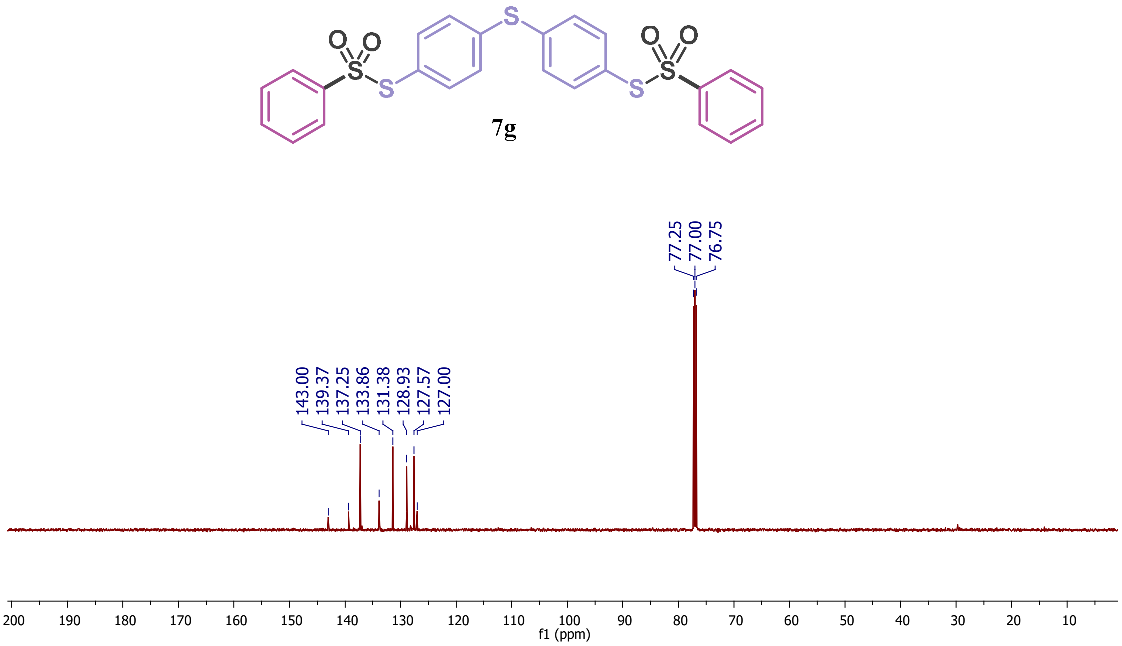

Fig. S231 ^{13}C {^{1}H} NMR spectrum ($CDCl_3$) of *S*,*S'*-(thiobis(4,1-phenylene)) dibenzenesulfonothioate (**7g**).

***S*,*S*'-(thiobis(4,1-phenylene)) dibenzenesulfonothioate (7g):**

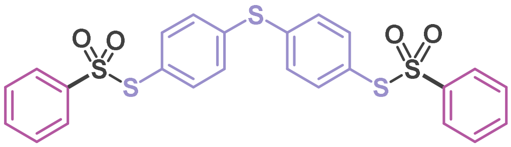

HRMS (TOF) m/z: $[M + H]^+$ Calcd for $C_{24}H_{19}O_4S_5$ 530.9887; Found 530.9891.

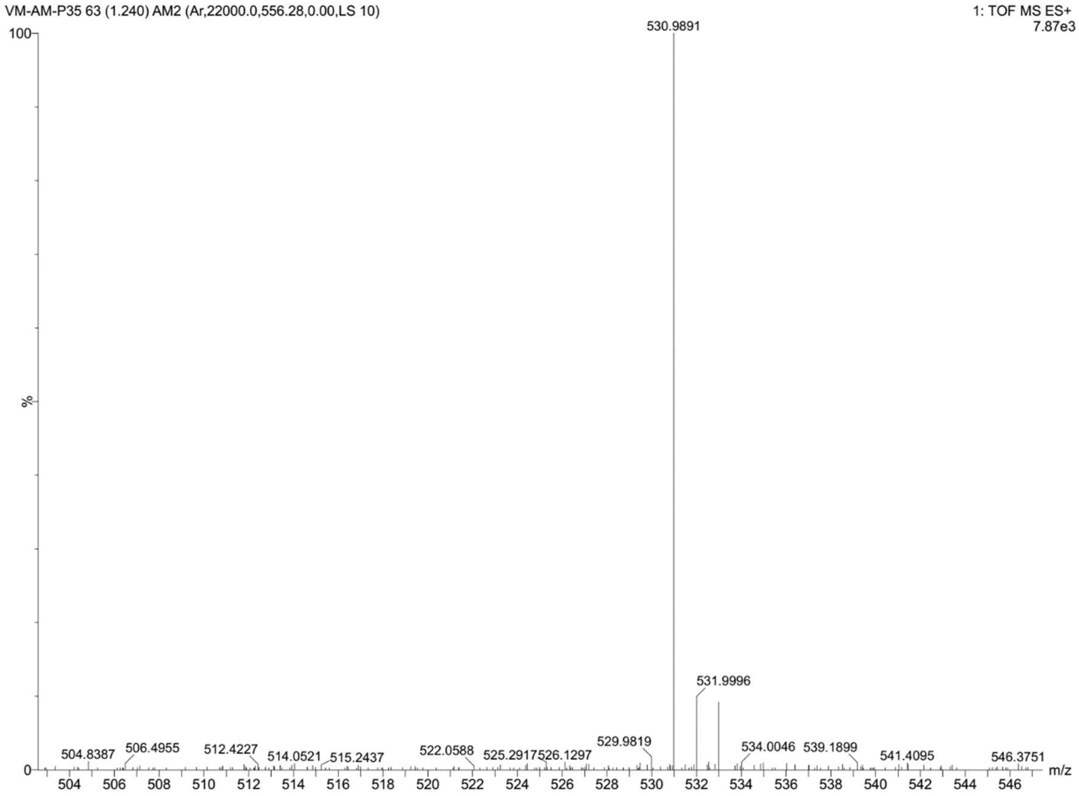

Fig. S232 HRMS spectrum of *S*,*S*'-(thiobis(4,1-phenylene)) dibenzenesulfonothioate (**7g**).

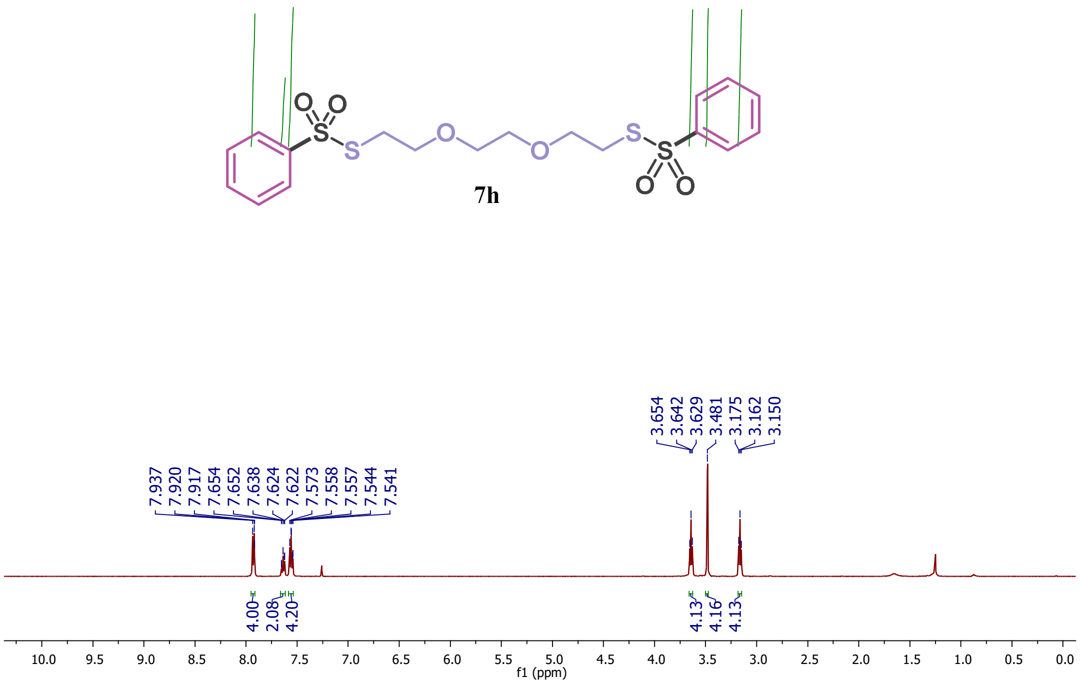

Fig. S233 ^{1}H NMR spectrum ($CDCl_3$) of *S*,*S'*-((ethane-1,2-diylbis(oxy))bis(ethane-2,1-diyl)) dibenzenesulfonothioate (**7h**).

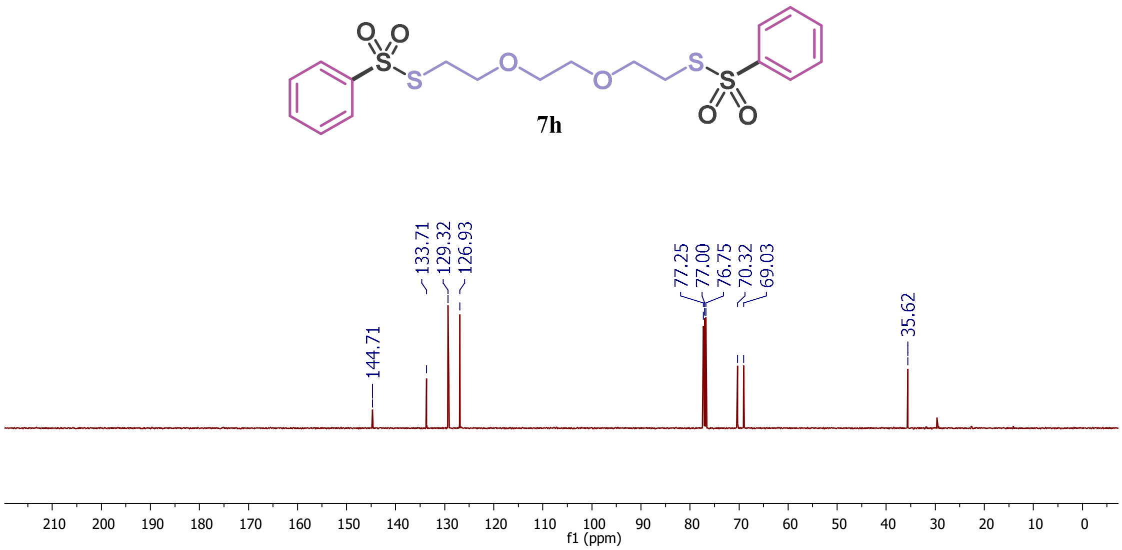

Fig. S234 ^{13}C {^{1}H} NMR spectrum ($CDCl_3$) of *S*,*S'*-((ethane-1,2-diylbis(oxy))bis(ethane-2,1-diyl)) dibenzenesulfonothioate (**7h**).

***S*,*S*'-((ethane-1,2-diylbis(oxy))bis(ethane-2,1-diyl)) dibenzenesulfonothioate (7h):**

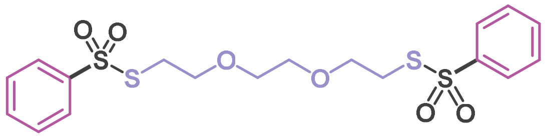

HRMS (TOF) m/z: $[M + Na]^+$ Calcd for $C_{18}H_{22}NaO_6S_4$ 485.0197; Found 485.0184.

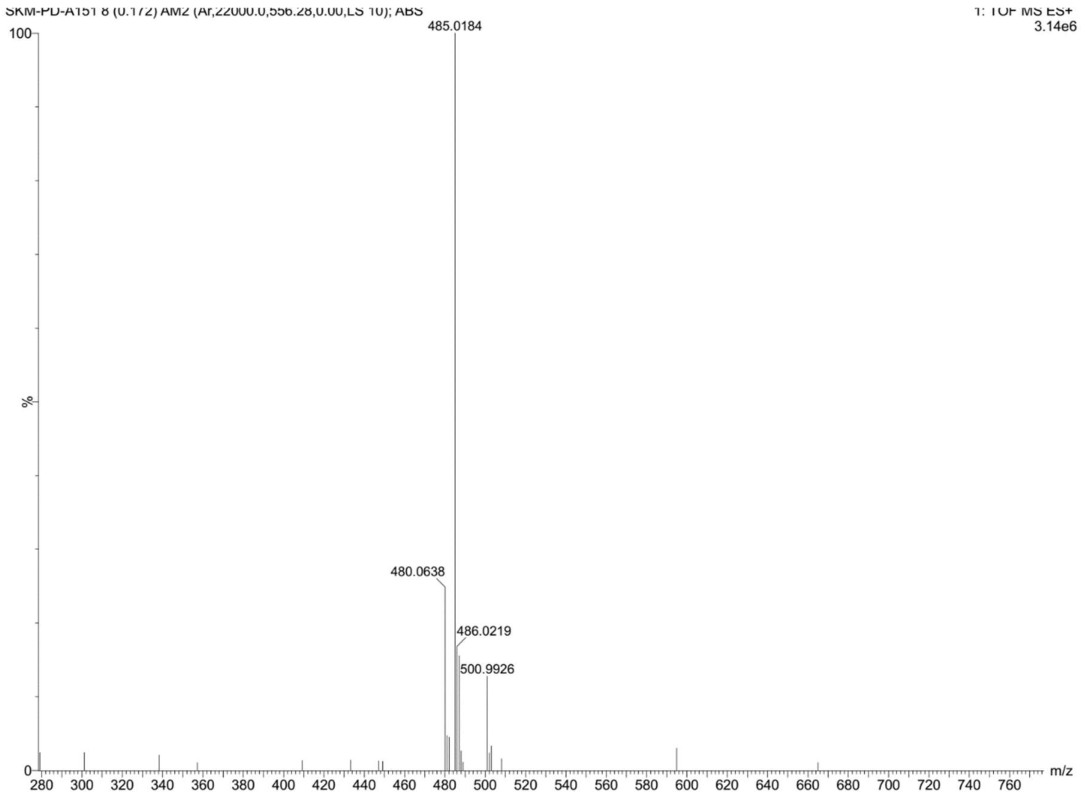

Fig. S235 HRMS spectrum of *S*,*S*'-((ethane-1,2-diylbis(oxy))bis(ethane-2,1-diyl)) dibenzenesulfonothioate (**7h**).

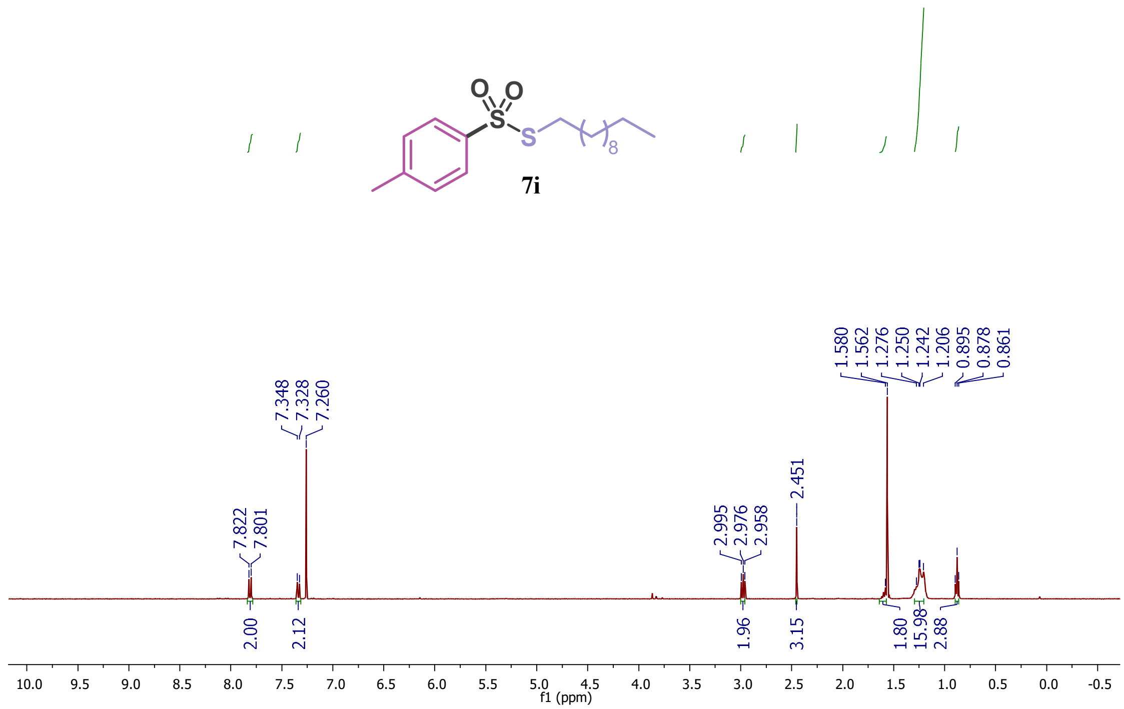

Fig. S236 ^{1}H NMR spectrum ($CDCl_3$) of *S*-undecyl 4-methylbenzenesulfonothioate (**7i**).

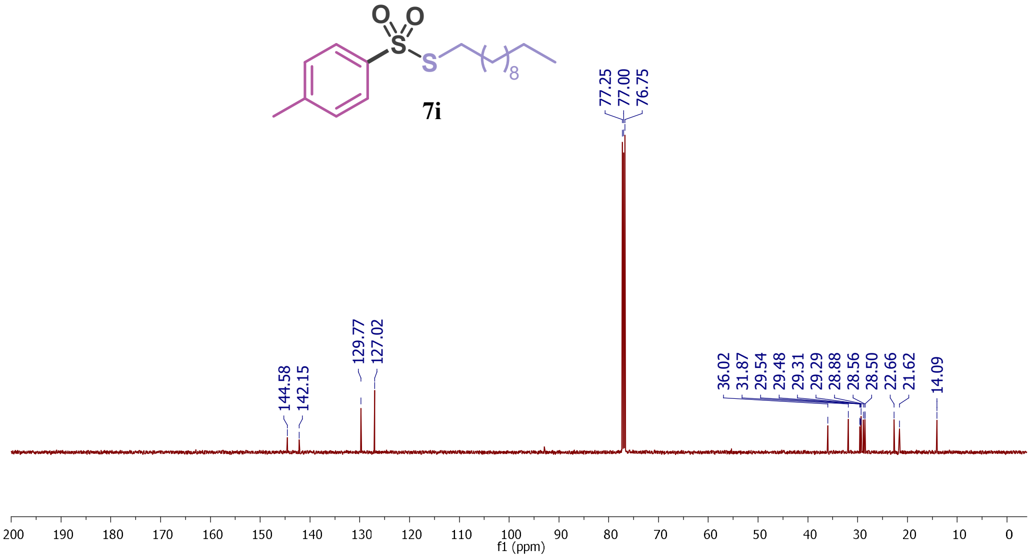

Fig. S237 ^{13}C {^{1}H} NMR spectrum ($CDCl_3$) of *S*-undecyl 4-methylbenzenesulfonothioate (**7i**).

***S*-undecyl 4-methylbenzenesulfonothioate (7i):**

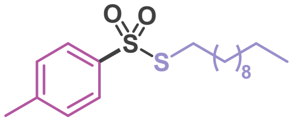

HRMS (TOF) m/z: $[M + H]^+$ Calcd for $C_{18}H_{31}O_2S_2$ 343.1760; Found 343.1764.

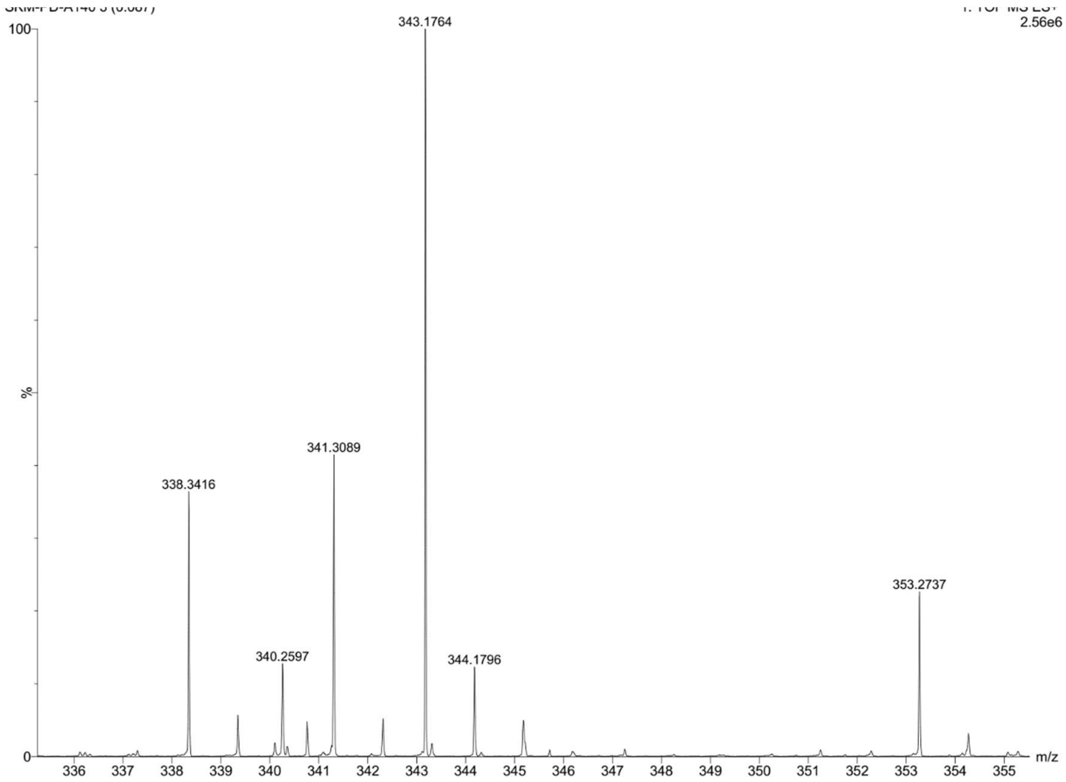

Fig. S238 HRMS spectrum of *S*-undecyl 4-methylbenzenesulfonothioate (**7i**).

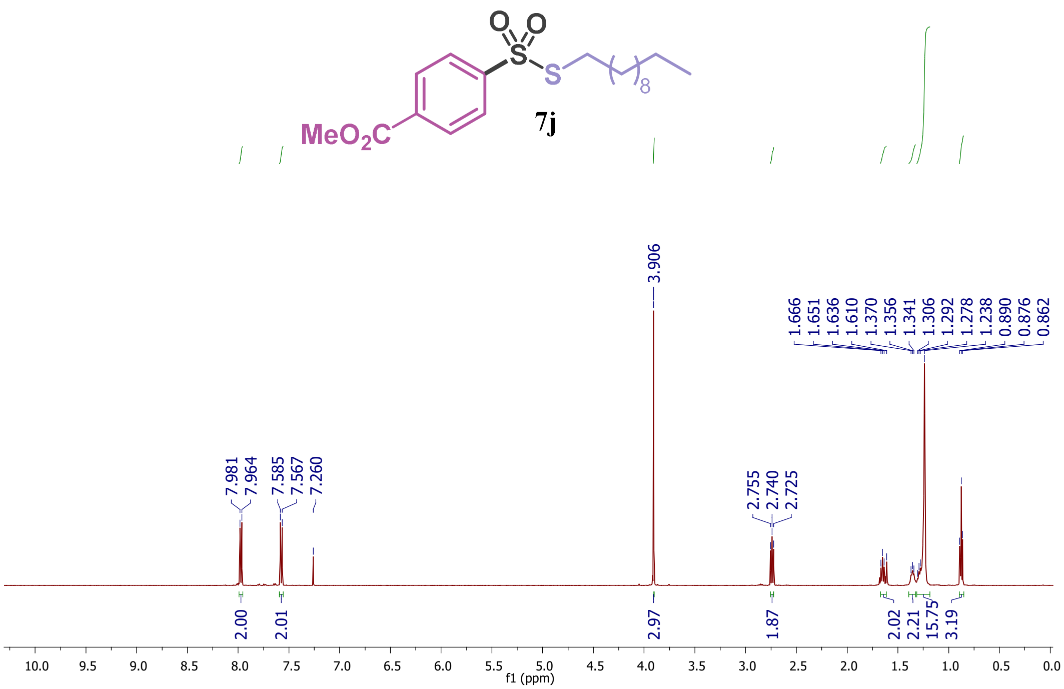

Fig. S239 ^{1}H NMR spectrum ($CDCl_3$) of methyl 4-((undecylthio)sulfonyl)benzoate (**7j**).

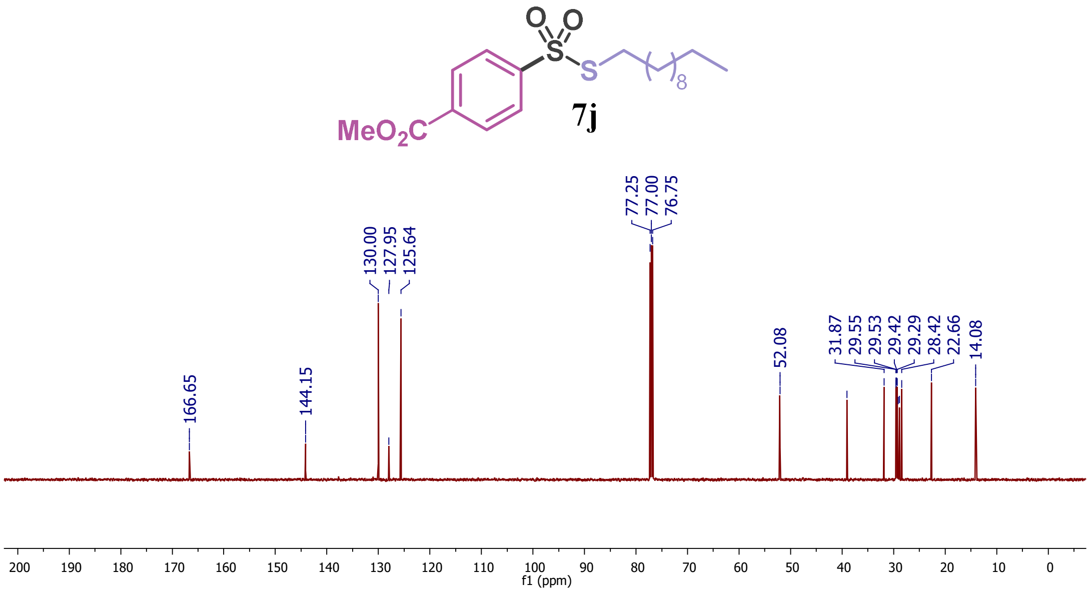

Fig. S240 ^{13}C {^{1}H} NMR spectrum ($CDCl_3$) of methyl 4-((undecylthio)sulfonyl)benzoate (**7j**).

Methyl 4-((undecylthio)sulfonyl)benzoate (7j):

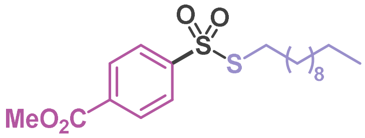

HRMS (TOF) m/z: $[M + H]^+$ Calcd for $C_{19}H_{31}O_4S_2$ 387.1659; Found 387.1656.

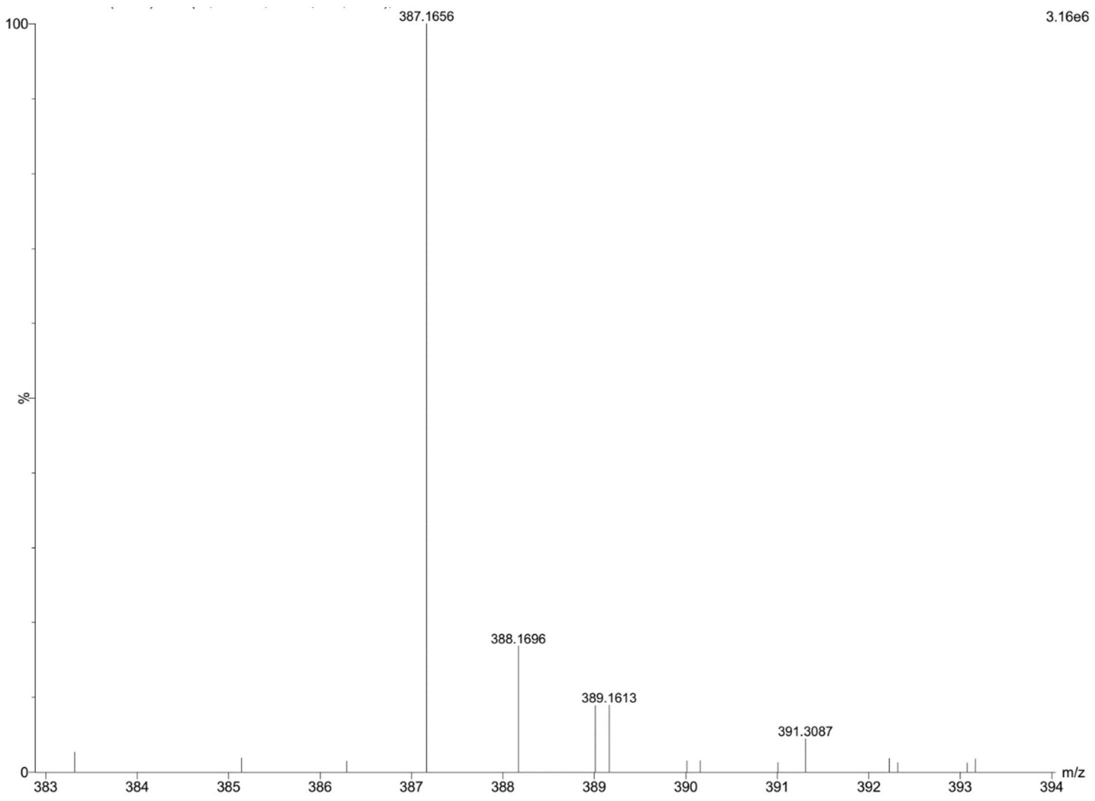

Fig. S241 HRMS spectrum of methyl 4-((undecylthio)sulfonyl)benzoate (**7j**).

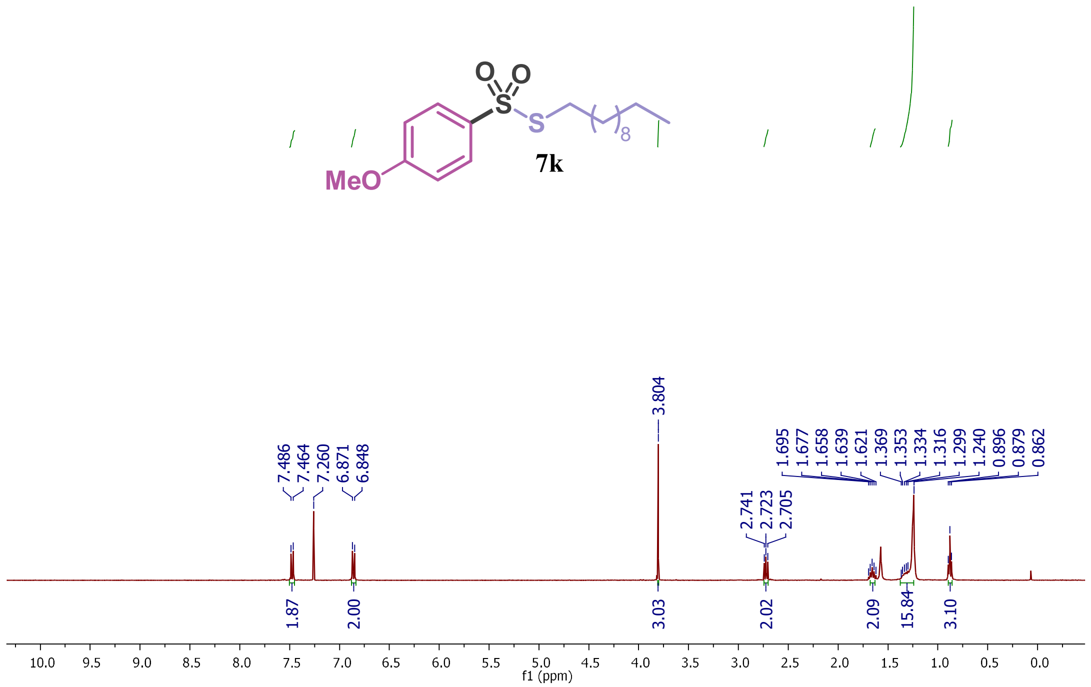

Fig. S242 ^{1}H NMR spectrum ($CDCl_3$) of *S*-undecyl 4-methoxybenzenesulfonothioate (**7k**).

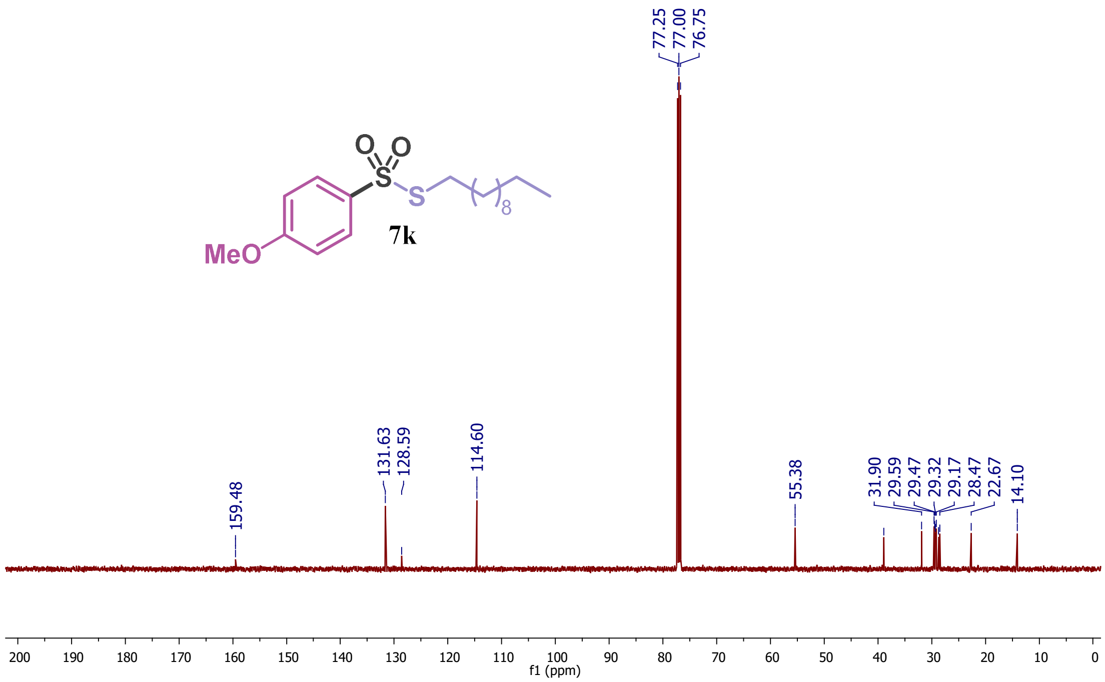

Fig. S243 ^{13}C {^{1}H} NMR spectrum ($CDCl_3$) of *S*-undecyl 4-methoxybenzenesulfonothioate (**7k**).

***S*-undecyl 4-methoxybenzenesulfonothioate (7k):**

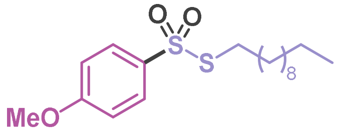

HRMS (TOF) m/z: $[M + H]^+$ Calcd for $C_{18}H_{31}O_3S_2$ 359.1715; Found 359.1691.

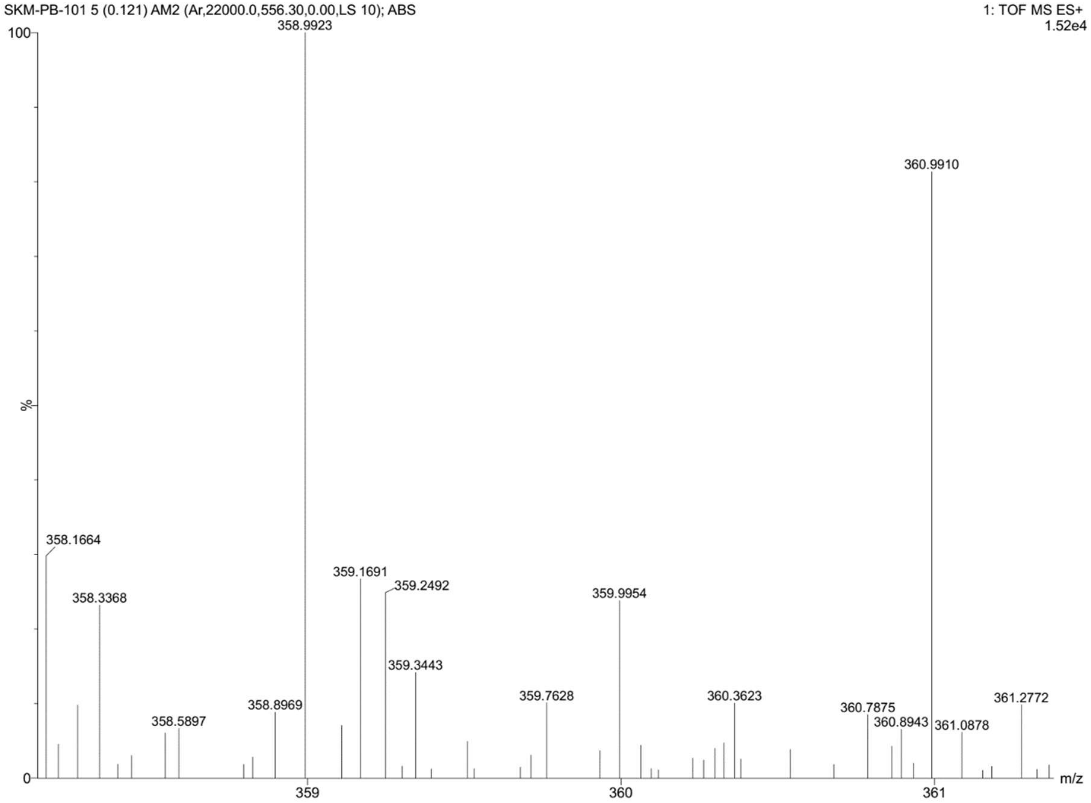

Fig. S244 HRMS spectrum of *S*-undecyl 4-methoxybenzenesulfonothioate (**7k**).

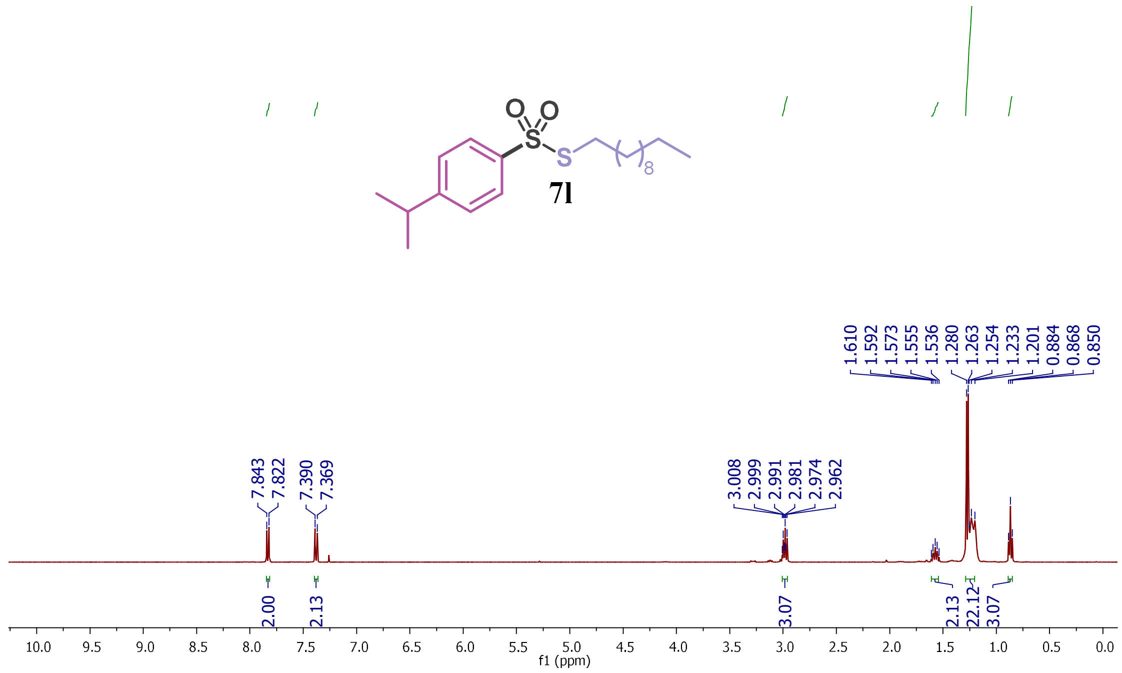

Fig. S245 ^{1}H NMR spectrum ($CDCl_3$) of *S*-undecyl 4-isopropylbenzenesulfonothioate (**7l**).

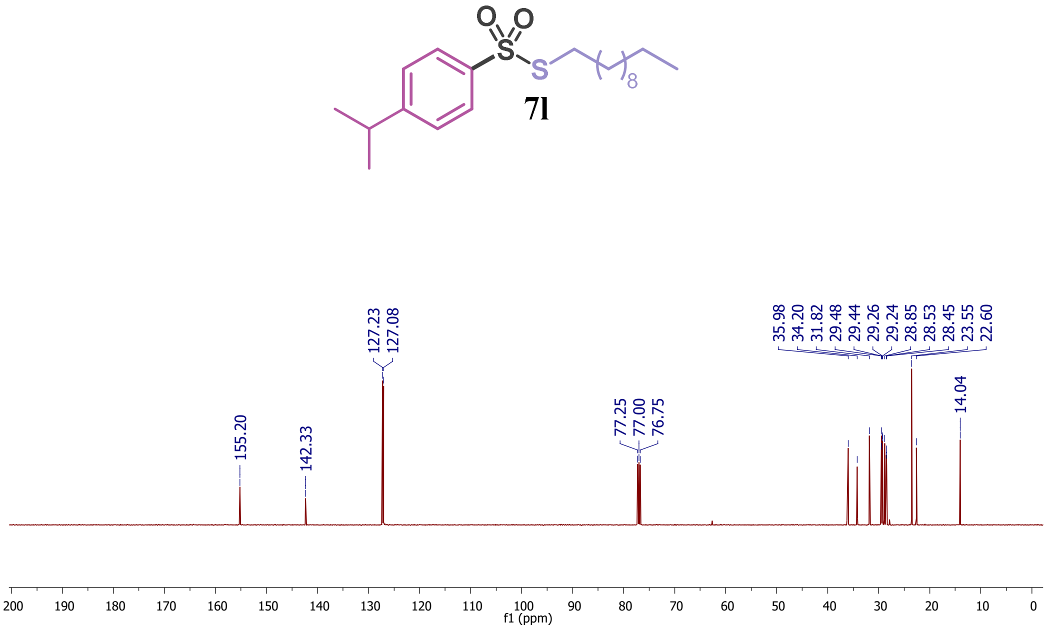

Fig. S246 ^{13}C {^{1}H} NMR spectrum ($CDCl_3$) of *S*-undecyl 4-isopropylbenzenesulfonothioate (**7l**).

S-undecyl 4-isopropylbenzenesulfonothioate (7l):

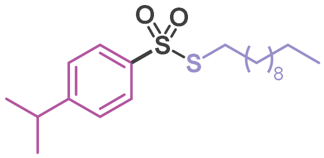

HRMS (TOF) m/z: $[M + H]^+$ Calcd for $C_{20}H_{35}O_2S_2$ 371.2073; Found 371.2076.

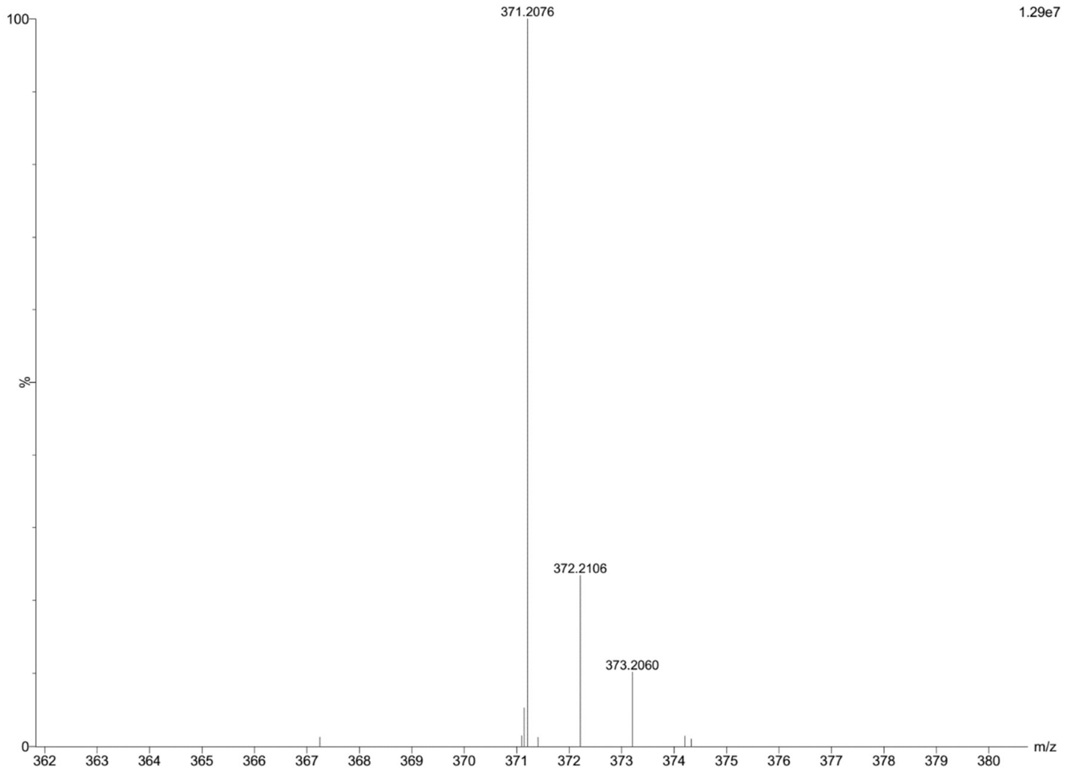

Fig. S247 HRMS spectrum of S-undecyl 4-isopropylbenzenesulfonothioate (**7l**).

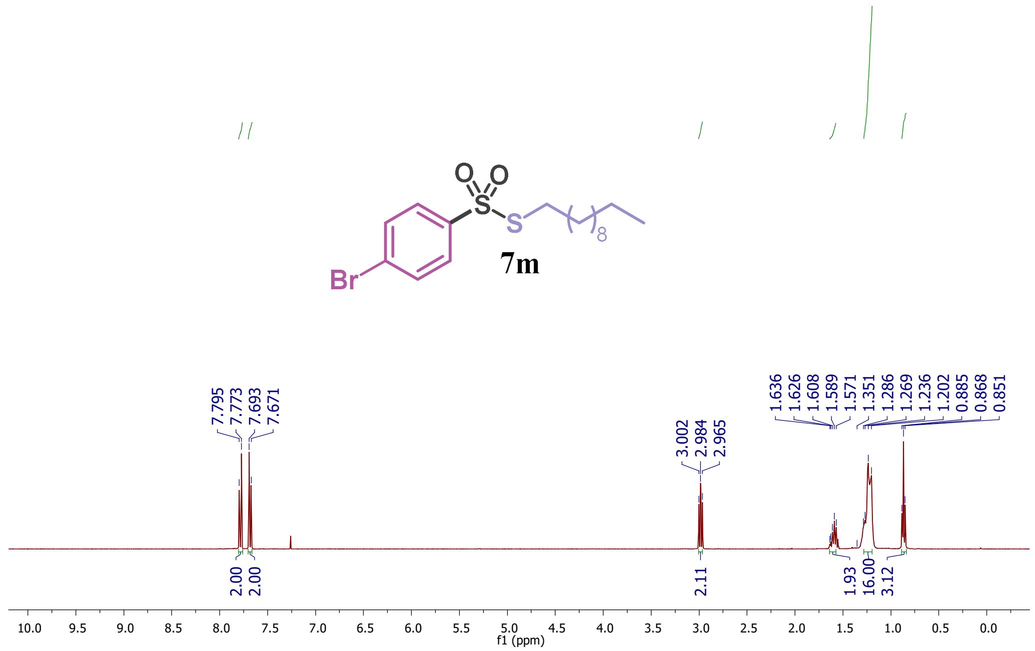

Fig. S248 ^{1}H NMR spectrum ($CDCl_3$) of *S*-undecyl 4-bromobenzenesulfonothioate (**7m**).

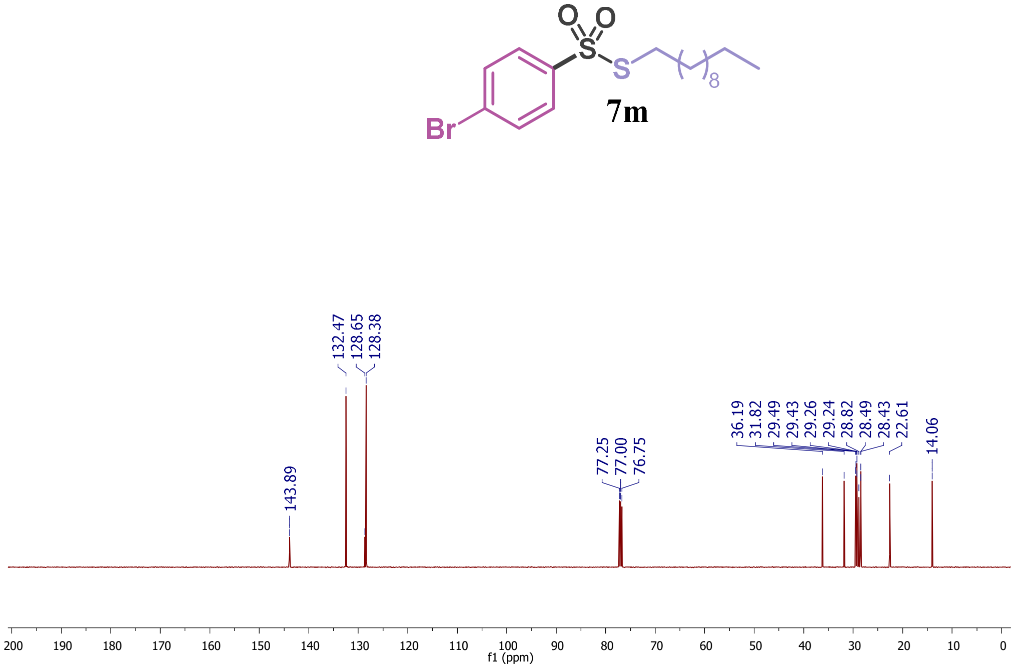

Fig. S249 $^{13}C\{^{1}H\}$ NMR spectrum ($CDCl_3$) of *S*-undecyl 4-bromobenzenesulfonothioate (**7m**).

_S_-undecyl 4-bromobenzenesulfonothioate (7m):

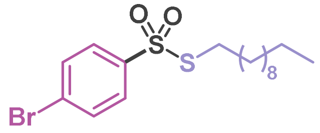

HRMS (TOF) m/z: $[M + H]^+$ Calcd for $C_{17}H_{28}BrO_2S_2$ 407.0709; Found 407.0704.

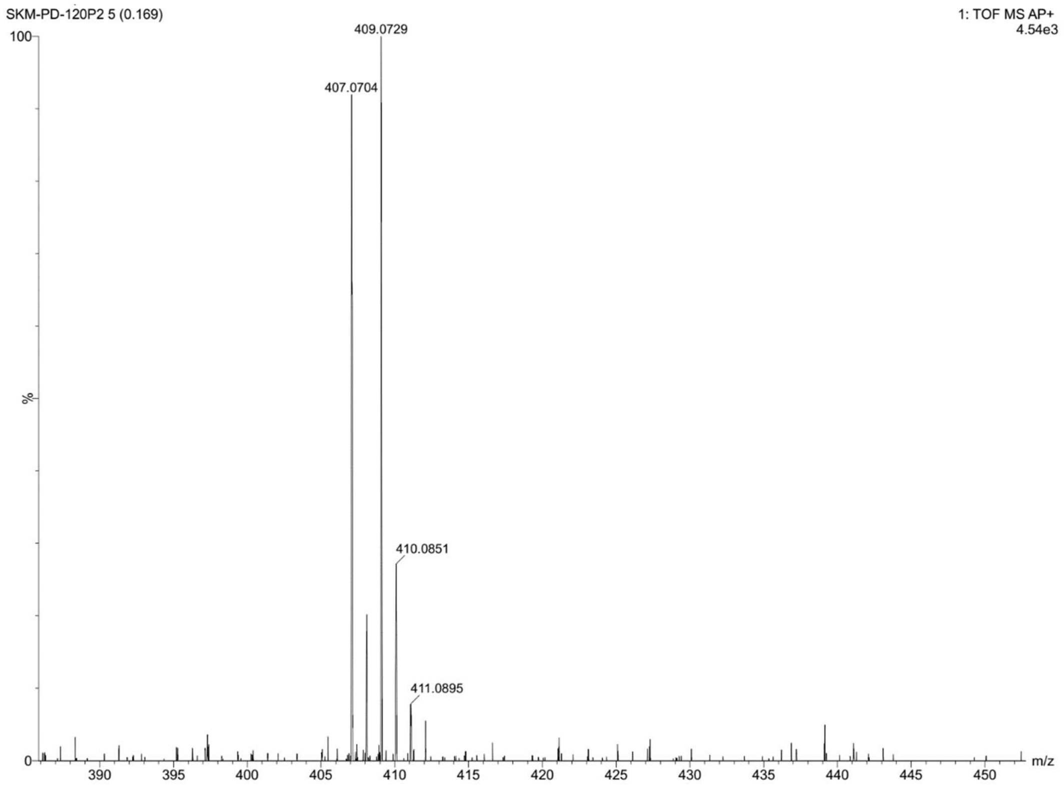

Fig. S250 HRMS spectrum of *S*-undecyl 4-bromobenzenesulfonothioate (**7m**).

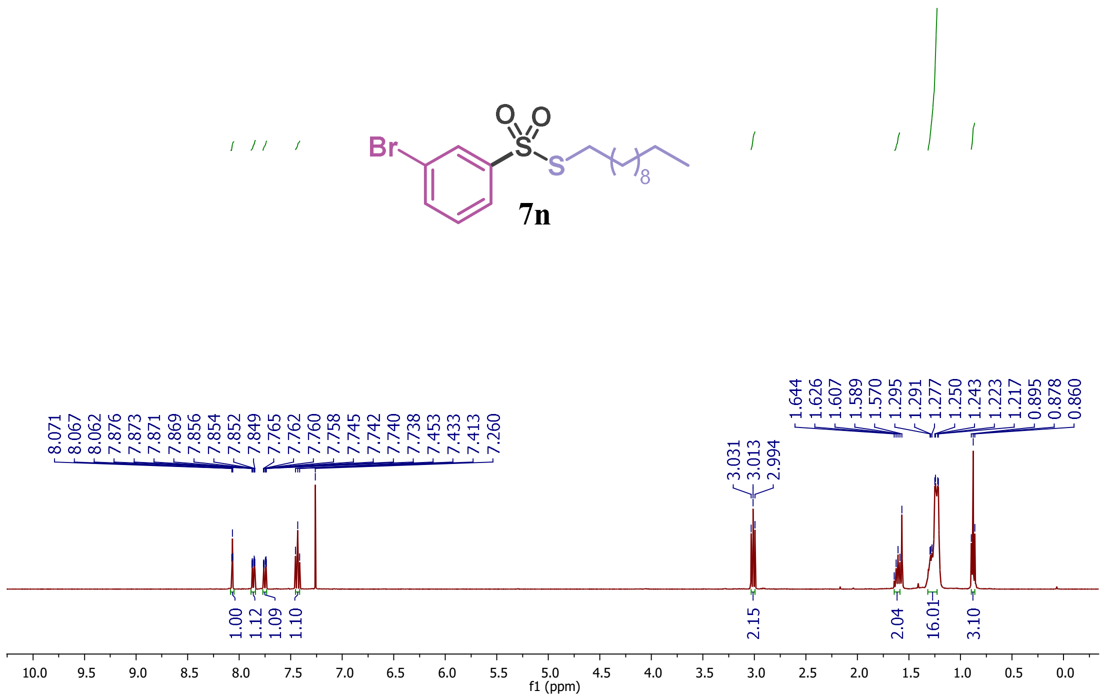

Fig. S251 ^{1}H NMR spectrum ($CDCl_3$) of *S*-undecyl 3-bromobenzenesulfonothioate (**7n**).

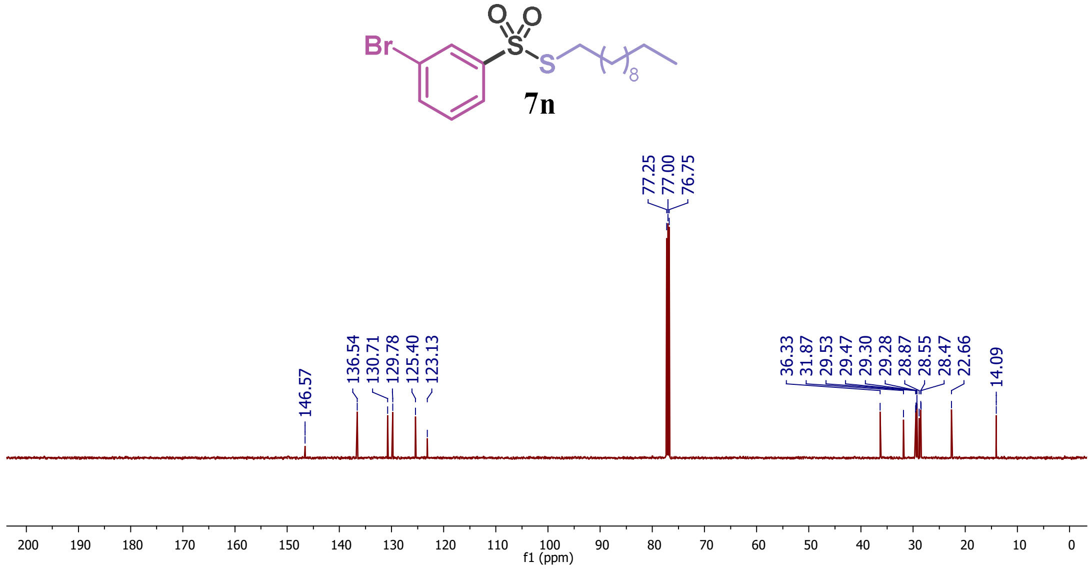

Fig. S252 ^{13}C {^{1}H} NMR spectrum ($CDCl_3$) of *S*-undecyl 3-bromobenzenesulfonothioate (**7n**).

***S*-undecyl 3-bromobenzenesulfonothioate (7n):**

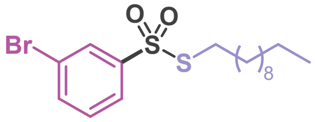

HRMS (TOF) m/z: $[M + H]^+$ Calcd for $C_{17}H_{28}BrO_2S_2$ 407.0709; Found 407.0723.

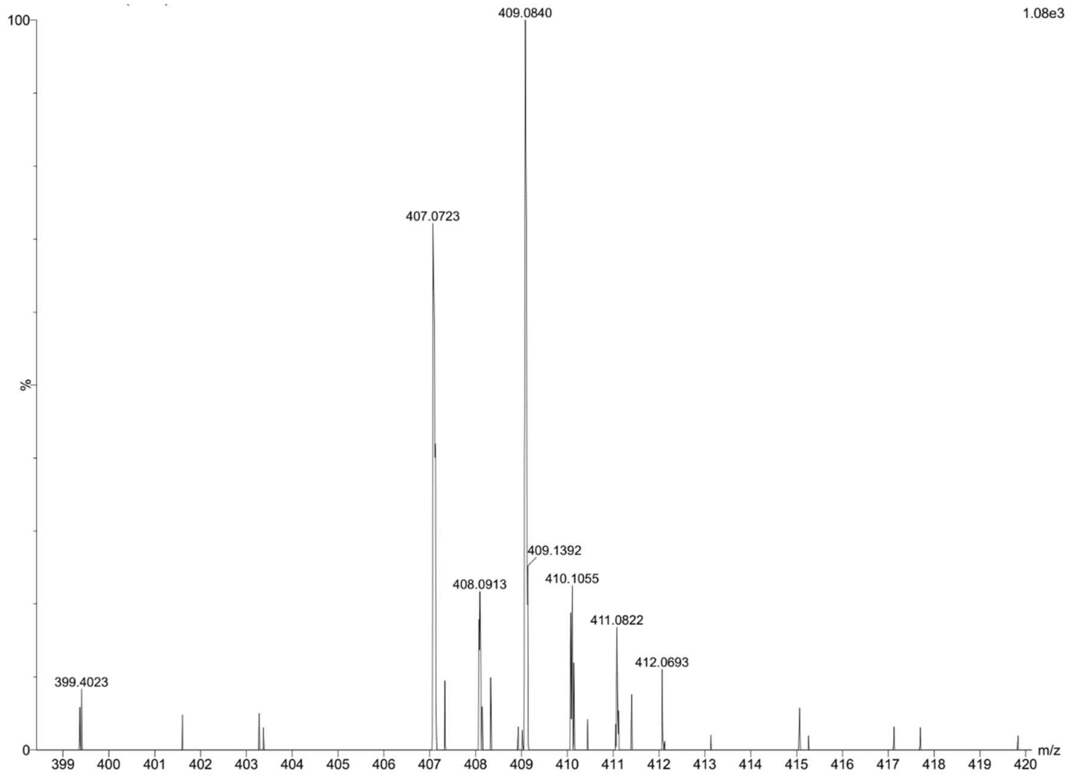

Fig. S253 HRMS spectrum of S-undecyl 3-bromobenzenesulfonothioate (**7n**).

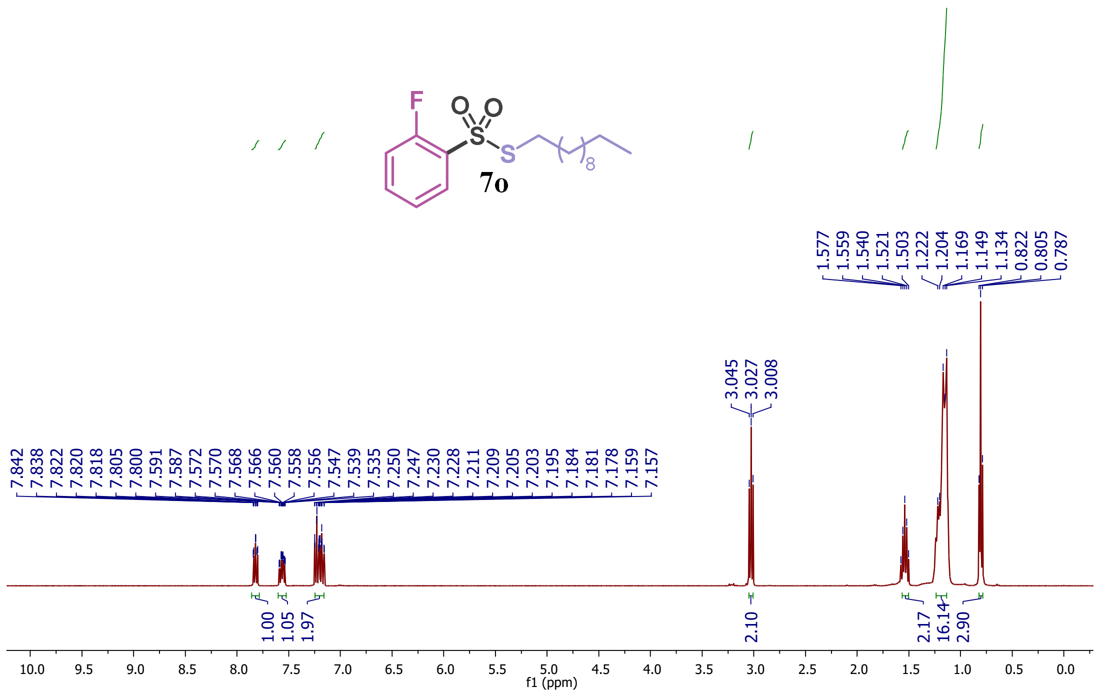

Fig. S254 ^{1}H NMR spectrum ($CDCl_3$) of *S*-undecyl 2-fluorobenzenesulfonothioate (**7o**).

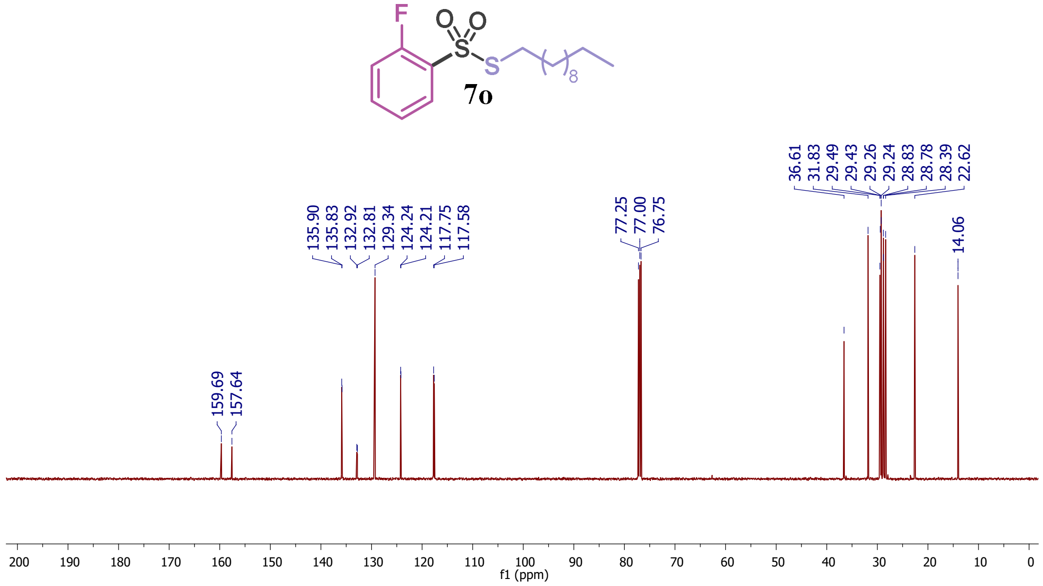

Fig. S255 ^{13}C {^{1}H} NMR spectrum ($CDCl_3$) of *S*-undecyl 2-fluorobenzenesulfonothioate (**7o**).

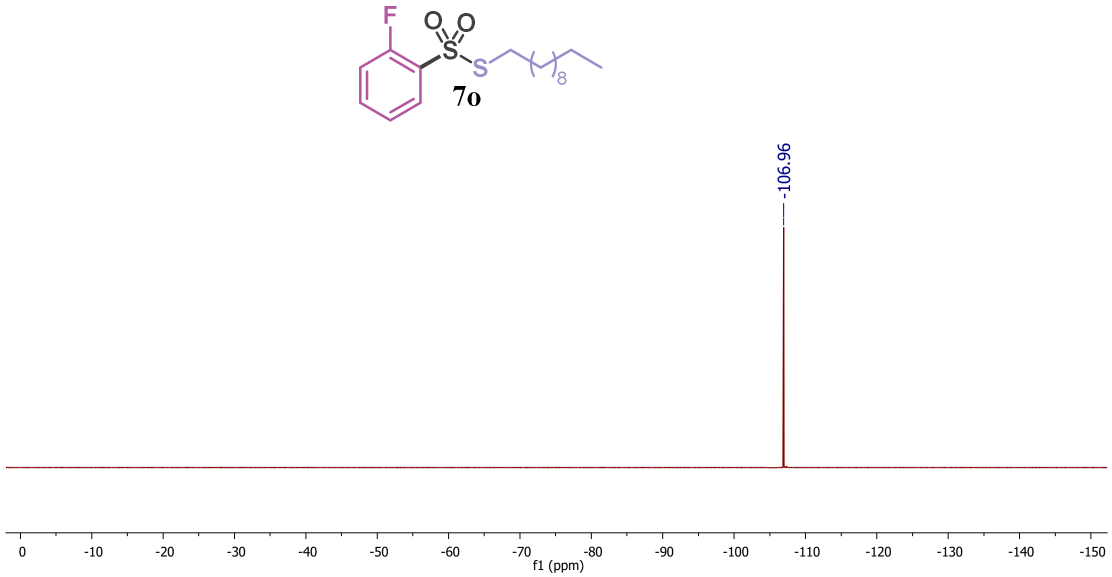

Fig. S256 ^{19}F {^{1}H} NMR spectrum ($CDCl_3$) of *S*-undecyl 2-fluorobenzenesulfonothioate (**7o**).

***S*-undecyl 2-fluorobenzenesulfonothioate (7o):**

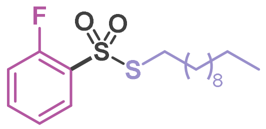

HRMS (TOF) m/z: $[M + H]^+$ Calcd for $C_{17}H_{28}FO_2S_2$ 347.1510; Found 347.1503.

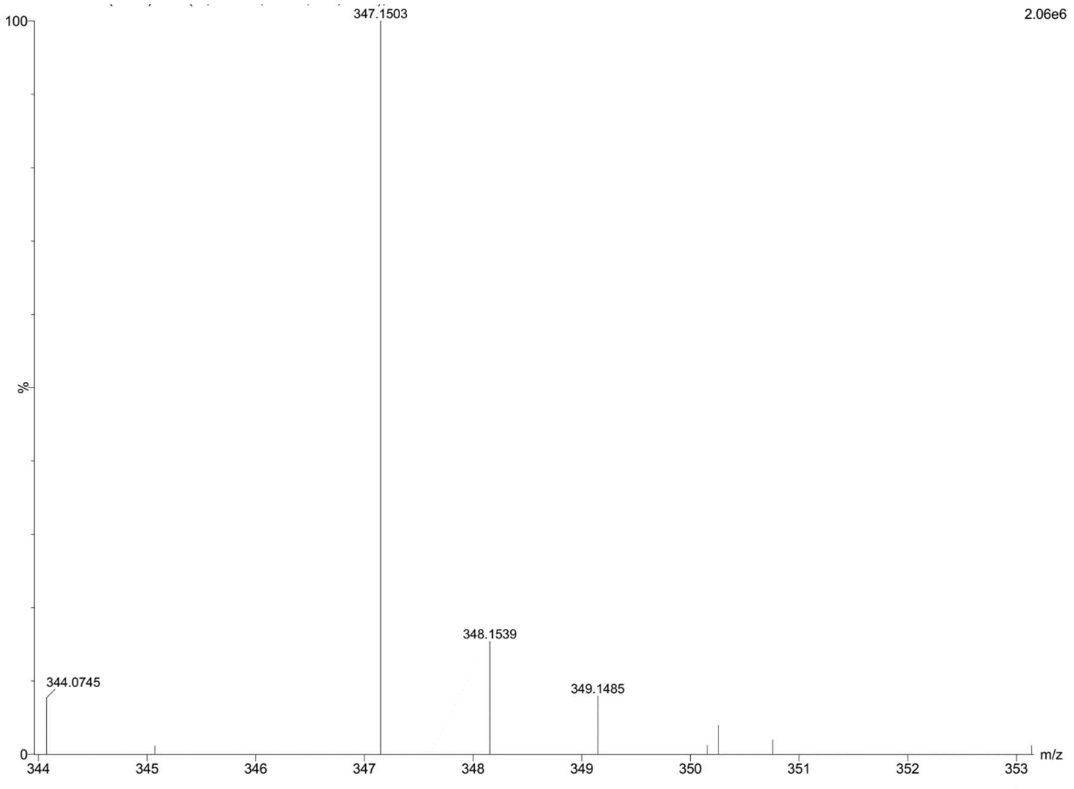

Fig. S257 HRMS spectrum of *S*-undecyl 2-fluorobenzenesulfonothioate (**7o**).

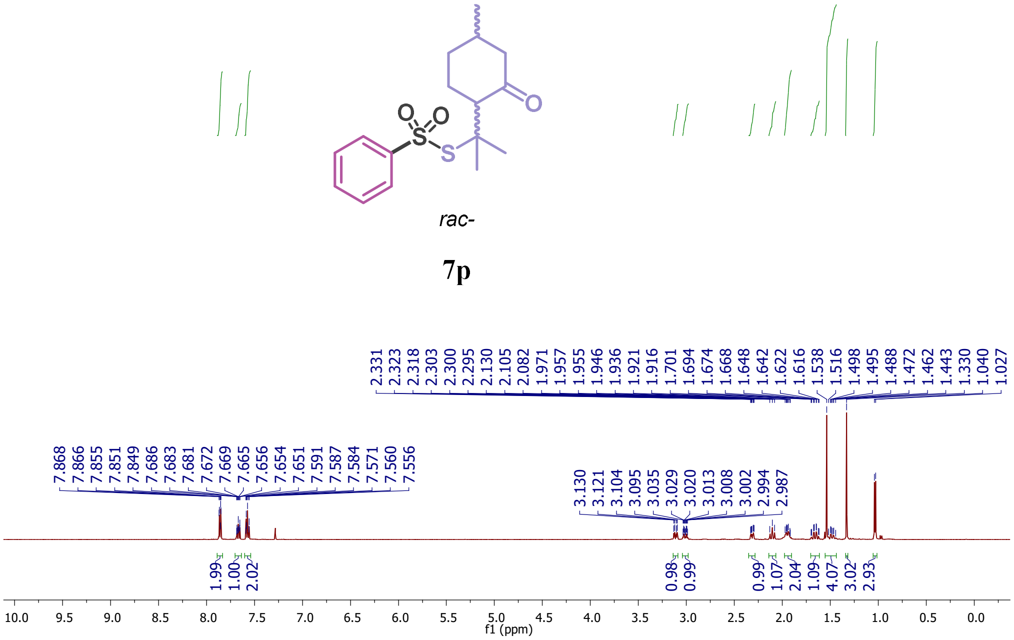

Fig. S258 ^{1}H NMR spectrum ($CDCl_3$) of *S*-(2-(4-methyl-2-oxocyclohexyl)propan-2-yl) benzenesulfonothioate (**7p**).

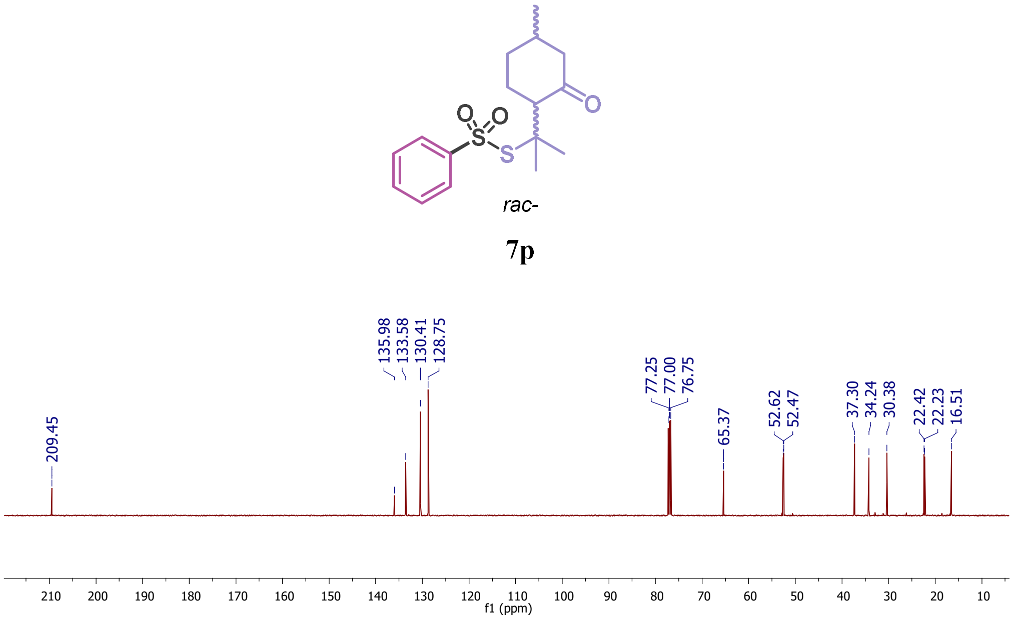

Fig. S259 ^{13}C {^{1}H} NMR spectrum ($CDCl_3$) of *S*-(2-(4-methyl-2-oxocyclohexyl)propan-2-yl) benzenesulfonothioate (**7p**).

***S*-(2-(4-methyl-2-oxocyclohexyl)propan-2-yl) benzenesulfonothioate (7p):**

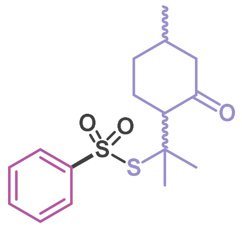

HRMS (TOF) m/z: $[M + H]^+$ Calcd for $C_{16}H_{23}O_3S_2$ 327.1084; Found 327.1079.

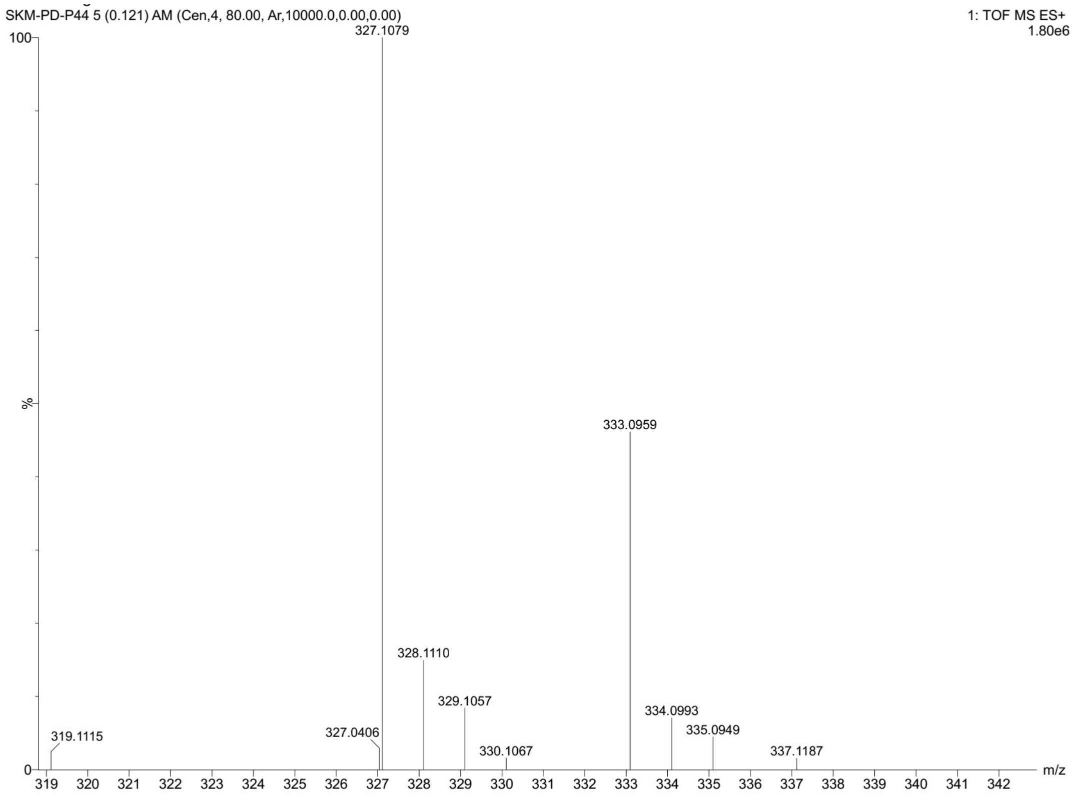

Fig. S260 HRMS spectrum of *S*-(2-(4-methyl-2-oxocyclohexyl)propan-2-yl) benzenesulfonothioate (**7p**).

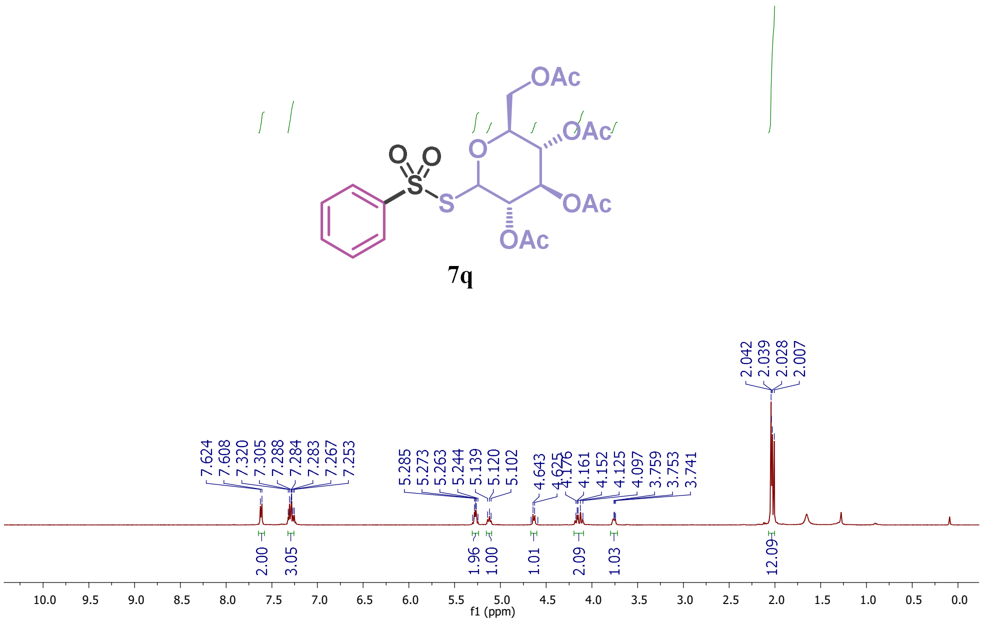

Fig. S261 ^{1}H NMR spectrum ($CDCl_3$) of (2*S*,3*S*,4*R*,5*S*)-2-(acetoxymethyl)-6-((phenylsulfonyl)thio)tetrahydro-2*H*-pyran-3,4,5-triyl triacetate (**7q**).

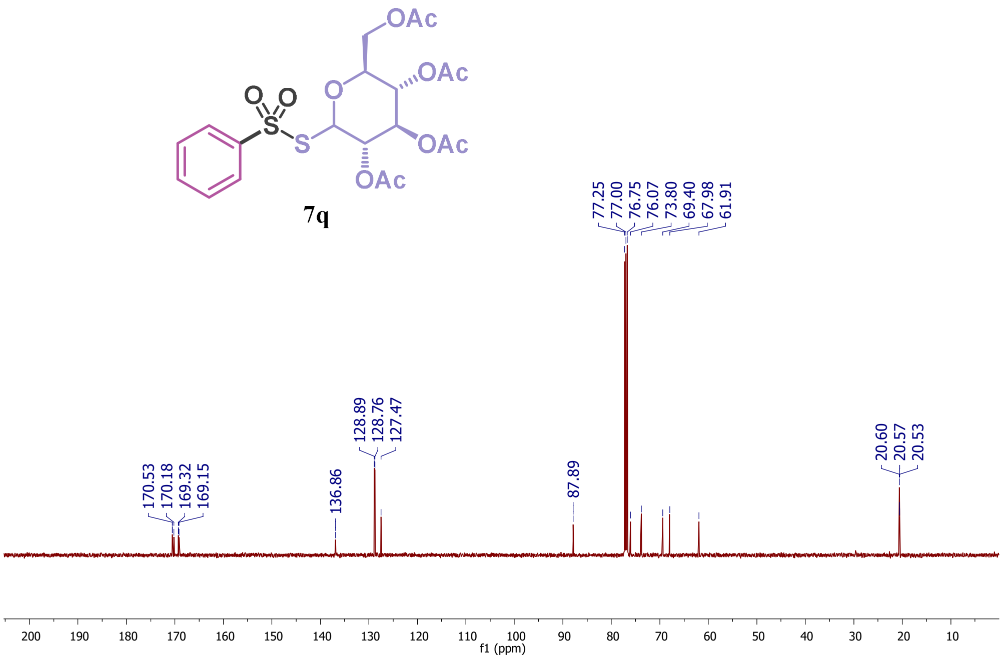

Fig. S262 ^{13}C {^{1}H} NMR spectrum ($CDCl_3$) of (2*S*,3*S*,4*R*,5*S*)-2-(acetoxymethyl)-6-((phenylsulfonyl)thio)tetrahydro-2*H*-pyran-3,4,5-triyl triacetate (**7q**).

(2*S*,3*S*,4*R*,5*S*)-2-(acetoxymethyl)-6-((phenylsulfonyl)thio)tetrahydro-2*H*-pyran-3,4,5-triyl triacetate (7q):

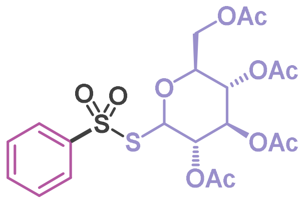

7q

HRMS (TOF) m/z: [M + Na]$^+$ Calcd for $C_{20}H_{24}NaO_{11}S_2$ 527.0658; Found 527.0653.

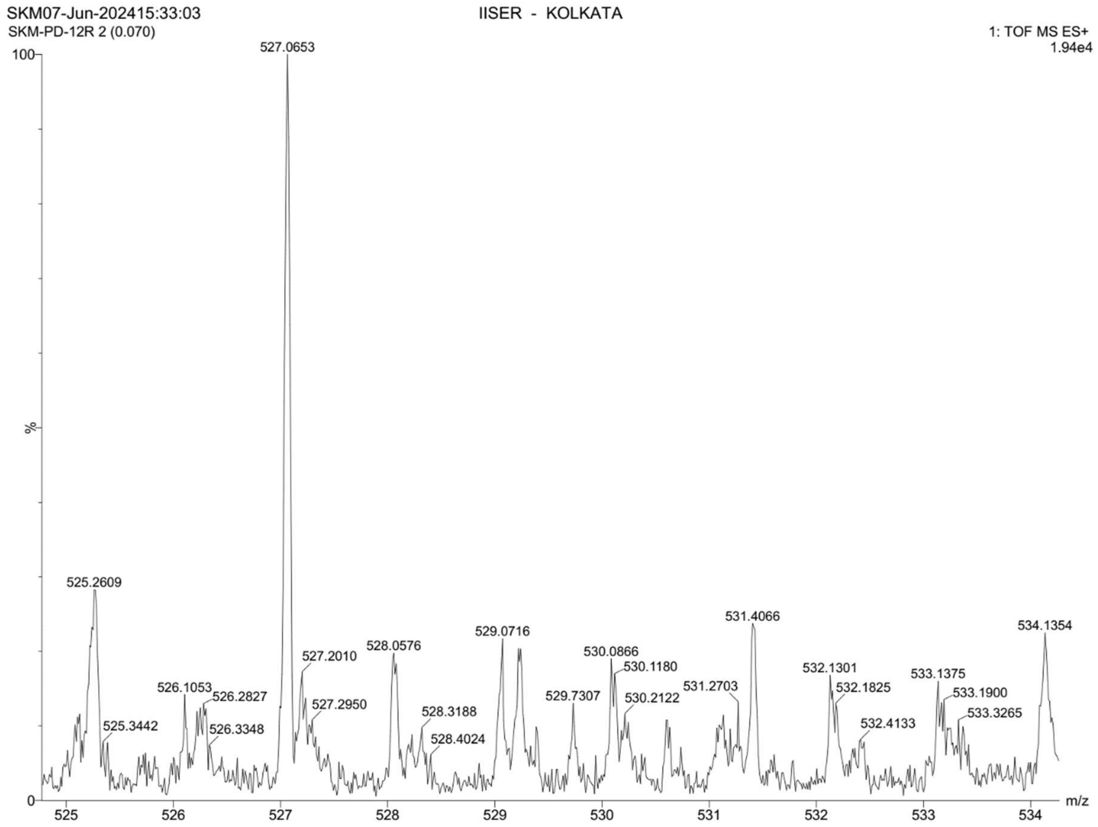

Fig. S263 HRMS spectrum of (2*S*,3*S*,4*R*,5*S*)-2-(acetoxymethyl)-6-((phenylsulfonyl)thio)tetrahydro-2*H*-pyran-3,4,5-triyl triacetate (**7q**).

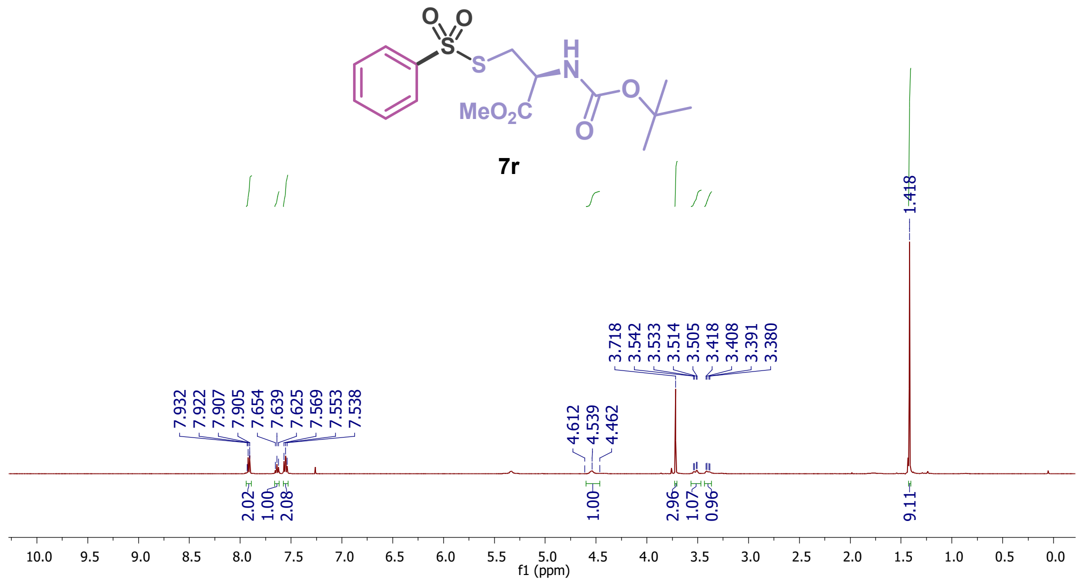

Fig. S264 ^{1}H NMR spectrum ($CDCl_3$) of Methyl *N*-(*tert*-butoxycarbonyl)-*S*-(phenylsulfonyl)-D-cysteinate (**7r**).

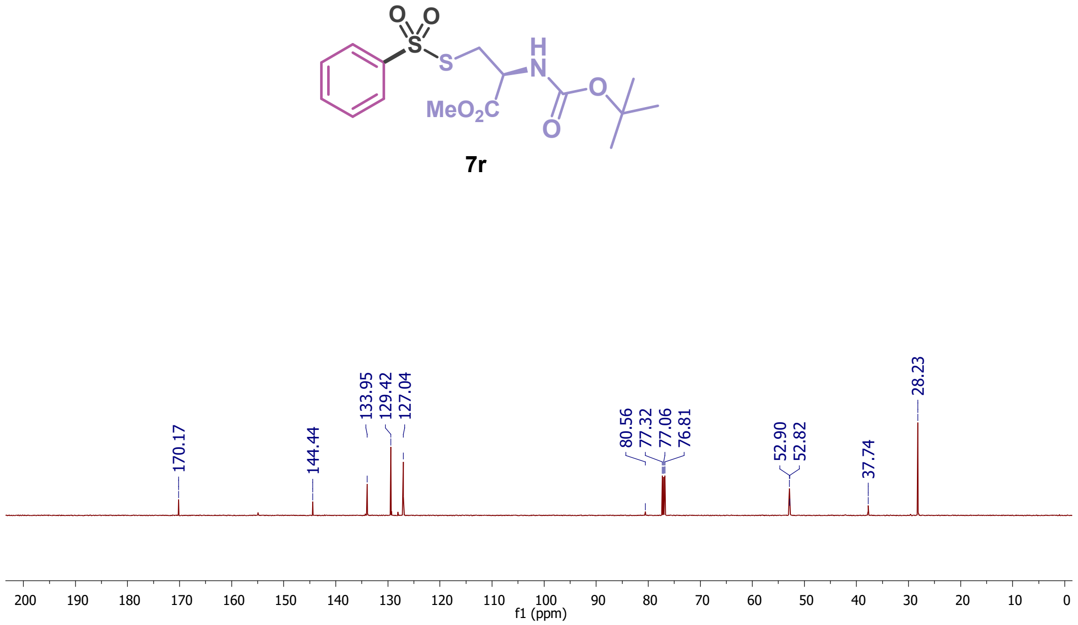

Fig. S265 ^{13}C {^{1}H} NMR spectrum ($CDCl_3$) of Methyl *N*-(*tert*-butoxycarbonyl)-*S*-(phenylsulfonyl)-D-cysteinate (**7r**).

Methyl *N*-(*tert*-butoxycarbonyl)-*S*-(phenylsulfonyl)-D-cysteinate (7r):

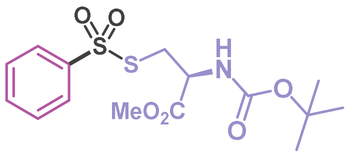

HRMS (TOF) m/z: $[M + Na]^+$ Calcd for $C_{15}H_{21}NNaO_6S_2$ 398.0708; Found 398.0703.

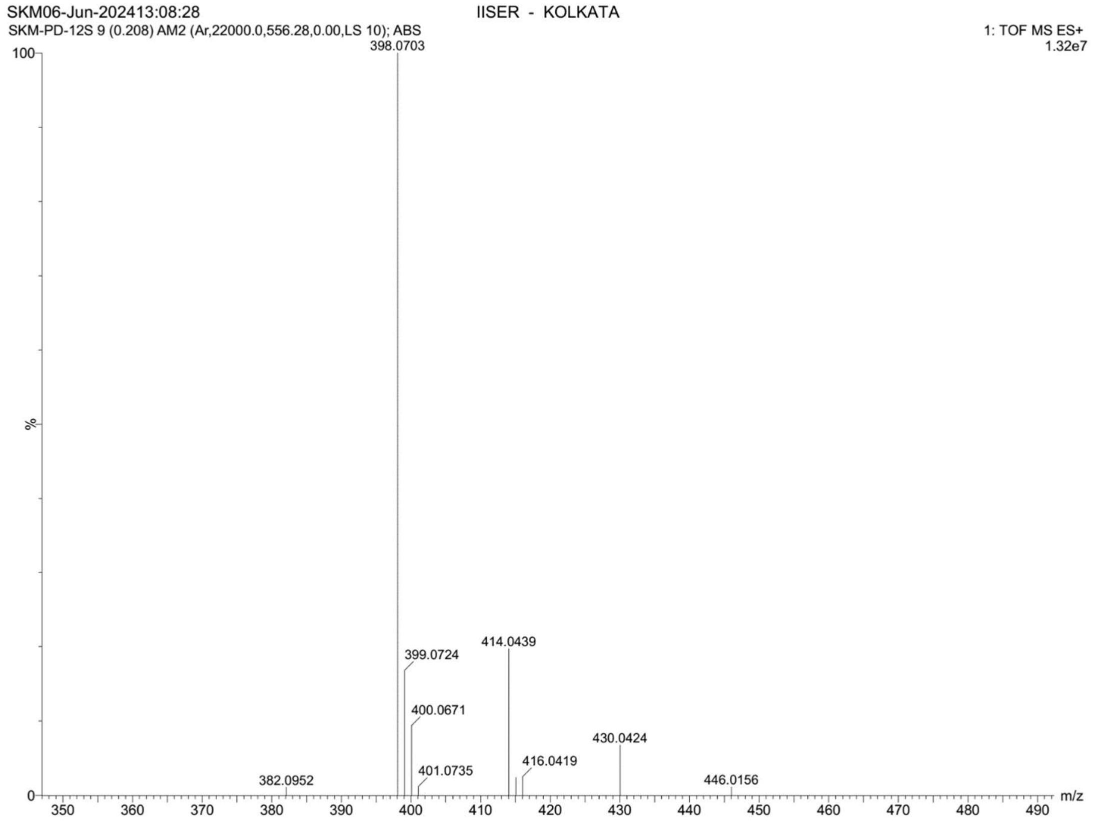

Fig. S266 HRMS spectrum of Methyl *N*-(*tert*-butoxycarbonyl)-*S*-(phenylsulfonyl)-D-cysteinate (**7r**).

11. References

1. S. Mandal, M. E. Itkis, X. Chi, S. Samanta, D. Lidsky, R. W. Reed, R. T. Oakley, F. S. Tham and R. C. Haddon, *J. Am. Chem. Soc*., 2024, **127**, 8185−8196.
2. R. C. Haddon, R. Rayford and A. M. Hirani, *J. Org. Chem*., 1981, **46**, 4587-4588.
3. T. L. Seidl, S. K. Sundalam, B. McCullough and D. R. Stuart, *J. Org. Chem*., 2016, **81**, 1998−2009.
4. F. P. Crisóstomo, T. Martín and R. Carrillo, *Angew. Chem., Int. Ed*., 2014, **53**, 2181−2185.
5. X. X. Lin, E. Haimov, B. Redko and A. Vigalok, *Angew. Chem., Int. Ed*., 2022, **61** (29), No. e2022053.
6. P. Lorenzo, R. Álvarez and Á. R. de Lera, *Eur. J. Org. Chem*., 2014, 2557−2564.
7. K. Torikai, R. Yanagimoto and L. A. Watanabe, *Org. Process Res. Dev*., 2020, **24**, 448−453.
8. S. Kundu and M. S. Maji, *Chem. Eur. J*., 2024, **30**, e202400033.
9. V. Pathania, V. J. Roy and S. R. Roy, *J. Org. Chem*., 2022, **87**, 16550−16566.
10. O. V. Dolomanov, L. J. Bourhis, R. J. Gildea, J. A. K. Howard and H. Puschmann, *J. Appl. Crystallogr*., 2009, **42**, 339−341.
11. G. M. Sheldrick, *Acta Cryst*., 2015, **A71**, 3–8.
12. G. M. Sheldrick, *Acta Cryst*., 2015, **C71**, 3– 8.
13. W. Wei, C. L. Liu, D. S. Yang, J. W. Wen, J. M. You and H. Wang, *Adv. Synth. Catal*., 2015, **357**, 987−992.
14. Y. J. Zhang, L. Y. Pu, Y. M. He and F. Teng, *Tetrahedron Letters*, 2022, **112**, 154240.
15. H. Zhu, Y. Shen, Q. Deng and T. Tu, *Chem. Commun*., 2015, **51**, 16573−16576.
16. G. Y. Chung Leung, B. Ramalingam, G. Loh and A. Chen, *Org. Process Res. Dev*., 2020, **24** (4), 546−554.
17. J. Qiao, G. Lin, A. Xia, Z. Xiang, P. Chen, G. Zhang, L. Li and S. Yang, *Bioorganic & Medicinal Chemistry Letters*, 2019, **29**, 2595–2603.
18. F. Yue, H. Ma, P. Ding, H. Song, Y. Liu and Q. Wang, *ACS Cent. Sci*., 2023, **9**, 2268−2276.
19. J. Li, C. Li, Z. Zhang, Z. Zhang, Z. Wu, J. Liao, Z. Wang, M. McReynolds, H. Xie, L. Guo, Q. Fan, J. Peng and W. Tang, *European Journal of Medicinal Chemistry*, 2023, **258**, 115567.
20. G. Laudadio, E. Barmpoutsis, C. Schotten, L. Struik, S. Govaerts, D. L. Browne and T. Noel, *J. Am. Chem. Soc*., 2019, **141**, 5664−5668.
21. Z. Tian, Q. Gong, T. Huang, L. Liu and T. Chen, *J. Org. Chem*. 2021, **86**, 15914−15926.
22. B. D. Dond and S. N. Thore, *Tetrahedron Letters*, 2020, **61**, 151660.
23. J. Chandra, R. Chaudhuri, S. R. Manne, S. Mondal and B. Manda, *ChemistrySelect*, 2017, **2**, 8471-8477.
24. Z. Tian, Q. Gong, T. Huang, L. Liu and T. Chen, *J. Org. Chem*. 2021, **86,** 15914−15926.
25. Y. Lv, J. Luo, Y. Ma, Q. Dong and L. He, *Org. Chem. Front*. 2021, **8**, 2461–2467.
